# Predation and fragmentation portrayed in the statistical structure of prey time series

**DOI:** 10.1186/1472-6785-9-10

**Published:** 2009-05-06

**Authors:** Ditte K Hendrichsen, Chris J Topping, Mads C Forchhammer

**Affiliations:** 1 Department of Arctic Environment, National Environmental Research Institute, Aarhus University, Frederiksborgvej 399, DK-4000 Roskilde, Denmark; 2 Department of Population Biology, Institute of Biology, University of Copenhagen, Universitetsparken 15, DK-2100 Copenhagen, Denmark; 3 Department of Wildlife Ecology and Biodiversity, National Environmental Research Institute, Aarhus University, Grenåvej 12, DK-8410 Rønde, Denmark

## Abstract

**Background:**

Statistical autoregressive analyses of direct and delayed density dependence are widespread in ecological research. The models suggest that changes in ecological factors affecting density dependence, like predation and landscape heterogeneity are directly portrayed in the first and second order autoregressive parameters, and the models are therefore used to decipher complex biological patterns. However, independent tests of model predictions are complicated by the inherent variability of natural populations, where differences in landscape structure, climate or species composition prevent controlled repeated analyses. To circumvent this problem, we applied second-order autoregressive time series analyses to data generated by a realistic agent-based computer model. The model simulated life history decisions of individual field voles under controlled variations in predator pressure and landscape fragmentation. Analyses were made on three levels: comparisons between predated and non-predated populations, between populations exposed to different types of predators and between populations experiencing different degrees of habitat fragmentation.

**Results:**

The results are unambiguous: Changes in landscape fragmentation and the numerical response of predators are clearly portrayed in the statistical time series structure as predicted by the autoregressive model. Populations without predators displayed significantly stronger negative direct density dependence than did those exposed to predators, where direct density dependence was only moderately negative. The effects of predation versus no predation had an even stronger effect on the delayed density dependence of the simulated prey populations. In non-predated prey populations, the coefficients of delayed density dependence were distinctly positive, whereas they were negative in predated populations. Similarly, increasing the degree of fragmentation of optimal habitat available to the prey was accompanied with a shift in the delayed density dependence, from strongly negative to gradually becoming less negative.

**Conclusion:**

We conclude that statistical second-order autoregressive time series analyses are capable of deciphering interactions within and across trophic levels and their effect on direct and delayed density dependence.

## Background

Time series analyses are increasingly employed in ecological studies as a pivotal and powerful tool in population ecology, involving important aspects of conservation and climate change [1]. In particular, they are used to disentangle the relative importance of different factors in complex biological interactions across trophic levels [2,3]. For example, Bjørnstad *et al*. (1995) investigated the impact of specialist vs. generalist predators on density dependence of small rodents along a longitudinal gradient in Fennoscandia [4]. In a comprehensive review including multiple time series from microtine populations in both Fennoscandian and Hokkaido, Japan, Stenseth [5] investigated density dependent structure of the populations emphasizing how time series analyses can apparently separate intraspecific and interspecific interactions, with the two dimensional structure caused by either bottom-up (plant-herbivore) or top-down (predator-prey) interactions. In a recent review focusing on the northern microtine populations, Lima *et al*. [6] conclude, based on time series analyses, that the populations are characterized mainly by direct density-dependence, caused by intraspecific interactions and generalist predators, and less consistently by delayed density-dependence originating from delayed responses from food or specialist predators. These comparative studies of natural populations of microtines affected by different sets of multiple predators, emphasize that time series analyses are applicable in deciphering the impact of interactions within and across trophic levels as well as between different types of predator-prey interactions.

Overall, these studies clearly suggest that factors affecting population growth, density dependence and trophic interactions are directly mirrored in the autoregressive structure of the time series of population density [7]. Focusing on the predator-prey model formulation specifically, the interactions within and between species in a two-species predator-prey system can be described by a set of coupled equations:

(1)

(2)

where *N *and *P *denote the densities in year t of prey and predators respectively, *X*_*t *_and *Y*_*t *_are the log-transformed densities of prey and predators in year *t*, and *a*_*ji *_is the ecological interaction coefficient specifying the influence of predator species *j *on prey species *i *[for a detailed description of the model see 2–4]. If we take the natural logarithm on both sides of equation 1 and 2, rearrange and combine the equations into one, we obtain a description of the density of prey at time *t*, X_*t*_, as a function of intra- and interspecific interactions two time steps back:

(3)

Eq. 3 is equivalent to a second-order autoregressive model:

(4)

The model in eq. 3 and 4 predicts that the presence and functional type of predator leave specific traceable marks in the statistical structure of the prey time series: Whereas intraspecific interactions (*a*_*ii *_and *a*_*jj*_) will affect both 1+*b*_1 _and *b*_2_, changes in predator-prey interactions (*a*_*ji *_and *a*_*ij*_) will affect *b*_2 _but not 1+*b*_1 _(Table 1). Eq. 4 can be expressed in terms of density dependence, where the first and second order parameters (1+*b*_1 _and *b*_2_), corresponds to direct and delayed density dependence, respectively [2-4]. Analyses of the autocovariate structure of a time series should therefore in theory decipher the relative strength of intra- and interspecific interactions and their contributions to the density dependent structure of the population.

**Table 1:** The relationship between statistical density dependence in prey time series and the combination of ecological interaction coefficients.

Statistical density dependence	Ecological interaction coefficients			Ecological interpretation
		
	predator (*a*_*jj *_= *a*_*ji *_= 0)	+	predator^a^	
Direct: (1+*b*_1_)	2+*a*_*ii*_	<	2+*a*_*ii *_+ *a*_*jj*_	Stronger competition among non-predated than predated vole populations [8,9]
Delayed: *b*_2_	-*a*_*ii *_- 1	>	*a*_*ji*_*a*_*ij*_-*a*_*jj*_*a*_*ii*_- *a*_*ii*_- *a*_*jj*_-1	Predation of voles and increased feed-back from voles to predators [9,10]

^a ^Increased specialization of predator (generalist→intermediate→specialist) is expected to result in increasingly negative *b*_2 _because of the gradually increased negative influence on prey (*a*_*ji*_) and increased feed-back from prey (*a*_*ij*_) [4].

Quantitative investigations analyzing whether statistical time series structures unambiguously portray the changes in the interactions within and across trophic levels, as has been suggested [2-5], are however lacking. Natural data series usually cover relatively short time spans, originate from different geographical regions and are characterized by variable environmental settings and different combinations of prey and predator species. Clear statistical signals of the relative importance of inter- and intraspecific interactions in natural prey time series may thus obscured by a suite of factors preventing controlled replicable analyses.

We circumvented these inherent problems by applying the statistical time series analyses to long-term prey dynamics, independently generated in a realistic agent-based computer model (ABM; see ODD documentation below). The use of an ABM enables generation of independently replicated time series data from the same prey population originating from a high realistic setup of individuals interacting with conspecifics and their environment. Hence, ABM's allow investigation on how prey populations respond to different combinations of predator type and habitat fragmentation under controlled conditions.

Theory and lab experiments predict that the presence and functional type of predators (i.e., degree of specialization) affect the statistical autocovariate structure of prey time series [3-5,7]. Since predator effect on prey is influenced by environmental changes [11-13], we also expect changes in environmental conditions to be portrayed in the statistical structure of the prey populations. For example, habitat fragmentation affects predator efficiency [13-15] and the degree of intra-specific competition. Consequently, the model also predicts that any changes in fragmentation are expected to affect the 1+*b*_1 _and *b*_2 _of the prey time series. Here we demonstrate that specific, gradual changes in predator specialization and habitat fragmentation result in corresponding gradual changes of the statistical time series structure of prey dynamics.

## Results

**Figure 1:**
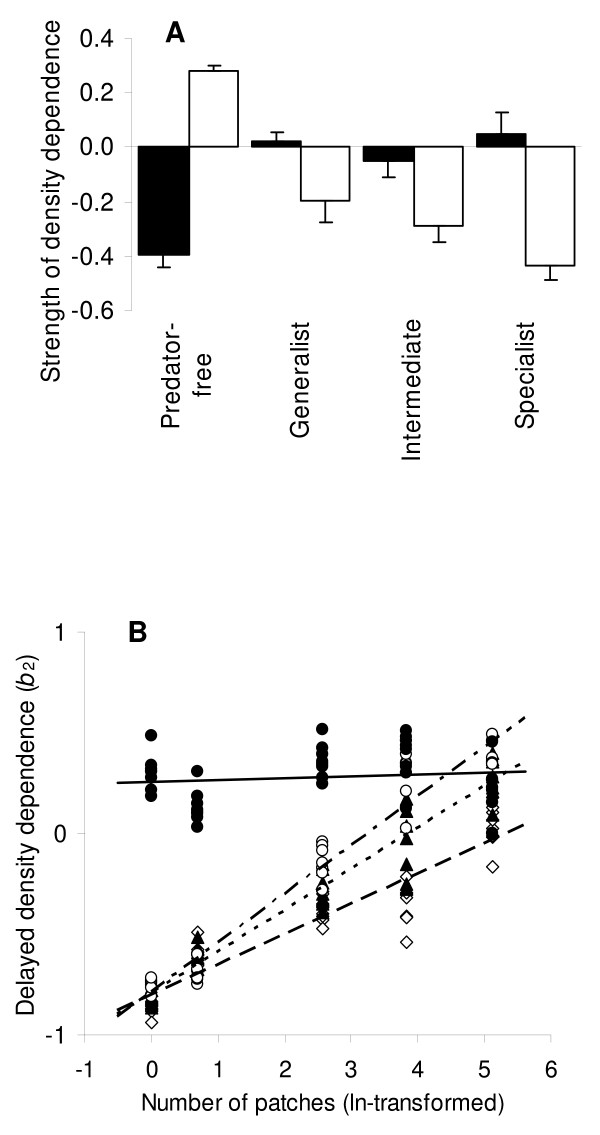
**Coefficients of direct and delayed density dependence**. A) The average autoregressive coefficients of direct (1+*b*_1_) (solid columns) and delayed (*b*_2_) (open columns) density dependence are given for each of the four predator treatments (n = 40 in each bar) Vertical lines given for each bar are standard error of means. B) The response in delayed density dependence, *b*_2_, to increasing fragmentation for each of the four predator treatments: Specialist-exposed (open diamonds), intermediate (solid triangles) generalist-exposed (open circles), and predator-free populations (solid circles). One point represents one time series, 160 in total. The corresponding linear regression lines for each predator treatment are denoted as: specialist (dashed line), intermediate (dotted line), generalists (dash/dotted line) and predator free (solid line). Regression lines explain 94%, 94%, 86% and <1% of the variation, respectively.

The autoregressive analyses of the time series from the simulated prey populations showed a marked difference between predated and non-predated populations with respect to the first and second order coefficients, that is, the strength of direct and delayed density dependence. Populations without predators displayed significantly stronger negative direct density dependence (1+*b*_1_<<1) than did those exposed to predators, where direct density dependence was only moderately negative (1+*b*_1_<1) (Table 2, Fig. 1a). The effects of predation versus no predation had an even stronger effect on the delayed density dependence of the simulated prey populations. In non-predated prey populations, the coefficients of delayed density dependence was distinctly positive (*b*_2_>0), whereas they were negative in predated populations (*b*_2_<0), albeit with different strength depending on the type of predator (Fig. 1a).

**Table 2:** Covariance analyses of the effects of predation and fragmentation on the variables direct (1+*b*_1_) and delayed (*b*_2_) density dependence, and density (ln(*N*)).

Variable	d.f.	SS	MS	F	P
1+*b*_1_					
Predator level	3	5.06	1.69	34.90	<0.0001
Fragmentation level	1	10.55	10.55	218.39	<0.0001
Interaction	3	2.77	0.92	19.10	<0.0001
Residuals	152	7.34	0.05		
					
*b*_2_					
Predator level	3	11.46	3.82	264.22	<0.0001
Fragmentation level	1	13.45	13.45	930.18	<0.0001
Interaction	3	4.59	1.53	105.77	<0.0001
Residuals	152	2.20	0.01		
					
ln(*N*)					
Predator level	3	18.13	6.04	60.76	<0.0001
Fragmentation level	1	0.08	0.08	0.84	0.36
Interaction	3	2.62	0.87	8.79	<0.0001
Residuals	152	15.12	0.10		

The autoregressive structures of the prey populations changed as a response to differences in the predators' numerical responses. The values of *b*_2 _became significantly more negative (Fig. 1a) with increasing specialization of the predators, i.e. when exposed to generalist, intermediate and specialist predators, respectively. By contrast, the coefficients of direct density dependence were approximately equal in all populations, regardless of the degree of specialization in the predator (Table 2, Fig. 1a)

**Figure 2:**
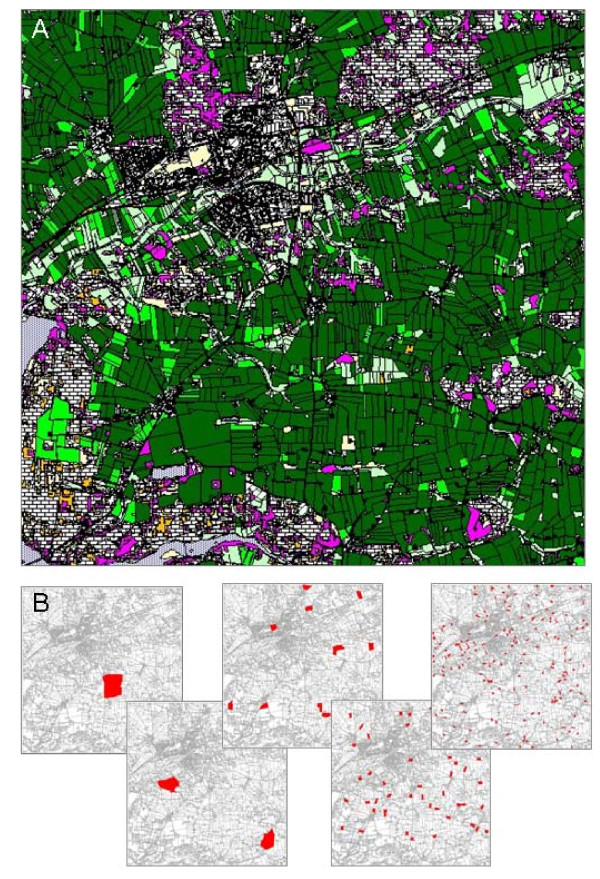
**Landscape characteristics**. A) The 10*10 km basic landscape used in the simulations, representing an existing area in central Jylland, Denmark, centred around 56°22'N, 9°40'E. The polygons represent landscape structures, i.e. forests, fields, watercourses, build-up areas etc. The resolution is 1 m, enabling the inclusion of narrow landscape structure such as road verges, potentially important for vole distribution. B) Superimposed on the basic landscape, is the size and position (in red) of the areas of optimal vole habitat in each of the five levels of landscape fragmentation. The total area of optimal habitat is unchanged (1.5%) between fragmentation levels.

Increasing the degree of fragmentation of optimal habitat available to the prey (Fig. 2), caused a shift in the delayed density dependence, *b*_2_, from being strongly negative to gradually becoming less negative. Although this pattern was clearly seen in all predated populations, the effect was strongest in populations with generalist predators (Fig. 1b). This pattern this corresponded with the predictions of Table 1.

**Figure 3:**
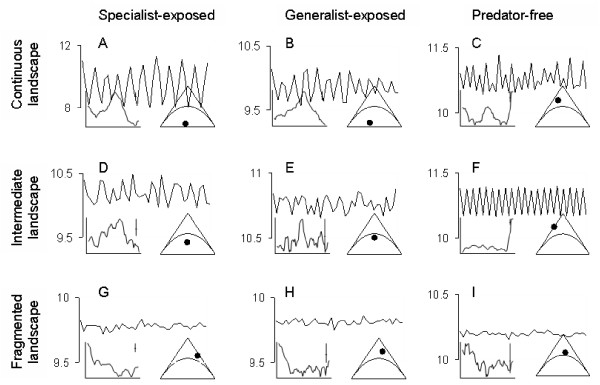
**Time series plots for nine representative examples**. The plots characterize the width of the vole dynamics as a result of different combinations of predator-types and landscape fragmentations. Each graph represents a 40-year fragment of annual vole density (ln-transformed) for one specific time series (note that scales differ). Columns represent three of the predator-types; specialist-exposed (A, D, G), generalist-exposed (B, E, H) and predator-free populations (C, F, I), whereas rows represent three of the fragmentation levels: continuous (A-C), intermediate (D-F) and fragmented (G-I). Superimposed on the time series plots are the (1+*b*_1_)- (*b*_2_)-plane, with the position of the particular time series, and the spectrum plot of the time series.

Clearly, both degree of predator specialization and degree of landscape fragmentation acted in concurrence to alter population dynamics, and the GLM showed highly significant effects of both predators and fragmentation on the coefficients of statistical direct and delayed density dependence (Table 2). Whereas non-predated prey populations displayed stable or biennially fluctuating populations, all predator exposed populations fluctuated to some degree (Fig. 3). A combination of a homogenous landscape and specialized predators lead prey populations to the most regular cycles, displaying pronounced amplitudes, peaking approximately every fourth year. As long as the landscape was kept entirely homogenous, populations with generalist predators also fluctuated, albeit with much less amplitude and somewhat shorter cycle length. However, when the available landscape became increasingly more fragmented, the regularity of the fluctuations broke down and the fluctuations moved into the regions of stable fluctuations. This effect was considerably stronger if the predators were generalists, than if they were specialists (Figs. 1b, 3).

## Discussion

The population dynamics of the simulated prey populations changed considerably with changes in predators and fragmentation, as revealed by the autoregressive analyses. The multi-annual fluctuations displayed by prey populations set in a homogenous landscape with specialist predators (Fig. 3a), closely resemble the changes in density observed in natural populations of microtines under similar conditions, with pronounced amplitude and fluctuations recurring every 3–5 years [16,17]. Similarly, the gradual breakdown in period and amplitude of the prey time series with changes in predation and increased landscape fragmentation (Fig 3b, d–e, g–h), resemble patterns of changing predator compositions and landscape structure observed in natural prey populations, characterized by only annual changes in density [18-20]. The similarity with natural populations is further corroborated by the structure of the simulated time series for populations without predators. These populations do not experience any interspecific interactions, neither through food limitation nor predation, and emergent patterns are the result of intraspecific interactions only. Indeed, natural populations controlled predominantly by intraspecific interactions commonly show biennial fluctuations [6], resembling our results for predator-free populations (Fig. 3).

We found a pronounced difference in direct density dependence (1+*b*_1_) between predated and non-predated populations (Fig. 1a). The differences corresponded closely with the model predictions (Table 1), where (1+*b*_1_) in predated populations was predicted to be smaller, more negative, than in populations with predators. This was likely due to a reduction in the competition among prey, i.e. a reduction in *a*_*ii*_, in predated populations. Indeed, the overall density of prey was lowered significantly when predators were present (Table 2), decreasing the competition for suitable habitat. Such predator-caused reduction in density or competition among prey has been comprehensively documented in natural populations [8,21,22].

The influence of predators was even more obvious and clearly portrayed in the delayed autoregressive component (*b*_2_) of the prey time series. We found a clear segregation between treatments, where predated populations were characterized by a negative *b*_2_, as opposed to the significant, positive *b*_2 _displayed by non-predated prey populations (Fig. 1a). The differences corresponded quite accurately with model predictions where both *a*_*ii *_(intraspecific competition among prey) and *a*_*ji *_(predator effect) affect *b*_2 _(Table 1). Positive delayed effects have been recorded in natural prey populations [23,24] and have, in predator-free populations, been ascribed to inter-annual carry-over effects, where a high population density affects the fecundity of the following generation through competition [25]. Negative delayed effects are usually ascribed to interspecific interactions, such as those between predators and prey, or between herbivores and their forage [5]. Since prey in this study is not food limited, we can concentrate the analyses of the negative delayed density effects to predator-prey interactions. The delayed effect arises because a high prey density facilitates a high predator survival and fecundity, which the adversely affects the density of the prey. Indeed, we find a strong coupling between the densities of prey and predators, in particular when examining the populations exposed to specialist predators where the numerical coupling between predators and prey is particularly strong.

It is well-known that an increased degree of specialization of the predator increases its effect on prey population [21,26], that is, *a*_*ji *_would differ between generalist and specialist predators. Hence, the autoregressive model not only predicts different *b*_2 _between non-predated and predated prey populations, but also that *b*_2 _will vary specifically with the type of predator. This was indeed what our ABM generated prey time series showed: exposed to a generalist, an intermediate and a specialist predator, respectively, values of *b*_2 _from the autoregressive analyses prey time series became significantly more negative (Fig. 1a).

A final evidence for the close linkage between variations in predator-prey interactions and the resulting two-dimensional time series structure of prey population dynamics comes from our independent analyses of the effects of habitat fragmentation on predator efficiency. Concurrent with a gradual increase in the fragmentation of optimal prey habitat, the *b*_2 _of time series from predated prey populations became gradually less negative, which was most pronounced for generalist predators and less for specialist predators (Fig. 1b). That this was related to the effect of fragmentation on the predators influence on prey (i.e. *a*_*ji*_) was corroborated by our time series analyses on non-predated prey populations, where increased habitat fragmentation had no effect on *b*_2 _(Fig. 1b). This apparent effect of habitat fragmentation on predator's influence on prey is further supported by findings from natural predator-prey interactions, where several studies have unequivocally shown that increased fragmentation leads to decreased effect of predators on prey [13,27-29]. In fact, as is clearly shown by our analyses (Fig. 1b), predation by generalist is more affected than specialists [9,27].

## Conclusion

By performing standard time series analyses on prey time series, independently generated from complex, yet controllable agent-based simulations in a natural landscape [30], we have shown that changes in the direct and delayed components of the two-dimensional autoregressive structure of prey time series portray specifically changes in predator-prey interactions. Our results are clearly supported by three lines of evidence: comparisons of time series between predated and non-predated prey populations, between prey populations exposed to different types of predators and between prey populations experiencing different degrees of habitat fragmentation. These results strongly support previous notions [2-5,7] of autoregressive modeling of prey time series as powerful analytical tool for disentangling direct and indirect effects of both predators and environment on long-term dynamics of prey.

## Methods

Time series were generated in the spatially explicit agent-based model, the Animal, Landscape and Man Simulation System (ref. [30]; see also Additional file 1). The model is a C++ based adaptive system incorporating species specific information on ecology, as well as biotic and abiotic environmental factors. The detailed model description is given in html format in Additional file 2. In general, it follows the ODD protocol for describing individual- and agent-based models [31] embracing three core elements, model Overview (O), model Design (D) and model Details (D). The Detail section has been implemented by using the Doxygen program  on the commented source code, creating a html-based documentation. In this way the source can be viewed and navigated through in an efficient and manageable manner. A major advantage is that connections between objects, methods and functions are hyperlinked and can therefore be followed easily together with the source code, even without more than a very basic understanding of programming.

The ABM system described above models the ecology and behavior of the target species at an individual level, and its interactions with conspecifics, predators and environment [30]. For this study, a 10 × 10 km natural landscape (Fig. 2) was used, comprised of patches of optimal vole habitat, interspersed in a matrix, allowing the agent-animals to move through freely, but restricting reproduction and long-term survival to habitat patches. Although voles could not deplete food resources in habitat patches, preventing direct bottom-up predator-prey interactions from food availability, density dependence were incorporated in our model through local scale contest competition for territories by both sexes (see also ODD documentation link above).

Predators, representing specialist, intermediate and generalist predators, respectively, were modeled through different numerical response to changes in prey density. There was no direct link between predators and landscape, thus predators were regulated only by vole density and distribution. The underlying model of the agent-animals (voles) was unchanged in all scenarios, thus the emergent changes in population dynamic patterns originated from changes in predator composition and landscape fragmentation, only, leading to variations in both intra- and interspecific interactions. In addition, both voles and predators were modeled through an individual-based approach, thus emergent population patterns were the result of intra- and inter specific interactions between individuals, not pre-assumed population characteristics.

Landscape fragmentation was obtained by fragmenting the available patches of optimal vole habitat. Using the same basic landscape, fragmentation differed with respect to the number, size and degree of isolation of habitat patches. Total amount of available habitat was unchanged between scenarios, so that both habitat quality and quantity was kept constant between scenarios. An increase in fragmentation level lead to simultaneous changes in three landscape parameters: 1) an approximately exponential increase in the number of patches, 2) a corresponding decrease in patch size, and 3) a decrease in the degree of patch isolation.

Each of four model scenarios: no predators, generalist, intermediate, and specialist predators were separately combined with five degrees of fragmentation, totaling 20 scenarios, each with eight replicates. For each of the resulting 160 replicates, we sampled a 200-year continuous time series of the field vole population density. The generated time series were subsequently analyzed using standard second-order autoregressive analyses [10,32] to determine the coefficients of direct (1+*b*_1_) and delayed, *b*_2_, density dependence. Prior to analyses, time series were ln-transformed, standardized and, if necessary, de-trended.

A generalized linear model (GLM) was used to determine the contribution to the covariance of predation and fragmentation on mean density and the strength of the coefficients of direct and delayed density dependence (i.e., (1+*b*_1_) and *b*_2_). Predation level was treated as a class variable, fragmentation as a continuous variable based on the ln-transformed number of patches. Cycle length was determined by spectral analyses, whereas the amplitude was calculated as the ratio between maximum and minimum density. All analyses were performed using S-plus 6.1 for Windows [33].

## Authors' contributions

MCF conceived the fundamental idea. DKH performed the computer simulations, conducted the statistical analyses and drafted the manuscript. CJT were responsible for design and programming in C++ and wrote the ODD documentation and supplementary material. All authors participated in manuscript revision, and approved the final manuscript.

## Supplementary Material

Additional file 1 **Confronting agent-based models outputs with reality**. The agent-based model (ALMaSS) used by Hendrichsen et al. is summarised together with an example of confronting model outputs with real long-term data. Click here for file

Additional file 2 **Voles and related classes ODDox Documentation**. ODDox documentation of the agent-based model (ALMaSS) applied by Hendrichsen et al. The documentation is started by activating main.html. Click here for file
