# Supplementary material for: Predation and fragmentation portrayed in the statistical structure of prey time series
Source: BMC Ecol. 2009 May 6;9:10. doi: 10.1186/1472-6785-9-10 (PMC2689204; doi:10.1186/1472-6785-9-10)

Confronting agent-based models outputs with reality

An individual agent-based model: The Animal Landscape and Man Simulation System (ALMaSS) (1), originally designed as a predictive tool for answering policy questions regarding the effect of changing landscape structure or management on key species in the Danish landscape, emphasises the animals as affected by landscape and anthropogenic influences. The system is spatially and behaviourally explicit, incorporating dynamic modelling of the biotic and abiotic components of the organism's environment. Whilst many models have been created using agent-based concepts, few if any have attempted so comprehensive a representation of behaviour and environment as is possible in ALMaSS. The combination of detailed environmental simulation with a mechanistic agent-based modelling approach results in a system that is capable of handling at the population level the hitherto largely intractable problems associated with the spatio-temporal interactions between organisms and their environment. The system has been applied to risk assessment (2, 3) as well as to population genetics (4-6), impact assessment (7) and behavioural and landscape ecology (8-10).

In contrast to traditional, conceptually based population models, the ALMaSS concept relies exclusively on the comprehensive dynamic modelling of the individual's behaviour in response to changes in its environment, whereby changes in habitat quality for the organisms modelled vary on a timescale commensurate with the behaviour and ecology of the organism, typically on a daily basis. This model environment is populated with individual animal agents equipped with highly realistic behavioural rules enabling them to extract information from their local surroundings and act upon it via decision making to achieve the goal of reproduction and survival

(1). As in the real world, con-specifics form part of the environment of any individual; hence social behaviour is an important component of the agent's rule base permitting the simulation of processes such as mate selection and territoriality. Importantly, the resulting population dynamics, such as vole cycling, are emergent properties of the organism's daily activity and interactions with its local environment, rather than pre-programmed population-level patterns. Since population-level dynamics are an epiphenomenon of the daily activity of individuals, the model can also be used to predict how the genetic and demographic parameters behave in transitory periods, or with other spatio-temporal factors such as predators (2, 4).

The landscape simulation of ALMaSS utilises a map, typically 10x10km with a $1m^2$ resolution to define the topographical structure of the landscape. The map is defined using 35 landscape element types and almost 70 vegetation types. Vegetation growth models exist for all vegetation types to describe the daily changes in vegetation height and biomass. Weather records are used to provide driving factors for the vegetation and animal models as well as for crop management. Crop and farm management is modelled in terms of events that either affects the state of vegetation or animals directly. The resulting pattern of management events across the landscape is highly realistic and variable. Other miscellaneous landscape events such as cutting of roadside vegetation and traffic loads on roads are also simulated (1). Landscape heterogeneity is therefore controlled spatially by the topography and by cropping choices of the farmer, and temporally by weather, vegetation development, and management.

Confronting agent-based models outputs with reality: Animal modelling is individual-based founded upon a state-machine concept, with states defined as time-variable behavioural or physiological states linked by condition-based transitions. Each animal agent has a set of behavioural rules defining the state-machine together with a set of interface functions that define its interactions with its environment. The resulting models are scaled to the information level available with the aim of representing the current state of ecological knowledge for the species modelled, making the type of ABM applied highly realistic. Indeed, the ALMaSS vole model can re-create the well-known patterns of vole population fluctuations, resulting in highly realistic time-series. As generally predicted (11-15), the form of the dynamics achieved depends on a balance between predator-prey interactions and the spatial dynamics of the vole (Fig. S1). Cyclic dynamics were achieved using a homogenous landscape of suitable habitat and specialist predators, whilst the non-cyclic dynamics were achieved by fragmentation of the habitat and the introduction of generalist predators. Since the vole model was not altered for these simulations, we suggest that this indicates that the model captures the essential dynamics of the system and is suitable for generating the time series utilised in this paper.

1. Topping, C. J., Hansen, T. S., Jensen, T. S., Jepsen, J. U., Nikolajsen, F. & Odderskær, P. (2003) *Ecol. Model*. **167**, 65-82.
2. Topping, C. J. & Odderskær, P. (2004) *Env. Toxicol. and Chem*. **23**, 509-520.
3. Topping, C. J., Sibly, R.M., Akçakaya, H.R., Smith, G.C. & Crocker, D.R. (2005) *Ecotoxicology* **14**, 925-936.
4. Pertoldi, C. & Topping, C. J. (2004) *J. Nat. Cons*. **12**, 111-120.
5. Pertoldi, C. & Topping, C. J. (2004) *Crit. Rev. Ecotox*. **34**, 487-498.

6. Topping, C. J., Østergaard, S., Pertoldi, C. B. & Bach, L. A. (2003) *Ann. Zool. Fen*. **40**, 255-267.
7. Jepsen, J. U., Topping, C. J., Odderskær, P. & Andersen, P. N. (2005) *Agric. Ecosyst. Environ*. **105**, 581-594.
8. Bilde, T. & Topping, C. J. (2004) *EcoScience* 11, 64-73.
9. Jepsen, J. U. & Topping, C. J. (2004) *Can. J. Zool*. **82**, 1528-1541.
10. Thorbek, P. & Topping, C. J. (2005) *BioControl* **50**, 1-33.
11. Erlinge, S., Göranson, G., Hansson, L., Högsted, G., Liberg, O., Nilsson, I. N., Nilsson, T., Von Schantz, T. & Sylven, M. (1983) *Oikos* 40, 36-52.
12. Hentonnen, H., Oksanen, T., Jortikka, A. & Haukisalmi, V. (1987) *Oikos* **50**, 353-365.
13. Hanski, I. (1987) *Trends Ecol. Evol*. **2**, 55-56.
14. Hanski, I., Hansson, L. & Henttonen, H. (1991) *J. Anim. Ecol*. **60**, 353-367.
15. Hansson, L. & Henttonen, H. (1988) *Trends Ecol. Evol*. **3**, 195-200.

Fig. S1. Comparisons between model generated and real *Microtus agrestis* biennial population fluctuations. A) non-cyclic dynamics from southern Sweden (10). B) cyclic dynamics from northern Finland (11). C) simulated dynamics using a fragmented landscape and mixed predator assemblage. D) simulated cyclic dynamics using a homogenous landscape and specialist predators. A and B are taken from (15).

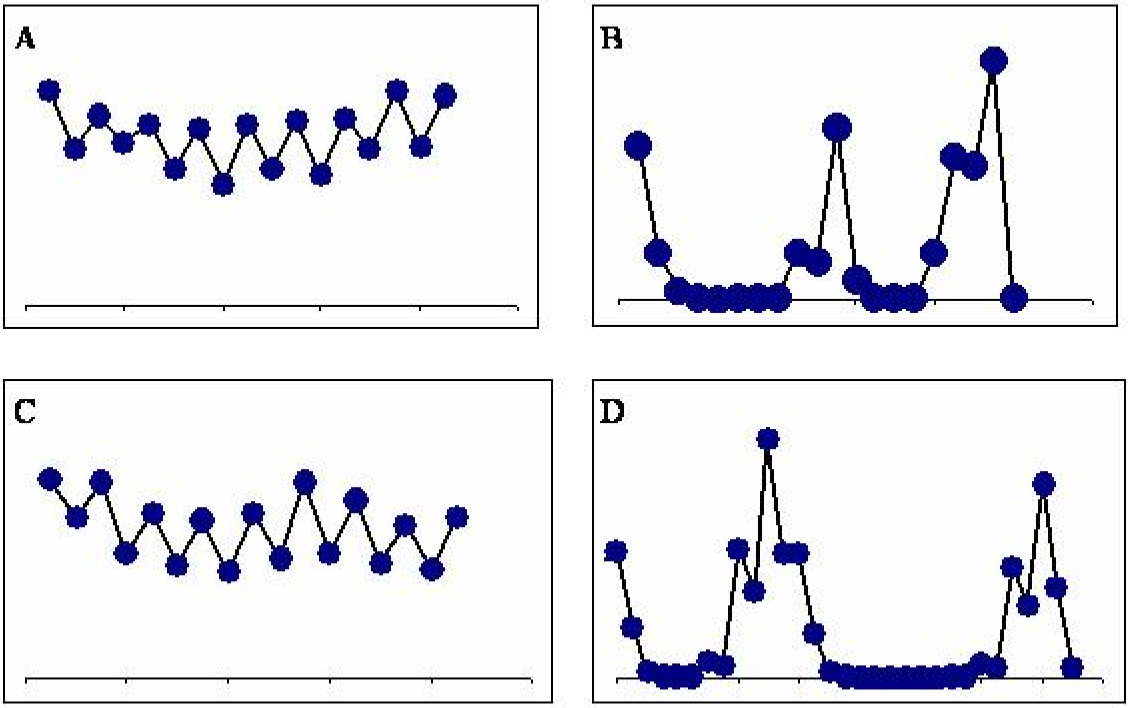

Supplement: Additional file 1 — Confronting agent-based models outputs with reality. The agent-based model (ALMaSS) used by Hendrichsen et al. is summarised together with an example of confronting model outputs with real long-term data. [file 1472-6785-9-10-S1.pdf]
