# Supplementary material for: Predation and fragmentation portrayed in the statistical structure of prey time series
Source: BMC Ecol. 2009 May 6;9:10. doi: 10.1186/1472-6785-9-10 (PMC2689204; doi:10.1186/1472-6785-9-10)
Supplement: Additional file 2 — Voles and related classes ODDox Documentation. ODDox documentation of the agent-based model (ALMaSS) applied by Hendrichsen et al. The documentation is started by activating main.html. [file 1472-6785-9-10-S2.zip › Vole_ODDox/annotated.html]

ALMaSS ODDox: Class List

- Main Page
- Related Pages
- Classes
- Files

- Alphabetical List
- Class List
- Class Hierarchy
- Class Members

# Class List

Here are the classes, structs, unions and interfaces with brief descriptions:

|  |  |
| --- | --- |
| AgroChemIndustryCerealFarm1 | Inbuilt special purpose farm type |
| AgroChemIndustryCerealFarm2 | Inbuilt special purpose farm type |
| AgroChemIndustryCerealFarm3 | Inbuilt special purpose farm type |
| AlleleFreq | Class to handle statistics and constructs based on allele frequencies |
| AnimalPosition | A class defining an animals position |
| CompareState | Function to compare to TAnimal's Current behavioural state |
| CompareX | Function to compare to TAnimal's m\_Location\_x |
| CompareY | Function to compare to TAnimal's m\_Location\_y |
| ConventionalCattle | Inbuilt farm type |
| ConventionalPig | Inbuilt farm type |
| ConventionalPlant | Inbuilt farm type |
| ConvMarginalJord | Inbuilt special purpose farm type |
| Crop | The base class for all crops |
| CropRotation |  |
| Farm |  |
| FarmEvent | A struct to hold the information required to trigger a farm event |
| GeneticMaterial | Class for the genetic material optionally carried by animals in ALMaSS |
| GeneticMaterial1616 |  |
| IntArray100 | A struct of 100 ints |
| NoPesticideBaseFarm | Inbuilt special purpose farm type |
| NoPesticideNoPFarm | Inbuilt special purpose farm type |
| OrganicCattle | A farm that can have its rotation defined by the user at runtime |
| OrganicPig | A farm that can have its rotation defined by the user at runtime |
| OrganicPlant | A farm that can have its rotation defined by the user at runtime |
| Owl | One of two current implementations of TPredator |
| PesticideTrialControl | Inbuilt special purpose farm type |
| PesticideTrialToxicControl | Inbuilt special purpose farm type |
| PesticideTrialTreatment | Inbuilt special purpose farm type |
| Population\_Manager | Base class for all population managers |
| probe\_data | Data structure to hold & output probe data probe data is designed to be used to return the number of objects in a given area or areas in specific element or vegetation types or farms |
| rectangle | A struct defining two x,y coordinate sets |
| RoeDeerInfo | Part of the basic ALMaSS system (obselete) |
| Rotation |  |
| SetAside | Rotational set-aside management class |
| Starter |  |
| struct\_Predator | Used for creation of a new predator object |
| struct\_Vole\_Adult | A struct for passing data to create a new vole |
| TALMaSSObject | The base class of all ALMaSS objects requiring Step code |
| TAnimal | The base class for all ALMaSS animal classes |
| TPredator | The base class for predators encompsassing all their general behaviours |
| TPredator\_Population\_Manager | The class to handle all predator population related matters |
| UserDefinedFarm1 | A farm that can have its rotation defined by the user at runtime |
| UserDefinedFarm10 | A farm that can have its rotation defined by the user at runtime |
| UserDefinedFarm11 | A farm that can have its rotation defined by the user at runtime |
| UserDefinedFarm12 | A farm that can have its rotation defined by the user at runtime |
| UserDefinedFarm13 | A farm that can have its rotation defined by the user at runtime |
| UserDefinedFarm14 | A farm that can have its rotation defined by the user at runtime |
| UserDefinedFarm15 | A farm that can have its rotation defined by the user at runtime |
| UserDefinedFarm16 | A farm that can have its rotation defined by the user at runtime |
| UserDefinedFarm2 | A farm that can have its rotation defined by the user at runtime |
| UserDefinedFarm3 | A farm that can have its rotation defined by the user at runtime |
| UserDefinedFarm4 | A farm that can have its rotation defined by the user at runtime |
| UserDefinedFarm5 | A farm that can have its rotation defined by the user at runtime |
| UserDefinedFarm6 | A farm that can have its rotation defined by the user at runtime |
| UserDefinedFarm7 | A farm that can have its rotation defined by the user at runtime |
| UserDefinedFarm8 | A farm that can have its rotation defined by the user at runtime |
| UserDefinedFarm9 | A farm that can have its rotation defined by the user at runtime |
| Vole\_Base | Base class for voles - all vole objects are descended from this class |
| Vole\_Female | The class for female voles |
| Vole\_Male | The class for male voles |
| Vole\_Population\_Manager | The class to handle all vole population related matters |
| Weasel | One of two current implementations of TPredator |
| WinterWheat | WinterWheat class |

---

Generated on Thu Jan 22 14:13:45 2009 for ALMaSS ODDox by 
 1.5.6 
