# Supplementary material for: Predation and fragmentation portrayed in the statistical structure of prey time series
Source: BMC Ecol. 2009 May 6;9:10. doi: 10.1186/1472-6785-9-10 (PMC2689204; doi:10.1186/1472-6785-9-10)
Supplement: Additional file 2 — Voles and related classes ODDox Documentation. ODDox documentation of the agent-based model (ALMaSS) applied by Hendrichsen et al. The documentation is started by activating main.html. [file 1472-6785-9-10-S2.zip › Vole_ODDox/classes.html]

ALMaSS ODDox: Alphabetical List

- Main Page
- Related Pages
- Classes
- Files

- Alphabetical List
- Class List
- Class Hierarchy
- Class Members

# Class Index

A | C | F | G | I | N | O | P | R | S | T | U | V | W

|  |  |  |  |  |  |  |  |  |
| --- | --- | --- | --- | --- | --- | --- | --- | --- |
| |  | | --- | | A | | |  | | --- | | F | | |  | | --- | | P | | |  | | --- | | T | | UserDefinedFarm3 |
| AgroChemIndustryCerealFarm1 | Farm | PesticideTrialControl | TALMaSSObject | UserDefinedFarm4 |
| AgroChemIndustryCerealFarm2 | FarmEvent | PesticideTrialToxicControl | TAnimal | UserDefinedFarm5 |
| AgroChemIndustryCerealFarm3 | |  | | --- | | G | | PesticideTrialTreatment | TPredator | UserDefinedFarm6 |
| AlleleFreq | GeneticMaterial | Population\_Manager | TPredator\_Population\_Manager | UserDefinedFarm7 |
| AnimalPosition | GeneticMaterial1616 | probe\_data | |  | | --- | | U | | UserDefinedFarm8 |
| |  | | --- | | C | | |  | | --- | | I | | |  | | --- | | R | | UserDefinedFarm1 | UserDefinedFarm9 |
| CompareState | IntArray100 | rectangle | UserDefinedFarm10 | |  | | --- | | V | |
| CompareX | |  | | --- | | N | | RoeDeerInfo | UserDefinedFarm11 | Vole\_Base |
| CompareY | NoPesticideBaseFarm | Rotation | UserDefinedFarm12 | Vole\_Female |
| ConventionalCattle | NoPesticideNoPFarm | |  | | --- | | S | | UserDefinedFarm13 | Vole\_Male |
| ConventionalPig | |  | | --- | | O | | SetAside | UserDefinedFarm14 | Vole\_Population\_Manager |
| ConventionalPlant | OrganicCattle | Starter | UserDefinedFarm15 | |  | | --- | | W | |
| ConvMarginalJord | OrganicPig | struct\_Predator | UserDefinedFarm16 | Weasel |
| Crop | OrganicPlant | struct\_Vole\_Adult | UserDefinedFarm2 | WinterWheat |
| CropRotation | Owl |

A | C | F | G | I | N | O | P | R | S | T | U | V | W

---

Generated on Thu Jan 22 14:13:45 2009 for ALMaSS ODDox by 
 1.5.6 
