# Supplementary material for: Predation and fragmentation portrayed in the statistical structure of prey time series
Source: BMC Ecol. 2009 May 6;9:10. doi: 10.1186/1472-6785-9-10 (PMC2689204; doi:10.1186/1472-6785-9-10)
Supplement: Additional file 2 — Voles and related classes ODDox Documentation. ODDox documentation of the agent-based model (ALMaSS) applied by Hendrichsen et al. The documentation is started by activating main.html. [file 1472-6785-9-10-S2.zip › Vole_ODDox/classprobe__data-members.html]

ALMaSS ODDox: Member List

- Main Page
- Related Pages
- Classes
- Files

- Alphabetical List
- Class List
- Class Hierarchy
- Class Members

# probe\_data Member List

This is the complete list of members for probe\_data, including all inherited members.

|  |  |  |
| --- | --- | --- |
| AppendToFile() | probe\_data | `[inline]` |
| CloseFile() | probe\_data |  |
| FileAppendOutput(int No, int time) | probe\_data |  |
| FileOutput(int No, int time, int ProbeNo) | probe\_data |  |
| FileRecord | probe\_data |  |
| m\_NoAreas | probe\_data |  |
| m\_NoEleTypes | probe\_data |  |
| m\_NoFarms | probe\_data |  |
| m\_NoVegTypes | probe\_data |  |
| m\_Rect | probe\_data |  |
| m\_RefEle | probe\_data |  |
| m\_RefFarms | probe\_data |  |
| m\_RefVeg | probe\_data |  |
| m\_ReportInterval | probe\_data |  |
| m\_TargetTypes | probe\_data |  |
| MyFile | probe\_data | `[protected]` |
| MyFileName | probe\_data | `[protected]` |
| OpenFile(char \*Nme) | probe\_data |  |
| probe\_data() | probe\_data |  |
| SetFile(FILE \*F) | probe\_data |  |
| Time | probe\_data | `[protected]` |
| ~probe\_data() | probe\_data |  |

---

Generated on Thu Jan 22 14:13:46 2009 for ALMaSS ODDox by 
 1.5.6 
