# Supplementary material for: Predation and fragmentation portrayed in the statistical structure of prey time series
Source: BMC Ecol. 2009 May 6;9:10. doi: 10.1186/1472-6785-9-10 (PMC2689204; doi:10.1186/1472-6785-9-10)
Supplement: Additional file 2 — Voles and related classes ODDox Documentation. ODDox documentation of the agent-based model (ALMaSS) applied by Hendrichsen et al. The documentation is started by activating main.html. [file 1472-6785-9-10-S2.zip › Vole_ODDox/classprobe__data.html]

ALMaSS ODDox: probe\_data Class Reference

- Main Page
- Related Pages
- Classes
- Files

- Alphabetical List
- Class List
- Class Hierarchy
- Class Members

# probe\_data Class Reference

`#include <PopulationManager.h>`

List of all members.

---

## Detailed Description

Data structure to hold & output probe data probe data is designed to be used to return the number of objects in a given area or areas in specific element or vegetation types or farms.

|  |
| --- |
|  |
| Public Member Functions | |
| bool | AppendToFile () |
| void | CloseFile () |
| void | FileAppendOutput (int No, int time) |
| void | FileOutput (int No, int time, int ProbeNo) |
| FILE \* | OpenFile (char \*Nme) |
|  | probe\_data () |
| void | SetFile (FILE \*F) |
|  | ~probe\_data () |
| Public Attributes | |
| bool | FileRecord |
| unsigned | m\_NoAreas |
| unsigned | m\_NoEleTypes |
| unsigned | m\_NoFarms |
| unsigned | m\_NoVegTypes |
| rectangle | m\_Rect [10] |
| TTypesOfLandscapeElement | m\_RefEle [25] |
| unsigned | m\_RefFarms [25] |
| TTypesOfVegetation | m\_RefVeg [25] |
| unsigned | m\_ReportInterval |
| bool | m\_TargetTypes [10] |
| Protected Attributes | |
| FILE \* | MyFile |
| char | MyFileName [255] |
| int | Time |

---

## Constructor & Destructor Documentation

|  |  |  |  |  |
| --- | --- | --- | --- | --- |
| probe\_data::probe\_data | ( |  | ) |  |

Constructor for probe\_data

References FileRecord, and Time.

```
01354                        {
01355   Time = 0;
01356   FileRecord = false;
01357 }
```

|  |  |  |  |  |
| --- | --- | --- | --- | --- |
| probe\_data::~probe\_data | ( |  | ) |  |

Destructor for probe\_data

```
01388                         {
01389 }
```

---

## Member Function Documentation

|  |  |  |  |  |
| --- | --- | --- | --- | --- |
| bool probe\_data::AppendToFile | ( |  | ) | `[inline]` |

References MyFile, and MyFileName.

```
00212                       {
00213         MyFile=fopen(MyFileName, "a");
00214     if (!MyFile) {
00215                 g_msg->Warn( WARN_FILE, "PopulationManager::AppendToFile() Unable to open file for reading: ", MyFileName );
00216                 exit(1);
00217     }
00218     return true;
00219   }
```

|  |  |  |  |  |
| --- | --- | --- | --- | --- |
| void probe\_data::CloseFile | ( |  | ) |  |

References MyFile.

```
00098                            {
00099   if ( MyFile != NULL ) {
00100     fclose( MyFile );
00101   }
00102 };
```

|  |  |  |  |
| --- | --- | --- | --- |
| void probe\_data::FileAppendOutput | ( | int | *No*, |
|  |  | int | *time* |  |
|  | ) |  |  |  |

References FileRecord, MyFile, and MyFileName.

```
01337                                                     {
01338   MyFile = fopen(MyFileName, "a" );
01339   if ( !MyFile ) {
01340     g_msg->Warn( (MapErrorState)0,"Cannot open file for append: ", MyFileName );
01341     exit( 0 );
01342   }
01343   if ( FileRecord ) {
01344     fprintf( MyFile, "%d %d\n", time, No );
01345   }
01346   fclose( MyFile );
01347 }
```

|  |  |  |  |
| --- | --- | --- | --- |
| void probe\_data::FileOutput | ( | int | *No*, |
|  |  | int | *time*, |
|  |  | int | *ProbeNo* |  |
|  | ) |  |  |  |

Basic output function of the default probe data file.   
This just counts numbers in specified areas

References FileRecord, and MyFile.

Referenced by Population\_Manager::ProbeReport().

```
01322                                                            {
01323   if ( FileRecord ) {
01324 
01325     if ( ProbeNo == 0 ) {
01326       // First probe so write the time and a new line
01327       fprintf( MyFile, "\n" );
01328       fprintf( MyFile, "%d\t%d", time, No );
01329     } else
01330       fprintf( MyFile, "\t%d", No );
01331   }
01332   fflush( MyFile );
01333 }
```

|  |  |  |  |  |  |
| --- | --- | --- | --- | --- | --- |
| FILE \* probe\_data::OpenFile | ( | char \* | *Nme* | ) |  |

Opens the default probe data output file

References MyFile, and MyFileName.

```
01364                                         {
01365   MyFile = fopen( Nme, "w" );
01366   if ( !MyFile ) {
01367     g_msg->Warn( (MapErrorState)0,"probe_data::OpenFile - Cannot open file for append: ", Nme );
01368     exit( 0 );
01369   }
01370   strcpy( MyFileName, Nme );
01371   return MyFile;
01372 }
```

|  |  |  |  |  |  |
| --- | --- | --- | --- | --- | --- |
| void probe\_data::SetFile | ( | FILE \* | *F* | ) |  |

Sets the filename for the default probe data output

References MyFile.

```
01379                                    {
01380   MyFile = F;
01381 }
```

---

## Member Data Documentation

|  |
| --- |
| bool probe\_data::FileRecord |

Referenced by FileAppendOutput(), FileOutput(), probe\_data(), and Population\_Manager::ProbeFileInput().

|  |
| --- |
| unsigned probe\_data::m\_NoAreas |

Referenced by Population\_Manager::Probe(), and Population\_Manager::ProbeFileInput().

|  |
| --- |
| unsigned probe\_data::m\_NoEleTypes |

Referenced by Population\_Manager::Probe(), and Population\_Manager::ProbeFileInput().

|  |
| --- |
| unsigned probe\_data::m\_NoFarms |

Referenced by Population\_Manager::Probe(), and Population\_Manager::ProbeFileInput().

|  |
| --- |
| unsigned probe\_data::m\_NoVegTypes |

Referenced by Population\_Manager::Probe(), and Population\_Manager::ProbeFileInput().

|  |
| --- |
| rectangle probe\_data::m\_Rect[10] |

Referenced by Population\_Manager::Probe(), and Population\_Manager::ProbeFileInput().

|  |
| --- |
| TTypesOfLandscapeElement probe\_data::m\_RefEle[25] |

Referenced by Population\_Manager::Probe(), and Population\_Manager::ProbeFileInput().

|  |
| --- |
| unsigned probe\_data::m\_RefFarms[25] |

Referenced by Population\_Manager::Probe(), and Population\_Manager::ProbeFileInput().

|  |
| --- |
| TTypesOfVegetation probe\_data::m\_RefVeg[25] |

Referenced by Population\_Manager::Probe(), and Population\_Manager::ProbeFileInput().

|  |
| --- |
| unsigned probe\_data::m\_ReportInterval |

Referenced by Population\_Manager::ProbeFileInput().

|  |
| --- |
| bool probe\_data::m\_TargetTypes[10] |

Referenced by Population\_Manager::ProbeFileInput().

|  |
| --- |
| FILE\* probe\_data::MyFile `[protected]` |

Referenced by AppendToFile(), CloseFile(), FileAppendOutput(), FileOutput(), OpenFile(), and SetFile().

|  |
| --- |
| char probe\_data::MyFileName[255] `[protected]` |

Referenced by AppendToFile(), FileAppendOutput(), and OpenFile().

|  |
| --- |
| int probe\_data::Time `[protected]` |

Referenced by probe\_data().

---

The documentation for this class was generated from the following files:

- PopulationManager.h- PopulationManager.cpp

---

Generated on Thu Jan 22 14:13:46 2009 for ALMaSS ODDox by 
 1.5.6 
