# Supplementary material for: Predation and fragmentation portrayed in the statistical structure of prey time series
Source: BMC Ecol. 2009 May 6;9:10. doi: 10.1186/1472-6785-9-10 (PMC2689204; doi:10.1186/1472-6785-9-10)
Supplement: Additional file 2 — Voles and related classes ODDox Documentation. ODDox documentation of the agent-based model (ALMaSS) applied by Hendrichsen et al. The documentation is started by activating main.html. [file 1472-6785-9-10-S2.zip › Vole_ODDox/classstruct___predator.html]

ALMaSS ODDox: struct\_Predator Class Reference

- Main Page
- Related Pages
- Classes
- Files

- Alphabetical List
- Class List
- Class Hierarchy
- Class Members

# struct\_Predator Class Reference

`#include <Predators.H>`

List of all members.

---

## Detailed Description

Used for creation of a new predator object.

|  |
| --- |
|  |
| Public Attributes | |
| Landscape \* | L |
| TPredator\_Population\_Manager \* | PM |
| int | x |
| int | y |

---

## Member Data Documentation

|  |
| --- |
| Landscape\* struct\_Predator::L |

Referenced by Owl::BeginStep(), Weasel::BeginStep(), TPredator\_Population\_Manager::CreateObjects(), and TPredator\_Population\_Manager::TPredator\_Population\_Manager().

|  |
| --- |
| TPredator\_Population\_Manager\* struct\_Predator::PM |

Referenced by Owl::BeginStep(), Weasel::BeginStep(), TPredator\_Population\_Manager::CreateObjects(), and TPredator\_Population\_Manager::TPredator\_Population\_Manager().

|  |
| --- |
| int struct\_Predator::x |

Referenced by Owl::BeginStep(), Weasel::BeginStep(), TPredator\_Population\_Manager::CreateObjects(), and TPredator\_Population\_Manager::TPredator\_Population\_Manager().

|  |
| --- |
| int struct\_Predator::y |

Referenced by Owl::BeginStep(), Weasel::BeginStep(), TPredator\_Population\_Manager::CreateObjects(), and TPredator\_Population\_Manager::TPredator\_Population\_Manager().

---

The documentation for this class was generated from the following file:

- Predators.H

---

Generated on Thu Jan 22 14:13:46 2009 for ALMaSS ODDox by 
 1.5.6 
