# Supplementary material for: Predation and fragmentation portrayed in the statistical structure of prey time series
Source: BMC Ecol. 2009 May 6;9:10. doi: 10.1186/1472-6785-9-10 (PMC2689204; doi:10.1186/1472-6785-9-10)
Supplement: Additional file 2 — Voles and related classes ODDox Documentation. ODDox documentation of the agent-based model (ALMaSS) applied by Hendrichsen et al. The documentation is started by activating main.html. [file 1472-6785-9-10-S2.zip › Vole_ODDox/class_agro_chem_industry_cereal_farm1.html]

ALMaSS ODDox: AgroChemIndustryCerealFarm1 Class Reference

- Main Page
- Related Pages
- Classes
- Files

- Alphabetical List
- Class List
- Class Hierarchy
- Class Members

# AgroChemIndustryCerealFarm1 Class Reference

`#include <farm.h>`

Inheritance diagram for AgroChemIndustryCerealFarm1:

List of all members.

---

## Detailed Description

Inbuilt special purpose farm type.

|  |
| --- |
|  |
| Public Member Functions | |
|  | AgroChemIndustryCerealFarm1 (void) |

---

## Constructor & Destructor Documentation

|  |  |  |  |  |  |
| --- | --- | --- | --- | --- | --- |
| AgroChemIndustryCerealFarm1::AgroChemIndustryCerealFarm1 | ( | void |  | ) |  |

References Farm::m\_farmtype, Farm::m\_rotation, Farm::m\_stockfarmer, tof\_AgroChemIndustryCerealFarm1, tov\_AgroChemIndustryCereal, tov\_CloverGrassGrazed1, tov\_CloverGrassGrazed2, and tov\_FodderBeet.

```
01190                                                                : Farm() // 10
01191 {
01192   m_farmtype = tof_AgroChemIndustryCerealFarm1;
01193   m_stockfarmer = true;
01194   // Adjust as needed.
01195   m_rotation.resize( 9 );
01196   m_rotation[ 0 ] = tov_AgroChemIndustryCereal;
01197   m_rotation[ 1 ] = tov_CloverGrassGrazed1;
01198   m_rotation[ 2 ] = tov_CloverGrassGrazed2;
01199   m_rotation[ 3 ] = tov_AgroChemIndustryCereal;
01200   m_rotation[ 4 ] = tov_AgroChemIndustryCereal;
01201   m_rotation[ 5 ] = tov_AgroChemIndustryCereal;
01202   m_rotation[ 6 ] = tov_FodderBeet;
01203   m_rotation[ 7 ] = tov_CloverGrassGrazed1;
01204   m_rotation[ 8 ] = tov_AgroChemIndustryCereal;
01205 }
```

---

The documentation for this class was generated from the following files:

- farm.h- farm.cpp

---

Generated on Thu Jan 22 14:13:45 2009 for ALMaSS ODDox by 
 1.5.6 
