# Supplementary material for: Predation and fragmentation portrayed in the statistical structure of prey time series
Source: BMC Ecol. 2009 May 6;9:10. doi: 10.1186/1472-6785-9-10 (PMC2689204; doi:10.1186/1472-6785-9-10)
Supplement: Additional file 2 — Voles and related classes ODDox Documentation. ODDox documentation of the agent-based model (ALMaSS) applied by Hendrichsen et al. The documentation is started by activating main.html. [file 1472-6785-9-10-S2.zip › Vole_ODDox/class_agro_chem_industry_cereal_farm2.html]

ALMaSS ODDox: AgroChemIndustryCerealFarm2 Class Reference

- Main Page
- Related Pages
- Classes
- Files

- Alphabetical List
- Class List
- Class Hierarchy
- Class Members

# AgroChemIndustryCerealFarm2 Class Reference

`#include <farm.h>`

Inheritance diagram for AgroChemIndustryCerealFarm2:

List of all members.

---

## Detailed Description

Inbuilt special purpose farm type.

|  |
| --- |
|  |
| Public Member Functions | |
|  | AgroChemIndustryCerealFarm2 (void) |

---

## Constructor & Destructor Documentation

|  |  |  |  |  |  |
| --- | --- | --- | --- | --- | --- |
| AgroChemIndustryCerealFarm2::AgroChemIndustryCerealFarm2 | ( | void |  | ) |  |

References Farm::m\_farmtype, Farm::m\_rotation, Farm::m\_stockfarmer, tof\_AgroChemIndustryCerealFarm2, tov\_AgroChemIndustryCereal, and tov\_WinterRape.

```
01207                                                                : Farm() // 11
01208 {
01209   m_farmtype = tof_AgroChemIndustryCerealFarm2;
01210   m_stockfarmer = true;
01211 
01212   // Adjust as needed.
01213   m_rotation.resize( 3 );
01214   m_rotation[ 0 ] = tov_WinterRape;
01215   m_rotation[ 1 ] = tov_AgroChemIndustryCereal;
01216   m_rotation[ 2 ] = tov_AgroChemIndustryCereal;
01217   /*  m_rotation[ 3] = tov_AgroChemIndustryCereal; m_rotation[ 4] = tov_Setaside; m_rotation[ 5] = tov_FieldPeas;
01218   m_rotation[ 6] = tov_AgroChemIndustryCereal; m_rotation[ 7] = tov_WinterRye; m_rotation[ 8] = tov_AgroChemIndustryCereal; */
01219 }
```

---

The documentation for this class was generated from the following files:

- farm.h- farm.cpp

---

Generated on Thu Jan 22 14:13:45 2009 for ALMaSS ODDox by 
 1.5.6 
