# Supplementary material for: Predation and fragmentation portrayed in the statistical structure of prey time series
Source: BMC Ecol. 2009 May 6;9:10. doi: 10.1186/1472-6785-9-10 (PMC2689204; doi:10.1186/1472-6785-9-10)
Supplement: Additional file 2 — Voles and related classes ODDox Documentation. ODDox documentation of the agent-based model (ALMaSS) applied by Hendrichsen et al. The documentation is started by activating main.html. [file 1472-6785-9-10-S2.zip › Vole_ODDox/class_agro_chem_industry_cereal_farm3.html]

ALMaSS ODDox: AgroChemIndustryCerealFarm3 Class Reference

- Main Page
- Related Pages
- Classes
- Files

- Alphabetical List
- Class List
- Class Hierarchy
- Class Members

# AgroChemIndustryCerealFarm3 Class Reference

`#include <farm.h>`

Inheritance diagram for AgroChemIndustryCerealFarm3:

List of all members.

---

## Detailed Description

Inbuilt special purpose farm type.

|  |
| --- |
|  |
| Public Member Functions | |
|  | AgroChemIndustryCerealFarm3 (void) |

---

## Constructor & Destructor Documentation

|  |  |  |  |  |  |
| --- | --- | --- | --- | --- | --- |
| AgroChemIndustryCerealFarm3::AgroChemIndustryCerealFarm3 | ( | void |  | ) |  |

References Farm::m\_farmtype, Farm::m\_rotation, Farm::m\_stockfarmer, tof\_AgroChemIndustryCerealFarm3, tov\_AgroChemIndustryCereal, tov\_FieldPeas, tov\_Setaside, tov\_WinterRape, and tov\_WinterRye.

```
01223                                                                : Farm() // 12
01224 {
01225   m_farmtype = tof_AgroChemIndustryCerealFarm3;
01226   m_stockfarmer = false;
01227 
01228   // Adjust as needed.
01229   m_rotation.resize( 9 );
01230   m_rotation[ 0 ] = tov_Setaside;
01231   m_rotation[ 1 ] = tov_FieldPeas;
01232   m_rotation[ 2 ] = tov_AgroChemIndustryCereal;
01233   m_rotation[ 3 ] = tov_WinterRye;
01234   m_rotation[ 4 ] = tov_AgroChemIndustryCereal;
01235   m_rotation[ 5 ] = tov_WinterRape;
01236   m_rotation[ 6 ] = tov_AgroChemIndustryCereal;
01237   m_rotation[ 7 ] = tov_AgroChemIndustryCereal;
01238   m_rotation[ 8 ] = tov_AgroChemIndustryCereal;
01239 }
```

---

The documentation for this class was generated from the following files:

- farm.h- farm.cpp

---

Generated on Thu Jan 22 14:13:45 2009 for ALMaSS ODDox by 
 1.5.6 
