# Supplementary material for: Predation and fragmentation portrayed in the statistical structure of prey time series
Source: BMC Ecol. 2009 May 6;9:10. doi: 10.1186/1472-6785-9-10 (PMC2689204; doi:10.1186/1472-6785-9-10)
Supplement: Additional file 2 — Voles and related classes ODDox Documentation. ODDox documentation of the agent-based model (ALMaSS) applied by Hendrichsen et al. The documentation is started by activating main.html. [file 1472-6785-9-10-S2.zip › Vole_ODDox/class_allele_freq.html]

ALMaSS ODDox: AlleleFreq Class Reference

- Main Page
- Related Pages
- Classes
- Files

- Alphabetical List
- Class List
- Class Hierarchy
- Class Members

# AlleleFreq Class Reference

`#include <GeneticMaterial.H>`

List of all members.

---

## Detailed Description

Class to handle statistics and constructs based on allele frequencies.

|  |
| --- |
|  |
| Public Member Functions | |
|  | AlleleFreq () |
| int | SupplyAN (int loc, int al) |
| Protected Attributes | |
| float | AlleleFrequency [32][16] |
| int | AlleleNumber [32][16] |
| float | HE [32] |
| float | HO [32] |
| int | NoAlleles [32] |

---

## Constructor & Destructor Documentation

|  |  |  |  |  |
| --- | --- | --- | --- | --- |
| AlleleFreq::AlleleFreq | ( |  | ) |  |

References AlleleNumber.

```
00030                         {
00031   FILE * FreqFile = fopen("GeneticFrequencies.txt", "r" );
00032   int data;
00033   if ( !FreqFile ) {
00034     g_msg->Warn( "GeneticFrequencies File missing", NULL );
00035     exit( 0 );
00036   }
00037   for ( int i = 0; i < 16; i++ ) {
00038     for ( int j = 0; j < 4; j++ ) {
00039       fscanf( FreqFile, "%d", & data );
00040       AlleleNumber[ i ] [ j ] = data;
00041     }
00042   }
00043   for ( int i = 16; i < 32; i++ ) {
00044     for ( int j = 0; j < 16; j++ ) {
00045       fscanf( FreqFile, "%d", & data );
00046       AlleleNumber[ i ] [ j ] = data;
00047     }
00048   }
00049   fclose( FreqFile );
00050 }
```

---

## Member Function Documentation

|  |  |  |  |
| --- | --- | --- | --- |
| int AlleleFreq::SupplyAN | ( | int | *loc*, |
|  |  | int | *al* |  |
|  | ) |  |  | `[inline]` |

References AlleleNumber.

Referenced by GeneticMaterial::Initiation().

```
00041 { return AlleleNumber[loc][al];}
```

---

## Member Data Documentation

|  |
| --- |
| float AlleleFreq::AlleleFrequency[32][16] `[protected]` |

|  |
| --- |
| int AlleleFreq::AlleleNumber[32][16] `[protected]` |

Referenced by AlleleFreq(), and SupplyAN().

|  |
| --- |
| float AlleleFreq::HE[32] `[protected]` |

|  |
| --- |
| float AlleleFreq::HO[32] `[protected]` |

|  |
| --- |
| int AlleleFreq::NoAlleles[32] `[protected]` |

---

The documentation for this class was generated from the following files:

- GeneticMaterial.H- GeneticMaterial.cpp

---

Generated on Thu Jan 22 14:13:45 2009 for ALMaSS ODDox by 
 1.5.6 
