# Supplementary material for: Predation and fragmentation portrayed in the statistical structure of prey time series
Source: BMC Ecol. 2009 May 6;9:10. doi: 10.1186/1472-6785-9-10 (PMC2689204; doi:10.1186/1472-6785-9-10)
Supplement: Additional file 2 — Voles and related classes ODDox Documentation. ODDox documentation of the agent-based model (ALMaSS) applied by Hendrichsen et al. The documentation is started by activating main.html. [file 1472-6785-9-10-S2.zip › Vole_ODDox/class_animal_position.html]

ALMaSS ODDox: AnimalPosition Class Reference

- Main Page
- Related Pages
- Classes
- Files

- Alphabetical List
- Class List
- Class Hierarchy
- Class Members

# AnimalPosition Class Reference

`#include <PopulationManager.h>`

Inheritance diagram for AnimalPosition:

List of all members.

---

## Detailed Description

A class defining an animals position.

|  |
| --- |
|  |
| Public Attributes | |
| TTypesOfLandscapeElement | m\_EleType |
| TTypesOfVegetation | m\_VegType |
| unsigned | m\_x |
| unsigned | m\_y |

---

## Member Data Documentation

|  |
| --- |
| TTypesOfLandscapeElement AnimalPosition::m\_EleType |

Referenced by Population\_Manager::Probe(), and TAnimal::SupplyPosition().

|  |
| --- |
| TTypesOfVegetation AnimalPosition::m\_VegType |

Referenced by Population\_Manager::Probe(), and TAnimal::SupplyPosition().

|  |
| --- |
| unsigned AnimalPosition::m\_x |

Referenced by Vole\_Population\_Manager::DoFirst(), Population\_Manager::LOG(), Population\_Manager::Probe(), and TAnimal::SupplyPosition().

|  |
| --- |
| unsigned AnimalPosition::m\_y |

Referenced by Vole\_Population\_Manager::DoFirst(), Population\_Manager::LOG(), Population\_Manager::Probe(), and TAnimal::SupplyPosition().

---

The documentation for this class was generated from the following file:

- PopulationManager.h

---

Generated on Thu Jan 22 14:13:45 2009 for ALMaSS ODDox by 
 1.5.6 
