# Supplementary material for: Predation and fragmentation portrayed in the statistical structure of prey time series
Source: BMC Ecol. 2009 May 6;9:10. doi: 10.1186/1472-6785-9-10 (PMC2689204; doi:10.1186/1472-6785-9-10)
Supplement: Additional file 2 — Voles and related classes ODDox Documentation. ODDox documentation of the agent-based model (ALMaSS) applied by Hendrichsen et al. The documentation is started by activating main.html. [file 1472-6785-9-10-S2.zip › Vole_ODDox/class_compare_y.html]

ALMaSS ODDox: CompareY Class Reference

- Main Page
- Related Pages
- Classes
- Files

- Alphabetical List
- Class List
- Class Hierarchy
- Class Members

# CompareY Class Reference

List of all members.

---

## Detailed Description

Function to compare to TAnimal's m\_Location\_y.

|  |
| --- |
|  |
| Public Member Functions | |
| bool | operator() (TAnimal \*A1, TAnimal \*A2) const |

---

## Member Function Documentation

|  |  |  |  |
| --- | --- | --- | --- |
| bool CompareY::operator() | ( | TAnimal \* | *A1*, |
|  |  | TAnimal \* | *A2* |  |
|  | ) |  |  | const `[inline]` |

References TAnimal::Supply\_m\_Location\_y().

```
00127                                                        {
00128     return (A1->Supply_m_Location_y() < A2->Supply_m_Location_y());
00129   }
```

---

The documentation for this class was generated from the following file:

- PopulationManager.cpp

---

Generated on Thu Jan 22 14:13:45 2009 for ALMaSS ODDox by 
 1.5.6 
