# Supplementary material for: Predation and fragmentation portrayed in the statistical structure of prey time series
Source: BMC Ecol. 2009 May 6;9:10. doi: 10.1186/1472-6785-9-10 (PMC2689204; doi:10.1186/1472-6785-9-10)
Supplement: Additional file 2 — Voles and related classes ODDox Documentation. ODDox documentation of the agent-based model (ALMaSS) applied by Hendrichsen et al. The documentation is started by activating main.html. [file 1472-6785-9-10-S2.zip › Vole_ODDox/class_conventional_plant.html]

ALMaSS ODDox: ConventionalPlant Class Reference

- Main Page
- Related Pages
- Classes
- Files

- Alphabetical List
- Class List
- Class Hierarchy
- Class Members

# ConventionalPlant Class Reference

`#include <farm.h>`

Inheritance diagram for ConventionalPlant:

List of all members.

---

## Detailed Description

Inbuilt farm type.

|  |
| --- |
|  |
| Public Member Functions | |
|  | ConventionalPlant (void) |
| virtual void | MakeStockFarmer (void) |

---

## Constructor & Destructor Documentation

|  |  |  |  |  |  |
| --- | --- | --- | --- | --- | --- |
| ConventionalPlant::ConventionalPlant | ( | void |  | ) |  |

References Farm::m\_farmtype, Farm::m\_rotation, Farm::m\_stockfarmer, tof\_ConventionalPlant, tov\_FieldPeas, tov\_Setaside, tov\_SpringBarley, tov\_WinterBarley, tov\_WinterRape, tov\_WinterRye, and tov\_WinterWheat.

```
01018                                            : Farm() // 2
01019 {
01020   m_farmtype = tof_ConventionalPlant;
01021   m_stockfarmer = false;
01022 
01023   // Adjust as needed.
01024   m_rotation.resize( 9 );
01025   m_rotation[ 0 ] = tov_WinterRape;
01026   m_rotation[ 1 ] = tov_WinterWheat;
01027   m_rotation[ 2 ] = tov_SpringBarley;
01028   m_rotation[ 3 ] = tov_SpringBarley;
01029   m_rotation[ 4 ] = tov_Setaside;
01030   m_rotation[ 5 ] = tov_FieldPeas;
01031   m_rotation[ 6 ] = tov_WinterWheat;
01032   m_rotation[ 7 ] = tov_WinterRye;
01033   m_rotation[ 8 ] = tov_WinterBarley;
01034 }
```

---

## Member Function Documentation

|  |  |  |  |  |  |
| --- | --- | --- | --- | --- | --- |
| virtual void ConventionalPlant::MakeStockFarmer | ( | void |  | ) | `[inline, virtual]` |

Reimplemented from Farm.

References Farm::m\_stockfarmer.

```
00636 { m_stockfarmer = false; }
```

---

The documentation for this class was generated from the following files:

- farm.h- farm.cpp

---

Generated on Thu Jan 22 14:13:45 2009 for ALMaSS ODDox by 
 1.5.6 
