# Supplementary material for: Predation and fragmentation portrayed in the statistical structure of prey time series
Source: BMC Ecol. 2009 May 6;9:10. doi: 10.1186/1472-6785-9-10 (PMC2689204; doi:10.1186/1472-6785-9-10)
Supplement: Additional file 2 — Voles and related classes ODDox Documentation. ODDox documentation of the agent-based model (ALMaSS) applied by Hendrichsen et al. The documentation is started by activating main.html. [file 1472-6785-9-10-S2.zip › Vole_ODDox/class_conv_marginal_jord.html]

ALMaSS ODDox: ConvMarginalJord Class Reference

- Main Page
- Related Pages
- Classes
- Files

- Alphabetical List
- Class List
- Class Hierarchy
- Class Members

# ConvMarginalJord Class Reference

`#include <farm.h>`

Inheritance diagram for ConvMarginalJord:

List of all members.

---

## Detailed Description

Inbuilt special purpose farm type.

|  |
| --- |
|  |
| Public Member Functions | |
|  | ConvMarginalJord (void) |

---

## Constructor & Destructor Documentation

|  |  |  |  |  |  |
| --- | --- | --- | --- | --- | --- |
| ConvMarginalJord::ConvMarginalJord | ( | void |  | ) |  |

References Farm::m\_farmtype, Farm::m\_rotation, Farm::m\_stockfarmer, tof\_ConvMarginalJord, tov\_CloverGrassGrazed1, tov\_CloverGrassGrazed2, tov\_FodderBeet, and tov\_SpringBarleyCloverGrass.

```
01176                                          : Farm() // 9
01177 {
01178   m_farmtype = tof_ConvMarginalJord;
01179   m_stockfarmer = true;
01180 
01181   m_rotation.resize( 6 );
01182   m_rotation[ 0 ] = tov_SpringBarleyCloverGrass;
01183   m_rotation[ 1 ] = tov_CloverGrassGrazed1;
01184   m_rotation[ 2 ] = tov_CloverGrassGrazed2;
01185   m_rotation[ 3 ] = tov_SpringBarleyCloverGrass;
01186   m_rotation[ 4 ] = tov_CloverGrassGrazed1;
01187   m_rotation[ 5 ] = tov_FodderBeet;
01188 }
```

---

The documentation for this class was generated from the following files:

- farm.h- farm.cpp

---

Generated on Thu Jan 22 14:13:45 2009 for ALMaSS ODDox by 
 1.5.6 
