# Supplementary material for: Predation and fragmentation portrayed in the statistical structure of prey time series
Source: BMC Ecol. 2009 May 6;9:10. doi: 10.1186/1472-6785-9-10 (PMC2689204; doi:10.1186/1472-6785-9-10)
Supplement: Additional file 2 — Voles and related classes ODDox Documentation. ODDox documentation of the agent-based model (ALMaSS) applied by Hendrichsen et al. The documentation is started by activating main.html. [file 1472-6785-9-10-S2.zip › Vole_ODDox/class_crop.html]

ALMaSS ODDox: Crop Class Reference

- Main Page
- Related Pages
- Classes
- Files

- Alphabetical List
- Class List
- Class Hierarchy
- Class Members

# Crop Class Reference

`#include <farm.h>`

Inheritance diagram for Crop:

List of all members.

---

## Detailed Description

The base class for all crops.

|  |
| --- |
|  |
| Public Member Functions | |
| virtual bool | Do (Farm \*a\_farm, LE \*a\_field, FarmEvent \*a\_ev) |
| int | GetFirstDate (void) |
| virtual | ~Crop () |
| Protected Member Functions | |
| void | SimpleEvent (long a\_date, int a\_todo, bool a\_lock) |
|  | Adds an event to this crop management. |
| Protected Attributes | |
| int | m\_count |
| FarmEvent \* | m\_ev |
| Farm \* | m\_farm |
| LE \* | m\_field |
| int | m\_first\_date |
| int | m\_last\_date |

---

## Constructor & Destructor Documentation

|  |  |  |  |  |
| --- | --- | --- | --- | --- |
| virtual Crop::~Crop | ( |  | ) | `[inline, virtual]` |

```
00344 {}
```

---

## Member Function Documentation

|  |  |  |  |
| --- | --- | --- | --- |
| bool Crop::Do | ( | Farm \* | *a\_farm*, |
|  |  | LE \* | *a\_field*, |
|  |  | FarmEvent \* | *a\_ev* |  |
|  | ) |  |  | `[virtual]` |

Reimplemented in WinterWheat, and SetAside.

```
00191                                                                               {
00192   return true;
00193 }
```

|  |  |  |  |  |  |
| --- | --- | --- | --- | --- | --- |
| int Crop::GetFirstDate | ( | void |  | ) | `[inline]` |

References m\_first\_date.

Referenced by Farm::GetFirstDate().

```
00345 { return m_first_date; }
```

|  |  |  |  |
| --- | --- | --- | --- |
| void Crop::SimpleEvent | ( | long | *a\_date*, |
|  |  | int | *a\_todo*, |
|  |  | bool | *a\_lock* |  |
|  | ) |  |  | `[protected]` |

Adds an event to this crop management.

References Farm::AddNewEvent(), m\_farm, and m\_field.

Referenced by WinterWheat::Do(), and SetAside::Do().

```
00195                                                              {
00196 
00197   m_farm->AddNewEvent( m_field->GetVegType(), a_date, m_field, a_todo, m_field->GetRunNum(), a_lock, 0, false, (TTypesOfVegetation) 0 );
00198 }
```

---

## Member Data Documentation

|  |
| --- |
| int Crop::m\_count `[protected]` |

|  |
| --- |
| FarmEvent\* Crop::m\_ev `[protected]` |

Referenced by WinterWheat::Do(), and SetAside::Do().

|  |
| --- |
| Farm\* Crop::m\_farm `[protected]` |

Referenced by WinterWheat::Do(), SetAside::Do(), and SimpleEvent().

|  |
| --- |
| LE\* Crop::m\_field `[protected]` |

Referenced by WinterWheat::Do(), SetAside::Do(), and SimpleEvent().

|  |
| --- |
| int Crop::m\_first\_date `[protected]` |

Referenced by WinterWheat::Do(), SetAside::Do(), GetFirstDate(), SetAside::SetAside(), and WinterWheat::WinterWheat().

|  |
| --- |
| int Crop::m\_last\_date `[protected]` |

Referenced by SetAside::Do().

---

The documentation for this class was generated from the following files:

- farm.h- farm.cpp

---

Generated on Thu Jan 22 14:13:45 2009 for ALMaSS ODDox by 
 1.5.6 
