# Supplementary material for: Predation and fragmentation portrayed in the statistical structure of prey time series
Source: BMC Ecol. 2009 May 6;9:10. doi: 10.1186/1472-6785-9-10 (PMC2689204; doi:10.1186/1472-6785-9-10)
Supplement: Additional file 2 — Voles and related classes ODDox Documentation. ODDox documentation of the agent-based model (ALMaSS) applied by Hendrichsen et al. The documentation is started by activating main.html. [file 1472-6785-9-10-S2.zip › Vole_ODDox/class_crop_rotation-members.html]

ALMaSS ODDox: Member List

- Main Page
- Related Pages
- Classes
- Files

- Alphabetical List
- Class List
- Class Hierarchy
- Class Members

# CropRotation Member List

This is the complete list of members for CropRotation, including all inherited members.

|  |  |  |
| --- | --- | --- |
| CropRotation(int a\_num\_crops) | CropRotation |  |
| GetFirstCrop(int a\_farmtype, bool \*a\_low\_nutrient) | CropRotation |  |
| GetNextCrop(int a\_farmtype, int a\_current\_crop) | CropRotation |  |
| m\_rots | CropRotation | `[private]` |
| m\_start | CropRotation | `[private]` |
| ~CropRotation(void) | CropRotation |  |

---

Generated on Thu Jan 22 14:13:45 2009 for ALMaSS ODDox by 
 1.5.6 
