# Supplementary material for: Predation and fragmentation portrayed in the statistical structure of prey time series
Source: BMC Ecol. 2009 May 6;9:10. doi: 10.1186/1472-6785-9-10 (PMC2689204; doi:10.1186/1472-6785-9-10)
Supplement: Additional file 2 — Voles and related classes ODDox Documentation. ODDox documentation of the agent-based model (ALMaSS) applied by Hendrichsen et al. The documentation is started by activating main.html. [file 1472-6785-9-10-S2.zip › Vole_ODDox/class_crop_rotation.html]

ALMaSS ODDox: CropRotation Class Reference

- Main Page
- Related Pages
- Classes
- Files

- Alphabetical List
- Class List
- Class Hierarchy
- Class Members

# CropRotation Class Reference

`#include <croprotation.h>`

List of all members.

|  |
| --- |
|  |
| Public Member Functions | |
|  | CropRotation (int a\_num\_crops) |
| TTypesOfVegetation | GetFirstCrop (int a\_farmtype, bool \*a\_low\_nutrient) |
| TTypesOfVegetation | GetNextCrop (int a\_farmtype, int a\_current\_crop) |
|  | ~CropRotation (void) |
| Private Attributes | |
| vector< Rotation \* > | m\_rots |
| vector< Starter \* > | m\_start |

---

## Constructor & Destructor Documentation

|  |  |  |  |  |  |
| --- | --- | --- | --- | --- | --- |
| CropRotation::CropRotation | ( | int | *a\_num\_crops* | ) |  |

References l\_map\_rotation\_files\_prefix(), m\_rots, m\_start, and NoFarmTypes.

```
00055 {
00056   FILE *inpfile;
00057   char filename[50];
00058 
00059   m_rots.resize( a_num_crops );
00060   m_start.resize( a_num_crops );
00061   
00062   for (int i=0; i<a_num_crops; i++) {
00063     m_rots[ i ] = new Rotation;
00064     m_start[ i ] = new Starter;
00065   }
00066 
00067   for (int i=0; i<NoFarmTypes; i++) {
00068     // Now works for any number of rotations.
00069     sprintf( filename, "%sFarmType_%d.rot",
00070              l_map_rotation_files_prefix.value(),
00071              i );
00072 
00073     inpfile = fopen(filename, "r" );
00074         if (!inpfile) {
00075       g_msg->Warn( WARN_FILE, "CropRotation::CropRotation():"
00076                    " Unable to open file ", filename);
00077       exit(1);
00078     }
00079     for (int j=0; j<a_num_crops; j++) {
00080       fscanf( inpfile, "%d", (int*)&(m_rots[ j ]->CropNum[ i ]) );
00081       for (int k=0; k<4; k++) {
00082               fscanf( inpfile, "%d", (int*)&m_rots[ j ]->NewCrop[ i ][ k ] );
00083               fscanf( inpfile, "%d", &m_rots[ j ]->Percent[ i ][ k ] );
00084       }
00085     }
00086     fclose( inpfile );
00087 
00088     sprintf( filename, "FarmType_%d.stt", i );
00089     
00090      inpfile = fopen(filename, "r" );
00091          if (!inpfile) {
00092       g_msg->Warn( WARN_FILE, "CropRotation::CropRotation():"
00093                    " Unable to open file ", filename);
00094       exit(1);
00095     }
00096     for (int j=0; j<a_num_crops; j++) {
00097       fscanf( inpfile, "%d %d",
00098               (int*)&m_start[ j ]->CropNum[ i ],
00099               &m_start[ j ]->Percent[ i ] );
00100     }
00101     fclose(inpfile);
00102   }
00103 }
```

|  |  |  |  |  |  |
| --- | --- | --- | --- | --- | --- |
| CropRotation::~CropRotation | ( | void |  | ) |  |

References m\_rots, and m\_start.

```
00108 {
00109   for (unsigned int i=0; i<m_rots.size(); i++) {
00110     delete m_rots[ i ];
00111     delete m_start[ i ];
00112   }
00113 }
```

---

## Member Function Documentation

|  |  |  |  |
| --- | --- | --- | --- |
| TTypesOfVegetation CropRotation::GetFirstCrop | ( | int | *a\_farmtype*, |
|  |  | bool \* | *a\_low\_nutrient* |  |
|  | ) |  |  |  |

References m\_start.

```
00139 {
00140   int num_crops = g_crops->GetNumCrops();
00141 
00142   for ( int i=0; i<num_crops; i++ ) {
00143     int percent = m_start[ i ]->Percent[ a_farmtype ];
00144 
00145     if ( (num_crops==i) ||
00146          (-1 == m_start[ i+1 ]->Percent[ a_farmtype ]) ||
00147          (rand()%100 < percent) ) {
00148       *a_low_nutrient = g_crops->GetNutStatus( i );
00149       return (TTypesOfVegetation) i;
00150     }
00151   }
00152   g_msg->Warn( WARN_BUG, "CropRotation::GetFirstCrop():"
00153                " Unable to recover crop type?!", "");
00154   exit(1);
00155 }
```

|  |  |  |  |
| --- | --- | --- | --- |
| TTypesOfVegetation CropRotation::GetNextCrop | ( | int | *a\_farmtype*, |
|  |  | int | *a\_current\_crop* |  |
|  | ) |  |  |  |

References m\_rots.

```
00118 {
00119   //int NumCrops = g_crops->GetNumCrops();
00120   for ( int i=0; i<4; i++ ) {
00121     int percent = m_rots[ a_current_crop ]->Percent[ a_farmtype ][ i ];
00122 
00123     // Lazy evaluation for experts. ;-)
00124     if ( (3==i) ||
00125          (-1 == m_rots[ a_current_crop ]->Percent[ a_farmtype ][ i+1 ]) ||
00126          (rand()%100 < percent) ) {
00127       return m_rots[ a_current_crop ]->NewCrop[ a_farmtype ][ i ];
00128     }
00129   }
00130   g_msg->Warn( WARN_BUG, "CropRotation::GetNextCrop():"
00131                " Unable to recover crop type?!", "");
00132   exit(1);
00133 }
```

---

## Member Data Documentation

|  |
| --- |
| vector< Rotation\* > CropRotation::m\_rots `[private]` |

Referenced by CropRotation(), GetNextCrop(), and ~CropRotation().

|  |
| --- |
| vector< Starter\* > CropRotation::m\_start `[private]` |

Referenced by CropRotation(), GetFirstCrop(), and ~CropRotation().

---

The documentation for this class was generated from the following files:

- croprotation.h- croprotation.cpp

---

Generated on Thu Jan 22 14:13:45 2009 for ALMaSS ODDox by 
 1.5.6 
