# Supplementary material for: Predation and fragmentation portrayed in the statistical structure of prey time series
Source: BMC Ecol. 2009 May 6;9:10. doi: 10.1186/1472-6785-9-10 (PMC2689204; doi:10.1186/1472-6785-9-10)
Supplement: Additional file 2 — Voles and related classes ODDox Documentation. ODDox documentation of the agent-based model (ALMaSS) applied by Hendrichsen et al. The documentation is started by activating main.html. [file 1472-6785-9-10-S2.zip › Vole_ODDox/class_farm.html]

ALMaSS ODDox: Farm Class Reference

- Main Page
- Related Pages
- Classes
- Files

- Alphabetical List
- Class List
- Class Hierarchy
- Class Members

# Farm Class Reference

`#include <farm.h>`

Inheritance diagram for Farm:

List of all members.

|  |
| --- |
|  |
| Public Member Functions | |
| void | AddField (LE \*a\_newfield) |
|  | Adds a field to a farm. |
| void | AddNewEvent (TTypesOfVegetation a\_event, long a\_date, LE \*a\_field, int a\_todo, long a\_num, bool a\_lock, int a\_start, bool a\_first\_year, TTypesOfVegetation a\_crop) |
|  | Adds an event to the event queue for a farm. |
| virtual bool | AutumnHarrow (LE \*a\_field, double a\_user, int a\_days) |
|  | Carry out a harrow event in the autumn on a\_field. |
| virtual bool | AutumnPlough (LE \*a\_field, double a\_user, int a\_days) |
|  | Carry out a ploughing event in the autumn on a\_field. |
| virtual bool | AutumnRoll (LE \*a\_field, double a\_user, int a\_days) |
|  | Carry out a roll event in the autumn on a\_field. |
| virtual bool | AutumnSow (LE \*a\_field, double a\_user, int a\_days) |
|  | Carry out a sowing event in the autumn on a\_field. |
| virtual bool | BurnStrawStubble (LE \*a\_field, double a\_user, int a\_days) |
|  | Burn stubble on a\_field. |
| virtual bool | CattleIsOut (LE \*a\_field, double a\_user, int a\_days, int a\_max) |
|  | Generate a 'cattle\_out' event for every day the cattle are on a\_field. |
| virtual bool | CattleIsOutLow (LE \*a\_field, double a\_user, int a\_days, int a\_max) |
|  | Generate a 'cattle\_out\_low' event for every day the cattle are on a\_field. |
| virtual bool | CattleOut (LE \*a\_field, double a\_user, int a\_days) |
|  | Start a grazing event on a\_field today. |
| virtual bool | CattleOutLowGrazing (LE \*a\_field, double a\_user, int a\_days) |
|  | Start a extensive grazing event on a\_field today. |
| virtual bool | CutToHay (LE \*a\_field, double a\_user, int a\_days) |
|  | Carry out hay cutting on a\_field. |
| virtual bool | CutToSilage (LE \*a\_field, double a\_user, int a\_days) |
|  | Cut vegetation for silage on a\_field. |
| virtual bool | CutWeeds (LE \*a\_field, double a\_user, int a\_days) |
|  | Carry out weed topping on a\_field. |
| virtual bool | DeepPlough (LE \*a\_field, double a\_user, int a\_days) |
|  | Carry out a deep ploughing event on a\_field. |
| bool | DoIt (int a\_probability) |
|  | Return chance out of 100. |
| virtual bool | FA\_AmmoniumSulphate (LE \*a\_field, double a\_user, int a\_days) |
|  | Apply ammonium supahte to a\_field owned by an stock farmer. |
| virtual bool | FA\_GreenManure (LE \*a\_field, double a\_user, int a\_days) |
|  | Spread green manure on a\_field owned by an stock farmer. |
| virtual bool | FA\_Manure (LE \*a\_field, double a\_user, int a\_days) |
|  | Spread manure on a\_field owned by an stock farmer. |
| virtual bool | FA\_NPK (LE \*a\_field, double a\_user, int a\_days) |
|  | Apply NPK fertilizer to a\_field owned by an stock farmer. |
| virtual bool | FA\_PK (LE \*a\_field, double a\_user, int a\_days) |
|  | Apply PK fertilizer to a\_field owned by an stock farmer. |
| virtual bool | FA\_Sludge (LE \*a\_field, double a\_user, int a\_days) |
|  | Spread sewege sludge on a\_field owned by an stock farmer. |
| virtual bool | FA\_Slurry (LE \*a\_field, double a\_user, int a\_days) |
|  | Spready slurry on a\_field owned by an stock farmer. |
|  | Farm (void) |
|  | Farm constructor - creates an instance of each possible crop type. |
| virtual bool | FP\_GreenManure (LE \*a\_field, double a\_user, int a\_days) |
|  | Spread green manure on a\_field owned by an arable farmer. |
| virtual bool | FP\_LiquidNH3 (LE \*a\_field, double a\_user, int a\_days) |
|  | Apply liquid ammonia fertilizer to a\_field owned by an arable farmer. |
| virtual bool | FP\_ManganeseSulphate (LE \*a\_field, double a\_user, int a\_days) |
|  | Apply Manganse Sulphate to a\_field owned by an arable farmer. |
| virtual bool | FP\_Manure (LE \*a\_field, double a\_user, int a\_days) |
|  | Spread manure on a\_field owned by an arable farmer. |
| virtual bool | FP\_NPK (LE \*a\_field, double a\_user, int a\_days) |
|  | Apply NPK fertilizer, on a\_field owned by an arable farmer. |
| virtual bool | FP\_NPKS (LE \*a\_field, double a\_user, int a\_days) |
|  | Apply NPKS fertilizer, on a\_field owned by an arable farmer. |
| virtual bool | FP\_PK (LE \*a\_field, double a\_user, int a\_days) |
|  | Apply PK fertilizer, on a\_field owned by an arable farmer. |
| virtual bool | FP\_Sludge (LE \*a\_field, double a\_user, int a\_days) |
|  | Spread sewege on a\_field owned by an arable farmer. |
| virtual bool | FP\_Slurry (LE \*a\_field, double a\_user, int a\_days) |
|  | Apply slurry to a\_field owned by an arable farmer. |
| virtual bool | FungicideTreat (LE \*a\_field, double a\_user, int a\_days) |
|  | Apply fungicide to a\_field. |
| int | GetFarmNumber (void) |
| bool | GetIntensity (void) |
| TTypesOfFarm | GetType (void) |
| virtual bool | Glyphosate (LE \*a\_field, double a\_user, int a\_days) |
| virtual bool | GrowthRegulator (LE \*a\_field, double a\_user, int a\_days) |
|  | Apply growth regulator to a\_field. |
| virtual bool | Harvest (LE \*a\_field, double a\_user, int a\_days) |
|  | Carry out a harvest on a\_field. |
| virtual bool | HayBailing (LE \*a\_field, double a\_user, int a\_days) |
|  | Carry out hay bailing on a\_field. |
| virtual bool | HayTurning (LE \*a\_field, double a\_user, int a\_days) |
|  | Carry out hay turning on a\_field. |
| virtual bool | HerbicideTreat (LE \*a\_field, double a\_user, int a\_days) |
|  | Apply herbicide to a\_field. |
| virtual bool | HillingUp (LE \*a\_field, double a\_user, int a\_days) |
|  | Do hilling up on a\_field, probably of potatoes. |
| virtual void | InitiateManagement (void) |
|  | Kicks of the farm's management. |
| virtual bool | InsecticideTreat (LE \*a\_field, double a\_user, int a\_days) |
|  | Apply insecticide to a\_field. |
| bool | IsStockFarmer (void) |
| virtual void | MakeStockFarmer (void) |
| virtual void | Management (void) |
|  | Starts the main management loop for the farm and performs some error checking. |
| virtual bool | Molluscicide (LE \*a\_field, double a\_user, int a\_days) |
|  | Apply molluscidie to a\_field. |
| virtual bool | PigsAreOut (LE \*a\_field, double a\_user, int a\_days) |
|  | Start a pig grazing event on a\_field today or soon. |
| virtual bool | PigsAreOutForced (LE \*a\_field, double a\_user, int a\_days) |
|  | Start a pig grazing event on a\_field today - no exceptions. |
| virtual bool | PigsOut (LE \*a\_field, double a\_user, int a\_days) |
|  | Generate a 'pigs\_out' event for every day the cattle are on a\_field. |
| virtual bool | ProductApplication0 (LE \*a\_field, double a\_user, int a\_days) |
|  | Apply test pesticide to a\_field. |
| virtual bool | ProductApplication1 (LE \*a\_field, double a\_user, int a\_days) |
|  | Apply test pesticide to a\_field. |
| void | RemoveField (LE \*a\_field) |
|  | Removes a field to a farm. |
| virtual bool | RowCultivation (LE \*a\_field, double a\_user, int a\_days) |
|  | Carry out a harrowing between crop rows on a\_field. |
| void | SetFarmNumber (int a\_farm\_num) |
| virtual bool | SleepAllDay (LE \*a\_field, double a\_user, int a\_days) |
|  | Nothing to to today on a\_field. |
| virtual bool | SpringHarrow (LE \*a\_field, double a\_user, int a\_days) |
|  | Carry out a harrow event in the spring on a\_field. |
| virtual bool | SpringPlough (LE \*a\_field, double a\_user, int a\_days) |
|  | Carry out a ploughing event in the spring on a\_field. |
| virtual bool | SpringRoll (LE \*a\_field, double a\_user, int a\_days) |
|  | Carry out a roll event in the spring on a\_field. |
| virtual bool | SpringSow (LE \*a\_field, double a\_user, int a\_days) |
|  | Carry out a sowing event in the spring on a\_field. |
| virtual bool | StrawChopping (LE \*a\_field, double a\_user, int a\_days) |
|  | Carry out straw chopping on a\_field. |
| virtual bool | Strigling (LE \*a\_field, double a\_user, int a\_days) |
|  | Carry out a mechanical weeding on a\_field. |
| virtual bool | StriglingSow (LE \*a\_field, double a\_user, int a\_days) |
|  | Carry out a mechanical weeding followed by sowing on a\_field. |
| virtual bool | StubbleHarrowing (LE \*a\_field, double a\_user, int a\_days) |
|  | Carry out stubble harrowing on a\_field. |
| virtual bool | Swathing (LE \*a\_field, double a\_user, int a\_days) |
|  | Cut the crop on a\_field and leave it lying (probably rape). |
| virtual bool | SynInsecticideTreat (LE \*a\_field, double a\_user, int a\_days) |
|  | Apply special insecticide to a\_field. |
| TTypesOfVegetation | TranslateCropCodes (const char \*str) |
| virtual bool | Trial\_Control (LE \*a\_field, double a\_user, int a\_days) |
|  | Special pesticide trial functionality. |
| virtual bool | Trial\_InsecticideTreat (LE \*a\_field, double a\_user, int a\_days) |
|  | Special pesticide trial functionality. |
| virtual bool | Trial\_ToxicControl (LE \*a\_field, double a\_user, int a\_days) |
|  | Special pesticide trial functionality. |
| virtual bool | Water (LE \*a\_field, double a\_user, int a\_days) |
|  | Carry out a watering on a\_field. |
| virtual bool | WinterPlough (LE \*a\_field, double a\_user, int a\_days) |
|  | Carry out a ploughing event in the winter on a\_field. |
| virtual | ~Farm (void) |
|  | Farm destructor - deletes all crop instances and empties event queues. |
| Protected Member Functions | |
| void | CheckRotationManagementLoop (FarmEvent \*ev) |
| virtual int | GetFirstCropIndex (TTypesOfLandscapeElement a\_type) |
|  | Gets the first crop for the farm. |
| int | GetFirstDate (TTypesOfVegetation a\_tov) |
|  | Gets the start date for a crop type. |
| virtual int | GetNextCropIndex (int a\_rot\_index) |
|  | Returns the next crop in the rotation. |
| int | GetNextCropStartDate (LE \*a\_field, TTypesOfVegetation &a\_curr\_veg) |
|  | Returns the start date of the next crop in the rotation. |
| void | HandleEvents (void) |
|  | If there are events to carry out do this, and perhaps start a new crop. |
| bool | LeSwitch (FarmEvent \*ev) |
|  | Call do function for any crop with an outstanding event. Signal if the crop has terminated. |
| Protected Attributes | |
| AgroChemIndustryCereal \* | m\_agrochemindustrycereal |
| Carrots \* | m\_carrots |
| CloverGrassGrazed1 \* | m\_CGG1 |
| CloverGrassGrazed2 \* | m\_CGG2 |
| int | m\_farm\_num |
| TTypesOfFarm | m\_farmtype |
| FieldPeas \* | m\_fieldpeas |
| FieldPeasStrigling \* | m\_fieldpeasstrigling |
| vector< LE \* > | m\_fields |
| Fodderbeet \* | m\_fodderbeet |
| bool | m\_intensity |
| Maize \* | m\_maize |
| MaizeStrigling \* | m\_maizestrigling |
| Oats \* | m\_oats |
| OBarleyPeaCloverGrass \* | m\_OBarleyPCG |
| OCarrots \* | m\_ocarrots |
| OCloverGrassGrazed1 \* | m\_OCGG1 |
| OCloverGrassGrazed2 \* | m\_OCGG2 |
| OCloverGrassSilage1 \* | m\_OCGS1 |
| OFieldPeas \* | m\_ofieldpeas |
| OFieldPeasSilage \* | m\_ofieldpeassilage |
| OFirstYearDanger \* | m\_ofirstyeardanger |
| OGrazingPigs \* | m\_ograzingpigs |
| OOats \* | m\_ooats |
| OPermanentGrassGrazed \* | m\_opermgrassgrazed |
| OPotatoes \* | m\_opotatoes |
| OSBarleySilage \* | m\_OSBarleysilage |
| OSpringBarley \* | m\_ospringbarley |
| OSpringBarleyPigs \* | m\_ospringbarleypigs |
| OWinterBarley \* | m\_owinterbarley |
| OWinterRape \* | m\_owinterrape |
| OWinterRye \* | m\_owinterrye |
| OWinterWheatUndersown \* | m\_owinterwheatundersown |
| PermanentSetAside \* | m\_permanentsetaside |
| PermanentGrassGrazed \* | m\_permgrassgrazed |
| PermanentGrassLowGrazed \* | m\_permgrasslowgrazed |
| Potatoes \* | m\_potatoes |
| LowPriority< FarmEvent \* > | m\_queue |
| vector< TTypesOfVegetation > | m\_rotation |
| int | m\_rotation\_sync\_index |
| SpringBarleyCloverGrass \* | m\_sbarleyclovergrass |
| SeedGrass1 \* | m\_seedgrass1 |
| SeedGrass2 \* | m\_seedgrass2 |
| SetAside \* | m\_setaside |
| SpringBarley \* | m\_springbarley |
| SpringBarleyCloverGrassStrigling \* | m\_springbarleyclovergrassstrigling |
| SpringBarleyPeaCloverGrassStrigling \* | m\_springbarleypeaclovergrassstrigling |
| SpringBarleySeed \* | m\_springbarleyseed |
| SpringBarleySilage \* | m\_springbarleysilage |
| SpringBarleyStrigling \* | m\_springbarleystrigling |
| SpringBarleyStriglingCulm \* | m\_springbarleystriglingculm |
| SpringBarleyStriglingSingle \* | m\_springbarleystriglingsingle |
| bool | m\_stockfarmer |
| Triticale \* | m\_triticale |
| WinterBarley \* | m\_winterbarley |
| WinterBarleyStrigling \* | m\_winterbarleystrigling |
| WinterRape \* | m\_winterrape |
| WinterRapeStrigling \* | m\_winterrapestrigling |
| WinterRye \* | m\_winterrye |
| WinterRyeStrigling \* | m\_winterryestrigling |
| WinterWheat \* | m\_winterwheat |
| WinterWheatStrigling \* | m\_winterwheatstrigling |
| WinterWheatStriglingCulm \* | m\_winterwheatstriglingculm |
| WinterWheatStriglingSingle \* | m\_winterwheatstriglingsingle |
| WWheatPControl \* | m\_wwheatpcontrol |
| WWheatPToxicControl \* | m\_wwheatptoxiccontrol |
| WWheatPTreatment \* | m\_wwheatptreatment |
| YoungForestCrop \* | m\_youngforest |

---

## Constructor & Destructor Documentation

|  |  |  |  |  |  |
| --- | --- | --- | --- | --- | --- |
| Farm::Farm | ( | void |  | ) |  |

Farm constructor - creates an instance of each possible crop type.

References m\_agrochemindustrycereal, m\_carrots, m\_CGG1, m\_CGG2, m\_fieldpeas, m\_fieldpeasstrigling, m\_fodderbeet, m\_intensity, m\_maize, m\_maizestrigling, m\_oats, m\_OBarleyPCG, m\_ocarrots, m\_OCGG1, m\_OCGG2, m\_OCGS1, m\_ofieldpeas, m\_ofieldpeassilage, m\_ofirstyeardanger, m\_ograzingpigs, m\_ooats, m\_opermgrassgrazed, m\_opotatoes, m\_OSBarleysilage, m\_ospringbarley, m\_ospringbarleypigs, m\_owinterbarley, m\_owinterrape, m\_owinterrye, m\_owinterwheatundersown, m\_permanentsetaside, m\_permgrassgrazed, m\_permgrasslowgrazed, m\_potatoes, m\_rotation\_sync\_index, m\_sbarleyclovergrass, m\_seedgrass1, m\_seedgrass2, m\_setaside, m\_springbarley, m\_springbarleyclovergrassstrigling, m\_springbarleypeaclovergrassstrigling, m\_springbarleyseed, m\_springbarleysilage, m\_springbarleystrigling, m\_springbarleystriglingculm, m\_springbarleystriglingsingle, m\_stockfarmer, m\_triticale, m\_winterbarley, m\_winterbarleystrigling, m\_winterrape, m\_winterrapestrigling, m\_winterrye, m\_winterryestrigling, m\_winterwheat, m\_winterwheatstrigling, m\_winterwheatstriglingculm, m\_winterwheatstriglingsingle, m\_wwheatpcontrol, m\_wwheatptoxiccontrol, m\_wwheatptreatment, and m\_youngforest.

```
00574                  {
00575   m_carrots = new Carrots;
00576   m_CGG1 = new CloverGrassGrazed1;
00577   m_CGG2 = new CloverGrassGrazed2;
00578   m_fieldpeas = new FieldPeas;
00579   m_fodderbeet = new Fodderbeet;
00580   m_maize = new Maize;
00581   m_OBarleyPCG = new OBarleyPeaCloverGrass;
00582   m_OSBarleysilage = new OSBarleySilage;
00583   m_ocarrots = new OCarrots;
00584   m_OCGG1 = new OCloverGrassGrazed1;
00585   m_OCGG2 = new OCloverGrassGrazed2;
00586   m_OCGS1 = new OCloverGrassSilage1;
00587   m_ofieldpeas = new OFieldPeas;
00588   m_ofieldpeassilage = new OFieldPeasSilage;
00589   m_ofirstyeardanger = new OFirstYearDanger;
00590   m_ograzingpigs = new OGrazingPigs;
00591   m_oats = new Oats;
00592   m_ooats = new OOats;
00593   m_opermgrassgrazed = new OPermanentGrassGrazed;
00594   m_opotatoes = new OPotatoes;
00595   m_ospringbarley = new OSpringBarley;
00596   m_ospringbarleypigs = new OSpringBarleyPigs;
00597   m_owinterbarley = new OWinterBarley;
00598   m_owinterrape = new OWinterRape;
00599   m_owinterrye = new OWinterRye;
00600   m_owinterwheatundersown = new OWinterWheatUndersown;
00601   m_permanentsetaside = new PermanentSetAside;
00602   m_permgrassgrazed = new PermanentGrassGrazed;
00603   m_permgrasslowgrazed = new PermanentGrassLowGrazed;
00604   m_potatoes = new Potatoes;
00605   m_sbarleyclovergrass = new SpringBarleyCloverGrass;
00606   m_seedgrass1 = new SeedGrass1;
00607   m_seedgrass2 = new SeedGrass2;
00608   m_setaside = new SetAside;
00609   m_springbarley = new SpringBarley;
00610   m_springbarleyseed = new SpringBarleySeed;
00611   m_springbarleysilage = new SpringBarleySilage;
00612   m_triticale = new Triticale;
00613   m_winterbarley = new WinterBarley;
00614   m_winterrape = new WinterRape;
00615   m_winterrye = new WinterRye;
00616   m_winterwheat = new WinterWheat;
00617   m_wwheatpcontrol = new WWheatPControl;
00618   m_wwheatptoxiccontrol = new WWheatPToxicControl;
00619   m_wwheatptreatment = new WWheatPTreatment;
00620   m_agrochemindustrycereal = new AgroChemIndustryCereal;
00621   m_winterwheatstrigling = new WinterWheatStrigling;
00622   m_winterwheatstriglingsingle = new WinterWheatStriglingSingle;
00623   m_winterwheatstriglingculm = new WinterWheatStriglingCulm;
00624   m_springbarleyclovergrassstrigling = new SpringBarleyCloverGrassStrigling;
00625   m_springbarleystrigling = new SpringBarleyStrigling;
00626   m_springbarleystriglingsingle = new SpringBarleyStriglingSingle;
00627   m_springbarleystriglingculm = new SpringBarleyStriglingCulm;
00628   m_maizestrigling = new MaizeStrigling;
00629   m_winterrapestrigling = new WinterRapeStrigling;
00630   m_winterryestrigling = new WinterRyeStrigling;
00631   m_winterbarleystrigling = new WinterBarleyStrigling;
00632   m_fieldpeasstrigling = new FieldPeasStrigling;
00633   m_springbarleypeaclovergrassstrigling = new SpringBarleyPeaCloverGrassStrigling;
00634   m_youngforest = new YoungForestCrop;
00635   m_stockfarmer = false;
00636   m_rotation_sync_index = -1;
00637   m_intensity = (random(2)==0);
00638 }
```

|  |  |  |  |  |  |
| --- | --- | --- | --- | --- | --- |
| Farm::~Farm | ( | void |  | ) | `[virtual]` |

Farm destructor - deletes all crop instances and empties event queues.

References m\_agrochemindustrycereal, m\_carrots, m\_CGG1, m\_CGG2, m\_fieldpeas, m\_fieldpeasstrigling, m\_fodderbeet, m\_maize, m\_maizestrigling, m\_oats, m\_OBarleyPCG, m\_ocarrots, m\_OCGG1, m\_OCGG2, m\_OCGS1, m\_ofieldpeas, m\_ofieldpeassilage, m\_ofirstyeardanger, m\_ograzingpigs, m\_ooats, m\_opermgrassgrazed, m\_opotatoes, m\_OSBarleysilage, m\_ospringbarley, m\_ospringbarleypigs, m\_owinterbarley, m\_owinterrape, m\_owinterrye, m\_owinterwheatundersown, m\_permanentsetaside, m\_permgrassgrazed, m\_permgrasslowgrazed, m\_potatoes, m\_queue, m\_sbarleyclovergrass, m\_seedgrass1, m\_seedgrass2, m\_setaside, m\_springbarley, m\_springbarleyclovergrassstrigling, m\_springbarleypeaclovergrassstrigling, m\_springbarleyseed, m\_springbarleysilage, m\_springbarleystrigling, m\_springbarleystriglingculm, m\_springbarleystriglingsingle, m\_triticale, m\_winterbarley, m\_winterbarleystrigling, m\_winterrape, m\_winterrapestrigling, m\_winterrye, m\_winterryestrigling, m\_winterwheat, m\_winterwheatstrigling, m\_winterwheatstriglingculm, m\_winterwheatstriglingsingle, m\_wwheatpcontrol, m\_wwheatptoxiccontrol, m\_wwheatptreatment, and m\_youngforest.

```
00645                   {
00646   delete m_carrots;
00647   delete m_CGG2;
00648   delete m_CGG1;
00649   delete m_fieldpeas;
00650   delete m_fodderbeet;
00651   delete m_maize;
00652   delete m_ocarrots;
00653   delete m_OCGG1;
00654   delete m_OCGG2;
00655   delete m_OCGS1;
00656   delete m_ofieldpeas;
00657   delete m_ofieldpeassilage;
00658   delete m_ofirstyeardanger;
00659   delete m_ograzingpigs;
00660   delete m_ooats;
00661   delete m_oats;
00662   delete m_opermgrassgrazed;
00663   delete m_opotatoes;
00664   delete m_ospringbarley;
00665   delete m_ospringbarleypigs;
00666   delete m_owinterbarley;
00667   delete m_owinterrape;
00668   delete m_owinterrye;
00669   delete m_owinterwheatundersown;
00670   delete m_OBarleyPCG;
00671   delete m_OSBarleysilage;
00672   delete m_permanentsetaside;
00673   delete m_permgrassgrazed;
00674   delete m_permgrasslowgrazed;
00675   delete m_potatoes;
00676   delete m_sbarleyclovergrass;
00677   delete m_seedgrass2;
00678   delete m_seedgrass1;
00679   delete m_setaside;
00680   delete m_springbarley;
00681   delete m_springbarleyseed;
00682   delete m_springbarleysilage;
00683   delete m_agrochemindustrycereal;
00684   delete m_triticale;
00685   delete m_winterbarley;
00686   delete m_winterrape;
00687   delete m_winterrye;
00688   delete m_winterwheat;
00689   delete m_wwheatpcontrol;
00690   delete m_wwheatptoxiccontrol;
00691   delete m_wwheatptreatment;
00692   delete  m_winterwheatstrigling;
00693   delete  m_winterwheatstriglingsingle;
00694   delete  m_winterwheatstriglingculm;
00695   delete  m_springbarleyclovergrassstrigling;
00696   delete  m_springbarleystrigling;
00697   delete  m_springbarleystriglingsingle;
00698   delete  m_springbarleystriglingculm;
00699   delete  m_maizestrigling;
00700   delete  m_winterrapestrigling;
00701   delete  m_winterryestrigling;
00702   delete  m_winterbarleystrigling;
00703   delete  m_fieldpeasstrigling;
00704   delete  m_springbarleypeaclovergrassstrigling;
00705   delete  m_youngforest;
00706   LowPriPair < FarmEvent * > pair;
00707 
00708   while ( !m_queue.Empty() ) {
00709     pair = m_queue.Bottom();
00710     m_queue.Pop();
00711     delete pair.m_element;
00712   }
00713 }
```

---

## Member Function Documentation

|  |  |  |  |  |  |
| --- | --- | --- | --- | --- | --- |
| void Farm::AddField | ( | LE \* | *a\_newfield* | ) |  |

Adds a field to a farm.

References m\_fields.

```
00786                                      {
00787   int i = (int) m_fields.size();
00788 
00789   m_fields.resize( i + 1 );
00790   m_fields[ i ] = a_newfield;
00791 }
```

|  |  |  |  |
| --- | --- | --- | --- |
| void Farm::AddNewEvent | ( | TTypesOfVegetation | *a\_event*, |
|  |  | long | *a\_date*, |
|  |  | LE \* | *a\_field*, |
|  |  | int | *a\_todo*, |
|  |  | long | *a\_num*, |
|  |  | bool | *a\_lock*, |
|  |  | int | *a\_start*, |
|  |  | bool | *a\_first\_year*, |
|  |  | TTypesOfVegetation | *a\_crop* |  |
|  | ) |  |  |  |

Adds an event to the event queue for a farm.

References m\_queue.

Referenced by HandleEvents(), InitiateManagement(), and Crop::SimpleEvent().

```
00433                                                                  {
00434 
00435        FarmEvent * ev = new FarmEvent( a_event, a_field, a_todo, a_run, a_lock, a_start, a_first_year, a_crop );
00436        m_queue.Push( ev, a_date );
00437 }
```

|  |  |  |  |
| --- | --- | --- | --- |
| bool Farm::AutumnHarrow | ( | LE \* | *a\_field*, |
|  |  | double | *a\_user*, |
|  |  | int | *a\_days* |  |
|  | ) |  |  | `[virtual]` |

Carry out a harrow event in the autumn on a\_field.

References autumn\_harrow, DO\_IT\_PROB, DoIt(), g\_landscape\_p, and UNREFERENCED\_PARAMETER.

Referenced by WinterWheat::Do().

```
00143 {
00144     UNREFERENCED_PARAMETER( a_user );
00145   if ( (0 >= a_days) || (!g_weather->Raining() && DoIt(DO_IT_PROB))) {
00146     a_field->Trace( autumn_harrow );
00147     a_field->SetLastTreatment( autumn_harrow );
00148    // Apply 90% mortality to the insects
00149     a_field->InsectMortality( 0.1 );
00150    // Reduce the vegetation to zero
00151     a_field->ZeroVeg();
00152     int pref=a_field->GetUnsprayedMarginPolyRef();
00153     if (pref!=-1){
00154       // Must have an unsprayed margin so need to pass the information on to it
00155       LE* um=g_landscape_p->SupplyLEPointer(pref);
00156       um->SetLastTreatment( autumn_harrow );
00157       um->InsectMortality( 0.1 );
00158       um->ZeroVeg();
00159     }
00160     return true;
00161   }
00162   return false;
00163 }
```

|  |  |  |  |
| --- | --- | --- | --- |
| bool Farm::AutumnPlough | ( | LE \* | *a\_field*, |
|  |  | double | *a\_user*, |
|  |  | int | *a\_days* |  |
|  | ) |  |  | `[virtual]` |

Carry out a ploughing event in the autumn on a\_field.

References autumn\_plough, DO\_IT\_PROB, DoIt(), g\_landscape\_p, and UNREFERENCED\_PARAMETER.

Referenced by WinterWheat::Do().

```
00088 {
00089         UNREFERENCED_PARAMETER( a_user );
00090         // LE is a pointer to the field element
00091         // a_user is a pointer to the farm
00092         // a_days is the end of the operation time - today
00093         // if a_days <0 then the time to do it is passed
00094         // the line below reads 'plough if last day possible OR if not raining and pass a probability test
00095         if ( (0 >= a_days) || (!g_weather->Raining() && DoIt(DO_IT_PROB))) {
00096                 // this bit sets up the events to occur when ploughing occurs
00097                 // The trace below is for debugging checks
00098                 a_field->Trace( autumn_plough );
00099                 // Record the event for this field, so other objects can find out it has happened
00100                 a_field->SetLastTreatment( autumn_plough );
00101                 // Apply mortality to the insects present, in this case 90%. This only affects the general insect model, any ALMaSS model species need to take their specific action.
00102                 a_field->InsectMortality( 0.1 );
00103                 // Reduce the vegetation, in this case to zero
00104                 a_field->ZeroVeg();
00105                 // If the field has a field margin, then do all this to the field margin too. In events that don't occur on an unsprayed margin, e.g. insecticide, then is part is skipped.
00106                 int pref=a_field->GetUnsprayedMarginPolyRef();
00107                 if (pref!=-1){
00108                         // Must have an unsprayed margin so need to pass the information on to it
00109                         LE* um=g_landscape_p->SupplyLEPointer(pref);
00110                         um->SetLastTreatment( autumn_plough );
00111                         um->InsectMortality( 0.1 );
00112                         um->ZeroVeg();
00113                 }
00114                 return true;      // completed
00115         }
00116         return false;       // not completed
00117 }
```

|  |  |  |  |
| --- | --- | --- | --- |
| bool Farm::AutumnRoll | ( | LE \* | *a\_field*, |
|  |  | double | *a\_user*, |
|  |  | int | *a\_days* |  |
|  | ) |  |  | `[virtual]` |

Carry out a roll event in the autumn on a\_field.

References autumn\_roll, DO\_IT\_PROB, DoIt(), g\_landscape\_p, and UNREFERENCED\_PARAMETER.

Referenced by WinterWheat::Do().

```
00170 {
00171     UNREFERENCED_PARAMETER( a_user );
00172   if ( (0 >= a_days) || (!g_weather->Raining() && DoIt(DO_IT_PROB))) {
00173     a_field->Trace( autumn_roll );
00174     a_field->SetLastTreatment( autumn_roll );
00175     a_field->ZeroVeg();
00176     int pref=a_field->GetUnsprayedMarginPolyRef();
00177     if (pref!=-1){
00178       // Must have an unsprayed margin so need to pass the information on to it
00179       LE* um=g_landscape_p->SupplyLEPointer(pref);
00180       um->SetLastTreatment( autumn_roll );
00181       um->ZeroVeg();
00182     }
00183     return true;
00184   }
00185   return false;
00186 }
```

|  |  |  |  |
| --- | --- | --- | --- |
| bool Farm::AutumnSow | ( | LE \* | *a\_field*, |
|  |  | double | *a\_user*, |
|  |  | int | *a\_days* |  |
|  | ) |  |  | `[virtual]` |

Carry out a sowing event in the autumn on a\_field.

References autumn\_sow, DO\_IT\_PROB, DoIt(), g\_landscape\_p, and UNREFERENCED\_PARAMETER.

Referenced by WinterWheat::Do().

```
00193 {
00194     UNREFERENCED_PARAMETER( a_user );
00195   if ( (0 >= a_days) || (!g_weather->Raining() && DoIt(DO_IT_PROB))) {
00196     a_field->Trace( autumn_sow );
00197     a_field->SetLastTreatment( autumn_sow );
00198     a_field->SetGrowthPhase( sow );
00199    // Reduce the vegetation to zero
00200     a_field->ZeroVeg();
00201     int pref=a_field->GetUnsprayedMarginPolyRef();
00202     if (pref!=-1){
00203       // Must have an unsprayed margin so need to pass the information on to it
00204       LE* um=g_landscape_p->SupplyLEPointer(pref);
00205       um->SetLastTreatment( autumn_sow );
00206       um->SetGrowthPhase( sow );
00207       um->ZeroVeg();
00208     }
00209     return true;
00210   }
00211   return false;
00212 }
```

|  |  |  |  |
| --- | --- | --- | --- |
| bool Farm::BurnStrawStubble | ( | LE \* | *a\_field*, |
|  |  | double | *a\_user*, |
|  |  | int | *a\_days* |  |
|  | ) |  |  | `[virtual]` |

Burn stubble on a\_field.

References burn\_straw\_stubble, DO\_IT\_PROB, DoIt(), g\_landscape\_p, and UNREFERENCED\_PARAMETER.

```
01585 {
01586   UNREFERENCED_PARAMETER( a_user );
01587   if ( (0 >= a_days) && (g_weather->GetRainPeriod(g_date->Date(),3)>0.1))
01588   {
01589     return true;
01590   }
01591   if ( (0 >= a_days) || ((g_weather->GetRainPeriod(g_date->Date(),3)<0.1)
01592           && DoIt(DO_IT_PROB)))
01593   {
01594     a_field->Trace( burn_straw_stubble );
01595     a_field->SetLastTreatment( burn_straw_stubble );
01596     a_field->InsectMortality( 0.4 );
01597     a_field->ReduceVeg( 0.2 );
01598     a_field->SetTramlinesDecay( EL_TRAMLINE_DECAYTIME );
01599     int pref=a_field->GetUnsprayedMarginPolyRef();
01600     if (pref!=-1){
01601       // Must have an unsprayed margin so need to pass the information on to it
01602       LE* um=g_landscape_p->SupplyLEPointer(pref);
01603       um->SetLastTreatment( burn_straw_stubble );
01604       um->ReduceVeg( 0.2 );
01605       um->InsectMortality( 0.4 );
01606       um->SetTramlinesDecay( EL_TRAMLINE_DECAYTIME );
01607     }
01608     return true;
01609   }
01610   return false;
01611 }
```

|  |  |  |  |
| --- | --- | --- | --- |
| bool Farm::CattleIsOut | ( | LE \* | *a\_field*, |
|  |  | double | *a\_user*, |
|  |  | int | *a\_days*, |
|  |  | int | *a\_max* |  |
|  | ) |  |  | `[virtual]` |

Generate a 'cattle\_out' event for every day the cattle are on a\_field.

References cattle\_out, DoIt(), g\_landscape\_p, l\_farm\_cattle\_veg\_reduce(), and UNREFERENCED\_PARAMETER.

Referenced by SetAside::Do().

```
01328 {
01329   UNREFERENCED_PARAMETER( a_user );
01330   a_field->SetLastTreatment( cattle_out );
01331   a_field->Trace( cattle_out );
01332   // Reduce the vegetation because of grazing
01333   double h=a_field->GetVegHeight();
01334   double reduc = 1-(l_farm_cattle_veg_reduce.value()*((h-15)/15));
01335   a_field->ReduceVeg_Extended( reduc );
01336   int pref=a_field->GetUnsprayedMarginPolyRef();
01337   if (pref!=-1){
01338     // Must have an unsprayed margin so need to pass the information on to it
01339     LE* um=g_landscape_p->SupplyLEPointer(pref);
01340     um->SetLastTreatment( cattle_out );
01341     um->ReduceVeg_Extended( reduc );
01342   }
01343   // **cjt** added 24/05/03 to prevent conflict with date checking code
01344   // in the management plans
01345   int d1=g_date->DayInYear(5,9);
01346   if (d1>a_max)
01347     d1=a_max;
01348   if ( ( g_date->DayInYear()> d1 ) &&
01349        ((0 >= a_days)||  DoIt(50/a_days))
01350        ) {
01351     a_field->ToggleCattleGrazing();
01352     int pref=a_field->GetUnsprayedMarginPolyRef();
01353     if (pref!=-1){
01354       // Must have an unsprayed margin so need to pass the information on to it
01355       LE* um=g_landscape_p->SupplyLEPointer(pref);
01356       um->ToggleCattleGrazing();
01357     }
01358     return true;
01359   }
01360   return false;
01361 }
```

|  |  |  |  |
| --- | --- | --- | --- |
| bool Farm::CattleIsOutLow | ( | LE \* | *a\_field*, |
|  |  | double | *a\_user*, |
|  |  | int | *a\_days*, |
|  |  | int | *a\_max* |  |
|  | ) |  |  | `[virtual]` |

Generate a 'cattle\_out\_low' event for every day the cattle are on a\_field.

References cattle\_out\_low, DoIt(), g\_landscape\_p, l\_farm\_cattle\_veg\_reduce2(), and UNREFERENCED\_PARAMETER.

```
01368 {
01369   UNREFERENCED_PARAMETER( a_user );
01370   // Generate a 'cattle_in_out' event for every day the cattle is on the
01371   // field.
01372     a_field->SetLastTreatment( cattle_out_low );
01373     a_field->Trace( cattle_out_low );
01374     // Reduce the vegetation because of grazing
01375     double h=a_field->GetVegHeight();
01376     double reduc = 1-(l_farm_cattle_veg_reduce2.value()*((h-15)/15));
01377     a_field->ReduceVeg_Extended( reduc );
01378     int pref=a_field->GetUnsprayedMarginPolyRef();
01379     if (pref!=-1){
01380       // Must have an unsprayed margin so need to pass the information on to it
01381       LE* um=g_landscape_p->SupplyLEPointer(pref);
01382       um->SetLastTreatment( cattle_out_low );
01383       um->ReduceVeg_Extended( reduc );
01384     }
01385     // **cjt** added 24/05/03 to prevent conflict with date checking code
01386     // in the management plans
01387     int d1=g_date->DayInYear(5,9);
01388     if (d1>a_max) d1=a_max;
01389     if ( ( g_date->DayInYear()>g_date->DayInYear(5,9) )&&
01390            ((0 >= a_days)||  DoIt(50/a_days)))
01391     {
01392       a_field->ToggleCattleGrazing();
01393       int pref=a_field->GetUnsprayedMarginPolyRef();
01394       if (pref!=-1){
01395         // Must have an unsprayed margin so need to pass the information on to it
01396         LE* um=g_landscape_p->SupplyLEPointer(pref);
01397         um->ToggleCattleGrazing();
01398       }
01399       return true;
01400     }
01401   return false;
01402 }
```

|  |  |  |  |
| --- | --- | --- | --- |
| bool Farm::CattleOut | ( | LE \* | *a\_field*, |
|  |  | double | *a\_user*, |
|  |  | int | *a\_days* |  |
|  | ) |  |  | `[virtual]` |

Start a grazing event on a\_field today.

References cattle\_out, DO\_IT\_PROB, DoIt(), g\_landscape\_p, l\_farm\_cattle\_veg\_reduce(), and UNREFERENCED\_PARAMETER.

Referenced by SetAside::Do().

```
01270 {
01271   UNREFERENCED_PARAMETER( a_user );
01272   if ( (0 >= a_days)||  DoIt(DO_IT_PROB)) {
01273           a_field->ToggleCattleGrazing();
01274     a_field->Trace( cattle_out );
01275     a_field->SetLastTreatment( cattle_out );
01276     // Reduce the vegetation because of grazing
01277     double h=a_field->GetVegHeight();
01278     double reduc = 1-(l_farm_cattle_veg_reduce.value()*((h-15)/15));
01279     a_field->ReduceVeg_Extended( reduc );
01280     int pref=a_field->GetUnsprayedMarginPolyRef();
01281     if (pref!=-1){
01282       // Must have an unsprayed margin so need to pass the information on to it
01283          // This happens if all arable fields are given unsprayed margins - they have no effect on grass unless it is sprayed with pesticides
01284       LE* um=g_landscape_p->SupplyLEPointer(pref);
01285       um->ToggleCattleGrazing();
01286       um->SetLastTreatment( cattle_out );
01287       um->ReduceVeg_Extended( reduc );
01288     }
01289     return true;
01290   }
01291   return false;
01292 }
```

|  |  |  |  |
| --- | --- | --- | --- |
| bool Farm::CattleOutLowGrazing | ( | LE \* | *a\_field*, |
|  |  | double | *a\_user*, |
|  |  | int | *a\_days* |  |
|  | ) |  |  | `[virtual]` |

Start a extensive grazing event on a\_field today.

References cattle\_out\_low, DO\_IT\_PROB, DoIt(), g\_landscape\_p, l\_farm\_cattle\_veg\_reduce2(), and UNREFERENCED\_PARAMETER.

```
01299 {
01300   UNREFERENCED_PARAMETER( a_user );
01301   if ( (0 >= a_days)||  DoIt(DO_IT_PROB)) {
01302           a_field->ToggleCattleGrazing();
01303     a_field->Trace( cattle_out_low );
01304     a_field->SetLastTreatment( cattle_out_low );
01305     // Reduce the vegetation because of grazing
01306     double h=a_field->GetVegHeight();
01307     double reduc = 1-(l_farm_cattle_veg_reduce2.value()*((h-15)/15));
01308     a_field->ReduceVeg_Extended( reduc );
01309     int pref=a_field->GetUnsprayedMarginPolyRef();
01310     if (pref!=-1){
01311       // Must have an unsprayed margin so need to pass the information on to it
01312          // This happens if all arable fields are given unsprayed margins - they have no effect on grass unless it is sprayed with pesticides
01313       LE* um=g_landscape_p->SupplyLEPointer(pref);
01314       um->ToggleCattleGrazing();
01315       um->SetLastTreatment( cattle_out_low );
01316       um->ReduceVeg_Extended( reduc );
01317     }
01318     return true;
01319   }
01320   return false;
01321 }
```

|  |  |  |  |  |  |
| --- | --- | --- | --- | --- | --- |
| void Farm::CheckRotationManagementLoop | ( | FarmEvent \* | *ev* | ) | `[protected]` |

Rotation error check function

References m\_farmtype, FarmEvent::m\_field, and m\_rotation.

Referenced by HandleEvents().

```
00452                                                        {
00453   if ( ev->m_field->GetMgtLoopDetectDate() == g_date->Date() ) {
00454     // The last crop managment plan stopped on the same day as
00455     // it was started.
00456 
00457     // Bump loop counter.
00458     ev->m_field->SetMgtLoopDetectCount( ev->m_field->GetMgtLoopDetectCount() + 1 );
00459 
00460     if ( ev->m_field->GetMgtLoopDetectCount() > ( long )( m_rotation.size() + 2 ) ) {
00461       // We have a loop.
00462       char errornum[ 20 ];
00463       sprintf( errornum, "%d", m_farmtype );
00464       g_msg->Warn( WARN_BUG, "Rotation management loop detected in farmtype ", errornum );
00465       exit( 1 );
00466     }
00467   } else {
00468     ev->m_field->SetMgtLoopDetectCount( 0 );
00469   }
00470 }
```

|  |  |  |  |
| --- | --- | --- | --- |
| bool Farm::CutToHay | ( | LE \* | *a\_field*, |
|  |  | double | *a\_user*, |
|  |  | int | *a\_days* |  |
|  | ) |  |  | `[virtual]` |

Carry out hay cutting on a\_field.

References cut\_to\_hay, DO\_IT\_PROB, DoIt(), g\_landscape\_p, and UNREFERENCED\_PARAMETER.

Referenced by SetAside::Do().

```
01618 {
01619   UNREFERENCED_PARAMETER( a_user );
01620   if ( (0 >= a_days) || ((g_weather->GetRainPeriod(g_date->Date(),5)<0.1)
01621           && DoIt(DO_IT_PROB)))
01622   {
01623     a_field->Trace( cut_to_hay );
01624     a_field->SetLastTreatment( cut_to_hay );
01625     a_field->InsectMortality( 0.4 );
01626     a_field->ReduceVeg_Extended( 0.2 );
01627     a_field->SetVegHeight( 10, 0.3, 0.0, 0 );
01628     a_field->SetTramlinesDecay( EL_TRAMLINE_DECAYTIME );
01629     int pref=a_field->GetUnsprayedMarginPolyRef();
01630     if (pref!=-1){
01631       // Must have an unsprayed margin so need to pass the information on to it
01632       LE* um=g_landscape_p->SupplyLEPointer(pref);
01633       um->SetLastTreatment( cut_to_hay );
01634       um->InsectMortality( 0.4 );
01635       um->ReduceVeg_Extended( 0.2 );
01636       um->SetVegHeight( 10, 0.3, 0.0, 0 );
01637       um->SetTramlinesDecay( EL_TRAMLINE_DECAYTIME );
01638     }
01639     return true;
01640   }
01641   return false;
01642 }
```

|  |  |  |  |
| --- | --- | --- | --- |
| bool Farm::CutToSilage | ( | LE \* | *a\_field*, |
|  |  | double | *a\_user*, |
|  |  | int | *a\_days* |  |
|  | ) |  |  | `[virtual]` |

Cut vegetation for silage on a\_field.

References cut\_to\_silage, DO\_IT\_PROB, DoIt(), g\_landscape\_p, and UNREFERENCED\_PARAMETER.

Referenced by SetAside::Do().

```
01675 {
01676     UNREFERENCED_PARAMETER( a_user );
01677     if ( (0 >= a_days) || (!g_weather->Raining() && DoIt(DO_IT_PROB))) {
01678     a_field->Trace( cut_to_silage );
01679     a_field->SetLastTreatment( cut_to_silage );
01680     a_field->ReduceVeg_Extended( 0.2 );
01681     a_field->InsectMortality( 0.4 );
01682     a_field->SetVegHeight( 10, 0.3, 0.0, 0 );
01683     a_field->SetTramlinesDecay( EL_TRAMLINE_DECAYTIME );
01684     int pref=a_field->GetUnsprayedMarginPolyRef();
01685     if (pref!=-1){
01686       // Must have an unsprayed margin so need to pass the information on to it
01687       LE* um=g_landscape_p->SupplyLEPointer(pref);
01688       um->SetLastTreatment( cut_to_silage );
01689       um->ReduceVeg_Extended( 0.2 );
01690       um->InsectMortality( 0.4 );
01691       um->SetVegHeight( 10, 0.3, 0.0, 0 );
01692       um->SetTramlinesDecay( EL_TRAMLINE_DECAYTIME );
01693     }
01694     return true;
01695   }
01696   return false;
01697 }
```

|  |  |  |  |
| --- | --- | --- | --- |
| bool Farm::CutWeeds | ( | LE \* | *a\_field*, |
|  |  | double | *a\_user*, |
|  |  | int | *a\_days* |  |
|  | ) |  |  | `[virtual]` |

Carry out weed topping on a\_field.

References cut\_weeds, DO\_IT\_PROB, DoIt(), g\_landscape\_p, and UNREFERENCED\_PARAMETER.

```
01649 {
01650   UNREFERENCED_PARAMETER( a_user );
01651   if ( (0 >= a_days) || (!g_weather->Raining() && DoIt(DO_IT_PROB)))
01652   {
01653     a_field->Trace( cut_weeds );
01654     a_field->SetLastTreatment( cut_weeds );
01655     a_field->ReduceVeg( 0.8 );
01656     a_field->SetTramlinesDecay( EL_TRAMLINE_DECAYTIME );
01657     int pref=a_field->GetUnsprayedMarginPolyRef();
01658     if (pref!=-1){
01659       // Must have an unsprayed margin so need to pass the information on to it
01660       LE* um=g_landscape_p->SupplyLEPointer(pref);
01661       um->SetLastTreatment( cut_weeds );
01662       um->ReduceVeg( 0.8 );
01663       um->SetTramlinesDecay( EL_TRAMLINE_DECAYTIME );
01664     }
01665     return true;
01666   }
01667   return false;
01668 }
```

|  |  |  |  |
| --- | --- | --- | --- |
| bool Farm::DeepPlough | ( | LE \* | *a\_field*, |
|  |  | double | *a\_user*, |
|  |  | int | *a\_days* |  |
|  | ) |  |  | `[virtual]` |

Carry out a deep ploughing event on a\_field.

References deep\_ploughing, DO\_IT\_PROB, DoIt(), g\_landscape\_p, and UNREFERENCED\_PARAMETER.

Referenced by WinterWheat::Do().

```
00244 {
00245   UNREFERENCED_PARAMETER( a_user );
00246   if ( (0 >= a_days) || (!g_weather->Raining() && DoIt(DO_IT_PROB))) {
00247     a_field->Trace( deep_ploughing );
00248     a_field->SetLastTreatment( deep_ploughing );
00249    // Apply 90% mortality to the insects
00250     a_field->InsectMortality( 0.1 );
00251    // Reduce the vegetation to zero
00252     a_field->ZeroVeg();
00253     int pref=a_field->GetUnsprayedMarginPolyRef();
00254     if (pref!=-1){
00255       // Must have an unsprayed margin so need to pass the information on to it
00256       LE* um=g_landscape_p->SupplyLEPointer(pref);
00257       um->SetLastTreatment( deep_ploughing );
00258       um->InsectMortality( 0.1 );
00259       um->ZeroVeg();
00260     }
00261     return true;
00262   }
00263   return false;
00264 }
```

|  |  |  |  |  |  |
| --- | --- | --- | --- | --- | --- |
| bool Farm::DoIt | ( | int | *a\_probability* | ) |  |

Return chance out of 100.

Referenced by AutumnHarrow(), AutumnPlough(), AutumnRoll(), AutumnSow(), BurnStrawStubble(), CattleIsOut(), CattleIsOutLow(), CattleOut(), CattleOutLowGrazing(), CutToHay(), CutToSilage(), CutWeeds(), DeepPlough(), WinterWheat::Do(), SetAside::Do(), FA\_AmmoniumSulphate(), FA\_GreenManure(), FA\_Manure(), FA\_NPK(), FA\_PK(), FA\_Sludge(), FA\_Slurry(), FP\_GreenManure(), FP\_LiquidNH3(), FP\_ManganeseSulphate(), FP\_Manure(), FP\_NPK(), FP\_NPKS(), FP\_PK(), FP\_Sludge(), FP\_Slurry(), FungicideTreat(), GrowthRegulator(), Harvest(), HayBailing(), HayTurning(), HerbicideTreat(), HillingUp(), InsecticideTreat(), Molluscicide(), PigsAreOut(), PigsOut(), RowCultivation(), SpringHarrow(), SpringPlough(), SpringRoll(), SpringSow(), StrawChopping(), Strigling(), StriglingSow(), StubbleHarrowing(), Swathing(), Water(), and WinterPlough().

```
00444                                    {
00445   return ( a_probability > ( int )( rand() % 100 ) );
00446 }
```

|  |  |  |  |
| --- | --- | --- | --- |
| bool Farm::FA\_AmmoniumSulphate | ( | LE \* | *a\_field*, |
|  |  | double | *a\_user*, |
|  |  | int | *a\_days* |  |
|  | ) |  |  | `[virtual]` |

Apply ammonium supahte to a\_field owned by an stock farmer.

References DO\_IT\_PROB, DoIt(), fa\_ammoniumsulphate, g\_landscape\_p, and UNREFERENCED\_PARAMETER.

```
00669 {
00670   UNREFERENCED_PARAMETER( a_user );
00671   if ( (0 >= a_days) || (!g_weather->Raining() && DoIt(DO_IT_PROB))) {
00672     a_field->Trace( fa_ammoniumsulphate );
00673     a_field->SetLastTreatment( fa_ammoniumsulphate );
00674     a_field->SetTramlinesDecay( EL_TRAMLINE_DECAYTIME );
00675     int pref=a_field->GetUnsprayedMarginPolyRef();
00676     if (pref!=-1){
00677       // Must have an unsprayed margin so need to pass the information on to it
00678       LE* um=g_landscape_p->SupplyLEPointer(pref);
00679       um->SetLastTreatment( fa_ammoniumsulphate );
00680       um->SetTramlinesDecay( EL_TRAMLINE_DECAYTIME );
00681     }
00682     return true;
00683   }
00684   return false;
00685 }
```

|  |  |  |  |
| --- | --- | --- | --- |
| bool Farm::FA\_GreenManure | ( | LE \* | *a\_field*, |
|  |  | double | *a\_user*, |
|  |  | int | *a\_days* |  |
|  | ) |  |  | `[virtual]` |

Spread green manure on a\_field owned by an stock farmer.

References DO\_IT\_PROB, DoIt(), fa\_greenmanure, g\_landscape\_p, and UNREFERENCED\_PARAMETER.

```
00719 {
00720   UNREFERENCED_PARAMETER( a_user );
00721   if ( (0 >= a_days) || (!g_weather->Raining() && DoIt(DO_IT_PROB))) {
00722     a_field->Trace( fa_greenmanure );
00723     a_field->SetLastTreatment( fa_greenmanure );
00724     a_field->SetTramlinesDecay( EL_TRAMLINE_DECAYTIME );
00725     int pref=a_field->GetUnsprayedMarginPolyRef();
00726     if (pref!=-1){
00727       // Must have an unsprayed margin so need to pass the information on to it
00728       LE* um=g_landscape_p->SupplyLEPointer(pref);
00729       um->SetLastTreatment( fa_greenmanure );
00730       um->SetTramlinesDecay( EL_TRAMLINE_DECAYTIME );
00731     }
00732     return true;
00733   }
00734   return false;
00735 }
```

|  |  |  |  |
| --- | --- | --- | --- |
| bool Farm::FA\_Manure | ( | LE \* | *a\_field*, |
|  |  | double | *a\_user*, |
|  |  | int | *a\_days* |  |
|  | ) |  |  | `[virtual]` |

Spread manure on a\_field owned by an stock farmer.

References DO\_IT\_PROB, DoIt(), fa\_manure, g\_landscape\_p, and UNREFERENCED\_PARAMETER.

Referenced by WinterWheat::Do().

```
00693 {
00694   UNREFERENCED_PARAMETER( a_user );
00695   if ( (0 >= a_days) || ((g_weather->GetTemp()>0)&&
00696                                  !g_weather->Raining() && DoIt(DO_IT_PROB)))
00697 {
00698     a_field->Trace( fa_manure );
00699     a_field->SetLastTreatment( fa_manure );
00700     a_field->SetTramlinesDecay( EL_TRAMLINE_DECAYTIME );
00701     int pref=a_field->GetUnsprayedMarginPolyRef();
00702     if (pref!=-1){
00703       // Must have an unsprayed margin so need to pass the information on to it
00704       LE* um=g_landscape_p->SupplyLEPointer(pref);
00705       um->SetLastTreatment( fa_manure );
00706       um->SetTramlinesDecay( EL_TRAMLINE_DECAYTIME );
00707     }
00708     return true;
00709   }
00710   return false;
00711 }
```

|  |  |  |  |
| --- | --- | --- | --- |
| bool Farm::FA\_NPK | ( | LE \* | *a\_field*, |
|  |  | double | *a\_user*, |
|  |  | int | *a\_days* |  |
|  | ) |  |  | `[virtual]` |

Apply NPK fertilizer to a\_field owned by an stock farmer.

References DO\_IT\_PROB, DoIt(), fa\_npk, g\_landscape\_p, and UNREFERENCED\_PARAMETER.

Referenced by WinterWheat::Do().

```
00596 {
00597   UNREFERENCED_PARAMETER( a_user );
00598   if ( (0 >= a_days) || (!g_weather->Raining() && DoIt(DO_IT_PROB))) {
00599     a_field->Trace( fa_npk );
00600     a_field->SetLastTreatment( fa_npk );
00601     a_field->SetTramlinesDecay( EL_TRAMLINE_DECAYTIME );
00602     int pref=a_field->GetUnsprayedMarginPolyRef();
00603     if (pref!=-1){
00604       // Must have an unsprayed margin so need to pass the information on to it
00605       LE* um=g_landscape_p->SupplyLEPointer(pref);
00606       um->SetLastTreatment( fa_npk );
00607       um->SetTramlinesDecay( EL_TRAMLINE_DECAYTIME );
00608     }
00609     return true;
00610   }
00611   return false;
00612 }
```

|  |  |  |  |
| --- | --- | --- | --- |
| bool Farm::FA\_PK | ( | LE \* | *a\_field*, |
|  |  | double | *a\_user*, |
|  |  | int | *a\_days* |  |
|  | ) |  |  | `[virtual]` |

Apply PK fertilizer to a\_field owned by an stock farmer.

References DO\_IT\_PROB, DoIt(), fa\_pk, g\_landscape\_p, and UNREFERENCED\_PARAMETER.

```
00620 {
00621   UNREFERENCED_PARAMETER( a_user );
00622   if ( (0 >= a_days) || (!g_weather->Raining() && DoIt(DO_IT_PROB))) {
00623     a_field->Trace( fa_pk );
00624     a_field->SetLastTreatment( fa_pk );
00625     a_field->SetTramlinesDecay( EL_TRAMLINE_DECAYTIME );
00626     int pref=a_field->GetUnsprayedMarginPolyRef();
00627     if (pref!=-1){
00628       // Must have an unsprayed margin so need to pass the information on to it
00629       LE* um=g_landscape_p->SupplyLEPointer(pref);
00630       um->SetLastTreatment( fa_pk );
00631       um->SetTramlinesDecay( EL_TRAMLINE_DECAYTIME );
00632     }
00633     return true;
00634   }
00635   return false;
00636 }
```

|  |  |  |  |
| --- | --- | --- | --- |
| bool Farm::FA\_Sludge | ( | LE \* | *a\_field*, |
|  |  | double | *a\_user*, |
|  |  | int | *a\_days* |  |
|  | ) |  |  | `[virtual]` |

Spread sewege sludge on a\_field owned by an stock farmer.

References DO\_IT\_PROB, DoIt(), fa\_sludge, g\_landscape\_p, and UNREFERENCED\_PARAMETER.

```
00743 {
00744   UNREFERENCED_PARAMETER( a_user );
00745   if ( (0 >= a_days) || ((g_weather->GetTemp()>0)&&
00746                                  !g_weather->Raining() && DoIt(DO_IT_PROB)))
00747 {
00748     a_field->Trace( fa_sludge );
00749     a_field->SetLastTreatment( fa_sludge );
00750     a_field->SetTramlinesDecay( EL_TRAMLINE_DECAYTIME );
00751     int pref=a_field->GetUnsprayedMarginPolyRef();
00752     if (pref!=-1){
00753       // Must have an unsprayed margin so need to pass the information on to it
00754       LE* um=g_landscape_p->SupplyLEPointer(pref);
00755       um->SetLastTreatment( fa_sludge );
00756       um->SetTramlinesDecay( EL_TRAMLINE_DECAYTIME );
00757     }
00758     return true;
00759   }
00760   return false;
00761 }
```

|  |  |  |  |
| --- | --- | --- | --- |
| bool Farm::FA\_Slurry | ( | LE \* | *a\_field*, |
|  |  | double | *a\_user*, |
|  |  | int | *a\_days* |  |
|  | ) |  |  | `[virtual]` |

Spready slurry on a\_field owned by an stock farmer.

References DO\_IT\_PROB, DoIt(), fa\_slurry, g\_landscape\_p, and UNREFERENCED\_PARAMETER.

Referenced by WinterWheat::Do().

```
00644 {
00645   UNREFERENCED_PARAMETER( a_user );
00646   if ( (0 >= a_days) || ((g_weather->GetTemp()>0)&&
00647                         !g_weather->Raining() && DoIt(DO_IT_PROB)))
00648 {
00649     a_field->Trace( fa_slurry );
00650     a_field->SetLastTreatment( fa_slurry );
00651     a_field->SetTramlinesDecay( EL_TRAMLINE_DECAYTIME );
00652     int pref=a_field->GetUnsprayedMarginPolyRef();
00653     if (pref!=-1){
00654       // Must have an unsprayed margin so need to pass the information on to it
00655       LE* um=g_landscape_p->SupplyLEPointer(pref);
00656       um->SetLastTreatment( fa_slurry );
00657       um->SetTramlinesDecay( EL_TRAMLINE_DECAYTIME );
00658     }
00659     return true;
00660   }
00661   return false;
00662 }
```

|  |  |  |  |
| --- | --- | --- | --- |
| bool Farm::FP\_GreenManure | ( | LE \* | *a\_field*, |
|  |  | double | *a\_user*, |
|  |  | int | *a\_days* |  |
|  | ) |  |  | `[virtual]` |

Spread green manure on a\_field owned by an arable farmer.

References DO\_IT\_PROB, DoIt(), fp\_greenmanure, g\_landscape\_p, and UNREFERENCED\_PARAMETER.

```
00546 {
00547   UNREFERENCED_PARAMETER( a_user );
00548   if ( (0 >= a_days) || (!g_weather->Raining() && DoIt(DO_IT_PROB))) {
00549     a_field->Trace( fp_greenmanure );
00550     a_field->SetLastTreatment( fp_greenmanure );
00551     a_field->SetTramlinesDecay( EL_TRAMLINE_DECAYTIME );
00552     int pref=a_field->GetUnsprayedMarginPolyRef();
00553     if (pref!=-1){
00554       // Must have an unsprayed margin so need to pass the information on to it
00555       LE* um=g_landscape_p->SupplyLEPointer(pref);
00556       um->SetLastTreatment( fp_greenmanure );
00557       um->SetTramlinesDecay( EL_TRAMLINE_DECAYTIME );
00558     }
00559     return true;
00560   }
00561   return false;
00562 }
```

|  |  |  |  |
| --- | --- | --- | --- |
| bool Farm::FP\_LiquidNH3 | ( | LE \* | *a\_field*, |
|  |  | double | *a\_user*, |
|  |  | int | *a\_days* |  |
|  | ) |  |  | `[virtual]` |

Apply liquid ammonia fertilizer to a\_field owned by an arable farmer.

References DO\_IT\_PROB, DoIt(), fp\_liquidNH3, g\_landscape\_p, and UNREFERENCED\_PARAMETER.

```
00447 {
00448   UNREFERENCED_PARAMETER( a_user );
00449   if ( (0 >= a_days) || (!g_weather->Raining() && DoIt(DO_IT_PROB))) {
00450     a_field->Trace( fp_liquidNH3 );
00451     a_field->SetLastTreatment( fp_liquidNH3 );
00452     a_field->SetTramlinesDecay( EL_TRAMLINE_DECAYTIME );
00453     int pref=a_field->GetUnsprayedMarginPolyRef();
00454     if (pref!=-1){
00455       // Must have an unsprayed margin so need to pass the information on to it
00456       LE* um=g_landscape_p->SupplyLEPointer(pref);
00457       um->SetLastTreatment(fp_liquidNH3);
00458       um->SetTramlinesDecay( EL_TRAMLINE_DECAYTIME );
00459     }
00460     return true;
00461   }
00462   return false;
00463 }
```

|  |  |  |  |
| --- | --- | --- | --- |
| bool Farm::FP\_ManganeseSulphate | ( | LE \* | *a\_field*, |
|  |  | double | *a\_user*, |
|  |  | int | *a\_days* |  |
|  | ) |  |  | `[virtual]` |

Apply Manganse Sulphate to a\_field owned by an arable farmer.

References DO\_IT\_PROB, DoIt(), fp\_manganesesulphate, g\_landscape\_p, and UNREFERENCED\_PARAMETER.

Referenced by WinterWheat::Do().

```
00497 {
00498   UNREFERENCED_PARAMETER( a_user );
00499   if ( (0 >= a_days) || (!g_weather->Raining() && DoIt(DO_IT_PROB))) {
00500     a_field->Trace( fp_manganesesulphate );
00501     a_field->SetLastTreatment( fp_manganesesulphate );
00502     a_field->SetTramlinesDecay( EL_TRAMLINE_DECAYTIME );
00503     int pref=a_field->GetUnsprayedMarginPolyRef();
00504     if (pref!=-1){
00505       // Must have an unsprayed margin so need to pass the information on to it
00506       LE* um=g_landscape_p->SupplyLEPointer(pref);
00507       um->SetLastTreatment( fp_manganesesulphate );
00508       um->SetTramlinesDecay( EL_TRAMLINE_DECAYTIME );
00509     }
00510     return true;
00511   }
00512   return false;
00513 }
```

|  |  |  |  |
| --- | --- | --- | --- |
| bool Farm::FP\_Manure | ( | LE \* | *a\_field*, |
|  |  | double | *a\_user*, |
|  |  | int | *a\_days* |  |
|  | ) |  |  | `[virtual]` |

Spread manure on a\_field owned by an arable farmer.

References DO\_IT\_PROB, DoIt(), fp\_manure, g\_landscape\_p, and UNREFERENCED\_PARAMETER.

```
00521 {
00522   UNREFERENCED_PARAMETER( a_user );
00523   if ( (0 >= a_days) || ((g_weather->GetTemp()>0)&&
00524                         !g_weather->Raining() && DoIt(DO_IT_PROB))) {
00525     a_field->Trace( fp_manure );
00526     a_field->SetLastTreatment( fp_manure );
00527     a_field->SetTramlinesDecay( EL_TRAMLINE_DECAYTIME );
00528     int pref=a_field->GetUnsprayedMarginPolyRef();
00529     if (pref!=-1){
00530       // Must have an unsprayed margin so need to pass the information on to it
00531       LE* um=g_landscape_p->SupplyLEPointer(pref);
00532       um->SetLastTreatment( fp_manure );
00533       um->SetTramlinesDecay( EL_TRAMLINE_DECAYTIME );
00534     }
00535     return true;
00536   }
00537   return false;
00538 }
```

|  |  |  |  |
| --- | --- | --- | --- |
| bool Farm::FP\_NPK | ( | LE \* | *a\_field*, |
|  |  | double | *a\_user*, |
|  |  | int | *a\_days* |  |
|  | ) |  |  | `[virtual]` |

Apply NPK fertilizer, on a\_field owned by an arable farmer.

References DO\_IT\_PROB, DoIt(), fp\_npk, g\_landscape\_p, and UNREFERENCED\_PARAMETER.

Referenced by WinterWheat::Do().

```
00399 {
00400   UNREFERENCED_PARAMETER( a_user );
00401   if ( (0 >= a_days) || (!g_weather->Raining() && DoIt(DO_IT_PROB))) {
00402     a_field->Trace( fp_npk );
00403     a_field->SetLastTreatment( fp_npk );
00404     a_field->SetTramlinesDecay( EL_TRAMLINE_DECAYTIME );
00405     int pref=a_field->GetUnsprayedMarginPolyRef();
00406     if (pref!=-1){
00407       // Must have an unsprayed margin so need to pass the information on to it
00408       LE* um=g_landscape_p->SupplyLEPointer(pref);
00409       um->SetLastTreatment(fp_npk);
00410       um->SetTramlinesDecay( EL_TRAMLINE_DECAYTIME );
00411     }
00412     return true;
00413   }
00414   return false;
00415 }
```

|  |  |  |  |
| --- | --- | --- | --- |
| bool Farm::FP\_NPKS | ( | LE \* | *a\_field*, |
|  |  | double | *a\_user*, |
|  |  | int | *a\_days* |  |
|  | ) |  |  | `[virtual]` |

Apply NPKS fertilizer, on a\_field owned by an arable farmer.

References DO\_IT\_PROB, DoIt(), fp\_npks, g\_landscape\_p, sleep\_all\_day, and UNREFERENCED\_PARAMETER.

```
00372 {
00373   UNREFERENCED_PARAMETER( a_user );
00374   if ( (0 >= a_days) || (!g_weather->Raining() && DoIt(DO_IT_PROB))) {
00375     a_field->Trace( fp_npks );
00376     a_field->SetLastTreatment( fp_npks );
00377     a_field->SetTramlinesDecay( EL_TRAMLINE_DECAYTIME );
00378     int pref=a_field->GetUnsprayedMarginPolyRef();
00379 if (pref!=-1){
00380   // Must have an unsprayed margin so need to pass the information on to it
00381   LE* um=g_landscape_p->SupplyLEPointer(pref);
00382   um->SetLastTreatment(sleep_all_day);
00383   um->SetGrowthPhase( harvest1 );
00384   um->InsectMortality( 0.4 );
00385   um->SetTramlinesDecay( EL_TRAMLINE_DECAYTIME );
00386 }
00387 
00388     return true;
00389   }
00390   return false;
00391 }
```

|  |  |  |  |
| --- | --- | --- | --- |
| bool Farm::FP\_PK | ( | LE \* | *a\_field*, |
|  |  | double | *a\_user*, |
|  |  | int | *a\_days* |  |
|  | ) |  |  | `[virtual]` |

Apply PK fertilizer, on a\_field owned by an arable farmer.

References DO\_IT\_PROB, DoIt(), fp\_pk, g\_landscape\_p, and UNREFERENCED\_PARAMETER.

```
00423 {
00424   UNREFERENCED_PARAMETER( a_user );
00425   if ( (0 >= a_days) || (!g_weather->Raining() && DoIt(DO_IT_PROB))) {
00426     a_field->Trace( fp_pk );
00427     a_field->SetLastTreatment( fp_pk );
00428     a_field->SetTramlinesDecay( EL_TRAMLINE_DECAYTIME );
00429     int pref=a_field->GetUnsprayedMarginPolyRef();
00430     if (pref!=-1){
00431       // Must have an unsprayed margin so need to pass the information on to it
00432       LE* um=g_landscape_p->SupplyLEPointer(pref);
00433       um->SetLastTreatment(fp_pk);
00434       um->SetTramlinesDecay( EL_TRAMLINE_DECAYTIME );
00435     }
00436     return true;
00437   }
00438   return false;
00439 }
```

|  |  |  |  |
| --- | --- | --- | --- |
| bool Farm::FP\_Sludge | ( | LE \* | *a\_field*, |
|  |  | double | *a\_user*, |
|  |  | int | *a\_days* |  |
|  | ) |  |  | `[virtual]` |

Spread sewege on a\_field owned by an arable farmer.

References DO\_IT\_PROB, DoIt(), fp\_sludge, g\_landscape\_p, and UNREFERENCED\_PARAMETER.

```
00570 {
00571   UNREFERENCED_PARAMETER( a_user );
00572   if ( (0 >= a_days) || ((g_weather->GetTemp()>0)&&
00573                                  !g_weather->Raining() && DoIt(DO_IT_PROB)))
00574 {
00575     a_field->Trace( fp_sludge );
00576     a_field->SetLastTreatment( fp_sludge );
00577     a_field->SetTramlinesDecay( EL_TRAMLINE_DECAYTIME );
00578     int pref=a_field->GetUnsprayedMarginPolyRef();
00579     if (pref!=-1){
00580       // Must have an unsprayed margin so need to pass the information on to it
00581       LE* um=g_landscape_p->SupplyLEPointer(pref);
00582       um->SetLastTreatment( fp_sludge );
00583       um->SetTramlinesDecay( EL_TRAMLINE_DECAYTIME );
00584     }
00585     return true;
00586   }
00587   return false;
00588 }
```

|  |  |  |  |
| --- | --- | --- | --- |
| bool Farm::FP\_Slurry | ( | LE \* | *a\_field*, |
|  |  | double | *a\_user*, |
|  |  | int | *a\_days* |  |
|  | ) |  |  | `[virtual]` |

Apply slurry to a\_field owned by an arable farmer.

References DO\_IT\_PROB, DoIt(), fp\_slurry, g\_landscape\_p, and UNREFERENCED\_PARAMETER.

Referenced by WinterWheat::Do().

```
00471 {
00472   UNREFERENCED_PARAMETER( a_user );
00473   if ( (0 >= a_days) || ((g_weather->GetTemp()>0)&&
00474                                  !g_weather->Raining() && DoIt(DO_IT_PROB)))
00475 {
00476     a_field->Trace( fp_slurry );
00477     a_field->SetLastTreatment( fp_slurry );
00478     a_field->SetTramlinesDecay( EL_TRAMLINE_DECAYTIME );
00479     int pref=a_field->GetUnsprayedMarginPolyRef();
00480     if (pref!=-1){
00481       // Must have an unsprayed margin so need to pass the information on to it
00482       LE* um=g_landscape_p->SupplyLEPointer(pref);
00483       um->SetLastTreatment(fp_slurry);
00484       um->SetTramlinesDecay( EL_TRAMLINE_DECAYTIME );
00485     }
00486     return true;
00487   }
00488   return false;
00489 }
```

|  |  |  |  |
| --- | --- | --- | --- |
| bool Farm::FungicideTreat | ( | LE \* | *a\_field*, |
|  |  | double | *a\_user*, |
|  |  | int | *a\_days* |  |
|  | ) |  |  | `[virtual]` |

Apply fungicide to a\_field.

References DO\_IT\_PROB, DoIt(), fungicide\_treat, and UNREFERENCED\_PARAMETER.

Referenced by WinterWheat::Do().

```
00834 {
00835   UNREFERENCED_PARAMETER( a_user );
00836   if (0 >= a_days)
00837   {
00838     if ( (!g_weather->Raining()) && (g_weather->GetWind()<4.5) &&
00839         ( ! a_field->GetSignal() & LE_SIG_NO_FUNGICIDE )) {
00840       a_field->Trace( fungicide_treat );
00841       a_field->SetLastTreatment( fungicide_treat );
00842       a_field->SetTramlinesDecay( EL_TRAMLINE_DECAYTIME );
00843     }
00844     return true;
00845   }
00846   else if ( (g_weather->GetWind()<4.5) &&
00847             (!g_weather->Raining()) && DoIt(DO_IT_PROB)) {
00848     if ( ! (a_field->GetSignal() & LE_SIG_NO_FUNGICIDE) ) {
00849       a_field->Trace( fungicide_treat );
00850       a_field->SetLastTreatment( fungicide_treat );
00851       a_field->SetTramlinesDecay( EL_TRAMLINE_DECAYTIME );
00852     }
00853     return true;
00854   }
00855   return false;
00856 }
```

|  |  |  |  |  |  |
| --- | --- | --- | --- | --- | --- |
| int Farm::GetFarmNumber | ( | void |  | ) | `[inline]` |

References m\_farm\_num.

```
00442 { return m_farm_num; }
```

|  |  |  |  |  |  |
| --- | --- | --- | --- | --- | --- |
| int Farm::GetFirstCropIndex | ( | TTypesOfLandscapeElement | *a\_type* | ) | `[protected, virtual]` |

Gets the first crop for the farm.

This method also synchronises farm rotations either within or between farms if needed. This is useful to try simple what if scenarios.

References m\_rotation, and m\_rotation\_sync\_index.

Referenced by InitiateManagement().

```
00724                                                                    {
00725   // If g_farm_fixed_rotation, then determine the first
00726   // crop number in the rotation rotation number.
00727   if ( g_farm_fixed_rotation_enable.value() ) {
00728 
00729     if ( !g_farm_fixed_rotation_farms_async.value() ) {
00730       // We are running all the farms synchronized, so
00731       // simply set the first crop to run on all farm fields.
00732       return 0;
00733     }
00734 
00735     // Each farm runs its fields sync'ed but independently from
00736     // the other farmers.
00737 
00738     // Determine if this farm has selected its own start index
00739     // and set it if not. m_rotation_sync_index is initialized
00740     // to -1 by the Farm::Farm() constructor.
00741     if ( -1 == m_rotation_sync_index ) {
00742       m_rotation_sync_index = (int) (rand() % m_rotation.size());
00743     }
00744     // Return farm localized rotation index.
00745     return m_rotation_sync_index;
00746   }
00747 
00748   // Not synchronised, but we want to follow our rotation sequence, so check
00749   // if we have started this process, if not set the sync value.
00750   // afterwards just increment this.
00751   if ( -1 == m_rotation_sync_index ) {
00752       m_rotation_sync_index = (int) (rand() % m_rotation.size());
00753   }
00754   else m_rotation_sync_index = (int) ((m_rotation_sync_index+1) % m_rotation.size());
00755   return m_rotation_sync_index;
00756 }
```

|  |  |  |  |  |  |
| --- | --- | --- | --- | --- | --- |
| int Farm::GetFirstDate | ( | TTypesOfVegetation | *a\_tov* | ) | `[protected]` |

Gets the start date for a crop type.

References Crop::GetFirstDate(), m\_agrochemindustrycereal, m\_carrots, m\_CGG1, m\_CGG2, m\_fieldpeas, m\_fodderbeet, m\_maize, m\_oats, m\_OBarleyPCG, m\_ocarrots, m\_OCGG1, m\_OCGG2, m\_OCGS1, m\_ofieldpeas, m\_ofirstyeardanger, m\_ograzingpigs, m\_ooats, m\_opermgrassgrazed, m\_opotatoes, m\_OSBarleysilage, m\_ospringbarley, m\_ospringbarleypigs, m\_owinterbarley, m\_owinterrape, m\_owinterrye, m\_owinterwheatundersown, m\_permanentsetaside, m\_permgrassgrazed, m\_permgrasslowgrazed, m\_potatoes, m\_sbarleyclovergrass, m\_seedgrass1, m\_seedgrass2, m\_setaside, m\_springbarley, m\_springbarleyseed, m\_springbarleysilage, m\_springbarleystrigling, m\_springbarleystriglingculm, m\_springbarleystriglingsingle, m\_triticale, m\_winterbarley, m\_winterrape, m\_winterrye, m\_winterwheat, m\_winterwheatstrigling, m\_winterwheatstriglingculm, m\_winterwheatstriglingsingle, m\_wwheatpcontrol, m\_wwheatptoxiccontrol, m\_wwheatptreatment, m\_youngforest, tov\_AgroChemIndustryCereal, tov\_Carrots, tov\_CloverGrassGrazed1, tov\_CloverGrassGrazed2, tov\_FieldPeas, tov\_FodderBeet, tov\_Maize, tov\_Oats, tov\_OBarleyPeaCloverGrass, tov\_OCarrots, tov\_OCloverGrassGrazed1, tov\_OCloverGrassGrazed2, tov\_OCloverGrassSilage1, tov\_OFieldPeas, tov\_OFirstYearDanger, tov\_OGrazingPigs, tov\_OOats, tov\_OPermanentGrassGrazed, tov\_OPotatoes, tov\_OSBarleySilage, tov\_OSpringBarley, tov\_OSpringBarleyPigs, tov\_OWinterBarley, tov\_OWinterRape, tov\_OWinterRye, tov\_OWinterWheatUndersown, tov\_PermanentGrassGrazed, tov\_PermanentGrassLowGrazed, tov\_PermanentSetaside, tov\_Potatoes, tov\_SeedGrass1, tov\_SeedGrass2, tov\_Setaside, tov\_SpringBarley, tov\_SpringBarleyCloverGrass, tov\_SpringBarleySeed, tov\_SpringBarleySilage, tov\_SpringBarleyStrigling, tov\_SpringBarleyStriglingCulm, tov\_SpringBarleyStriglingSingle, tov\_Triticale, tov\_WinterBarley, tov\_WinterRape, tov\_WinterRye, tov\_WinterWheat, tov\_WinterWheatStrigling, tov\_WinterWheatStriglingCulm, tov\_WinterWheatStriglingSingle, tov\_WWheatPControl, tov\_WWheatPToxicControl, tov\_WWheatPTreatment, and tov\_YoungForest.

Referenced by GetNextCropStartDate().

```
00869                                                   {
00870   switch ( a_tov2 ) {
00871     case tov_Carrots:
00872       return m_carrots->GetFirstDate();
00873     case tov_CloverGrassGrazed1:
00874       return m_CGG1->GetFirstDate();
00875     case tov_CloverGrassGrazed2:
00876       return m_CGG2->GetFirstDate();
00877     case tov_FieldPeas:
00878       return m_fieldpeas->GetFirstDate();
00879     case tov_FodderBeet:
00880       return m_fodderbeet->GetFirstDate();
00881     case tov_Maize:
00882       return m_maize->GetFirstDate();
00883     case tov_OBarleyPeaCloverGrass:
00884       return m_OBarleyPCG->GetFirstDate();
00885     case tov_OSBarleySilage:
00886       return m_OSBarleysilage->GetFirstDate();
00887     case tov_OCarrots:
00888       return m_ocarrots->GetFirstDate();
00889     case tov_OCloverGrassGrazed1:
00890       return m_OCGG1->GetFirstDate();
00891     case tov_OCloverGrassGrazed2:
00892       return m_OCGG2->GetFirstDate();
00893     case tov_OCloverGrassSilage1:
00894       return m_OCGS1->GetFirstDate();
00895     case tov_OFieldPeas:
00896       return m_ofieldpeas->GetFirstDate();
00897     case tov_OFirstYearDanger:
00898       return m_ofirstyeardanger->GetFirstDate();
00899     case tov_OGrazingPigs:
00900       return m_ograzingpigs->GetFirstDate();
00901     case tov_OOats:
00902       return m_ooats->GetFirstDate();
00903     case tov_Oats:
00904       return m_oats->GetFirstDate();
00905     case tov_OPermanentGrassGrazed:
00906       return m_opermgrassgrazed->GetFirstDate();
00907     case tov_OPotatoes:
00908       return m_opotatoes->GetFirstDate();
00909     case tov_OSpringBarley:
00910       return m_ospringbarley->GetFirstDate();
00911     case tov_OSpringBarleyPigs:
00912       return m_ospringbarleypigs->GetFirstDate();
00913     case tov_OWinterBarley:
00914       return m_owinterbarley->GetFirstDate();
00915     case tov_OWinterRape:
00916       return m_owinterrape->GetFirstDate();
00917     case tov_OWinterRye:
00918       return m_owinterrye->GetFirstDate();
00919     case tov_OWinterWheatUndersown:
00920       return m_owinterwheatundersown->GetFirstDate();
00921     case tov_PermanentGrassGrazed:
00922       return m_permgrassgrazed->GetFirstDate();
00923     case tov_PermanentGrassLowGrazed:
00924       return m_permgrasslowgrazed->GetFirstDate();
00925     case tov_PermanentSetaside:
00926       return m_permanentsetaside->GetFirstDate();
00927     case tov_Potatoes:
00928       return m_potatoes->GetFirstDate();
00929     case tov_SeedGrass1:
00930       return m_seedgrass1->GetFirstDate();
00931     case tov_SeedGrass2:
00932       return m_seedgrass2->GetFirstDate();
00933     case tov_Setaside:
00934       return m_setaside->GetFirstDate();
00935     case tov_SpringBarley:
00936       return m_springbarley->GetFirstDate();
00937     case tov_SpringBarleyCloverGrass:
00938       return m_sbarleyclovergrass->GetFirstDate();
00939     case tov_SpringBarleySeed:
00940       return m_springbarleyseed->GetFirstDate();
00941     case tov_SpringBarleySilage:
00942       return m_springbarleysilage->GetFirstDate();
00943     case tov_SpringBarleyStrigling:
00944       return m_springbarleystrigling->GetFirstDate();
00945     case tov_SpringBarleyStriglingSingle:
00946       return m_springbarleystriglingsingle->GetFirstDate();
00947     case tov_SpringBarleyStriglingCulm:
00948       return m_springbarleystriglingculm->GetFirstDate();
00949     case tov_Triticale:
00950       return m_triticale->GetFirstDate();
00951     case tov_WinterBarley:
00952       return m_winterbarley->GetFirstDate();
00953     case tov_WinterRape:
00954       return m_winterrape->GetFirstDate();
00955     case tov_WinterRye:
00956       return m_winterrye->GetFirstDate();
00957     case tov_WinterWheat:
00958       return m_winterwheat->GetFirstDate();
00959     case tov_WinterWheatStrigling:
00960       return m_winterwheatstrigling->GetFirstDate();
00961     case tov_WinterWheatStriglingSingle:
00962       return m_winterwheatstriglingsingle->GetFirstDate();
00963     case tov_WinterWheatStriglingCulm:
00964       return m_winterwheatstriglingculm->GetFirstDate();
00965     case tov_WWheatPControl:
00966       return m_wwheatpcontrol->GetFirstDate();
00967     case tov_WWheatPToxicControl:
00968       return m_wwheatptoxiccontrol->GetFirstDate();
00969     case tov_WWheatPTreatment:
00970       return m_wwheatptreatment->GetFirstDate();
00971     case tov_AgroChemIndustryCereal:
00972       return m_agrochemindustrycereal->GetFirstDate();
00973     case tov_YoungForest:
00974       return m_youngforest->GetFirstDate();
00975     default:
00976       return 0;
00977   }
00978 }
```

|  |  |  |  |  |  |
| --- | --- | --- | --- | --- | --- |
| bool Farm::GetIntensity | ( | void |  | ) | `[inline]` |

References m\_intensity.

```
00518 { return m_intensity; }
```

|  |  |  |  |  |  |
| --- | --- | --- | --- | --- | --- |
| int Farm::GetNextCropIndex | ( | int | *a\_rot\_index* | ) | `[protected, virtual]` |

Returns the next crop in the rotation.

Also provides the possibility of over-riding rotations using configuration settings

References m\_rotation.

Referenced by GetNextCropStartDate(), and HandleEvents().

```
00766                                             {
00767   if ( !g_farm_enable_crop_rotation.value() ) {
00768     // Rotation not enabled.
00769     return a_rot_index;
00770   }
00771 
00772   if ( a_rot_index == -1 )
00773     return -1;
00774 
00775   if ( ( unsigned int ) ( ++a_rot_index ) == m_rotation.size() )
00776     a_rot_index = 0;
00777 
00778   return a_rot_index;
00779 }
```

|  |  |  |  |
| --- | --- | --- | --- |
| int Farm::GetNextCropStartDate | ( | LE \* | *a\_field*, |
|  |  | TTypesOfVegetation & | *a\_curr\_veg* |  |
|  | ) |  |  | `[protected]` |

Returns the start date of the next crop in the rotation.

References GetFirstDate(), GetNextCropIndex(), and m\_rotation.

Referenced by HandleEvents(), and InitiateManagement().

```
00477                                                                               {
00478   TTypesOfVegetation l_tov2;
00479 
00480   if ( a_field->GetRotIndex() < 0 || g_farm_fixed_crop_enable.value() //|| g_farm_test_crop.value() 
00481                                                                           ) {
00482     l_tov2 = a_curr_veg; // don't do it if no rotation
00483   } else {
00484     l_tov2 = m_rotation[ GetNextCropIndex( a_field->GetRotIndex() ) ];
00485   }
00486   a_curr_veg = l_tov2;
00487   return GetFirstDate( l_tov2 );
00488 }
```

|  |  |  |  |  |  |
| --- | --- | --- | --- | --- | --- |
| TTypesOfFarm Farm::GetType | ( | void |  | ) | `[inline]` |

References m\_farmtype.

```
00515 { return m_farmtype; }
```

|  |  |  |  |
| --- | --- | --- | --- |
| bool Farm::Glyphosate | ( | LE \* | *a\_field*, |
|  |  | double | *a\_user*, |
|  |  | int | *a\_days* |  |
|  | ) |  |  | `[virtual]` |

References glyphosate, and UNREFERENCED\_PARAMETER.

Referenced by SetAside::Do().

```
01747 {
01748   UNREFERENCED_PARAMETER( a_user );
01749   UNREFERENCED_PARAMETER( a_days );
01750   // Will always do this at the first chance
01751   a_field->Trace( glyphosate );
01752   a_field->SetLastTreatment( glyphosate );
01753   a_field->InsectMortality( 0.5 );
01754   a_field->ReduceVeg_Extended( 0.05 );
01755   return true;
01756 }
```

|  |  |  |  |
| --- | --- | --- | --- |
| bool Farm::GrowthRegulator | ( | LE \* | *a\_field*, |
|  |  | double | *a\_user*, |
|  |  | int | *a\_days* |  |
|  | ) |  |  | `[virtual]` |

Apply growth regulator to a\_field.

References DO\_IT\_PROB, DoIt(), growth\_regulator, and UNREFERENCED\_PARAMETER.

Referenced by WinterWheat::Do().

```
00805 {
00806   UNREFERENCED_PARAMETER( a_user );
00807   if (0 >= a_days)
00808   {
00809     if ( (!g_weather->Raining()) && (g_weather->GetWind()<4.5) &&
00810         (!(a_field->GetSignal() & LE_SIG_NO_GROWTH_REG) )) {
00811       a_field->Trace( growth_regulator );
00812       a_field->SetLastTreatment( growth_regulator );
00813       a_field->SetTramlinesDecay( EL_TRAMLINE_DECAYTIME );
00814     }
00815     return true;
00816   }
00817   else if ( (g_weather->GetWind()<4.5) &&
00818            (!g_weather->Raining()) && DoIt(DO_IT_PROB)) {
00819     if ( ! (a_field->GetSignal() & LE_SIG_NO_GROWTH_REG) ) {
00820       a_field->Trace( growth_regulator );
00821       a_field->SetLastTreatment( growth_regulator );
00822       a_field->SetTramlinesDecay( EL_TRAMLINE_DECAYTIME );
00823     }
00824     return true;
00825   }
00826   return false;
00827 }
```

|  |  |  |  |  |  |
| --- | --- | --- | --- | --- | --- |
| void Farm::HandleEvents | ( | void |  | ) | `[protected]` |

If there are events to carry out do this, and perhaps start a new crop.

References AddNewEvent(), CheckRotationManagementLoop(), GetNextCropIndex(), GetNextCropStartDate(), LeSwitch(), FarmEvent::m\_field, m\_queue, m\_rotation, PROG\_START, and tov\_Undefined.

Referenced by Management().

```
00495                               {
00496   if ( m_queue.Empty() )
00497     return;
00498 
00499   LowPriPair < FarmEvent * > pair = m_queue.Bottom();
00500   FarmEvent * ev = pair.m_element;
00501   while ( pair.m_pri <= g_date->Date() ) {
00502     m_queue.Pop();
00503 
00504         if ( LeSwitch( ev ) ) {
00505       // This crop management plan has terminated.
00506 
00507       // First check for an infinite loop in the rotation scheme,
00508       // ie. a scenario where all crops decide not to run given
00509       // the date.
00510       CheckRotationManagementLoop( ev );
00511 
00512       // Outdate any remaining events for this field.
00513       ev->m_field->BumpRunNum();
00514 
00515       // Crop treatment done, select and initiate new crop if in rotation.
00516       TTypesOfVegetation new_veg = ev->m_field->GetVegType();
00517 
00518       if ( ev->m_field->GetRotIndex() >= 0 ) {
00519         int new_index = GetNextCropIndex( ev->m_field->GetRotIndex() );
00520         new_veg = m_rotation[ new_index ];
00521         // Running in fixed crop mode?
00522         if ( g_farm_fixed_crop_enable.value() ) {
00523           new_veg = g_letype->TranslateVegTypes( g_farm_fixed_crop_type.value() );
00524         }
00525         /*
00526                 if ( g_farm_test_crop.value() ) {
00527           new_veg = g_letype->TranslateVegTypes( g_farm_test_crop_type.value() );
00528         }
00529                 */
00530         ev->m_field->SetRotIndex( new_index );
00531         ev->m_field->SetVegType( new_veg, tov_Undefined );
00532         ev->m_field->ForceGrowthTest();
00533       }
00534 
00535       // Reset the event list for this field.
00536       ev->m_field->ResetTrace();
00537       // Reset event timeout counter.
00538       ev->m_field->SetVegStore( 0 );
00539 
00540       // The next bit simply determines the start date of the next crop in
00541       // the rotation and passes this to the start crop event.
00542       // The crop is responsible for raising an error if the next crop is
00543       // not possible or otherwise handling the problem
00544 
00545       // 19/5-2003: Note: This code was moved out into a dedicated
00546       // method of the Farm class, GetNextCropStartDate(), as precisely
00547       // the same piece of code needs to be run during initialization of
00548       // farm management.
00549       TTypesOfVegetation l_tov = new_veg;
00550       int l_nextcropstartdate = GetNextCropStartDate( ev->m_field, l_tov );
00551 
00552       // Create 'start' event for today and put it on the queue.
00553       AddNewEvent( new_veg, g_date->Date(), ev->m_field, PROG_START, ev->m_field->GetRunNum(),
00554            false, l_nextcropstartdate, false, l_tov );
00555 
00556       // Set starting date for rotation mgmt loop detection.
00557       ev->m_field->SetMgtLoopDetectDate( g_date->Date() );
00558     }
00559 
00560     delete ev;
00561 
00562     if ( m_queue.Empty() )
00563       return;
00564     pair = m_queue.Bottom();
00565     ev = pair.m_element;
00566   }
00567 }
```

|  |  |  |  |
| --- | --- | --- | --- |
| bool Farm::Harvest | ( | LE \* | *a\_field*, |
|  |  | double | *a\_user*, |
|  |  | int | *a\_days* |  |
|  | ) |  |  | `[virtual]` |

Carry out a harvest on a\_field.

References DO\_IT\_PROB, DoIt(), g\_landscape\_p, harvest, and UNREFERENCED\_PARAMETER.

Referenced by WinterWheat::Do().

```
01236 {
01237   UNREFERENCED_PARAMETER( a_user );
01238   //5 days good weather before
01239   if ( (0 >= a_days) ||
01240        ((g_weather->GetRainPeriod(g_date->Date(),5)<0.1) && DoIt(DO_IT_PROB))
01241   ) {
01242     a_field->Trace( harvest );
01243     a_field->SetLastTreatment( harvest );
01244     a_field->SetGrowthPhase( harvest1 );
01245     // Here we have to do a little skip to avoid too low insect populations after harvest, but a correct veg biomass
01246     a_field->InsectMortality( 0.4 );
01247     double insects=a_field->GetInsectPop();
01248     a_field->RecalculateBugsNStuff();
01249     a_field->SetInsectPop(insects);
01250     a_field->SetTramlinesDecay( EL_TRAMLINE_DECAYTIME );
01251     int pref=a_field->GetUnsprayedMarginPolyRef();
01252     if (pref!=-1){
01253       // Must have an unsprayed margin so need to pass the information on to it
01254       LE* um=g_landscape_p->SupplyLEPointer(pref);
01255       um->SetLastTreatment(harvest);
01256       um->SetGrowthPhase( harvest1 );
01257       um->InsectMortality( 0.4 );
01258       um->SetTramlinesDecay( EL_TRAMLINE_DECAYTIME );
01259     }
01260     return true;
01261   }
01262   return false;
01263 }
```

|  |  |  |  |
| --- | --- | --- | --- |
| bool Farm::HayBailing | ( | LE \* | *a\_field*, |
|  |  | double | *a\_user*, |
|  |  | int | *a\_days* |  |
|  | ) |  |  | `[virtual]` |

Carry out hay bailing on a\_field.

References DO\_IT\_PROB, DoIt(), g\_landscape\_p, hay\_bailing, and UNREFERENCED\_PARAMETER.

Referenced by WinterWheat::Do().

```
01533 {
01534   UNREFERENCED_PARAMETER( a_user );
01535   if ( (0 >= a_days) ||
01536        ((g_weather->GetRainPeriod(g_date->Date(),5)<0.1) && DoIt(DO_IT_PROB))
01537   ) {
01538     a_field->Trace( hay_bailing );
01539     a_field->SetLastTreatment( hay_bailing );
01540     a_field->SetTramlinesDecay( EL_TRAMLINE_DECAYTIME );
01541     int pref=a_field->GetUnsprayedMarginPolyRef();
01542     if (pref!=-1){
01543       // Must have an unsprayed margin so need to pass the information on to it
01544       LE* um=g_landscape_p->SupplyLEPointer(pref);
01545       um->SetLastTreatment( hay_bailing );
01546       um->SetTramlinesDecay( EL_TRAMLINE_DECAYTIME );
01547     }
01548     return true;
01549   }
01550   return false;
01551 }
```

|  |  |  |  |
| --- | --- | --- | --- |
| bool Farm::HayTurning | ( | LE \* | *a\_field*, |
|  |  | double | *a\_user*, |
|  |  | int | *a\_days* |  |
|  | ) |  |  | `[virtual]` |

Carry out hay turning on a\_field.

References DO\_IT\_PROB, DoIt(), g\_landscape\_p, hay\_turning, and UNREFERENCED\_PARAMETER.

Referenced by WinterWheat::Do().

```
01508 {
01509   UNREFERENCED_PARAMETER( a_user );
01510   if ( (0 >= a_days) ||
01511        ((g_weather->GetRainPeriod(g_date->Date(),5)<0.1) && DoIt(DO_IT_PROB))
01512   ) {
01513     a_field->Trace( hay_turning );
01514     a_field->SetLastTreatment( hay_turning );
01515     a_field->SetTramlinesDecay( EL_TRAMLINE_DECAYTIME );
01516     int pref=a_field->GetUnsprayedMarginPolyRef();
01517     if (pref!=-1){
01518       // Must have an unsprayed margin so need to pass the information on to it
01519       LE* um=g_landscape_p->SupplyLEPointer(pref);
01520       um->SetLastTreatment( hay_turning );
01521       um->SetTramlinesDecay( EL_TRAMLINE_DECAYTIME );
01522     }
01523     return true;
01524   }
01525   return false;
01526 }
```

|  |  |  |  |
| --- | --- | --- | --- |
| bool Farm::HerbicideTreat | ( | LE \* | *a\_field*, |
|  |  | double | *a\_user*, |
|  |  | int | *a\_days* |  |
|  | ) |  |  | `[virtual]` |

Apply herbicide to a\_field.

References DO\_IT\_PROB, DoIt(), herbicide\_treat, l\_farm\_herbicide\_kills(), and UNREFERENCED\_PARAMETER.

Referenced by WinterWheat::Do().

```
00769 {
00770   UNREFERENCED_PARAMETER( a_user );
00771   if (0 >= a_days) {
00772     if ( (!g_weather->Raining() && (g_weather->GetWind()<4.5)) && ((a_field->GetSignal() & LE_SIG_NO_HERBICIDE)==0 )) {
00773       a_field->Trace( herbicide_treat );
00774       a_field->SetLastTreatment( herbicide_treat );
00775       if ( l_farm_herbicide_kills.value()) {
00776         a_field->ReduceWeedBiomass( 0.05 );
00777       }
00778       a_field->SetTramlinesDecay( EL_TRAMLINE_DECAYTIME );
00779       a_field->SetHerbicideDelay( EL_HERBICIDE_DELAYTIME );
00780     }
00781     a_field->SetTramlinesDecay( EL_TRAMLINE_DECAYTIME );
00782     return true;
00783   }
00784   else if ((g_weather->GetWind()<4.5) &&
00785            (!g_weather->Raining()) && DoIt(DO_IT_PROB)) {
00786     if ( !(a_field->GetSignal() & LE_SIG_NO_HERBICIDE) ) {
00787       a_field->Trace( herbicide_treat );
00788       a_field->SetLastTreatment( herbicide_treat );
00789       if ( l_farm_herbicide_kills.value()) {
00790         a_field->ReduceWeedBiomass( 0.05 );
00791       }
00792       a_field->SetTramlinesDecay( EL_TRAMLINE_DECAYTIME );
00793       a_field->SetHerbicideDelay( EL_HERBICIDE_DELAYTIME );
00794     }
00795   return true;
00796   }
00797   return false;
00798 }
```

|  |  |  |  |
| --- | --- | --- | --- |
| bool Farm::HillingUp | ( | LE \* | *a\_field*, |
|  |  | double | *a\_user*, |
|  |  | int | *a\_days* |  |
|  | ) |  |  | `[virtual]` |

Do hilling up on a\_field, probably of potatoes.

References DO\_IT\_PROB, DoIt(), g\_landscape\_p, hilling\_up, and UNREFERENCED\_PARAMETER.

```
01157 {
01158   UNREFERENCED_PARAMETER( a_user );
01159   if ( (0 >= a_days) || (!g_weather->Raining() && DoIt(DO_IT_PROB))) {
01160     a_field->Trace( hilling_up );
01161     a_field->SetLastTreatment( hilling_up );
01162     a_field->InsectMortality( 0.75 );
01163     a_field->ReduceWeedBiomass( 0.25 );
01164     a_field->SetTramlinesDecay( EL_TRAMLINE_DECAYTIME );
01165     int pref=a_field->GetUnsprayedMarginPolyRef();
01166     if (pref!=-1){
01167       // Must have an unsprayed margin so need to pass the information on to it
01168       LE* um=g_landscape_p->SupplyLEPointer(pref);
01169       um->SetLastTreatment( hilling_up );
01170       um->ReduceWeedBiomass( 0.25 );
01171       um->InsectMortality( 0.75 );
01172       um->SetTramlinesDecay( EL_TRAMLINE_DECAYTIME );
01173     }
01174     return true;
01175   }
01176   return false;
01177 }
```

|  |  |  |  |  |  |
| --- | --- | --- | --- | --- | --- |
| void Farm::InitiateManagement | ( | void |  | ) | `[virtual]` |

Kicks of the farm's management.

References AddNewEvent(), GetFirstCropIndex(), GetNextCropStartDate(), m\_fields, m\_rotation, PROG\_START, tole\_PermanentSetaside, tole\_PermPasture, tole\_PermPastureLowGrazing, tole\_YoungForest, tov\_PermanentGrassGrazed, tov\_PermanentGrassLowGrazed, tov\_PermanentSetaside, tov\_Undefined, and tov\_YoungForest.

```
00816                                     {
00817   for ( unsigned int i = 0; i < m_fields.size(); i++ ) {
00818     int rot_index = -1;
00819     TTypesOfVegetation new_veg = tov_PermanentGrassGrazed;
00820 
00821     // Check for either type of permanent pasture
00822     // field (with management plan).
00823     if ( m_fields[ i ]->GetElementType() == tole_PermanentSetaside ) {
00824       // Vegetation type is already set correctly by the constructor
00825       // for this element type. Start management plan and skip to next field.
00826       // Rotation is *not* possible on a field of type tole_PermanentSetaside!
00827       new_veg = tov_PermanentSetaside;
00828 
00829     } else if ( m_fields[ i ]->GetElementType() == tole_PermPastureLowGrazing ) {
00830       new_veg = tov_PermanentGrassLowGrazed;
00831     } else if ( m_fields[ i ]->GetElementType() == tole_YoungForest ) {
00832       new_veg = tov_YoungForest;
00833     } else if ( m_fields[ i ]->GetElementType() != tole_PermPasture ) {
00834       rot_index = GetFirstCropIndex( m_fields[ i ]->GetElementType() );
00835       new_veg = m_rotation[ rot_index ];
00836     }
00837 
00838     long prog_start_date = g_date->Date();
00839 
00840     // Running in fixed crop mode?
00841     if ( g_farm_fixed_crop_enable.value() ) {
00842       int fv = g_farm_fixed_crop_type.value();
00843       new_veg = g_letype->TranslateVegTypes( fv );
00844     }
00845     /*
00846         if ( g_farm_test_crop.value() ) {
00847       prog_start_date = 0;
00848       new_veg = g_letype->TranslateVegTypes( g_farm_test_crop_type.value() );
00849     }
00850         */
00851     m_fields[ i ]->SetVegType( new_veg, tov_Undefined );
00852     m_fields[ i ]->SetRotIndex( rot_index );
00853     m_fields[ i ]->SetGrowthPhase( janfirst );
00854 
00855     // Reset event timeout counter. We are now 800 days from
00856     // oblivion.
00857     m_fields[ i ]->SetVegStore( 0 );
00858     TTypesOfVegetation l_tov = new_veg;
00859     int l_nextcropstartdate = GetNextCropStartDate( m_fields[ i ], l_tov );
00860 
00861     AddNewEvent( new_veg, prog_start_date, m_fields[ i ], PROG_START, 0, false, l_nextcropstartdate, true, l_tov );
00862   }
00863 }
```

|  |  |  |  |
| --- | --- | --- | --- |
| bool Farm::InsecticideTreat | ( | LE \* | *a\_field*, |
|  |  | double | *a\_user*, |
|  |  | int | *a\_days* |  |
|  | ) |  |  | `[virtual]` |

Apply insecticide to a\_field.

References DO\_IT\_PROB, DoIt(), insecticide\_treat, l\_farm\_insecticide\_kills(), and UNREFERENCED\_PARAMETER.

Referenced by WinterWheat::Do().

```
00863 {
00864   UNREFERENCED_PARAMETER( a_user );
00865   if (0 >= a_days) {
00866   if ( (!g_weather->Raining()) && (g_weather->GetWind()<4.5) && ( ! (a_field->GetSignal() & LE_SIG_NO_INSECTICIDE) )) {
00867 // **CJT** Turn this code on to use the pesticide engine with insecticides
00868 //      g_pest->DailyQueueAdd( a_field, l_pest_insecticide_amount.value());
00869 //
00870       a_field->Trace( insecticide_treat );
00871       a_field->SetLastTreatment( insecticide_treat );
00872       if ( l_farm_insecticide_kills.value()) {
00873        a_field->Insecticide( 0.36 );
00874       }
00875       a_field->SetTramlinesDecay( EL_TRAMLINE_DECAYTIME );
00876     }
00877     return true;
00878   }
00879   else if ( (g_weather->GetWind()<4.5) &&
00880             (!g_weather->Raining()) && DoIt(DO_IT_PROB)) {
00881     if ( ! (a_field->GetSignal() & LE_SIG_NO_INSECTICIDE )) {
00882 // **CJT** Turn this code on to use the pesticide engine with insecticides
00883 //      g_pest->DailyQueueAdd( a_field, l_pest_insecticide_amount.value());
00884 //
00885       a_field->Trace( insecticide_treat );
00886       a_field->SetLastTreatment( insecticide_treat );
00887       if ( l_farm_insecticide_kills.value()) {
00888        a_field->Insecticide( 0.36 );
00889       }
00890       a_field->SetTramlinesDecay( EL_TRAMLINE_DECAYTIME );
00891     }
00892     return true;
00893   }
00894   return false;
00895 }
```

|  |  |  |  |  |  |
| --- | --- | --- | --- | --- | --- |
| bool Farm::IsStockFarmer | ( | void |  | ) | `[inline]` |

References m\_stockfarmer.

Referenced by WinterWheat::Do(), and SetAside::Do().

```
00516 { return m_stockfarmer; }
```

|  |  |  |  |  |  |
| --- | --- | --- | --- | --- | --- |
| bool Farm::LeSwitch | ( | FarmEvent \* | *ev* | ) | `[protected]` |

Call do function for any crop with an outstanding event. Signal if the crop has terminated.

References WinterWheat::Do(), SetAside::Do(), m\_agrochemindustrycereal, m\_carrots, m\_CGG1, m\_CGG2, FarmEvent::m\_event, FarmEvent::m\_field, m\_fieldpeas, m\_fieldpeasstrigling, m\_fodderbeet, m\_maize, m\_maizestrigling, m\_oats, m\_OBarleyPCG, m\_ocarrots, m\_OCGG1, m\_OCGG2, m\_OCGS1, m\_ofieldpeas, m\_ofieldpeassilage, m\_ograzingpigs, m\_ooats, m\_opermgrassgrazed, m\_opotatoes, m\_ospringbarley, m\_ospringbarleypigs, m\_owinterbarley, m\_owinterrape, m\_owinterrye, m\_owinterwheatundersown, m\_permanentsetaside, m\_permgrassgrazed, m\_permgrasslowgrazed, m\_potatoes, FarmEvent::m\_run, m\_sbarleyclovergrass, m\_seedgrass1, m\_seedgrass2, m\_setaside, m\_springbarley, m\_springbarleyclovergrassstrigling, m\_springbarleypeaclovergrassstrigling, m\_springbarleyseed, m\_springbarleysilage, m\_springbarleystrigling, m\_springbarleystriglingculm, m\_springbarleystriglingsingle, FarmEvent::m\_todo, m\_triticale, m\_winterbarley, m\_winterbarleystrigling, m\_winterrape, m\_winterrapestrigling, m\_winterrye, m\_winterryestrigling, m\_winterwheat, m\_winterwheatstrigling, m\_winterwheatstriglingculm, m\_winterwheatstriglingsingle, m\_wwheatpcontrol, m\_wwheatptoxiccontrol, m\_wwheatptreatment, m\_youngforest, tov\_AgroChemIndustryCereal, tov\_Carrots, tov\_CloverGrassGrazed1, tov\_CloverGrassGrazed2, tov\_FieldPeas, tov\_FieldPeasStrigling, tov\_FodderBeet, tov\_Maize, tov\_MaizeStrigling, tov\_Oats, tov\_OBarleyPeaCloverGrass, tov\_OCarrots, tov\_OCloverGrassGrazed1, tov\_OCloverGrassGrazed2, tov\_OCloverGrassSilage1, tov\_OFieldPeas, tov\_OFieldPeasSilage, tov\_OGrazingPigs, tov\_OOats, tov\_OPermanentGrassGrazed, tov\_OPotatoes, tov\_OSpringBarley, tov\_OSpringBarleyPigs, tov\_OWinterBarley, tov\_OWinterRape, tov\_OWinterRye, tov\_OWinterWheatUndersown, tov\_PermanentGrassGrazed, tov\_PermanentGrassLowGrazed, tov\_PermanentSetaside, tov\_Potatoes, tov\_SeedGrass1, tov\_SeedGrass2, tov\_Setaside, tov\_SpringBarley, tov\_SpringBarleyCloverGrass, tov\_SpringBarleyCloverGrassStrigling, tov\_SpringBarleyPeaCloverGrassStrigling, tov\_SpringBarleySeed, tov\_SpringBarleySilage, tov\_SpringBarleyStrigling, tov\_SpringBarleyStriglingCulm, tov\_SpringBarleyStriglingSingle, tov\_Triticale, tov\_WinterBarley, tov\_WinterBarleyStrigling, tov\_WinterRape, tov\_WinterRapeStrigling, tov\_WinterRye, tov\_WinterRyeStrigling, tov\_WinterWheat, tov\_WinterWheatStrigling, tov\_WinterWheatStriglingCulm, tov\_WinterWheatStriglingSingle, tov\_WWheatPControl, tov\_WWheatPToxicControl, tov\_WWheatPTreatment, and tov\_YoungForest.

Referenced by HandleEvents().

```
00231                                     {
00232   // Ignore this event if it is from the execution of
00233   // a previous management plan.
00234   if ( ev->m_field->GetRunNum() > ev->m_run )
00235     return false;
00236 
00237   // Store what we are trying to do this time.
00238   // ***FN*** To be cleaned up later.
00239   ev->m_field->m_tried_to_do = ev->m_todo;
00240 
00241   bool done;
00242 
00243   switch ( ev->m_event ) {
00244     case tov_Carrots:
00245       done = m_carrots->Do( this, ev->m_field, ev );
00246     break;
00247     case tov_CloverGrassGrazed1:
00248       done = m_CGG1->Do( this, ev->m_field, ev );
00249     break;
00250     case tov_CloverGrassGrazed2:
00251       done = m_CGG2->Do( this, ev->m_field, ev );
00252     break;
00253     case tov_FieldPeas:
00254       done = m_fieldpeas->Do( this, ev->m_field, ev );
00255     break;
00256     case tov_FodderBeet:
00257       done = m_fodderbeet->Do( this, ev->m_field, ev );
00258     break;
00259     case tov_Maize:
00260       done = m_maize->Do( this, ev->m_field, ev );
00261     break;
00262     case tov_OBarleyPeaCloverGrass:
00263       done = m_OBarleyPCG->Do( this, ev->m_field, ev );
00264     break;
00265     case tov_OCarrots:
00266       done = m_ocarrots->Do( this, ev->m_field, ev );
00267     break;
00268     case tov_OCloverGrassSilage1:
00269       done = m_OCGS1->Do( this, ev->m_field, ev );
00270     break;
00271     case tov_OCloverGrassGrazed1:
00272       done = m_OCGG1->Do( this, ev->m_field, ev );
00273     break;
00274     case tov_OCloverGrassGrazed2:
00275       done = m_OCGG2->Do( this, ev->m_field, ev );
00276     break;
00277     case tov_OFieldPeas:
00278       done = m_ofieldpeas->Do( this, ev->m_field, ev );
00279     break;
00280     case tov_OFieldPeasSilage:
00281       done = m_ofieldpeassilage->Do( this, ev->m_field, ev );
00282     break;
00283     case tov_OGrazingPigs:
00284       done = m_ograzingpigs->Do( this, ev->m_field, ev );
00285     break;
00286     case tov_OOats:
00287       done = m_ooats->Do( this, ev->m_field, ev );
00288     break;
00289     case tov_Oats:
00290       done = m_oats->Do( this, ev->m_field, ev );
00291     break;
00292     case tov_OPermanentGrassGrazed:
00293       done = m_opermgrassgrazed->Do( this, ev->m_field, ev );
00294     break;
00295     case tov_OPotatoes:
00296       done = m_opotatoes->Do( this, ev->m_field, ev );
00297     break;
00298     case tov_OSpringBarley:
00299       done = m_ospringbarley->Do( this, ev->m_field, ev );
00300     break;
00301     case tov_OSpringBarleyPigs:
00302       done = m_ospringbarleypigs->Do( this, ev->m_field, ev );
00303     break;
00304     case tov_OWinterBarley:
00305       done = m_owinterbarley->Do( this, ev->m_field, ev );
00306     break;
00307     case tov_OWinterWheatUndersown:
00308       done = m_owinterwheatundersown->Do( this, ev->m_field, ev );
00309     break;
00310     case tov_OWinterRape:
00311       done = m_owinterrape->Do( this, ev->m_field, ev );
00312     break;
00313     case tov_OWinterRye:
00314       done = m_owinterrye->Do( this, ev->m_field, ev );
00315     break;
00316     case tov_PermanentGrassGrazed:
00317       done = m_permgrassgrazed->Do( this, ev->m_field, ev );
00318     break;
00319     case tov_PermanentGrassLowGrazed:
00320       done = m_permgrasslowgrazed->Do( this, ev->m_field, ev );
00321     break;
00322     case tov_PermanentSetaside:
00323       done = m_permanentsetaside->Do( this, ev->m_field, ev );
00324     break;
00325     case tov_Potatoes:
00326       done = m_potatoes->Do( this, ev->m_field, ev );
00327     break;
00328     case tov_SeedGrass1:
00329       done = m_seedgrass1->Do( this, ev->m_field, ev );
00330     break;
00331     case tov_SeedGrass2:
00332       done = m_seedgrass2->Do( this, ev->m_field, ev );
00333     break;
00334     case tov_Setaside:
00335       done = m_setaside->Do( this, ev->m_field, ev );
00336     break;
00337     case tov_SpringBarley:
00338       done = m_springbarley->Do( this, ev->m_field, ev );
00339     break;
00340     case tov_SpringBarleyCloverGrass:
00341       done = m_sbarleyclovergrass->Do( this, ev->m_field, ev );
00342     break;
00343     case tov_SpringBarleySeed:
00344       done = m_springbarleyseed->Do( this, ev->m_field, ev );
00345     break;
00346     case tov_SpringBarleySilage:
00347       done = m_springbarleysilage->Do( this, ev->m_field, ev );
00348     break;
00349     case tov_Triticale:
00350       done = m_triticale->Do( this, ev->m_field, ev );
00351     break;
00352     case tov_WinterBarley:
00353       done = m_winterbarley->Do( this, ev->m_field, ev );
00354     break;
00355     case tov_WinterRape:
00356       done = m_winterrape->Do( this, ev->m_field, ev );
00357     break;
00358     case tov_WinterRye:
00359       done = m_winterrye->Do( this, ev->m_field, ev );
00360     break;
00361     case tov_WinterWheat:
00362       done = m_winterwheat->Do( this, ev->m_field, ev );
00363     break;
00364     case tov_WWheatPControl:
00365       done = m_wwheatpcontrol->Do( this, ev->m_field, ev );
00366     break;
00367     case tov_WWheatPToxicControl:
00368       done = m_wwheatptoxiccontrol->Do( this, ev->m_field, ev );
00369     break;
00370     case tov_WWheatPTreatment:
00371       done = m_wwheatptreatment->Do( this, ev->m_field, ev );
00372     break;
00373     case tov_AgroChemIndustryCereal:
00374       done = m_agrochemindustrycereal->Do( this, ev->m_field, ev );
00375     break;
00376     case tov_WinterWheatStrigling:
00377       done = m_winterwheatstrigling->Do( this, ev->m_field, ev );
00378       break;
00379     case tov_WinterWheatStriglingSingle:
00380       done = m_winterwheatstriglingsingle->Do( this, ev->m_field, ev );
00381       break;
00382     case tov_WinterWheatStriglingCulm:
00383       done = m_winterwheatstriglingculm->Do( this, ev->m_field, ev );
00384       break;
00385     case tov_SpringBarleyCloverGrassStrigling:
00386       done = m_springbarleyclovergrassstrigling->Do( this, ev->m_field, ev );
00387     break;
00388     case tov_SpringBarleyStrigling:
00389       done = m_springbarleystrigling->Do( this, ev->m_field, ev );
00390     break;
00391     case tov_SpringBarleyStriglingSingle:
00392       done = m_springbarleystriglingsingle->Do( this, ev->m_field, ev );
00393     break;
00394     case tov_SpringBarleyStriglingCulm:
00395       done = m_springbarleystriglingculm->Do( this, ev->m_field, ev );
00396     break;
00397     case tov_MaizeStrigling:
00398       done = m_maizestrigling->Do( this, ev->m_field, ev );
00399     break;
00400     case tov_WinterRapeStrigling:
00401       done = m_winterrapestrigling->Do( this, ev->m_field, ev );
00402     break;
00403     case tov_WinterRyeStrigling:
00404       done = m_winterryestrigling->Do( this, ev->m_field, ev );
00405     break;
00406     case tov_WinterBarleyStrigling:
00407       done = m_winterbarleystrigling->Do( this, ev->m_field, ev );
00408     break;
00409     case tov_FieldPeasStrigling:
00410       done = m_fieldpeasstrigling->Do( this, ev->m_field, ev );
00411     break;
00412     case tov_SpringBarleyPeaCloverGrassStrigling:
00413       done = m_springbarleypeaclovergrassstrigling->Do( this, ev->m_field, ev );
00414     break;
00415     case tov_YoungForest:
00416       done = m_youngforest->Do( this, ev->m_field, ev );
00417     break;
00418 
00419       /* case tov_OFirstYearDanger: done = m_ofirstyeardanger->Do( this, ev->m_field, ev ); break; */
00420     default:
00421       g_msg->Warn( WARN_BUG, "Farm::LeSwitch(): ""Unknown crop type! ", "" );
00422       exit( 1 );
00423   }
00424   return done;
00425 }
```

|  |  |  |  |  |  |
| --- | --- | --- | --- | --- | --- |
| virtual void Farm::MakeStockFarmer | ( | void |  | ) | `[inline, virtual]` |

Reimplemented in ConventionalPlant, OrganicPlant, PesticideTrialControl, PesticideTrialToxicControl, and PesticideTrialTreatment.

References m\_stockfarmer.

```
00517 { m_stockfarmer = true; }
```

|  |  |  |  |  |  |
| --- | --- | --- | --- | --- | --- |
| void Farm::Management | ( | void |  | ) | `[virtual]` |

Starts the main management loop for the farm and performs some error checking.

References HandleEvents(), and m\_fields.

```
00206                             {
00207   HandleEvents();
00208   for ( unsigned int i = 0; i < m_fields.size(); i++ ) {
00209     // Check for infinite loop in management plan.
00210     int count = m_fields[ i ]->GetVegStore();
00211     if ( count >= 0 )
00212       m_fields[ i ]->SetVegStore( ++count );
00213     if ( count > 800 ) {
00214       // More than two years where nothing happened.
00215       // Raise 'Merry Christmas'!
00216       char error_num[ 20 ];
00217       sprintf( error_num, "%d", m_fields[ i ]->GetVegType() );
00218       g_msg->Warn( WARN_BUG, "I the Farm Manager caught infinite loop in tov type:", error_num );
00219       sprintf( error_num, "%d", m_fields[ i ]->m_tried_to_do );
00220       g_msg->Warn( WARN_BUG, "It was last seen trying to perform action # ""(or thereabouts):", error_num );
00221       exit( 1 );
00222     }
00223   }
00224 }
```

|  |  |  |  |
| --- | --- | --- | --- |
| bool Farm::Molluscicide | ( | LE \* | *a\_field*, |
|  |  | double | *a\_user*, |
|  |  | int | *a\_days* |  |
|  | ) |  |  | `[virtual]` |

Apply molluscidie to a\_field.

References DO\_IT\_PROB, DoIt(), molluscicide, and UNREFERENCED\_PARAMETER.

```
01006 {
01007     UNREFERENCED_PARAMETER( a_user );
01008        if (0 >= a_days) {
01009     if ( (!g_weather->Raining()) && (g_weather->GetWind()<4.5) &&
01010         (! (a_field->GetSignal() & LE_SIG_NO_MOLLUSC ))) {
01011       a_field->Trace( molluscicide );
01012       a_field->SetLastTreatment( molluscicide );
01013       a_field->SetTramlinesDecay( EL_TRAMLINE_DECAYTIME );
01014     }
01015     return true;
01016   }
01017   else if ( (0 >= a_days) || (!g_weather->Raining() && DoIt(DO_IT_PROB))) {
01018     if ( ! (a_field->GetSignal() & LE_SIG_NO_MOLLUSC) ) {
01019       a_field->Trace( molluscicide );
01020       a_field->SetLastTreatment( molluscicide );
01021       a_field->SetTramlinesDecay( EL_TRAMLINE_DECAYTIME );
01022     }
01023     return true;
01024   }
01025   return false;
01026 }
```

|  |  |  |  |
| --- | --- | --- | --- |
| bool Farm::PigsAreOut | ( | LE \* | *a\_field*, |
|  |  | double | *a\_user*, |
|  |  | int | *a\_days* |  |
|  | ) |  |  | `[virtual]` |

Start a pig grazing event on a\_field today or soon.

References DoIt(), g\_landscape\_p, and UNREFERENCED\_PARAMETER.

```
01461 {
01462   UNREFERENCED_PARAMETER( a_user );
01463   if ( (0 >= a_days)||  DoIt(50/a_days)) {
01464     a_field->TogglePigGrazing();
01465     int pref=a_field->GetUnsprayedMarginPolyRef();
01466     if (pref!=-1){
01467       // Must have an unsprayed margin so need to pass the information on to it
01468       LE* um=g_landscape_p->SupplyLEPointer(pref);
01469       um->TogglePigGrazing();
01470     }
01471     return true;
01472   }
01473   return false;
01474 }
```

|  |  |  |  |
| --- | --- | --- | --- |
| bool Farm::PigsAreOutForced | ( | LE \* | *a\_field*, |
|  |  | double | *a\_user*, |
|  |  | int | *a\_days* |  |
|  | ) |  |  | `[virtual]` |

Start a pig grazing event on a\_field today - no exceptions.

References g\_landscape\_p, l\_farm\_pig\_veg\_reduce(), pigs\_out, and UNREFERENCED\_PARAMETER.

```
01437 {
01438   UNREFERENCED_PARAMETER( a_days );
01439   UNREFERENCED_PARAMETER( a_user );
01440   a_field->SetLastTreatment( pigs_out );
01441   a_field->Trace( pigs_out );
01442   // Reduce the vegetation because of grazing
01443   a_field->ReduceVeg_Extended( l_farm_pig_veg_reduce.value() );
01444   // make this a function of grazing pressure
01445   //and field size - perhaps in a later life
01446   int pref=a_field->GetUnsprayedMarginPolyRef();
01447   if (pref!=-1){
01448     // Must have an unsprayed margin so need to pass the information on to it
01449     LE* um=g_landscape_p->SupplyLEPointer(pref);
01450     um->SetLastTreatment( pigs_out );
01451     um->ReduceVeg_Extended( l_farm_pig_veg_reduce.value() );
01452   }
01453   return false;
01454 }
```

|  |  |  |  |
| --- | --- | --- | --- |
| bool Farm::PigsOut | ( | LE \* | *a\_field*, |
|  |  | double | *a\_user*, |
|  |  | int | *a\_days* |  |
|  | ) |  |  | `[virtual]` |

Generate a 'pigs\_out' event for every day the cattle are on a\_field.

References DO\_IT\_PROB, DoIt(), g\_landscape\_p, l\_farm\_pig\_veg\_reduce(), pigs\_out, and UNREFERENCED\_PARAMETER.

```
01409 {
01410   UNREFERENCED_PARAMETER( a_user );
01411   if ( (0 >= a_days)||  DoIt(DO_IT_PROB))
01412  {
01413     a_field->TogglePigGrazing();
01414     a_field->Trace( pigs_out );
01415     a_field->SetLastTreatment( pigs_out );
01416     // Reduce the vegetation because of grazing
01417     a_field->ReduceVeg_Extended( l_farm_pig_veg_reduce.value());
01418                                  // make this a function of grazing pressure
01419                                  //and field size - perhaps in a later life
01420     int pref=a_field->GetUnsprayedMarginPolyRef();
01421     if (pref!=-1){
01422       // Must have an unsprayed margin so need to pass the information on to it
01423       LE* um=g_landscape_p->SupplyLEPointer(pref);
01424       um->SetLastTreatment( pigs_out );
01425       um->ReduceVeg_Extended( l_farm_pig_veg_reduce.value() );
01426     }
01427     return true;
01428  }
01429   return false;
01430 }
```

|  |  |  |  |
| --- | --- | --- | --- |
| bool Farm::ProductApplication0 | ( | LE \* | *a\_field*, |
|  |  | double | *a\_user*, |
|  |  | int | *a\_days* |  |
|  | ) |  |  | `[virtual]` |

Apply test pesticide to a\_field.

References l\_farm\_insecticide\_kills(), l\_pest\_product\_0\_amount, product\_treat, and UNREFERENCED\_PARAMETER.

```
00940 {
00941   UNREFERENCED_PARAMETER( a_user );
00942   // NOTE Differs from normal pesticide in that it will be done on the last
00943   // day if not managed before
00944   if (0 >= a_days) {
00945     a_field->Trace( product_treat );
00946     a_field->SetLastTreatment( product_treat );
00947     if ( l_farm_insecticide_kills.value()) {        
00948         a_field->Insecticide( 0.2 );     }
00949     a_field->SetTramlinesDecay( EL_TRAMLINE_DECAYTIME );
00950     double p=l_pest_product_0_amount.value();
00951     g_pest->DailyQueueAdd( a_field,p );
00952     return true;
00953   } else {
00954     if ( (!g_weather->Raining()) && (g_weather->GetWind()<4.5)) {
00955     a_field->Trace( product_treat );
00956     a_field->SetLastTreatment( product_treat );
00957     if ( l_farm_insecticide_kills.value()) {        
00958          a_field->Insecticide( 0.2 );     }
00959     a_field->SetTramlinesDecay( EL_TRAMLINE_DECAYTIME );
00960     double p=l_pest_product_0_amount.value();
00961     g_pest->DailyQueueAdd( a_field,p );
00962       return true;
00963          }
00964   }
00965   return false;
00966 }
```

|  |  |  |  |
| --- | --- | --- | --- |
| bool Farm::ProductApplication1 | ( | LE \* | *a\_field*, |
|  |  | double | *a\_user*, |
|  |  | int | *a\_days* |  |
|  | ) |  |  | `[virtual]` |

Apply test pesticide to a\_field.

References l\_farm\_insecticide\_kills(), l\_pest\_product\_1\_amount, product\_treat, and UNREFERENCED\_PARAMETER.

```
00973 {
00974   UNREFERENCED_PARAMETER( a_user );
00975   // NOTE Differs from normal pesticide in that it will be done on the last
00976   // day if not managed before
00977   if (0 >= a_days) {
00978     a_field->Trace( product_treat );
00979     a_field->SetLastTreatment( product_treat );
00980     if ( l_farm_insecticide_kills.value()) {        
00981         a_field->Insecticide( 0.2 );     }
00982     a_field->SetTramlinesDecay( EL_TRAMLINE_DECAYTIME );
00983     double p=l_pest_product_1_amount.value();
00984     g_pest->DailyQueueAdd( a_field,p );
00985     return true;
00986   } else {
00987     if ( (!g_weather->Raining()) && (g_weather->GetWind()<4.5)) {
00988     a_field->Trace( product_treat );
00989     a_field->SetLastTreatment( product_treat );
00990     if ( l_farm_insecticide_kills.value()) {        
00991          a_field->Insecticide( 0.2 );     }
00992     a_field->SetTramlinesDecay( EL_TRAMLINE_DECAYTIME );
00993     double p=l_pest_product_1_amount.value();
00994     g_pest->DailyQueueAdd( a_field,p );
00995       return true;
00996          }
00997   }
00998   return false;
00999 }
```

|  |  |  |  |  |  |
| --- | --- | --- | --- | --- | --- |
| void Farm::RemoveField | ( | LE \* | *a\_field* | ) |  |

Removes a field to a farm.

References m\_fields.

```
00797                                      {
00798   int nf = (int) m_fields.size();
00799   for ( int i = 0; i < nf; i++ ) {
00800     if ( m_fields[ i ] == a_field ) {
00801       m_fields.erase( m_fields.begin() + i );
00802       return;
00803     }
00804   }
00805   // If we reach here there is something wrong because the field is not a
00806   // member of this farm
00807   g_msg->Warn( WARN_BUG, "Farm::RemoveField(LE* a_field): ""Unknown field! ", "" );
00808   exit( 1 );
00809 }
```

|  |  |  |  |
| --- | --- | --- | --- |
| bool Farm::RowCultivation | ( | LE \* | *a\_field*, |
|  |  | double | *a\_user*, |
|  |  | int | *a\_days* |  |
|  | ) |  |  | `[virtual]` |

Carry out a harrowing between crop rows on a\_field.

References DO\_IT\_PROB, DoIt(), g\_landscape\_p, row\_cultivation, and UNREFERENCED\_PARAMETER.

```
01033 {
01034   UNREFERENCED_PARAMETER( a_user );
01035   if ( (0 >= a_days) && (g_weather->GetRainPeriod(g_date->Date(),3)<0.1) )
01036   {
01037     // Too much rain, just give up and claim we did it.
01038     return true;
01039   }
01040 
01041   if ( (0 >= a_days) ||
01042        ((g_weather->GetRainPeriod(g_date->Date(),3)<0.1) && DoIt(DO_IT_PROB))
01043   ) {
01044     a_field->Trace( row_cultivation );
01045     a_field->SetLastTreatment( row_cultivation );
01046     a_field->ReduceWeedBiomass( 0.5 );
01047     a_field->InsectMortality( 0.25 );
01048     a_field->SetTramlinesDecay( EL_TRAMLINE_DECAYTIME );
01049     int pref=a_field->GetUnsprayedMarginPolyRef();
01050     if (pref!=-1){
01051       // Must have an unsprayed margin so need to pass the information on to it
01052       LE* um=g_landscape_p->SupplyLEPointer(pref);
01053       um->SetLastTreatment( row_cultivation );
01054       um->InsectMortality( 0.25 );
01055       um->ReduceWeedBiomass( 0.5 );
01056       um->SetTramlinesDecay( EL_TRAMLINE_DECAYTIME );
01057     }
01058     return true;
01059   }
01060   return false;
01061 }
```

|  |  |  |  |  |  |
| --- | --- | --- | --- | --- | --- |
| void Farm::SetFarmNumber | ( | int | *a\_farm\_num* | ) | `[inline]` |

References m\_farm\_num.

```
00441 { m_farm_num = a_farm_num; }
```

|  |  |  |  |
| --- | --- | --- | --- |
| bool Farm::SleepAllDay | ( | LE \* | *a\_field*, |
|  |  | double | *a\_user*, |
|  |  | int | *a\_days* |  |
|  | ) |  |  | `[virtual]` |

Nothing to to today on a\_field.

References g\_landscape\_p, sleep\_all\_day, and UNREFERENCED\_PARAMETER.

```
00124 {
00125     UNREFERENCED_PARAMETER( a_user );
00126     UNREFERENCED_PARAMETER( a_days );
00127   a_field->Trace( sleep_all_day );
00128   a_field->SetLastTreatment( sleep_all_day );
00129   int pref=a_field->GetUnsprayedMarginPolyRef();
00130   if (pref!=-1){
00131     // Must have an unsprayed margin so need to pass the information on to it
00132     LE* um=g_landscape_p->SupplyLEPointer(pref);
00133     um->SetLastTreatment(sleep_all_day);
00134   }
00135   return true;
00136 }
```

|  |  |  |  |
| --- | --- | --- | --- |
| bool Farm::SpringHarrow | ( | LE \* | *a\_field*, |
|  |  | double | *a\_user*, |
|  |  | int | *a\_days* |  |
|  | ) |  |  | `[virtual]` |

Carry out a harrow event in the spring on a\_field.

References DO\_IT\_PROB, DoIt(), g\_landscape\_p, spring\_harrow, and UNREFERENCED\_PARAMETER.

```
00297 {
00298   UNREFERENCED_PARAMETER( a_user );
00299   if ( (0 >= a_days) || (!g_weather->Raining() && DoIt(DO_IT_PROB))) {
00300     a_field->Trace( spring_harrow );
00301     a_field->SetLastTreatment( spring_harrow );
00302     // 30% insect mortality
00303        a_field->InsectMortality( 0.7 );
00304     // remove all vegetation
00305        a_field->ZeroVeg();
00306     int pref=a_field->GetUnsprayedMarginPolyRef();
00307     if (pref!=-1){
00308       // Must have an unsprayed margin so need to pass the information on to it
00309       LE* um=g_landscape_p->SupplyLEPointer(pref);
00310       um->SetLastTreatment( spring_harrow );
00311       um->InsectMortality( 0.7 );
00312       um->ZeroVeg();
00313     }
00314     return true;
00315   }
00316   return false;
00317 }
```

|  |  |  |  |
| --- | --- | --- | --- |
| bool Farm::SpringPlough | ( | LE \* | *a\_field*, |
|  |  | double | *a\_user*, |
|  |  | int | *a\_days* |  |
|  | ) |  |  | `[virtual]` |

Carry out a ploughing event in the spring on a\_field.

References DO\_IT\_PROB, DoIt(), g\_landscape\_p, spring\_plough, and UNREFERENCED\_PARAMETER.

```
00271 {
00272   UNREFERENCED_PARAMETER( a_user );
00273   if ( (0 >= a_days) || (!g_weather->Raining() && DoIt(DO_IT_PROB))) {
00274     a_field->Trace( spring_plough );
00275     a_field->SetLastTreatment( spring_plough );
00276    // Apply 90% mortality to the insects
00277     a_field->InsectMortality( 0.1 );
00278    // Reduce the vegetation to zero
00279     int pref=a_field->GetUnsprayedMarginPolyRef();
00280     if (pref!=-1){
00281       // Must have an unsprayed margin so need to pass the information on to it
00282       LE* um=g_landscape_p->SupplyLEPointer(pref);
00283       um->SetLastTreatment( spring_plough );
00284       um->InsectMortality( 0.1);
00285       um->ZeroVeg();
00286     }
00287     return true;
00288   }
00289   return false;
00290 }
```

|  |  |  |  |
| --- | --- | --- | --- |
| bool Farm::SpringRoll | ( | LE \* | *a\_field*, |
|  |  | double | *a\_user*, |
|  |  | int | *a\_days* |  |
|  | ) |  |  | `[virtual]` |

Carry out a roll event in the spring on a\_field.

References DO\_IT\_PROB, DoIt(), g\_landscape\_p, spring\_roll, and UNREFERENCED\_PARAMETER.

Referenced by WinterWheat::Do().

```
00324 {
00325   UNREFERENCED_PARAMETER( a_user );
00326   if ( (0 >= a_days) || (!g_weather->Raining() && DoIt(DO_IT_PROB))) {
00327     a_field->Trace( spring_roll );
00328     a_field->SetLastTreatment( spring_roll );
00329     int pref=a_field->GetUnsprayedMarginPolyRef();
00330     if (pref!=-1){
00331       // Must have an unsprayed margin so need to pass the information on to it
00332       LE* um=g_landscape_p->SupplyLEPointer(pref);
00333       um->SetLastTreatment( spring_roll );
00334       um->ZeroVeg();
00335     }
00336     return true;
00337   }
00338   return false;
00339 }
```

|  |  |  |  |
| --- | --- | --- | --- |
| bool Farm::SpringSow | ( | LE \* | *a\_field*, |
|  |  | double | *a\_user*, |
|  |  | int | *a\_days* |  |
|  | ) |  |  | `[virtual]` |

Carry out a sowing event in the spring on a\_field.

References DO\_IT\_PROB, DoIt(), g\_landscape\_p, spring\_sow, and UNREFERENCED\_PARAMETER.

```
00346 {
00347   UNREFERENCED_PARAMETER( a_user );
00348   if ( (0 >= a_days) || (!g_weather->Raining() && DoIt(DO_IT_PROB))) {
00349     a_field->Trace( spring_sow );
00350     a_field->SetLastTreatment( spring_sow );
00351     a_field->SetGrowthPhase( sow );
00352    // Reduce the  vegetation to zero - should not strictly be necessary, but prevents any false starts in the crop growth.
00353     a_field->ZeroVeg();
00354     int pref=a_field->GetUnsprayedMarginPolyRef();
00355    if (pref!=-1){
00356      // Must have an unsprayed margin so need to pass the information on to it
00357      LE* um=g_landscape_p->SupplyLEPointer(pref);
00358      um->SetLastTreatment( spring_sow );
00359      um->SetGrowthPhase( sow );
00360      um->ZeroVeg();
00361    }
00362    return true;
00363   }
00364   return false;
00365 }
```

|  |  |  |  |
| --- | --- | --- | --- |
| bool Farm::StrawChopping | ( | LE \* | *a\_field*, |
|  |  | double | *a\_user*, |
|  |  | int | *a\_days* |  |
|  | ) |  |  | `[virtual]` |

Carry out straw chopping on a\_field.

References DO\_IT\_PROB, DoIt(), g\_landscape\_p, straw\_chopping, and UNREFERENCED\_PARAMETER.

Referenced by WinterWheat::Do().

```
01481 {
01482   UNREFERENCED_PARAMETER( a_user );
01483   if ( (0 >= a_days) ||
01484        ((g_weather->GetRainPeriod(g_date->Date(),5)<0.1) && DoIt(DO_IT_PROB))
01485   ) {
01486     a_field->Trace( straw_chopping );
01487     a_field->SetLastTreatment( straw_chopping );
01488     a_field->InsectMortality( 0.4 );
01489     a_field->SetTramlinesDecay( EL_TRAMLINE_DECAYTIME );
01490     int pref=a_field->GetUnsprayedMarginPolyRef();
01491     if (pref!=-1){
01492       // Must have an unsprayed margin so need to pass the information on to it
01493       LE* um=g_landscape_p->SupplyLEPointer(pref);
01494       um->SetLastTreatment( straw_chopping );
01495       um->InsectMortality( 0.4 );
01496       um->SetTramlinesDecay( EL_TRAMLINE_DECAYTIME );
01497     }
01498     return true;
01499   }
01500   return false;
01501 }
```

|  |  |  |  |
| --- | --- | --- | --- |
| bool Farm::Strigling | ( | LE \* | *a\_field*, |
|  |  | double | *a\_user*, |
|  |  | int | *a\_days* |  |
|  | ) |  |  | `[virtual]` |

Carry out a mechanical weeding on a\_field.

References DO\_IT\_PROB, DoIt(), g\_landscape\_p, strigling, and UNREFERENCED\_PARAMETER.

Referenced by WinterWheat::Do().

```
01068 {
01069   UNREFERENCED_PARAMETER( a_user );
01070   // Force strigling if it has not been done already!!!  This happens regardless of weather as of 26/10/2005
01071   if ( (0 >= a_days) )// && (g_weather->GetRainPeriod(g_date->Date(),3)>0.1) )
01072   {
01073     a_field->Trace( strigling );
01074     a_field->SetLastTreatment( strigling );
01075     a_field->ReduceWeedBiomass( 0.05 );
01076     a_field->InsectMortality( 0.7 );
01077     a_field->SetTramlinesDecay( EL_TRAMLINE_DECAYTIME );
01078     a_field->SetHerbicideDelay( EL_STRIGLING_DELAYTIME );
01079     int pref=a_field->GetUnsprayedMarginPolyRef();
01080     if (pref!=-1){
01081       // Must have an unsprayed margin so need to pass the information on to it
01082       LE* um=g_landscape_p->SupplyLEPointer(pref);
01083       um->SetLastTreatment( strigling );
01084       um->ReduceWeedBiomass( 0.05 );
01085       um->InsectMortality( 0.7 );
01086       um->SetTramlinesDecay( EL_TRAMLINE_DECAYTIME );
01087       um->SetHerbicideDelay( EL_STRIGLING_DELAYTIME );
01088     return true;
01089   }
01090   }
01091   if ( (0 >= a_days) ||
01092        ((g_weather->GetRainPeriod(g_date->Date(),3)<0.1) && DoIt(DO_IT_PROB))
01093   ) {
01094     a_field->Trace( strigling );
01095     a_field->SetLastTreatment( strigling );
01096     a_field->ReduceWeedBiomass( 0.05 );
01097     a_field->InsectMortality( 0.7 );
01098     a_field->SetTramlinesDecay( EL_TRAMLINE_DECAYTIME );
01099     a_field->SetHerbicideDelay( EL_STRIGLING_DELAYTIME );
01100     int pref=a_field->GetUnsprayedMarginPolyRef();
01101     if (pref!=-1){
01102       // Must have an unsprayed margin so need to pass the information on to it
01103       LE* um=g_landscape_p->SupplyLEPointer(pref);
01104       um->SetLastTreatment( strigling );
01105       um->ReduceWeedBiomass( 0.05 );
01106       um->InsectMortality( 0.7 );
01107       um->SetTramlinesDecay( EL_TRAMLINE_DECAYTIME );
01108       um->SetHerbicideDelay( EL_STRIGLING_DELAYTIME );
01109     }
01110     return true;
01111   }
01112   return false;
01113 }
```

|  |  |  |  |
| --- | --- | --- | --- |
| bool Farm::StriglingSow | ( | LE \* | *a\_field*, |
|  |  | double | *a\_user*, |
|  |  | int | *a\_days* |  |
|  | ) |  |  | `[virtual]` |

Carry out a mechanical weeding followed by sowing on a\_field.

References DO\_IT\_PROB, DoIt(), g\_landscape\_p, strigling\_sow, and UNREFERENCED\_PARAMETER.

```
01120 {
01121   UNREFERENCED_PARAMETER( a_user );
01122   //2 days good weather afterwards
01123   if ( (0 >= a_days) && (g_weather->GetRainPeriod(g_date->Date(),3)<0.1) )
01124   {
01125     return true;
01126   }
01127 
01128   if ( (0 >= a_days) ||
01129        ((g_weather->GetRainPeriod(g_date->Date(),3)<0.1) && DoIt(DO_IT_PROB))
01130   ) {
01131     a_field->Trace( strigling_sow );
01132     a_field->SetLastTreatment( strigling_sow );
01133     a_field->ReduceWeedBiomass( 0.05 );
01134     a_field->InsectMortality( 0.7 );
01135     a_field->SetTramlinesDecay( EL_TRAMLINE_DECAYTIME );
01136     a_field->SetHerbicideDelay( EL_STRIGLING_DELAYTIME );
01137     int pref=a_field->GetUnsprayedMarginPolyRef();
01138     if (pref!=-1){
01139       // Must have an unsprayed margin so need to pass the information on to it
01140       LE* um=g_landscape_p->SupplyLEPointer(pref);
01141       um->SetLastTreatment( strigling_sow );
01142       um->ReduceWeedBiomass( 0.05 );
01143       um->InsectMortality( 0.7 );
01144       um->SetTramlinesDecay( EL_TRAMLINE_DECAYTIME );
01145       um->SetHerbicideDelay( EL_STRIGLING_DELAYTIME );
01146     }
01147     return true;
01148   }
01149   return false;
01150 }
```

|  |  |  |  |
| --- | --- | --- | --- |
| bool Farm::StubbleHarrowing | ( | LE \* | *a\_field*, |
|  |  | double | *a\_user*, |
|  |  | int | *a\_days* |  |
|  | ) |  |  | `[virtual]` |

Carry out stubble harrowing on a\_field.

References DO\_IT\_PROB, DoIt(), g\_landscape\_p, stubble\_harrowing, and UNREFERENCED\_PARAMETER.

Referenced by WinterWheat::Do().

```
01558 {
01559   UNREFERENCED_PARAMETER( a_user );
01560   if ( (0 >= a_days) ||
01561        ((g_weather->GetRainPeriod(g_date->Date(),3)<0.1) && DoIt(DO_IT_PROB))
01562   ) {
01563     a_field->Trace( stubble_harrowing );
01564     a_field->SetLastTreatment( stubble_harrowing );
01565     a_field->InsectMortality( 0.25 );
01566     a_field->ZeroVeg();
01567     int pref=a_field->GetUnsprayedMarginPolyRef();
01568     if (pref!=-1){
01569       // Must have an unsprayed margin so need to pass the information on to it
01570       LE* um=g_landscape_p->SupplyLEPointer(pref);
01571       um->SetLastTreatment( stubble_harrowing );
01572       um->ZeroVeg();
01573       um->InsectMortality( 0.25 );
01574     }
01575     return true;
01576   }
01577   return false;
01578 }
```

|  |  |  |  |
| --- | --- | --- | --- |
| bool Farm::Swathing | ( | LE \* | *a\_field*, |
|  |  | double | *a\_user*, |
|  |  | int | *a\_days* |  |
|  | ) |  |  | `[virtual]` |

Cut the crop on a\_field and leave it lying (probably rape).

References DO\_IT\_PROB, DoIt(), g\_landscape\_p, swathing, and UNREFERENCED\_PARAMETER.

```
01211 {
01212   UNREFERENCED_PARAMETER( a_user );
01213   if ( (0 >= a_days) || (!g_weather->Raining() && DoIt(DO_IT_PROB))) {
01214     a_field->Trace( swathing );
01215     a_field->SetLastTreatment( swathing );
01216     a_field->InsectMortality( 0.5 );
01217     a_field->SetTramlinesDecay( EL_TRAMLINE_DECAYTIME );
01218     int pref=a_field->GetUnsprayedMarginPolyRef();
01219     if (pref!=-1){
01220       // Must have an unsprayed margin so need to pass the information on to it
01221       LE* um=g_landscape_p->SupplyLEPointer(pref);
01222       um->SetLastTreatment( swathing );
01223       um->InsectMortality( 0.5 );
01224       um->SetTramlinesDecay( EL_TRAMLINE_DECAYTIME );
01225     }
01226     return true;
01227   }
01228   return false;
01229 }
```

|  |  |  |  |
| --- | --- | --- | --- |
| bool Farm::SynInsecticideTreat | ( | LE \* | *a\_field*, |
|  |  | double | *a\_user*, |
|  |  | int | *a\_days* |  |
|  | ) |  |  | `[virtual]` |

Apply special insecticide to a\_field.

References l\_farm\_insecticide\_kills(), syninsecticide\_treat, and UNREFERENCED\_PARAMETER.

```
00902 {
00903   UNREFERENCED_PARAMETER( a_user );
00904   // NOTE Differs from normal insecticide in that it will be done on the last
00905   // day if not managed before
00906   if (0 >= a_days) {
00907     if ( ! (a_field->GetSignal() & LE_SIG_NO_SYNG_INSECT )) {
00908       a_field->Trace( syninsecticide_treat );
00909       a_field->SetLastTreatment( syninsecticide_treat );
00910       if ( l_farm_insecticide_kills.value()) {
00911        a_field->Insecticide( 0.2 );
00912       }
00913       a_field->SetTramlinesDecay( EL_TRAMLINE_DECAYTIME );
00914     }
00915 //    double p=l_pest_insecticide_amount.value();
00916 //    g_pest->DailyQueueAdd( a_field,p );
00917     return true;
00918   } else {
00919     if ( (!g_weather->Raining()) && (g_weather->GetWind()<4.5)) {
00920       if ( ! (a_field->GetSignal() & LE_SIG_NO_SYNG_INSECT ) ) {
00921        a_field->Trace( syninsecticide_treat );
00922        a_field->SetLastTreatment( syninsecticide_treat );
00923        if ( l_farm_insecticide_kills.value()) {
00924          a_field->Insecticide( 0.2 );
00925        }
00926        a_field->SetTramlinesDecay( EL_TRAMLINE_DECAYTIME );
00927       }
00928 //      g_pest->DailyQueueAdd( a_field, l_pest_insecticide_amount.value());
00929       return true;
00930     }
00931   }
00932   return false;
00933 }
```

|  |  |  |  |  |  |
| --- | --- | --- | --- | --- | --- |
| TTypesOfVegetation Farm::TranslateCropCodes | ( | const char \* | *str* | ) |  |

References tov\_AgroChemIndustryCereal, tov\_Carrots, tov\_CloverGrassGrazed1, tov\_CloverGrassGrazed2, tov\_FieldPeas, tov\_FieldPeasStrigling, tov\_FodderBeet, tov\_Maize, tov\_MaizeStrigling, tov\_Oats, tov\_OBarleyPeaCloverGrass, tov\_OCarrots, tov\_OCloverGrassGrazed1, tov\_OCloverGrassGrazed2, tov\_OFieldPeas, tov\_OFieldPeasSilage, tov\_OGrazingPigs, tov\_OOats, tov\_OPermanentGrassGrazed, tov\_OPotatoes, tov\_OSeedGrass1, tov\_OSeedGrass2, tov\_OSpringBarley, tov\_OSpringBarleyClover, tov\_OSpringBarleyGrass, tov\_OTriticale, tov\_OWinterBarley, tov\_OWinterRape, tov\_OWinterRye, tov\_OWinterWheatUndersown, tov\_PermanentGrassGrazed, tov\_PermanentGrassLowGrazed, tov\_PermanentSetaside, tov\_Potatoes, tov\_PotatoesIndustry, tov\_SeedGrass1, tov\_SeedGrass2, tov\_Setaside, tov\_SpringBarley, tov\_SpringBarleyCloverGrass, tov\_SpringBarleyCloverGrassStrigling, tov\_SpringBarleyGrass, tov\_SpringBarleyPeaCloverGrassStrigling, tov\_SpringBarleySeed, tov\_SpringBarleySilage, tov\_SpringBarleyStrigling, tov\_SpringBarleyStriglingCulm, tov\_SpringBarleyStriglingSingle, tov\_SpringRape, tov\_SpringWheat, tov\_Triticale, tov\_WinterBarley, tov\_WinterBarleyStrigling, tov\_WinterRape, tov\_WinterRapeStrigling, tov\_WinterRye, tov\_WinterRyeStrigling, tov\_WinterWheat, tov\_WinterWheatShort, tov\_WinterWheatStrigling, tov\_WinterWheatStriglingCulm, tov\_WinterWheatStriglingSingle, tov\_WWheatPControl, tov\_WWheatPToxicControl, tov\_WWheatPTreatment, and tov\_YoungForest.

Referenced by OrganicCattle::OrganicCattle(), OrganicPig::OrganicPig(), OrganicPlant::OrganicPlant(), PesticideTrialTreatment::PesticideTrialTreatment(), UserDefinedFarm1::UserDefinedFarm1(), UserDefinedFarm10::UserDefinedFarm10(), UserDefinedFarm11::UserDefinedFarm11(), UserDefinedFarm12::UserDefinedFarm12(), UserDefinedFarm13::UserDefinedFarm13(), UserDefinedFarm14::UserDefinedFarm14(), UserDefinedFarm15::UserDefinedFarm15(), UserDefinedFarm16::UserDefinedFarm16(), UserDefinedFarm2::UserDefinedFarm2(), UserDefinedFarm3::UserDefinedFarm3(), UserDefinedFarm4::UserDefinedFarm4(), UserDefinedFarm5::UserDefinedFarm5(), UserDefinedFarm6::UserDefinedFarm6(), UserDefinedFarm7::UserDefinedFarm7(), UserDefinedFarm8::UserDefinedFarm8(), and UserDefinedFarm9::UserDefinedFarm9().

```
01663                                                                {
01664   // This simply checks through the list of legal crop names and returns
01665   // the correct tov type
01666 
01667   string str = astr;
01668 
01669   // Unfortunately switch cannot use string so the long way:
01670   if ( str == "SpringBarley" ) return tov_SpringBarley;
01671   if ( str == "WinterBarley" ) return tov_WinterBarley;
01672   if ( str == "SpringWheat" ) return tov_SpringWheat;
01673   if ( str == "WinterWheat" ) return tov_WinterWheat;
01674   if ( str == "WinterRye" ) return tov_WinterRye;
01675   if ( str == "Oats" ) return tov_Oats;
01676   if ( str == "OOats" ) return tov_OOats;
01677   if ( str == "Triticale" ) return tov_Triticale;
01678   if ( str == "Maize" ) return tov_Maize;
01679   if ( str == "SpringBarleySeed" ) return tov_SpringBarleySeed;
01680   if ( str == "SpringRape" ) return tov_SpringRape;
01681   if ( str == "WinterRape" ) return tov_WinterRape;
01682   if ( str == "FieldPeas" ) return tov_FieldPeas;
01683   if ( str == "Setaside" ) return tov_Setaside;
01684   if ( str == "PermanentSetaside" ) return tov_PermanentSetaside;
01685   if ( str == "FodderBeet" ) return tov_FodderBeet;
01686   if ( str == "CloverGrassGrazed1" ) return tov_CloverGrassGrazed1;
01687   if ( str == "PotatoesIndustry" ) return tov_PotatoesIndustry;
01688   if ( str == "Potatoes" ) return tov_Potatoes;
01689   if ( str == "SeedGrass1" ) return tov_SeedGrass1;
01690   if ( str == "OWinterBarley" ) return tov_OWinterBarley;
01691   if ( str == "SpringBarleySilage" ) return tov_SpringBarleySilage;
01692   if ( str == "OWinterRye" ) return tov_OWinterRye;
01693   if ( str == "OFieldPeasSilage" ) return tov_OFieldPeasSilage;
01694   if ( str == "SpringBarleyGrass" ) return tov_SpringBarleyGrass;
01695   if ( str == "SpringBarleyCloverGrass" ) return tov_SpringBarleyCloverGrass;
01696   if ( str == "OBarleyPeaCloverGrass" ) return tov_OBarleyPeaCloverGrass;
01697   if ( str == "OWinterRape" ) return tov_OWinterRape;
01698   if ( str == "PermanentGrassGrazed" ) return tov_PermanentGrassGrazed;
01699   if ( str == "PermanentGrassLowGrazed" ) return tov_PermanentGrassLowGrazed;
01700   if ( str == "CloverGrassGrazed2" ) return tov_CloverGrassGrazed2;
01701   if ( str == "SeedGrass2" ) return tov_SeedGrass2;
01702   if ( str == "OSpringBarley" ) return tov_OSpringBarley;
01703   if ( str == "OWinterWheatUndersown" ) return tov_OWinterWheatUndersown;
01704   if ( str == "OOats" ) return tov_OOats;
01705   if ( str == "OTriticale" ) return tov_OTriticale;
01706   if ( str == "OFieldPeas" ) return tov_OFieldPeas;
01707   if ( str == "OCloverGrassGrazed1" ) return tov_OCloverGrassGrazed1;
01708   if ( str == "OGrazingPigs" ) return tov_OGrazingPigs;
01709   if ( str == "OCarrots" ) return tov_OCarrots;
01710   if ( str == "Carrots" ) return tov_Carrots;
01711   if ( str == "OPotatoes" ) return tov_OPotatoes;
01712   if ( str == "OSeedGrass1" ) return tov_OSeedGrass1;
01713   if ( str == "OSpringBarleyGrass" ) return tov_OSpringBarleyGrass;
01714   if ( str == "OSpringBarleyClover" ) return tov_OSpringBarleyClover;
01715   if ( str == "OPermanentGrassGrazed" ) return tov_OPermanentGrassGrazed;
01716   if ( str == "OCloverGrassGrazed2" ) return tov_OCloverGrassGrazed2;
01717   if ( str == "OSeedGrass2" ) return tov_OSeedGrass2;
01718   if ( str == "WWheatPControl" ) return tov_WWheatPControl;
01719   if ( str == "WWheatPToxicControl" ) return tov_WWheatPToxicControl;
01720   if ( str == "WWheatPTreatment" ) return tov_WWheatPTreatment;
01721   if ( str == "AgroChemIndustryCereal" ) return tov_AgroChemIndustryCereal;
01722   if ( str == "WinterWheatShort" ) return tov_WinterWheatShort;
01723   if ( str == "WinterWheatStrigling" ) return tov_WinterWheatStrigling;
01724   if ( str == "WinterWheatStriglingCulm" ) return tov_WinterWheatStriglingCulm;
01725   if ( str == "WinterWheatStriglingSgl" ) return tov_WinterWheatStriglingSingle;
01726   if ( str == "SpringBarleyCloverGrassStrigling" ) return tov_SpringBarleyCloverGrassStrigling;
01727   if ( str == "SpringBarleyStrigling" ) return tov_SpringBarleyStrigling;
01728   if ( str == "SpringBarleyStriglingSingle" ) return tov_SpringBarleyStriglingSingle;
01729   if ( str == "SpringBarleyStriglingCulm" ) return tov_SpringBarleyStriglingCulm;
01730   if ( str == "MaizeStrigling" ) return tov_MaizeStrigling;
01731   if ( str == "WinterRapeStrigling" ) return tov_WinterRapeStrigling;
01732   if ( str == "WinterRyeStrigling" ) return tov_WinterRyeStrigling;
01733   if ( str == "WinterBarleyStrigling" ) return tov_WinterBarleyStrigling;
01734   if ( str == "FieldPeasStrigling" ) return tov_FieldPeasStrigling;
01735   if ( str == "SpringBarleyPeaCloverGrassStrigling" ) return tov_SpringBarleyPeaCloverGrassStrigling;
01736   if (str == "YoungForest") return tov_YoungForest;
01737 
01738   // No match so issue a warning and quit
01739   g_msg->Warn( WARN_FILE, "Farm::TranslateCropCodes():"" Unknown Crop Code ", str.c_str() );
01740   exit( 1 );
01741 }
```

|  |  |  |  |
| --- | --- | --- | --- |
| bool Farm::Trial\_Control | ( | LE \* | *a\_field*, |
|  |  | double | *a\_user*, |
|  |  | int | *a\_days* |  |
|  | ) |  |  | `[virtual]` |

Special pesticide trial functionality.

References trial\_control, and UNREFERENCED\_PARAMETER.

```
01736 {
01737   UNREFERENCED_PARAMETER( a_user );
01738   UNREFERENCED_PARAMETER( a_days );
01739   // Will always do this at the first chance
01740   a_field->Trace( trial_control );
01741   a_field->SetLastTreatment( trial_control );
01742   a_field->SetTramlinesDecay( EL_TRAMLINE_DECAYTIME );
01743   return true;
01744 }
```

|  |  |  |  |
| --- | --- | --- | --- |
| bool Farm::Trial\_InsecticideTreat | ( | LE \* | *a\_field*, |
|  |  | double | *a\_user*, |
|  |  | int | *a\_days* |  |
|  | ) |  |  | `[virtual]` |

Special pesticide trial functionality.

References trial\_insecticidetreat, and UNREFERENCED\_PARAMETER.

```
01705 {
01706    UNREFERENCED_PARAMETER( a_days );
01707    UNREFERENCED_PARAMETER( a_user );
01708     a_field->Trace( trial_insecticidetreat );
01709     a_field->SetLastTreatment( trial_insecticidetreat );
01710     a_field->InsectMortality( 0.1 );
01711     a_field->SetTramlinesDecay( EL_TRAMLINE_DECAYTIME );
01712     return true;
01713 }
```

|  |  |  |  |
| --- | --- | --- | --- |
| bool Farm::Trial\_ToxicControl | ( | LE \* | *a\_field*, |
|  |  | double | *a\_user*, |
|  |  | int | *a\_days* |  |
|  | ) |  |  | `[virtual]` |

Special pesticide trial functionality.

References trial\_toxiccontrol, and UNREFERENCED\_PARAMETER.

```
01720 {
01721     UNREFERENCED_PARAMETER( a_user );
01722     UNREFERENCED_PARAMETER( a_days );
01723     a_field->Trace( trial_toxiccontrol );
01724     a_field->SetLastTreatment( trial_toxiccontrol );
01725     a_field->InsectMortality( 0.001 );
01726     a_field->SetTramlinesDecay( EL_TRAMLINE_DECAYTIME );
01727     return true;
01728 }
```

|  |  |  |  |
| --- | --- | --- | --- |
| bool Farm::Water | ( | LE \* | *a\_field*, |
|  |  | double | *a\_user*, |
|  |  | int | *a\_days* |  |
|  | ) |  |  | `[virtual]` |

Carry out a watering on a\_field.

References DO\_IT\_PROB, DoIt(), g\_landscape\_p, UNREFERENCED\_PARAMETER, and water.

Referenced by WinterWheat::Do().

```
01184 {
01185   UNREFERENCED_PARAMETER( a_user );
01186 
01187   /* Turn on this code to avoid watering on heavy soils
01188   int soiltype = a_field->GetSoilType();
01189   if ( soiltype < 1 || soiltype > 4 )
01190     return true;
01191 */
01192   if ( (0 >= a_days) || (!g_weather->Raining() && DoIt(DO_IT_PROB))) {
01193     a_field->Trace( water );
01194     a_field->SetLastTreatment( water );
01195     int pref=a_field->GetUnsprayedMarginPolyRef();
01196     if (pref!=-1){
01197       // Must have an unsprayed margin so need to pass the information on to it
01198       LE* um=g_landscape_p->SupplyLEPointer(pref);
01199       um->SetLastTreatment( water );
01200     }
01201     return true;
01202   }
01203   return false;
01204 }
```

|  |  |  |  |
| --- | --- | --- | --- |
| bool Farm::WinterPlough | ( | LE \* | *a\_field*, |
|  |  | double | *a\_user*, |
|  |  | int | *a\_days* |  |
|  | ) |  |  | `[virtual]` |

Carry out a ploughing event in the winter on a\_field.

References DO\_IT\_PROB, DoIt(), g\_landscape\_p, UNREFERENCED\_PARAMETER, and winter\_plough.

```
00219 {
00220     UNREFERENCED_PARAMETER( a_user );
00221     if ( (0 >= a_days) || (!g_weather->Raining() && DoIt(DO_IT_PROB))) {
00222     a_field->Trace( winter_plough );
00223     a_field->SetLastTreatment( winter_plough );
00224     a_field->InsectMortality( 0.1 );
00225     a_field->ZeroVeg();
00226     int pref=a_field->GetUnsprayedMarginPolyRef();
00227     if (pref!=-1){
00228       // Must have an unsprayed margin so need to pass the information on to it
00229       LE* um=g_landscape_p->SupplyLEPointer(pref);
00230       um->SetLastTreatment( winter_plough );
00231       um->InsectMortality( 0.1 );
00232       um->ZeroVeg();
00233     }
00234     return true;
00235   }
00236   return false;
00237 }
```

---

## Member Data Documentation

|  |
| --- |
| AgroChemIndustryCereal\* Farm::m\_agrochemindustrycereal `[protected]` |

Referenced by Farm(), GetFirstDate(), LeSwitch(), and ~Farm().

|  |
| --- |
| Carrots\* Farm::m\_carrots `[protected]` |

Referenced by Farm(), GetFirstDate(), LeSwitch(), and ~Farm().

|  |
| --- |
| CloverGrassGrazed1\* Farm::m\_CGG1 `[protected]` |

Referenced by Farm(), GetFirstDate(), LeSwitch(), and ~Farm().

|  |
| --- |
| CloverGrassGrazed2\* Farm::m\_CGG2 `[protected]` |

Referenced by Farm(), GetFirstDate(), LeSwitch(), and ~Farm().

|  |
| --- |
| int Farm::m\_farm\_num `[protected]` |

Referenced by GetFarmNumber(), and SetFarmNumber().

|  |
| --- |
| TTypesOfFarm Farm::m\_farmtype `[protected]` |

Referenced by AgroChemIndustryCerealFarm1::AgroChemIndustryCerealFarm1(), AgroChemIndustryCerealFarm2::AgroChemIndustryCerealFarm2(), AgroChemIndustryCerealFarm3::AgroChemIndustryCerealFarm3(), CheckRotationManagementLoop(), ConventionalCattle::ConventionalCattle(), ConventionalPig::ConventionalPig(), ConventionalPlant::ConventionalPlant(), ConvMarginalJord::ConvMarginalJord(), GetType(), NoPesticideBaseFarm::NoPesticideBaseFarm(), NoPesticideNoPFarm::NoPesticideNoPFarm(), OrganicCattle::OrganicCattle(), OrganicPig::OrganicPig(), OrganicPlant::OrganicPlant(), PesticideTrialControl::PesticideTrialControl(), PesticideTrialToxicControl::PesticideTrialToxicControl(), PesticideTrialTreatment::PesticideTrialTreatment(), UserDefinedFarm1::UserDefinedFarm1(), UserDefinedFarm10::UserDefinedFarm10(), UserDefinedFarm11::UserDefinedFarm11(), UserDefinedFarm12::UserDefinedFarm12(), UserDefinedFarm13::UserDefinedFarm13(), UserDefinedFarm14::UserDefinedFarm14(), UserDefinedFarm15::UserDefinedFarm15(), UserDefinedFarm16::UserDefinedFarm16(), UserDefinedFarm2::UserDefinedFarm2(), UserDefinedFarm3::UserDefinedFarm3(), UserDefinedFarm4::UserDefinedFarm4(), UserDefinedFarm5::UserDefinedFarm5(), UserDefinedFarm6::UserDefinedFarm6(), UserDefinedFarm7::UserDefinedFarm7(), UserDefinedFarm8::UserDefinedFarm8(), and UserDefinedFarm9::UserDefinedFarm9().

|  |
| --- |
| FieldPeas\* Farm::m\_fieldpeas `[protected]` |

Referenced by Farm(), GetFirstDate(), LeSwitch(), and ~Farm().

|  |
| --- |
| FieldPeasStrigling\* Farm::m\_fieldpeasstrigling `[protected]` |

Referenced by Farm(), LeSwitch(), and ~Farm().

|  |
| --- |
| vector< LE\* > Farm::m\_fields `[protected]` |

Referenced by AddField(), InitiateManagement(), Management(), and RemoveField().

|  |
| --- |
| Fodderbeet\* Farm::m\_fodderbeet `[protected]` |

Referenced by Farm(), GetFirstDate(), LeSwitch(), and ~Farm().

|  |
| --- |
| bool Farm::m\_intensity `[protected]` |

Referenced by Farm(), and GetIntensity().

|  |
| --- |
| Maize\* Farm::m\_maize `[protected]` |

Referenced by Farm(), GetFirstDate(), LeSwitch(), and ~Farm().

|  |
| --- |
| MaizeStrigling\* Farm::m\_maizestrigling `[protected]` |

Referenced by Farm(), LeSwitch(), and ~Farm().

|  |
| --- |
| Oats\* Farm::m\_oats `[protected]` |

Referenced by Farm(), GetFirstDate(), LeSwitch(), and ~Farm().

|  |
| --- |
| OBarleyPeaCloverGrass\* Farm::m\_OBarleyPCG `[protected]` |

Referenced by Farm(), GetFirstDate(), LeSwitch(), and ~Farm().

|  |
| --- |
| OCarrots\* Farm::m\_ocarrots `[protected]` |

Referenced by Farm(), GetFirstDate(), LeSwitch(), and ~Farm().

|  |
| --- |
| OCloverGrassGrazed1\* Farm::m\_OCGG1 `[protected]` |

Referenced by Farm(), GetFirstDate(), LeSwitch(), and ~Farm().

|  |
| --- |
| OCloverGrassGrazed2\* Farm::m\_OCGG2 `[protected]` |

Referenced by Farm(), GetFirstDate(), LeSwitch(), and ~Farm().

|  |
| --- |
| OCloverGrassSilage1\* Farm::m\_OCGS1 `[protected]` |

Referenced by Farm(), GetFirstDate(), LeSwitch(), and ~Farm().

|  |
| --- |
| OFieldPeas\* Farm::m\_ofieldpeas `[protected]` |

Referenced by Farm(), GetFirstDate(), LeSwitch(), and ~Farm().

|  |
| --- |
| OFieldPeasSilage\* Farm::m\_ofieldpeassilage `[protected]` |

Referenced by Farm(), LeSwitch(), and ~Farm().

|  |
| --- |
| OFirstYearDanger\* Farm::m\_ofirstyeardanger `[protected]` |

Referenced by Farm(), GetFirstDate(), and ~Farm().

|  |
| --- |
| OGrazingPigs\* Farm::m\_ograzingpigs `[protected]` |

Referenced by Farm(), GetFirstDate(), LeSwitch(), and ~Farm().

|  |
| --- |
| OOats\* Farm::m\_ooats `[protected]` |

Referenced by Farm(), GetFirstDate(), LeSwitch(), and ~Farm().

|  |
| --- |
| OPermanentGrassGrazed\* Farm::m\_opermgrassgrazed `[protected]` |

Referenced by Farm(), GetFirstDate(), LeSwitch(), and ~Farm().

|  |
| --- |
| OPotatoes\* Farm::m\_opotatoes `[protected]` |

Referenced by Farm(), GetFirstDate(), LeSwitch(), and ~Farm().

|  |
| --- |
| OSBarleySilage\* Farm::m\_OSBarleysilage `[protected]` |

Referenced by Farm(), GetFirstDate(), and ~Farm().

|  |
| --- |
| OSpringBarley\* Farm::m\_ospringbarley `[protected]` |

Referenced by Farm(), GetFirstDate(), LeSwitch(), and ~Farm().

|  |
| --- |
| OSpringBarleyPigs\* Farm::m\_ospringbarleypigs `[protected]` |

Referenced by Farm(), GetFirstDate(), LeSwitch(), and ~Farm().

|  |
| --- |
| OWinterBarley\* Farm::m\_owinterbarley `[protected]` |

Referenced by Farm(), GetFirstDate(), LeSwitch(), and ~Farm().

|  |
| --- |
| OWinterRape\* Farm::m\_owinterrape `[protected]` |

Referenced by Farm(), GetFirstDate(), LeSwitch(), and ~Farm().

|  |
| --- |
| OWinterRye\* Farm::m\_owinterrye `[protected]` |

Referenced by Farm(), GetFirstDate(), LeSwitch(), and ~Farm().

|  |
| --- |
| OWinterWheatUndersown\* Farm::m\_owinterwheatundersown `[protected]` |

Referenced by Farm(), GetFirstDate(), LeSwitch(), and ~Farm().

|  |
| --- |
| PermanentSetAside\* Farm::m\_permanentsetaside `[protected]` |

Referenced by Farm(), GetFirstDate(), LeSwitch(), and ~Farm().

|  |
| --- |
| PermanentGrassGrazed\* Farm::m\_permgrassgrazed `[protected]` |

Referenced by Farm(), GetFirstDate(), LeSwitch(), and ~Farm().

|  |
| --- |
| PermanentGrassLowGrazed\* Farm::m\_permgrasslowgrazed `[protected]` |

Referenced by Farm(), GetFirstDate(), LeSwitch(), and ~Farm().

|  |
| --- |
| Potatoes\* Farm::m\_potatoes `[protected]` |

Referenced by Farm(), GetFirstDate(), LeSwitch(), and ~Farm().

|  |
| --- |
| LowPriority< FarmEvent\* > Farm::m\_queue `[protected]` |

Referenced by AddNewEvent(), HandleEvents(), and ~Farm().

|  |
| --- |
| vector<TTypesOfVegetation> Farm::m\_rotation `[protected]` |

Referenced by AgroChemIndustryCerealFarm1::AgroChemIndustryCerealFarm1(), AgroChemIndustryCerealFarm2::AgroChemIndustryCerealFarm2(), AgroChemIndustryCerealFarm3::AgroChemIndustryCerealFarm3(), CheckRotationManagementLoop(), ConventionalCattle::ConventionalCattle(), ConventionalPig::ConventionalPig(), ConventionalPlant::ConventionalPlant(), ConvMarginalJord::ConvMarginalJord(), GetFirstCropIndex(), GetNextCropIndex(), GetNextCropStartDate(), HandleEvents(), InitiateManagement(), NoPesticideBaseFarm::NoPesticideBaseFarm(), NoPesticideNoPFarm::NoPesticideNoPFarm(), OrganicCattle::OrganicCattle(), OrganicPig::OrganicPig(), OrganicPlant::OrganicPlant(), PesticideTrialControl::PesticideTrialControl(), PesticideTrialToxicControl::PesticideTrialToxicControl(), PesticideTrialTreatment::PesticideTrialTreatment(), UserDefinedFarm1::UserDefinedFarm1(), UserDefinedFarm10::UserDefinedFarm10(), UserDefinedFarm11::UserDefinedFarm11(), UserDefinedFarm12::UserDefinedFarm12(), UserDefinedFarm13::UserDefinedFarm13(), UserDefinedFarm14::UserDefinedFarm14(), UserDefinedFarm15::UserDefinedFarm15(), UserDefinedFarm16::UserDefinedFarm16(), UserDefinedFarm2::UserDefinedFarm2(), UserDefinedFarm3::UserDefinedFarm3(), UserDefinedFarm4::UserDefinedFarm4(), UserDefinedFarm5::UserDefinedFarm5(), UserDefinedFarm6::UserDefinedFarm6(), UserDefinedFarm7::UserDefinedFarm7(), UserDefinedFarm8::UserDefinedFarm8(), and UserDefinedFarm9::UserDefinedFarm9().

|  |
| --- |
| int Farm::m\_rotation\_sync\_index `[protected]` |

Referenced by Farm(), and GetFirstCropIndex().

|  |
| --- |
| SpringBarleyCloverGrass\* Farm::m\_sbarleyclovergrass `[protected]` |

Referenced by Farm(), GetFirstDate(), LeSwitch(), and ~Farm().

|  |
| --- |
| SeedGrass1\* Farm::m\_seedgrass1 `[protected]` |

Referenced by Farm(), GetFirstDate(), LeSwitch(), and ~Farm().

|  |
| --- |
| SeedGrass2\* Farm::m\_seedgrass2 `[protected]` |

Referenced by Farm(), GetFirstDate(), LeSwitch(), and ~Farm().

|  |
| --- |
| SetAside\* Farm::m\_setaside `[protected]` |

Referenced by Farm(), GetFirstDate(), LeSwitch(), and ~Farm().

|  |
| --- |
| SpringBarley\* Farm::m\_springbarley `[protected]` |

Referenced by Farm(), GetFirstDate(), LeSwitch(), and ~Farm().

|  |
| --- |
| SpringBarleyCloverGrassStrigling\* Farm::m\_springbarleyclovergrassstrigling `[protected]` |

Referenced by Farm(), LeSwitch(), and ~Farm().

|  |
| --- |
| SpringBarleyPeaCloverGrassStrigling\* Farm::m\_springbarleypeaclovergrassstrigling `[protected]` |

Referenced by Farm(), LeSwitch(), and ~Farm().

|  |
| --- |
| SpringBarleySeed\* Farm::m\_springbarleyseed `[protected]` |

Referenced by Farm(), GetFirstDate(), LeSwitch(), and ~Farm().

|  |
| --- |
| SpringBarleySilage\* Farm::m\_springbarleysilage `[protected]` |

Referenced by Farm(), GetFirstDate(), LeSwitch(), and ~Farm().

|  |
| --- |
| SpringBarleyStrigling\* Farm::m\_springbarleystrigling `[protected]` |

Referenced by Farm(), GetFirstDate(), LeSwitch(), and ~Farm().

|  |
| --- |
| SpringBarleyStriglingCulm\* Farm::m\_springbarleystriglingculm `[protected]` |

Referenced by Farm(), GetFirstDate(), LeSwitch(), and ~Farm().

|  |
| --- |
| SpringBarleyStriglingSingle\* Farm::m\_springbarleystriglingsingle `[protected]` |

Referenced by Farm(), GetFirstDate(), LeSwitch(), and ~Farm().

|  |
| --- |
| bool Farm::m\_stockfarmer `[protected]` |

Referenced by AgroChemIndustryCerealFarm1::AgroChemIndustryCerealFarm1(), AgroChemIndustryCerealFarm2::AgroChemIndustryCerealFarm2(), AgroChemIndustryCerealFarm3::AgroChemIndustryCerealFarm3(), ConventionalCattle::ConventionalCattle(), ConventionalPig::ConventionalPig(), ConventionalPlant::ConventionalPlant(), ConvMarginalJord::ConvMarginalJord(), Farm(), IsStockFarmer(), PesticideTrialTreatment::MakeStockFarmer(), PesticideTrialToxicControl::MakeStockFarmer(), PesticideTrialControl::MakeStockFarmer(), OrganicPlant::MakeStockFarmer(), ConventionalPlant::MakeStockFarmer(), MakeStockFarmer(), NoPesticideBaseFarm::NoPesticideBaseFarm(), NoPesticideNoPFarm::NoPesticideNoPFarm(), OrganicCattle::OrganicCattle(), OrganicPig::OrganicPig(), OrganicPlant::OrganicPlant(), PesticideTrialControl::PesticideTrialControl(), PesticideTrialToxicControl::PesticideTrialToxicControl(), PesticideTrialTreatment::PesticideTrialTreatment(), UserDefinedFarm1::UserDefinedFarm1(), UserDefinedFarm10::UserDefinedFarm10(), UserDefinedFarm11::UserDefinedFarm11(), UserDefinedFarm12::UserDefinedFarm12(), UserDefinedFarm13::UserDefinedFarm13(), UserDefinedFarm14::UserDefinedFarm14(), UserDefinedFarm15::UserDefinedFarm15(), UserDefinedFarm16::UserDefinedFarm16(), UserDefinedFarm2::UserDefinedFarm2(), UserDefinedFarm3::UserDefinedFarm3(), UserDefinedFarm4::UserDefinedFarm4(), UserDefinedFarm5::UserDefinedFarm5(), UserDefinedFarm6::UserDefinedFarm6(), UserDefinedFarm7::UserDefinedFarm7(), UserDefinedFarm8::UserDefinedFarm8(), and UserDefinedFarm9::UserDefinedFarm9().

|  |
| --- |
| Triticale\* Farm::m\_triticale `[protected]` |

Referenced by Farm(), GetFirstDate(), LeSwitch(), and ~Farm().

|  |
| --- |
| WinterBarley\* Farm::m\_winterbarley `[protected]` |

Referenced by Farm(), GetFirstDate(), LeSwitch(), and ~Farm().

|  |
| --- |
| WinterBarleyStrigling\* Farm::m\_winterbarleystrigling `[protected]` |

Referenced by Farm(), LeSwitch(), and ~Farm().

|  |
| --- |
| WinterRape\* Farm::m\_winterrape `[protected]` |

Referenced by Farm(), GetFirstDate(), LeSwitch(), and ~Farm().

|  |
| --- |
| WinterRapeStrigling\* Farm::m\_winterrapestrigling `[protected]` |

Referenced by Farm(), LeSwitch(), and ~Farm().

|  |
| --- |
| WinterRye\* Farm::m\_winterrye `[protected]` |

Referenced by Farm(), GetFirstDate(), LeSwitch(), and ~Farm().

|  |
| --- |
| WinterRyeStrigling\* Farm::m\_winterryestrigling `[protected]` |

Referenced by Farm(), LeSwitch(), and ~Farm().

|  |
| --- |
| WinterWheat\* Farm::m\_winterwheat `[protected]` |

Referenced by Farm(), GetFirstDate(), LeSwitch(), and ~Farm().

|  |
| --- |
| WinterWheatStrigling\* Farm::m\_winterwheatstrigling `[protected]` |

Referenced by Farm(), GetFirstDate(), LeSwitch(), and ~Farm().

|  |
| --- |
| WinterWheatStriglingCulm\* Farm::m\_winterwheatstriglingculm `[protected]` |

Referenced by Farm(), GetFirstDate(), LeSwitch(), and ~Farm().

|  |
| --- |
| WinterWheatStriglingSingle\* Farm::m\_winterwheatstriglingsingle `[protected]` |

Referenced by Farm(), GetFirstDate(), LeSwitch(), and ~Farm().

|  |
| --- |
| WWheatPControl\* Farm::m\_wwheatpcontrol `[protected]` |

Referenced by Farm(), GetFirstDate(), LeSwitch(), and ~Farm().

|  |
| --- |
| WWheatPToxicControl\* Farm::m\_wwheatptoxiccontrol `[protected]` |

Referenced by Farm(), GetFirstDate(), LeSwitch(), and ~Farm().

|  |
| --- |
| WWheatPTreatment\* Farm::m\_wwheatptreatment `[protected]` |

Referenced by Farm(), GetFirstDate(), LeSwitch(), and ~Farm().

|  |
| --- |
| YoungForestCrop\* Farm::m\_youngforest `[protected]` |

Referenced by Farm(), GetFirstDate(), LeSwitch(), and ~Farm().

---

The documentation for this class was generated from the following files:

- farm.h- farm.cpp- farmfuncs.cpp

---

Generated on Thu Jan 22 14:13:46 2009 for ALMaSS ODDox by 
 1.5.6 
