# Supplementary material for: Predation and fragmentation portrayed in the statistical structure of prey time series
Source: BMC Ecol. 2009 May 6;9:10. doi: 10.1186/1472-6785-9-10 (PMC2689204; doi:10.1186/1472-6785-9-10)
Supplement: Additional file 2 — Voles and related classes ODDox Documentation. ODDox documentation of the agent-based model (ALMaSS) applied by Hendrichsen et al. The documentation is started by activating main.html. [file 1472-6785-9-10-S2.zip › Vole_ODDox/class_genetic_material.html]

ALMaSS ODDox: GeneticMaterial Class Reference

- Main Page
- Related Pages
- Classes
- Files

- Alphabetical List
- Class List
- Class Hierarchy
- Class Members

# GeneticMaterial Class Reference

`#include <GeneticMaterial.H>`

List of all members.

---

## Detailed Description

Class for the genetic material optionally carried by animals in ALMaSS.

|  |
| --- |
|  |
| Public Member Functions | |
|  | GeneticMaterial () |
| uint32 | GetAllele (int pos, int Chromosome) |
| uint32 | GetDirectFlag () |
| uint32 | GetGeneticFlag () |
| int | HeterozygosityCount () |
| int | HomozygosityCount () |
| void | Initiation (AlleleFreq \*Al) |
| void | Mutation\_1 () |
| void | Mutation\_1ab () |
| void | Mutation\_2 () |
| void | Mutation\_3 () |
| void | PrintChromosome (char \*C, int Chromosome) |
| void | ReadFrequencies () |
| void | Recombine (GeneticMaterial \*Gen21, GeneticMaterial \*Gene2) |
| float | ScoreHQThreshold () |
| float | ScoreReproduction () |
| void | SetAllele (int pos, uint32 value, int Chromosome) |
| void | SetDirectFlag () |
| void | SetGeneticFlag () |
| void | UnsetDirectFlag () |
| void | UnsetGeneticFlag () |
| Protected Attributes | |
| uint32 | Chromosome [6] |

---

## Constructor & Destructor Documentation

|  |  |  |  |  |
| --- | --- | --- | --- | --- |
| GeneticMaterial::GeneticMaterial | ( |  | ) |  |

References Chromosome.

```
00330                                  {
00331   // ensure zeros in all loci
00332   for ( int i = 0; i < 6; i++ ) Chromosome[ i ] = 0;
00333 }
```

---

## Member Function Documentation

|  |  |  |  |
| --- | --- | --- | --- |
| uint32 GeneticMaterial::GetAllele | ( | int | *pos*, |
|  |  | int | *Chromosome* |  |
|  | ) |  |  |  |

References Chromosome.

Referenced by Vole\_Population\_Manager::CreateObjects(), GetDirectFlag(), GetGeneticFlag(), HeterozygosityCount(), HomozygosityCount(), Mutation\_2(), Mutation\_3(), PrintChromosome(), Recombine(), and Vole\_Base::SupplyAllele().

```
00208                                                          {
00209   uint32 value;
00210   // Get the right chromosome
00211   // if Chromo==0 then 0-2, else 3-5
00212   Chromo *= 3; // 0 or 3
00213   if ( locus < 16 ) {
00214     // Shift it so the locus is in the last two bits
00215     // Does it twice because 32 bis coding for 16 loci
00216     value = Chromosome[ Chromo ] >> locus;
00217     value = ( value >> locus ) & 0x03;
00218   } else {
00219     Chromo++; // 1 or 4
00220     locus -= 16; // Now 0 to 16
00221     if ( locus >= 8 ) {
00222       Chromo++; // 2 or 5
00223       locus -= 8;
00224     }
00225     value = Chromosome[ Chromo ] >> ( locus * 4 );
00226     value = value & 0x0f;
00227   }
00228   return value;
00229 }
```

|  |  |  |  |  |
| --- | --- | --- | --- | --- |
| uint32 GeneticMaterial::GetDirectFlag | ( |  | ) |  |

References GetAllele().

Referenced by Vole\_Base::GetDirectFlag().

```
00166                                       {
00167         return GetAllele(0,1);
00168 }
```

|  |  |  |  |  |
| --- | --- | --- | --- | --- |
| uint32 GeneticMaterial::GetGeneticFlag | ( |  | ) |  |

References GetAllele().

Referenced by Vole\_Base::GetGeneticFlag().

```
00162                                        {
00163         return GetAllele(0,0);
00164 }
```

|  |  |  |  |  |
| --- | --- | --- | --- | --- |
| int GeneticMaterial::HeterozygosityCount | ( |  | ) |  |

References GetAllele().

Referenced by Vole\_Base::SupplyHeteroZyg().

```
00303                                          {
00304   int heterozyg = 0;
00305   for ( int i = 0; i < 32; i++ ) {
00306     if ( GetAllele( i, 0 ) != GetAllele( i, 1 ) ) heterozyg++;
00307   }
00308   return heterozyg;
00309 }
```

|  |  |  |  |  |
| --- | --- | --- | --- | --- |
| int GeneticMaterial::HomozygosityCount | ( |  | ) |  |

References GetAllele().

Referenced by Vole\_Base::SupplyHomoZyg().

```
00292                                        {
00293   // OK OK there is an easy way to do this by calling HeterozygosityCount and
00294   // subtracting this from 32, but just is case that little bit of saved time is useful:
00295   int homozyg=0;
00296   for ( int i = 0; i < 32; i++ ) {
00297     if ( GetAllele( i, 0 ) == GetAllele( i, 1 ) ) homozyg++;
00298   }
00299   return homozyg;
00300 }
```

|  |  |  |  |  |  |
| --- | --- | --- | --- | --- | --- |
| void GeneticMaterial::Initiation | ( | AlleleFreq \* | *Al* | ) |  |

The method called to intialise genes on initiation of the simulation.   
Gene frequencies are based on an external text file input read in on construction.

References SetAllele(), and AlleleFreq::SupplyAN().

Referenced by Vole\_Population\_Manager::Init().

```
00341                                                   {
00342   uint32 value, c;
00343   for ( int l = 0; l < 32; l++ ) {
00344     if ( l < 16 ) c = 0; else if ( l < 24 ) c = 1; else c = 2;
00345 
00346     int chance = random( 1000 );
00347     uint32 index = 0;
00348     while ( chance > Al->SupplyAN( l, index ) ) {
00349       index++;
00350     }
00351     value = index;
00352     // set the value
00353     SetAllele( l, value, 0 );
00354     chance = random( 1000 );
00355     index = 0;
00356     while ( chance > Al->SupplyAN( l, index ) ) {
00357       index++;
00358     }
00359     value = index;
00360     // set the value
00361     SetAllele( l, value, 1 );
00362   }
00363 }
```

|  |  |  |  |  |
| --- | --- | --- | --- | --- |
| void GeneticMaterial::Mutation\_1 | ( |  | ) |  |

random allele choice

References MutationChance, and SetAllele().

Referenced by Vole\_Female::st\_Lactating().

```
00417 {
00418   for ( int i = 0; i < 16; i++ ) {
00419     if ( random( MutationChance ) == 1 ) // one chance in Mutation Chance
00420     {
00421       SetAllele( i, random( 4 ), random( 2 ) );
00422     }
00423   }
00424   for ( int i = 16; i < 32; i++ ) {
00425     if ( random( MutationChance ) == 1 ) // one chance in Mutation Chance
00426     {
00427       SetAllele( i, random( 16 ), random( 2 ) );
00428     }
00429   }
00430 }
```

|  |  |  |  |  |
| --- | --- | --- | --- | --- |
| void GeneticMaterial::Mutation\_1ab | ( |  | ) |  |

random allele choice a & b only

References MutationChance, and SetAllele().

```
00438 {
00439   // Only used when all loci have only two alleles!
00440   for ( int i = 0; i < 32; i++ ) {
00441     if ( random( MutationChance ) == 1 ) // one chance in Mutation Chance
00442     {
00443       SetAllele( i, random( 2 ), random( 2 ) );
00444     }
00445   }
00446 }
```

|  |  |  |  |  |
| --- | --- | --- | --- | --- |
| void GeneticMaterial::Mutation\_2 | ( |  | ) |  |

Move one allele +/-

References GetAllele(), MutationChance, and SetAllele().

```
00454 {
00455   for ( int i = 0; i < 16; i++ ) {
00456     if ( random( MutationChance ) == 1 ) // one chance in Mutation Chance
00457     {
00458       int strand = random( 2 );
00459       int allele = GetAllele( i, strand );
00460       if ( random( 2 ) == 1 ) allele++; else allele--;
00461       if ( allele == -1 ) allele = 3; else if ( allele == 4 ) allele = 0;
00462       SetAllele( i, allele, strand );
00463     }
00464   }
00465   for ( int i = 16; i < 32; i++ ) {
00466     if ( random( MutationChance ) == 1 ) // one chance in Mutation Chance
00467     {
00468       int strand = random( 2 );
00469       int allele = GetAllele( i, strand );
00470       if ( random( 2 ) == 1 ) allele++; else allele--;
00471       if ( allele == -1 ) allele = 15; else if ( allele == 16 ) allele = 0;
00472       SetAllele( i, allele, strand );
00473     }
00474   }
00475 }
```

|  |  |  |  |  |
| --- | --- | --- | --- | --- |
| void GeneticMaterial::Mutation\_3 | ( |  | ) |  |

switch a<->b & c<->d

References GetAllele(), MutationChance, and SetAllele().

```
00483 {
00484   // NB Only works for the first 16 loci
00485   for ( int i = 0; i < 16; i++ ) {
00486     if ( random( MutationChance ) == 1 ) // one chance in Mutation Chance
00487     {
00488       int strand = random( 2 );
00489       int allele = GetAllele( i, strand );
00490       switch ( allele ) {
00491         case 0:
00492           allele = 1;
00493         break;
00494         case 1:
00495           allele = 0;
00496         break;
00497         case 2:
00498           allele = 3;
00499         break;
00500         case 3:
00501           allele = 2;
00502         break;
00503       }
00504       SetAllele( i, allele, strand );
00505     }
00506   }
00507 }
```

|  |  |  |  |
| --- | --- | --- | --- |
| void GeneticMaterial::PrintChromosome | ( | char \* | *C*, |
|  |  | int | *Chromosome* |  |
|  | ) |  |  |  |

References GetAllele().

```
00233                                                             {
00234   for ( int i = 0; i < 16; i++ ) {
00235     uint32 allele = GetAllele( i, Chromo );
00236     switch ( allele ) {
00237       case 0:
00238         C[ i ] = 'a';
00239       break;
00240       case 1:
00241         C[ i ] = 'b';
00242       break;
00243       case 2:
00244         C[ i ] = 'c';
00245       break;
00246       case 3:
00247         C[ i ] = 'd';
00248       break;
00249       case 4:
00250         C[ i ] = 'e';
00251       break;
00252       case 5:
00253         C[ i ] = 'f';
00254       break;
00255       case 6:
00256         C[ i ] = 'g';
00257       break;
00258       case 7:
00259         C[ i ] = 'h';
00260       break;
00261       case 8:
00262         C[ i ] = 'i';
00263       break;
00264       case 9:
00265         C[ i ] = 'j';
00266       break;
00267       case 10:
00268         C[ i ] = 'k';
00269       break;
00270       case 11:
00271         C[ i ] = 'l';
00272       break;
00273       case 12:
00274         C[ i ] = 'm';
00275       break;
00276       case 13:
00277         C[ i ] = 'n';
00278       break;
00279       case 14:
00280         C[ i ] = 'o';
00281       break;
00282       case 15:
00283         C[ i ] = 'p';
00284       break;
00285     }
00286   }
00287   C[ 16 ] = 0;
00288 }
```

|  |  |  |  |  |
| --- | --- | --- | --- | --- |
| void GeneticMaterial::ReadFrequencies | ( |  | ) |  |

|  |  |  |  |
| --- | --- | --- | --- |
| void GeneticMaterial::Recombine | ( | GeneticMaterial \* | *Gen21*, |
|  |  | GeneticMaterial \* | *Gene2* |  |
|  | ) |  |  |  |

References GetAllele(), and SetAllele().

Referenced by Vole\_Base::CopyMyself(), and Vole\_Female::st\_Lactating().

```
00313                                                                                   {
00314   for ( int i = 0; i < 32; i++ ) {
00315     // For each locus
00316     // Choose which chromosome for each parent
00317     int g0 = random( 2 );
00318     int g1 = random( 2 );
00319     // get the two alleles
00320     uint32 a0 = Gene1->GetAllele( i, g0 );
00321     uint32 a1 = Gene2->GetAllele( i, g1 );
00322     //  put a0 into chromo0 & a1 to chromo1 & vice versa
00323     SetAllele( i, a0, 0 );
00324     SetAllele( i, a1, 1 );
00325   }
00326 }
```

|  |  |  |  |  |
| --- | --- | --- | --- | --- |
| float GeneticMaterial::ScoreHQThreshold | ( |  | ) |  |

This function can be used to alter fitness based on associated genetic codes. These are only used in population genetic research, e.g. to create hybrid zones.

Referenced by Vole\_Male::Init(), and Vole\_Female::Init().

```
00389                                         {
00390   return 1.0;
00391   // OLD CODE OUTDATED
00392   /* Ditte's Simulation Version uint32 allele0a = GetAllele(0,0); // loci 0 uint32 allele1a = GetAllele(1,0); // loci 1
00393   uint32 allele0b = GetAllele(0,1); // loci 0 uint32 allele1b = GetAllele(1,1); // loci 1
00394   // Initial rules are that if 0a and 1a are 0 & 2 or 2 & 0 then OK   (a,c)(c,a)
00395   // likewise they may be 1 & 3 or 3 & 1  (b,d) (d,b) // any other combination is bad // Same for loci 1 bool IsOK0=false;
00396   switch(allele0a) { case 0: if (allele1a==2) IsOK0=true; break; case 1: if (allele1a==3) IsOK0=true; break; case 2:
00397   if (allele1a==0) IsOK0=true; break; case 3: if (allele1a==1) IsOK0=true; break; default: assert(NULL); break; }
00398   bool IsOK1=false; switch(allele0b) { case 0: if (allele1b==2) IsOK1=true; break; case 1: if (allele1b==3) IsOK1=true; break;
00399   case 2: if (allele1b==0) IsOK1=true; break; case 3: if (allele1b==1) IsOK1=true; break; default: assert(NULL); break; }
00400   // determine the effect of the genetics // In the simple case it is good or bad
00401   if (IsOK0 && IsOK1) return 1.0; else return 0.9;
00402 
00403   // Lar Bach's version uint32 allele2a = GetAllele(2,0); // loci 0 uint32 allele2b = GetAllele(2,1); // loci 0
00404   float result= -0.5; switch (allele2a) { case 0: case 2: case 3: break; default: result+=0.5; } switch (allele2b) { case 0:
00405   case 2: case 3: break; default: result+=0.5; break; } return result;
00406   // returns -0.5 if homozygous aa, 0.5 if homozygous bb & het=0
00407 
00408   */
00409 }
```

|  |  |  |  |  |
| --- | --- | --- | --- | --- |
| float GeneticMaterial::ScoreReproduction | ( |  | ) |  |

This function can be used to alter reproductive effects based on genetic codes. These are only used in population genetic research.

Referenced by Vole\_Base::Vole\_Base().

```
00369                                          {
00370   return 1.0;
00371   /* OLD CODE OUTDATED uint32 allele0a = GetAllele(0,0); // loci 0 uint32 allele1a = GetAllele(1,0); // loci 1
00372   uint32 allele0b = GetAllele(0,1); // loci 0 uint32 allele1b = GetAllele(1,1); // loci 1
00373   // Initial rules are that locus 0 and 1 are ac or bd then OK // likewise they may be ca or db
00374   // any other combination is bad // Same for loci 1 bool IsOK0=false; switch(allele0a) { case 0:   //a
00375   if (allele1a==2) IsOK0=true; break; case 1:   //b if (allele1a==3) IsOK0=true; break; case 2:   //c
00376   if (allele1a==0) IsOK0=true; break; case 3:   //d if (allele1a==1) IsOK0=true; break; default:
00377   FILE* errfile=fopen("GeneticErrorFile.Txt","w"); fprintf(errfile,"Unknown Allele Number\n"); fclose(errfile); exit(10);
00378   break; } bool IsOK1=false; switch(allele0b) { case 0: if (allele1b==2) IsOK1=true; break; case 1:
00379   if (allele1b==3) IsOK1=true; break; case 2: if (allele1b==0) IsOK1=true; break; case 3: if (allele1b==1) IsOK1=true; break;
00380   default: FILE* errfile=fopen("GeneticErrorFile.Txt","w"); fprintf(errfile,"Unknown Allele Number\n"); fclose(errfile);
00381   exit(11); break; } // determine the effect of the genetics // In the simple case it is good or bad
00382   if (IsOK0 && IsOK1) return 1.0; else return 0.05; */
00383 }
```

|  |  |  |  |
| --- | --- | --- | --- |
| void GeneticMaterial::SetAllele | ( | int | *pos*, |
|  |  | uint32 | *value*, |
|  |  | int | *Chromosome* |  |
|  | ) |  |  |  |

References Chromosome.

Referenced by Vole\_Population\_Manager::Init(), Initiation(), Mutation\_1(), Mutation\_1ab(), Mutation\_2(), Mutation\_3(), Recombine(), SetDirectFlag(), SetGeneticFlag(), UnsetDirectFlag(), and UnsetGeneticFlag().

```
00171                                                                      {
00172   Chromo*=3; // now 0 or 3
00173   uint32 mask;
00174   if (locus<16) {
00175   // Get the right chromosome
00176   // Create the mask
00177   // Does it twice because 32 bits coding for 16 loci
00178   mask = 0x03 << locus;
00179   mask = mask << locus;
00180   // just to make make sure it is 0-3
00181   value = value & 0x03;
00182   // create the value mask
00183   value = value << locus;
00184   value = value << locus;
00185   // clear the locus
00186   Chromosome[ Chromo ] &= ~mask;
00187   // write the value
00188   Chromosome[ Chromo ] |= value;
00189   } else {
00190     Chromo++; // now 1 or 4
00191     locus-=16;
00192     if (locus>=8) {
00193       Chromo++;
00194       locus-=8;
00195     }
00196     mask = 0x0F << (locus*4);
00197     value = value & 0x0f; // make sure there was no extra stuff added!
00198     // create the value mask
00199     value = value << (locus*4);
00200     Chromosome[ Chromo ] &= ~mask;
00201     // write the value
00202     Chromosome[ Chromo ] |= value;
00203     }
00204 }
```

|  |  |  |  |  |
| --- | --- | --- | --- | --- |
| void GeneticMaterial::SetDirectFlag | ( |  | ) |  |

References SetAllele().

Referenced by Vole\_Base::SetDirectFlag().

```
00148                                     {
00149         SetAllele(0,1,1);
00150 }
```

|  |  |  |  |  |
| --- | --- | --- | --- | --- |
| void GeneticMaterial::SetGeneticFlag | ( |  | ) |  |

References SetAllele().

Referenced by Vole\_Base::SetGeneticFlag().

```
00144                                      {
00145         SetAllele(0,1,0);
00146 }
```

|  |  |  |  |  |
| --- | --- | --- | --- | --- |
| void GeneticMaterial::UnsetDirectFlag | ( |  | ) |  |

References SetAllele().

Referenced by Vole\_Base::UnsetDirectFlag().

```
00157                                       {
00158         SetAllele(0,0,1);
00159 }
```

|  |  |  |  |  |
| --- | --- | --- | --- | --- |
| void GeneticMaterial::UnsetGeneticFlag | ( |  | ) |  |

References SetAllele().

Referenced by Vole\_Base::UnsetGeneticFlag().

```
00153                                        {
00154         SetAllele(0,0,0);
00155 }
```

---

## Member Data Documentation

|  |
| --- |
| uint32 GeneticMaterial::Chromosome[6] `[protected]` |

Referenced by GeneticMaterial(), GetAllele(), and SetAllele().

---

The documentation for this class was generated from the following files:

- GeneticMaterial.H- GeneticMaterial.cpp

---

Generated on Thu Jan 22 14:13:46 2009 for ALMaSS ODDox by 
 1.5.6 
