# Supplementary material for: Predation and fragmentation portrayed in the statistical structure of prey time series
Source: BMC Ecol. 2009 May 6;9:10. doi: 10.1186/1472-6785-9-10 (PMC2689204; doi:10.1186/1472-6785-9-10)
Supplement: Additional file 2 — Voles and related classes ODDox Documentation. ODDox documentation of the agent-based model (ALMaSS) applied by Hendrichsen et al. The documentation is started by activating main.html. [file 1472-6785-9-10-S2.zip › Vole_ODDox/class_genetic_material1616-members.html]

ALMaSS ODDox: Member List

- Main Page
- Related Pages
- Classes
- Files

- Alphabetical List
- Class List
- Class Hierarchy
- Class Members

# GeneticMaterial1616 Member List

This is the complete list of members for GeneticMaterial1616, including all inherited members.

|  |  |  |
| --- | --- | --- |
| Chromosome | GeneticMaterial1616 | `[protected]` |
| GeneticMaterial1616() | GeneticMaterial1616 |  |
| GetAllele(unsigned int locus, unsigned int Chromo) | GeneticMaterial1616 |  |
| GetDirectFlag() | GeneticMaterial1616 |  |
| GetGeneticFlag() | GeneticMaterial1616 |  |
| HeterozygosityCount() | GeneticMaterial1616 |  |
| HomozygosityCount() | GeneticMaterial1616 |  |
| PrintChromosome(char \*C, unsigned int Chromosome) | GeneticMaterial1616 |  |
| SetAllele(unsigned int locus, uint32 value, unsigned int Chromo) | GeneticMaterial1616 |  |
| SetDirectFlag() | GeneticMaterial1616 |  |
| SetGeneticFlag() | GeneticMaterial1616 |  |
| UnsetDirectFlag() | GeneticMaterial1616 |  |
| UnsetGeneticFlag() | GeneticMaterial1616 |  |

---

Generated on Thu Jan 22 14:13:46 2009 for ALMaSS ODDox by 
 1.5.6 
