# Supplementary material for: Predation and fragmentation portrayed in the statistical structure of prey time series
Source: BMC Ecol. 2009 May 6;9:10. doi: 10.1186/1472-6785-9-10 (PMC2689204; doi:10.1186/1472-6785-9-10)
Supplement: Additional file 2 — Voles and related classes ODDox Documentation. ODDox documentation of the agent-based model (ALMaSS) applied by Hendrichsen et al. The documentation is started by activating main.html. [file 1472-6785-9-10-S2.zip › Vole_ODDox/class_genetic_material1616.html]

ALMaSS ODDox: GeneticMaterial1616 Class Reference

- Main Page
- Related Pages
- Classes
- Files

- Alphabetical List
- Class List
- Class Hierarchy
- Class Members

# GeneticMaterial1616 Class Reference

`#include <GeneticMaterial.H>`

List of all members.

|  |
| --- |
|  |
| Public Member Functions | |
|  | GeneticMaterial1616 () |
| uint32 | GetAllele (unsigned int locus, unsigned int Chromo) |
| uint32 | GetDirectFlag () |
| uint32 | GetGeneticFlag () |
| int | HeterozygosityCount () |
| int | HomozygosityCount () |
| void | PrintChromosome (char \*C, unsigned int Chromosome) |
| void | SetAllele (unsigned int locus, uint32 value, unsigned int Chromo) |
| void | SetDirectFlag () |
| void | SetGeneticFlag () |
| void | UnsetDirectFlag () |
| void | UnsetGeneticFlag () |
| Protected Attributes | |
| uint32 | Chromosome [4] |

---

## Constructor & Destructor Documentation

|  |  |  |  |  |
| --- | --- | --- | --- | --- |
| GeneticMaterial1616::GeneticMaterial1616 | ( |  | ) |  |

References Chromosome.

```
00515                                          {
00516   // ensure zeros in all loci
00517   for ( int i = 0; i < 4; i++ ) Chromosome[ i ] = 0;
00518 }
```

---

## Member Function Documentation

|  |  |  |  |
| --- | --- | --- | --- |
| uint32 GeneticMaterial1616::GetAllele | ( | unsigned int | *locus*, |
|  |  | unsigned int | *Chromo* |  |
|  | ) |  |  |  |

References Chromosome.

Referenced by GetDirectFlag(), GetGeneticFlag(), HeterozygosityCount(), HomozygosityCount(), and PrintChromosome().

```
00522                                                                                {
00523         // This is for 32 bit machines, 64bit is easier
00524         // locus must be 0 to 15
00525         //Chromo must be either 0 or 1
00526         // These debug tests below are costly so turn off in release code
00527         #ifdef __GENDEBUG
00528                 if (Chromo>1) {
00529                         g_msg->Warn( "Chromo > 1 in GeneticMaterial1616", NULL );
00530                         exit( 0 );
00531                 }
00532                 if (locus>15) {
00533                         g_msg->Warn( "locus > 15 in GeneticMaterial1616", NULL );
00534                         exit( 0 );
00535                 }
00536         #endif
00537     uint32 segment=((Chromo<<1) | (locus & 0x08));
00538         uint32 allele=0x0F & (Chromosome[segment]>>((locus & 7)<<2));
00539         return allele;
00540 }
```

|  |  |  |  |  |
| --- | --- | --- | --- | --- |
| uint32 GeneticMaterial1616::GetDirectFlag | ( |  | ) |  |

References GetAllele().

```
00669                                           {
00670         return GetAllele(0,1);
00671 }
```

|  |  |  |  |  |
| --- | --- | --- | --- | --- |
| uint32 GeneticMaterial1616::GetGeneticFlag | ( |  | ) |  |

References GetAllele().

```
00665                                            {
00666         return GetAllele(0,0);
00667 }
```

|  |  |  |  |  |
| --- | --- | --- | --- | --- |
| int GeneticMaterial1616::HeterozygosityCount | ( |  | ) |  |

References GetAllele().

```
00639                                              {
00640   int heterozyg = 0;
00641   for ( int i = 0; i < 16; i++ ) {
00642     if ( GetAllele( i, 0 ) != GetAllele( i, 1 ) ) heterozyg++;
00643   }
00644   return heterozyg;
00645 }
```

|  |  |  |  |  |
| --- | --- | --- | --- | --- |
| int GeneticMaterial1616::HomozygosityCount | ( |  | ) |  |

References GetAllele().

```
00628                                            {
00629   // OK OK there is an easy way to do this by calling HeterozygosityCount and
00630   // subtracting this from 32, but just is case that little bit of saved time is useful:
00631   int homozyg=0;
00632   for ( int i = 0; i < 16; i++ ) {
00633     if ( GetAllele( i, 0 ) == GetAllele( i, 1 ) ) homozyg++;
00634   }
00635   return homozyg;
00636 }
```

|  |  |  |  |
| --- | --- | --- | --- |
| void GeneticMaterial1616::PrintChromosome | ( | char \* | *C*, |
|  |  | unsigned int | *Chromosome* |  |
|  | ) |  |  |  |

References GetAllele().

```
00570                                                                          {
00571   for ( int i = 0; i < 16; i++ ) {
00572     uint32 allele = GetAllele( i, Chromo );
00573     switch ( allele ) {
00574       case 0:
00575         C[ i ] = 'a';
00576       break;
00577       case 1:
00578         C[ i ] = 'b';
00579       break;
00580       case 2:
00581         C[ i ] = 'c';
00582       break;
00583       case 3:
00584         C[ i ] = 'd';
00585       break;
00586       case 4:
00587         C[ i ] = 'e';
00588       break;
00589       case 5:
00590         C[ i ] = 'f';
00591       break;
00592       case 6:
00593         C[ i ] = 'g';
00594       break;
00595       case 7:
00596         C[ i ] = 'h';
00597       break;
00598       case 8:
00599         C[ i ] = 'i';
00600       break;
00601       case 9:
00602         C[ i ] = 'j';
00603       break;
00604       case 10:
00605         C[ i ] = 'k';
00606       break;
00607       case 11:
00608         C[ i ] = 'l';
00609       break;
00610       case 12:
00611         C[ i ] = 'm';
00612       break;
00613       case 13:
00614         C[ i ] = 'n';
00615       break;
00616       case 14:
00617         C[ i ] = 'o';
00618       break;
00619       case 15:
00620         C[ i ] = 'p';
00621       break;
00622     }
00623   }
00624   C[ 16 ] = 0;
00625 }
```

|  |  |  |  |
| --- | --- | --- | --- |
| void GeneticMaterial1616::SetAllele | ( | unsigned int | *locus*, |
|  |  | uint32 | *value*, |
|  |  | unsigned int | *Chromo* |  |
|  | ) |  |  |  |

References Chromosome.

Referenced by SetDirectFlag(), SetGeneticFlag(), UnsetDirectFlag(), and UnsetGeneticFlag().

```
00543                                                                                            {
00544         // This is for 32 bit machines, 64bit is easier
00545         // locus must be 0 to 15
00546         //Chromo must be either 0 or 1
00547         // These debug tests below are costly so turn off in release code
00548         #ifdef __GENDEBUG
00549                 if (Chromo>1) {
00550                         g_msg->Warn( "Chromo > 1 in GeneticMaterial1616", NULL );
00551                         exit( 0 );
00552                 }
00553                 if (locus>15) {
00554                         g_msg->Warn( "locus > 15 in GeneticMaterial1616", NULL );
00555                         exit( 0 );
00556                 }
00557         #endif
00558     uint32 segment=((Chromo<<1) | (locus & 0x08));
00559         uint32 mask = 0x0F;
00560         // Need to shift the mask over the correct allele
00561         mask=mask<<((locus&7)<<2);
00562         value = value & 0x0f; // make sure there was no extra stuff added!
00563     // create the value mask
00564     value = value << ((locus&7)<<2);
00565     Chromosome[ segment ] &= ~mask; // get rid of the current info
00566     Chromosome[ segment ] |= value; // write the value
00567 }
```

|  |  |  |  |  |
| --- | --- | --- | --- | --- |
| void GeneticMaterial1616::SetDirectFlag | ( |  | ) |  |

References SetAllele().

```
00651                                         {
00652         SetAllele(0,1,1);
00653 }
```

|  |  |  |  |  |
| --- | --- | --- | --- | --- |
| void GeneticMaterial1616::SetGeneticFlag | ( |  | ) |  |

References SetAllele().

```
00647                                          {
00648         SetAllele(0,1,0);
00649 }
```

|  |  |  |  |  |
| --- | --- | --- | --- | --- |
| void GeneticMaterial1616::UnsetDirectFlag | ( |  | ) |  |

References SetAllele().

```
00660                                           {
00661         SetAllele(0,0,1);
00662 }
```

|  |  |  |  |  |
| --- | --- | --- | --- | --- |
| void GeneticMaterial1616::UnsetGeneticFlag | ( |  | ) |  |

References SetAllele().

```
00656                                            {
00657         SetAllele(0,0,0);
00658 }
```

---

## Member Data Documentation

|  |
| --- |
| uint32 GeneticMaterial1616::Chromosome[4] `[protected]` |

Referenced by GeneticMaterial1616(), GetAllele(), and SetAllele().

---

The documentation for this class was generated from the following files:

- GeneticMaterial.H- GeneticMaterial.cpp

---

Generated on Thu Jan 22 14:13:46 2009 for ALMaSS ODDox by 
 1.5.6 
