# Supplementary material for: Predation and fragmentation portrayed in the statistical structure of prey time series
Source: BMC Ecol. 2009 May 6;9:10. doi: 10.1186/1472-6785-9-10 (PMC2689204; doi:10.1186/1472-6785-9-10)
Supplement: Additional file 2 — Voles and related classes ODDox Documentation. ODDox documentation of the agent-based model (ALMaSS) applied by Hendrichsen et al. The documentation is started by activating main.html. [file 1472-6785-9-10-S2.zip › Vole_ODDox/class_no_pesticide_base_farm.html]

ALMaSS ODDox: NoPesticideBaseFarm Class Reference

- Main Page
- Related Pages
- Classes
- Files

- Alphabetical List
- Class List
- Class Hierarchy
- Class Members

# NoPesticideBaseFarm Class Reference

`#include <farm.h>`

Inheritance diagram for NoPesticideBaseFarm:

List of all members.

---

## Detailed Description

Inbuilt special purpose farm type.

|  |
| --- |
|  |
| Public Member Functions | |
|  | NoPesticideBaseFarm (void) |

---

## Constructor & Destructor Documentation

|  |  |  |  |  |  |
| --- | --- | --- | --- | --- | --- |
| NoPesticideBaseFarm::NoPesticideBaseFarm | ( | void |  | ) |  |

References Farm::m\_farmtype, Farm::m\_rotation, Farm::m\_stockfarmer, tof\_NoPesticideBase, tov\_CloverGrassGrazed1, tov\_CloverGrassGrazed2, tov\_FieldPeas, tov\_FodderBeet, tov\_Setaside, tov\_SpringBarley, tov\_SpringBarleyCloverGrass, tov\_WinterBarley, tov\_WinterRape, tov\_WinterRye, and tov\_WinterWheat.

```
01241                                                : Farm() // 13
01242 {
01243   m_farmtype = tof_NoPesticideBase;
01244   m_stockfarmer = false;
01245 
01246   // Adjust as needed.
01247   m_rotation.resize( 36 );
01248   m_rotation[ 0 ] = tov_SpringBarleyCloverGrass;
01249   m_rotation[ 1 ] = tov_CloverGrassGrazed1;
01250   m_rotation[ 2 ] = tov_CloverGrassGrazed2;
01251   m_rotation[ 3 ] = tov_WinterWheat;
01252   m_rotation[ 4 ] = tov_SpringBarley;
01253   m_rotation[ 5 ] = tov_SpringBarleyCloverGrass;
01254   m_rotation[ 6 ] = tov_CloverGrassGrazed1;
01255   m_rotation[ 7 ] = tov_FodderBeet;
01256   m_rotation[ 8 ] = tov_SpringBarley;
01257   m_rotation[ 9 ] = tov_WinterRape;
01258   m_rotation[ 10 ] = tov_WinterWheat;
01259   m_rotation[ 11 ] = tov_SpringBarley;
01260   m_rotation[ 12 ] = tov_SpringBarley;
01261   m_rotation[ 13 ] = tov_Setaside;
01262   m_rotation[ 14 ] = tov_FieldPeas;
01263   m_rotation[ 15 ] = tov_WinterWheat;
01264   m_rotation[ 16 ] = tov_WinterRye;
01265   m_rotation[ 17 ] = tov_WinterBarley;
01266   m_rotation[ 18 ] = tov_WinterRape;
01267   m_rotation[ 19 ] = tov_WinterWheat;
01268   m_rotation[ 20 ] = tov_SpringBarley;
01269   m_rotation[ 21 ] = tov_SpringBarley;
01270   m_rotation[ 22 ] = tov_Setaside;
01271   m_rotation[ 23 ] = tov_FieldPeas;
01272   m_rotation[ 24 ] = tov_WinterWheat;
01273   m_rotation[ 25 ] = tov_WinterRye;
01274   m_rotation[ 26 ] = tov_WinterBarley;
01275   m_rotation[ 27 ] = tov_WinterRape;
01276   m_rotation[ 28 ] = tov_WinterWheat;
01277   m_rotation[ 29 ] = tov_SpringBarley;
01278   m_rotation[ 30 ] = tov_SpringBarley;
01279   m_rotation[ 31 ] = tov_Setaside;
01280   m_rotation[ 32 ] = tov_FieldPeas;
01281   m_rotation[ 33 ] = tov_WinterWheat;
01282   m_rotation[ 34 ] = tov_WinterRye;
01283   m_rotation[ 35 ] = tov_WinterBarley;
01284 }
```

---

The documentation for this class was generated from the following files:

- farm.h- farm.cpp

---

Generated on Thu Jan 22 14:13:46 2009 for ALMaSS ODDox by 
 1.5.6 
