# Supplementary material for: Predation and fragmentation portrayed in the statistical structure of prey time series
Source: BMC Ecol. 2009 May 6;9:10. doi: 10.1186/1472-6785-9-10 (PMC2689204; doi:10.1186/1472-6785-9-10)
Supplement: Additional file 2 — Voles and related classes ODDox Documentation. ODDox documentation of the agent-based model (ALMaSS) applied by Hendrichsen et al. The documentation is started by activating main.html. [file 1472-6785-9-10-S2.zip › Vole_ODDox/class_no_pesticide_no_p_farm.html]

ALMaSS ODDox: NoPesticideNoPFarm Class Reference

- Main Page
- Related Pages
- Classes
- Files

- Alphabetical List
- Class List
- Class Hierarchy
- Class Members

# NoPesticideNoPFarm Class Reference

`#include <farm.h>`

Inheritance diagram for NoPesticideNoPFarm:

List of all members.

---

## Detailed Description

Inbuilt special purpose farm type.

|  |
| --- |
|  |
| Public Member Functions | |
|  | NoPesticideNoPFarm (void) |

---

## Constructor & Destructor Documentation

|  |  |  |  |  |  |
| --- | --- | --- | --- | --- | --- |
| NoPesticideNoPFarm::NoPesticideNoPFarm | ( | void |  | ) |  |

References Farm::m\_farmtype, Farm::m\_rotation, Farm::m\_stockfarmer, tof\_NoPesticideNoP, tov\_OBarleyPeaCloverGrass, tov\_OCloverGrassGrazed1, tov\_OCloverGrassGrazed2, tov\_OFieldPeas, tov\_OWinterRape, tov\_OWinterRye, tov\_OWinterWheatUndersown, and tov\_Setaside.

```
01286                                              : Farm() // 14
01287 {
01288   m_farmtype = tof_NoPesticideNoP;
01289   m_stockfarmer = false;
01290 
01291   // Adjust as needed.
01292   m_rotation.resize( 17 );
01293   m_rotation[ 0 ] = tov_OBarleyPeaCloverGrass;
01294   m_rotation[ 1 ] = tov_OCloverGrassGrazed1;
01295   m_rotation[ 2 ] = tov_OCloverGrassGrazed2;
01296   m_rotation[ 3 ] = tov_OWinterRape;
01297   m_rotation[ 4 ] = tov_OFieldPeas;
01298   m_rotation[ 5 ] = tov_OBarleyPeaCloverGrass;
01299   m_rotation[ 6 ] = tov_OCloverGrassGrazed1;
01300   m_rotation[ 7 ] = tov_OWinterWheatUndersown;
01301   m_rotation[ 8 ] = tov_OCloverGrassGrazed1;
01302   m_rotation[ 9 ] = tov_OCloverGrassGrazed2;
01303   m_rotation[ 10 ] = tov_OFieldPeas;
01304   m_rotation[ 11 ] = tov_OBarleyPeaCloverGrass;
01305   m_rotation[ 12 ] = tov_OCloverGrassGrazed1;
01306   m_rotation[ 13 ] = tov_Setaside;
01307   m_rotation[ 14 ] = tov_OFieldPeas;
01308   m_rotation[ 15 ] = tov_OWinterRye;
01309   m_rotation[ 16 ] = tov_OFieldPeas;
01310 }
```

---

The documentation for this class was generated from the following files:

- farm.h- farm.cpp

---

Generated on Thu Jan 22 14:13:46 2009 for ALMaSS ODDox by 
 1.5.6 
