# Supplementary material for: Predation and fragmentation portrayed in the statistical structure of prey time series
Source: BMC Ecol. 2009 May 6;9:10. doi: 10.1186/1472-6785-9-10 (PMC2689204; doi:10.1186/1472-6785-9-10)
Supplement: Additional file 2 — Voles and related classes ODDox Documentation. ODDox documentation of the agent-based model (ALMaSS) applied by Hendrichsen et al. The documentation is started by activating main.html. [file 1472-6785-9-10-S2.zip › Vole_ODDox/class_organic_cattle-members.html]

ALMaSS ODDox: Member List

- Main Page
- Related Pages
- Classes
- Files

- Alphabetical List
- Class List
- Class Hierarchy
- Class Members

# OrganicCattle Member List

This is the complete list of members for OrganicCattle, including all inherited members.

|  |  |  |
| --- | --- | --- |
| AddField(LE \*a\_newfield) | Farm |  |
| AddNewEvent(TTypesOfVegetation a\_event, long a\_date, LE \*a\_field, int a\_todo, long a\_num, bool a\_lock, int a\_start, bool a\_first\_year, TTypesOfVegetation a\_crop) | Farm |  |
| AutumnHarrow(LE \*a\_field, double a\_user, int a\_days) | Farm | `[virtual]` |
| AutumnPlough(LE \*a\_field, double a\_user, int a\_days) | Farm | `[virtual]` |
| AutumnRoll(LE \*a\_field, double a\_user, int a\_days) | Farm | `[virtual]` |
| AutumnSow(LE \*a\_field, double a\_user, int a\_days) | Farm | `[virtual]` |
| BurnStrawStubble(LE \*a\_field, double a\_user, int a\_days) | Farm | `[virtual]` |
| CattleIsOut(LE \*a\_field, double a\_user, int a\_days, int a\_max) | Farm | `[virtual]` |
| CattleIsOutLow(LE \*a\_field, double a\_user, int a\_days, int a\_max) | Farm | `[virtual]` |
| CattleOut(LE \*a\_field, double a\_user, int a\_days) | Farm | `[virtual]` |
| CattleOutLowGrazing(LE \*a\_field, double a\_user, int a\_days) | Farm | `[virtual]` |
| CheckRotationManagementLoop(FarmEvent \*ev) | Farm | `[protected]` |
| CutToHay(LE \*a\_field, double a\_user, int a\_days) | Farm | `[virtual]` |
| CutToSilage(LE \*a\_field, double a\_user, int a\_days) | Farm | `[virtual]` |
| CutWeeds(LE \*a\_field, double a\_user, int a\_days) | Farm | `[virtual]` |
| DeepPlough(LE \*a\_field, double a\_user, int a\_days) | Farm | `[virtual]` |
| DoIt(int a\_probability) | Farm |  |
| FA\_AmmoniumSulphate(LE \*a\_field, double a\_user, int a\_days) | Farm | `[virtual]` |
| FA\_GreenManure(LE \*a\_field, double a\_user, int a\_days) | Farm | `[virtual]` |
| FA\_Manure(LE \*a\_field, double a\_user, int a\_days) | Farm | `[virtual]` |
| FA\_NPK(LE \*a\_field, double a\_user, int a\_days) | Farm | `[virtual]` |
| FA\_PK(LE \*a\_field, double a\_user, int a\_days) | Farm | `[virtual]` |
| FA\_Sludge(LE \*a\_field, double a\_user, int a\_days) | Farm | `[virtual]` |
| FA\_Slurry(LE \*a\_field, double a\_user, int a\_days) | Farm | `[virtual]` |
| Farm(void) | Farm |  |
| FP\_GreenManure(LE \*a\_field, double a\_user, int a\_days) | Farm | `[virtual]` |
| FP\_LiquidNH3(LE \*a\_field, double a\_user, int a\_days) | Farm | `[virtual]` |
| FP\_ManganeseSulphate(LE \*a\_field, double a\_user, int a\_days) | Farm | `[virtual]` |
| FP\_Manure(LE \*a\_field, double a\_user, int a\_days) | Farm | `[virtual]` |
| FP\_NPK(LE \*a\_field, double a\_user, int a\_days) | Farm | `[virtual]` |
| FP\_NPKS(LE \*a\_field, double a\_user, int a\_days) | Farm | `[virtual]` |
| FP\_PK(LE \*a\_field, double a\_user, int a\_days) | Farm | `[virtual]` |
| FP\_Sludge(LE \*a\_field, double a\_user, int a\_days) | Farm | `[virtual]` |
| FP\_Slurry(LE \*a\_field, double a\_user, int a\_days) | Farm | `[virtual]` |
| FungicideTreat(LE \*a\_field, double a\_user, int a\_days) | Farm | `[virtual]` |
| GetFarmNumber(void) | Farm | `[inline]` |
| GetFirstCropIndex(TTypesOfLandscapeElement a\_type) | Farm | `[protected, virtual]` |
| GetFirstDate(TTypesOfVegetation a\_tov) | Farm | `[protected]` |
| GetIntensity(void) | Farm | `[inline]` |
| GetNextCropIndex(int a\_rot\_index) | Farm | `[protected, virtual]` |
| GetNextCropStartDate(LE \*a\_field, TTypesOfVegetation &a\_curr\_veg) | Farm | `[protected]` |
| GetType(void) | Farm | `[inline]` |
| Glyphosate(LE \*a\_field, double a\_user, int a\_days) | Farm | `[virtual]` |
| GrowthRegulator(LE \*a\_field, double a\_user, int a\_days) | Farm | `[virtual]` |
| HandleEvents(void) | Farm | `[protected]` |
| Harvest(LE \*a\_field, double a\_user, int a\_days) | Farm | `[virtual]` |
| HayBailing(LE \*a\_field, double a\_user, int a\_days) | Farm | `[virtual]` |
| HayTurning(LE \*a\_field, double a\_user, int a\_days) | Farm | `[virtual]` |
| HerbicideTreat(LE \*a\_field, double a\_user, int a\_days) | Farm | `[virtual]` |
| HillingUp(LE \*a\_field, double a\_user, int a\_days) | Farm | `[virtual]` |
| InitiateManagement(void) | Farm | `[virtual]` |
| InsecticideTreat(LE \*a\_field, double a\_user, int a\_days) | Farm | `[virtual]` |
| IsStockFarmer(void) | Farm | `[inline]` |
| LeSwitch(FarmEvent \*ev) | Farm | `[protected]` |
| m\_agrochemindustrycereal | Farm | `[protected]` |
| m\_carrots | Farm | `[protected]` |
| m\_CGG1 | Farm | `[protected]` |
| m\_CGG2 | Farm | `[protected]` |
| m\_farm\_num | Farm | `[protected]` |
| m\_farmtype | Farm | `[protected]` |
| m\_fieldpeas | Farm | `[protected]` |
| m\_fieldpeasstrigling | Farm | `[protected]` |
| m\_fields | Farm | `[protected]` |
| m\_fodderbeet | Farm | `[protected]` |
| m\_intensity | Farm | `[protected]` |
| m\_maize | Farm | `[protected]` |
| m\_maizestrigling | Farm | `[protected]` |
| m\_oats | Farm | `[protected]` |
| m\_OBarleyPCG | Farm | `[protected]` |
| m\_ocarrots | Farm | `[protected]` |
| m\_OCGG1 | Farm | `[protected]` |
| m\_OCGG2 | Farm | `[protected]` |
| m\_OCGS1 | Farm | `[protected]` |
| m\_ofieldpeas | Farm | `[protected]` |
| m\_ofieldpeassilage | Farm | `[protected]` |
| m\_ofirstyeardanger | Farm | `[protected]` |
| m\_ograzingpigs | Farm | `[protected]` |
| m\_ooats | Farm | `[protected]` |
| m\_opermgrassgrazed | Farm | `[protected]` |
| m\_opotatoes | Farm | `[protected]` |
| m\_OSBarleysilage | Farm | `[protected]` |
| m\_ospringbarley | Farm | `[protected]` |
| m\_ospringbarleypigs | Farm | `[protected]` |
| m\_owinterbarley | Farm | `[protected]` |
| m\_owinterrape | Farm | `[protected]` |
| m\_owinterrye | Farm | `[protected]` |
| m\_owinterwheatundersown | Farm | `[protected]` |
| m\_permanentsetaside | Farm | `[protected]` |
| m\_permgrassgrazed | Farm | `[protected]` |
| m\_permgrasslowgrazed | Farm | `[protected]` |
| m\_potatoes | Farm | `[protected]` |
| m\_queue | Farm | `[protected]` |
| m\_rotation | Farm | `[protected]` |
| m\_rotation\_sync\_index | Farm | `[protected]` |
| m\_sbarleyclovergrass | Farm | `[protected]` |
| m\_seedgrass1 | Farm | `[protected]` |
| m\_seedgrass2 | Farm | `[protected]` |
| m\_setaside | Farm | `[protected]` |
| m\_springbarley | Farm | `[protected]` |
| m\_springbarleyclovergrassstrigling | Farm | `[protected]` |
| m\_springbarleypeaclovergrassstrigling | Farm | `[protected]` |
| m\_springbarleyseed | Farm | `[protected]` |
| m\_springbarleysilage | Farm | `[protected]` |
| m\_springbarleystrigling | Farm | `[protected]` |
| m\_springbarleystriglingculm | Farm | `[protected]` |
| m\_springbarleystriglingsingle | Farm | `[protected]` |
| m\_stockfarmer | Farm | `[protected]` |
| m\_triticale | Farm | `[protected]` |
| m\_winterbarley | Farm | `[protected]` |
| m\_winterbarleystrigling | Farm | `[protected]` |
| m\_winterrape | Farm | `[protected]` |
| m\_winterrapestrigling | Farm | `[protected]` |
| m\_winterrye | Farm | `[protected]` |
| m\_winterryestrigling | Farm | `[protected]` |
| m\_winterwheat | Farm | `[protected]` |
| m\_winterwheatstrigling | Farm | `[protected]` |
| m\_winterwheatstriglingculm | Farm | `[protected]` |
| m\_winterwheatstriglingsingle | Farm | `[protected]` |
| m\_wwheatpcontrol | Farm | `[protected]` |
| m\_wwheatptoxiccontrol | Farm | `[protected]` |
| m\_wwheatptreatment | Farm | `[protected]` |
| m\_youngforest | Farm | `[protected]` |
| MakeStockFarmer(void) | Farm | `[inline, virtual]` |
| Management(void) | Farm | `[virtual]` |
| Molluscicide(LE \*a\_field, double a\_user, int a\_days) | Farm | `[virtual]` |
| OrganicCattle(void) | OrganicCattle |  |
| PigsAreOut(LE \*a\_field, double a\_user, int a\_days) | Farm | `[virtual]` |
| PigsAreOutForced(LE \*a\_field, double a\_user, int a\_days) | Farm | `[virtual]` |
| PigsOut(LE \*a\_field, double a\_user, int a\_days) | Farm | `[virtual]` |
| ProductApplication0(LE \*a\_field, double a\_user, int a\_days) | Farm | `[virtual]` |
| ProductApplication1(LE \*a\_field, double a\_user, int a\_days) | Farm | `[virtual]` |
| RemoveField(LE \*a\_field) | Farm |  |
| RowCultivation(LE \*a\_field, double a\_user, int a\_days) | Farm | `[virtual]` |
| SetFarmNumber(int a\_farm\_num) | Farm | `[inline]` |
| SleepAllDay(LE \*a\_field, double a\_user, int a\_days) | Farm | `[virtual]` |
| SpringHarrow(LE \*a\_field, double a\_user, int a\_days) | Farm | `[virtual]` |
| SpringPlough(LE \*a\_field, double a\_user, int a\_days) | Farm | `[virtual]` |
| SpringRoll(LE \*a\_field, double a\_user, int a\_days) | Farm | `[virtual]` |
| SpringSow(LE \*a\_field, double a\_user, int a\_days) | Farm | `[virtual]` |
| StrawChopping(LE \*a\_field, double a\_user, int a\_days) | Farm | `[virtual]` |
| Strigling(LE \*a\_field, double a\_user, int a\_days) | Farm | `[virtual]` |
| StriglingSow(LE \*a\_field, double a\_user, int a\_days) | Farm | `[virtual]` |
| StubbleHarrowing(LE \*a\_field, double a\_user, int a\_days) | Farm | `[virtual]` |
| Swathing(LE \*a\_field, double a\_user, int a\_days) | Farm | `[virtual]` |
| SynInsecticideTreat(LE \*a\_field, double a\_user, int a\_days) | Farm | `[virtual]` |
| TranslateCropCodes(const char \*str) | Farm |  |
| Trial\_Control(LE \*a\_field, double a\_user, int a\_days) | Farm | `[virtual]` |
| Trial\_InsecticideTreat(LE \*a\_field, double a\_user, int a\_days) | Farm | `[virtual]` |
| Trial\_ToxicControl(LE \*a\_field, double a\_user, int a\_days) | Farm | `[virtual]` |
| Water(LE \*a\_field, double a\_user, int a\_days) | Farm | `[virtual]` |
| WinterPlough(LE \*a\_field, double a\_user, int a\_days) | Farm | `[virtual]` |
| ~Farm(void) | Farm | `[virtual]` |

---

Generated on Thu Jan 22 14:13:46 2009 for ALMaSS ODDox by 
 1.5.6 
