# Supplementary material for: Predation and fragmentation portrayed in the statistical structure of prey time series
Source: BMC Ecol. 2009 May 6;9:10. doi: 10.1186/1472-6785-9-10 (PMC2689204; doi:10.1186/1472-6785-9-10)
Supplement: Additional file 2 — Voles and related classes ODDox Documentation. ODDox documentation of the agent-based model (ALMaSS) applied by Hendrichsen et al. The documentation is started by activating main.html. [file 1472-6785-9-10-S2.zip › Vole_ODDox/class_organic_plant.html]

ALMaSS ODDox: OrganicPlant Class Reference

- Main Page
- Related Pages
- Classes
- Files

- Alphabetical List
- Class List
- Class Hierarchy
- Class Members

# OrganicPlant Class Reference

`#include <farm.h>`

Inheritance diagram for OrganicPlant:

List of all members.

---

## Detailed Description

A farm that can have its rotation defined by the user at runtime.

|  |
| --- |
|  |
| Public Member Functions | |
| virtual void | MakeStockFarmer (void) |
|  | OrganicPlant (void) |

---

## Constructor & Destructor Documentation

|  |  |  |  |  |  |
| --- | --- | --- | --- | --- | --- |
| OrganicPlant::OrganicPlant | ( | void |  | ) |  |

References Farm::m\_farmtype, Farm::m\_rotation, Farm::m\_stockfarmer, tof\_OrganicPlant, and Farm::TranslateCropCodes().

```
01100                                  : Farm() // 5
01101 {
01102   m_farmtype = tof_OrganicPlant;
01103   m_stockfarmer = false;
01104   FILE * inpfile = fopen("OrganicPlant.rot", "r" );
01105   if (!inpfile) {
01106     g_msg->Warn( WARN_FILE, "OrganicPlant::OrganicPlant():"" Unable to open file ", "OrganicPlant.rot" );
01107     exit( 1 );
01108   }
01109   int nocrops;
01110   fscanf( inpfile, "%d\n", & nocrops );
01111   m_rotation.resize( nocrops );
01112   char cropref[ 255 ];
01113   for ( int i = 0; i < nocrops; i++ ) {
01114     fscanf( inpfile, "%s\n", & cropref );
01115     TTypesOfVegetation tov = TranslateCropCodes( cropref );
01116     m_rotation[ i ] = tov;
01117   }
01118   fclose( inpfile );
01119 }
```

---

## Member Function Documentation

|  |  |  |  |  |  |
| --- | --- | --- | --- | --- | --- |
| virtual void OrganicPlant::MakeStockFarmer | ( | void |  | ) | `[inline, virtual]` |

Reimplemented from Farm.

References Farm::m\_stockfarmer.

```
00649 { m_stockfarmer = false; }
```

---

The documentation for this class was generated from the following files:

- farm.h- farm.cpp

---

Generated on Thu Jan 22 14:13:46 2009 for ALMaSS ODDox by 
 1.5.6 
