# Supplementary material for: Predation and fragmentation portrayed in the statistical structure of prey time series
Source: BMC Ecol. 2009 May 6;9:10. doi: 10.1186/1472-6785-9-10 (PMC2689204; doi:10.1186/1472-6785-9-10)
Supplement: Additional file 2 — Voles and related classes ODDox Documentation. ODDox documentation of the agent-based model (ALMaSS) applied by Hendrichsen et al. The documentation is started by activating main.html. [file 1472-6785-9-10-S2.zip › Vole_ODDox/class_owl.html]

ALMaSS ODDox: Owl Class Reference

- Main Page
- Related Pages
- Classes
- Files

- Alphabetical List
- Class List
- Class Hierarchy
- Class Members

# Owl Class Reference

`#include <Predators.H>`

Inheritance diagram for Owl:

List of all members.

---

## Detailed Description

The Owl class is one of two current implementations of TPredator.

It is configurable via config parameters and in other than name and default configuration it is identical to the Weasel class

|  |
| --- |
|  |
| Public Member Functions | |
| virtual void | BeginStep (void) |
| virtual void | EndStep (void) |
|  | Owl (Vole\_Population\_Manager \*ThePrey, int p\_x, int p\_y, Landscape \*p\_L, TPredator\_Population\_Manager \*p\_PPM) |
| virtual void | Step (void) |
|  | ~Owl () |

---

## Constructor & Destructor Documentation

|  |  |  |  |
| --- | --- | --- | --- |
| Owl::Owl | ( | Vole\_Population\_Manager \* | *ThePrey*, |
|  |  | int | *p\_x*, |
|  |  | int | *p\_y*, |
|  |  | Landscape \* | *p\_L*, |
|  |  | TPredator\_Population\_Manager \* | *p\_PPM* |  |
|  | ) |  |  |  |

References TPredator::m\_DispersalMax, TPredator::m\_HomeRange, TPredator::m\_KillEfficiency, TPredator::m\_NoFailuresBeforeDispersal, TPredator::m\_SearchArea, TPredator::PreyResponse1, TPredator::PreyResponse2, and TPredator::SpeciesID.

```
00447                                           : TPredator(ThePrey,p_x,p_y,p_L,p_PPM)
00448 {
00449     SpeciesID=1;
00450     m_KillEfficiency=250;  // 25%
00451     m_HomeRange=800;
00452     m_SearchArea=50;
00453     m_DispersalMax=1000;
00454     m_NoFailuresBeforeDispersal=200;
00455     PreyResponse1=1;
00456     PreyResponse2=1;
00457 }
```

|  |  |  |  |  |
| --- | --- | --- | --- | --- |
| Owl::~Owl | ( |  | ) |  |

```
00532 {
00533     //Nothing to do
00534 }
```

---

## Member Function Documentation

|  |  |  |  |  |  |
| --- | --- | --- | --- | --- | --- |
| void Owl::BeginStep | ( | void |  | ) | `[virtual]` |

Reimplemented from TPredator.

References TPredator\_Population\_Manager::CreateObjects(), TPredator\_Population\_Manager::dec\_inds(), TAnimal::KillThis(), struct\_Predator::L, TPredator::m\_kills\_this\_season, TAnimal::m\_Location\_x, TAnimal::m\_Location\_y, TAnimal::m\_OurLandscape, TPredator::m\_OurPopulationManager, OWL, owl\_breed\_day, owl\_breed\_threshold, owl\_death\_threshold, struct\_Predator::PM, TPredator\_Population\_Manager::supply\_no\_inds(), struct\_Predator::x, and struct\_Predator::y.

```
00461 {
00462   int day= m_OurLandscape->SupplyDayInYear();
00463   if (day==owl_breed_day)
00464   {
00465     int noToMake=m_kills_this_season/owl_breed_threshold;
00466     for (int k=0; k<noToMake; k++)
00467     {
00468       // make a new owl
00469       struct_Predator* sp;
00470       sp = new struct_Predator;
00471       sp->PM = m_OurPopulationManager;
00472       sp->L = m_OurLandscape;
00473       sp->x = m_Location_x;
00474       sp->y = m_Location_y;
00475       m_OurPopulationManager->CreateObjects(OWL,NULL,sp,1); // 1 = owl
00476       delete sp;
00477     }
00478   }
00479   else if (day==364)
00480   {
00481     if (m_kills_this_season<owl_death_threshold)
00482     {
00483       if (m_OurPopulationManager->supply_no_inds(OWL)>1)
00484       {
00485         m_OurPopulationManager->dec_inds(OWL);
00486         KillThis();
00487       }
00488     }
00489     m_kills_this_season=0; // reset the count
00490   }
00491 }
```

|  |  |  |  |  |  |
| --- | --- | --- | --- | --- | --- |
| virtual void Owl::EndStep | ( | void |  | ) | `[inline, virtual]` |

Reimplemented from TPredator.

```
00192 {}
```

|  |  |  |  |  |  |
| --- | --- | --- | --- | --- | --- |
| void Owl::Step | ( | void |  | ) | `[virtual]` |

Reimplemented from TPredator.

References TPredator::CurrentPState, TALMaSSObject::CurrentStateNo, TPredator::m\_FailureCount, TPredator::m\_HaveTerritory, TPredator::m\_NoFailuresBeforeDispersal, TPredator::PreyResponse1, TPredator::PreyResponse2, TPredator::st\_Dispersal(), TPredator::st\_Hunting(), TPredator::st\_Movement(), TALMaSSObject::StepDone, tops\_Dispersal, tops\_Hunting, tops\_InitialState, and tops\_Movement.

```
00495 {
00496   if (StepDone || CurrentStateNo == -1) return;
00497   switch (CurrentPState)
00498   {
00499    case tops_InitialState: // Initial state
00500     CurrentPState=tops_Dispersal;
00501     m_HaveTerritory=false;
00502     break;
00503    case tops_Hunting:
00504     if (st_Hunting()<PreyResponse1) CurrentPState=tops_Movement;
00505     StepDone=true;
00506     break;
00507    case tops_Dispersal:
00508     st_Dispersal();
00509     CurrentPState=tops_Hunting;
00510     StepDone=true;
00511     break;
00512    case tops_Movement:
00513     st_Movement();
00514     if (st_Hunting()<PreyResponse2) // alter this figure to increase functional response
00515      m_FailureCount++;
00516     else m_FailureCount=0;
00517     if (m_FailureCount>m_NoFailuresBeforeDispersal)
00518     {
00519       m_HaveTerritory=false;
00520       CurrentPState=tops_Dispersal;
00521     }
00522     else CurrentPState=tops_Hunting;
00523     StepDone=true;
00524     break;
00525    default:
00526     exit(1);
00527    }
00528 }
```

---

The documentation for this class was generated from the following files:

- Predators.H- Predators.cpp

---

Generated on Thu Jan 22 14:13:46 2009 for ALMaSS ODDox by 
 1.5.6 
