# Supplementary material for: Predation and fragmentation portrayed in the statistical structure of prey time series
Source: BMC Ecol. 2009 May 6;9:10. doi: 10.1186/1472-6785-9-10 (PMC2689204; doi:10.1186/1472-6785-9-10)
Supplement: Additional file 2 — Voles and related classes ODDox Documentation. ODDox documentation of the agent-based model (ALMaSS) applied by Hendrichsen et al. The documentation is started by activating main.html. [file 1472-6785-9-10-S2.zip › Vole_ODDox/class_pesticide_trial_control.html]

ALMaSS ODDox: PesticideTrialControl Class Reference

- Main Page
- Related Pages
- Classes
- Files

- Alphabetical List
- Class List
- Class Hierarchy
- Class Members

# PesticideTrialControl Class Reference

`#include <farm.h>`

Inheritance diagram for PesticideTrialControl:

List of all members.

---

## Detailed Description

Inbuilt special purpose farm type.

|  |
| --- |
|  |
| Public Member Functions | |
| virtual void | MakeStockFarmer (void) |
|  | PesticideTrialControl (void) |

---

## Constructor & Destructor Documentation

|  |  |  |  |  |  |
| --- | --- | --- | --- | --- | --- |
| PesticideTrialControl::PesticideTrialControl | ( | void |  | ) |  |

References Farm::m\_farmtype, Farm::m\_rotation, Farm::m\_stockfarmer, tof\_PTrialControl, and tov\_WWheatPControl.

```
01133                                                    : Farm() // 6
01134 {
01135   m_farmtype = tof_PTrialControl;
01136   m_stockfarmer = false;
01137   m_rotation.resize( 1 );
01138   m_rotation[ 0 ] = tov_WWheatPControl;
01139 }
```

---

## Member Function Documentation

|  |  |  |  |  |  |
| --- | --- | --- | --- | --- | --- |
| virtual void PesticideTrialControl::MakeStockFarmer | ( | void |  | ) | `[inline, virtual]` |

Reimplemented from Farm.

References Farm::m\_stockfarmer.

```
00686 { m_stockfarmer = false; }
```

---

The documentation for this class was generated from the following files:

- farm.h- farm.cpp

---

Generated on Thu Jan 22 14:13:46 2009 for ALMaSS ODDox by 
 1.5.6 
