# Supplementary material for: Predation and fragmentation portrayed in the statistical structure of prey time series
Source: BMC Ecol. 2009 May 6;9:10. doi: 10.1186/1472-6785-9-10 (PMC2689204; doi:10.1186/1472-6785-9-10)
Supplement: Additional file 2 — Voles and related classes ODDox Documentation. ODDox documentation of the agent-based model (ALMaSS) applied by Hendrichsen et al. The documentation is started by activating main.html. [file 1472-6785-9-10-S2.zip › Vole_ODDox/class_pesticide_trial_treatment.html]

ALMaSS ODDox: PesticideTrialTreatment Class Reference

- Main Page
- Related Pages
- Classes
- Files

- Alphabetical List
- Class List
- Class Hierarchy
- Class Members

# PesticideTrialTreatment Class Reference

`#include <farm.h>`

Inheritance diagram for PesticideTrialTreatment:

List of all members.

---

## Detailed Description

Inbuilt special purpose farm type.

|  |
| --- |
|  |
| Public Member Functions | |
| virtual void | MakeStockFarmer (void) |
|  | PesticideTrialTreatment (void) |

---

## Constructor & Destructor Documentation

|  |  |  |  |  |  |
| --- | --- | --- | --- | --- | --- |
| PesticideTrialTreatment::PesticideTrialTreatment | ( | void |  | ) |  |

References Farm::m\_farmtype, Farm::m\_rotation, Farm::m\_stockfarmer, tof\_PTrialTreatment, and Farm::TranslateCropCodes().

```
01149                                                        : Farm() // 8
01150 {
01151   m_farmtype = tof_PTrialTreatment;
01152   m_stockfarmer = false;
01153   // This farm type reads its rotation from a special file PesticideTrialTreatment.rot
01154   FILE * inpfile = fopen("PesticideTrialTreatment.rot", "r" );
01155   if (!inpfile) {
01156     g_msg->Warn( WARN_FILE, "PesticideTrialTreatment::PesticideTrialTreatment():"" Unable to open file ",
01157          "PesticideTrialTreatment.rot" );
01158     exit( 1 );
01159   }
01160   int nocrops;
01161   fscanf( inpfile, "%d\n", & nocrops );
01162   m_rotation.resize( nocrops );
01163   char cropref[ 255 ];
01164   for ( int i = 0; i < nocrops; i++ ) {
01165     fscanf( inpfile, "%s\n", & cropref );
01166     TTypesOfVegetation tov = TranslateCropCodes( cropref );
01167     m_rotation[ i ] = tov;
01168   }
01169   /* m_rotation.resize( 9 ); m_rotation[ 0] = tov_WinterRape; m_rotation[ 1] = tov_WWheatPTreatment;
01170   m_rotation[ 2] = tov_SpringBarley; m_rotation[ 3] = tov_SpringBarley; m_rotation[ 4] = tov_Setaside;
01171   m_rotation[ 5] = tov_FieldPeas; m_rotation[ 6] = tov_WWheatPTreatment; m_rotation[ 7] = tov_WWheatPTreatment;
01172   m_rotation[ 8] = tov_WWheatPTreatment; */
01173   fclose( inpfile );
01174 }
```

---

## Member Function Documentation

|  |  |  |  |  |  |
| --- | --- | --- | --- | --- | --- |
| virtual void PesticideTrialTreatment::MakeStockFarmer | ( | void |  | ) | `[inline, virtual]` |

Reimplemented from Farm.

References Farm::m\_stockfarmer.

```
00706 { m_stockfarmer = false; }
```

---

The documentation for this class was generated from the following files:

- farm.h- farm.cpp

---

Generated on Thu Jan 22 14:13:46 2009 for ALMaSS ODDox by 
 1.5.6 
