# Supplementary material for: Predation and fragmentation portrayed in the statistical structure of prey time series
Source: BMC Ecol. 2009 May 6;9:10. doi: 10.1186/1472-6785-9-10 (PMC2689204; doi:10.1186/1472-6785-9-10)
Supplement: Additional file 2 — Voles and related classes ODDox Documentation. ODDox documentation of the agent-based model (ALMaSS) applied by Hendrichsen et al. The documentation is started by activating main.html. [file 1472-6785-9-10-S2.zip › Vole_ODDox/class_population___manager.html]

ALMaSS ODDox: Population\_Manager Class Reference

- Main Page
- Related Pages
- Classes
- Files

- Alphabetical List
- Class List
- Class Hierarchy
- Class Members

# Population\_Manager Class Reference

`#include <PopulationManager.h>`

Inheritance diagram for Population\_Manager:

List of all members.

---

## Detailed Description

Base class for all population managers.

The core of the handling of animal populations. All time-step code and most input/output is handled by this class and its descendents. This class effectively implements a state machine to facilitate simulation of animal behaviours and handle potential issues with concurrency. The PopulationManager class is never instantiated but must be used by deriving a descendent class.

|  |
| --- |
|  |
| Public Member Functions | |
| bool | BeginningOfMonth () |
| virtual void | BreedingPairsOutput (int, int, int) |
| virtual void | BreedingSuccessProbeOutput (double, int, int, int, int, int, int, int) |
| virtual void | Catastrophe (int) |
| void | CloseTheCIPEGridOutputProbe () |
| void | CloseTheMonthlyRipleysOutputProbe () |
| void | CloseTheReallyBigOutputProbe () |
| void | CloseTheRipleysOutputProbe () |
| virtual void | DisplayLocations () |
| TAnimal \* | FindClosest (int x, int y, unsigned Type) |
| virtual void | FledgelingProbeOutput (int, int) |
| virtual void | GeneticsResultsOutput (FILE \*, unsigned) |
| virtual void | ImpactedProbe () |
| void | ImpactProbeReport (int a\_Time) |
| bool | IsLast (unsigned listindex) |
| void | LamdaBirth (int x, int y, int z) |
| void | LamdaBirth (int x, int y) |
| void | LamdaClear () |
| void | LamdaDeath (int x, int y) |
| void | LamdaDumpOutput () |
| void | LOG (const char \*fname) |
| virtual bool | OpenTheBreedingPairsProbe () |
| virtual bool | OpenTheBreedingSuccessProbe () |
| bool | OpenTheCIPEGridOutputProbe () |
| virtual bool | OpenTheFledgelingProbe () |
| bool | OpenTheMonthlyRipleysOutputProbe () |
| bool | OpenTheReallyBigProbe () |
| bool | OpenTheRipleysOutputProbe () |
|  | Population\_Manager (Landscape \*L) |
| virtual float | Probe (int ListIndex, probe\_data \*p\_TheProbe) |
| int | ProbeFileInput (char \*p\_Filename, int p\_ProbeNo) |
| char \* | ProbeReport (int a\_time) |
| virtual void | Run (int NoTSteps) |
| void | SetNoProbes (int a\_pn) |
| char \* | SpeciesSpecificReporting (int a\_species, int a\_time) |
| virtual int | SupplyCovPosx (int) |
| virtual int | SupplyCovPosy (int) |
| unsigned | SupplyListIndexSize () |
| AnsiString | SupplyListName (int i) |
| int | SupplyListNameLength () |
| unsigned | SupplyListSize (unsigned listindex) |
| virtual void | SupplyLocXY (unsigned listindex, unsigned j, int &x, int &y) |
| virtual int | SupplyPegPosx (int) |
| virtual int | SupplyPegPosy (int) |
| int | SupplySimH () |
| int | SupplySimW () |
| int | SupplyState (unsigned listindex, unsigned j) |
| IntArray100 \* | SupplyStateList () |
| AnsiString | SupplyStateNames (int i) |
| unsigned | SupplyStateNamesLength () |
| int | SupplyStepSize () |
| virtual int | TheBreedingFemalesProbe (int) |
| virtual int | TheBreedingSuccessProbe (int &, int &, int &, int &, int &, int &) |
| virtual void | TheCIPEGridOutputProbe () |
| virtual void | TheCIPEGridOutputProbeB () |
| virtual int | TheFledgelingProbe () |
| virtual void | TheGeneticProbe (unsigned, int, unsigned &) |
| virtual void | TheReallyBigOutputProbe () |
| virtual void | TheRipleysOutputProbe (FILE \*a\_prb) |
| void | WriteToTest2File (char \*n, int n2) |
| void | WriteToTest2File (int n, int n2) |
| void | WriteToTestFile (char \*n, int n2) |
| void | WriteToTestFile (int n, int n2) |
| virtual | ~Population\_Manager (void) |
| Public Attributes | |
| AlleleFreq \* | AlFreq |
| unsigned | Counts [10][100] |
| int | IndexArrayX [5][10000] |
| MyDialog \* | m\_MainForm |
| char | m\_SimulationName [255] |
| Landscape \* | m\_TheLandscape |
| bool | ProbesSet |
| int | SimH |
| int | SimW |
| probe\_data \* | TheProbe [100] |
| Protected Member Functions | |
| virtual void | Catastrophe () |
| virtual void | DoAfter () |
| virtual void | DoAlmostLast () |
| virtual void | DoBefore () |
| virtual void | DoFirst () |
| virtual void | DoLast () |
| void | Shuffle (unsigned Type) |
| void | Shuffle\_or\_Sort (unsigned Type) |
| void | SortState (unsigned Type) |
| void | SortX (unsigned Type) |
| void | SortXIndex (unsigned Type) |
| void | SortY (unsigned Type) |
| virtual bool | StepFinished () |
| Protected Attributes | |
| unsigned | BeforeStepActions [10] |
| FILE \* | CIPEGridOutputPrb |
| FILE \* | CIPEGridOutputPrbB |
| double | gridcount [25][25] |
| long int | lamdagrid [2][257][257] |
| unsigned | ListNameLength |
| AnsiString | ListNames [10] |
| FILE \* | m\_AlleleFreqsFile |
| int | m\_catastrophestartyear |
| FILE \* | m\_EasyPopRes |
| FILE \* | m\_GeneticsFile |
| int | m\_NoProbes |
| int | m\_StepSize |
| FILE \* | ReallyBigOutputPrb |
| FILE \* | RipleysOutputPrb |
| FILE \* | RipleysOutputPrb1 |
| FILE \* | RipleysOutputPrb10 |
| FILE \* | RipleysOutputPrb11 |
| FILE \* | RipleysOutputPrb12 |
| FILE \* | RipleysOutputPrb2 |
| FILE \* | RipleysOutputPrb3 |
| FILE \* | RipleysOutputPrb4 |
| FILE \* | RipleysOutputPrb5 |
| FILE \* | RipleysOutputPrb6 |
| FILE \* | RipleysOutputPrb7 |
| FILE \* | RipleysOutputPrb8 |
| FILE \* | RipleysOutputPrb9 |
| IntArray100 | StateList |
| AnsiString | StateNames [100] |
| unsigned | StateNamesLength |
| FILE \* | TestFile |
| FILE \* | TestFile2 |
| vector< TListOfAnimals > | TheArray |

---

## Constructor & Destructor Documentation

|  |  |  |  |  |  |
| --- | --- | --- | --- | --- | --- |
| Population\_Manager::Population\_Manager | ( | Landscape \* | *L* | ) |  |

Constructor for the Population\_Manager class

References BeforeStepActions, cfg\_RipleysOutputMonthly\_used(), LamdaClear(), m\_catastrophestartyear, m\_TheLandscape, OpenTheMonthlyRipleysOutputProbe(), SimH, SimW, StateNamesLength, and TheArray.

```
00160                                                       {
00161         // Keep a pointer to the landscape this population is in        
00162         m_TheLandscape = L;
00163         // create 10 empty arrays as default, excess can be removed in descendent classes
00164         TListOfAnimals alist;
00165         TheArray.insert( TheArray.end(), 10, alist );
00166         // Set the simulation bounds
00167         SimH = m_TheLandscape->SupplySimAreaHeight();
00168         SimW = m_TheLandscape->SupplySimAreaWidth();
00169         // Set default BeforeStepActions
00170         for ( int i = 0; i < 10; i++ ) BeforeStepActions[ i ] = 0;
00171         // modify this if they need to in descendent classes
00172         StateNamesLength = 0; // initialise this variable.
00173         m_catastrophestartyear=-1; // Default is don't do this
00174 #ifdef __LAMBDA_RECORD
00175         ofstream fout("LambdaGridOuput.txt", ios_base::out);  // open for writing
00176         fout.close();
00177         LamdaClear();
00178 #endif
00179   if ( cfg_RipleysOutputMonthly_used.value() ) {
00180         OpenTheMonthlyRipleysOutputProbe();
00181   }
00182 
00183 }
```

|  |  |  |  |  |  |
| --- | --- | --- | --- | --- | --- |
| Population\_Manager::~Population\_Manager | ( | void |  | ) | `[virtual]` |

Destructor for the Population\_Manager class

References cfg\_ReallyBigOutput\_used, cfg\_RipleysOutput\_used, cfg\_RipleysOutputMonthly\_used(), CloseTheMonthlyRipleysOutputProbe(), CloseTheReallyBigOutputProbe(), CloseTheRipleysOutputProbe(), and TheArray.

```
00190                                               {
00191   // clean-up
00192   for ( unsigned i = 0; i < TheArray.size(); i++ ) {
00193     // if objects in the list need to be deleted:
00194     for ( unsigned j = 0; j < TheArray[ i ].size(); j++ ) {
00195       delete TheArray[ i ] [ j ];
00196     }
00197     // empty the array
00198     TheArray[ i ].clear();
00199     // --
00200   }
00201   if ( cfg_RipleysOutput_used.value() ) {
00202     CloseTheRipleysOutputProbe();
00203   }
00204   if ( cfg_RipleysOutputMonthly_used.value() ) {
00205         CloseTheMonthlyRipleysOutputProbe();
00206   }
00207   if ( cfg_ReallyBigOutput_used.value() ) {
00208     CloseTheReallyBigOutputProbe();
00209   }
00210 }
```

---

## Member Function Documentation

|  |  |  |  |  |
| --- | --- | --- | --- | --- |
| bool Population\_Manager::BeginningOfMonth | ( |  | ) |  |

Is it the first day of the month?

References cfg\_DayInMonth(), and m\_TheLandscape.

Referenced by ImpactProbeReport(), and ProbeReport().

```
01015                                           {
01016   if ( m_TheLandscape->SupplyDayInMonth() == cfg_DayInMonth.value() ) return true;
01017   return false;
01018 }
```

|  |  |  |  |
| --- | --- | --- | --- |
| virtual void Population\_Manager::BreedingPairsOutput | ( | int | , |
|  |  | int | , |
|  |  | int |  |  |
|  | ) |  |  | `[inline, virtual]` |

```
00427                                                        {
00428   }
```

|  |  |  |  |
| --- | --- | --- | --- |
| virtual void Population\_Manager::BreedingSuccessProbeOutput | ( | double | , |
|  |  | int | , |
|  |  | int | , |
|  |  | int | , |
|  |  | int | , |
|  |  | int | , |
|  |  | int | , |
|  |  | int |  |  |
|  | ) |  |  | `[inline, virtual]` |

Referenced by SpeciesSpecificReporting().

```
00435                                                                                         {
00436   }
```

|  |  |  |  |  |  |
| --- | --- | --- | --- | --- | --- |
| void Population\_Manager::Catastrophe | ( | void |  | ) | `[protected, virtual]` |

This method MUST be overidden in descendent classes if this functionality is does not match with the animals requirements

Reimplemented in Vole\_Population\_Manager.

Referenced by Run().

```
01022                                      {
01027         return;
01028 }
```

|  |  |  |  |  |  |
| --- | --- | --- | --- | --- | --- |
| virtual void Population\_Manager::Catastrophe | ( | int |  | ) | `[inline, virtual]` |

```
00237                                                      {
00238         }
```

|  |  |  |  |  |
| --- | --- | --- | --- | --- |
| void Population\_Manager::CloseTheCIPEGridOutputProbe | ( |  | ) |  |

close the probe

References CIPEGridOutputPrb, and CIPEGridOutputPrbB.

```
00709                                                      {
00710   if ( CIPEGridOutputPrb != NULL )
00711     fclose( CIPEGridOutputPrb );
00712     fclose( CIPEGridOutputPrbB );
00713 }
```

|  |  |  |  |  |
| --- | --- | --- | --- | --- |
| void Population\_Manager::CloseTheMonthlyRipleysOutputProbe | ( |  | ) |  |

close the monthly probes

References RipleysOutputPrb1, RipleysOutputPrb10, RipleysOutputPrb11, RipleysOutputPrb12, RipleysOutputPrb2, RipleysOutputPrb3, RipleysOutputPrb4, RipleysOutputPrb5, RipleysOutputPrb6, RipleysOutputPrb7, RipleysOutputPrb8, and RipleysOutputPrb9.

Referenced by ~Population\_Manager().

```
00847                                                            {
00848   fclose( RipleysOutputPrb1 );
00849   fclose( RipleysOutputPrb2 );
00850   fclose( RipleysOutputPrb3 );
00851   fclose( RipleysOutputPrb4 );
00852   fclose( RipleysOutputPrb5 );
00853   fclose( RipleysOutputPrb6 );
00854   fclose( RipleysOutputPrb7 );
00855   fclose( RipleysOutputPrb8 );
00856   fclose( RipleysOutputPrb9 );
00857   fclose( RipleysOutputPrb10 );
00858   fclose( RipleysOutputPrb11 );
00859   fclose( RipleysOutputPrb12 );
00860 }
```

|  |  |  |  |  |
| --- | --- | --- | --- | --- |
| void Population\_Manager::CloseTheReallyBigOutputProbe | ( |  | ) |  |

close the probe

Reimplemented in TPredator\_Population\_Manager.

References ReallyBigOutputPrb.

Referenced by ~Population\_Manager().

```
00866                                                       {
00867   if ( ReallyBigOutputPrb != 0 )
00868     fclose( ReallyBigOutputPrb );
00869   ReallyBigOutputPrb=0;
00870 }
```

|  |  |  |  |  |
| --- | --- | --- | --- | --- |
| void Population\_Manager::CloseTheRipleysOutputProbe | ( |  | ) |  |

close the probe

Reimplemented in TPredator\_Population\_Manager.

References RipleysOutputPrb.

Referenced by ~Population\_Manager().

```
00836                                                     {
00837   if ( RipleysOutputPrb != 0 )
00838     fclose( RipleysOutputPrb );
00839   RipleysOutputPrb=0;
00840 }
```

|  |  |  |  |  |
| --- | --- | --- | --- | --- |
| void Population\_Manager::DisplayLocations | ( |  | ) | `[virtual]` |

Not currently used

```
00480                                           {
00481   /*  for (unsigned j=0; j<TheArray[listindex].size(); j++) { int x=TheArray[listindex][j]->Supply_m_Location_x();
00482   int y=TheArray[listindex][j]->Supply_m_Location_y(); m_MainForm->Spot(listindex,x,y); }
00483 
00484   /*        // Skylark below if (m_MainForm->RadioButton2->Checked)  {
00485   switch ((TTypesOfSkState)TheArray[listindex][j]->WhatState()) { case toss_MFloating: case toss_MFlocking:
00486   case toss_FFloating: case toss_FFlocking: break; default: int x=TheArray[listindex][j]->Supply_m_Location_x();
00487   int y=TheArray[listindex][j]->Supply_m_Location_y(); m_MainForm->Spot(listindex,x,y); break; } }
00488 
00489   */
00490   /*   Partridge This code will only show coveys with chicks switch ((Partridge_State)AManager->SupplyState(listindex,j)) {
00491   case pars_MCaringForYoung: case pars_FCaringForYoung: AManager->SupplyLocXY(listindex,j,x,y); Form6->Spot(listindex,x,y);
00492   break; default: break; } */
00493   /* This code shows all covey pegs & positions if (listindex==4) // Coveys { int x = SupplyPegPosx(j);
00494   int y = SupplyPegPosy(j); m_MainForm->Spot(1,x,y); x = SupplyCovPosx(j); y = SupplyCovPosy(j); m_MainForm->Spot(2,x,y); }
00495   #else int x=TheArray[listindex][j]->Supply_m_Location_x(); int y=TheArray[listindex][j]->Supply_m_Location_y();
00496   m_MainForm->Spot(listindex,x,y); */
00497 }
```

|  |  |  |  |  |
| --- | --- | --- | --- | --- |
| void Population\_Manager::DoAfter | ( |  | ) | `[protected, virtual]` |

Can be used in descendent classes

Reimplemented in TPredator\_Population\_Manager.

Referenced by Run().

```
00431                                  {
00432   //TODO: Add your source code here
00433 }
```

|  |  |  |  |  |
| --- | --- | --- | --- | --- |
| void Population\_Manager::DoAlmostLast | ( |  | ) | `[protected, virtual]` |

Can be used in descendent classes

Referenced by Run().

```
00440                                       {
00441   //TODO: Add your source code here
00442 }
```

|  |  |  |  |  |
| --- | --- | --- | --- | --- |
| void Population\_Manager::DoBefore | ( |  | ) | `[protected, virtual]` |

Can be used in descendent classes

Reimplemented in TPredator\_Population\_Manager.

Referenced by Run().

```
00251                                   {
00252 }
```

|  |  |  |  |  |
| --- | --- | --- | --- | --- |
| void Population\_Manager::DoFirst | ( |  | ) | `[protected, virtual]` |

Can be used in descendent classes

Reimplemented in TPredator\_Population\_Manager, and Vole\_Population\_Manager.

Referenced by Run().

```
00243                                  {
00244 }
```

|  |  |  |  |  |
| --- | --- | --- | --- | --- |
| void Population\_Manager::DoLast | ( |  | ) | `[protected, virtual]` |

Collects some data to describe the number of animals in each state at the end of the day

Reimplemented in TPredator\_Population\_Manager.

References Counts, ListNameLength, IntArray100::n, StateList, and TheArray.

Referenced by Run().

```
00448                                 {
00449 
00450 #ifdef __UNIX__
00451   for ( unsigned i = 0; i < 100; i++ ) {
00452     StateList.n[ i ] = 0;
00453   }
00454 #else
00455   // Zero results
00456   for ( unsigned listindex = 0; listindex < ListNameLength; listindex++ ) {
00457     for ( unsigned i = 0; i < 100; i++ ) {
00458       Counts[ listindex ] [ i ] = 0;
00459     }
00460   }
00461 #endif
00462   // How many animals in each state?
00463   for ( unsigned listindex = 0; listindex < TheArray.size(); listindex++ ) {
00464     unsigned size = (unsigned) TheArray[ listindex ].size();
00465     for ( unsigned j = 0; j < size; j++ ) {
00466 #ifdef __UNIX__
00467       StateList.n[ TheArray[ listindex ] [ j ]->WhatState() ] ++;
00468 #else
00469       Counts[ listindex ] [ TheArray[ listindex ] [ j ]->WhatState() ] ++;
00470 #endif
00471     }
00472   }
00473 }
```

|  |  |  |  |
| --- | --- | --- | --- |
| TAnimal \* Population\_Manager::FindClosest | ( | int | *x*, |
|  |  | int | *y*, |
|  |  | unsigned | *Type* |  |
|  | ) |  |  |  |

Finds the closest individual to an x,y point

References TheArray.

```
00893                                                                        {
00894   int distance = 100000000;
00895   TAnimal * TA = NULL;
00896   int dx, dy, d;
00897   for ( unsigned j = 0; j < TheArray[ Type ].size(); j++ ) {
00898     dx = TheArray[ Type ] [ j ]->Supply_m_Location_x();
00899     dy = TheArray[ Type ] [ j ]->Supply_m_Location_y();
00900     dx = ( dx - x );
00901     dx *= dx;
00902     dy = ( dy - y );
00903 
00904     dy *= dy;
00905     d = dx + dy;
00906     if ( d < distance ) {
00907       distance = d;
00908       TA = TheArray[ Type ] [ j ];
00909     }
00910   }
00911   return TA;
00912 }
```

|  |  |  |  |
| --- | --- | --- | --- |
| virtual void Population\_Manager::FledgelingProbeOutput | ( | int | , |
|  |  | int |  |  |
|  | ) |  |  | `[inline, virtual]` |

Referenced by SpeciesSpecificReporting().

```
00440                                                  {
00441   }
```

|  |  |  |  |
| --- | --- | --- | --- |
| virtual void Population\_Manager::GeneticsResultsOutput | ( | FILE \* | , |
|  |  | unsigned |  |  |
|  | ) |  |  | `[inline, virtual]` |

Reimplemented in Vole\_Population\_Manager.

Referenced by SpeciesSpecificReporting().

```
00444                                                            {
00445   }
```

|  |  |  |  |  |
| --- | --- | --- | --- | --- |
| void Population\_Manager::ImpactedProbe | ( |  | ) | `[virtual]` |

Special pesticide related probe. Overidden in descendent classes

Reimplemented in Vole\_Population\_Manager.

Referenced by ImpactProbeReport().

```
00602                                         {
00603         
00604 }
```

|  |  |  |  |  |  |
| --- | --- | --- | --- | --- | --- |
| void Population\_Manager::ImpactProbeReport | ( | int | *a\_Time* | ) |  |

Special probe

References BeginningOfMonth(), ImpactedProbe(), and TheProbe.

Referenced by SpeciesSpecificReporting().

```
01196                                                        {
01197 
01198   for ( int ProbeNo = 0; ProbeNo < 1; ProbeNo++ ) {
01199     // See if we need to record/update this one
01200     // if time/months/years ==0 or every time
01201     if ( ( TheProbe[ ProbeNo ]->m_ReportInterval == 3 )
01202          || ( ( TheProbe[ ProbeNo ]->m_ReportInterval == 2 ) && ( BeginningOfMonth() ) )
01203                  || ( ( TheProbe[ ProbeNo ]->m_ReportInterval == 1 ) && ( a_Time % 365 == 0 ) ) ) {
01204              ImpactedProbe();
01205     }
01206   }
01207 }
```

|  |  |  |  |  |  |
| --- | --- | --- | --- | --- | --- |
| bool Population\_Manager::IsLast | ( | unsigned | *listindex* | ) | `[inline]` |

References TheArray.

```
00282                                     {
00283     if ( TheArray[ listindex ].size() > 1 ) return false; else
00284       return true;
00285   };
```

|  |  |  |  |
| --- | --- | --- | --- |
| void Population\_Manager::LamdaBirth | ( | int | *x*, |
|  |  | int | *y*, |
|  |  | int | *z* |  |
|  | ) |  |  | `[inline]` |

References \_\_lgridsize, and lamdagrid.

```
00392                                             {
00393                lamdagrid[0][x / __lgridsize][y / __lgridsize ]+=z;
00394        }
```

|  |  |  |  |
| --- | --- | --- | --- |
| void Population\_Manager::LamdaBirth | ( | int | *x*, |
|  |  | int | *y* |  |
|  | ) |  |  | `[inline]` |

References \_\_lgridsize, and lamdagrid.

Referenced by Vole\_Female::st\_GiveBirth().

```
00389                                      {
00390                lamdagrid[0][x / __lgridsize][y / __lgridsize ]++;
00391        }
```

|  |  |  |  |  |
| --- | --- | --- | --- | --- |
| void Population\_Manager::LamdaClear | ( |  | ) | `[inline]` |

References lamdagrid.

Referenced by Vole\_Population\_Manager::DoFirst(), and Population\_Manager().

```
00395                          {
00396                for (int i=0; i<257; i++ ) {
00397                        for (int j=0; j<257; j++) {
00398                                lamdagrid[0][i][j]=0;
00399                                lamdagrid[1][i][j]=0;
00400                        }
00401                }
00402        }
```

|  |  |  |  |
| --- | --- | --- | --- |
| void Population\_Manager::LamdaDeath | ( | int | *x*, |
|  |  | int | *y* |  |
|  | ) |  |  | `[inline]` |

References \_\_lgridsize, and lamdagrid.

Referenced by Vole\_Base::st\_Dying().

```
00385                                      {
00386                // inlined for speed
00387                lamdagrid[1][x / __lgridsize][y / __lgridsize ]++;
00388        }
```

|  |  |  |  |  |
| --- | --- | --- | --- | --- |
| void Population\_Manager::LamdaDumpOutput | ( |  | ) |  |

Special probe

References lamdagrid.

Referenced by Vole\_Population\_Manager::DoFirst().

```
00587                                          {
00588    ofstream fout("LambdaGridOuput.txt", ios_base::app);  // open for writing
00589    for (int i=0; i<257; i++ ) {
00590            for (int j=0; j<257; j++) {
00591                    fout << lamdagrid[0][i][j] << "\t" << lamdagrid[1][i][j] << endl;
00592            }
00593    }
00594    //fout << "NEXT" << endl;
00595    fout.close();
00596 }
```

|  |  |  |  |  |  |
| --- | --- | --- | --- | --- | --- |
| void Population\_Manager::LOG | ( | const char \* | *fname* | ) |  |

Debug function used to log whatever is needed - this is just a place to write whatever is needed at the time - so contents vary

References ListNames, m\_TheLandscape, AnimalPosition::m\_x, AnimalPosition::m\_y, and TheArray.

```
00217                                                 {
00218   FILE * PFile = fopen(fname, "w" );
00219   if (PFile) {
00220           m_TheLandscape->Warn("PopulationManager::LOG - Could not open file ",fname);
00221           exit(0);
00222   }
00223   AnimalPosition AP;
00224   for ( unsigned listindex = 0; listindex < TheArray.size(); listindex++ ) {
00225 #ifndef __BCB__
00226     fprintf( PFile, "%s :\n", ListNames[ listindex ] );
00227 #else
00228     fprintf( PFile, "%s :\n", ListNames[ listindex ].c_str() );
00229 #endif
00230     for ( unsigned j = 0; j < TheArray[ listindex ].size(); j++ ) {
00231       AP = TheArray[ listindex ] [ j ]->SupplyPosition();
00232       fprintf( PFile, "%i %i %i\n", j, AP.m_x, AP.m_y );
00233     }
00234   }
00235   fclose( PFile );
00236 }
```

|  |  |  |  |  |
| --- | --- | --- | --- | --- |
| virtual bool Population\_Manager::OpenTheBreedingPairsProbe | ( |  | ) | `[inline, virtual]` |

```
00421                                            {
00422     return false;
00423   }
```

|  |  |  |  |  |
| --- | --- | --- | --- | --- |
| virtual bool Population\_Manager::OpenTheBreedingSuccessProbe | ( |  | ) | `[inline, virtual]` |

```
00424                                              {
00425     return false;
00426   }
```

|  |  |  |  |  |
| --- | --- | --- | --- | --- |
| bool Population\_Manager::OpenTheCIPEGridOutputProbe | ( |  | ) |  |

open the probe

References cfg\_CIPEGridOutput\_filename(), cfg\_CIPEGridOutput\_filenameB(), CIPEGridOutputPrb, and CIPEGridOutputPrbB.

Referenced by Vole\_Population\_Manager::Init().

```
00688                                                     {
00689   CIPEGridOutputPrb = fopen(cfg_CIPEGridOutput_filename.value(), "w" );
00690   if ( !CIPEGridOutputPrb ) {
00691     g_msg->Warn( WARN_FILE, "Population_Manager::OpenTheCIPEGridOutputProbe(): ""Unable to open probe file",
00692          cfg_CIPEGridOutput_filename.value() );
00693     exit( 1 );
00694   }
00695   CIPEGridOutputPrbB = fopen(cfg_CIPEGridOutput_filenameB.value(), "w" );
00696   if ( !CIPEGridOutputPrbB ) {
00697     g_msg->Warn( WARN_FILE, "Population_Manager::OpenTheCIPEGridOutputProbe(): ""Unable to open probe file",
00698          cfg_CIPEGridOutput_filenameB.value() );
00699     exit( 1 );
00700   }
00701   return true;
00702 }
```

|  |  |  |  |  |
| --- | --- | --- | --- | --- |
| virtual bool Population\_Manager::OpenTheFledgelingProbe | ( |  | ) | `[inline, virtual]` |

```
00418                                         {
00419     return false;
00420   }
```

|  |  |  |  |  |
| --- | --- | --- | --- | --- |
| bool Population\_Manager::OpenTheMonthlyRipleysOutputProbe | ( |  | ) |  |

open 12 ripley output probles, one for each month

References cfg\_RipleysOutput\_filename(), RipleysOutputPrb1, RipleysOutputPrb10, RipleysOutputPrb11, RipleysOutputPrb12, RipleysOutputPrb2, RipleysOutputPrb3, RipleysOutputPrb4, RipleysOutputPrb5, RipleysOutputPrb6, RipleysOutputPrb7, RipleysOutputPrb8, and RipleysOutputPrb9.

Referenced by Population\_Manager().

```
00740                                                           {
00741   RipleysOutputPrb1 = fopen("RipleyOutput_Jan.txt", "w" );
00742   if ( !RipleysOutputPrb1 ) {
00743     g_msg->Warn( WARN_FILE, "Population_Manager::OpenTheRipleysOutputProbe(): ""Unable to open probe file",
00744          cfg_RipleysOutput_filename.value() );
00745     exit( 1 );
00746   }
00747   RipleysOutputPrb2 = fopen("RipleyOutput_Feb.txt", "w" );
00748   if ( !RipleysOutputPrb2 ) {
00749     g_msg->Warn( WARN_FILE, "Population_Manager::OpenTheRipleysOutputProbe(): ""Unable to open probe file",
00750          cfg_RipleysOutput_filename.value() );
00751     exit( 1 );
00752   }
00753   RipleysOutputPrb3 = fopen("RipleyOutput_Mar.txt", "w" );
00754   if ( !RipleysOutputPrb3 ) {
00755     g_msg->Warn( WARN_FILE, "Population_Manager::OpenTheRipleysOutputProbe(): ""Unable to open probe file",
00756          cfg_RipleysOutput_filename.value() );
00757     exit( 1 );
00758   }
00759   RipleysOutputPrb4 = fopen("RipleyOutput_Apr.txt", "w" );
00760   if ( !RipleysOutputPrb4 ) {
00761     g_msg->Warn( WARN_FILE, "Population_Manager::OpenTheRipleysOutputProbe(): ""Unable to open probe file",
00762          cfg_RipleysOutput_filename.value() );
00763     exit( 1 );
00764   }
00765   RipleysOutputPrb5 = fopen("RipleyOutput_May.txt", "w" );
00766   if ( !RipleysOutputPrb5 ) {
00767     g_msg->Warn( WARN_FILE, "Population_Manager::OpenTheRipleysOutputProbe(): ""Unable to open probe file",
00768          cfg_RipleysOutput_filename.value() );
00769     exit( 1 );
00770   }
00771   RipleysOutputPrb6 = fopen("RipleyOutput_Jun.txt", "w" );
00772   if ( !RipleysOutputPrb6 ) {
00773     g_msg->Warn( WARN_FILE, "Population_Manager::OpenTheRipleysOutputProbe(): ""Unable to open probe file",
00774          cfg_RipleysOutput_filename.value() );
00775     exit( 1 );
00776   }
00777   RipleysOutputPrb7 = fopen("RipleyOutput_Jul.txt", "w" );
00778   if ( !RipleysOutputPrb7 ) {
00779     g_msg->Warn( WARN_FILE, "Population_Manager::OpenTheRipleysOutputProbe(): ""Unable to open probe file",
00780          cfg_RipleysOutput_filename.value() );
00781     exit( 1 );
00782   }
00783   RipleysOutputPrb8 = fopen("RipleyOutput_Aug.txt", "w" );
00784   if ( !RipleysOutputPrb8 ) {
00785     g_msg->Warn( WARN_FILE, "Population_Manager::OpenTheRipleysOutputProbe(): ""Unable to open probe file",
00786          cfg_RipleysOutput_filename.value() );
00787     exit( 1 );
00788   }
00789   RipleysOutputPrb9 = fopen("RipleyOutput_Sep.txt", "w" );
00790   if ( !RipleysOutputPrb9 ) {
00791     g_msg->Warn( WARN_FILE, "Population_Manager::OpenTheRipleysOutputProbe(): ""Unable to open probe file",
00792          cfg_RipleysOutput_filename.value() );
00793     exit( 1 );
00794   }
00795   RipleysOutputPrb10 = fopen("RipleyOutput_Oct.txt", "w" );
00796   if ( !RipleysOutputPrb10 ) {
00797     g_msg->Warn( WARN_FILE, "Population_Manager::OpenTheRipleysOutputProbe(): ""Unable to open probe file",
00798          cfg_RipleysOutput_filename.value() );
00799     exit( 1 );
00800   }
00801   RipleysOutputPrb11 = fopen("RipleyOutput_Nov.txt", "w" );
00802   if ( !RipleysOutputPrb11 ) {
00803     g_msg->Warn( WARN_FILE, "Population_Manager::OpenTheRipleysOutputProbe(): ""Unable to open probe file",
00804          cfg_RipleysOutput_filename.value() );
00805     exit( 1 );
00806   }
00807   RipleysOutputPrb12 = fopen("RipleyOutput_Dec.txt", "w" );
00808   if ( !RipleysOutputPrb12 ) {
00809     g_msg->Warn( WARN_FILE, "Population_Manager::OpenTheRipleysOutputProbe(): ""Unable to open probe file",
00810          cfg_RipleysOutput_filename.value() );
00811     exit( 1 );
00812   }
00813   return true;
00814 }
```

|  |  |  |  |  |
| --- | --- | --- | --- | --- |
| bool Population\_Manager::OpenTheReallyBigProbe | ( |  | ) |  |

open the probe

References cfg\_ReallyBigOutput\_filename(), and ReallyBigOutputPrb.

Referenced by Vole\_Population\_Manager::Init().

```
00821                                                {
00822   ReallyBigOutputPrb = fopen(cfg_ReallyBigOutput_filename.value(), "w" );
00823   if ( !ReallyBigOutputPrb ) {
00824     g_msg->Warn( WARN_FILE, "Population_Manager::OpenTheRipleysOutputProbe(): ""Unable to open probe file",
00825          cfg_ReallyBigOutput_filename.value() );
00826     exit( 1 );
00827   }
00828   return true;
00829 }
```

|  |  |  |  |  |
| --- | --- | --- | --- | --- |
| bool Population\_Manager::OpenTheRipleysOutputProbe | ( |  | ) |  |

open the probe

References cfg\_RipleysOutput\_filename(), and RipleysOutputPrb.

Referenced by Vole\_Population\_Manager::Init().

```
00726                                                    {
00727   RipleysOutputPrb = fopen(cfg_RipleysOutput_filename.value(), "w" );
00728   if ( !RipleysOutputPrb ) {
00729     g_msg->Warn( WARN_FILE, "Population_Manager::OpenTheRipleysOutputProbe(): ""Unable to open probe file",
00730          cfg_RipleysOutput_filename.value() );
00731     exit( 1 );
00732   }
00733   return true;
00734 }
```

|  |  |  |  |
| --- | --- | --- | --- |
| float Population\_Manager::Probe | ( | int | *ListIndex*, |
|  |  | probe\_data \* | *p\_TheProbe* |  |
|  | ) |  |  | `[virtual]` |

Default data probe. Rarely used in actuality but always available

References AnimalPosition::m\_EleType, probe\_data::m\_NoAreas, probe\_data::m\_NoEleTypes, probe\_data::m\_NoFarms, probe\_data::m\_NoVegTypes, probe\_data::m\_Rect, probe\_data::m\_RefEle, probe\_data::m\_RefFarms, probe\_data::m\_RefVeg, AnimalPosition::m\_VegType, AnimalPosition::m\_x, rectangle::m\_x1, rectangle::m\_x2, AnimalPosition::m\_y, rectangle::m\_y1, rectangle::m\_y2, SimH, SimW, and TheArray.

Referenced by ProbeReport().

```
00610                                                                         {
00611         // Counts through the list and goes through each area to see if the animal
00612         // is standing there and if the farm, veg or element conditions are met
00613         AnimalPosition Sp;
00614         float NumberSk = 0;
00615         // Four possibilites
00616         // either NoVegTypes or NoElementTypes or NoFarmTypes is >0 or all==0
00617         if ( p_TheProbe->m_NoFarms != 0 ) {
00618                 for ( unsigned j = 0; j < TheArray[ ListIndex ].size(); j++ ) {
00619                         Sp = TheArray[ ListIndex ] [ j ]->SupplyPosition();
00620                         unsigned Farm = TheArray[ ListIndex ] [ j ]->SupplyFarmOwnerRef();
00621                         for ( unsigned i = 0; i < p_TheProbe->m_NoAreas; i++ ) {
00622                                 if ( ( Sp.m_x >= p_TheProbe->m_Rect[ i ].m_x1 ) && ( Sp.m_y >= p_TheProbe->m_Rect[ i ].m_y1 )
00623                                                 && ( Sp.m_x <= p_TheProbe->m_Rect[ i ].m_x2 ) && ( Sp.m_y <= p_TheProbe->m_Rect[ i ].m_y2 ) )
00624                                 for ( unsigned k = 0; k < p_TheProbe->m_NoFarms; k++ ) {
00625                                         if ( p_TheProbe->m_RefFarms[ k ] == Farm )
00626                                         NumberSk++; // it is in the square so increment number
00627                                 }
00628 
00629                         }
00630     }
00631         } else if ( p_TheProbe->m_NoEleTypes != 0 ) {
00632     for ( unsigned j = 0; j < TheArray[ ListIndex ].size(); j++ ) {
00633       Sp = TheArray[ ListIndex ] [ j ]->SupplyPosition();
00634       for ( unsigned i = 0; i < p_TheProbe->m_NoAreas; i++ ) {
00635         if ( ( Sp.m_x >= p_TheProbe->m_Rect[ i ].m_x1 ) && ( Sp.m_y >= p_TheProbe->m_Rect[ i ].m_y1 )
00636              && ( Sp.m_x <= p_TheProbe->m_Rect[ i ].m_x2 ) && ( Sp.m_y <= p_TheProbe->m_Rect[ i ].m_y2 ) )
00637                for ( unsigned k = 0; k < p_TheProbe->m_NoEleTypes; k++ ) {
00638                  if ( p_TheProbe->m_RefEle[ k ] == Sp.m_EleType )
00639                    NumberSk++; // it is in the square so increment number
00640                }
00641       }
00642     }
00643         } else {
00644     if ( p_TheProbe->m_NoVegTypes != 0 ) {
00645       for ( unsigned j = 0; j < TheArray[ ListIndex ].size(); j++ ) {
00646         Sp = TheArray[ ListIndex ] [ j ]->SupplyPosition();
00647 
00648         for ( unsigned i = 0; i < p_TheProbe->m_NoAreas; i++ ) {
00649           if ( ( Sp.m_x >= p_TheProbe->m_Rect[ i ].m_x1 ) && ( Sp.m_y >= p_TheProbe->m_Rect[ i ].m_y1 )
00650                && ( Sp.m_x <= p_TheProbe->m_Rect[ i ].m_x2 ) && ( Sp.m_y <= p_TheProbe->m_Rect[ i ].m_y2 ) ) {
00651                  for ( unsigned k = 0; k < p_TheProbe->m_NoVegTypes; k++ ) {
00652                    if ( p_TheProbe->m_RefVeg[ k ] == Sp.m_VegType )
00653                      NumberSk++; // it is in the square so increment number
00654                  }
00655           }
00656         }
00657       }
00658     } else // both must be zero
00659     {
00660                 unsigned sz = (unsigned) TheArray[ ListIndex ].size();
00661                 // It is worth checking whether we have a total dump of all individuals
00662                 // if so don't bother with asking each where it is
00663                 if (p_TheProbe->m_NoAreas==1) {
00664                         if ((p_TheProbe->m_Rect[0].m_x1==0) && (p_TheProbe->m_Rect[0].m_y1==0) && (p_TheProbe->m_Rect[0].m_x2==(unsigned)SimW) && 
00665                                 (p_TheProbe->m_Rect[0].m_y2==(unsigned)SimH)) {
00666                                         return (float) sz;
00667                         }
00668                 }
00669                 // Asking for a subset - need to test them all
00670                 for ( unsigned j = 0; j < sz; j++ ) {
00671                         Sp = TheArray[ ListIndex ] [ j ]->SupplyPosition();
00672                         for ( unsigned i = 0; i < p_TheProbe->m_NoAreas; i++ ) {
00673                                 if ( ( Sp.m_x >= p_TheProbe->m_Rect[ i ].m_x1 ) && ( Sp.m_y >= p_TheProbe->m_Rect[ i ].m_y1 )
00674                                            && ( Sp.m_x <= p_TheProbe->m_Rect[ i ].m_x2 ) && ( Sp.m_y <= p_TheProbe->m_Rect[ i ].m_y2 ) )
00675                                                 NumberSk++; // it is in the square so increment number
00676                         }
00677                 }
00678         }
00679         }
00680         return NumberSk;
00681 }
```

|  |  |  |  |
| --- | --- | --- | --- |
| int Population\_Manager::ProbeFileInput | ( | char \* | *p\_Filename*, |
|  |  | int | *p\_ProbeNo* |  |
|  | ) |  |  |  |

Default probe file input

References probe\_data::FileRecord, probe\_data::m\_NoAreas, probe\_data::m\_NoEleTypes, probe\_data::m\_NoFarms, probe\_data::m\_NoVegTypes, probe\_data::m\_Rect, probe\_data::m\_RefEle, probe\_data::m\_RefFarms, probe\_data::m\_RefVeg, probe\_data::m\_ReportInterval, probe\_data::m\_TargetTypes, m\_TheLandscape, rectangle::m\_x1, rectangle::m\_x2, rectangle::m\_y1, rectangle::m\_y2, and TheProbe.

```
00504                                                                          {
00505   FILE * PFile;
00506   int data = 0;
00507   int data2 = 0;
00508   char S[ 255 ];
00509   PFile = fopen(p_Filename, "r" );
00510   if ( !PFile ) {
00511     m_TheLandscape->Warn( "Population Manager - cannot open Probe File ", p_Filename );
00512     exit( 0 );
00513   }
00514   fgets( S, 255, PFile ); // dummy line
00515   fgets( S, 255, PFile ); // dummy line
00516   fscanf( PFile, "%d\n", & data ); // Reporting interval
00517   TheProbe[ p_ProbeNo ]->m_ReportInterval = data;
00518   fgets( S, 255, PFile ); // dummy line
00519   fscanf( PFile, "%d\n", & data ); // Write to file
00520   if ( data == 0 ) TheProbe[ p_ProbeNo ]->FileRecord = false; else
00521     TheProbe[ p_ProbeNo ]->FileRecord = true;
00522   fgets( S, 255, PFile ); // dummy line
00523   for ( int i = 0; i < 10; i++ ) {
00524     fscanf( PFile, "%d", & data );
00525     if ( data > 0 ) TheProbe[ p_ProbeNo ]->m_TargetTypes[ i ] = true; else
00526       TheProbe[ p_ProbeNo ]->m_TargetTypes[ i ] = false;
00527   }
00528 
00529   fgets( S, 255, PFile ); // dummy line
00530   fgets( S, 255, PFile ); // dummy line
00531   fscanf( PFile, "%d", & data );
00532   TheProbe[ p_ProbeNo ]->m_NoAreas = data;
00533   fgets( S, 255, PFile ); // dummy line
00534   fgets( S, 255, PFile ); // dummy line
00535   fscanf( PFile, "%d", & data2 ); // No References areas
00536   fgets( S, 255, PFile ); // dummy line
00537   fgets( S, 255, PFile ); // dummy line
00538   fscanf( PFile, "%d", & data ); // Type reference for probe
00539   if ( data == 1 ) TheProbe[ p_ProbeNo ]->m_NoEleTypes = data2; else
00540     TheProbe[ p_ProbeNo ]->m_NoEleTypes = 0;
00541   if ( data == 2 ) TheProbe[ p_ProbeNo ]->m_NoVegTypes = data2; else
00542     TheProbe[ p_ProbeNo ]->m_NoVegTypes = 0;
00543   if ( data == 3 ) TheProbe[ p_ProbeNo ]->m_NoFarms = data2; else
00544     TheProbe[ p_ProbeNo ]->m_NoFarms = 0;
00545   fgets( S, 255, PFile ); // dummy line
00546   fgets( S, 255, PFile ); // dummy line
00547   // Now read in the areas data
00548   for ( int i = 0; i < 10; i++ ) {
00549     fscanf( PFile, "%d", & data );
00550     TheProbe[ p_ProbeNo ]->m_Rect[ i ].m_x1 = data;
00551     fscanf( PFile, "%d", & data );
00552     TheProbe[ p_ProbeNo ]->m_Rect[ i ].m_y1 = data;
00553     fscanf( PFile, "%d", & data );
00554     TheProbe[ p_ProbeNo ]->m_Rect[ i ].m_x2 = data;
00555     fscanf( PFile, "%d", & data );
00556     TheProbe[ p_ProbeNo ]->m_Rect[ i ].m_y2 = data;
00557   }
00558   fgets( S, 255, PFile ); // dummy line
00559   fgets( S, 255, PFile ); // dummy line
00560   if ( TheProbe[ p_ProbeNo ]->m_NoVegTypes > 0 ) {
00561     for ( int i = 0; i < 25; i++ ) {
00562       fscanf( PFile, "%d", & data );
00563       if ( data != 999 )
00564         TheProbe[ p_ProbeNo ]->m_RefVeg[ i ] = m_TheLandscape->TranslateVegTypes( data );
00565     }
00566   } else if ( TheProbe[ p_ProbeNo ]->m_NoFarms > 0 ) {
00567     for ( int i = 0; i < 25; i++ ) {
00568       fscanf( PFile, "%d", & data );
00569       if ( data != 999 )
00570         TheProbe[ p_ProbeNo ]->m_RefFarms[ i ] = data;
00571     }
00572   } else {
00573     for ( int i = 0; i < 25; i++ ) {
00574       fscanf( PFile, "%d", & data );
00575       if ( data != 999 ) TheProbe[ p_ProbeNo ]->m_RefEle[ i ] = m_TheLandscape->TranslateEleTypes( data );
00576     }
00577   }
00578   fclose( PFile );
00579   return data2; // number of data references
00580 }
```

|  |  |  |  |  |  |
| --- | --- | --- | --- | --- | --- |
| char \* Population\_Manager::ProbeReport | ( | int | *a\_time* | ) |  |

References BeginningOfMonth(), probe\_data::FileOutput(), g\_str, m\_NoProbes, Probe(), SupplyListIndexSize(), and TheProbe.

Referenced by SpeciesSpecificReporting().

```
01164                                                 {
01165 
01166   int No;
01167   char str[100]; // 100 out to be enough!!
01168   strcpy(g_str,"");
01169   bool found = false;
01170   for ( int ProbeNo = 0; ProbeNo < m_NoProbes; ProbeNo++ ) {
01171     No = 0;
01172     // See if we need to record/update this one
01173     // if time/months/years ==0 or every time
01174     if ( ( TheProbe[ ProbeNo ]->m_ReportInterval == 3 )
01175          || ( ( TheProbe[ ProbeNo ]->m_ReportInterval == 2 ) && ( BeginningOfMonth() ) )
01176          || ( ( TheProbe[ ProbeNo ]->m_ReportInterval == 1 ) && ( Time % 365 == 0 ) ) ) {
01177            // Goes through each area and sends a value to OutputForm
01178            unsigned Index = SupplyListIndexSize();
01179            for ( unsigned listindex = 0; listindex < Index; listindex++ ) {
01180              if ( TheProbe[ ProbeNo ]->m_TargetTypes[ listindex ] )
01181                No += (int) Probe( listindex, TheProbe[ ProbeNo ] );
01182            }
01183            TheProbe[ ProbeNo ]->FileOutput( No, Time, ProbeNo );
01184                         sprintf(str," %d ", No );
01185                         strcat(g_str,str);                              
01186                         found = true;
01187     }
01188   }
01189   return g_str;
01190 }
```

|  |  |  |  |  |  |
| --- | --- | --- | --- | --- | --- |
| void Population\_Manager::Run | ( | int | *NoTSteps* | ) | `[virtual]` |

This is the main scheduling method for the population manager.   
Note the structure of Shuffle\_or\_Sort(), DoFirst(), BeginStep, DoBefore(), Step looping until all are finished, DoAfter(), DoAlmostLast(), EndStep, DoLast().   

Can do multiple time-steps here inside one landscape time-step (a day). This is used in the roe deer model to provide 10 minute behavioural time-steps.

Reimplemented in TPredator\_Population\_Manager.

References Catastrophe(), cfg\_CatastropheEventStartYear(), cfg\_CIPEGridOutput\_day(), cfg\_CIPEGridOutput\_day\_b(), cfg\_CIPEGridOutput\_Interval(), cfg\_CIPEGridOutput\_used, cfg\_ReallyBigOutput\_used, cfg\_RipleysOutput\_day(), cfg\_RipleysOutput\_interval(), cfg\_RipleysOutput\_used, cfg\_RipleysOutputFirstYear(), cfg\_RipleysOutputMonthly\_used(), DoAfter(), DoAlmostLast(), DoBefore(), DoFirst(), DoLast(), m\_catastrophestartyear, m\_TheLandscape, RipleysOutputPrb, RipleysOutputPrb1, RipleysOutputPrb10, RipleysOutputPrb11, RipleysOutputPrb12, RipleysOutputPrb2, RipleysOutputPrb3, RipleysOutputPrb4, RipleysOutputPrb5, RipleysOutputPrb6, RipleysOutputPrb7, RipleysOutputPrb8, RipleysOutputPrb9, Shuffle\_or\_Sort(), StepFinished(), TheArray, TheCIPEGridOutputProbe(), TheCIPEGridOutputProbeB(), TheReallyBigOutputProbe(), and TheRipleysOutputProbe().

```
00260                                            {
00261   // begin step actions ...
00262   // set all stepdone to false.... is this really necessary??
00263   unsigned size2;
00264   unsigned size1 =  (unsigned) TheArray.size();
00265   for ( unsigned listindex = 0; listindex < size1; listindex++ ) {
00266     size2 = (unsigned) TheArray[ listindex ].size();
00267     for ( unsigned j = 0; j < size2; j++ ) {
00268       TheArray[ listindex ] [ j ]->StepDone = false;
00269     }
00270   }
00274   for ( int TSteps = 0; TSteps < NoTSteps; TSteps++ ) {
00275 
00276     for ( unsigned listindex = 0; listindex < size1; listindex++ ) {
00277       // Call the Shuffle/Sort procedures
00278       Shuffle_or_Sort( listindex );
00279     }
00280     // Need to check if Ripleys Statistic needs to be saved
00281     if ( cfg_RipleysOutput_used.value() ) {
00282       int Year = m_TheLandscape->SupplyYearNumber();
00283           if (Year>=cfg_RipleysOutputFirstYear.value()) {
00284                   if ( Year % cfg_RipleysOutput_interval.value() == 0 ) {
00285                         int day = m_TheLandscape->SupplyDayInYear();
00286                         if ( cfg_RipleysOutput_day.value() == day ) {
00287                           // Do the Ripley Probe
00288                           TheRipleysOutputProbe( RipleysOutputPrb );
00289                         }
00290                   }
00291                 }
00292         }
00293     // Need to check if Monthly Ripleys Statistic needs to be saved
00294     if ( cfg_RipleysOutputMonthly_used.value() ) {
00295                 if (m_TheLandscape->SupplyDayInMonth()==1) {
00296                         int Year = m_TheLandscape->SupplyYearNumber();
00297                         if (Year>=cfg_RipleysOutputFirstYear.value()) {
00298                                 if ( Year % cfg_RipleysOutput_interval.value() == 0 ) {
00299                                         int month = m_TheLandscape->SupplyMonth();
00300                                         // Do the Ripley Probe
00301                                         switch (month) {
00302                                                 case 1: TheRipleysOutputProbe( RipleysOutputPrb1 );
00303                                                         break;
00304                                                 case 2: TheRipleysOutputProbe( RipleysOutputPrb2 );
00305                                                         break;
00306                                                 case 3: TheRipleysOutputProbe( RipleysOutputPrb3 );
00307                                                         break;
00308                                                 case 4: TheRipleysOutputProbe( RipleysOutputPrb4 );
00309                                                         break;
00310                                                 case 5: TheRipleysOutputProbe( RipleysOutputPrb5 );
00311                                                         break;
00312                                                 case 6: TheRipleysOutputProbe( RipleysOutputPrb6 );
00313                                                         break;
00314                                                 case 7: TheRipleysOutputProbe( RipleysOutputPrb7 );
00315                                                         break;
00316                                                 case 8: TheRipleysOutputProbe( RipleysOutputPrb8 );
00317                                                         break;
00318                                                 case 9: TheRipleysOutputProbe( RipleysOutputPrb9 );
00319                                                         break;
00320                                                 case 10: TheRipleysOutputProbe( RipleysOutputPrb10 );
00321                                                         break;
00322                                                 case 11: TheRipleysOutputProbe( RipleysOutputPrb11 );
00323                                                         break;
00324                                                 case 12: TheRipleysOutputProbe( RipleysOutputPrb12 );
00325                                                         break;
00326                                         }
00327                                 }
00328                         }
00329                 }
00330         }
00331     // Need to check if Really Big Probe needs to be saved
00332     if ( cfg_ReallyBigOutput_used.value() ) {
00333       int Year = m_TheLandscape->SupplyYearNumber();
00334           if (Year>=cfg_RipleysOutputFirstYear.value()) {
00335                   if ( Year % cfg_RipleysOutput_interval.value() == 0 ) {
00336                         int day = m_TheLandscape->SupplyDayInYear();
00337                         if ( cfg_RipleysOutput_day.value() == day ) {
00338                           // Do the Ripley Probe
00339                           TheReallyBigOutputProbe();
00340                         }
00341                   }
00342                 }
00343         }
00344     // Need to check if CIPEGrid Statistic needs to be saved
00345     if ( cfg_CIPEGridOutput_used.value() ) {
00346       int Year = m_TheLandscape->SupplyYearNumber();
00347       if ( Year % cfg_CIPEGridOutput_Interval.value() == 0 ) {
00348         int day = m_TheLandscape->SupplyDayInYear();
00349         if ( cfg_CIPEGridOutput_day.value() == day ) {
00350           // Do the Ripley Probe
00351           TheCIPEGridOutputProbe();
00352         }
00353         if ( cfg_CIPEGridOutput_day_b.value() == day ) {
00354           // Do the Ripley Probe
00355           TheCIPEGridOutputProbeB();
00356         }
00357       }
00358     }
00359         int yr=m_TheLandscape->SupplyYearNumber();
00360         if ( yr > cfg_CatastropheEventStartYear.value() ) {
00361                 if (m_catastrophestartyear==-1) m_catastrophestartyear=yr;
00362                 Catastrophe(); // This method must be overidden in descendent classes
00363         }
00364     DoFirst();
00365     // call the begin-step-method of all objects
00366     for ( unsigned listindex = 0; listindex < size1; listindex++ ) {
00367       size2 = (unsigned) TheArray[ listindex ].size();
00368       for ( unsigned j = 0; j < size2; j++ )
00369         TheArray[ listindex ] [ j ]->BeginStep();
00370     }
00371     DoBefore();
00372     // call the step-method of all objects
00373     do {
00374       for ( unsigned listindex = 0; listindex < size1; listindex++ ) {
00375         //DEBUG
00376         //LOG("SecondLog.txt");
00377         size2 = (unsigned) TheArray[ listindex ].size();
00378         for ( unsigned j = 0; j < size2; j++ ) {
00379           TheArray[ listindex ] [ j ]->Step();
00380         }
00381       } // for listindex
00382     } while ( !StepFinished() );
00383     DoAfter();
00384     // call the end-step-method of all objects
00385     for ( unsigned listindex = 0; listindex < size1; listindex++ ) {
00386       size2 = (int) TheArray[ listindex ].size();
00387       for ( unsigned j = 0; j < size2; j++ ) {
00388         TheArray[ listindex ] [ j ]->EndStep();
00389       }
00390     }
00391     // ----------------
00392     // end of this step actions
00393 
00394     // For each animal list
00395     DoAlmostLast();
00396     for ( unsigned listindex = 0; listindex < size1; listindex++ ) {
00397       // Must check each object in the list for CurrentStateNo==-1
00398       int TAend = (int) TheArray[ listindex ].size() - 1;
00399       for ( int j = TAend; j >= 0; j-- ) // Search backwards is more efficicent
00400       {
00401         if ( TheArray[ listindex ] [ j ]->CurrentStateNo == -1 ) // code for kill it
00402         {
00403           delete TheArray[ listindex ] [ j ];
00404           TheArray[ listindex ].erase( TheArray[ listindex ].begin() + j );
00405         }
00406       }
00407     }
00408     DoLast();
00409 
00410   } // End of time step loop
00411 }
```

|  |  |  |  |  |  |
| --- | --- | --- | --- | --- | --- |
| void Population\_Manager::SetNoProbes | ( | int | *a\_pn* | ) | `[inline]` |

References m\_NoProbes.

```
00236 { m_NoProbes=a_pn; }
```

|  |  |  |  |  |  |
| --- | --- | --- | --- | --- | --- |
| void Population\_Manager::Shuffle | ( | unsigned | *Type* | ) | `[protected]` |

Run once through the list swapping randomly chosen elements

References TheArray.

Referenced by Shuffle\_or\_Sort().

```
00973                                                 {
00974   unsigned s = (unsigned) TheArray[ Type ].size();
00975   for ( unsigned i = 0; i < s; i++ ) {
00976     TAnimal * temp;
00977     unsigned a = random( s );
00978     unsigned b = random( s );
00979     temp = TheArray[ Type ] [ a ];
00980     TheArray[ Type ] [ a ] = TheArray[ Type ] [ b ];
00981     TheArray[ Type ] [ b ] = temp;
00982   }
00983 }
```

|  |  |  |  |  |  |
| --- | --- | --- | --- | --- | --- |
| void Population\_Manager::Shuffle\_or\_Sort | ( | unsigned | *Type* | ) | `[protected]` |

This method is used to determine whether the array of animals should be shuffled or sorted.   
To do nothing simply avoid calling this at the beginning of the time-step

References BeforeStepActions, m\_TheLandscape, Shuffle(), SortX(), SortXIndex(), and SortY().

Referenced by Run().

```
00991                                                         {
00992   switch ( BeforeStepActions[ Type ] ) {
00993     case 0:
00994       Shuffle( Type );
00995     break;
00996     case 1:
00997       SortX( Type );
00998     break;
00999     case 2:
01000       SortY( Type );
01001     break;
01002     case 3:
01003       SortXIndex( Type );
01004     break;
01005     default:
01006       m_TheLandscape->Warn( "Population_Manager::Shuffle_or_Sort- BeforeStepAction Unknown", NULL );
01007       exit( 1 );
01008   }
01009 }
```

|  |  |  |  |  |  |
| --- | --- | --- | --- | --- | --- |
| void Population\_Manager::SortState | ( | unsigned | *Type* | ) | `[protected]` |

Sort the array w.r.t. the current state attribute

References TheArray.

```
00937                                                   {
00938   sort( TheArray[ Type ].begin(), TheArray[ Type ].end(), CompareState() );
00939 }
```

|  |  |  |  |  |  |
| --- | --- | --- | --- | --- | --- |
| void Population\_Manager::SortX | ( | unsigned | *Type* | ) | `[protected]` |

Sort the array w.r.t. the m\_Location\_x attribute

References TheArray.

Referenced by Shuffle\_or\_Sort().

```
00919                                               {
00920   sort( TheArray[ Type ].begin(), TheArray[ Type ].end(), CompareX() );
00921 }
```

|  |  |  |  |  |  |
| --- | --- | --- | --- | --- | --- |
| void Population\_Manager::SortXIndex | ( | unsigned | *Type* | ) | `[protected]` |

Sort the array w.r.t. the m\_Location\_x attribute, and make an indexing array

References IndexArrayX, and TheArray.

Referenced by Shuffle\_or\_Sort().

```
00946                                                    {
00947   sort( TheArray[ Type ].begin(), TheArray[ Type ].end(), CompareX() );
00948   unsigned s = (unsigned) TheArray[ Type ].size();
00949   // Now make the index array to X;
00950   int counter = 0;
00951   int x;
00952   // for each individual
00953   for ( unsigned i = 0; i < s; i++ ) {
00954     // Get Next co-ordiated
00955     x = TheArray[ Type ] [ i ]->Supply_m_Location_x();
00956     // fill gaps up to x
00957     while ( counter < x ) IndexArrayX[ Type ] [ counter++ ] = -1;
00958     if ( x == counter ) {
00959       IndexArrayX[ Type ] [ counter++ ] = i;
00960     }
00961   }
00962   // Fill up the rest of the array with -1;
00963   for ( int c = counter; c < 10000; c++ ) {
00964     IndexArrayX[ Type ] [ c ] = -1;
00965   }
00966 }
```

|  |  |  |  |  |  |
| --- | --- | --- | --- | --- | --- |
| void Population\_Manager::SortY | ( | unsigned | *Type* | ) | `[protected]` |

Sort the array w.r.t. the m\_Location\_y attribute

References TheArray.

Referenced by Shuffle\_or\_Sort().

```
00928                                               {
00929   sort( TheArray[ Type ].begin(), TheArray[ Type ].end(), CompareY() );
00930 }
```

|  |  |  |  |
| --- | --- | --- | --- |
| char \* Population\_Manager::SpeciesSpecificReporting | ( | int | *a\_species*, |
|  |  | int | *a\_time* |  |
|  | ) |  |  |  |

This method handles species specific outputs. This is one place to do it. More commonly this is done in descendent classes

References BreedingSuccessProbeOutput(), cfg\_UseEasyPop(), FledgelingProbeOutput(), g\_str, GeneticsResultsOutput(), ImpactProbeReport(), m\_EasyPopRes, m\_NoProbes, m\_TheLandscape, ProbeReport(), TheBreedingSuccessProbe(), and TheFledgelingProbe().

```
01034                                                                             {
01035   strcpy(g_str,"");
01036 // Vole Model
01037   if ( a_species == 1 ) {
01038 /*
01039 // The next lines are obselete code from genetic simulations
01040 float MeanNoAlleles;
01041     float MeanHO;
01042     float MeanHE;
01043     unsigned size;
01044 
01045         if ( m_time % ( 365 * 24 * 60 ) == 0 ) // End of year
01046     {
01047 
01048       // empty the GenStruc data
01049       for ( int ProbeNo = 0; ProbeNo < ( int )m_NoProbes; ProbeNo++ ) {
01050         fprintf( m_GeneticsFile, "%d ", m_time );
01051         int TotalSize = 0;
01052         m_AManager->AlFreq->Flush();
01053         for ( unsigned listindex = 0; listindex <= 1; listindex++ ) {
01054           size = 0;
01055           m_AManager->TheGeneticProbe( listindex, ProbeNo, size );
01056           TotalSize += size;
01057         }
01058         if ( TotalSize > 0 ) {
01059           m_AManager->AlFreq->CalcAF();
01060           m_AManager->AlFreq->CalcNoAlleles();
01061           m_AManager->AlFreq->CalcHE();
01062           m_AManager->AlFreq->CalcHO( TotalSize );
01063         }
01064         // Get the means
01065         // For N alleles
01066         int NA = 0;
01067         float NHO = 0;
01068         float NHE = 0;
01069         for ( int l = 0; l < 16; l++ ) {
01070           NA += m_AManager->AlFreq->SupplyNoAlleles( l );
01071         }
01072         MeanNoAlleles = ( float )NA / 16.0;
01073         for ( int l = 0; l < 16; l++ ) {
01074           NHO += m_AManager->AlFreq->SupplyHO( l );
01075         }
01076         MeanHO = NHO / 16.0;
01077         for ( int l = 0; l < 16; l++ ) {
01078           NHE += m_AManager->AlFreq->SupplyHE( l );
01079         }
01080         MeanHE = NHE / 16.0;
01081 
01082         // Do some output
01083         // Output is to two files, the genetics file and the AllelerequencyFile
01084         fprintf( m_GeneticsFile, "%2.2f ", MeanNoAlleles );
01085         fprintf( m_GeneticsFile, "%1.3f ", MeanHO );
01086         fprintf( m_GeneticsFile, "%1.3f ", MeanHE );
01087         for ( int l = 0; l < 16; l++ ) {
01088           fprintf( m_GeneticsFile, "%04i ", m_AManager->AlFreq->SupplyNoAlleles( l ) );
01089         }
01090         for ( int l = 0; l < 16; l++ ) {
01091           fprintf( m_GeneticsFile, "%1.3f ", m_AManager->AlFreq->SupplyHO( l ) );
01092         }
01093         for ( int l = 0; l < 16; l++ ) {
01094           fprintf( m_GeneticsFile, "%1.3f ", m_AManager->AlFreq->SupplyHE( l ) );
01095         }
01096         fprintf( m_GeneticsFile, "\n" );
01097         fprintf( m_AlleleFreqsFile, "%d ", m_time );
01098         for ( int l = 0; l < 16; l++ ) {
01099           for ( int al = 0; al < 4; al++ ) {
01100             fprintf( m_AlleleFreqsFile, "%1.3f ", m_AManager->AlFreq->SupplyAF( l, al ) );
01101           }
01102         }
01103         fprintf( m_AlleleFreqsFile, "\n" );
01104       }
01105       // make sure progress reaches the disk
01106       fflush( m_GeneticsFile );
01107       fflush( m_AlleleFreqsFile );
01108     }
01109 */
01110     if ( cfg_UseEasyPop.value()) {
01111       int targettime=a_time % ( 365 * 100 );
01112       if ( (  targettime == 59 ) ||(targettime == 181)||(targettime ==304)||(targettime ==59+365)||(targettime ==181+365)||(targettime ==304+365)) {
01113           for ( unsigned listindex = 0; listindex <= 1; listindex++ ) {
01114             GeneticsResultsOutput( m_EasyPopRes, listindex);
01115         }
01116         fflush( m_EasyPopRes );
01117       }
01118     }
01119 
01120     /* Three times a year reporting 
01121         if ( m_time % 365 == 60 ) ProbeReportNow( m_time );
01122     if ( m_time % 365 == 152 ) ProbeReportNow( m_time );
01123     if ( m_time % 365 == 273 ) ProbeReportNow( m_time ); */
01124     ProbeReport( a_time );
01125 #ifdef __SpecificPesticideEffectsVinclozolinLike__
01126         ImpactProbeReport( a_time );
01127 #endif
01128   }
01129   // Skylark Model
01130   else if ( a_species == 0 ) {
01131     int No;
01132     //if ( a_time % 365 == 364 ) 
01133         {
01134       //Write the Breeding Attempts Probe
01135       int BreedingFemales, YoungOfTheYear, TotalPop, TotalFemales, TotalMales, BreedingAttempts;
01136       int No = TheBreedingSuccessProbe( BreedingFemales, YoungOfTheYear, TotalPop, TotalFemales, TotalMales, BreedingAttempts );
01137       float bs = 0;
01138       if ( BreedingFemales > 0 ) {
01139         bs = No / ( float )BreedingFemales;
01140       }
01141       BreedingSuccessProbeOutput( bs, BreedingFemales, YoungOfTheYear, TotalPop,
01142            TotalFemales, TotalMales, a_time, BreedingAttempts );
01143     }
01144     if ( a_time % 365 == 152 ) {
01145       // Need to fill in the landscape from 1st June, it will change before
01146       // the count of fledgelings is needed
01147       m_TheLandscape->FillVegAreaData();
01148     }
01149     if ( a_time % 365 == 197 ) {
01150       for ( int ProbeNo = 0; ProbeNo < m_NoProbes; ProbeNo++ ) {
01151         No = TheFledgelingProbe();
01152         // Do some output
01153         FledgelingProbeOutput( No, a_time );
01154       }
01155     }
01156     return ProbeReport( a_time ); // ProbeReport135( m_time );
01157   } 
01158   else return ProbeReport( a_time );
01159   return g_str;
01160 }
```

|  |  |  |  |  |  |
| --- | --- | --- | --- | --- | --- |
| bool Population\_Manager::StepFinished | ( | void |  | ) | `[protected, virtual]` |

Returns true if and only if all objects have finished the current step

Reimplemented in TPredator\_Population\_Manager.

References TheArray.

Referenced by Run().

```
00416                                             {
00417   for ( unsigned listindex = 0; listindex < TheArray.size(); listindex++ ) {
00418     for ( unsigned j = 0; j < TheArray[ listindex ].size(); j++ ) {
00419       if ( TheArray[ listindex ] [ j ]->StepDone == false ) {
00420         return false;
00421       }
00422     }
00423   }
00424   return true;
00425 }
```

|  |  |  |  |  |  |
| --- | --- | --- | --- | --- | --- |
| virtual int Population\_Manager::SupplyCovPosx | ( | int |  | ) | `[inline, virtual]` |

```
00412                                    {
00413     return 0;
00414   }
```

|  |  |  |  |  |  |
| --- | --- | --- | --- | --- | --- |
| virtual int Population\_Manager::SupplyCovPosy | ( | int |  | ) | `[inline, virtual]` |

```
00415                                    {
00416     return 0;
00417   }
```

|  |  |  |  |  |
| --- | --- | --- | --- | --- |
| unsigned Population\_Manager::SupplyListIndexSize | ( |  | ) | `[inline]` |

References TheArray.

Referenced by ProbeReport().

```
00271                                  {
00272         return (unsigned)TheArray.size();
00273   }
```

|  |  |  |  |  |  |
| --- | --- | --- | --- | --- | --- |
| AnsiString Population\_Manager::SupplyListName | ( | int | *i* | ) | `[inline]` |

References ListNames.

```
00279                                      {
00280     return ListNames[ i ];
00281   }
```

|  |  |  |  |  |
| --- | --- | --- | --- | --- |
| int Population\_Manager::SupplyListNameLength | ( |  | ) | `[inline]` |

References ListNameLength.

```
00268                              {
00269     return ListNameLength;
00270   }
```

|  |  |  |  |  |  |
| --- | --- | --- | --- | --- | --- |
| unsigned Population\_Manager::SupplyListSize | ( | unsigned | *listindex* | ) | `[inline]` |

References TheArray.

```
00274                                                 {
00275     return (unsigned) TheArray[ listindex ].size();
00276   }
```

|  |  |  |  |
| --- | --- | --- | --- |
| virtual void Population\_Manager::SupplyLocXY | ( | unsigned | *listindex*, |
|  |  | unsigned | *j*, |
|  |  | int & | *x*, |
|  |  | int & | *y* |  |
|  | ) |  |  | `[inline, virtual]` |

References TheArray.

```
00292                                                                                {
00293     x = TheArray[ listindex ] [ j ]->Supply_m_Location_x();
00294     y = TheArray[ listindex ] [ j ]->Supply_m_Location_y();
00295   }
```

|  |  |  |  |  |  |
| --- | --- | --- | --- | --- | --- |
| virtual int Population\_Manager::SupplyPegPosx | ( | int |  | ) | `[inline, virtual]` |

```
00406                                    {
00407     return 0;
00408   }
```

|  |  |  |  |  |  |
| --- | --- | --- | --- | --- | --- |
| virtual int Population\_Manager::SupplyPegPosy | ( | int |  | ) | `[inline, virtual]` |

```
00409                                    {
00410     return 0;
00411   }
```

|  |  |  |  |  |
| --- | --- | --- | --- | --- |
| int Population\_Manager::SupplySimH | ( |  | ) | `[inline]` |

References SimH.

```
00262                    {
00263     return SimH;
00264   }
```

|  |  |  |  |  |
| --- | --- | --- | --- | --- |
| int Population\_Manager::SupplySimW | ( |  | ) | `[inline]` |

References SimW.

```
00259                    {
00260     return SimW;
00261   }
```

|  |  |  |  |
| --- | --- | --- | --- |
| int Population\_Manager::SupplyState | ( | unsigned | *listindex*, |
|  |  | unsigned | *j* |  |
|  | ) |  |  | `[inline]` |

References TheArray.

```
00289                                                     {
00290     return TheArray[ listindex ] [ j ]->WhatState();
00291   }
```

|  |  |  |  |  |
| --- | --- | --- | --- | --- |
| IntArray100\* Population\_Manager::SupplyStateList | ( |  | ) | `[inline]` |

References StateList.

```
00286                                   {
00287     return & StateList;
00288   }
```

|  |  |  |  |  |  |
| --- | --- | --- | --- | --- | --- |
| AnsiString Population\_Manager::SupplyStateNames | ( | int | *i* | ) | `[inline]` |

References StateNames.

```
00296                                        {
00297     return StateNames[ i ];
00298   }
```

|  |  |  |  |  |
| --- | --- | --- | --- | --- |
| unsigned Population\_Manager::SupplyStateNamesLength | ( |  | ) | `[inline]` |

References StateNamesLength.

```
00299                                     {
00300     return StateNamesLength;
00301   }
```

|  |  |  |  |  |
| --- | --- | --- | --- | --- |
| int Population\_Manager::SupplyStepSize | ( |  | ) | `[inline]` |

References m\_StepSize.

```
00256                        {
00257     return m_StepSize;
00258   }
```

|  |  |  |  |  |  |
| --- | --- | --- | --- | --- | --- |
| virtual int Population\_Manager::TheBreedingFemalesProbe | ( | int |  | ) | `[inline, virtual]` |

```
00429                                              {
00430         return 0;
00431   }
```

|  |  |  |  |
| --- | --- | --- | --- |
| virtual int Population\_Manager::TheBreedingSuccessProbe | ( | int & | , |
|  |  | int & | , |
|  |  | int & | , |
|  |  | int & | , |
|  |  | int & | , |
|  |  | int & |  |  |
|  | ) |  |  | `[inline, virtual]` |

Referenced by SpeciesSpecificReporting().

```
00437                                                                                        {
00438          return 0;
00439   }
```

|  |  |  |  |  |
| --- | --- | --- | --- | --- |
| void Population\_Manager::TheCIPEGridOutputProbe | ( |  | ) | `[virtual]` |

This method MUST be overridden in descendent classes, if you want it to work

Reimplemented in Vole\_Population\_Manager.

Referenced by Run().

```
00718                                                 {
00719 }
```

|  |  |  |  |  |
| --- | --- | --- | --- | --- |
| virtual void Population\_Manager::TheCIPEGridOutputProbeB | ( |  | ) | `[inline, virtual]` |

Referenced by Run().

```
00363 {}
```

|  |  |  |  |  |
| --- | --- | --- | --- | --- |
| virtual int Population\_Manager::TheFledgelingProbe | ( |  | ) | `[inline, virtual]` |

Referenced by SpeciesSpecificReporting().

```
00432                                    {
00433     return 0;
00434   }
```

|  |  |  |  |
| --- | --- | --- | --- |
| virtual void Population\_Manager::TheGeneticProbe | ( | unsigned | , |
|  |  | int | , |
|  |  | unsigned & |  |  |
|  | ) |  |  | `[inline, virtual]` |

```
00442                                                                {
00443   }
```

|  |  |  |  |  |
| --- | --- | --- | --- | --- |
| void Population\_Manager::TheReallyBigOutputProbe | ( |  | ) | `[virtual]` |

This method must be overridden in descendent classes

Reimplemented in Vole\_Population\_Manager.

Referenced by Run().

```
00875                                                  {
00876 }
```

|  |  |  |  |  |  |
| --- | --- | --- | --- | --- | --- |
| void Population\_Manager::TheRipleysOutputProbe | ( | FILE \* | *a\_prb* | ) | `[virtual]` |

This method must be overridden in descendent classes

Reimplemented in Vole\_Population\_Manager.

Referenced by Run().

```
00880                                                                   {
00881 }
```

|  |  |  |  |
| --- | --- | --- | --- |
| void Population\_Manager::WriteToTest2File | ( | char \* | *n*, |
|  |  | int | *n2* |  |
|  | ) |  |  | `[inline]` |

References TestFile2.

```
00253                                             {
00254     fprintf( TestFile2, "%s %i\n", n, n2 );
00255   }
```

|  |  |  |  |
| --- | --- | --- | --- |
| void Population\_Manager::WriteToTest2File | ( | int | *n*, |
|  |  | int | *n2* |  |
|  | ) |  |  | `[inline]` |

References TestFile2.

```
00247                                          {
00248         fprintf( TestFile2, "%i %i\n", n, n2 );
00249   }
```

|  |  |  |  |
| --- | --- | --- | --- |
| void Population\_Manager::WriteToTestFile | ( | char \* | *n*, |
|  |  | int | *n2* |  |
|  | ) |  |  | `[inline]` |

References TestFile.

```
00250                                            {
00251     fprintf( TestFile, "%s %i\n", n, n2 );
00252   }
```

|  |  |  |  |
| --- | --- | --- | --- |
| void Population\_Manager::WriteToTestFile | ( | int | *n*, |
|  |  | int | *n2* |  |
|  | ) |  |  | `[inline]` |

References TestFile.

```
00244                                               {
00245             fprintf( TestFile, "%i %i\n", n, n2 ); fflush( TestFile );
00246   }
```

---

## Member Data Documentation

|  |
| --- |
| AlleleFreq\* Population\_Manager::AlFreq |

Reimplemented in Vole\_Population\_Manager.

|  |
| --- |
| unsigned Population\_Manager::BeforeStepActions[10] `[protected]` |

Referenced by Vole\_Population\_Manager::Init(), Population\_Manager(), and Shuffle\_or\_Sort().

|  |
| --- |
| FILE\* Population\_Manager::CIPEGridOutputPrb `[protected]` |

Referenced by CloseTheCIPEGridOutputProbe(), OpenTheCIPEGridOutputProbe(), and Vole\_Population\_Manager::TheCIPEGridOutputProbe().

|  |
| --- |
| FILE\* Population\_Manager::CIPEGridOutputPrbB `[protected]` |

Referenced by CloseTheCIPEGridOutputProbe(), and OpenTheCIPEGridOutputProbe().

|  |
| --- |
| unsigned Population\_Manager::Counts[10][100] |

Referenced by DoLast().

|  |
| --- |
| double Population\_Manager::gridcount[25][25] `[protected]` |

Referenced by Vole\_Population\_Manager::TheCIPEGridOutputProbe().

|  |
| --- |
| int Population\_Manager::IndexArrayX[5][10000] |

Referenced by SortXIndex().

|  |
| --- |
| long int Population\_Manager::lamdagrid[2][257][257] `[protected]` |

Referenced by LamdaBirth(), LamdaClear(), LamdaDeath(), and LamdaDumpOutput().

|  |
| --- |
| unsigned Population\_Manager::ListNameLength `[protected]` |

Referenced by DoLast(), Vole\_Population\_Manager::Init(), and SupplyListNameLength().

|  |
| --- |
| AnsiString Population\_Manager::ListNames[10] `[protected]` |

Referenced by Vole\_Population\_Manager::Init(), LOG(), and SupplyListName().

|  |
| --- |
| FILE\* Population\_Manager::m\_AlleleFreqsFile `[protected]` |

Referenced by Vole\_Population\_Manager::Init(), and Vole\_Population\_Manager::~Vole\_Population\_Manager().

|  |
| --- |
| int Population\_Manager::m\_catastrophestartyear `[protected]` |

Referenced by Vole\_Population\_Manager::Catastrophe(), Population\_Manager(), and Run().

|  |
| --- |
| FILE\* Population\_Manager::m\_EasyPopRes `[protected]` |

Referenced by Vole\_Population\_Manager::Init(), SpeciesSpecificReporting(), and Vole\_Population\_Manager::~Vole\_Population\_Manager().

|  |
| --- |
| FILE\* Population\_Manager::m\_GeneticsFile `[protected]` |

Referenced by Vole\_Population\_Manager::Init(), and Vole\_Population\_Manager::~Vole\_Population\_Manager().

|  |
| --- |
| MyDialog\* Population\_Manager::m\_MainForm |

|  |
| --- |
| int Population\_Manager::m\_NoProbes `[protected]` |

Referenced by ProbeReport(), SetNoProbes(), and SpeciesSpecificReporting().

|  |
| --- |
| char Population\_Manager::m\_SimulationName[255] |

Referenced by Vole\_Population\_Manager::Init().

|  |
| --- |
| int Population\_Manager::m\_StepSize `[protected]` |

Referenced by Vole\_Population\_Manager::Init(), and SupplyStepSize().

|  |
| --- |
| Landscape\* Population\_Manager::m\_TheLandscape |

Referenced by BeginningOfMonth(), Vole\_Population\_Manager::Catastrophe(), Vole\_Population\_Manager::DoFirst(), Vole\_Population\_Manager::GeneticsResultsOutput(), Vole\_Population\_Manager::Init(), LOG(), Population\_Manager(), ProbeFileInput(), Run(), Vole\_Population\_Manager::SendMessage(), Shuffle\_or\_Sort(), SpeciesSpecificReporting(), Vole\_Population\_Manager::TheReallyBigOutputProbe(), Vole\_Population\_Manager::TheRipleysOutputProbe(), and TPredator\_Population\_Manager::TPredator\_Population\_Manager().

|  |
| --- |
| bool Population\_Manager::ProbesSet |

|  |
| --- |
| FILE\* Population\_Manager::ReallyBigOutputPrb `[protected]` |

Referenced by CloseTheReallyBigOutputProbe(), Vole\_Population\_Manager::Init(), OpenTheReallyBigProbe(), Vole\_Population\_Manager::TheReallyBigOutputProbe(), and TPredator\_Population\_Manager::TPredator\_Population\_Manager().

|  |
| --- |
| FILE\* Population\_Manager::RipleysOutputPrb `[protected]` |

Referenced by CloseTheRipleysOutputProbe(), OpenTheRipleysOutputProbe(), Run(), and TPredator\_Population\_Manager::TPredator\_Population\_Manager().

|  |
| --- |
| FILE\* Population\_Manager::RipleysOutputPrb1 `[protected]` |

Referenced by CloseTheMonthlyRipleysOutputProbe(), OpenTheMonthlyRipleysOutputProbe(), and Run().

|  |
| --- |
| FILE\* Population\_Manager::RipleysOutputPrb10 `[protected]` |

Referenced by CloseTheMonthlyRipleysOutputProbe(), OpenTheMonthlyRipleysOutputProbe(), and Run().

|  |
| --- |
| FILE\* Population\_Manager::RipleysOutputPrb11 `[protected]` |

Referenced by CloseTheMonthlyRipleysOutputProbe(), OpenTheMonthlyRipleysOutputProbe(), and Run().

|  |
| --- |
| FILE\* Population\_Manager::RipleysOutputPrb12 `[protected]` |

Referenced by CloseTheMonthlyRipleysOutputProbe(), OpenTheMonthlyRipleysOutputProbe(), and Run().

|  |
| --- |
| FILE\* Population\_Manager::RipleysOutputPrb2 `[protected]` |

Referenced by CloseTheMonthlyRipleysOutputProbe(), OpenTheMonthlyRipleysOutputProbe(), and Run().

|  |
| --- |
| FILE\* Population\_Manager::RipleysOutputPrb3 `[protected]` |

Referenced by CloseTheMonthlyRipleysOutputProbe(), OpenTheMonthlyRipleysOutputProbe(), and Run().

|  |
| --- |
| FILE\* Population\_Manager::RipleysOutputPrb4 `[protected]` |

Referenced by CloseTheMonthlyRipleysOutputProbe(), OpenTheMonthlyRipleysOutputProbe(), and Run().

|  |
| --- |
| FILE\* Population\_Manager::RipleysOutputPrb5 `[protected]` |

Referenced by CloseTheMonthlyRipleysOutputProbe(), OpenTheMonthlyRipleysOutputProbe(), and Run().

|  |
| --- |
| FILE\* Population\_Manager::RipleysOutputPrb6 `[protected]` |

Referenced by CloseTheMonthlyRipleysOutputProbe(), OpenTheMonthlyRipleysOutputProbe(), and Run().

|  |
| --- |
| FILE\* Population\_Manager::RipleysOutputPrb7 `[protected]` |

Referenced by CloseTheMonthlyRipleysOutputProbe(), OpenTheMonthlyRipleysOutputProbe(), and Run().

|  |
| --- |
| FILE\* Population\_Manager::RipleysOutputPrb8 `[protected]` |

Referenced by CloseTheMonthlyRipleysOutputProbe(), OpenTheMonthlyRipleysOutputProbe(), and Run().

|  |
| --- |
| FILE\* Population\_Manager::RipleysOutputPrb9 `[protected]` |

Referenced by CloseTheMonthlyRipleysOutputProbe(), OpenTheMonthlyRipleysOutputProbe(), and Run().

|  |
| --- |
| int Population\_Manager::SimH |

Referenced by Vole\_Base::CalculateCarryingCapacity(), Vole\_Base::CopyMyself(), Vole\_Population\_Manager::FindClosestFemale(), Vole\_Population\_Manager::FindClosestMale(), Vole\_Population\_Manager::Init(), Vole\_Population\_Manager::InSquare(), Vole\_Population\_Manager::ListClosestFemales(), Vole\_Population\_Manager::ListClosestMales(), Population\_Manager(), Probe(), Vole\_Female::st\_Lactating(), Vole\_Population\_Manager::SupplyHowManyVoles(), Vole\_Population\_Manager::SupplyInOlderTerr(), Vole\_Population\_Manager::SupplyOlderFemales(), SupplySimH(), Vole\_Population\_Manager::SupplyVoleList(), TPredator\_Population\_Manager::TPredator\_Population\_Manager(), and Vole\_Base::Vole\_Base().

|  |
| --- |
| int Population\_Manager::SimW |

Referenced by Vole\_Base::CalculateCarryingCapacity(), Vole\_Base::CopyMyself(), Vole\_Population\_Manager::FindClosestFemale(), Vole\_Population\_Manager::FindClosestMale(), Vole\_Population\_Manager::Init(), Vole\_Population\_Manager::InSquare(), Vole\_Population\_Manager::ListClosestFemales(), Vole\_Population\_Manager::ListClosestMales(), Population\_Manager(), Probe(), Vole\_Female::st\_Lactating(), Vole\_Population\_Manager::SupplyHowManyVoles(), Vole\_Population\_Manager::SupplyInOlderTerr(), Vole\_Population\_Manager::SupplyOlderFemales(), SupplySimW(), Vole\_Population\_Manager::SupplyVoleList(), TPredator\_Population\_Manager::TPredator\_Population\_Manager(), and Vole\_Base::Vole\_Base().

|  |
| --- |
| IntArray100 Population\_Manager::StateList `[protected]` |

Referenced by DoLast(), and SupplyStateList().

|  |
| --- |
| AnsiString Population\_Manager::StateNames[100] `[protected]` |

Referenced by Vole\_Population\_Manager::Init(), and SupplyStateNames().

|  |
| --- |
| unsigned Population\_Manager::StateNamesLength `[protected]` |

Referenced by Population\_Manager(), and SupplyStateNamesLength().

|  |
| --- |
| FILE\* Population\_Manager::TestFile `[protected]` |

Reimplemented in Vole\_Population\_Manager.

Referenced by WriteToTestFile().

|  |
| --- |
| FILE\* Population\_Manager::TestFile2 `[protected]` |

Reimplemented in Vole\_Population\_Manager.

Referenced by WriteToTest2File().

|  |
| --- |
| vector< TListOfAnimals > Population\_Manager::TheArray `[protected]` |

Referenced by Vole\_Population\_Manager::Catastrophe(), Vole\_Population\_Manager::CreateObjects(), TPredator\_Population\_Manager::CreateObjects(), Vole\_Population\_Manager::CreateObjects\_Init(), Vole\_Population\_Manager::DoFirst(), DoLast(), FindClosest(), Vole\_Population\_Manager::FindRandomMale(), Vole\_Population\_Manager::GeneticsResultsOutput(), Vole\_Population\_Manager::ImpactedProbe(), TPredator\_Population\_Manager::InOtherTerritory(), IsLast(), LOG(), Population\_Manager(), Probe(), TPredator\_Population\_Manager::Run(), Run(), Vole\_Population\_Manager::SendMessage(), Shuffle(), SortState(), SortX(), SortXIndex(), SortY(), TPredator\_Population\_Manager::StepFinished(), StepFinished(), SupplyListIndexSize(), SupplyListSize(), SupplyLocXY(), SupplyState(), Vole\_Population\_Manager::TheCIPEGridOutputProbe(), Vole\_Population\_Manager::TheReallyBigOutputProbe(), Vole\_Population\_Manager::TheRipleysOutputProbe(), TPredator\_Population\_Manager::TPredator\_Population\_Manager(), Vole\_Population\_Manager::Vole\_Population\_Manager(), and ~Population\_Manager().

|  |
| --- |
| probe\_data\* Population\_Manager::TheProbe[100] |

Referenced by ImpactProbeReport(), ProbeFileInput(), and ProbeReport().

---

The documentation for this class was generated from the following files:

- PopulationManager.h- PopulationManager.cpp

---

Generated on Thu Jan 22 14:13:46 2009 for ALMaSS ODDox by 
 1.5.6 
