# Supplementary material for: Predation and fragmentation portrayed in the statistical structure of prey time series
Source: BMC Ecol. 2009 May 6;9:10. doi: 10.1186/1472-6785-9-10 (PMC2689204; doi:10.1186/1472-6785-9-10)
Supplement: Additional file 2 — Voles and related classes ODDox Documentation. ODDox documentation of the agent-based model (ALMaSS) applied by Hendrichsen et al. The documentation is started by activating main.html. [file 1472-6785-9-10-S2.zip › Vole_ODDox/class_roe_deer_info.html]

ALMaSS ODDox: RoeDeerInfo Class Reference

- Main Page
- Related Pages
- Classes
- Files

- Alphabetical List
- Class List
- Class Hierarchy
- Class Members

# RoeDeerInfo Class Reference

`#include <PopulationManager.h>`

Inheritance diagram for RoeDeerInfo:

List of all members.

---

## Detailed Description

Part of the basic ALMaSS system (obselete).

Communicates the range centre, age and size of animals to other objects

|  |
| --- |
|  |
| Public Attributes | |
| int | m\_Age |
| unsigned | m\_OldRange\_x |
| unsigned | m\_OldRange\_y |
| unsigned | m\_Range\_x |
| unsigned | m\_Range\_y |
| int | m\_Size |

---

## Member Data Documentation

|  |
| --- |
| int RoeDeerInfo::m\_Age |

|  |
| --- |
| unsigned RoeDeerInfo::m\_OldRange\_x |

|  |
| --- |
| unsigned RoeDeerInfo::m\_OldRange\_y |

|  |
| --- |
| unsigned RoeDeerInfo::m\_Range\_x |

|  |
| --- |
| unsigned RoeDeerInfo::m\_Range\_y |

|  |
| --- |
| int RoeDeerInfo::m\_Size |

---

The documentation for this class was generated from the following file:

- PopulationManager.h

---

Generated on Thu Jan 22 14:13:46 2009 for ALMaSS ODDox by 
 1.5.6 
