# Supplementary material for: Predation and fragmentation portrayed in the statistical structure of prey time series
Source: BMC Ecol. 2009 May 6;9:10. doi: 10.1186/1472-6785-9-10 (PMC2689204; doi:10.1186/1472-6785-9-10)
Supplement: Additional file 2 — Voles and related classes ODDox Documentation. ODDox documentation of the agent-based model (ALMaSS) applied by Hendrichsen et al. The documentation is started by activating main.html. [file 1472-6785-9-10-S2.zip › Vole_ODDox/class_set_aside.html]

ALMaSS ODDox: SetAside Class Reference

- Main Page
- Related Pages
- Classes
- Files

- Alphabetical List
- Class List
- Class Hierarchy
- Class Members

# SetAside Class Reference

`#include <setaside.h>`

Inheritance diagram for SetAside:

List of all members.

---

## Detailed Description

Rotational set-aside management class   
.

setaside.h::SetAsideToDo is the list of things that a farmer can do if he is has rotational setaside, at least following this basic plan. For setaside this list is pretty short, because he can't do much with standard setaside (rules as of DK, 2000). So all we have to do is figure out when to do the different things using SetAside::Do Once we have done some kind of management, then an event is triggered and the fact that this particular management is done is registered with the particular polygon. This information is available for any ALMaSS components to inspect - e.g. animals & birds.

|  |
| --- |
|  |
| Public Member Functions | |
| bool | Do (Farm \*a\_farm, LE \*a\_field, FarmEvent \*a\_ev) |
|  | The one and only method for a crop management plan. All farm actions go through here. |
|  | SetAside () |

---

## Constructor & Destructor Documentation

|  |  |  |  |  |
| --- | --- | --- | --- | --- |
| SetAside::SetAside | ( |  | ) | `[inline]` |

References Crop::m\_first\_date.

```
00085   {
00086       m_first_date=g_date->DayInYear(1,1); // was 30,6
00087   }
```

---

## Member Function Documentation

|  |  |  |  |
| --- | --- | --- | --- |
| bool SetAside::Do | ( | Farm \* | *a\_farm*, |
|  |  | LE \* | *a\_field*, |
|  |  | FarmEvent \* | *a\_ev* |  |
|  | ) |  |  | `[virtual]` |

The one and only method for a crop management plan. All farm actions go through here.

Called every time something is done to the crop by the farmer in the first instance it is always called with m\_ev->todo set to start, but susequently will be called whenever the farmer wants to carry out a new operation.   
This method details all the management and relationships between operations necessary to grow and ALMaSS crop - in this case rotational setaside.

Reimplemented from Crop.

References Farm::CattleIsOut(), Farm::CattleOut(), cfg\_setaside\_glyphosate(), Farm::CutToHay(), Farm::CutToSilage(), Farm::DoIt(), Farm::Glyphosate(), Farm::IsStockFarmer(), Crop::m\_ev, Crop::m\_farm, Crop::m\_field, Crop::m\_first\_date, FarmEvent::m\_first\_year, Crop::m\_last\_date, FarmEvent::m\_lock, FarmEvent::m\_startday, FarmEvent::m\_todo, sa\_cattle\_is\_out, sa\_cattle\_out, sa\_cut\_to\_hay, sa\_cut\_to\_silage, sa\_glyphosate, sa\_start, sa\_wait, and Crop::SimpleEvent().

Referenced by Farm::LeSwitch().

```
00066 {
00067   m_farm      = a_farm;
00068   m_field     = a_field;
00069   m_ev        = a_ev;
00070   int d1      = 0;
00071   int noDates = 3;
00072   bool done   = false;
00073 
00074   switch ( m_ev->m_todo ) {
00075   case sa_start:
00076     // Special for set-aside:
00077     m_field->SetVegPatchy( true );
00078     // Set up the date management stuff
00079     // Could save the start day in case it is needed later
00080     // m_field->m_startday = m_ev->m_startday;
00081     m_last_date=g_date->DayInYear(10,10);
00082     // Start and stop dates for all events after harvest
00083     m_field->SetMDates(0,0,g_date->DayInYear(30,6));
00084     // Determined by harvest date - used to see if at all possible
00085     m_field->SetMDates(1,0,g_date->DayInYear(25,8));
00086     m_field->SetMDates(0,1,g_date->DayInYear(1,9));
00087     m_field->SetMDates(1,1,g_date->DayInYear(15,9));
00088     m_field->SetMDates(0,2,g_date->DayInYear(1,9));
00089     m_field->SetMDates(1,2,g_date->DayInYear(10,10));
00090     // Check the next crop for early start, unless it is a spring crop
00091     // in which case we ASSUME that no checking is necessary!!!!
00092     // So DO NOT implement a crop that runs over the year boundary
00093     if (m_ev->m_startday>g_date->DayInYear(1,7)) {
00094       if (m_field->GetMDates(0,0) >=m_ev->m_startday) {
00095         g_msg->Warn( WARN_BUG, "Setaside::Do(): "
00096                      "Harvest too late for the next crop to start!!!", "" );
00097         exit( 1 );
00098       }
00099       // Now fix any late finishing problems
00100       for (int i=0; i<noDates; i++) {
00101         if  (m_field->GetMDates(0,i)>=m_ev->m_startday)
00102           m_field->SetMDates(0,i,m_ev->m_startday-1);
00103         if  (m_field->GetMDates(1,i)>=m_ev->m_startday)
00104           m_field->SetMDates(1,i,m_ev->m_startday-1);
00105       }
00106     }
00107 
00108     // Now no operations can be timed after the start of the next crop.
00109     if ( ! m_ev->m_first_year ) {
00110       // Are we before July 1st?
00111       d1 = g_date->OldDays() + g_date->DayInYear( 1,7 );
00112       if (g_date->Date() < d1) {
00113         // Yes, too early. We assumme this is because the last crop was late
00114         g_msg->Warn( WARN_BUG, "Setaside::Do(): "
00115                      "Crop start attempt between 1st Jan & 1st July", "" );
00116         exit( 1 );
00117       } else {
00118         d1 = g_date->OldDays() + m_first_date+365; // Add 365 for spring crop
00119         if (g_date->Date() > d1) {
00120           // Yes too late - should not happen - raise an error
00121           g_msg->Warn( WARN_BUG, "SetAside::Do(): "
00122                        "Crop start attempt after last possible start date",
00123                        "" );
00124           exit( 1 );
00125         }
00126       }
00127     }
00128 
00129     // New begin.
00130     d1 = g_date->OldDays() + m_first_date;
00131 
00132     if ( ! m_ev->m_first_year ) {
00133         d1 +=365 ;
00134     }
00135     if ( g_date->Date() > d1 ) {
00136       d1 = g_date->Date();
00137     }
00138 
00139     // ***CJT*** altered 16 August 2004 to be more similar to non-managed set-aside
00140           SimpleEvent( d1, sa_wait, false );
00141           break;
00142     /*
00143         SimpleEvent( d1, sa_cut_to_hay, false );
00144     break;
00145     */
00146   case sa_cut_to_hay:
00147     if (!m_farm->CutToHay( m_field, 0.0,
00148          g_date->DayInYear( 30, 7 ) - g_date->DayInYear())) {
00149       SimpleEvent( g_date->Date() + 1, sa_cut_to_hay, false );
00150       break;
00151     }
00152     if ( !m_farm->IsStockFarmer())
00153     {
00154       SimpleEvent( g_date->Date() + 1, sa_wait, false );
00155       break;
00156     }
00157     SimpleEvent( g_date->OldDays() + m_field->GetMDates(0,1),
00158                    sa_cattle_out, false );
00159     break;
00160 
00161   case sa_cattle_out:
00162     if ( m_ev->m_lock || m_farm->DoIt( 15 ))
00163     {
00164       if (!m_farm->CattleOut( m_field, 0.0,
00165            m_field->GetMDates(1,1) - g_date->DayInYear())) {
00166         SimpleEvent( g_date->Date() + 1, sa_cattle_out, true );
00167         break;
00168       }
00169             SimpleEvent( g_date->Date() + 1, sa_cattle_is_out, false );
00170       break;
00171     }
00172     SimpleEvent( g_date->OldDays() + m_field->GetMDates(0,1),
00173                  sa_cut_to_silage, false );
00174     break;
00175 
00176   case sa_cattle_is_out:
00177     if (!m_farm->CattleIsOut( m_field, 0.0,
00178          m_field->GetMDates(1,2) - g_date->DayInYear(),m_field->GetMDates(1,2)))
00179     {
00180       SimpleEvent( g_date->Date() + 1, sa_cattle_is_out, false );
00181       break;
00182     }
00183     SimpleEvent( g_date->Date() + 1, sa_wait, false );
00184     break;
00185 
00186   case sa_cut_to_silage:
00187     if ( m_ev->m_lock || m_farm->DoIt( 15 ))
00188     {
00189       if (!m_farm->CutToSilage( m_field, 0.0,
00190            m_field->GetMDates(1,0) - g_date->DayInYear())) {
00191         SimpleEvent( g_date->Date() + 1, sa_cut_to_silage, true );
00192         break;
00193       }
00194     }
00195     SimpleEvent( g_date->Date() + 1, sa_wait, false );
00196     break;
00197 
00198   case sa_glyphosate:
00199           if (cfg_setaside_glyphosate.value()==-1) {
00200             SimpleEvent( g_date->Date() + 1, sa_wait, false );
00201                 break;
00202           }
00203           else if (g_date->DayInYear()>cfg_setaside_glyphosate.value()) {
00204             SimpleEvent( g_date->Date() + 1, sa_wait, false );
00205                 break;
00206           }
00207                   else if (g_date->DayInYear()==cfg_setaside_glyphosate.value()) {
00208                     m_farm->Glyphosate(m_field,0,0);
00209                         SimpleEvent( g_date->Date() + 1, sa_wait, false );
00210                         break;
00211                   }
00212          break;
00213 
00214   case sa_wait:
00215 
00216         d1= g_date->DayInYear();
00217         if ((g_date->DayInYear()>=cfg_setaside_glyphosate.value()) && (g_date->DayInYear()<=cfg_setaside_glyphosate.value()+20)) {
00218                 m_farm->Glyphosate(m_field,0,0);
00219         }
00220         // Cannot terminate too early otherwise will cause a rotation loop
00221 
00222     if ((g_date->DayInYear()-g_date->DayInYear(2,7))>=0)
00223     {
00224       // Special for set-aside:
00225       m_field->SetVegPatchy( false );
00226       done = true;
00227     }
00228     else // queue this up again
00229     SimpleEvent( g_date->Date() + 1, sa_wait, true );
00230     break;
00231 
00232   default:
00233     g_msg->Warn( WARN_BUG, "SetAside::Do(): "
00234                  "Unknown event type! ", "" );
00235     exit( 1 );
00236   }
00237 
00238   return done;
00239 }
```

---

The documentation for this class was generated from the following files:

- setaside.h- setaside.cpp

---

Generated on Thu Jan 22 14:13:46 2009 for ALMaSS ODDox by 
 1.5.6 
