# Supplementary material for: Predation and fragmentation portrayed in the statistical structure of prey time series
Source: BMC Ecol. 2009 May 6;9:10. doi: 10.1186/1472-6785-9-10 (PMC2689204; doi:10.1186/1472-6785-9-10)
Supplement: Additional file 2 — Voles and related classes ODDox Documentation. ODDox documentation of the agent-based model (ALMaSS) applied by Hendrichsen et al. The documentation is started by activating main.html. [file 1472-6785-9-10-S2.zip › Vole_ODDox/class_t_animal-members.html]

ALMaSS ODDox: Member List

- Main Page
- Related Pages
- Classes
- Files

- Alphabetical List
- Class List
- Class Hierarchy
- Class Members

# TAnimal Member List

This is the complete list of members for TAnimal, including all inherited members.

|  |  |  |
| --- | --- | --- |
| BeginStep(void) | TAnimal | `[inline, virtual]` |
| CheckManagement(void) | TAnimal |  |
| CheckManagementXY(int x, int y) | TAnimal |  |
| CopyMyself() | TAnimal | `[inline, virtual]` |
| CurrentStateNo | TALMaSSObject |  |
| Dying() | TAnimal | `[inline, virtual]` |
| EndStep(void) | TAnimal | `[inline, virtual]` |
| KillThis() | TAnimal | `[inline, virtual]` |
| m\_Location\_x | TAnimal | `[protected]` |
| m\_Location\_y | TAnimal | `[protected]` |
| m\_OurLandscape | TAnimal | `[protected]` |
| OnArrayBoundsError() | TALMaSSObject |  |
| OnFarmEvent(FarmToDo) | TAnimal | `[inline, virtual]` |
| SetX(int a\_x) | TAnimal | `[inline]` |
| SetY(int a\_y) | TAnimal | `[inline]` |
| Step(void) | TAnimal | `[inline, virtual]` |
| StepDone | TALMaSSObject |  |
| Supply\_m\_Location\_x() | TAnimal | `[inline]` |
| Supply\_m\_Location\_y() | TAnimal | `[inline]` |
| SupplyFarmOwnerRef() | TAnimal |  |
| SupplyPosition() | TAnimal |  |
| TALMaSSObject() | TALMaSSObject |  |
| TAnimal(int x, int y, Landscape \*L) | TAnimal |  |
| WhatState() | TAnimal | `[inline, virtual]` |
| ~TALMaSSObject() | TALMaSSObject | `[virtual]` |

---

Generated on Thu Jan 22 14:13:47 2009 for ALMaSS ODDox by 
 1.5.6 
