# Supplementary material for: Predation and fragmentation portrayed in the statistical structure of prey time series
Source: BMC Ecol. 2009 May 6;9:10. doi: 10.1186/1472-6785-9-10 (PMC2689204; doi:10.1186/1472-6785-9-10)
Supplement: Additional file 2 — Voles and related classes ODDox Documentation. ODDox documentation of the agent-based model (ALMaSS) applied by Hendrichsen et al. The documentation is started by activating main.html. [file 1472-6785-9-10-S2.zip › Vole_ODDox/class_t_animal.html]

ALMaSS ODDox: TAnimal Class Reference

- Main Page
- Related Pages
- Classes
- Files

- Alphabetical List
- Class List
- Class Hierarchy
- Class Members

# TAnimal Class Reference

`#include <PopulationManager.h>`

Inheritance diagram for TAnimal:

List of all members.

---

## Detailed Description

The base class for all ALMaSS animal classes.

Includes all the functionality required to be handled by classes derived from Population\_Manager, hence a number of empty methods that MUST be reimplemented in descendent classes e.g. CopyMyself()

|  |
| --- |
|  |
| Public Member Functions | |
| virtual void | BeginStep (void) |
| void | CheckManagement (void) |
| void | CheckManagementXY (int x, int y) |
| virtual void | CopyMyself () |
| virtual void | Dying () |
| virtual void | EndStep (void) |
| virtual void | KillThis () |
| virtual bool | OnFarmEvent (FarmToDo) |
| void | SetX (int a\_x) |
| void | SetY (int a\_y) |
| virtual void | Step (void) |
| int | Supply\_m\_Location\_x () |
| int | Supply\_m\_Location\_y () |
| unsigned | SupplyFarmOwnerRef () |
| AnimalPosition | SupplyPosition () |
|  | TAnimal (int x, int y, Landscape \*L) |
| virtual int | WhatState () |
| Protected Attributes | |
| int | m\_Location\_x |
| int | m\_Location\_y |
| Landscape \* | m\_OurLandscape |

---

## Constructor & Destructor Documentation

|  |  |  |  |
| --- | --- | --- | --- |
| TAnimal::TAnimal | ( | int | *x*, |
|  |  | int | *y*, |
|  |  | Landscape \* | *L* |  |
|  | ) |  |  |  |

TAnimal Constructor

References m\_Location\_x, m\_Location\_y, and m\_OurLandscape.

```
01242                                               {
01243   m_OurLandscape = L;
01244   m_Location_x = x;
01245   m_Location_y = y;
01246 }
```

---

## Member Function Documentation

|  |  |  |  |  |  |
| --- | --- | --- | --- | --- | --- |
| virtual void TAnimal::BeginStep | ( | void |  | ) | `[inline, virtual]` |

Reimplemented from TALMaSSObject.

Reimplemented in TPredator, Weasel, Owl, Vole\_Base, Vole\_Male, and Vole\_Female.

```
00162                                  {
00163   }
```

|  |  |  |  |  |  |
| --- | --- | --- | --- | --- | --- |
| void TAnimal::CheckManagement | ( | void |  | ) |  |

Checks to see if there has been a management event at the TAnimals' x,y location. If so calls an event handler to handle the management event.

References m\_Location\_x, m\_Location\_y, m\_OurLandscape, OnFarmEvent(), and sleep\_all\_day.

Referenced by Vole\_Male::EndStep(), and Vole\_Female::EndStep().

```
01279                                     {
01280   FarmToDo event;
01281   int i = 0;
01282   while ( ( event = ( FarmToDo )m_OurLandscape->SupplyLastTreatment( m_Location_x, m_Location_y, & i ) ) != sleep_all_day ) {
01283     if ( OnFarmEvent( event ) ) break;
01284   }
01285 }
```

|  |  |  |  |
| --- | --- | --- | --- |
| void TAnimal::CheckManagementXY | ( | int | *x*, |
|  |  | int | *y* |  |
|  | ) |  |  |  |

Checks to see if there has been a management event at the x,y location. If so calls an event handler to handle the management event.

References m\_OurLandscape, OnFarmEvent(), and sleep\_all\_day.

```
01292                                               {
01293   FarmToDo event;
01294   int i = 0;
01295   while ( ( event = ( FarmToDo )m_OurLandscape->SupplyLastTreatment( x, y, & i ) ) != sleep_all_day ) {
01296     OnFarmEvent( event );
01297   }
01298 }
```

|  |  |  |  |  |
| --- | --- | --- | --- | --- |
| virtual void TAnimal::CopyMyself | ( |  | ) | `[inline, virtual]` |

```
00145                             {
00146   };
```

|  |  |  |  |  |
| --- | --- | --- | --- | --- |
| virtual void TAnimal::Dying | ( |  | ) | `[inline, virtual]` |

References KillThis().

```
00171                        {
00172     KillThis();
00173   }
```

|  |  |  |  |  |  |
| --- | --- | --- | --- | --- | --- |
| virtual void TAnimal::EndStep | ( | void |  | ) | `[inline, virtual]` |

Reimplemented from TALMaSSObject.

Reimplemented in TPredator, Weasel, Owl, Vole\_Base, Vole\_Male, and Vole\_Female.

```
00166                                {
00167   }
```

|  |  |  |  |  |
| --- | --- | --- | --- | --- |
| virtual void TAnimal::KillThis | ( |  | ) | `[inline, virtual]` |

References TALMaSSObject::CurrentStateNo, and TALMaSSObject::StepDone.

Referenced by Owl::BeginStep(), Weasel::BeginStep(), and Dying().

```
00142                           {
00143     CurrentStateNo = -1; StepDone = true;
00144   };
```

|  |  |  |  |  |  |
| --- | --- | --- | --- | --- | --- |
| virtual bool TAnimal::OnFarmEvent | ( | FarmToDo |  | ) | `[inline, virtual]` |

Reimplemented in Vole\_Male, and Vole\_Female.

Referenced by CheckManagement(), and CheckManagementXY().

```
00176                                                    {
00177     return false;
00178   }
```

|  |  |  |  |  |  |
| --- | --- | --- | --- | --- | --- |
| void TAnimal::SetX | ( | int | *a\_x* | ) | `[inline]` |

References m\_Location\_x.

```
00152                        {
00153     m_Location_x = a_x;
00154   }
```

|  |  |  |  |  |  |
| --- | --- | --- | --- | --- | --- |
| void TAnimal::SetY | ( | int | *a\_y* | ) | `[inline]` |

References m\_Location\_y.

```
00155                        {
00156 
00157     m_Location_y = a_y;
00158 
00159   }
```

|  |  |  |  |  |  |
| --- | --- | --- | --- | --- | --- |
| virtual void TAnimal::Step | ( | void |  | ) | `[inline, virtual]` |

Reimplemented from TALMaSSObject.

Reimplemented in TPredator, Weasel, Owl, Vole\_Base, Vole\_Male, and Vole\_Female.

```
00164                             {
00165   }
```

|  |  |  |  |  |
| --- | --- | --- | --- | --- |
| int TAnimal::Supply\_m\_Location\_x | ( |  | ) | `[inline]` |

References m\_Location\_x.

Referenced by Vole\_Population\_Manager::GeneticsResultsOutput(), CompareX::operator()(), Vole\_Population\_Manager::TheCIPEGridOutputProbe(), Vole\_Population\_Manager::TheReallyBigOutputProbe(), and Vole\_Population\_Manager::TheRipleysOutputProbe().

```
00136                             {
00137     return m_Location_x;
00138   }
```

|  |  |  |  |  |
| --- | --- | --- | --- | --- |
| int TAnimal::Supply\_m\_Location\_y | ( |  | ) | `[inline]` |

References m\_Location\_y.

Referenced by CompareY::operator()(), Vole\_Population\_Manager::TheCIPEGridOutputProbe(), Vole\_Population\_Manager::TheReallyBigOutputProbe(), and Vole\_Population\_Manager::TheRipleysOutputProbe().

```
00139                             {
00140     return m_Location_y;
00141   }
```

|  |  |  |  |  |
| --- | --- | --- | --- | --- |
| unsigned TAnimal::SupplyFarmOwnerRef | ( |  | ) |  |

Provides the farmer reference for the location of a TAnimal

References m\_Location\_x, m\_Location\_y, and m\_OurLandscape.

```
01234                                      {
01235   return m_OurLandscape->SupplyFarmOwner( m_Location_x, m_Location_y );
01236 }
```

|  |  |  |  |  |
| --- | --- | --- | --- | --- |
| AnimalPosition TAnimal::SupplyPosition | ( |  | ) |  |

Provides the location of an animal in terms of x,y,elementtype and vegetation type

References AnimalPosition::m\_EleType, m\_Location\_x, m\_Location\_y, m\_OurLandscape, AnimalPosition::m\_VegType, AnimalPosition::m\_x, and AnimalPosition::m\_y.

Referenced by Vole\_Population\_Manager::DoFirst().

```
01220                                        {
01221   AnimalPosition SkP;
01222   SkP.m_x = m_Location_x;
01223   SkP.m_y = m_Location_y;
01224   SkP.m_EleType = m_OurLandscape->SupplyElementType( m_Location_x, m_Location_y );
01225   SkP.m_VegType = m_OurLandscape->SupplyVegType( m_Location_x, m_Location_y );
01226   return SkP;
01227 }
```

|  |  |  |  |  |
| --- | --- | --- | --- | --- |
| virtual int TAnimal::WhatState | ( |  | ) | `[inline, virtual]` |

Reimplemented in Vole\_Base.

Referenced by CompareState::operator()().

```
00168                           {
00169     return 0;
00170   }
```

---

## Member Data Documentation

|  |
| --- |
| int TAnimal::m\_Location\_x `[protected]` |

Referenced by Vole\_Female::BeginStep(), Owl::BeginStep(), Weasel::BeginStep(), Vole\_Male::CanFeed(), CheckManagement(), Vole\_Base::CopyMyself(), Vole\_Male::Dispersal(), Vole\_Female::Dispersal(), Vole\_Base::Escape(), Vole\_Male::FreeLocation(), Vole\_Female::FreeLocation(), Vole\_Base::MoveTo(), TPredator::OverlapMyTerritory(), Vole\_Male::SetLocation(), Vole\_Female::SetLocation(), SetX(), TPredator::st\_Dispersal(), Vole\_Base::st\_Dying(), Vole\_Male::st\_Eval\_n\_Explore(), Vole\_Female::st\_Evaluate\_n\_Explore(), Vole\_Female::st\_GiveBirth(), Vole\_Male::st\_Infanticide(), Vole\_Female::st\_Lactating(), Vole\_Female::st\_Mating(), TPredator::st\_Movement(), Vole\_Female::st\_Special\_Explore(), Supply\_m\_Location\_x(), SupplyFarmOwnerRef(), SupplyPosition(), Vole\_Base::SupplyX(), TAnimal(), and TPredator::TPredator().

|  |
| --- |
| int TAnimal::m\_Location\_y `[protected]` |

Referenced by Vole\_Female::BeginStep(), Owl::BeginStep(), Weasel::BeginStep(), Vole\_Male::CanFeed(), CheckManagement(), Vole\_Base::CopyMyself(), Vole\_Male::Dispersal(), Vole\_Female::Dispersal(), Vole\_Base::Escape(), Vole\_Male::FreeLocation(), Vole\_Female::FreeLocation(), Vole\_Base::MoveTo(), TPredator::OverlapMyTerritory(), Vole\_Male::SetLocation(), Vole\_Female::SetLocation(), SetY(), TPredator::st\_Dispersal(), Vole\_Base::st\_Dying(), Vole\_Male::st\_Eval\_n\_Explore(), Vole\_Female::st\_Evaluate\_n\_Explore(), Vole\_Female::st\_GiveBirth(), Vole\_Male::st\_Infanticide(), Vole\_Female::st\_Lactating(), Vole\_Female::st\_Mating(), TPredator::st\_Movement(), Vole\_Female::st\_Special\_Explore(), Supply\_m\_Location\_y(), SupplyFarmOwnerRef(), SupplyPosition(), Vole\_Base::SupplyY(), TAnimal(), and TPredator::TPredator().

|  |
| --- |
| Landscape\* TAnimal::m\_OurLandscape `[protected]` |

Referenced by Vole\_Base::AssessHabitat(), Vole\_Female::BeginStep(), Owl::BeginStep(), Weasel::BeginStep(), Vole\_Base::CalculateCarryingCapacity(), CheckManagement(), CheckManagementXY(), Vole\_Base::CopyMyself(), Vole\_Male::Dispersal(), Vole\_Male::EndStep(), Vole\_Female::EndStep(), Vole\_Base::MoveQuality(), Vole\_Male::OnFarmEvent(), Vole\_Female::OnFarmEvent(), Vole\_Female::st\_BecomeReproductive(), Vole\_Male::st\_Eval\_n\_Explore(), Vole\_Female::st\_Evaluate\_n\_Explore(), Vole\_Female::st\_GiveBirth(), Vole\_Female::st\_Lactating(), Vole\_Female::st\_Mating(), Vole\_Male::st\_Maturation(), Vole\_Female::st\_UpdateGestation(), Vole\_Male::Step(), Vole\_Female::Step(), SupplyFarmOwnerRef(), SupplyPosition(), TAnimal(), and TPredator::TPredator().

---

The documentation for this class was generated from the following files:

- PopulationManager.h- PopulationManager.cpp

---

Generated on Thu Jan 22 14:13:47 2009 for ALMaSS ODDox by 
 1.5.6 
