# Supplementary material for: Predation and fragmentation portrayed in the statistical structure of prey time series
Source: BMC Ecol. 2009 May 6;9:10. doi: 10.1186/1472-6785-9-10 (PMC2689204; doi:10.1186/1472-6785-9-10)
Supplement: Additional file 2 — Voles and related classes ODDox Documentation. ODDox documentation of the agent-based model (ALMaSS) applied by Hendrichsen et al. The documentation is started by activating main.html. [file 1472-6785-9-10-S2.zip › Vole_ODDox/class_t_a_l_ma_s_s_object.html]

ALMaSS ODDox: TALMaSSObject Class Reference

- Main Page
- Related Pages
- Classes
- Files

- Alphabetical List
- Class List
- Class Hierarchy
- Class Members

# TALMaSSObject Class Reference

`#include <PopulationManager.h>`

Inheritance diagram for TALMaSSObject:

List of all members.

---

## Detailed Description

The base class of all ALMaSS objects requiring Step code.

|  |
| --- |
|  |
| Public Member Functions | |
| virtual void | BeginStep (void) |
| virtual void | EndStep (void) |
| void | OnArrayBoundsError () |
| virtual void | Step (void) |
|  | TALMaSSObject () |
| virtual | ~TALMaSSObject () |
| Public Attributes | |
| int | CurrentStateNo |
| bool | StepDone |

---

## Constructor & Destructor Documentation

|  |  |  |  |  |
| --- | --- | --- | --- | --- |
| TALMaSSObject::TALMaSSObject | ( |  | ) |  |

TALMaSSObject Constructor

References CurrentStateNo, and StepDone.

```
01254                              {
01255 #ifdef __CJTDebug_5
01256   AmAlive = 0xDEADC0DE;
01257 #endif
01258   StepDone = false;
01259   CurrentStateNo = 0;
01260 }
```

|  |  |  |  |  |
| --- | --- | --- | --- | --- |
| TALMaSSObject::~TALMaSSObject | ( |  | ) | `[virtual]` |

TALMaSSObject Destructor

```
01268                               {
01269 #ifdef __CJTDebug_5
01270   AmAlive = 0;
01271 #endif
01272 }
```

---

## Member Function Documentation

|  |  |  |  |  |  |
| --- | --- | --- | --- | --- | --- |
| virtual void TALMaSSObject::BeginStep | ( | void |  | ) | `[inline, virtual]` |

Reimplemented in TAnimal, TPredator, Weasel, Owl, Vole\_Base, Vole\_Male, and Vole\_Female.

```
00072                                  {
00073   }
```

|  |  |  |  |  |  |
| --- | --- | --- | --- | --- | --- |
| virtual void TALMaSSObject::EndStep | ( | void |  | ) | `[inline, virtual]` |

Reimplemented in TAnimal, TPredator, Weasel, Owl, Vole\_Base, Vole\_Male, and Vole\_Female.

```
00076                                {
00077   }
```

|  |  |  |  |  |
| --- | --- | --- | --- | --- |
| void TALMaSSObject::OnArrayBoundsError | ( |  | ) |  |

```
01302                                        {
01303   exit( 1 );
01304 }
```

|  |  |  |  |  |  |
| --- | --- | --- | --- | --- | --- |
| virtual void TALMaSSObject::Step | ( | void |  | ) | `[inline, virtual]` |

Reimplemented in TAnimal, TPredator, Weasel, Owl, Vole\_Base, Vole\_Male, and Vole\_Female.

```
00074                             {
00075   }
```

---

## Member Data Documentation

|  |
| --- |
| int TALMaSSObject::CurrentStateNo |

Referenced by Vole\_Population\_Manager::FindRandomMale(), TAnimal::KillThis(), Vole\_Base::st\_Dying(), Vole\_Male::Step(), Owl::Step(), Weasel::Step(), and TALMaSSObject().

|  |
| --- |
| bool TALMaSSObject::StepDone |

Referenced by Vole\_Male::BeginStep(), Vole\_Female::BeginStep(), TAnimal::KillThis(), Vole\_Male::Step(), Vole\_Female::Step(), Owl::Step(), Weasel::Step(), and TALMaSSObject().

---

The documentation for this class was generated from the following files:

- PopulationManager.h- PopulationManager.cpp

---

Generated on Thu Jan 22 14:13:46 2009 for ALMaSS ODDox by 
 1.5.6 
