# Supplementary material for: Predation and fragmentation portrayed in the statistical structure of prey time series
Source: BMC Ecol. 2009 May 6;9:10. doi: 10.1186/1472-6785-9-10 (PMC2689204; doi:10.1186/1472-6785-9-10)
Supplement: Additional file 2 — Voles and related classes ODDox Documentation. ODDox documentation of the agent-based model (ALMaSS) applied by Hendrichsen et al. The documentation is started by activating main.html. [file 1472-6785-9-10-S2.zip › Vole_ODDox/class_t_predator.html]

ALMaSS ODDox: TPredator Class Reference

- Main Page
- Related Pages
- Classes
- Files

- Alphabetical List
- Class List
- Class Hierarchy
- Class Members

# TPredator Class Reference

`#include <Predators.H>`

Inheritance diagram for TPredator:

List of all members.

---

## Detailed Description

The base class for predators encompsassing all their general behaviours.

Defines simple predators that are really nothing more than moving mortality probabilities of different sizes and strengths. Breeding is once a year if enough prey are eaten, and death occurs if a starvation criteria is reached. The last individual cannot die so the population can never go extinct. If predators do not eat for a definable number of hunts then they may disperse, otherwise they move only locally. Different types of predators can be defined in the same simulation by defining the Weasel and Owl classes using configuration variables to create e.g. specialist or generalist predators, with different movement patterns, hunting efficiency and numerical responses.

|  |
| --- |
|  |
| Public Member Functions | |
| virtual void | BeginStep (void) |
| virtual void | EndStep (void) |
| bool | OverlapMyTerritory (unsigned x, unsigned y) |
| virtual void | st\_Dispersal () |
| virtual int | st\_Hunting () |
| virtual void | st\_Movement () |
| virtual void | Step (void) |
|  | TPredator (Vole\_Population\_Manager \*ThePrey, int p\_x, int p\_y, Landscape \*p\_L, TPredator\_Population\_Manager \*p\_PPM) |
|  | ~TPredator () |
| Protected Attributes | |
| vector< Vole\_Base \* > \* | CurrentPrey |
| TTypeOfPredatorState | CurrentPState |
| unsigned | m\_DispersalMax |
| unsigned | m\_FailureCount |
| bool | m\_HaveTerritory |
| unsigned | m\_HomeRange |
| int | m\_KillEfficiency |
| int | m\_kills\_this\_season |
| unsigned | m\_NoFailuresBeforeDispersal |
| TPredator\_Population\_Manager \* | m\_OurPopulationManager |
| Vole\_Population\_Manager \* | m\_Prey |
| int | m\_Search\_x |
| int | m\_Search\_y |
| unsigned | m\_SearchArea |
| int | PreyResponse1 |
| int | PreyResponse2 |
| int | SimH |
| int | SimW |
| unsigned | SpeciesID |

---

## Constructor & Destructor Documentation

|  |  |  |  |
| --- | --- | --- | --- |
| TPredator::TPredator | ( | Vole\_Population\_Manager \* | *ThePrey*, |
|  |  | int | *p\_x*, |
|  |  | int | *p\_y*, |
|  |  | Landscape \* | *p\_L*, |
|  |  | TPredator\_Population\_Manager \* | *p\_PPM* |  |
|  | ) |  |  |  |

Tpredator constructor

References CurrentPState, m\_DispersalMax, m\_FailureCount, m\_HaveTerritory, m\_HomeRange, m\_KillEfficiency, m\_kills\_this\_season, TAnimal::m\_Location\_x, TAnimal::m\_Location\_y, m\_NoFailuresBeforeDispersal, TAnimal::m\_OurLandscape, m\_OurPopulationManager, m\_Prey, m\_Search\_x, m\_Search\_y, m\_SearchArea, PreyResponse1, PreyResponse2, SimH, SimW, and tops\_InitialState.

```
00227                                                           : TAnimal(p_x,p_y,p_L)
00228 {
00229 
00230     CurrentPState=tops_InitialState;
00231     m_KillEfficiency=0;
00232     m_kills_this_season=0;
00233     m_FailureCount=0;
00234     m_NoFailuresBeforeDispersal=5; // Default
00235     m_OurPopulationManager=p_PPM;
00236     m_Prey = ThePrey;
00237     m_Search_x=m_Location_x;
00238     m_Search_y=m_Location_y;
00239     m_HomeRange=0;
00240     m_HaveTerritory=false;
00241     m_SearchArea=0;
00242     m_DispersalMax=0;
00243     SimH=m_OurLandscape->SupplySimAreaHeight();
00244     SimW=m_OurLandscape->SupplySimAreaWidth();
00245     PreyResponse1=0;
00246     PreyResponse2=0;
00247 
00248 }
```

|  |  |  |  |  |
| --- | --- | --- | --- | --- |
| TPredator::~TPredator | ( |  | ) |  |

```
00252 {
00253 }
```

---

## Member Function Documentation

|  |  |  |  |  |  |
| --- | --- | --- | --- | --- | --- |
| virtual void TPredator::BeginStep | ( | void |  | ) | `[inline, virtual]` |

Reimplemented from TAnimal.

Reimplemented in Weasel, and Owl.

```
00118 {}
```

|  |  |  |  |  |  |
| --- | --- | --- | --- | --- | --- |
| virtual void TPredator::EndStep | ( | void |  | ) | `[inline, virtual]` |

Reimplemented from TAnimal.

Reimplemented in Weasel, and Owl.

```
00120 {}
```

|  |  |  |  |
| --- | --- | --- | --- |
| bool TPredator::OverlapMyTerritory | ( | unsigned | *x*, |
|  |  | unsigned | *y* |  |
|  | ) |  |  |  |

References m\_HomeRange, TAnimal::m\_Location\_x, TAnimal::m\_Location\_y, SimH, and SimW.

Referenced by TPredator\_Population\_Manager::InOtherTerritory().

```
00302 {
00303    // ensure we can't go negative
00304    x+=SimW;
00305    y+=SimH;
00306    unsigned mx = m_Location_x+SimW;
00307    unsigned my = m_Location_y+SimH;
00308    // most likely that it is not in so test for false
00309    if (x<mx-m_HomeRange) return false;
00310     else if (x>=mx+m_HomeRange) return false;
00311       else if (y<my-m_HomeRange) return false;
00312         else if (y>=my+m_HomeRange) return false;
00313          else return true;
00314 }
```

|  |  |  |  |  |
| --- | --- | --- | --- | --- |
| void TPredator::st\_Dispersal | ( |  | ) | `[virtual]` |

References TPredator\_Population\_Manager::InOtherTerritory(), m\_DispersalMax, m\_HaveTerritory, TAnimal::m\_Location\_x, TAnimal::m\_Location\_y, m\_OurPopulationManager, SimH, SimW, and SpeciesID.

Referenced by Owl::Step(), and Weasel::Step().

```
00319 {
00320 
00321   // Moves the home range to an area where it does not overlap with
00322   // a conspecific
00323   bool found=false;
00324   unsigned Count=0;
00325   while ((!found)&&(Count<100))
00326   {
00327     // Simple random walk
00328     Count++;
00329     m_Location_x=((m_Location_x+(random(2*m_DispersalMax)-m_DispersalMax)))%SimW;
00330     m_Location_y=((m_Location_y+(random(2*m_DispersalMax)-m_DispersalMax)))%SimH;
00331     if (!m_OurPopulationManager->InOtherTerritory(SpeciesID,m_Location_x,
00332                                                                  m_Location_y,this))
00333     {
00334       m_HaveTerritory=true;
00335       found=true;
00336     }
00337   }
00338 }
```

|  |  |  |  |  |
| --- | --- | --- | --- | --- |
| int TPredator::st\_Hunting | ( |  | ) | `[virtual]` |

References CurrentPrey, m\_KillEfficiency, m\_kills\_this\_season, m\_Prey, m\_Search\_x, m\_Search\_y, m\_SearchArea, and Vole\_Population\_Manager::SupplyVoleList().

Referenced by Owl::Step(), and Weasel::Step().

```
00258 {
00259    unsigned kills=0;
00260    // count days since last kill
00261    // Takes the Search_x, Search_y, SearchArea. Applies KillEfficiency to
00262    // all voles defined by this square
00263    CurrentPrey=m_Prey->SupplyVoleList(m_Search_x,m_Search_y,m_SearchArea);
00264    //int s=CurrentPrey->size();  // **CJT** to help with debug
00265    for (unsigned i=0; i<CurrentPrey->size(); i++)
00266    {
00267      if (random(1000)<m_KillEfficiency)
00268      {
00269       (*CurrentPrey)[i]->OnKilled();
00270       kills++;
00271      }
00272    }
00273    // Must tidy up here because m_Prey cannot know when to do it
00274    CurrentPrey->clear();
00275    delete CurrentPrey;
00276    // record the kills
00277    m_kills_this_season+=kills;
00278    return kills;
00279 }
```

|  |  |  |  |  |
| --- | --- | --- | --- | --- |
| void TPredator::st\_Movement | ( |  | ) | `[virtual]` |

References m\_HomeRange, TAnimal::m\_Location\_x, TAnimal::m\_Location\_y, m\_Search\_x, m\_Search\_y, m\_SearchArea, SimH, and SimW.

Referenced by Owl::Step(), and Weasel::Step().

```
00285 {
00286    // Can relocate search_x & search_y to be up somewhere in the homerange
00287    // but must have all the square in the HomeRange
00288    // 1. Drift a bit
00289    m_Location_x+=random(3)-1;
00290    m_Location_y+=random(3)-1;
00291    m_Location_x=(SimW+m_Location_x)%SimW;
00292    m_Location_y=(SimH+m_Location_y)%SimH;
00293    // 2. determine search area
00294    int max_dist=m_HomeRange-m_SearchArea;
00295    m_Search_x=(m_Location_x+random(max_dist))%SimW;
00296    m_Search_y=(m_Location_y+random(max_dist))%SimH;
00297 }
```

|  |  |  |  |  |  |
| --- | --- | --- | --- | --- | --- |
| virtual void TPredator::Step | ( | void |  | ) | `[inline, virtual]` |

Reimplemented from TAnimal.

Reimplemented in Weasel, and Owl.

```
00119 {}
```

---

## Member Data Documentation

|  |
| --- |
| vector<Vole\_Base\*>\* TPredator::CurrentPrey `[protected]` |

Referenced by st\_Hunting().

|  |
| --- |
| TTypeOfPredatorState TPredator::CurrentPState `[protected]` |

Referenced by Owl::Step(), Weasel::Step(), and TPredator().

|  |
| --- |
| unsigned TPredator::m\_DispersalMax `[protected]` |

Referenced by Owl::Owl(), st\_Dispersal(), TPredator(), and Weasel::Weasel().

|  |
| --- |
| unsigned TPredator::m\_FailureCount `[protected]` |

Referenced by Owl::Step(), Weasel::Step(), and TPredator().

|  |
| --- |
| bool TPredator::m\_HaveTerritory `[protected]` |

Referenced by st\_Dispersal(), Owl::Step(), Weasel::Step(), and TPredator().

|  |
| --- |
| unsigned TPredator::m\_HomeRange `[protected]` |

Referenced by OverlapMyTerritory(), Owl::Owl(), st\_Movement(), TPredator(), and Weasel::Weasel().

|  |
| --- |
| int TPredator::m\_KillEfficiency `[protected]` |

Referenced by Owl::Owl(), st\_Hunting(), TPredator(), and Weasel::Weasel().

|  |
| --- |
| int TPredator::m\_kills\_this\_season `[protected]` |

Referenced by Owl::BeginStep(), Weasel::BeginStep(), st\_Hunting(), and TPredator().

|  |
| --- |
| unsigned TPredator::m\_NoFailuresBeforeDispersal `[protected]` |

Referenced by Owl::Owl(), Owl::Step(), Weasel::Step(), TPredator(), and Weasel::Weasel().

|  |
| --- |
| TPredator\_Population\_Manager\* TPredator::m\_OurPopulationManager `[protected]` |

Referenced by Owl::BeginStep(), Weasel::BeginStep(), st\_Dispersal(), and TPredator().

|  |
| --- |
| Vole\_Population\_Manager\* TPredator::m\_Prey `[protected]` |

Referenced by st\_Hunting(), and TPredator().

|  |
| --- |
| int TPredator::m\_Search\_x `[protected]` |

Referenced by st\_Hunting(), st\_Movement(), and TPredator().

|  |
| --- |
| int TPredator::m\_Search\_y `[protected]` |

Referenced by st\_Hunting(), st\_Movement(), and TPredator().

|  |
| --- |
| unsigned TPredator::m\_SearchArea `[protected]` |

Referenced by Owl::Owl(), st\_Hunting(), st\_Movement(), TPredator(), and Weasel::Weasel().

|  |
| --- |
| int TPredator::PreyResponse1 `[protected]` |

Referenced by Owl::Owl(), Owl::Step(), Weasel::Step(), TPredator(), and Weasel::Weasel().

|  |
| --- |
| int TPredator::PreyResponse2 `[protected]` |

Referenced by Owl::Owl(), Owl::Step(), Weasel::Step(), TPredator(), and Weasel::Weasel().

|  |
| --- |
| int TPredator::SimH `[protected]` |

Referenced by OverlapMyTerritory(), st\_Dispersal(), st\_Movement(), and TPredator().

|  |
| --- |
| int TPredator::SimW `[protected]` |

Referenced by OverlapMyTerritory(), st\_Dispersal(), st\_Movement(), and TPredator().

|  |
| --- |
| unsigned TPredator::SpeciesID `[protected]` |

Referenced by Owl::Owl(), st\_Dispersal(), and Weasel::Weasel().

---

The documentation for this class was generated from the following files:

- Predators.H- Predators.cpp

---

Generated on Thu Jan 22 14:13:47 2009 for ALMaSS ODDox by 
 1.5.6 
