# Supplementary material for: Predation and fragmentation portrayed in the statistical structure of prey time series
Source: BMC Ecol. 2009 May 6;9:10. doi: 10.1186/1472-6785-9-10 (PMC2689204; doi:10.1186/1472-6785-9-10)
Supplement: Additional file 2 — Voles and related classes ODDox Documentation. ODDox documentation of the agent-based model (ALMaSS) applied by Hendrichsen et al. The documentation is started by activating main.html. [file 1472-6785-9-10-S2.zip › Vole_ODDox/class_t_predator___population___manager.html]

ALMaSS ODDox: TPredator\_Population\_Manager Class Reference

- Main Page
- Related Pages
- Classes
- Files

- Alphabetical List
- Class List
- Class Hierarchy
- Class Members

# TPredator\_Population\_Manager Class Reference

`#include <Predators.H>`

Inheritance diagram for TPredator\_Population\_Manager:

List of all members.

---

## Detailed Description

The class to handle all predator population related matters.

|  |
| --- |
|  |
| Public Member Functions | |
| void | CreateObjects (int ob\_type, TAnimal \*pvo, struct\_Predator \*data, int number) |
| void | dec\_inds (unsigned list) |
| void | inc\_inds (unsigned list) |
| bool | InOtherTerritory (unsigned sp, int p\_x, int p\_y, TPredator \*p\_Pred) |
| virtual void | Run (int) |
| unsigned | supply\_no\_inds (unsigned list) |
|  | TPredator\_Population\_Manager (Landscape \*L, Vole\_Population\_Manager \*VPM) |
| virtual | ~TPredator\_Population\_Manager (void) |
| Protected Member Functions | |
| void | CloseTheReallyBigOutputProbe () |
| virtual void | CloseTheRipleysOutputProbe () |
| virtual void | DoAfter () |
| virtual void | DoBefore () |
| virtual void | DoFirst () |
| virtual void | DoLast () |
| virtual bool | StepFinished () |
| Protected Attributes | |
| unsigned | m\_no\_individuals [2] |
| Vole\_Population\_Manager \* | m\_Prey |
| unsigned | NoPredatorTypes |

---

## Constructor & Destructor Documentation

|  |  |  |  |
| --- | --- | --- | --- |
| TPredator\_Population\_Manager::TPredator\_Population\_Manager | ( | Landscape \* | *L*, |
|  |  | Vole\_Population\_Manager \* | *VPM* |  |
|  | ) |  |  |  |

References cfg\_owl\_breed\_day(), cfg\_owl\_breed\_threshold(), cfg\_owl\_death\_threshold(), cfg\_owl\_StartingNo(), cfg\_weasel\_breed\_day(), cfg\_weasel\_breed\_threshold(), cfg\_weasel\_death\_threshold(), cfg\_weasel\_StartingNo(), CreateObjects(), struct\_Predator::L, m\_no\_individuals, m\_Prey, Population\_Manager::m\_TheLandscape, OWL, owl\_breed\_day, owl\_breed\_threshold, owl\_death\_threshold, owl\_StartingNo, struct\_Predator::PM, Population\_Manager::ReallyBigOutputPrb, Population\_Manager::RipleysOutputPrb, Population\_Manager::SimH, Population\_Manager::SimW, Population\_Manager::TheArray, WEASEL, weasel\_breed\_day, weasel\_breed\_threshold, weasel\_death\_threshold, weasel\_StartingNo, struct\_Predator::x, and struct\_Predator::y.

```
00056  : Population_Manager(L)
00057 {
00058   // Constants for the predator species
00059   weasel_breed_threshold=cfg_weasel_breed_threshold.value();
00060   owl_breed_threshold=cfg_owl_breed_threshold.value();
00061   weasel_death_threshold=cfg_weasel_death_threshold.value();
00062   owl_death_threshold=cfg_owl_death_threshold.value();
00063   weasel_breed_day=cfg_weasel_breed_day.value();
00064   owl_breed_day=cfg_owl_breed_day.value();
00065   weasel_StartingNo=cfg_weasel_StartingNo.value();
00066   owl_StartingNo=cfg_owl_StartingNo.value();
00067   // Must now create as many array elements as we have predator types
00068   // two lists are needed so need to remove 8 of the ten default arrays
00069   // Weasels and Owls
00070   for (int i=0; i<8; i++)
00071   {
00072     TheArray.pop_back();
00073   }
00074   // Remember the prey
00075   m_Prey=VPM;
00076   m_no_individuals[WEASEL]=0;
00077   m_no_individuals[OWL]=0;
00078   // Create some weasels and owls
00079   struct_Predator* sp;
00080   sp = new struct_Predator;
00081   sp->PM = this;
00082   sp->L = m_TheLandscape;
00083   for (int i=0; i<weasel_StartingNo; i++)
00084   {
00085     sp->x = random(SimW);
00086     sp->y = random(SimH);
00087     CreateObjects(0,NULL,sp,1); // 0 = weasel
00088   }
00089   for (int i=0; i<owl_StartingNo; i++)
00090   {
00091     sp->x = random(SimW);
00092     sp->y = random(SimH);
00093     CreateObjects(1,NULL,sp,1); // 1 = Owl
00094   }
00095   delete sp;
00096   ReallyBigOutputPrb=0;
00097   RipleysOutputPrb=0;
00098 }
```

|  |  |  |  |  |  |
| --- | --- | --- | --- | --- | --- |
| TPredator\_Population\_Manager::~TPredator\_Population\_Manager | ( | void |  | ) | `[virtual]` |

```
00049 {
00050    // Should all be done by the Population_Manager destructor
00051 }
```

---

## Member Function Documentation

|  |  |  |  |  |
| --- | --- | --- | --- | --- |
| void TPredator\_Population\_Manager::CloseTheReallyBigOutputProbe | ( |  | ) | `[inline, protected]` |

close the probe

Reimplemented from Population\_Manager.

```
00152 {}; // This will always be done by the main population manager
```

|  |  |  |  |  |
| --- | --- | --- | --- | --- |
| virtual void TPredator\_Population\_Manager::CloseTheRipleysOutputProbe | ( |  | ) | `[inline, protected, virtual]` |

close the probe

Reimplemented from Population\_Manager.

```
00153 {}; // This will always be done by the main population manager
```

|  |  |  |  |
| --- | --- | --- | --- |
| void TPredator\_Population\_Manager::CreateObjects | ( | int | *ob\_type*, |
|  |  | TAnimal \* | *pvo*, |
|  |  | struct\_Predator \* | *data*, |
|  |  | int | *number* |  |
|  | ) |  |  |  |

References inc\_inds(), struct\_Predator::L, m\_Prey, OWL, struct\_Predator::PM, Population\_Manager::TheArray, WEASEL, struct\_Predator::x, and struct\_Predator::y.

Referenced by Owl::BeginStep(), Weasel::BeginStep(), and TPredator\_Population\_Manager().

```
00103 {
00104    Weasel*  new_Weasel;
00105    Owl*  new_Owl;
00106    for (int i=0; i<number; i++)
00107    {
00108     if (ob_type == WEASEL)   // Weasel
00109     {
00110        new_Weasel = new Weasel(m_Prey,data->x, data->y, data->L, data->PM);
00111        TheArray[ob_type].push_back(new_Weasel);
00112        inc_inds(WEASEL);
00113     }
00114     if (ob_type == OWL)  // Owl
00115     {
00116        new_Owl = new Owl(m_Prey,data->x, data->y, data->L, data->PM);
00117        TheArray[ob_type].push_back(new_Owl);
00118        inc_inds(OWL);
00119     }
00120    }
00121 }
```

|  |  |  |  |  |  |
| --- | --- | --- | --- | --- | --- |
| void TPredator\_Population\_Manager::dec\_inds | ( | unsigned | *list* | ) | `[inline]` |

References m\_no\_individuals.

Referenced by Owl::BeginStep(), and Weasel::BeginStep().

```
00138 {m_no_individuals[list]--;}
```

|  |  |  |  |  |
| --- | --- | --- | --- | --- |
| virtual void TPredator\_Population\_Manager::DoAfter | ( |  | ) | `[inline, protected, virtual]` |

Can be used in descendent classes

Reimplemented from Population\_Manager.

Referenced by Run().

```
00150 {}
```

|  |  |  |  |  |
| --- | --- | --- | --- | --- |
| virtual void TPredator\_Population\_Manager::DoBefore | ( |  | ) | `[inline, protected, virtual]` |

Can be used in descendent classes

Reimplemented from Population\_Manager.

Referenced by Run().

```
00149 {}
```

|  |  |  |  |  |
| --- | --- | --- | --- | --- |
| virtual void TPredator\_Population\_Manager::DoFirst | ( |  | ) | `[inline, protected, virtual]` |

Can be used in descendent classes

Reimplemented from Population\_Manager.

Referenced by Run().

```
00148 {}
```

|  |  |  |  |  |
| --- | --- | --- | --- | --- |
| virtual void TPredator\_Population\_Manager::DoLast | ( |  | ) | `[inline, protected, virtual]` |

Collects some data to describe the number of animals in each state at the end of the day

Reimplemented from Population\_Manager.

Referenced by Run().

```
00151 {}
```

|  |  |  |  |  |  |
| --- | --- | --- | --- | --- | --- |
| void TPredator\_Population\_Manager::inc\_inds | ( | unsigned | *list* | ) | `[inline]` |

References m\_no\_individuals.

Referenced by CreateObjects().

```
00137 {m_no_individuals[list]++;}
```

|  |  |  |  |
| --- | --- | --- | --- |
| bool TPredator\_Population\_Manager::InOtherTerritory | ( | unsigned | *sp*, |
|  |  | int | *p\_x*, |
|  |  | int | *p\_y*, |
|  |  | TPredator \* | *p\_Pred* |  |
|  | ) |  |  |  |

References TPredator::OverlapMyTerritory(), and Population\_Manager::TheArray.

Referenced by TPredator::st\_Dispersal().

```
00204 {
00205   // Go through sp species and see if a territory at x,y will overlap with
00206   // theirs
00207   for (unsigned i=0; i<TheArray[sp].size(); i++)
00208   {
00209      TPredator* APredator=(TPredator *)TheArray[sp][i];
00210      if (APredator->OverlapMyTerritory(x,y))
00211      {
00212         if (APredator!=p_Pred) return true;
00213      }
00214   }
00215   return false;
00216 }
```

|  |  |  |  |  |  |
| --- | --- | --- | --- | --- | --- |
| void TPredator\_Population\_Manager::Run | ( | int | *NoTSteps* | ) | `[virtual]` |

This is the main scheduling method for the population manager.   
Note the structure of Shuffle\_or\_Sort(), DoFirst(), BeginStep, DoBefore(), Step looping until all are finished, DoAfter(), DoAlmostLast(), EndStep, DoLast().   

Can do multiple time-steps here inside one landscape time-step (a day). This is used in the roe deer model to provide 10 minute behavioural time-steps.

Reimplemented from Population\_Manager.

References DoAfter(), DoBefore(), DoFirst(), DoLast(), StepFinished(), and Population\_Manager::TheArray.

```
00126 {
00127   DoFirst();
00128  // begin step actions ...
00129  // set all stepdone to false.... is this really necessary??
00130  for (unsigned listindex=0; listindex<TheArray.size(); listindex++)
00131  {
00132    for (unsigned j=0; j<TheArray[listindex].size(); j++)
00133    {
00134        TheArray[listindex][j]->StepDone=false;
00135    }
00136  }
00137  // call the begin-step-method of all objects
00138  for (unsigned listindex=0; listindex<TheArray.size();listindex++)
00139  {
00140    for (unsigned j=0; j<TheArray[listindex].size(); j++)
00141     TheArray[listindex][j]->BeginStep();
00142  }
00143   DoBefore();
00144  // call the step-method of all objects
00145  do
00146  {
00147    for (unsigned listindex=0; listindex<TheArray.size();listindex++)
00148    {
00149      for (unsigned j=0; j<TheArray[listindex].size(); j++)
00150      {
00151        TheArray[listindex][j]->Step();
00152      }
00153    } // for listindex
00154  } while (!StepFinished());
00155  DoAfter();
00156  // call the end-step-method of all objects
00157  for (unsigned listindex=0; listindex<TheArray.size();listindex++)
00158  {
00159    for (unsigned j=0; j<TheArray[listindex].size(); j++)
00160    {
00161      TheArray[listindex][j]->EndStep();
00162    }
00163  }
00164  // ----------------
00165  // end of this step actions
00166 
00167  // For each animal list
00168   for (unsigned listindex=0; listindex<TheArray.size();listindex++)
00169  {
00170    // Must check each object in the list for CurrentStateNo==-1
00171    int TAend=(int)TheArray[listindex].size()-1;
00172    for (int j=TAend; j>=0; j--)  // Search backwards is more efficicent
00173    {
00174      if (TheArray[listindex][j]->CurrentStateNo==-1) // code for kill it
00175      {
00176        delete TheArray[listindex][j];
00177        TheArray[listindex].erase(TheArray[listindex].begin()+j);
00178      }
00179    }
00180  }
00181  DoLast();
00182 }
```

|  |  |  |  |  |  |
| --- | --- | --- | --- | --- | --- |
| bool TPredator\_Population\_Manager::StepFinished | ( | void |  | ) | `[protected, virtual]` |

Returns true if and only if all objects have finished the current step

Reimplemented from Population\_Manager.

References Population\_Manager::TheArray.

Referenced by Run().

```
00187 {
00188    for (unsigned listindex=0; listindex<TheArray.size();listindex++)
00189    {
00190      for (unsigned j=0; j<TheArray[listindex].size(); j++)
00191      {
00192        if (TheArray[listindex][j]->StepDone==false)
00193        {
00194          return false;
00195        }
00196      }
00197    }
00198  return true;
00199 }
```

|  |  |  |  |  |  |
| --- | --- | --- | --- | --- | --- |
| unsigned TPredator\_Population\_Manager::supply\_no\_inds | ( | unsigned | *list* | ) | `[inline]` |

References m\_no\_individuals.

Referenced by Owl::BeginStep(), and Weasel::BeginStep().

```
00139 {return m_no_individuals[list];}
```

---

## Member Data Documentation

|  |
| --- |
| unsigned TPredator\_Population\_Manager::m\_no\_individuals[2] `[protected]` |

Referenced by dec\_inds(), inc\_inds(), supply\_no\_inds(), and TPredator\_Population\_Manager().

|  |
| --- |
| Vole\_Population\_Manager\* TPredator\_Population\_Manager::m\_Prey `[protected]` |

Referenced by CreateObjects(), and TPredator\_Population\_Manager().

|  |
| --- |
| unsigned TPredator\_Population\_Manager::NoPredatorTypes `[protected]` |

---

The documentation for this class was generated from the following files:

- Predators.H- Predators.cpp

---

Generated on Thu Jan 22 14:13:47 2009 for ALMaSS ODDox by 
 1.5.6 
