# Supplementary material for: Predation and fragmentation portrayed in the statistical structure of prey time series
Source: BMC Ecol. 2009 May 6;9:10. doi: 10.1186/1472-6785-9-10 (PMC2689204; doi:10.1186/1472-6785-9-10)
Supplement: Additional file 2 — Voles and related classes ODDox Documentation. ODDox documentation of the agent-based model (ALMaSS) applied by Hendrichsen et al. The documentation is started by activating main.html. [file 1472-6785-9-10-S2.zip › Vole_ODDox/class_user_defined_farm1.html]

ALMaSS ODDox: UserDefinedFarm1 Class Reference

- Main Page
- Related Pages
- Classes
- Files

- Alphabetical List
- Class List
- Class Hierarchy
- Class Members

# UserDefinedFarm1 Class Reference

`#include <farm.h>`

Inheritance diagram for UserDefinedFarm1:

List of all members.

---

## Detailed Description

A farm that can have its rotation defined by the user at runtime.

The rotation is controlled by a file with the format: N  
Crop  
Crop  
Crop...  
..........where N is the number of entries and "Crop" is replaced by the crop name for each entry in the rotation. There are 16 Userdefined farm types available numbered 1 to 16, but 2->16 have been excluded from the documentation because they are identical to this class in all but name.  

|  |
| --- |
|  |
| Public Member Functions | |
|  | UserDefinedFarm1 (void) |

---

## Constructor & Destructor Documentation

|  |  |  |  |  |  |
| --- | --- | --- | --- | --- | --- |
| UserDefinedFarm1::UserDefinedFarm1 | ( | void |  | ) |  |

References Farm::m\_farmtype, Farm::m\_rotation, Farm::m\_stockfarmer, tof\_UserDefinedFarm1, and Farm::TranslateCropCodes().

```
01312                                          : Farm() // 15
01313 {
01314   m_farmtype = tof_UserDefinedFarm1;
01315   m_stockfarmer = true;
01316   // This farm type reads its rotation from a special file UserDefinedFarm1.rot
01317   FILE * inpfile = fopen("UserDefinedFarm1.rot", "r" );
01318   if (!inpfile) {
01319     g_msg->Warn( WARN_FILE, "UserDefinedFarm1::UserDefinedFarm1():"" Unable to open file ", "UserDefinedFarm1.rot" );
01320     exit( 1 );
01321   }
01322   int nocrops;
01323   fscanf( inpfile, "%d\n", & nocrops );
01324   m_rotation.resize( nocrops );
01325   char cropref[ 255 ];
01326   for ( int i = 0; i < nocrops; i++ ) {
01327     fscanf( inpfile, "%s\n", & cropref);
01328     TTypesOfVegetation tov = TranslateCropCodes( cropref );
01329     m_rotation[ i ] = tov;
01330   }
01331   fclose( inpfile );
01332 }
```

---

The documentation for this class was generated from the following files:

- farm.h- farm.cpp

---

Generated on Thu Jan 22 14:13:47 2009 for ALMaSS ODDox by 
 1.5.6 
