# Supplementary material for: Predation and fragmentation portrayed in the statistical structure of prey time series
Source: BMC Ecol. 2009 May 6;9:10. doi: 10.1186/1472-6785-9-10 (PMC2689204; doi:10.1186/1472-6785-9-10)
Supplement: Additional file 2 — Voles and related classes ODDox Documentation. ODDox documentation of the agent-based model (ALMaSS) applied by Hendrichsen et al. The documentation is started by activating main.html. [file 1472-6785-9-10-S2.zip › Vole_ODDox/class_user_defined_farm6.html]

ALMaSS ODDox: UserDefinedFarm6 Class Reference

- Main Page
- Related Pages
- Classes
- Files

- Alphabetical List
- Class List
- Class Hierarchy
- Class Members

# UserDefinedFarm6 Class Reference

`#include <farm.h>`

Inheritance diagram for UserDefinedFarm6:

List of all members.

---

## Detailed Description

A farm that can have its rotation defined by the user at runtime.

|  |
| --- |
|  |
| Public Member Functions | |
|  | UserDefinedFarm6 (void) |

---

## Constructor & Destructor Documentation

|  |  |  |  |  |  |
| --- | --- | --- | --- | --- | --- |
| UserDefinedFarm6::UserDefinedFarm6 | ( | void |  | ) |  |

References Farm::m\_farmtype, Farm::m\_rotation, Farm::m\_stockfarmer, tof\_UserDefinedFarm6, and Farm::TranslateCropCodes().

```
01422                                          : Farm() // 20
01423 {
01424   m_farmtype = tof_UserDefinedFarm6;
01425   m_stockfarmer = true;
01426   // This farm type reads its rotation from a special file PesticideTrialTreatment.rot
01427   FILE * inpfile = fopen("UserDefinedFarm6.rot", "r" );
01428   if (!inpfile) {
01429     g_msg->Warn( WARN_FILE, "UserDefinedFarm6::UserDefinedFarm6():"" Unable to open file ", "UserDefinedFarm6.rot" );
01430     exit( 1 );
01431   }
01432   int nocrops;
01433   fscanf( inpfile, "%d\n", & nocrops );
01434   m_rotation.resize( nocrops );
01435   char cropref[ 255 ];
01436   for ( int i = 0; i < nocrops; i++ ) {
01437     fscanf( inpfile, "%s\n", & cropref );
01438     TTypesOfVegetation tov = TranslateCropCodes( cropref );
01439     m_rotation[ i ] = tov;
01440   }
01441   fclose( inpfile );
01442 }
```

---

The documentation for this class was generated from the following files:

- farm.h- farm.cpp

---

Generated on Thu Jan 22 14:13:47 2009 for ALMaSS ODDox by 
 1.5.6 
