# Supplementary material for: Predation and fragmentation portrayed in the statistical structure of prey time series
Source: BMC Ecol. 2009 May 6;9:10. doi: 10.1186/1472-6785-9-10 (PMC2689204; doi:10.1186/1472-6785-9-10)
Supplement: Additional file 2 — Voles and related classes ODDox Documentation. ODDox documentation of the agent-based model (ALMaSS) applied by Hendrichsen et al. The documentation is started by activating main.html. [file 1472-6785-9-10-S2.zip › Vole_ODDox/class_vole___base.html]

ALMaSS ODDox: Vole\_Base Class Reference

- Main Page
- Related Pages
- Classes
- Files

- Alphabetical List
- Class List
- Class Hierarchy
- Class Members

# Vole\_Base Class Reference

`#include <vole_all.h>`

Inheritance diagram for Vole\_Base:

List of all members.

---

## Detailed Description

Base class for voles - all vole objects are descended from this class.

|  |
| --- |
|  |
| Public Member Functions | |
| virtual void | BeginStep () |
| void | CopyMyself (int a\_vtype) |
|  | Duplicates a vole. |
| virtual void | EndStep () |
|  | All voles age at the end of the day. |
| int | GetDirectFlag () |
| int | GetGeneticFlag () |
| bool | MortalityTest () |
|  | Do a mortality test. |
| virtual void | OnKilled () |
| void | Set\_Age (int Age) |
| void | SetDirectFlag () |
| void | SetGeneticFlag () |
| void | Setm\_Mature () |
| void | SetWeight (double W) |
| void | st\_Dying () |
|  | All voles end here on death. |
| virtual void | Step () |
| unsigned | SupplyAge () |
| int | SupplyAllele (int locus, int allele) |
| GeneticMaterial | SupplyGenes () |
| int | SupplyHeteroZyg () |
| int | SupplyHomoZyg () |
| bool | SupplySex () |
| bool | SupplyTerritorial () |
| unsigned | SupplyX () |
| unsigned | SupplyY () |
| void | UnsetDirectFlag () |
| void | UnsetGeneticFlag () |
|  | Vole\_Base (int x, int y, Landscape \*L, GeneticMaterial DNA, Vole\_Population\_Manager \*VPM) |
|  | Constructor for Vole\_Base. |
| virtual int | WhatState () |
|  | ~Vole\_Base () |
| Public Attributes | |
| TTypeOfVoleState | CurrentVState |
| Vole\_Population\_Manager \* | m\_OurPopulation |
| bool | m\_pesticideInfluenced2 |
| Protected Member Functions | |
| double | AssessHabitat (int polyref) |
|  | Assess the quality of habitat at p\_Polyref. |
| double | CalculateCarryingCapacity (int x, int y, int &p\_stand\_x, int &p\_stand\_y) |
| double | CalculateCarryingCapacity (int x, int y) |
| void | DoWalking (int p\_Distance, int &p\_Vector, int &vx, int &vy) |
|  | Walking. |
| void | DoWalkingCorrect (int p\_Distance, int &p\_Vector, int &vx, int &vy) |
|  | Walking where there is a danger of stepping off the world. |
| void | Escape (int p\_Vector, int p\_Distance) |
|  | Dispersal - directed movement. |
| virtual void | FreeLocation () |
| virtual bool | GetLocation (int, int) |
| int | MoveQuality (int p\_x, int p\_y) |
|  | Test a location for quality while moving. |
| void | MoveTo (int p\_Vector, int p\_Distance, int iterations) |
|  | Movement. |
| virtual void | SetLocation () |
| Protected Attributes | |
| unsigned | IDNo |
| int | m\_Age |
| int | m\_DispVector |
| bool | m\_Have\_Territory |
| int | m\_LifeSpan |
| bool | m\_Mature |
| bool | m\_pesticideInfluenced |
| int | m\_Reserves |
| bool | m\_Sex |
| int | m\_StarvationDays |
| int | m\_TerrRange |
| double | m\_Weight |
| GeneticMaterial | MyGenes |
| int | SimH |
| int | SimW |

---

## Constructor & Destructor Documentation

|  |  |  |  |
| --- | --- | --- | --- |
| Vole\_Base::Vole\_Base | ( | int | *x*, |
|  |  | int | *y*, |
|  |  | Landscape \* | *L*, |
|  |  | GeneticMaterial | *DNA*, |
|  |  | Vole\_Population\_Manager \* | *VPM* |  |
|  | ) |  |  |  |

Constructor for Vole\_Base.

References CurrentVState, g\_rand\_uni, IDNo, Vole\_Population\_Manager::IDNumber, m\_Age, m\_DispVector, m\_Have\_Territory, m\_LifeSpan, m\_Mature, m\_OurPopulation, m\_pesticideInfluenced, m\_pesticideInfluenced2, m\_Reserves, m\_StarvationDays, m\_TerrRange, MyGenes, Vole\_Population\_Manager::ReproTable, GeneticMaterial::ScoreReproduction(), Population\_Manager::SimH, SimH, Population\_Manager::SimW, SimW, and tovs\_InitialState.

```
00196                                                      : TAnimal(p_x,p_y,p_L)
00197 {
00198     m_OurPopulation=p_VPM;
00199     m_Mature=false;
00200     m_Age=0;
00201     m_StarvationDays=0;
00202     m_DispVector=-1;
00203     m_Have_Territory=false;
00204     m_TerrRange=0;  // The size of the territory
00205     m_Reserves=3;
00206     m_LifeSpan=(14*30)+ int(g_rand_uni()*(30*6)); // 14-20 months
00207 //    m_LifeSpan=random(5*365);
00208     CurrentVState=tovs_InitialState;
00209     MyGenes=DNA;
00210     SimH=m_OurPopulation->SimH;
00211     SimW=m_OurPopulation->SimW;
00212     IDNo=m_OurPopulation->IDNumber++;
00213 #ifdef __SpecificPesticideEffectsVinclozolinLike__
00214         m_pesticideInfluenced=false;
00215         m_pesticideInfluenced2=false;
00216 #endif
00217 
00218 #ifdef __PHENOTYPIC_LINK_REPRO
00219   // Must read our genetic code and set parameters accordingly
00220   // For sex specific parameters this is done in the constructor of the sexes
00221   // 1. Score our genetic code
00222   //
00223   double MultiplicationFactor=MyGenes.ScoreReproduction();
00224   // 2. Initialise parameter values
00225   double ReproVals[2][12];
00226   for (int i=0; i<2; i++) for (int j=0; j<12; j++)
00227   {
00228     ReproVals[i][j]=m_OurPopulation->ReproTable[i][j]*MultiplicationFactor;
00229   }
00230 
00231 
00232   // 3. Alter our copy of the ReproTable
00233   for (int i=0; i<12; i++)
00234   {
00235      MyReproTable[0][i]=(int)floor(ReproVals[0][i]);
00236      MyReproTable[2][i]=(int)floor(0.5+(ReproVals[0][i]*100)-
00237                                                       (MyReproTable[0][i]*100));
00238      MyReproTable[1][i]=(int)floor(ReproVals[1][i]);
00239      MyReproTable[3][i]=(int)floor(0.5+(ReproVals[1][i]*100)-
00240                                                       (MyReproTable[1][i]*100));
00241   }
00242 
00243 #endif
00244 }
```

|  |  |  |  |  |
| --- | --- | --- | --- | --- |
| Vole\_Base::~Vole\_Base | ( |  | ) |  |

```
00248 {
00249 }
```

---

## Member Function Documentation

|  |  |  |  |  |  |
| --- | --- | --- | --- | --- | --- |
| double Vole\_Base::AssessHabitat | ( | int | *p\_Polyref* | ) | `[protected]` |

Assess the quality of habitat at p\_Polyref.

Works by classifying into Optimal, Suboptimal, Marginal, No habitat,and Non-habitat

References TAnimal::m\_OurLandscape, tole\_ActivePit, tole\_Building, tole\_Coast, tole\_ConiferousForest, tole\_DeciduousForest, tole\_Field, tole\_FieldBoundary, tole\_Foobar, tole\_Freshwater, tole\_Garden, tole\_Heath, tole\_HedgeBank, tole\_Hedges, tole\_LargeRoad, tole\_Marsh, tole\_MixedForest, tole\_NaturalGrass, tole\_Orchard, tole\_PermanentSetaside, tole\_PermPasture, tole\_PermPastureLowGrazing, tole\_PitDisused, tole\_Railway, tole\_River, tole\_RiversidePlants, tole\_RiversideTrees, tole\_RoadsideVerge, tole\_Saltwater, tole\_Scrub, tole\_SmallRoad, tole\_StoneWall, tole\_Track, tole\_UnsprayedFieldMargin, tole\_Urban, tole\_YoungForest, tov\_AgroChemIndustryCereal, tov\_Carrots, tov\_CloverGrassGrazed1, tov\_CloverGrassGrazed2, tov\_FieldPeas, tov\_FodderBeet, tov\_Maize, tov\_Oats, tov\_OBarleyPeaCloverGrass, tov\_OCloverGrassGrazed1, tov\_OCloverGrassGrazed2, tov\_OCloverGrassSilage1, tov\_OFieldPeas, tov\_OFieldPeasSilage, tov\_OGrazingPigs, tov\_OPermanentGrassGrazed, tov\_OSBarleySilage, tov\_OSetaside, tov\_OSpringBarley, tov\_OSpringBarleyPigs, tov\_OWinterBarley, tov\_OWinterRape, tov\_OWinterRye, tov\_OWinterWheatUndersown, tov\_PermanentGrassGrazed, tov\_PermanentGrassLowGrazed, tov\_PermanentSetaside, tov\_Potatoes, tov\_SeedGrass1, tov\_SeedGrass2, tov\_Setaside, tov\_SpringBarley, tov\_SpringBarleyCloverGrass, tov\_SpringBarleyPeaCloverGrassStrigling, tov\_SpringBarleySeed, tov\_SpringBarleySilage, tov\_SpringBarleyStrigling, tov\_SpringBarleyStriglingCulm, tov\_SpringBarleyStriglingSingle, tov\_Triticale, tov\_WinterBarley, tov\_WinterRape, tov\_WinterRye, tov\_WinterWheat, tov\_WinterWheatShort, tov\_WinterWheatStrigling, tov\_WinterWheatStriglingCulm, tov\_WinterWheatStriglingSingle, tov\_WWheatPControl, tov\_WWheatPToxicControl, and tov\_WWheatPTreatment.

Referenced by CalculateCarryingCapacity().

```
01086 {
01087   TTypesOfLandscapeElement ElementType;
01088   ElementType = m_OurLandscape->SupplyElementType(p_Polyref);
01089   TTypesOfVegetation VType;
01090   double Cover;
01091   int score = -9999;
01092   switch (ElementType)
01093   {
01094     case tole_Heath:
01095     case tole_Hedges: // 130 (internal ALMaSS representation for Hedges)
01096      score=3;
01097      break;
01098     case tole_Railway: // 118
01099      score=3;
01100      break;
01101     case tole_Marsh: // 95
01102      score=2;
01103      break;
01104     case tole_Scrub: // 70
01105      score=1;
01106      break;
01107     case tole_Field: // 20 & 30
01108     case tole_UnsprayedFieldMargin:
01109      VType=m_OurLandscape->SupplyVegType(p_Polyref);
01110      Cover=m_OurLandscape->SupplyVegCover(p_Polyref);
01111      if (Cover<0.20) score=0;
01112      else switch (VType)
01113      {
01114        // return 3
01115        case tov_PermanentSetaside:
01116         score=3;
01117         break;
01118        // return 2
01119        case tov_SeedGrass1:
01120        case tov_SeedGrass2:
01121        case tov_OSetaside:
01122        case tov_Setaside:
01123          score=2;
01124          break;
01125        // return 1
01126        case tov_FieldPeas:
01127        case tov_WinterBarley:
01128        case tov_OWinterBarley:
01129        case tov_WinterRye:
01130        case tov_OWinterRye:
01131        case tov_Oats:
01132        case tov_AgroChemIndustryCereal:
01133        case tov_WinterWheat:
01134        case tov_WinterWheatStrigling:
01135        case tov_WinterWheatStriglingSingle:
01136        case tov_WinterWheatStriglingCulm:
01137        case tov_Triticale:
01138        case tov_WinterWheatShort:
01139        case tov_WWheatPControl:
01140        case tov_WWheatPToxicControl:
01141        case tov_WWheatPTreatment:
01142        case tov_WinterRape:
01143        case tov_OSpringBarley:
01144        case tov_SpringBarley:
01145        case tov_SpringBarleySeed:
01146        case tov_SpringBarleyStrigling:
01147        case tov_SpringBarleyStriglingSingle:
01148        case tov_SpringBarleyStriglingCulm:
01149        case tov_SpringBarleyCloverGrass:
01150        case tov_SpringBarleySilage:
01151        case tov_OBarleyPeaCloverGrass:
01152        case tov_SpringBarleyPeaCloverGrassStrigling:
01153        case tov_OSBarleySilage:
01154        case tov_OWinterWheatUndersown:
01155        case tov_OFieldPeas:
01156        case tov_OFieldPeasSilage:
01157        case tov_OSpringBarleyPigs:
01158        case tov_OWinterRape:
01159        case tov_Maize:
01160          score=1;
01161          break;
01162        //  return 0
01163        case tov_OGrazingPigs:
01164        case tov_FodderBeet:
01165        case tov_Carrots:
01166            case tov_Potatoes:
01167          score=0;
01168          break;
01169        //  Needs some more thought
01170        case tov_OCloverGrassSilage1:
01171        case tov_CloverGrassGrazed1:
01172        case tov_CloverGrassGrazed2:
01173        case tov_OCloverGrassGrazed1:
01174        case tov_OCloverGrassGrazed2:
01175        case tov_OPermanentGrassGrazed:
01176        case tov_PermanentGrassGrazed:
01177          if (m_OurLandscape->SupplyGrazingPressure(p_Polyref)) score=1;
01178          else score=3;
01179          break;
01180            case tov_PermanentGrassLowGrazed: 
01181          if (m_OurLandscape->SupplyGrazingPressure(p_Polyref)) score=2;
01182          else score=3;
01183          break;
01184        default:
01185        //   Unknown vegetation type
01186        m_OurLandscape->Warn("Vole_Base::AssessHabitat - Unknown vegt  type",NULL);
01187        exit(1);
01188        break;
01189      }
01190      break;
01191     case tole_PermPasture: // 35
01192      if (m_OurLandscape->SupplyGrazingPressure(p_Polyref))score=1; else score=3;
01193      break;
01194     case tole_PermPastureLowGrazing:
01195      if (m_OurLandscape->SupplyGrazingPressure(p_Polyref))score=3; else score=4;
01196      break;
01197     case tole_Orchard: // 32
01198      // Quality depends on when it was mown
01199      if (m_OurLandscape->SupplyJustMown(p_Polyref)) score=0; else score=99;
01200      break;
01201     case tole_FieldBoundary: // 160
01202     case tole_PermanentSetaside:
01203     case tole_RoadsideVerge: // 13
01204     case tole_NaturalGrass: // 110
01205     case tole_HedgeBank:
01206      score=4;
01207      break;
01208     case tole_RiversidePlants: // 98
01209      score=2;
01210      break;
01211     case tole_PitDisused: // 75
01212      score=2;
01213      break;
01214     case tole_RiversideTrees: // 97
01215      score=1;
01216      break;
01217     case tole_DeciduousForest: // 40
01218      score=1;
01219      break;
01220     case tole_YoungForest: // 60
01221      score=3;
01222      break;
01223     case tole_MixedForest: // 60
01224      score=1;
01225      break;
01226     case tole_ConiferousForest: // 50
01227      score=1;
01228      break;
01229     case tole_StoneWall: // 15
01230      score=-1;
01231      break;
01232     case tole_Garden: //11
01233      score=0;
01234      break;
01235     case tole_Track: // 123
01236      score=0;
01237      break;
01238     case tole_SmallRoad: // 122
01239      score=0;
01240      break;
01241     case tole_LargeRoad: // 121
01242      score=0;
01243      break;
01244     case tole_Building: // 5
01245      score=-1;
01246      break;
01247     case tole_Urban: // 10
01248      score=-1;
01249      break;
01250     case tole_ActivePit: // 115
01251      score=-1;
01252      break;
01253     case tole_Freshwater: // 90
01254      score=-1;
01255      break;
01256     case tole_River: // 96
01257      score=-1;
01258      break;
01259     case tole_Saltwater: // 80
01260      score=-1;
01261      break;
01262     case tole_Coast: // 100
01263      score=-1;
01264      break;
01265     case tole_Foobar: // 999 !! type unknown - should not happen
01266     default:
01267      static char errornum[20];
01268      sprintf(errornum, "%d", ElementType);
01269      m_OurLandscape->Warn("Vole_Base:AssessHabitat: Unknown tole_type",
01270      errornum);
01271      exit(1);
01272   }
01273   if (score!=99) return score;
01274   else
01275   {
01276    if (m_OurLandscape->SupplyVegCover(p_Polyref)<0.8) return 3;
01277    if (m_OurLandscape->SupplyVegHeight(p_Polyref)<=40) return 3;
01278    return 4;
01279 
01280   }
01281 }
```

|  |  |  |  |  |  |
| --- | --- | --- | --- | --- | --- |
| virtual void Vole\_Base::BeginStep | ( | void |  | ) | `[inline, virtual]` |

Reimplemented from TAnimal.

Reimplemented in Vole\_Male, and Vole\_Female.

```
00125 {};
```

|  |  |  |  |
| --- | --- | --- | --- |
| double Vole\_Base::CalculateCarryingCapacity | ( | int | *p\_x*, |
|  |  | int | *p\_y*, |
|  |  | int & | *p\_stand\_x*, |
|  |  | int & | *p\_stand\_y* |  |
|  | ) |  |  | `[protected]` |

Do an assessment of the quality of the voles territory, and move. This function does the same as the other CalcuateCarryingCapacity except that it alters the two values stand\_x, stand\_y to set them to be in the best polygon in the territory   
  
Returns the mean of the qualities in the area covered by the territory (even if the vole does not have one)   
  
Uses the algorithm for fast searching of square arrays with wrap around co-ordinates.   
parameters = x,y starting coordinates top left   
range = extent of the space to search   
bottom left coordinate is therefore x+range, y+range   
22/09/2000   
  
For each polygon it gets the quality and multiplies by area of that polygon in the territory. This is summed for all polygons in the territory to get overall quality.

References AssessHabitat(), TAnimal::m\_OurLandscape, m\_OurPopulation, m\_TerrRange, Population\_Manager::SimH, SimH, Population\_Manager::SimW, and SimW.

```
00545 {
00546   int NoPolygons = 0;
00547   int PolyRefData[200][2];
00548   // First convert centre co-rdinates to square co-ordinates
00549   double quality = 0;
00550   int x=p_x - m_TerrRange;
00551   if (x<0) x+=SimW;
00552   int y=p_y - m_TerrRange;
00553   if (y<0) y+=SimH;
00554   int range_x=m_TerrRange+m_TerrRange;
00555   int range_y=m_TerrRange+m_TerrRange;
00556   // Stage 1 make a list of polygons
00557   // create the extent variables
00558   int xextent0 = x+range_x;
00559   int yextent0 = y+range_y;
00560   int xextent1 = (x+range_x)-SimW;
00561   int yextent1 = (y+range_y)-SimH;
00562   // Create the looping variables needed
00563   int Dfinx=xextent0;
00564   int Dfiny=yextent0;
00565   int Afinx=0;  // unless the finx values for A-C are changed
00566   int Bfinx=0;  // the value of zero will stop the A-C loops from executing
00567   int Cfinx=0;
00568   int Afiny=0; // this one is only assigned as 0 to stop compiler complaints
00569   // Now create the loop values;
00570   if (x+range_x<=SimW)
00571   {
00572     // Type B & D (overlap bottom, no overlap)
00573     if (yextent0>SimH)
00574     {
00575       // Type B (overlap bottom only)
00576       Dfiny=SimH;
00577       Bfinx=x+range_x;
00578     }
00579   }
00580   else
00581   {
00582     // Type A & C overlap left edge or bottom & left
00583     if (yextent0>SimH)
00584     {
00585       // Type C overlap bottom and left
00586       Afinx=xextent1;
00587       Afiny=m_OurPopulation->SimH;
00588       Bfinx=m_OurPopulation->SimW;
00589       Cfinx=xextent1;
00590       Dfinx=m_OurPopulation->SimW;
00591       Dfiny=m_OurPopulation->SimH;
00592     }
00593     else
00594     {
00595       // Type A overlap left edge
00596       Afinx=xextent1;
00597       Afiny=yextent0;
00598       Dfinx=SimW;
00599     }
00600   }
00601   // the default is:
00602   // Type D no overlap
00603 
00604 
00605   // A Loop
00606   for (int i=0; i<Afinx; i++)
00607   {
00608     for (int j=y; j<Afiny; j++)
00609     {
00610       // Get the polyref
00611       int PRef = m_OurLandscape->SupplyPolyRef(i,j);
00612       // check if we have had this one already
00613       int index=-1;
00614       for (int k=0; k<NoPolygons; k++)
00615       {
00616         if (PolyRefData[k][0]==PRef)
00617         {
00618           index=k;
00619           break;
00620         }
00621       }
00622       if (index!=-1)
00623       {
00624         PolyRefData[index][1]++;
00625       }
00626       else
00627       {
00628         // don't have this one so get the height & type to the PolyDatas arrays
00629         PolyRefData[NoPolygons][0]=PRef;
00630         PolyRefData[NoPolygons][1]=1;
00631         NoPolygons++;
00632       }
00633     }
00634   }
00635   // B Loop
00636   for (int i=x; i<Bfinx; i++)
00637   {
00638     for (int j=0; j<yextent1; j++)
00639     {
00640       // Get the polyref
00641       int PRef = m_OurLandscape->SupplyPolyRef(i,j);
00642       // check if we have had this one already
00643       int index=-1;
00644       for (int k=0; k<NoPolygons; k++)
00645       {
00646         if (PolyRefData[k][0]==PRef)
00647         {
00648           index=k;
00649           break;
00650         }
00651       }
00652       if (index!=-1)
00653       {
00654         PolyRefData[index][1]++;
00655       }
00656       else
00657       {
00658         // don't have this one so get the height & type to the PolyDatas arrays
00659         PolyRefData[NoPolygons][0]=PRef;
00660         PolyRefData[NoPolygons][1]=1;
00661         NoPolygons++;
00662       }
00663     }
00664   }
00665   // C Loop
00666   for (int i=0; i<Cfinx; i++)
00667   {
00668     for (int j=0; j<yextent1; j++)
00669     {
00670       // Get the polyref
00671       int PRef = m_OurLandscape->SupplyPolyRef(i,j);
00672       // check if we have had this one already
00673       int index=-1;
00674       for (int k=0; k<NoPolygons; k++)
00675       {
00676         if (PolyRefData[k][0]==PRef)
00677         {
00678           index=k;
00679           break;
00680         }
00681       }
00682       if (index!=-1)
00683       {
00684         PolyRefData[index][1]++;
00685       }
00686       else
00687       {
00688         // Don't have this one so get the height & type to the PolyDatas arrays
00689         PolyRefData[NoPolygons][0]=PRef;
00690         PolyRefData[NoPolygons][1]=1;
00691         NoPolygons++;
00692       }
00693     }
00694   }
00695   // D Loop
00696   for (int i=x; i<Dfinx; i++)
00697   {
00698     for (int j=y; j<Dfiny; j++)
00699     {
00700       // Get the polyref
00701       int PRef = m_OurLandscape->SupplyPolyRef(i,j);
00702       // check if we have had this one already
00703       int index=-1;
00704       for (int k=0; k<NoPolygons; k++)
00705       {
00706         if (PolyRefData[k][0]==PRef)
00707         {
00708           index=k;
00709           break;
00710         }
00711       }
00712       if (index!=-1)
00713       {
00714         PolyRefData[index][1]++;
00715       }
00716       else
00717       {
00718         // don't have this one so get the height & type to the PolyDatas arrays
00719         PolyRefData[NoPolygons][0]=PRef;
00720         PolyRefData[NoPolygons][1]=1;
00721         NoPolygons++;
00722       }
00723     }
00724   }
00725   // End of search algorithm
00726     // for each polygon get the quality and multiply by amount of
00727     // that polygon in the territory
00728     // sum this to get overall quality
00729     double bestqual=-2;
00730     unsigned found=0;
00731     for ( int i=0; i<NoPolygons; i++)
00732     {
00733        double qual = AssessHabitat(PolyRefData[i][0]);
00734        if (bestqual<qual)
00735        {
00736          bestqual=qual;
00737          found = i;
00738        }
00739        quality+=PolyRefData[i][1]*qual;
00740     }
00741     // now find a location in polygon i
00742   // D Loop
00743   for (int i=x; i<Dfinx; i++)
00744   {
00745     for (int j=y; j<Dfiny; j++)
00746     {
00747       // Get the polyref
00748        if (m_OurLandscape->SupplyPolyRef(i,j)==PolyRefData[found][0])
00749       {
00750         p_stand_x = p_x;
00751         p_stand_y = p_y;
00752         return quality;
00753       }
00754     }
00755   }
00756   // A Loop
00757   for (int i=0; i<Afinx; i++)
00758   {
00759     for (int j=y; j<Afiny; j++)
00760     {
00761       // Get the polyref
00762       if (m_OurLandscape->SupplyPolyRef(i,j)==PolyRefData[found][0])
00763       {
00764         p_stand_x = p_x;
00765         p_stand_y = p_y;
00766         return quality;
00767       }
00768     }
00769   }
00770   // B Loop
00771   for (int i=x; i<Bfinx; i++)
00772   {
00773     for (int j=0; j<yextent1; j++)
00774     {
00775       // Get the polyref
00776       if (m_OurLandscape->SupplyPolyRef(i,j)==PolyRefData[found][0])
00777       {
00778         p_stand_x = p_x;
00779         p_stand_y = p_y;
00780         return quality;
00781       }
00782     }
00783   }
00784   // C Loop
00785   for (int i=0; i<Cfinx; i++)
00786   {
00787     for (int j=0; j<yextent1; j++)
00788     {
00789       if (m_OurLandscape->SupplyPolyRef(i,j)==PolyRefData[found][0])
00790       {
00791         p_stand_x = p_x;
00792         p_stand_y = p_y;
00793         return quality;
00794       }
00795     }
00796   }
00797   m_OurLandscape->Warn("Vole_Base::CalculateCarryingCapacity - error",NULL);
00798   exit(1);
00799 }
```

|  |  |  |  |
| --- | --- | --- | --- |
| double Vole\_Base::CalculateCarryingCapacity | ( | int | *p\_x*, |
|  |  | int | *p\_y* |  |
|  | ) |  |  | `[protected]` |

Do an assessment of the quality of the voles territory. Returns the sum of the qualities in the area covered by the territory (even if the vole does not have one)   
  
Uses the algorithm for fast searching of square arrays with wrap around co-ordinates.   
parameters = x,y starting coordinates top left   
range = extent of the space to search   
bottom left coordinate is therefore x+range, y+range   
22/09/2000   
  
For each polygon it gets the quality and multiplies by area of that polygon in the territory. This is summed for all polygons in the territoryto get overall quality.

References AssessHabitat(), TAnimal::m\_OurLandscape, m\_TerrRange, SimH, and SimW.

Referenced by Vole\_Male::CanFeed(), Vole\_Male::Dispersal(), Vole\_Female::Dispersal(), Vole\_Male::st\_Eval\_n\_Explore(), Vole\_Female::st\_Evaluate\_n\_Explore(), and Vole\_Female::st\_Special\_Explore().

```
00331 {
00332   int NoPolygons = 0;
00333   int PolyRefData[500][2];
00334   // First convert centre co-rdinates to square co-ordinates
00335   double quality = 0;
00336   int x=p_x - m_TerrRange;
00337   if (x<0) x+=SimW;
00338   int y=p_y - m_TerrRange;
00339   if (y<0) y+=SimH;
00340   int range_x=m_TerrRange+m_TerrRange;
00341   int range_y=m_TerrRange+m_TerrRange;
00342   // Stage 1 make a list of polygons
00343   // create the extent variables
00344   int xextent0 = x+range_x;
00345   int yextent0 = y+range_y;
00346   int xextent1 = (x+range_x)-SimW;
00347   int yextent1 = (y+range_y)-SimH;
00348   // Create the looping variables needed
00349   int Dfinx=xextent0;
00350   int Dfiny=yextent0;
00351   int Afinx=0;  // unless the finx values for A-C are changed
00352   int Bfinx=0;  // the value of zero will stop the A-C loops from executing
00353   int Cfinx=0;
00354   int Afiny=0; // this one assigned 0 to stop compiler complaints
00355   // Now create the loop values;
00356   if (x+range_x<=SimW)
00357   {
00358     // Type B & D (overlap bottom, no overlap)
00359     if (yextent0>SimH)
00360     {
00361       // Type B (overlap bottom only)
00362       Dfiny=SimH;
00363       Bfinx=x+range_x;
00364     }
00365   }
00366   else
00367   {
00368     // Type A & C overlap left edge or bottom & left
00369     if (yextent0>SimH)
00370     {
00371       // Type C overlap bottom and left
00372       Afinx=xextent1;
00373       Afiny=SimH;
00374       Bfinx=SimW;
00375       Cfinx=xextent1;
00376       Dfinx=SimW;
00377       Dfiny=SimH;
00378     }
00379     else
00380     {
00381       // Type A overlap left edge
00382       Afinx=xextent1;
00383       Afiny=yextent0;
00384       Dfinx=SimW;
00385     }
00386   }
00387   // the default is:
00388   // Type D no overlap
00389 
00390 
00391   // A Loop
00392   for (int i=0; i<Afinx; i++)
00393   {
00394     for (int j=y; j<Afiny; j++)
00395     {
00396       // Get the polyref
00397       int PRef = m_OurLandscape->SupplyPolyRef(i,j);
00398       // check if we have had this one already
00399       int index=-1;
00400       for (int k=0; k<NoPolygons; k++)
00401       {
00402         if (PolyRefData[k][0]==PRef)
00403         {
00404           index=k;
00405           break;
00406         }
00407       }
00408       if (index!=-1)
00409       {
00410         PolyRefData[index][1]++;
00411       }
00412       else
00413       {
00414         // don't have this one so get the height & type to the PolyDatas arrays
00415         PolyRefData[NoPolygons][0]=PRef;
00416         PolyRefData[NoPolygons][1]=1;
00417         NoPolygons++;
00418       }
00419     }
00420   }
00421   // B Loop
00422   for (int i=x; i<Bfinx; i++)
00423   {
00424     for (int j=0; j<yextent1; j++)
00425     {
00426       // Get the polyref
00427       int PRef = m_OurLandscape->SupplyPolyRef(i,j);
00428       // check if we have had this one already
00429       int index=-1;
00430       for (int k=0; k<NoPolygons; k++)
00431       {
00432         if (PolyRefData[k][0]==PRef)
00433         {
00434           index=k;
00435           break;
00436         }
00437       }
00438       if (index!=-1)
00439       {
00440         PolyRefData[index][1]++;
00441       }
00442       else
00443       {
00444         // don't have this one so get the height & type to the PolyDatas arrays
00445         PolyRefData[NoPolygons][0]=PRef;
00446         PolyRefData[NoPolygons][1]=1;
00447         NoPolygons++;
00448       }
00449     }
00450   }
00451   // C Loop
00452   for (int i=0; i<Cfinx; i++)
00453   {
00454     for (int j=0; j<yextent1; j++)
00455     {
00456       // Get the polyref
00457       int PRef = m_OurLandscape->SupplyPolyRef(i,j);
00458       // check if we have had this one already
00459       int index=-1;
00460       for (int k=0; k<NoPolygons; k++)
00461       {
00462         if (PolyRefData[k][0]==PRef)
00463         {
00464           index=k;
00465           break;
00466         }
00467       }
00468       if (index!=-1)
00469       {
00470         PolyRefData[index][1]++;
00471       }
00472       else
00473       {
00474         // Don't have this one so get the height & type to the PolyDatas arrays
00475         PolyRefData[NoPolygons][0]=PRef;
00476         PolyRefData[NoPolygons][1]=1;
00477         NoPolygons++;
00478       }
00479     }
00480   }
00481   // D Loop
00482   for (int i=x; i<Dfinx; i++)
00483   {
00484     for (int j=y; j<Dfiny; j++)
00485     {
00486       // Get the polyref
00487       int PRef = m_OurLandscape->SupplyPolyRef(i,j);
00488       // check if we have had this one already
00489       int index=-1;
00490       for (int k=0; k<NoPolygons; k++)
00491       {
00492         if (PolyRefData[k][0]==PRef)
00493         {
00494           index=k;
00495           break;
00496         }
00497       }
00498       if (index!=-1)
00499       {
00500         PolyRefData[index][1]++;
00501       }
00502       else
00503       {
00504         // don't have this one so get the height & type to the PolyDatas arrays
00505         PolyRefData[NoPolygons][0]=PRef;
00506         PolyRefData[NoPolygons][1]=1;
00507         NoPolygons++;
00508       }
00509     }
00510   }
00511   // End of search algorithm
00512     /* for each polygon get the quality and multiply by amount of that polygon in the territory sum this to get overall quality */
00513     for ( int i=0; i<NoPolygons; i++)
00514     {
00515        quality+= PolyRefData[i][1]*AssessHabitat(PolyRefData[i][0]);
00516     }
00517     // return the total quality
00518     return quality;
00519 
00520 }
```

|  |  |  |  |  |  |
| --- | --- | --- | --- | --- | --- |
| void Vole\_Base::CopyMyself | ( | int | *a\_vtype* | ) |  |

Duplicates a vole.

Method used to duplicate a vole - most commonly used for experimental manipulation of populations e.g. return rate experiments

References Vole\_Population\_Manager::CreateObjects(), struct\_Vole\_Adult::Genes, struct\_Vole\_Adult::L, struct\_Vole\_Adult::m\_flag, TAnimal::m\_Location\_x, TAnimal::m\_Location\_y, TAnimal::m\_OurLandscape, m\_OurPopulation, MyGenes, GeneticMaterial::Recombine(), Population\_Manager::SimH, Population\_Manager::SimW, struct\_Vole\_Adult::VPM, struct\_Vole\_Adult::x, and struct\_Vole\_Adult::y.

Referenced by Vole\_Population\_Manager::Catastrophe().

```
00256                                       {
00257       struct_Vole_Adult* av;
00258       av = new struct_Vole_Adult;
00259       av->VPM = m_OurPopulation;
00260       av->L = m_OurLandscape;
00261       av->m_flag=true; // Used to signal pesticide effect to CreateObjects
00262         av->x = ((m_Location_x+1)%m_OurPopulation->SimW);
00263         av->y = ((m_Location_y)%m_OurPopulation->SimH);
00264         // Do the genetics
00265         av->Genes.Recombine(&MyGenes,&MyGenes);
00266         m_OurPopulation->CreateObjects(a_vtype,this,av,1);
00267   // object will be destroyed by death state
00268   // but must let Dad know anyway
00269   delete av;
00270 }
```

|  |  |  |  |
| --- | --- | --- | --- |
| void Vole\_Base::DoWalking | ( | int | *p\_Distance*, |
|  |  | int & | *p\_Vector*, |
|  |  | int & | *vx*, |
|  |  | int & | *vy* |  |
|  | ) |  |  | `[protected]` |

Walking.

This method does the actual stepping - there is no look ahead here, so steps are taken one at a time based on the habitat type and vector given.

References g\_rand\_uni, MoveQuality(), MoveToLessFavourable, Vector\_x, and Vector\_y.

Referenced by MoveTo().

```
00860 {
00861     int t[5], q[5];
00862     for (int i=0; i<p_Distance; i++)
00863     {
00864       // test the squares at Vector, Vector+1+2, Vector-1-2
00865       // They have either a quality or are inaccessible (water,buildings)
00866       // if can go to one of these squares then pick the best one
00867       // if all or some are equal then take a random pick.
00868       // if all are 'bad' then add or subtract one from vector and try again
00869 
00870       t[0] = p_Vector;
00871       t[1] = (p_Vector+1) & 0x07;
00872       t[2] = (p_Vector+7) & 0x07;
00873       t[3] = (p_Vector+2) & 0x07;
00874       t[4] = (p_Vector+6) & 0x07;
00875       q[0] = MoveQuality((vx+Vector_x[t[0]]), (vy+Vector_y[t[0]]));
00876       q[1] = MoveQuality((vx+Vector_x[t[1]]),(vy+Vector_y[t[1]]));
00877       q[2] = MoveQuality((vx+Vector_x[t[2]]),(vy+Vector_y[t[2]]));
00878       q[3] = MoveQuality((vx+Vector_x[t[3]]),(vy+Vector_y[t[3]]));
00879       q[4] = MoveQuality((vx+Vector_x[t[4]]),(vy+Vector_y[t[4]]));
00880       if (g_rand_uni() < MoveToLessFavourable)
00881       {
00882         // allow a mistake once in a while
00883         for (int j=1; j<5; j++) q[j]=-1;
00884       }
00885       // Now pick the best of these
00886       int best = q[0];
00887       int score=1;
00888       for (int i=1; i<5; i++)
00889       {
00890         if (q[i]>best)
00891         {
00892           best=q[i];
00893           score=1;
00894         }
00895         else
00896         if (q[i]==best) score++;
00897       }
00898       if (best==-1)
00899       {
00900         // can't go anywhere so change the vector
00901         if (random(2)) ++p_Vector; else (--p_Vector);
00902         p_Vector&=0x07;
00903       }
00904       else
00905       {
00906         // Can go to one of score squares
00907         int scored=random(score); // pick one
00908         int loop=0;
00909         for (int i=0; i<5; i++)
00910         {
00911           if (best==q[i]) loop++; // count the squares with 'best' quality
00912           if (loop>scored)
00913           {
00914             loop=i; // go to i-square
00915             break;
00916           }
00917         }
00918         // change co-ordinates
00919         vx+=Vector_x[t[loop]];
00920         vy+=Vector_y[t[loop]];
00921       }
00922     }
00923 }
```

|  |  |  |  |
| --- | --- | --- | --- |
| void Vole\_Base::DoWalkingCorrect | ( | int | *p\_Distance*, |
|  |  | int & | *p\_Vector*, |
|  |  | int & | *vx*, |
|  |  | int & | *vy* |  |
|  | ) |  |  | `[protected]` |

Walking where there is a danger of stepping off the world.

This method does the actual stepping - there is no look ahead here, so steps are taken one at a time based on the habitat type and vector given.   
This version corrects coords for wrap around. This is slower so is only called when necessary.

References g\_rand\_uni, MoveQuality(), MoveToLessFavourable, SimH, SimW, Vector\_x, and Vector\_y.

Referenced by MoveTo().

```
00934 {
00935     int t[5], q[5];
00936     for (int i=0; i<p_Distance; i++)
00937     {
00938       // test the squares at Vector, Vector+1+2, Vector-1-2
00939       // They have either a quality or are inaccessible (water,buildings)
00940       // if can go to one of these squares then pick the best one
00941       // if all or some are equal then take a random pick.
00942       // if all are 'bad' then add or subtract one from vector and try again
00943 
00944       t[0] = p_Vector;
00945       t[1] = (p_Vector+1) & 0x07;
00946       t[2] = (p_Vector+7) & 0x07;
00947       t[3] = (p_Vector+2) & 0x07;
00948       t[4] = (p_Vector+6) & 0x07;
00949       q[0] = MoveQuality((vx+Vector_x[t[0]])%SimW, (vy+Vector_y[t[0]])%SimH);
00950       q[1] = MoveQuality((vx+Vector_x[t[1]])%SimW,(vy+Vector_y[t[1]])%SimH);
00951       q[2] = MoveQuality((vx+Vector_x[t[2]])%SimW,(vy+Vector_y[t[2]])%SimH);
00952       q[3] = MoveQuality((vx+Vector_x[t[3]])%SimW,(vy+Vector_y[t[3]])%SimH);
00953       q[4] = MoveQuality((vx+Vector_x[t[4]])%SimW,(vy+Vector_y[t[4]])%SimH);
00954       if (g_rand_uni()<MoveToLessFavourable)
00955       {
00956         // allow a mistake once in a while
00957         for (int j=1; j<5; j++) q[j]=-1;
00958       }
00959       // Now pick the best of these
00960       int best = q[0];
00961       int score=1;
00962       for (int i=1; i<5; i++)
00963       {
00964         if (q[i]>best)
00965         {
00966           best=q[i];
00967           score=1;
00968         }
00969         else
00970         if (q[i]==best) score++;
00971       }
00972       if (best==-1)
00973       {
00974         // can't go anywhere so change the vector
00975         if (random(2)) ++p_Vector; else (--p_Vector);
00976         p_Vector&=0x07;
00977       }
00978       else
00979       {
00980         // Can go to one of score squares
00981         int scored=random(score); // pick one
00982         int loop=0;
00983         for (int i=0; i<5; i++)
00984         {
00985           if (best==q[i]) loop++; // count the squares with 'best' quality
00986           if (loop>scored)
00987           {
00988             loop=i; // go to i-square
00989             break;
00990           }
00991         }
00992         // change co-ordinates
00993         vx+=Vector_x[t[loop]];
00994         vy+=Vector_y[t[loop]];
00995       }
00996     }
00997 }
```

|  |  |  |  |  |  |
| --- | --- | --- | --- | --- | --- |
| void Vole\_Base::EndStep | ( | void |  | ) | `[virtual]` |

All voles age at the end of the day.

Reimplemented from TAnimal.

Reimplemented in Vole\_Male, and Vole\_Female.

References m\_Age.

```
00277 {
00278     m_Age++; // Once a day increment m_Age
00279 }
```

|  |  |  |  |
| --- | --- | --- | --- |
| void Vole\_Base::Escape | ( | int | *p\_Vector*, |
|  |  | int | *p\_Distance* |  |
|  | ) |  |  | `[protected]` |

Dispersal - directed movement.

This works like MoveTo above, but with a rather more directed movement aimed at moving further from the start point.

References FreeLocation(), TAnimal::m\_Location\_x, TAnimal::m\_Location\_y, MoveQuality(), SetLocation(), SimH, SimW, Vector\_x, and Vector\_y.

```
01008 {
01009     // This will alter m_Location_x & m_Location_y
01010     // it will give a very directed movement towards p_Vector
01011 
01012     // p_Vector gives the preferred direction (0-7)
01013     // p_Distance is the number of steps
01014     int vx = m_Location_x+SimW;
01015     int vy = m_Location_y+SimH;
01016 //    int counter = 0;
01017     for (int i=0; i<p_Distance; i++)
01018     {
01019       // test the squares at Vector, Vector+1+2, Vector-1-2
01020       // They have either a quality or are inaccessible (water,buildings)
01021       // if can go to one of these squares then pick the best one
01022       // if all or some are equal then take a random pick.
01023       // if all are 'bad' then add or subtract one from p_Vector and try again
01024 //      counter ++;
01025       int t[3], q[3];
01026       t[0] = p_Vector;
01027       t[1] = (p_Vector+1) & 0x07;
01028       t[2] = (p_Vector+7) & 0x07;
01029       q[0] = MoveQuality((vx+Vector_x[t[0]])%SimW,(vy+Vector_y[t[0]])%SimH);
01030       q[1] = MoveQuality((vx+Vector_x[t[1]])%SimW,(vy+Vector_y[t[1]])%SimH);
01031       q[2] = MoveQuality((vx+Vector_x[t[2]])%SimW,(vy+Vector_y[t[2]])%SimH);
01032       // Now pick the best of these
01033       int noscore=0;
01034       for (int i=0; i<3; i++)
01035       {
01036         if (q[i]==-1)
01037         {
01038           noscore++;
01039         }
01040       }
01041       if (noscore==3)
01042       {
01043         // can't go anywhere so change the vector
01044         if (random(2)) p_Vector++; else p_Vector--;
01045       }
01046       else
01047       {
01048         // Can go to at least one of score squares
01049         // try the middle first
01050         int loop=0;
01051         if (q[0]==-1)
01052         {
01053           // otherwise randomly try one side or other
01054           // it must be possible to go to one of these
01055           int which=random(2);
01056           if (which==1)
01057           {
01058             if (q[1]!=-1) loop=1; else loop=2;
01059           }
01060           else
01061           {
01062             if (q[2]!=-1) loop=2; else loop=1;
01063           }
01064         }
01065         // change co-ordinates
01066         vx+=Vector_x[t[loop]];
01067         vy+=Vector_y[t[loop]];
01068       }
01069     }
01070   // alter the voles location
01071   FreeLocation();
01072   m_Location_x=vx%SimW;
01073   m_Location_y=vy%SimH;
01074   SetLocation();
01075 }
```

|  |  |  |  |  |
| --- | --- | --- | --- | --- |
| virtual void Vole\_Base::FreeLocation | ( |  | ) | `[inline, protected, virtual]` |

Reimplemented in Vole\_Male, and Vole\_Female.

Referenced by Escape(), MoveTo(), and st\_Dying().

```
00194 {};
```

|  |  |  |  |  |
| --- | --- | --- | --- | --- |
| int Vole\_Base::GetDirectFlag | ( |  | ) | `[inline]` |

Genetic functionality

References GeneticMaterial::GetDirectFlag(), and MyGenes.

Referenced by Vole\_Population\_Manager::ImpactedProbe(), and Vole\_Population\_Manager::TheReallyBigOutputProbe().

```
00157 { return MyGenes.GetDirectFlag(); }
```

|  |  |  |  |  |
| --- | --- | --- | --- | --- |
| int Vole\_Base::GetGeneticFlag | ( |  | ) | `[inline]` |

Genetic functionality

References GeneticMaterial::GetGeneticFlag(), and MyGenes.

Referenced by Vole\_Population\_Manager::ImpactedProbe(), and Vole\_Population\_Manager::TheReallyBigOutputProbe().

```
00155 { return MyGenes.GetGeneticFlag(); }
```

|  |  |  |  |
| --- | --- | --- | --- |
| virtual bool Vole\_Base::GetLocation | ( | int | , |
|  |  | int |  |  |
|  | ) |  |  | `[inline, protected, virtual]` |

Reimplemented in Vole\_Male, and Vole\_Female.

Referenced by MoveTo().

```
00195 {return false;};
```

|  |  |  |  |  |
| --- | --- | --- | --- | --- |
| bool Vole\_Base::MortalityTest | ( |  | ) |  |

Do a mortality test.

Takes both physiological lifespan and background mortality into account to determine whether the vole should die - repeated calls increase the risk of dying

References g\_rand\_uni, and m\_LifeSpan.

Referenced by Vole\_Male::BeginStep(), and Vole\_Female::BeginStep().

```
00306 {
00307   // returns true if the vole should die
00308   if (--m_LifeSpan<1)
00309   {
00310    return true;
00311   }
00312   if (g_rand_uni()<DailyMortChance) return true;
00313   return false;
00314 }
```

|  |  |  |  |
| --- | --- | --- | --- |
| int Vole\_Base::MoveQuality | ( | int | *p\_x*, |
|  |  | int | *p\_y* |  |
|  | ) |  |  | `[protected]` |

Test a location for quality while moving.

Returns the quality of a patch of habitat at p\_x,p\_y.   
Can't walk through another vole though - so test location first.

References TAnimal::m\_OurLandscape, m\_OurPopulation, tole\_ActivePit, tole\_Building, tole\_Coast, tole\_ConiferousForest, tole\_DeciduousForest, tole\_Field, tole\_FieldBoundary, tole\_Foobar, tole\_Freshwater, tole\_Garden, tole\_Heath, tole\_HedgeBank, tole\_Hedges, tole\_LargeRoad, tole\_Marsh, tole\_MixedForest, tole\_NaturalGrass, tole\_Orchard, tole\_PermanentSetaside, tole\_PermPasture, tole\_PermPastureLowGrazing, tole\_PitDisused, tole\_Railway, tole\_River, tole\_RiversidePlants, tole\_RiversideTrees, tole\_RoadsideVerge, tole\_Saltwater, tole\_Scrub, tole\_SmallRoad, tole\_StoneWall, tole\_Track, tole\_UnsprayedFieldMargin, tole\_Urban, tole\_YoungForest, tov\_CloverGrassGrazed1, tov\_CloverGrassGrazed2, tov\_OCloverGrassGrazed1, tov\_OCloverGrassGrazed2, tov\_OCloverGrassSilage1, tov\_OPermanentGrassGrazed, tov\_PermanentGrassGrazed, and Vole\_Population\_Manager::VoleMap.

Referenced by DoWalking(), DoWalkingCorrect(), and Escape().

```
01293 {
01294     if (m_OurPopulation->VoleMap->GetMapValue(p_x,p_y)) return -1;
01295     // Nobody there so carry on
01296     int polyref=m_OurLandscape->SupplyPolyRef(p_x,p_y);
01297   switch (m_OurLandscape->SupplyElementType(polyref))
01298   {
01299     case tole_Heath:
01300     case tole_Hedges: // 130
01301      return 3;
01302     case tole_RoadsideVerge: // 13
01303      return 4;
01304     case tole_Railway: // 118
01305      return 3;
01306     case tole_FieldBoundary: // 160
01307      return 4;
01308     case tole_Marsh: // 95
01309      return 2;
01310     case tole_Scrub: // 70
01311      return 1;
01312     case tole_PermPasture: // 35
01313      if (m_OurLandscape->SupplyGrazingPressure(polyref))return 2;
01314      else return 4;
01315     case tole_PermPastureLowGrazing: 
01316      if (m_OurLandscape->SupplyGrazingPressure(polyref))return 3;
01317      else return 4;
01318     case tole_Orchard:
01319     return 3;
01320     case tole_NaturalGrass: // 110
01321     case tole_HedgeBank:    // just as good
01322      return 4;
01323     case tole_PermanentSetaside:
01324      return 4;
01325     case tole_RiversidePlants: // 98
01326      return 2;
01327     case tole_PitDisused: // 75
01328      return 2;
01329     case tole_RiversideTrees: // 97
01330      return 1;
01331     case tole_DeciduousForest: // 40
01332      return 1;
01333     case tole_YoungForest: // 60
01334      return 2;
01335     case tole_MixedForest: // 60
01336      return 1;
01337     case tole_ConiferousForest: // 50
01338      return 1;
01339     case tole_StoneWall: // 15
01340      return -1;
01341     case tole_Garden: //11
01342      return 0;
01343     case tole_Track: // 123
01344      return 0;
01345     case tole_SmallRoad: // 122
01346      return 0;
01347     case tole_LargeRoad: // 121
01348      return 0;
01349     case tole_Building: // 5
01350     case tole_Urban: // 10
01351     case tole_ActivePit: // 115
01352     case tole_Freshwater: // 90
01353     case tole_River: // 96
01354     case tole_Saltwater: // 80
01355     case tole_Coast: // 100
01356      return -1;
01357     case tole_Field: // 20 & 30
01358     case tole_UnsprayedFieldMargin:
01359       {
01360        TTypesOfVegetation  VType=m_OurLandscape->SupplyVegType(polyref);
01361            switch (VType) {
01362        case tov_OCloverGrassSilage1:
01363        case tov_CloverGrassGrazed1:
01364        case tov_CloverGrassGrazed2:
01365        case tov_OCloverGrassGrazed1:
01366        case tov_OCloverGrassGrazed2:
01367        case tov_OPermanentGrassGrazed:
01368        case tov_PermanentGrassGrazed:
01369                 if (m_OurLandscape->SupplyGrazingPressure(polyref))return 2;
01370                         else return 4;  
01371                 break;
01372            default:
01373                 double cover = m_OurLandscape->SupplyVegCover(polyref);
01374                 double height = m_OurLandscape->SupplyVegHeight(polyref);
01375                 if ((cover>0.80) && (height>40)) return 2;
01376                 if ((cover<0.50) || (height<10)) return 0;
01377                         else return 2;
01378                 }
01379           }
01380     case tole_Foobar: // 999 !! type unknown - should not happen
01381     default:
01382      static char errornum[20];
01383      sprintf(errornum, "%d", m_OurLandscape->SupplyElementType(polyref));
01384      m_OurLandscape->Warn("Vole_Base:AssessHabitat: Unknown tole_type",
01385      errornum);
01386      exit(1);
01387   }
01388 }
```

|  |  |  |  |
| --- | --- | --- | --- |
| void Vole\_Base::MoveTo | ( | int | *p\_Vector*, |
|  |  | int | *p\_Distance*, |
|  |  | int | *p\_iterations* |  |
|  | ) |  |  | `[protected]` |

Movement.

This will alter m\_Location\_x & m\_Location\_y.   
It will give a rather directed movement towards p\_Vector.   
Generally the vole will stay in the best habitat, but occasional mis-steps occur.  
p\_Vector gives the preferred direction (0-7), p\_Distance is the number of steps.

References DoWalking(), DoWalkingCorrect(), FreeLocation(), GetLocation(), TAnimal::m\_Location\_x, TAnimal::m\_Location\_y, SetLocation(), SimH, and SimW.

Referenced by Vole\_Male::Dispersal(), Vole\_Female::Dispersal(), Vole\_Male::st\_JuvenileExplore(), and Vole\_Female::st\_Special\_Explore().

```
00813 {
00814   int offset=p_Distance*p_iterations;
00815   if ((m_Location_x-offset<0) ||(m_Location_x+offset>=SimW) ||
00816          (m_Location_y-offset<0) ||(m_Location_y+offset>=SimH))
00817   {
00818     // Need correct coords
00819     // Make sure that the coords can't become -ve
00820     int vx = m_Location_x+SimW;
00821     int vy = m_Location_y+SimH;
00822     do
00823     {
00824       DoWalkingCorrect(p_Distance,p_Vector,vx,vy);
00825       p_Distance=1;
00826     } while ((GetLocation(vx%SimW,vy%SimH)) && (p_iterations-->0));
00827     // alter the voles location (& correct coords)
00828     FreeLocation();
00829     m_Location_x=vx%SimW;
00830     m_Location_y=vy%SimH;
00831     SetLocation();
00832   }
00833   else
00834   {
00835     // Dont need correct coords
00836     int vx = m_Location_x;
00837     int vy = m_Location_y;
00838     do
00839     {
00840       DoWalking(p_Distance,p_Vector,vx,vy);
00841       p_Distance=1;
00842     } while ((GetLocation(vx,vy)) && (p_iterations-->0));
00843     // alter the voles location (& correct coords)
00844     FreeLocation();
00845     m_Location_x=vx;
00846     m_Location_y=vy;
00847     SetLocation();
00848   }
00849 }
```

|  |  |  |  |  |
| --- | --- | --- | --- | --- |
| virtual void Vole\_Base::OnKilled | ( |  | ) | `[inline, virtual]` |

Reimplemented in Vole\_Male, and Vole\_Female.

```
00168 {};
```

|  |  |  |  |  |  |
| --- | --- | --- | --- | --- | --- |
| void Vole\_Base::Set\_Age | ( | int | *Age* | ) | `[inline]` |

Set our age

References m\_Age.

Referenced by Vole\_Population\_Manager::CreateObjects\_Init().

```
00134 {m_Age=Age;}
```

|  |  |  |  |  |
| --- | --- | --- | --- | --- |
| void Vole\_Base::SetDirectFlag | ( |  | ) | `[inline]` |

Genetic functionality

References MyGenes, and GeneticMaterial::SetDirectFlag().

Referenced by Vole\_Population\_Manager::CreateObjects().

```
00161 { MyGenes.SetDirectFlag(); }
```

|  |  |  |  |  |
| --- | --- | --- | --- | --- |
| void Vole\_Base::SetGeneticFlag | ( |  | ) | `[inline]` |

Genetic functionality

References MyGenes, and GeneticMaterial::SetGeneticFlag().

Referenced by Vole\_Population\_Manager::CreateObjects().

```
00159 { MyGenes.SetGeneticFlag(); }
```

|  |  |  |  |  |
| --- | --- | --- | --- | --- |
| virtual void Vole\_Base::SetLocation | ( |  | ) | `[inline, protected, virtual]` |

Reimplemented in Vole\_Male, and Vole\_Female.

Referenced by Escape(), and MoveTo().

```
00193 {};
```

|  |  |  |  |  |
| --- | --- | --- | --- | --- |
| void Vole\_Base::Setm\_Mature | ( |  | ) | `[inline]` |

Become adult

References m\_Mature.

Referenced by Vole\_Population\_Manager::CreateObjects\_Init().

```
00132 {m_Mature=true;}
```

|  |  |  |  |  |  |
| --- | --- | --- | --- | --- | --- |
| void Vole\_Base::SetWeight | ( | double | *W* | ) | `[inline]` |

Set our weight

References m\_Weight.

Referenced by Vole\_Population\_Manager::CreateObjects\_Init().

```
00130 {m_Weight=W;}
```

|  |  |  |  |  |
| --- | --- | --- | --- | --- |
| void Vole\_Base::st\_Dying | ( |  | ) |  |

All voles end here on death.

Called when a vole dies. Just removes itself from the map and sets a flag to destroy the object in the endStep

References TALMaSSObject::CurrentStateNo, FreeLocation(), Population\_Manager::LamdaDeath(), TAnimal::m\_Location\_x, TAnimal::m\_Location\_y, and m\_OurPopulation.

Referenced by Vole\_Male::EndStep(), and Vole\_Female::EndStep().

```
00289 {
00290    FreeLocation();
00291    CurrentStateNo=-1;
00292 #ifdef __LAMBDA_RECORD
00293    m_OurPopulation->LamdaDeath(m_Location_x, m_Location_y);
00294 #endif
00295 }
```

|  |  |  |  |  |  |
| --- | --- | --- | --- | --- | --- |
| virtual void Vole\_Base::Step | ( | void |  | ) | `[inline, virtual]` |

Reimplemented from TAnimal.

Reimplemented in Vole\_Male, and Vole\_Female.

```
00126 {};
```

|  |  |  |  |  |
| --- | --- | --- | --- | --- |
| unsigned Vole\_Base::SupplyAge | ( |  | ) | `[inline]` |

Tell our age

References m\_Age.

Referenced by Vole\_Population\_Manager::SendMessage(), Vole\_Population\_Manager::SupplyInOlderTerr(), Vole\_Population\_Manager::SupplyOlderFemales(), and Vole\_Population\_Manager::TheReallyBigOutputProbe().

```
00142 {return m_Age;};
```

|  |  |  |  |
| --- | --- | --- | --- |
| int Vole\_Base::SupplyAllele | ( | int | *locus*, |
|  |  | int | *allele* |  |
|  | ) |  |  | `[inline]` |

Genetic functionality

References GeneticMaterial::GetAllele(), and MyGenes.

Referenced by Vole\_Population\_Manager::GeneticsResultsOutput().

```
00153                                       {return MyGenes.GetAllele(locus,allele);}
```

|  |  |  |  |  |
| --- | --- | --- | --- | --- |
| GeneticMaterial Vole\_Base::SupplyGenes | ( |  | ) | `[inline]` |

Genetic functionality

References MyGenes.

Referenced by Vole\_Female::st\_Mating().

```
00167 {return MyGenes;}
```

|  |  |  |  |  |
| --- | --- | --- | --- | --- |
| int Vole\_Base::SupplyHeteroZyg | ( |  | ) | `[inline]` |

Genetic functionality

References GeneticMaterial::HeterozygosityCount(), and MyGenes.

```
00150 {return MyGenes.HeterozygosityCount();}
```

|  |  |  |  |  |
| --- | --- | --- | --- | --- |
| int Vole\_Base::SupplyHomoZyg | ( |  | ) | `[inline]` |

Genetic functionality

References GeneticMaterial::HomozygosityCount(), and MyGenes.

```
00148 {return MyGenes.HomozygosityCount();}
```

|  |  |  |  |  |
| --- | --- | --- | --- | --- |
| bool Vole\_Base::SupplySex | ( |  | ) | `[inline]` |

Tell our sex

References m\_Sex.

Referenced by Vole\_Population\_Manager::FindClosestFemale(), Vole\_Population\_Manager::FindClosestMale(), Vole\_Population\_Manager::ListClosestFemales(), Vole\_Population\_Manager::ListClosestMales(), Vole\_Population\_Manager::SupplyInOlderTerr(), and Vole\_Population\_Manager::SupplyOlderFemales().

```
00140 {return m_Sex;};
```

|  |  |  |  |  |
| --- | --- | --- | --- | --- |
| bool Vole\_Base::SupplyTerritorial | ( |  | ) | `[inline]` |

Tell whether we have a territory

References m\_Have\_Territory.

Referenced by Vole\_Population\_Manager::FindClosestFemale(), Vole\_Population\_Manager::FindClosestMale(), Vole\_Population\_Manager::ListClosestFemales(), Vole\_Population\_Manager::ListClosestMales(), Vole\_Population\_Manager::SupplyInOlderTerr(), Vole\_Population\_Manager::TheCIPEGridOutputProbe(), and Vole\_Population\_Manager::TheReallyBigOutputProbe().

```
00138 {return m_Have_Territory;}
```

|  |  |  |  |  |
| --- | --- | --- | --- | --- |
| unsigned Vole\_Base::SupplyX | ( |  | ) | `[inline]` |

Tell our x coordinate

References TAnimal::m\_Location\_x.

Referenced by Vole\_Population\_Manager::SendMessage().

```
00144 {return m_Location_x;};
```

|  |  |  |  |  |
| --- | --- | --- | --- | --- |
| unsigned Vole\_Base::SupplyY | ( |  | ) | `[inline]` |

Tell our y coordinate

References TAnimal::m\_Location\_y.

Referenced by Vole\_Population\_Manager::SendMessage().

```
00146 {return m_Location_y;};
```

|  |  |  |  |  |
| --- | --- | --- | --- | --- |
| void Vole\_Base::UnsetDirectFlag | ( |  | ) | `[inline]` |

Genetic functionality

References MyGenes, and GeneticMaterial::UnsetDirectFlag().

Referenced by Vole\_Population\_Manager::CreateObjects(), and Vole\_Population\_Manager::CreateObjects\_Init().

```
00165 { MyGenes.UnsetDirectFlag(); }
```

|  |  |  |  |  |
| --- | --- | --- | --- | --- |
| void Vole\_Base::UnsetGeneticFlag | ( |  | ) | `[inline]` |

Genetic functionality

References MyGenes, and GeneticMaterial::UnsetGeneticFlag().

Referenced by Vole\_Population\_Manager::CreateObjects(), and Vole\_Population\_Manager::CreateObjects\_Init().

```
00163 { MyGenes.UnsetGeneticFlag(); }
```

|  |  |  |  |  |
| --- | --- | --- | --- | --- |
| virtual int Vole\_Base::WhatState | ( |  | ) | `[inline, virtual]` |

Get our current vole state

Reimplemented from TAnimal.

References CurrentVState.

```
00136 {return CurrentVState;}
```

---

## Member Data Documentation

|  |
| --- |
| TTypeOfVoleState Vole\_Base::CurrentVState |

Our current behavioural state

Referenced by Vole\_Male::BeginStep(), Vole\_Female::BeginStep(), Vole\_Population\_Manager::Catastrophe(), Vole\_Male::EndStep(), Vole\_Female::EndStep(), Vole\_Male::OnFarmEvent(), Vole\_Female::OnFarmEvent(), Vole\_Male::OnKilled(), Vole\_Female::OnKilled(), Vole\_Male::Step(), Vole\_Female::Step(), Vole\_Base(), and WhatState().

|  |
| --- |
| unsigned Vole\_Base::IDNo `[protected]` |

Their individual ID number

Referenced by Vole\_Base().

|  |
| --- |
| int Vole\_Base::m\_Age `[protected]` |

Their age in days

Referenced by Vole\_Male::Dispersal(), Vole\_Female::Dispersal(), Vole\_Male::EndStep(), Vole\_Female::EndStep(), EndStep(), Vole\_Male::Init(), Vole\_Female::Init(), Set\_Age(), Vole\_Female::st\_BecomeReproductive(), Vole\_Male::st\_Eval\_n\_Explore(), Vole\_Male::st\_Maturation(), Vole\_Female::st\_Special\_Explore(), SupplyAge(), and Vole\_Base().

|  |
| --- |
| int Vole\_Base::m\_DispVector `[protected]` |

The current dispersal direction

Referenced by Vole\_Male::DecideQualityAction(), Vole\_Male::Dispersal(), Vole\_Female::Dispersal(), Vole\_Male::st\_Eval\_n\_Explore(), Vole\_Female::st\_Evaluate\_n\_Explore(), and Vole\_Base().

|  |
| --- |
| bool Vole\_Base::m\_Have\_Territory `[protected]` |

Do they have a terriory?

Referenced by Vole\_Male::DecideQualityAction(), Vole\_Male::Dispersal(), Vole\_Female::Dispersal(), Vole\_Male::st\_Eval\_n\_Explore(), Vole\_Female::st\_Evaluate\_n\_Explore(), Vole\_Female::st\_Special\_Explore(), SupplyTerritorial(), and Vole\_Base().

|  |
| --- |
| int Vole\_Base::m\_LifeSpan `[protected]` |

Their lifespan remaining (unless killed by external events)

Referenced by MortalityTest(), and Vole\_Base().

|  |
| --- |
| bool Vole\_Base::m\_Mature `[protected]` |

Whether they are mature or not

Referenced by Vole\_Male::Dispersal(), Vole\_Female::Dispersal(), Vole\_Male::EndStep(), Vole\_Female::EndStep(), Setm\_Mature(), Vole\_Female::st\_BecomeReproductive(), Vole\_Male::st\_Eval\_n\_Explore(), Vole\_Female::st\_Evaluate\_n\_Explore(), Vole\_Female::st\_Mating(), Vole\_Female::st\_ReproBehaviour(), Vole\_Female::st\_Special\_Explore(), Vole\_Male::Step(), and Vole\_Base().

|  |
| --- |
| Vole\_Population\_Manager\* Vole\_Base::m\_OurPopulation |

Referenced by CalculateCarryingCapacity(), CopyMyself(), Vole\_Male::Dispersal(), Vole\_Female::Dispersal(), Vole\_Male::EndStep(), Vole\_Female::EndStep(), Vole\_Male::FreeLocation(), Vole\_Female::FreeLocation(), Vole\_Male::GetLocation(), Vole\_Female::GetLocation(), MoveQuality(), Vole\_Male::SetLocation(), Vole\_Female::SetLocation(), Vole\_Female::st\_BecomeReproductive(), st\_Dying(), Vole\_Male::st\_Eval\_n\_Explore(), Vole\_Female::st\_Evaluate\_n\_Explore(), Vole\_Female::st\_GiveBirth(), Vole\_Male::st\_Infanticide(), Vole\_Female::st\_Lactating(), Vole\_Female::st\_Mating(), Vole\_Male::st\_Maturation(), Vole\_Female::st\_Special\_Explore(), and Vole\_Base().

|  |
| --- |
| bool Vole\_Base::m\_pesticideInfluenced `[protected]` |

For specific pesticide use only

Referenced by Vole\_Female::st\_Lactating(), Vole\_Female::st\_Mating(), Vole\_Female::st\_UpdateGestation(), and Vole\_Base().

|  |
| --- |
| bool Vole\_Base::m\_pesticideInfluenced2 |

For specific pesticide use only

Referenced by Vole\_Population\_Manager::CreateObjects(), Vole\_Female::st\_Lactating(), Vole\_Female::st\_Mating(), and Vole\_Base().

|  |
| --- |
| int Vole\_Base::m\_Reserves `[protected]` |

Their reserves - in days that they can survive without food

Referenced by Vole\_Male::DecideQualityAction(), Vole\_Male::Dispersal(), Vole\_Female::st\_Evaluate\_n\_Explore(), Vole\_Female::st\_GiveBirth(), Vole\_Female::st\_Lactating(), and Vole\_Base().

|  |
| --- |
| bool Vole\_Base::m\_Sex `[protected]` |

Their sex Male==true Female==false

Referenced by Vole\_Male::Init(), Vole\_Female::Init(), and SupplySex().

|  |
| --- |
| int Vole\_Base::m\_StarvationDays `[protected]` |

How many days they have been starving

Referenced by Vole\_Male::DecideQualityAction(), Vole\_Male::Dispersal(), Vole\_Female::Dispersal(), Vole\_Female::st\_Evaluate\_n\_Explore(), and Vole\_Base().

|  |
| --- |
| int Vole\_Base::m\_TerrRange `[protected]` |

The size of their territory (radius of a square)

Referenced by CalculateCarryingCapacity(), Vole\_Male::DetermineTerritorySize(), Vole\_Male::Dispersal(), Vole\_Female::Dispersal(), Vole\_Female::EndStep(), Vole\_Male::Init(), Vole\_Female::Init(), Vole\_Male::st\_Eval\_n\_Explore(), Vole\_Female::st\_Evaluate\_n\_Explore(), Vole\_Male::st\_Infanticide(), Vole\_Female::st\_Special\_Explore(), and Vole\_Base().

|  |
| --- |
| double Vole\_Base::m\_Weight `[protected]` |

Their weight in grams

Referenced by Vole\_Male::DetermineTerritorySize(), Vole\_Male::EndStep(), Vole\_Female::EndStep(), Vole\_Male::Init(), Vole\_Female::Init(), and SetWeight().

|  |
| --- |
| GeneticMaterial Vole\_Base::MyGenes `[protected]` |

Their genes

Referenced by CopyMyself(), GetDirectFlag(), GetGeneticFlag(), Vole\_Male::Init(), Vole\_Female::Init(), SetDirectFlag(), SetGeneticFlag(), Vole\_Female::st\_Lactating(), SupplyAllele(), SupplyGenes(), SupplyHeteroZyg(), SupplyHomoZyg(), UnsetDirectFlag(), UnsetGeneticFlag(), and Vole\_Base().

|  |
| --- |
| int Vole\_Base::SimH `[protected]` |

The size of simulation landscape

Referenced by CalculateCarryingCapacity(), DoWalkingCorrect(), Escape(), MoveTo(), and Vole\_Base().

|  |
| --- |
| int Vole\_Base::SimW `[protected]` |

Referenced by CalculateCarryingCapacity(), DoWalkingCorrect(), Escape(), MoveTo(), and Vole\_Base().

---

The documentation for this class was generated from the following files:

- vole\_all.h- Vole\_all.cpp

---

Generated on Thu Jan 22 14:13:47 2009 for ALMaSS ODDox by 
 1.5.6 
