# Supplementary material for: Predation and fragmentation portrayed in the statistical structure of prey time series
Source: BMC Ecol. 2009 May 6;9:10. doi: 10.1186/1472-6785-9-10 (PMC2689204; doi:10.1186/1472-6785-9-10)
Supplement: Additional file 2 — Voles and related classes ODDox Documentation. ODDox documentation of the agent-based model (ALMaSS) applied by Hendrichsen et al. The documentation is started by activating main.html. [file 1472-6785-9-10-S2.zip › Vole_ODDox/class_vole___female-members.html]

ALMaSS ODDox: Member List

- Main Page
- Related Pages
- Classes
- Files

- Alphabetical List
- Class List
- Class Hierarchy
- Class Members

# Vole\_Female Member List

This is the complete list of members for Vole\_Female, including all inherited members.

|  |  |  |
| --- | --- | --- |
| AssessHabitat(int polyref) | Vole\_Base | `[protected]` |
| BeginStep() | Vole\_Female | `[virtual]` |
| CalculateCarryingCapacity(int x, int y) | Vole\_Base | `[protected]` |
| CalculateCarryingCapacity(int x, int y, int &p\_stand\_x, int &p\_stand\_y) | Vole\_Base | `[protected]` |
| CheckManagement(void) | TAnimal |  |
| CheckManagementXY(int x, int y) | TAnimal |  |
| CopyMyself(int a\_vtype) | Vole\_Base |  |
| TAnimal::CopyMyself() | TAnimal | `[inline, virtual]` |
| CurrentStateNo | TALMaSSObject |  |
| CurrentVState | Vole\_Base |  |
| Dispersal(double p\_OldQual, int p\_Distance) | Vole\_Female | `[protected]` |
| DoWalking(int p\_Distance, int &p\_Vector, int &vx, int &vy) | Vole\_Base | `[protected]` |
| DoWalkingCorrect(int p\_Distance, int &p\_Vector, int &vx, int &vy) | Vole\_Base | `[protected]` |
| Dying() | TAnimal | `[inline, virtual]` |
| EndStep() | Vole\_Female | `[virtual]` |
| Escape(int p\_Vector, int p\_Distance) | Vole\_Base | `[protected]` |
| FreeLocation() | Vole\_Female | `[inline, protected, virtual]` |
| GetDirectFlag() | Vole\_Base | `[inline]` |
| GetGeneticFlag() | Vole\_Base | `[inline]` |
| GetLocation(int px, int py) | Vole\_Female | `[inline, protected, virtual]` |
| IDNo | Vole\_Base | `[protected]` |
| Init() | Vole\_Female | `[protected]` |
| KillThis() | TAnimal | `[inline, virtual]` |
| m\_Age | Vole\_Base | `[protected]` |
| m\_BornLastYear | Vole\_Female | `[protected]` |
| m\_DaysUntilBirth | Vole\_Female | `[protected]` |
| m\_DispVector | Vole\_Base | `[protected]` |
| m\_Have\_Territory | Vole\_Base | `[protected]` |
| m\_LifeSpan | Vole\_Base | `[protected]` |
| m\_Location\_x | TAnimal | `[protected]` |
| m\_Location\_y | TAnimal | `[protected]` |
| m\_Mature | Vole\_Base | `[protected]` |
| m\_NoOfYoung | Vole\_Female | `[protected]` |
| m\_OurLandscape | TAnimal | `[protected]` |
| m\_OurPopulation | Vole\_Base |  |
| m\_pesticide\_accumulation | Vole\_Female | `[protected]` |
| m\_pesticideInfluenced | Vole\_Base | `[protected]` |
| m\_pesticideInfluenced2 | Vole\_Base |  |
| m\_Pregnant | Vole\_Female | `[protected]` |
| m\_Reserves | Vole\_Base | `[protected]` |
| m\_Sex | Vole\_Base | `[protected]` |
| m\_StarvationDays | Vole\_Base | `[protected]` |
| m\_TerrRange | Vole\_Base | `[protected]` |
| m\_Weight | Vole\_Base | `[protected]` |
| m\_YoungAge | Vole\_Female | `[protected]` |
| MatesGenes | Vole\_Female | `[protected]` |
| MortalityTest() | Vole\_Base |  |
| MoveQuality(int p\_x, int p\_y) | Vole\_Base | `[protected]` |
| MoveTo(int p\_Vector, int p\_Distance, int iterations) | Vole\_Base | `[protected]` |
| MyGenes | Vole\_Base | `[protected]` |
| OnArrayBoundsError() | TALMaSSObject |  |
| OnFarmEvent(FarmToDo event) | Vole\_Female | `[virtual]` |
| OnInfanticideAttempt() | Vole\_Female |  |
| OnKilled() | Vole\_Female | `[virtual]` |
| Set\_Age(int Age) | Vole\_Base | `[inline]` |
| SetDirectFlag() | Vole\_Base | `[inline]` |
| SetGeneticFlag() | Vole\_Base | `[inline]` |
| SetLocation() | Vole\_Female | `[inline, protected, virtual]` |
| Setm\_Mature() | Vole\_Base | `[inline]` |
| SetWeight(double W) | Vole\_Base | `[inline]` |
| SetX(int a\_x) | TAnimal | `[inline]` |
| SetY(int a\_y) | TAnimal | `[inline]` |
| SimH | Vole\_Base | `[protected]` |
| SimW | Vole\_Base | `[protected]` |
| st\_BecomeReproductive() | Vole\_Female | `[protected]` |
| st\_Dying() | Vole\_Base |  |
| st\_Evaluate\_n\_Explore() | Vole\_Female | `[protected]` |
| st\_GiveBirth() | Vole\_Female | `[protected]` |
| st\_Lactating() | Vole\_Female | `[protected]` |
| st\_Mating() | Vole\_Female | `[protected]` |
| st\_ReproBehaviour() | Vole\_Female | `[protected]` |
| st\_Special\_Explore() | Vole\_Female | `[protected]` |
| st\_UpdateGestation() | Vole\_Female | `[protected]` |
| Step() | Vole\_Female | `[virtual]` |
| StepDone | TALMaSSObject |  |
| Supply\_m\_Location\_x() | TAnimal | `[inline]` |
| Supply\_m\_Location\_y() | TAnimal | `[inline]` |
| SupplyAge() | Vole\_Base | `[inline]` |
| SupplyAllele(int locus, int allele) | Vole\_Base | `[inline]` |
| SupplyFarmOwnerRef() | TAnimal |  |
| SupplyGenes() | Vole\_Base | `[inline]` |
| SupplyHeteroZyg() | Vole\_Base | `[inline]` |
| SupplyHomoZyg() | Vole\_Base | `[inline]` |
| SupplyNoOfYoung() | Vole\_Female | `[inline]` |
| SupplyPosition() | TAnimal |  |
| SupplySex() | Vole\_Base | `[inline]` |
| SupplyTerritorial() | Vole\_Base | `[inline]` |
| SupplyX() | Vole\_Base | `[inline]` |
| SupplyY() | Vole\_Base | `[inline]` |
| TALMaSSObject() | TALMaSSObject |  |
| TAnimal(int x, int y, Landscape \*L) | TAnimal |  |
| UnsetDirectFlag() | Vole\_Base | `[inline]` |
| UnsetGeneticFlag() | Vole\_Base | `[inline]` |
| Vole\_Base(int x, int y, Landscape \*L, GeneticMaterial DNA, Vole\_Population\_Manager \*VPM) | Vole\_Base |  |
| Vole\_Female(int p\_x, int p\_y, Landscape \*p\_L, GeneticMaterial DNA, Vole\_Population\_Manager \*p\_VPM) | Vole\_Female |  |
| WhatState() | Vole\_Base | `[inline, virtual]` |
| ~TALMaSSObject() | TALMaSSObject | `[virtual]` |
| ~Vole\_Base() | Vole\_Base |  |
| ~Vole\_Female() | Vole\_Female |  |

---

Generated on Thu Jan 22 14:13:47 2009 for ALMaSS ODDox by 
 1.5.6 
