# Supplementary material for: Predation and fragmentation portrayed in the statistical structure of prey time series
Source: BMC Ecol. 2009 May 6;9:10. doi: 10.1186/1472-6785-9-10 (PMC2689204; doi:10.1186/1472-6785-9-10)
Supplement: Additional file 2 — Voles and related classes ODDox Documentation. ODDox documentation of the agent-based model (ALMaSS) applied by Hendrichsen et al. The documentation is started by activating main.html. [file 1472-6785-9-10-S2.zip › Vole_ODDox/class_vole___female.html]

ALMaSS ODDox: Vole\_Female Class Reference

- Main Page
- Related Pages
- Classes
- Files

- Alphabetical List
- Class List
- Class Hierarchy
- Class Members

# Vole\_Female Class Reference

`#include <vole_all.h>`

Inheritance diagram for Vole\_Female:

List of all members.

---

## Detailed Description

The class for female voles.

Contains all the behaviour specific to the female vole. The differences between the male and female are primarily in female reproductive behaviour, but there are small differences in other behaviours requiring re-implementation of many of the behaviours (e.g. dispersal).

|  |
| --- |
|  |
| Public Member Functions | |
| virtual void | BeginStep () |
|  | Female vole BeginStep. |
| virtual void | EndStep () |
|  | Female vole EndStep. |
| virtual bool | OnFarmEvent (FarmToDo event) |
|  | External event handler. |
| void | OnInfanticideAttempt () |
|  | Determines whether an infanticide attempt will succeed. |
| virtual void | OnKilled () |
|  | Death from external entity. |
| virtual void | Step () |
|  | Female vole Step. |
| int | SupplyNoOfYoung () |
|  | Vole\_Female (int p\_x, int p\_y, Landscape \*p\_L, GeneticMaterial DNA, Vole\_Population\_Manager \*p\_VPM) |
|  | Vole\_Female constructor. |
|  | ~Vole\_Female () |
| Protected Member Functions | |
| void | Dispersal (double p\_OldQual, int p\_Distance) |
|  | Female dispersal. |
| virtual void | FreeLocation () |
|  | Location map function. |
| virtual bool | GetLocation (int px, int py) |
|  | Location map function. |
| void | Init () |
|  | Sets parameters to their correct default values. |
| virtual void | SetLocation () |
|  | Location map function. |
| int | st\_BecomeReproductive () |
|  | Female vole maturation control. |
| int | st\_Evaluate\_n\_Explore () |
|  | Main territory evaluation behaviour. |
| int | st\_GiveBirth () |
|  | Litter production. |
| int | st\_Lactating () |
|  | Lactation. |
| int | st\_Mating () |
|  | Female mating. |
| int | st\_ReproBehaviour () |
|  | Reproductive switch. |
| int | st\_Special\_Explore () |
|  | Post weaning territory expansion. |
| int | st\_UpdateGestation () |
|  | Gestation control. |
| Protected Attributes | |
| unsigned | m\_BornLastYear |
|  | A flag set if the female was born the year before. |
| int | m\_DaysUntilBirth |
|  | A counter counting down gestation days. |
| int | m\_NoOfYoung |
|  | The number of young in the current litter (if one). |
| double | m\_pesticide\_accumulation |
|  | The current body burden of a pesticide. |
| bool | m\_Pregnant |
|  | A flag indicating whether pregnant or not. |
| unsigned | m\_YoungAge |
|  | The age of current litter in days. |
| GeneticMaterial | MatesGenes |
|  | The DNA passed from the male on mating. |

---

## Constructor & Destructor Documentation

|  |  |  |  |
| --- | --- | --- | --- |
| Vole\_Female::Vole\_Female | ( | int | *p\_x*, |
|  |  | int | *p\_y*, |
|  |  | Landscape \* | *p\_L*, |
|  |  | GeneticMaterial | *DNA*, |
|  |  | Vole\_Population\_Manager \* | *p\_VPM* |  |
|  | ) |  |  |  |

Vole\_Female constructor.

- just calls Init()

References Init().

```
01471                                                :Vole_Base(p_x,p_y,p_L,DNA,p_VPM)
01472 {
01473   Init();
01474 }
```

|  |  |  |  |  |
| --- | --- | --- | --- | --- |
| Vole\_Female::~Vole\_Female | ( |  | ) |  |

```
01479 {
01480   // Nothing to do
01481 }
```

---

## Member Function Documentation

|  |  |  |  |  |  |
| --- | --- | --- | --- | --- | --- |
| void Vole\_Female::BeginStep | ( | void |  | ) | `[virtual]` |

Female vole BeginStep.

The BeginStep is one of the three timestep divisions. This is called once for each vole before Step and EndStep.   
The main function here is to remove voles that die before they take up CPU resources in the Step code.   
Can also be used to check for pesticide accumulation levels in pesticide simulation

Reimplemented from Vole\_Base.

References Vole\_Base::CurrentVState, g\_SpeedyDivides, l\_pest\_enable\_pesticide\_engine, TAnimal::m\_Location\_x, TAnimal::m\_Location\_y, TAnimal::m\_OurLandscape, m\_pesticide\_accumulation, Vole\_Base::MortalityTest(), TALMaSSObject::StepDone, and tovs\_FDying.

```
01523 {
01524   if (MortalityTest())
01525   {
01526     CurrentVState=tovs_FDying;
01527     StepDone=true;
01528   }
01529 #ifdef __SpecificPesticideEffectsVinclozolinLike__
01530   // Pesticide influence code - assuming we only check its initial position
01531   // No bioaccumulation
01532   // Impact is checked elsewhere
01533 #ifdef __PesticideThresholdEffectsONLY__ // This is used to set threshold values instead of means
01534                 // during gestation
01535   m_pesticide_accumulation=0;
01536 #endif
01537 /*
01538 Ingestion Multiplyer 1.39                                       
01539 Wt 0.025g                                       
01540 Ingested weight = 0.03475       kg              
01541 But we need this as dry weight, so multiply by 0.3 = 0.010425
01542                                         
01543 Hence the vole consumes 0.010425kg per day      
01544 */
01545   double pest=0;
01546   if ( l_pest_enable_pesticide_engine.value()) pest =m_OurLandscape->SupplyPesticide( m_Location_x,m_Location_y ); // Units are mg/m
01547   int veg= 1 + int (m_OurLandscape->SupplyVegBiomass( m_Location_x,m_Location_y ));  // Units are g dw/m, 1 added to avoid divide by zero
01548   // pest is is mg/m2 (corrected for wetweight)
01549   // veg is in g/m2
01550   // we need this in mg/kg body weight
01551   // mg/g veg = pest/veg
01552   // ingested mg (X) = pest/veg * 0.010425 * 1000
01553   // mg/kg = X/0.025  (vole weight in kg)
01554   pest*=g_SpeedyDivides[veg]; // assuming 70% is water
01555   pest*=10.425;
01556   pest*=40; // = 1/0.025
01557   m_pesticide_accumulation += pest;
01558 #endif
01559   }
```

|  |  |  |  |
| --- | --- | --- | --- |
| void Vole\_Female::Dispersal | ( | double | *p\_OldQual*, |
|  |  | int | *p\_Distance* |  |
|  | ) |  |  | `[protected]` |

Female dispersal.

Checks p\_Distance away to see if it can find a territory with a higher quality than p\_OldQual   
This entails some risk though, so there is a fixed 2.5% increase in the mortality chance when it does this.

References Vole\_Base::CalculateCarryingCapacity(), FreeLocation(), g\_rand\_uni, Vole\_Base::m\_Age, Vole\_Base::m\_DispVector, Vole\_Base::m\_Have\_Territory, TAnimal::m\_Location\_x, TAnimal::m\_Location\_y, Vole\_Base::m\_Mature, Vole\_Base::m\_OurPopulation, Vole\_Base::m\_StarvationDays, Vole\_Base::m\_TerrRange, MinFVoleHabQual, Vole\_Base::MoveTo(), and SetLocation().

Referenced by st\_Evaluate\_n\_Explore().

```
01403 {
01404     // Do a predation test
01405     if (!m_Have_Territory) {
01406       if (g_rand_uni() < g_extradispmort ) {  // 2.5% per day
01407       m_StarvationDays=1000; // This will effectively kill it
01408       return;
01409     }}
01410     // p_OldQuatells whether dispersal is conditional on quality or not
01411     // p_OldQual is set to old habitat quality or -1
01412     // p_Distance is the p_Distance used by the move function
01413 
01414     // aim is to move in a directed way traversing the landscape using the best
01415     // habitats
01416     int oldx = m_Location_x;
01417     int oldy = m_Location_y;
01418     double CC;
01419     if (m_DispVector == -1) m_DispVector = random(8); // Choose direction 0-7
01420     // Go that far in that direction (assuming it is possible to do that)
01421     MoveTo(m_DispVector, p_Distance,10);
01422     //  Now we are there so what is the new quality
01423     // 1. Get the density of voles in a RANGE m radius is p_Older
01424     bool Older=m_OurPopulation->
01425              SupplyOlderFemales(m_Location_x,m_Location_y,m_Age,m_TerrRange);
01426     // Get the carrying capacity
01427     if (Older)
01428     {
01429       CC = CalculateCarryingCapacity(m_Location_x,m_Location_y);
01430     }
01431     else CC=-1;
01432     // CC is the mean quality, which if >2 allows there to be 1 other older vole
01433     // 3. Now have the information to make a decision to move or not
01434     if (p_OldQual==-1) // non-conditional move
01435     {
01436       if (CC<MinFVoleHabQual)
01437       {
01438         // Can't establish Territory Here
01439         m_Have_Territory=false;
01440       }
01441       else
01442       {
01443         // Can establish territory
01444         if (m_Mature)
01445         {
01446           m_Have_Territory=true;
01447         }
01448       }
01449     }
01450     else    //is a conditional move so new quality must be better than old
01451     {
01452       if (CC <= p_OldQual)
01453       {
01454         // Reset the original home co-ordinates
01455         FreeLocation();
01456         m_Location_x=oldx;
01457         m_Location_y=oldy;
01458         SetLocation();
01459       }
01460     }
01461 }
```

|  |  |  |  |  |  |
| --- | --- | --- | --- | --- | --- |
| void Vole\_Female::EndStep | ( | void |  | ) | `[virtual]` |

Female vole EndStep.

The EndStep one of the three timestep divisions. This is called once for each vole after BeginStep and Step.   
The main function here is to remove voles that have died during step and otherwise to grow if not at max weight. It also checks if the vole was killed due to human management and determines the potential territory size.

Reimplemented from Vole\_Base.

References TAnimal::CheckManagement(), Vole\_Base::CurrentVState, FemaleTerritoryRange, GrowStopDate, growthperdayF, Vole\_Base::m\_Age, m\_BornLastYear, Vole\_Base::m\_Mature, TAnimal::m\_OurLandscape, Vole\_Base::m\_OurPopulation, Vole\_Base::m\_TerrRange, Vole\_Base::m\_Weight, MaxWeightF, MinReproWeightF, Vole\_Base::st\_Dying(), Vole\_Population\_Manager::SupplyGrowthStartDate(), and tovs\_FDying.

```
01675 {
01676   CheckManagement();
01677   if (CurrentVState==tovs_FDying) st_Dying();
01678   else
01679   {
01680     m_Age++;
01681     /*              FEMALE GROWTH NOTES
01682 
01683      Female vole grows until 20g.
01684      After that she will only grow if he has matured
01685      Growth continues up to 55g.
01686 
01687      Reprduction cannot occur below 20g or 20 days
01688 
01689      Growth only occurs between 1 March and 1st August
01690 
01691      taken from Hanson L, 1977, Oikos 29.
01692     */
01693     int today=m_OurLandscape->SupplyDayInYear();
01694     if ((today<GrowStopDate) && (today>
01695                                m_OurPopulation->SupplyGrowthStartDate()))
01696     {
01697        if (m_Weight<20)
01698        {
01699          m_Weight+=growthperdayF;
01700        }
01701        else if ((m_Mature==true) && (m_Weight<MaxWeightF))
01702        {
01703          m_Weight+=growthperdayF;
01704          m_TerrRange=FemaleTerritoryRange+(FemaleTerritoryRangeSlope*
01705                                                     ((int)m_Weight-MinReproWeightF));
01706        }
01707     }
01708     else  if (today==1) m_BornLastYear=1; // must be true if alive on 1st Jan.
01709   }
01710 }
```

|  |  |  |  |  |
| --- | --- | --- | --- | --- |
| void Vole\_Female::FreeLocation | ( |  | ) | `[inline, protected, virtual]` |

Location map function.

Reimplemented from Vole\_Base.

References TAnimal::m\_Location\_x, TAnimal::m\_Location\_y, Vole\_Base::m\_OurPopulation, and Vole\_Population\_Manager::VoleMap.

Referenced by Dispersal(), st\_Special\_Explore(), and Step().

```
02445                                       {
02446      m_OurPopulation->VoleMap->ClearMapValue(m_Location_x,m_Location_y);
02447     };
```

|  |  |  |  |
| --- | --- | --- | --- |
| bool Vole\_Female::GetLocation | ( | int | *px*, |
|  |  | int | *py* |  |
|  | ) |  |  | `[inline, protected, virtual]` |

Location map function.

Reimplemented from Vole\_Base.

References Vole\_Base::m\_OurPopulation, and Vole\_Population\_Manager::VoleMap.

```
02452                                                    {
02453      if( m_OurPopulation->VoleMap->GetMapValue(px,py)) return true;
02454          return false;
02455     };
```

|  |  |  |  |  |  |
| --- | --- | --- | --- | --- | --- |
| void Vole\_Female::Init | ( | void |  | ) | `[protected]` |

Sets parameters to their correct default values.

References FemaleTerritoryRange, Vole\_Base::m\_Age, m\_BornLastYear, m\_NoOfYoung, m\_pesticide\_accumulation, m\_Pregnant, Vole\_Base::m\_Sex, Vole\_Base::m\_TerrRange, Vole\_Base::m\_Weight, Vole\_Base::MyGenes, GeneticMaterial::ScoreHQThreshold(), and WeanedWeight.

Referenced by Vole\_Female().

```
01489 {
01490   m_Sex=false;
01491   m_NoOfYoung=0;
01492   m_Pregnant=false;
01493   m_TerrRange=FemaleTerritoryRange;
01494   m_BornLastYear=0;
01495   m_Weight=WeanedWeight;
01496   m_Age=14;
01497 #ifdef __SpecificPesticideEffectsVinclozolinLike__
01498   m_pesticide_accumulation=0;
01499 #endif
01500 #ifdef __PHENOTYPIC_LINK_HQUAL
01501   // 1. Score our genetic code
01502   //
01503   double MultiplicationFactor=MyGenes.ScoreHQThreshold();
01504   // 2. Initialise parameter values
01505   //
01506   FHQThresh1 = FHabQualThreshold1 + MultiplicationFactor;
01507   FHQThresh2 = FHabQualThreshold2;
01508   FHQThresh3 = FHabQualThreshold3;
01509 #endif
01510 }
```

|  |  |  |  |  |  |
| --- | --- | --- | --- | --- | --- |
| bool Vole\_Female::OnFarmEvent | ( | FarmToDo | *event* | ) | `[virtual]` |

External event handler.

This method evaluates external events and chooses a suitable response (in this case a probability of dying because other effects will be taken up by the evaluate and explore state.

Reimplemented from TAnimal.

References autumn\_harrow, autumn\_or\_spring\_plough, autumn\_plough, autumn\_roll, autumn\_sow, burn\_straw\_stubble, cattle\_out, Vole\_Base::CurrentVState, cut\_to\_hay, cut\_to\_silage, cut\_weeds, deep\_ploughing, fa\_ammoniumsulphate, fa\_greenmanure, fa\_manure, fa\_npk, fa\_pk, fa\_sludge, fa\_slurry, fp\_greenmanure, fp\_liquidNH3, fp\_manganesesulphate, fp\_manure, fp\_npk, fp\_npks, fp\_pk, fp\_sludge, fp\_slurry, fungicide\_treat, g\_rand\_uni, growth\_regulator, harvest, hay\_bailing, hay\_turning, herbicide\_treat, hilling\_up, insecticide\_treat, TAnimal::m\_OurLandscape, molluscicide, mow, pigs\_out, row\_cultivation, sleep\_all\_day, spring\_harrow, spring\_plough, spring\_roll, spring\_sow, straw\_chopping, strigling, strigling\_sow, stubble\_harrowing, swathing, tovs\_FDying, water, and winter\_plough.

```
02218 {
02219  switch(event)
02220  {
02221 case  sleep_all_day:
02222   break;
02223 case  autumn_plough:
02224   if (g_rand_uni()<VoleSoilCultivationMort)
02225      CurrentVState=tovs_FDying;
02226   break;
02227 case  autumn_harrow:
02228   if (g_rand_uni()<VoleSoilCultivationMort)
02229      CurrentVState=tovs_FDying;
02230   break;
02231 case  autumn_roll:
02232   if (g_rand_uni()<VoleSoilCultivationMort)
02233      CurrentVState=tovs_FDying;
02234   break;
02235 case  autumn_sow:
02236   if (g_rand_uni()<VoleSoilCultivationMort)
02237      CurrentVState=tovs_FDying;
02238   break;
02239 case  winter_plough:
02240   if (g_rand_uni()<VoleSoilCultivationMort)
02241      CurrentVState=tovs_FDying;
02242   break;
02243 case  deep_ploughing:
02244   if (g_rand_uni()<VoleSoilCultivationMort)
02245      CurrentVState=tovs_FDying;
02246   break;
02247 case  spring_plough:
02248   if (g_rand_uni()<VoleSoilCultivationMort)
02249      CurrentVState=tovs_FDying;
02250   break;
02251 case  spring_harrow:
02252   if (g_rand_uni()<VoleSoilCultivationMort)
02253      CurrentVState=tovs_FDying;
02254   break;
02255 case  spring_roll:
02256   if (g_rand_uni()<VoleSoilCultivationMort)
02257      CurrentVState=tovs_FDying;
02258   break;
02259 case  spring_sow:
02260   if (g_rand_uni()<VoleSoilCultivationMort)
02261      CurrentVState=tovs_FDying;
02262   break;
02263 case  fp_npks:
02264   break;
02265 case  fp_npk:
02266   break;
02267 case  fp_pk:
02268   break;
02269 case  fp_liquidNH3:
02270   break;
02271 case  fp_slurry:
02272   break;
02273 case  fp_manganesesulphate:
02274   break;
02275 case  fp_manure:
02276   break;
02277 case  fp_greenmanure:
02278   break;
02279 case  fp_sludge:
02280   break;
02281 case  fa_npk:
02282   break;
02283 case  fa_pk:
02284   break;
02285 case  fa_slurry:
02286   break;
02287 case  fa_ammoniumsulphate:
02288   break;
02289 case  fa_manure:
02290   break;
02291 case  fa_greenmanure:
02292   break;
02293 case  fa_sludge:
02294   break;
02295 case  herbicide_treat:
02296   break;
02297 case  growth_regulator:
02298   break;
02299 case  fungicide_treat:
02300   break;
02301 case  insecticide_treat:
02302   break;
02303 case  molluscicide:
02304   break;
02305 case  row_cultivation:
02306   if (g_rand_uni()<VoleSoilCultivationMort)
02307      CurrentVState=tovs_FDying;
02308   break;
02309 case  strigling:
02310   if (g_rand_uni()<VoleStriglingMort)
02311      CurrentVState=tovs_FDying;
02312   break;
02313 case  hilling_up:
02314   if (g_rand_uni()<VoleSoilCultivationMort)
02315      CurrentVState=tovs_FDying;
02316   break;
02317 case  water:
02318   break;
02319 case  swathing:
02320   if (g_rand_uni()<VoleHarvestMort)
02321      CurrentVState=tovs_FDying;
02322   break;
02323 case  harvest:
02324   if (g_rand_uni()<VoleHarvestMort)
02325      CurrentVState=tovs_FDying;
02326   break;
02327 case  cattle_out:
02328   break;
02329 case  cut_to_hay:
02330   if (g_rand_uni()<VoleHarvestMort)
02331      CurrentVState=tovs_FDying;
02332   break;
02333 case  cut_to_silage:
02334   if (g_rand_uni()<VoleHarvestMort)
02335      CurrentVState=tovs_FDying;
02336   break;
02337 case  straw_chopping:
02338   if (g_rand_uni()<VoleHarvestMort)
02339      CurrentVState=tovs_FDying;
02340   break;
02341 case  hay_turning:
02342   if (g_rand_uni()<VoleHarvestMort)
02343      CurrentVState=tovs_FDying;
02344   break;
02345 case  hay_bailing:
02346   if (g_rand_uni()<VoleHarvestMort)
02347      CurrentVState=tovs_FDying;
02348   break;
02349 case  stubble_harrowing:
02350   if (g_rand_uni()<VoleSoilCultivationMort)
02351      CurrentVState=tovs_FDying;
02352   break;
02353 case  autumn_or_spring_plough:
02354   if (g_rand_uni()<VoleSoilCultivationMort)
02355      CurrentVState=tovs_FDying;
02356   break;
02357 case  burn_straw_stubble:
02358   if (g_rand_uni()<VoleSoilCultivationMort)
02359      CurrentVState=tovs_FDying;
02360   break;
02361 case mow:
02362   if (g_rand_uni()<VoleHarvestMort)
02363      CurrentVState=tovs_FDying;
02364   break;
02365 case cut_weeds:
02366   if (g_rand_uni()<VoleHarvestMort)
02367      CurrentVState=tovs_FDying;
02368   break;
02369 case pigs_out:
02370   if (g_rand_uni()<VolePigGrazingMort)
02371      CurrentVState=tovs_FDying;
02372   break;
02373 case strigling_sow:
02374   if (g_rand_uni()<VoleSoilCultivationMort)
02375      CurrentVState=tovs_FDying;
02376   break;
02377 default:
02378   g_msg->Warn( WARN_FILE, "Vole_Female::OnFarmEvent(): Unknown event type:",
02379           m_OurLandscape->EventtypeToString(event) );
02380   exit( 1 );
02381  }
02382  if (CurrentVState==tovs_FDying)
02383  {
02384    return true;
02385  }
02386  else
02387  return false;
02388 }
```

|  |  |  |  |  |
| --- | --- | --- | --- | --- |
| void Vole\_Female::OnInfanticideAttempt | ( |  | ) |  |

Determines whether an infanticide attempt will succeed.

A mate has attempted infanticide.  
If they are 9 days old there is no chance of mortality otherwise it is proportional to age.   
Data from M.arvalis (Heise & Lippke 1997)

References g\_rand\_uni, m\_NoOfYoung, m\_Pregnant, and m\_YoungAge.

Referenced by Vole\_Population\_Manager::SendMessage().

```
02416 {
02417    //try this if she has kids
02418    if (m_NoOfYoung)
02419    {
02420      if (m_YoungAge<9)
02421      {
02422        double mortchance=g_rand_uni();
02423        if (mortchance<=InfanticideChanceByAge[m_YoungAge])
02424        {
02425 
02426          // Young are killed
02427          m_NoOfYoung=0;    // will cause the vole to be mated
02428          m_Pregnant=false;
02429       }
02430      }
02431    }
02432 }
```

|  |  |  |  |  |
| --- | --- | --- | --- | --- |
| void Vole\_Female::OnKilled | ( |  | ) | `[virtual]` |

Death from external entity.

External event has caused death - probably eaten by a explicitly modelled predator

Reimplemented from Vole\_Base.

References Vole\_Base::CurrentVState, and tovs\_FDying.

Referenced by Vole\_Population\_Manager::DoFirst().

```
02400 {
02401   CurrentVState=tovs_FDying;
02402 }
```

|  |  |  |  |  |
| --- | --- | --- | --- | --- |
| void Vole\_Female::SetLocation | ( |  | ) | `[inline, protected, virtual]` |

Location map function.

Reimplemented from Vole\_Base.

References TAnimal::m\_Location\_x, TAnimal::m\_Location\_y, Vole\_Base::m\_OurPopulation, and Vole\_Population\_Manager::VoleMap.

Referenced by Dispersal(), and st\_Special\_Explore().

```
02438                                      {
02439      m_OurPopulation->VoleMap->SetMapValue(m_Location_x,m_Location_y,this);
02440     };
```

|  |  |  |  |  |
| --- | --- | --- | --- | --- |
| int Vole\_Female::st\_BecomeReproductive | ( |  | ) | `[protected]` |

Female vole maturation control.

Tests to see if the female should mature

References FemNoMature, g\_rand\_uni, Vole\_Base::m\_Age, Vole\_Base::m\_Mature, TAnimal::m\_OurLandscape, Vole\_Base::m\_OurPopulation, MaturationChance, and Vole\_Population\_Manager::SupplyGrowthStartDate().

Referenced by Step().

```
01764 {
01765     if ((m_OurLandscape->SupplyDayInYear()<FemNoMature) &&
01766               (m_OurLandscape->SupplyDayInYear()
01767                                   > m_OurPopulation->SupplyGrowthStartDate()))
01768    {
01769       int month = m_Age / 30;
01770       if (month > 5) month = 5;
01771       if (MaturationChance[month] > g_rand_uni()) m_Mature=true;
01772       return 1;
01773    }
01774    return 0;
01775 }
```

|  |  |  |  |  |
| --- | --- | --- | --- | --- |
| int Vole\_Female::st\_Evaluate\_n\_Explore | ( |  | ) | `[protected]` |

Main territory evaluation behaviour.

Evaluates the quality of her habitat and does some limited exploration in the surrounding area to see if she can improve it by moving.

References Vole\_Base::CalculateCarryingCapacity(), Dispersal(), Vole\_Base::m\_DispVector, Vole\_Base::m\_Have\_Territory, TAnimal::m\_Location\_x, TAnimal::m\_Location\_y, Vole\_Base::m\_Mature, TAnimal::m\_OurLandscape, Vole\_Base::m\_OurPopulation, Vole\_Base::m\_Reserves, Vole\_Base::m\_StarvationDays, Vole\_Base::m\_TerrRange, MinFVoleHabQual, Vole\_Population\_Manager::SupplyGrowthStartDate(), and Vole\_Population\_Manager::SupplyHowManyVoles().

Referenced by Step().

```
02012 {
02013   double Quality_1,Quality_2;
02014   int today=m_OurLandscape->SupplyDayInYear();
02015   Quality_1 = CalculateCarryingCapacity(m_Location_x, m_Location_y);
02016   int Voles;
02017   if ((today>=m_OurPopulation->SupplyGrowthStartDate())
02018                                &&(today<=MaleReproductFinish)&&(m_Mature)) {
02019       Voles=m_OurPopulation->SupplyHowManyVoles(m_Location_x,m_Location_y, m_TerrRange);
02020   } else Voles=1;
02021   Quality_2=Quality_1*FemaleResourceReq[m_OurLandscape->SupplyMonth()];
02022     if (Voles<1) Voles=1;
02023   Quality_2/=(double)Voles;
02024   // No social reason not be be here so is it good enough quality
02025   if (Quality_2 > FHabQualThreshold3)
02026   {
02027     // Very Good
02028     m_Reserves++;
02029     m_StarvationDays = 0;
02030     if (m_Mature)
02031     {
02032       m_Have_Territory=true;
02033     }
02034   }
02035    else if (Quality_2>FHabQualThreshold2)
02036    {
02037      // Average quality
02038      m_Reserves++;
02039      m_StarvationDays=0;
02040      // check an area MinFemaleMovement to FemaleMovement metres away
02041      Dispersal(Quality_2,random(FemaleMovement)+MinFemaleMovement);
02042      if (m_Mature)
02043      {
02044        m_Have_Territory=true;
02045      }
02046    }
02047     else if (Quality_2 >=FHabQualThreshold1) // Not Good
02048     {
02049       // v.bad but not forced out so will do an explore
02050       if (Quality_2>MinFVoleHabQual) m_StarvationDays = 0; else m_Reserves--;
02051       m_DispVector = random(8); // choose a direction
02052       // check an area FemaleMovement metres away, unless we have not
02053       // established a territory then we must look elsewhere
02054       if (m_Have_Territory) Dispersal(Quality_2,random(FemaleMovement)+MinFemaleMovement);
02055       else
02056       {
02057         Dispersal(-1,FemaleMovement);
02058       }
02059     }
02060      else  // Forced out, less than minimum
02061      {
02062        if (Quality_2<MinFVoleHabQual) m_Reserves--;
02063        if (m_Have_Territory == true)
02064        {
02065          m_Have_Territory = false;
02066          m_DispVector = random(8);
02067          // Not quality dependent dispersal, very directed
02068          Dispersal(-1,FemaleMovement);
02069        }
02070        else
02071        {
02072          // Don't change the dispersal vector because she is already dispersing
02073          Dispersal(-1,FemaleMovement);
02074        }
02075      }
02076   // if reserves drop below zero then start accumulating starvation days
02077   // max reserves are 3
02078   if (m_Reserves == -1)
02079   {
02080      m_Reserves = 0;
02081      m_StarvationDays++;
02082   }
02083   else if (m_Reserves > 3) m_Reserves = 3;
02084 
02085   if (m_StarvationDays>MaxStarvationDays) return 1; // Die of starvation
02086   else return 0;
02087 }
```

|  |  |  |  |  |
| --- | --- | --- | --- | --- |
| int Vole\_Female::st\_GiveBirth | ( |  | ) | `[protected]` |

Litter production.

Produces a litter and records the information if necessary

References Vole\_Population\_Manager::AddToYoung(), Population\_Manager::LamdaBirth(), m\_BornLastYear, TAnimal::m\_Location\_x, TAnimal::m\_Location\_y, m\_NoOfYoung, TAnimal::m\_OurLandscape, Vole\_Base::m\_OurPopulation, m\_Pregnant, Vole\_Base::m\_Reserves, m\_YoungAge, and Vole\_Population\_Manager::ReproTable.

Referenced by Step().

```
01786 {
01787    // TODO must locate the nest position if this is considered important - currently it is set as the centre of the territory
01788    m_Pregnant = false;
01789    if (m_Reserves == 3)
01790    {
01791 #ifndef  __PHENOTYPIC_LINK_REPRO
01792       // Must produce young
01793       int chance=m_OurPopulation->ReproTable[m_BornLastYear+2]
01794                                          [m_OurLandscape->SupplyDayInYear()/30];
01795       m_NoOfYoung= m_OurPopulation->ReproTable[m_BornLastYear]
01796                                          [m_OurLandscape->SupplyDayInYear()/30];
01797       if (random(100)<chance)
01798       {
01799         m_NoOfYoung+=1;
01800 
01801       }
01802 #else
01803       // Must produce young
01804       int chance=MyReproTable[m_BornLastYear+2]
01805                                          [m_OurLandscape->SupplyDayInYear()/30];
01806       m_NoOfYoung= m_OurPopulation->ReproTable[m_BornLastYear]
01807                                          [m_OurLandscape->SupplyDayInYear()/30];
01808       if (random(100)<chance)
01809       {
01810         m_NoOfYoung+=1;
01811       }
01812 #endif
01813 #ifdef __VOLE_LOWREPRO
01814   m_NoOfYoung=1;
01815 #endif
01816       // Must age young
01817       m_YoungAge = 0;
01818       // Update the YoungProduced Today Counter
01819       m_OurPopulation->AddToYoung(m_NoOfYoung);
01820 #ifdef __LAMBDA_RECORD
01821           m_OurPopulation->LamdaBirth(m_Location_x,m_Location_y,m_NoOfYoung);
01822 #endif
01823           return 0;
01824    }
01825    else return 1;
01826 }
```

|  |  |  |  |  |
| --- | --- | --- | --- | --- |
| int Vole\_Female::st\_Lactating | ( |  | ) | `[protected]` |

Lactation.

Once the litter reaches weaning age then individual voles are created and set in motion by this method. Pesticide effects may be specified here too.

References Vole\_Population\_Manager::AddToGeneticImpacted(), Vole\_Population\_Manager::AddToImpacted(), Vole\_Population\_Manager::AddToJuvs(), Vole\_Population\_Manager::AddToNotImpacted(), Vole\_Population\_Manager::CreateObjects(), struct\_Vole\_Adult::Genes, struct\_Vole\_Adult::L, struct\_Vole\_Adult::m\_dflag, struct\_Vole\_Adult::m\_flag, struct\_Vole\_Adult::m\_gflag, TAnimal::m\_Location\_x, TAnimal::m\_Location\_y, m\_NoOfYoung, TAnimal::m\_OurLandscape, Vole\_Base::m\_OurPopulation, m\_pesticide\_accumulation, Vole\_Base::m\_pesticideInfluenced, Vole\_Base::m\_pesticideInfluenced2, Vole\_Base::m\_Reserves, m\_YoungAge, MatesGenes, GeneticMaterial::Mutation\_1(), Vole\_Base::MyGenes, GeneticMaterial::Recombine(), Population\_Manager::SimH, Population\_Manager::SimW, struct\_Vole\_Adult::VPM, WeanedAge, struct\_Vole\_Adult::x, and struct\_Vole\_Adult::y.

Referenced by Step().

```
01836 {
01837     if(m_YoungAge++ >= WeanedAge)
01838     {
01839       struct_Vole_Adult* av;
01840       av = new struct_Vole_Adult;
01841       av->VPM = m_OurPopulation;
01842       av->L = m_OurLandscape;
01843       av->m_flag=true; // Used to signal pesticide effect to CreateObjects
01844 
01845       // Create the new voles (50% chance of male/female)
01846       for (int i=0; i<m_NoOfYoung; i++)
01847       {
01848         av->x = ((m_Location_x+random(10))%m_OurPopulation->SimW);
01849         av->y = ((m_Location_y+random(10))%m_OurPopulation->SimH);
01850         // Do the genetics
01851         av->Genes.Recombine(&MyGenes,&MatesGenes);
01852 #ifdef __Mutation
01853         else av->Genes.Mutation_1(); // This is only one of a number of mutation types we can use
01854 #endif
01855         if (random(2)==1) // Even sex ratio
01856         {
01857            // Males
01858 #ifdef __SpecificPesticideEffectsVinclozolinLike__
01859                 // If her mate was pesticide influenced then ensure all male offspring are also
01860                 // by setting the gene on
01861                         if ((m_pesticideInfluenced)||(m_pesticideInfluenced2)) {
01862                            // Any kind of influence set 0 chromo to 1
01863                            av->m_gflag=true;
01864                            if (m_pesticideInfluenced) {
01865                                         av->m_dflag=true;
01866                                         m_OurPopulation->AddToImpacted();
01867                            }
01868                            else {
01869                                         av->m_dflag=false;
01870                                         m_OurPopulation->AddToNotImpacted();
01871                                         m_OurPopulation->AddToGeneticImpacted();
01872                            }
01873 
01874                 } else {
01875                         av->m_gflag=false;
01876                         av->m_dflag=false;
01877                         m_OurPopulation->AddToNotImpacted();
01878                 }
01879 #endif
01880           m_OurPopulation->CreateObjects(0,this,av,1);
01881                 }
01882                 else {
01883 #ifdef __SpecificPesticideEffectsVinclozolinLike__
01884                         // The Vinclozolin effect is transmitted through the male line only, 
01885                         // so remove the effects if it is a female.
01886     av->m_gflag=false;
01887     av->m_dflag=false;
01888 #endif
01889                         m_OurPopulation->CreateObjects(1,this,av,1);
01890                 }
01891       }
01892       m_OurPopulation->AddToJuvs(m_NoOfYoung);
01893       m_Reserves=0; // all reserves used up
01894       m_NoOfYoung=0; // No more young to feed
01895       delete av; // clean up
01896 #ifdef __SpecificPesticideEffectsVinclozolinLike__
01897       m_pesticide_accumulation=0;
01898 #endif
01899       return 1; //  CurrentVState=14 Special Explore
01900     }
01901     else return 0; // carry on feeding young
01902 }
```

|  |  |  |  |  |
| --- | --- | --- | --- | --- |
| int Vole\_Female::st\_Mating | ( |  | ) | `[protected]` |

Female mating.

The mating state is where genes are passed and any genetic effects from the male need to be evaluated (e.g. pesticide induced infertility)

References Vole\_Population\_Manager::FindRandomMale(), Vole\_Male::GetFertile(), m\_DaysUntilBirth, Vole\_Population\_Manager::m\_geneticproductfertilityeffect, TAnimal::m\_Location\_x, TAnimal::m\_Location\_y, Vole\_Base::m\_Mature, TAnimal::m\_OurLandscape, Vole\_Base::m\_OurPopulation, m\_pesticide\_accumulation, Vole\_Base::m\_pesticideInfluenced, Vole\_Base::m\_pesticideInfluenced2, m\_Pregnant, MatesGenes, Vole\_Base::SupplyGenes(), Vole\_Population\_Manager::SupplyGrowthStartDate(), and TheGestationPeriod.

Referenced by Step().

```
02166 {
02167   m_pesticideInfluenced=false; // Got to reset this flag before gestation, so this is a good place
02168   //if it is the breeding season
02169   if ((m_OurLandscape->SupplyDayInYear()<274)
02170       && (m_OurLandscape->SupplyDayInYear() >
02171                                     m_OurPopulation->SupplyGrowthStartDate()))
02172   {
02173     // Find a male if there is one
02174 #ifdef __VOLE_PANMICTIC
02175     Vole_Male* Mate=m_OurPopulation->FindRandomMale();
02176 #else
02177     Vole_Male* Mate=m_OurPopulation->
02178                                  FindClosestMale(m_Location_x, m_Location_y,20);
02179 #endif    // Get his genes if we found a mate
02180     if (Mate)
02181     {
02182 #ifdef __SpecificPesticideEffectsVinclozolinLike__
02183       // If __SpecificPesticideEffectsVinclozolinLike__ then the vole is possibly infertile
02184                 m_pesticideInfluenced2=false;
02185                 int fertile = Mate->GetFertile();
02186                 if (fertile==-1) {
02187                         return 4; // No breeding - the return value is currently not used, but could be used to make the female wait before mating again
02188                         // otherwise breed but this will pass on the infertility gene
02189                 }
02190                 if (fertile==0) {
02191                         m_pesticideInfluenced2=true;
02192                         if (random(100)<m_OurPopulation->m_geneticproductfertilityeffect) return 4; // Total infertility needs 0 here, 100 is 100% fertility
02193                 }
02194 #endif
02195       MatesGenes=Mate->SupplyGenes();
02196       // Save these for when the young need to get their Genes
02197       m_Pregnant = true;
02198       m_DaysUntilBirth = TheGestationPeriod;
02199 #ifdef __SpecificPesticideEffectsVinclozolinLike__
02200       // Reset the pesticide accumulator
02201       m_pesticide_accumulation=0;
02202 #endif
02203     }
02204   }
02205   // otherwise stop reproduction
02206   else m_Mature=false;
02207   return 0;
02208 }
```

|  |  |  |  |  |
| --- | --- | --- | --- | --- |
| int Vole\_Female::st\_ReproBehaviour | ( |  | ) | `[protected]` |

Reproductive switch.

This state is simply a switch determing what behaviour to exhibit

References Vole\_Base::m\_Mature, m\_NoOfYoung, and m\_Pregnant.

Referenced by Step().

```
01722 {
01723    if (!m_Mature) return 0; // Go to BecomeReproductive;
01724      else if (m_NoOfYoung>0) return 3;  //Go to lactating
01725        else if (!m_Pregnant) return 1; // Go to Mating;
01726          else return 2; // Go to UpdateGestation;
01727 }
```

|  |  |  |  |  |
| --- | --- | --- | --- | --- |
| int Vole\_Female::st\_Special\_Explore | ( |  | ) | `[protected]` |

Post weaning territory expansion.

Called after weaning of a litter when the female needs to expand her territory again. This does not use the Dispersal function so avoids extra mortality at this point.

References Vole\_Base::CalculateCarryingCapacity(), FreeLocation(), Vole\_Base::m\_Age, Vole\_Base::m\_Have\_Territory, TAnimal::m\_Location\_x, TAnimal::m\_Location\_y, Vole\_Base::m\_Mature, Vole\_Base::m\_OurPopulation, Vole\_Base::m\_TerrRange, Vole\_Base::MoveTo(), SetLocation(), and Vole\_Population\_Manager::SupplyOlderFemales().

Referenced by Step().

```
02098 {
02099   //The vole must now find a good territory
02100   // She will explore in all 8 directions from the nest and pick the best
02101   // from these and here present position
02102   unsigned coords[9][2];
02103   double qualities[9];
02104   // Check the quality here
02105   coords[8][0]=m_Location_x;
02106   coords[8][1]=m_Location_y;
02107   if (!m_OurPopulation->SupplyOlderFemales(m_Location_x,m_Location_y,m_Age,m_TerrRange))
02108      qualities[8]=-1;
02109   else
02110   {
02111     qualities[8]=CalculateCarryingCapacity(m_Location_x,m_Location_y);
02112   }
02113   // Now check the quality in all 8 directions
02114   unsigned steps = 2 * FemaleMovement+MinFemaleMovement;
02115   for (unsigned i=0; i<8; i++)
02116   {
02117     MoveTo(i,steps,10); // changes m_Location_x & y
02118     coords[i][0]=m_Location_x;
02119     coords[i][1]=m_Location_y;
02120     if (!m_OurPopulation->SupplyOlderFemales(m_Location_x,m_Location_y,m_Age,
02121                                                                    m_TerrRange))
02122        qualities[i]=-1;
02123     else
02124     {
02125       qualities[i]=CalculateCarryingCapacity(m_Location_x,m_Location_y);
02126     }
02127   }
02128   // find the best out of the nine qualities
02129   double best =qualities[8]; // here
02130   unsigned found=8; // default stay here
02131   for (unsigned i=0; i<8; i++)
02132   {
02133     if (qualities[i]>best)
02134     {
02135       best=qualities[i];
02136       found=i;
02137     }
02138     if (best>1)
02139     {
02140       if (m_Mature)
02141       {
02142         m_Have_Territory=true;
02143       }
02144     }
02145     else
02146     {
02147       m_Have_Territory=false;
02148     }
02149     FreeLocation();
02150     m_Location_x=coords[found][0];
02151     m_Location_y=coords[found][1];
02152     SetLocation();
02153   }
02154   return 0;
02155 }
```

|  |  |  |  |  |
| --- | --- | --- | --- | --- |
| int Vole\_Female::st\_UpdateGestation | ( |  | ) | `[protected]` |

Gestation control.

Decreases the number of days until birth by 1, checks for special pesticide effects for vinclozolin like pesticide simulations

References cfg\_PesticideAccumulationThreshold(), cfg\_productapplicendyear, cfg\_productapplicstartyear, m\_DaysUntilBirth, TAnimal::m\_OurLandscape, m\_pesticide\_accumulation, and Vole\_Base::m\_pesticideInfluenced.

Referenced by Step().

```
01739 {
01740     m_DaysUntilBirth--;
01741  #ifdef __SpecificPesticideEffectsVinclozolinLike__
01742         // May also wish to specify certain gestation days for the effects here
01743         if ((m_DaysUntilBirth>0) && (m_DaysUntilBirth<5)) {
01744                 if (m_pesticide_accumulation>cfg_PesticideAccumulationThreshold.value()) {
01745                         int yr=m_OurLandscape->SupplyYearNumber();
01746                         if ((cfg_productapplicstartyear.value() <= yr) &&(cfg_productapplicendyear.value()>=yr)) {
01747                                 m_pesticideInfluenced=true;
01748                         }
01749                 }
01750         }
01751  #endif
01752     if (m_DaysUntilBirth == 0) return 1; else return 0;
01753 }
```

|  |  |  |  |  |  |
| --- | --- | --- | --- | --- | --- |
| void Vole\_Female::Step | ( | void |  | ) | `[virtual]` |

Female vole Step.

The Step is one of the three timestep divisions. This is called repeatedly after BeginStep and before EndStep, until all voles report that they are done with Step.   
  
Most of the behaviours are controlled by moving voles between behavioural states in Step (for other models this is also done in BeginStep and EndStep).   
When a vole is done for the day it will signal this by setting StepDone==true. NB that a call to one behaviour may trigger a call to another behaviour on the next call to step inside the same timestep. In this way a daily cycle of activity can be undertaken (i.e. do reproduction and explore)

Reimplemented from Vole\_Base.

References Vole\_Base::CurrentVState, FreeLocation(), TAnimal::m\_OurLandscape, st\_BecomeReproductive(), st\_Evaluate\_n\_Explore(), st\_GiveBirth(), st\_Lactating(), st\_Mating(), st\_ReproBehaviour(), st\_Special\_Explore(), st\_UpdateGestation(), TALMaSSObject::StepDone, tovs\_FDying, tovs\_FEvaluateExplore, tovs\_FMaturation, tovs\_GiveBirth, tovs\_Lactating, tovs\_Mating, tovs\_ReproBehaviour, tovs\_SpecialExplore, and tovs\_UpdateGestation.

```
01572 {
01573   if (StepDone) return;
01574   switch (CurrentVState)
01575   {
01576    case 0: // Initial state
01577     CurrentVState=tovs_FEvaluateExplore;
01578     break;
01579    case tovs_FEvaluateExplore: // Evaluate & Explore
01580     switch(st_Evaluate_n_Explore())
01581     {
01582       case 1:
01583        CurrentVState=tovs_FDying; // Die
01584        break;
01585       default:
01586        CurrentVState=tovs_ReproBehaviour; // repro behaviour
01587     }
01588     StepDone=true;
01589     break;
01590    case tovs_ReproBehaviour: // ReproBehaviour
01591     switch (st_ReproBehaviour())
01592     {
01593      case 0:
01594       CurrentVState=tovs_FMaturation; // Maturation?
01595       break;
01596      case 1:
01597       CurrentVState=tovs_Mating; // Mating
01598       break;
01599      case 2:
01600       CurrentVState=tovs_UpdateGestation; // UpdateGestation
01601       break;
01602      case 3:
01603       CurrentVState=tovs_Lactating; // Lactating
01604       break;
01605      default:
01606        m_OurLandscape->Warn("Vole_Female::Step - unknown return error",NULL);
01607     }
01608     break;
01609    case tovs_Lactating: // Lactating
01610     switch(st_Lactating())
01611     {
01612      case 1:
01613       CurrentVState=tovs_SpecialExplore; // Special Explore
01614       break;
01615      default:
01616       break;
01617     }
01618     StepDone=true;
01619     break;
01620    case tovs_GiveBirth:
01621     switch (st_GiveBirth())
01622     {
01623      case 0: // success
01624       CurrentVState=tovs_ReproBehaviour; // Go Back to reproBehaviour
01625       StepDone=true; // wait until next day
01626       break;
01627      case 1: // failure
01628       CurrentVState=tovs_FEvaluateExplore;
01629       StepDone=true;  // wait until next day
01630     }
01631     break;
01632    case tovs_FMaturation: // Maturation
01633     st_BecomeReproductive();
01634     CurrentVState=tovs_FEvaluateExplore; // Eval n Explore
01635     break;
01636    case tovs_Mating: // Mating
01637     st_Mating();
01638     CurrentVState=tovs_FEvaluateExplore; // Eval n Explore
01639     break;
01640    case tovs_UpdateGestation: // Update Gestation
01641     switch (st_UpdateGestation())
01642     {
01643      case 0:
01644       CurrentVState=tovs_FEvaluateExplore; // Eval & Explore
01645       break;
01646      case 1:
01647       CurrentVState=tovs_GiveBirth; // Give Birth
01648     }
01649     break;
01650    case tovs_SpecialExplore: // Special Explore
01651     st_Special_Explore();
01652     CurrentVState=tovs_ReproBehaviour;  // Repro behaviour
01653     StepDone=true;
01654     break;
01655    case tovs_FDying: // Die
01656     FreeLocation();
01657     StepDone=true;
01658     break;
01659    default:
01660        m_OurLandscape->Warn("Vole_Female::Step - unknown state",NULL);
01661   }
01662 }
```

|  |  |  |  |  |
| --- | --- | --- | --- | --- |
| int Vole\_Female::SupplyNoOfYoung | ( |  | ) | `[inline]` |

References m\_NoOfYoung.

```
00273 {return m_NoOfYoung;};
```

---

## Member Data Documentation

|  |
| --- |
| unsigned Vole\_Female::m\_BornLastYear `[protected]` |

A flag set if the female was born the year before.

Referenced by EndStep(), Init(), and st\_GiveBirth().

|  |
| --- |
| int Vole\_Female::m\_DaysUntilBirth `[protected]` |

A counter counting down gestation days.

Referenced by st\_Mating(), and st\_UpdateGestation().

|  |
| --- |
| int Vole\_Female::m\_NoOfYoung `[protected]` |

The number of young in the current litter (if one).

Referenced by Init(), OnInfanticideAttempt(), st\_GiveBirth(), st\_Lactating(), st\_ReproBehaviour(), and SupplyNoOfYoung().

|  |
| --- |
| double Vole\_Female::m\_pesticide\_accumulation `[protected]` |

The current body burden of a pesticide.

Referenced by BeginStep(), Init(), st\_Lactating(), st\_Mating(), and st\_UpdateGestation().

|  |
| --- |
| bool Vole\_Female::m\_Pregnant `[protected]` |

A flag indicating whether pregnant or not.

Referenced by Init(), OnInfanticideAttempt(), st\_GiveBirth(), st\_Mating(), and st\_ReproBehaviour().

|  |
| --- |
| unsigned Vole\_Female::m\_YoungAge `[protected]` |

The age of current litter in days.

Referenced by OnInfanticideAttempt(), st\_GiveBirth(), and st\_Lactating().

|  |
| --- |
| GeneticMaterial Vole\_Female::MatesGenes `[protected]` |

The DNA passed from the male on mating.

Referenced by st\_Lactating(), and st\_Mating().

---

The documentation for this class was generated from the following files:

- vole\_all.h- Vole\_all.cpp

---

Generated on Thu Jan 22 14:13:47 2009 for ALMaSS ODDox by 
 1.5.6 
