# Supplementary material for: Predation and fragmentation portrayed in the statistical structure of prey time series
Source: BMC Ecol. 2009 May 6;9:10. doi: 10.1186/1472-6785-9-10 (PMC2689204; doi:10.1186/1472-6785-9-10)
Supplement: Additional file 2 — Voles and related classes ODDox Documentation. ODDox documentation of the agent-based model (ALMaSS) applied by Hendrichsen et al. The documentation is started by activating main.html. [file 1472-6785-9-10-S2.zip › Vole_ODDox/class_vole___male.html]

ALMaSS ODDox: Vole\_Male Class Reference

- Main Page
- Related Pages
- Classes
- Files

- Alphabetical List
- Class List
- Class Hierarchy
- Class Members

# Vole\_Male Class Reference

`#include <vole_all.h>`

Inheritance diagram for Vole\_Male:

List of all members.

---

## Detailed Description

The class for male voles.

Contains all the behaviour specific to the male vole. Only st\_Infanticide and st\_JuvenileExplore are specific to the male, other behaviours differ only in details from the female.

|  |
| --- |
|  |
| Public Member Functions | |
| virtual void | BeginStep () |
|  | Male vole BeginStep. |
| virtual void | EndStep () |
|  | Male vole EndStep. |
| int | GetFertile () |
|  | Get the male vole fertility. |
| virtual bool | OnFarmEvent (FarmToDo event) |
|  | Male vole exernal event handler. |
| virtual void | OnKilled () |
|  | Male vole death by external entity. |
| void | SetFertile (int f) |
|  | Set the male vole fertility. |
| virtual void | Step () |
|  | Male vole Step. |
|  | Vole\_Male (int x, int y, Landscape \*L, GeneticMaterial DNA, Vole\_Population\_Manager \*VPM) |
|  | Vole\_Male constructor. |
|  | ~Vole\_Male () |
| Protected Member Functions | |
| bool | CanFeed () |
|  | Currently not used. |
| int | DecideQualityAction (double Qual) |
|  | Male vole decision making behaviour. |
| void | DetermineTerritorySize () |
|  | Calculates the territory size needed for a vole of his weight. |
| int | Dispersal (double p\_OldQual, int p\_Distance) |
|  | Male vole dispersal behaviour. |
| virtual void | FreeLocation () |
|  | Map location function. |
| virtual bool | GetLocation (int px, int py) |
|  | Map location function. |
| void | Init () |
|  | Sets default values. |
| virtual void | SetLocation () |
|  | Map location function. |
| int | st\_Eval\_n\_Explore (void) |
|  | Male vole main territory assessment behaviour. |
| void | st\_Infanticide (void) |
|  | Male vole infanticide behaviour. |
| void | st\_JuvenileExplore (void) |
|  | Extra movement on weaning. |
| int | st\_Maturation (void) |
|  | Male vole maturation control. |
| Protected Attributes | |
| int | m\_fertile |
|  | Flag indicating the fertility state (0=fertile). |

---

## Constructor & Destructor Documentation

|  |  |  |  |
| --- | --- | --- | --- |
| Vole\_Male::Vole\_Male | ( | int | *x*, |
|  |  | int | *y*, |
|  |  | Landscape \* | *L*, |
|  |  | GeneticMaterial | *DNA*, |
|  |  | Vole\_Population\_Manager \* | *VPM* |  |
|  | ) |  |  |  |

Vole\_Male constructor.

- just calls Init()

References Init().

```
02471                                                 :Vole_Base(x,y,L,DNA,VPM)
02472 {
02473     Init();
02474 }
```

|  |  |  |  |  |
| --- | --- | --- | --- | --- |
| Vole\_Male::~Vole\_Male | ( |  | ) |  |

```
02479 {
02480   // Nothing to do
02481 }
```

---

## Member Function Documentation

|  |  |  |  |  |  |
| --- | --- | --- | --- | --- | --- |
| void Vole\_Male::BeginStep | ( | void |  | ) | `[virtual]` |

Male vole BeginStep.

The BeginStep is one of the three timestep divisions. This is called once for each vole before Step and EndStep.   
The main function here is to remove voles that die before they take up CPU resources in the Step code.

Reimplemented from Vole\_Base.

References Vole\_Base::CurrentVState, Vole\_Base::MortalityTest(), TALMaSSObject::StepDone, and tovs\_MDying.

```
02520 {
02521   if (MortalityTest())
02522   {
02523     CurrentVState=tovs_MDying;
02524     StepDone=true;
02525   }
02526 }
```

|  |  |  |  |  |
| --- | --- | --- | --- | --- |
| bool Vole\_Male::CanFeed | ( |  | ) | `[inline, protected]` |

Currently not used.

Check whether our location is of sufficient quality to allow use to feed

References Vole\_Base::CalculateCarryingCapacity(), TAnimal::m\_Location\_x, TAnimal::m\_Location\_y, and MinMVoleHabQual.

```
03194 {
03195     if (CalculateCarryingCapacity(m_Location_x, m_Location_y)>=
03196       double(MinMVoleHabQual))  return true;
03197       // Min suitable quality
03198         else return false;
03199 }
```

|  |  |  |  |  |  |
| --- | --- | --- | --- | --- | --- |
| int Vole\_Male::DecideQualityAction | ( | double | *Quality* | ) | `[protected]` |

Male vole decision making behaviour.

Decision making method to determine what the vole ought to do given his physiogical state and territory quality.

References Vole\_Base::m\_DispVector, Vole\_Base::m\_Have\_Territory, Vole\_Base::m\_Reserves, and Vole\_Base::m\_StarvationDays.

Referenced by st\_Eval\_n\_Explore().

```
02826 {
02827   int return_value=0;
02828   if (Quality > MHabQualThreshold3)
02829   {
02830     // Very Good
02831     m_Reserves++;
02832     m_StarvationDays = 0;
02833     if (m_Reserves > 3) m_Reserves = 3;
02834     //return_value=0; Is already so not needed
02835   }
02836   else
02837   {
02838     if (Quality>MHabQualThreshold2)
02839     {
02840       // Average quality
02841       m_Reserves++;
02842       m_StarvationDays = 0;
02843       if (m_Reserves > 3) m_Reserves = 3;
02844            // check an area MinMaleMovement to MaleMovement metres away
02845       return_value=1;
02846     }
02847     else if (Quality >MHabQualThreshold1) // V. bad
02848     {
02849       // v.bad but not forced out so will do an explore
02850       if (Quality>1) m_StarvationDays = 0; else m_Reserves--;
02851       m_DispVector = random(8); // choose a direction
02852       // check an area MinMaleMovement to MaleMovement metres away
02853       return_value=1;
02854     }
02855     else  // Forced out - Habitat Quality is too low
02856     {
02857       if (Quality<1) m_Reserves--;
02858       if (m_Have_Territory == true)
02859       {
02860         m_Have_Territory = false;
02861         m_DispVector = random(8);
02862         // Not quality dependent dispersal, very directed
02863       }
02864       if (m_Reserves == -1)
02865       {
02866         m_Reserves = 0;
02867         m_StarvationDays++;
02868       }
02869       if (m_StarvationDays>MaxStarvationDays)
02870       {
02871         return_value=3; // Die of starvation
02872       }
02873       else return_value=2;   // Dispersal
02874     }
02875   }
02876   return return_value;
02877 }
```

|  |  |  |  |  |
| --- | --- | --- | --- | --- |
| void Vole\_Male::DetermineTerritorySize | ( |  | ) | `[inline, protected]` |

Calculates the territory size needed for a vole of his weight.

References Vole\_Base::m\_TerrRange, Vole\_Base::m\_Weight, MaleTerritoryRange, MaleTerritoryRangeSlope, MinReproAgeM, and MinReproWeightM.

Referenced by EndStep().

```
02982 {
02983     if (m_Weight>=MinReproAgeM)
02984     {
02985       m_TerrRange=MaleTerritoryRange+(MaleTerritoryRangeSlope*
02986                                                     ((int)m_Weight-MinReproWeightM));
02987     }
02988 }
```

|  |  |  |  |
| --- | --- | --- | --- |
| int Vole\_Male::Dispersal | ( | double | *p\_OldQual*, |
|  |  | int | *p\_Distance* |  |
|  | ) |  |  | `[protected]` |

Male vole dispersal behaviour.

Works like female dispersal - but a return code of 3 will trigger infanticide   
Checks p\_Distance away to see if it can find a territory with a higher quality than p\_OldQual  
This entails some risk though, so there is a fixed 2.5% increase in the mortality chance when it does this.

References Vole\_Base::CalculateCarryingCapacity(), FreeLocation(), g\_rand\_uni, Vole\_Base::m\_Age, Vole\_Base::m\_DispVector, Vole\_Base::m\_Have\_Territory, TAnimal::m\_Location\_x, TAnimal::m\_Location\_y, Vole\_Base::m\_Mature, TAnimal::m\_OurLandscape, Vole\_Base::m\_OurPopulation, Vole\_Base::m\_Reserves, Vole\_Base::m\_StarvationDays, Vole\_Base::m\_TerrRange, MinMVoleHabQual, Vole\_Base::MoveTo(), SetLocation(), and Vole\_Population\_Manager::SupplyGrowthStartDate().

Referenced by st\_Eval\_n\_Explore().

```
02891 {
02892     // Returns 1 for die, 0 for carry on, 3 for infanticide
02893     // Do a predation test
02894     if (!m_Have_Territory) {
02895       if (g_rand_uni() < g_extradispmort ) {  
02896                 return 1;
02897           }
02898         }
02899     bool Had_Terr=m_Have_Territory;
02900     bool BreedingSeason=false;
02901     int today=m_OurLandscape->SupplyDayInYear();
02902     if ((today>m_OurPopulation->SupplyGrowthStartDate())
02903                             &&(today<=MaleReproductFinish)) BreedingSeason=true;
02904     // p_OldQuatells whether dispersal is conditional on quality or not
02905     // p_OldQual is set to old habitat quality or -1
02906     // p_Distance is the p_Distance used by the move function
02907 
02908     // aim is to move in a directed way traversing the landscape using the best
02909     // habitats
02910     // remember the old co-ordinates in case infanticide needs to be triggered
02911     int oldx = m_Location_x;
02912     int oldy = m_Location_y;
02913     if (m_DispVector == -1) m_DispVector = random(8); // Choose direction 0-7
02914     // Go that far in that direction (assuming it is possible to do that)
02915     MoveTo(m_DispVector, p_Distance,10);
02916     //  Now we are there so what is the new quality
02917 
02918     // Get the carrying capacity
02919     double CC = CalculateCarryingCapacity(m_Location_x,m_Location_y);
02920     if (p_OldQual==-1) // Must move
02921     {
02922       int Fems=1;
02923       if (BreedingSeason && m_Mature)
02924       {
02925         Fems=m_OurPopulation->
02926              SupplyInOlderTerr(m_Location_x,m_Location_y,m_Age,m_TerrRange);
02927       }
02928       // 3. Now have the information to make a decision establish territory
02929       if ((CC<MinMVoleHabQual)||(Fems<1))
02930       {
02931         // Can't establish Territory Here
02932         m_Have_Territory=false;
02933         m_Reserves--;
02934         if (m_Reserves == -1)
02935         {
02936           m_Reserves = 0;
02937           m_StarvationDays++;
02938         }
02939         if (m_StarvationDays>MaxStarvationDays)
02940         {
02941           return 1; // Die of starvation
02942         }
02943       }
02944       else
02945       {
02946         // Can establish territory
02947         m_Have_Territory=true;
02948       }
02949     }
02950     else
02951     {
02952       if (CC<=p_OldQual)
02953       {
02954         // Don't want to move
02955         FreeLocation();
02956         m_Location_x=oldx;
02957         m_Location_y=oldy;
02958         SetLocation();
02959       }
02960       else
02961       {
02962         // Can establish territory
02963         m_Have_Territory=true;
02964       }
02965     }
02966     // Have we moved more than _TerrRange from old home
02967     if (m_Have_Territory && BreedingSeason)
02968     {
02969       if ((abs(oldx-m_Location_x)>m_TerrRange)
02970         || (abs(oldy-m_Location_y)>m_TerrRange))
02971            return 3;
02972       else if(!Had_Terr) return 3; // This will result in an infanticide attempt if females in the new territory have young.
02973     }
02974     return 0;
02975 }
```

|  |  |  |  |  |  |
| --- | --- | --- | --- | --- | --- |
| void Vole\_Male::EndStep | ( | void |  | ) | `[virtual]` |

Male vole EndStep.

The EndStep is one of the three timestep divisions. This is called once for each vole after BeginStep and Step.   
The main function here is to remove voles that have died during step and otherwise to grow if not at max weight. It also checks if the vole was killed due to human management and determines the potential territory size.

Reimplemented from Vole\_Base.

References TAnimal::CheckManagement(), Vole\_Base::CurrentVState, DetermineTerritorySize(), GrowStopDate, growthperdayM, Vole\_Base::m\_Age, Vole\_Base::m\_Mature, TAnimal::m\_OurLandscape, Vole\_Base::m\_OurPopulation, Vole\_Base::m\_Weight, MaxWeightM, Vole\_Base::st\_Dying(), Vole\_Population\_Manager::SupplyGrowthStartDate(), and tovs\_MDying.

```
02603 {
02604   CheckManagement();
02605   if (CurrentVState==tovs_MDying)
02606   {
02607     st_Dying();
02608   }
02609   else
02610   {
02611     m_Age++;
02612     /*              MALE GROWTH NOTES
02613 
02614      Male vole grows until 20g. After that he will only grow if he has matured
02615      Growth continues up to 60g.
02616 
02617      Reproduction cannot occur below 40g or 40 days
02618 
02619      Growth only occurs between 1 March and 1st August
02620      taken from Hanson L, 1977, Oikos 29.
02621     */
02622     if ((m_OurLandscape->SupplyDayInYear()<GrowStopDate) && (m_OurLandscape->
02623              SupplyDayInYear()>m_OurPopulation->SupplyGrowthStartDate()))
02624     {
02625       if (m_Weight<20)
02626       {
02627         m_Weight+=growthperdayM;
02628       }
02629       else if ((m_Mature==true) && (m_Weight<MaxWeightM))
02630       {
02631         m_Weight+=growthperdayM;
02632       }
02633     }
02634     DetermineTerritorySize();
02635   }
02636 }
```

|  |  |  |  |  |
| --- | --- | --- | --- | --- |
| void Vole\_Male::FreeLocation | ( |  | ) | `[inline, protected, virtual]` |

Map location function.

Reimplemented from Vole\_Base.

References TAnimal::m\_Location\_x, TAnimal::m\_Location\_y, Vole\_Base::m\_OurPopulation, and Vole\_Population\_Manager::VoleMap.

Referenced by Dispersal(), and Step().

```
03212                                     {
03213      m_OurPopulation->VoleMap->ClearMapValue(m_Location_x,m_Location_y);
03214     };
```

|  |  |  |  |  |
| --- | --- | --- | --- | --- |
| int Vole\_Male::GetFertile | ( |  | ) | `[inline]` |

Get the male vole fertility.

Primarily used in ecotoxicological simulations where toxic effects may render the vole sterile.

References m\_fertile.

Referenced by Vole\_Female::st\_Mating().

```
00230                      {
00234                 return m_fertile;
00235         }
```

|  |  |  |  |
| --- | --- | --- | --- |
| bool Vole\_Male::GetLocation | ( | int | *px*, |
|  |  | int | *py* |  |
|  | ) |  |  | `[inline, protected, virtual]` |

Map location function.

Reimplemented from Vole\_Base.

References Vole\_Base::m\_OurPopulation, and Vole\_Population\_Manager::VoleMap.

```
03219                                                  {
03220      if( m_OurPopulation->VoleMap->GetMapValue(px,py)) return true;
03221          return false;
03222     };
```

|  |  |  |  |  |  |
| --- | --- | --- | --- | --- | --- |
| void Vole\_Male::Init | ( | void |  | ) | `[protected]` |

Sets default values.

Called everytime a new male vole is created.

References Vole\_Base::m\_Age, Vole\_Base::m\_Sex, Vole\_Base::m\_TerrRange, Vole\_Base::m\_Weight, MaleTerritoryRange, Vole\_Base::MyGenes, GeneticMaterial::ScoreHQThreshold(), and WeanedWeight.

Referenced by Vole\_Male().

```
02493 {
02494     m_Sex=true;
02495     m_TerrRange=MaleTerritoryRange;
02496     m_Age=14;
02497     m_Weight=WeanedWeight;
02498 #ifdef __PHENOTYPIC_LINK_HQUAL
02499   // 1. Score our genetic code
02500   //
02501   double MultiplicationFactor=MyGenes.ScoreHQThreshold();
02502   // 2. Initialise parameter values
02503   //
02504   MHQThresh1 = MHabQualThreshold1 + MultiplicationFactor;
02505   MHQThresh2 = MHabQualThreshold2;
02506   MHQThresh3 = MHabQualThreshold3;
02507 #endif
02508 }
```

|  |  |  |  |  |  |
| --- | --- | --- | --- | --- | --- |
| bool Vole\_Male::OnFarmEvent | ( | FarmToDo | *event* | ) | `[virtual]` |

Male vole exernal event handler.

This method evaluates external events and chooses a suitable response (in this case a probability of dying because other effects will be taken up by the evaluate and explore state.

Reimplemented from TAnimal.

References autumn\_harrow, autumn\_or\_spring\_plough, autumn\_plough, autumn\_roll, autumn\_sow, burn\_straw\_stubble, cattle\_out, Vole\_Base::CurrentVState, cut\_to\_hay, cut\_to\_silage, cut\_weeds, deep\_ploughing, fa\_ammoniumsulphate, fa\_greenmanure, fa\_manure, fa\_npk, fa\_pk, fa\_sludge, fa\_slurry, fp\_greenmanure, fp\_liquidNH3, fp\_manganesesulphate, fp\_manure, fp\_npk, fp\_npks, fp\_pk, fp\_sludge, fp\_slurry, fungicide\_treat, g\_rand\_uni, growth\_regulator, harvest, hay\_bailing, hay\_turning, herbicide\_treat, hilling\_up, insecticide\_treat, TAnimal::m\_OurLandscape, molluscicide, mow, pigs\_out, row\_cultivation, sleep\_all\_day, spring\_harrow, spring\_plough, spring\_roll, spring\_sow, straw\_chopping, strigling, strigling\_sow, stubble\_harrowing, swathing, tovs\_MDying, water, and winter\_plough.

```
02999 {
03000  switch(event)
03001  {
03002 case  sleep_all_day:
03003   break;
03004 case  autumn_plough:
03005   if (g_rand_uni()<VoleSoilCultivationMort)
03006      CurrentVState=tovs_MDying;
03007   break;
03008 case  autumn_harrow:
03009   if (g_rand_uni()<VoleSoilCultivationMort)
03010      CurrentVState=tovs_MDying;
03011   break;
03012 case  autumn_roll:
03013   if (g_rand_uni()<VoleSoilCultivationMort)
03014      CurrentVState=tovs_MDying;
03015   break;
03016 case  autumn_sow:
03017   if (g_rand_uni()<VoleSoilCultivationMort)
03018      CurrentVState=tovs_MDying;
03019   break;
03020 case  winter_plough:
03021   if (g_rand_uni()<VoleSoilCultivationMort)
03022      CurrentVState=tovs_MDying;
03023   break;
03024 case  deep_ploughing:
03025   if (g_rand_uni()<VoleSoilCultivationMort)
03026      CurrentVState=tovs_MDying;
03027   break;
03028 case  spring_plough:
03029   if (g_rand_uni()<VoleSoilCultivationMort)
03030      CurrentVState=tovs_MDying;
03031   break;
03032 case  spring_harrow:
03033   if (g_rand_uni()<VoleSoilCultivationMort)
03034      CurrentVState=tovs_MDying;
03035   break;
03036 case  spring_roll:
03037   if (g_rand_uni()<VoleSoilCultivationMort)
03038      CurrentVState=tovs_MDying;
03039   break;
03040 case  spring_sow:
03041   if (g_rand_uni()<VoleSoilCultivationMort)
03042      CurrentVState=tovs_MDying;
03043   break;
03044 case  fp_npks:
03045   break;
03046 case  fp_npk:
03047   break;
03048 case  fp_pk:
03049   break;
03050 case  fp_liquidNH3:
03051   break;
03052 case  fp_slurry:
03053   break;
03054 case  fp_manganesesulphate:
03055   break;
03056 case  fp_manure:
03057   break;
03058 case  fp_greenmanure:
03059   break;
03060 case  fp_sludge:
03061   break;
03062 case  fa_npk:
03063   break;
03064 case  fa_pk:
03065   break;
03066 case  fa_slurry:
03067   break;
03068 case  fa_ammoniumsulphate:
03069   break;
03070 case  fa_manure:
03071   break;
03072 case  fa_greenmanure:
03073   break;
03074 case  fa_sludge:
03075   break;
03076 case  herbicide_treat:
03077   if (g_rand_uni()<VoleHerbicicideMort)
03078      CurrentVState=tovs_MDying;
03079   break;
03080 case  growth_regulator:
03081   break;
03082 case  fungicide_treat:
03083   break;
03084 case  insecticide_treat:
03085   if (g_rand_uni()<VoleInsecticideMort)
03086      CurrentVState=tovs_MDying;
03087   break;
03088 case  molluscicide:
03089   break;
03090 case  row_cultivation:
03091   if (g_rand_uni()<VoleSoilCultivationMort)
03092      CurrentVState=tovs_MDying;
03093   break;
03094 case  strigling:
03095   if (g_rand_uni()<VoleStriglingMort)
03096      CurrentVState=tovs_MDying;
03097   break;
03098 case  hilling_up:
03099   if (g_rand_uni()<VoleSoilCultivationMort)
03100      CurrentVState=tovs_MDying;
03101   break;
03102 case  water:
03103   break;
03104 case  swathing:
03105   if (g_rand_uni()<VoleHarvestMort)
03106      CurrentVState=tovs_MDying;
03107   break;
03108 case  harvest:
03109   if (g_rand_uni()<VoleHarvestMort)
03110      CurrentVState=tovs_MDying;
03111   break;
03112 case  cattle_out:
03113   break;
03114 case  cut_to_hay:
03115   if (g_rand_uni()<VoleHarvestMort)
03116      CurrentVState=tovs_MDying;
03117   break;
03118 case  cut_to_silage:
03119   if (g_rand_uni()<VoleHarvestMort)
03120      CurrentVState=tovs_MDying;
03121   break;
03122 case  straw_chopping:
03123   if (g_rand_uni()<VoleHarvestMort)
03124      CurrentVState=tovs_MDying;
03125   break;
03126 case  hay_turning:
03127   if (g_rand_uni()<VoleHarvestMort)
03128      CurrentVState=tovs_MDying;
03129   break;
03130 case  hay_bailing:
03131   if (g_rand_uni()<VoleHarvestMort)
03132      CurrentVState=tovs_MDying;
03133   break;
03134 case  stubble_harrowing:
03135   if (g_rand_uni()<VoleSoilCultivationMort)
03136      CurrentVState=tovs_MDying;
03137   break;
03138 case  autumn_or_spring_plough:
03139   if (g_rand_uni()<VoleSoilCultivationMort)
03140      CurrentVState=tovs_MDying;
03141   break;
03142 case  burn_straw_stubble:
03143   if (g_rand_uni()<VoleSoilCultivationMort)
03144      CurrentVState=tovs_MDying;
03145   break;
03146 case mow:
03147   if (g_rand_uni()<VoleHarvestMort)
03148      CurrentVState=tovs_MDying;
03149   break;
03150 case cut_weeds:
03151   if (g_rand_uni()<VoleHarvestMort)
03152      CurrentVState=tovs_MDying;
03153   break;
03154 case pigs_out:
03155   if (g_rand_uni()<VolePigGrazingMort)
03156      CurrentVState=tovs_MDying;
03157   break;
03158 case strigling_sow:
03159   if (g_rand_uni()<VoleSoilCultivationMort)
03160      CurrentVState=tovs_MDying;
03161   break;
03162 default:
03163   g_msg->Warn( WARN_FILE, "Vole_Male::OnFarmEvent(): Unknown event type:",
03164           m_OurLandscape->EventtypeToString(event) );
03165   exit( 1 );
03166  }
03167  if (CurrentVState==tovs_MDying) return true;
03168  else
03169  return false;
03170 }
```

|  |  |  |  |  |
| --- | --- | --- | --- | --- |
| void Vole\_Male::OnKilled | ( |  | ) | `[virtual]` |

Male vole death by external entity.

Response to external death event - most likely eaten by a explicitly modelled predator

Reimplemented from Vole\_Base.

References Vole\_Base::CurrentVState, and tovs\_MDying.

Referenced by Vole\_Population\_Manager::DoFirst().

```
03182 {
03183   CurrentVState=tovs_MDying;
03184 }
```

|  |  |  |  |  |  |
| --- | --- | --- | --- | --- | --- |
| void Vole\_Male::SetFertile | ( | int | *f* | ) | `[inline]` |

Set the male vole fertility.

Primarily used in ecotoxicological simulations where toxic effects may render the vole sterile.

References m\_fertile.

Referenced by Vole\_Population\_Manager::CreateObjects(), and Vole\_Population\_Manager::CreateObjects\_Init().

```
00220                            {
00224                 m_fertile=f;
00225         }
```

|  |  |  |  |  |
| --- | --- | --- | --- | --- |
| void Vole\_Male::SetLocation | ( |  | ) | `[inline, protected, virtual]` |

Map location function.

Reimplemented from Vole\_Base.

References TAnimal::m\_Location\_x, TAnimal::m\_Location\_y, Vole\_Base::m\_OurPopulation, and Vole\_Population\_Manager::VoleMap.

Referenced by Dispersal().

```
03205                                    {
03206      m_OurPopulation->VoleMap->SetMapValue(m_Location_x,m_Location_y,this);
03207     };
```

|  |  |  |  |  |  |
| --- | --- | --- | --- | --- | --- |
| int Vole\_Male::st\_Eval\_n\_Explore | ( | void |  | ) | `[protected]` |

Male vole main territory assessment behaviour.

Evaluates the quality of his habitat and does some limited exploration in the surrounding area to see if she can improve it by moving.

References Vole\_Base::CalculateCarryingCapacity(), DecideQualityAction(), Dispersal(), Vole\_Base::m\_Age, Vole\_Base::m\_DispVector, Vole\_Base::m\_Have\_Territory, TAnimal::m\_Location\_x, TAnimal::m\_Location\_y, Vole\_Base::m\_Mature, TAnimal::m\_OurLandscape, Vole\_Base::m\_OurPopulation, Vole\_Base::m\_TerrRange, Vole\_Population\_Manager::SupplyGrowthStartDate(), and Vole\_Population\_Manager::SupplyHowManyVoles().

Referenced by Step().

```
02697 {
02698     unsigned MovementIndex;
02699     if (m_Age>90) MovementIndex=3;
02700      else if (m_Age>60) MovementIndex=2;
02701        else if (m_Age>30) MovementIndex=1;
02702          else MovementIndex=0;
02703     int today=m_OurLandscape->SupplyDayInYear();
02704     if ((today>=m_OurPopulation->SupplyGrowthStartDate())
02705                                     &&(today<=MaleReproductFinish)&&(m_Mature))
02706     {
02707       // 1. If he is in an older males territory then he must move
02708       // 2. If there are no females here he must move
02709       // 3. Otherwise stay put
02710 
02711       // Must move if in an older males territory
02712       int NoFems=m_OurPopulation->
02713           SupplyInOlderTerr(m_Location_x,m_Location_y,m_Age,m_TerrRange);
02714       if (NoFems<1) // In larger males territory, must move or are no females
02715       {
02716         m_Have_Territory = false;
02717         m_DispVector = random(8);
02718         // Just need to move and try again later
02719         // Not quality dependent dispersal, very directed
02720         return(Dispersal(-1,MaleMovement[MovementIndex]));
02721       }
02722       return 0;
02723     }
02724     // Outside the breeding season, he doesn't care about other males
02725     // or females, only the habitat quality
02726     double Qual = CalculateCarryingCapacity(m_Location_x, m_Location_y);
02727     int Voles=m_OurPopulation->SupplyHowManyVoles(m_Location_x,m_Location_y,
02728                                                                   m_TerrRange);
02729     if (Voles<1) Voles=1;
02730     Qual/=(double)Voles;
02731     // This function determines whether he must leave and whether he does
02732     // an explore. He will do this if he is not in optimal conditions
02733     switch (DecideQualityAction(Qual))
02734     {
02735       case 0:  // Excellent habitat
02736         break;
02737       case 1:  // OK, quality dependent dispersal
02738         m_DispVector=-1;
02739         return(Dispersal(Qual,MaleMovement[MovementIndex]));
02740       case 2:  // Poor quality, forced disperse
02741         return(Dispersal(-1,MaleMovement[MovementIndex]));
02742       case 3:
02743         return 1; // Die
02744       default:
02745         m_OurLandscape->Warn("Vole_Male::Eval&Explore - unknown return error",NULL);
02746         exit(1);
02747     }
02748     return 0;
02749 }
```

|  |  |  |  |  |  |
| --- | --- | --- | --- | --- | --- |
| void Vole\_Male::st\_Infanticide | ( | void |  | ) | `[protected]` |

Male vole infanticide behaviour.

Will only enter here if have taken over a new area a reasonable distance from the old one.   

Tells the population manager to send an infanticide message to all females in the territory.

References TAnimal::m\_Location\_x, TAnimal::m\_Location\_y, Vole\_Base::m\_OurPopulation, Vole\_Base::m\_TerrRange, Vole\_Population\_Manager::SendMessage(), and tovm\_Infanticide.

Referenced by Step().

```
02651 {
02652     m_OurPopulation->SendMessage(tovm_Infanticide,m_Location_x,m_Location_y,m_TerrRange,0,false);
02653 }
```

|  |  |  |  |  |  |
| --- | --- | --- | --- | --- | --- |
| void Vole\_Male::st\_JuvenileExplore | ( | void |  | ) | `[protected]` |

Extra movement on weaning.

Cause the vole to do some exploration on maturity, just to move him away from the litter centre.   
After this first day he will go into the normal Eval\_n\_Explore.

References Vole\_Base::MoveTo().

Referenced by Step().

```
02665 {
02666     // Just do some movement at first
02667     MoveTo(random(8),MinMaleMovement,20);
02668 }
```

|  |  |  |  |  |  |
| --- | --- | --- | --- | --- | --- |
| int Vole\_Male::st\_Maturation | ( | void |  | ) | `[protected]` |

Male vole maturation control.

Decide whether to become mature or not

References Vole\_Base::m\_Age, TAnimal::m\_OurLandscape, Vole\_Base::m\_OurPopulation, and Vole\_Population\_Manager::SupplyGrowthStartDate().

Referenced by Step().

```
02679 {
02680     // Male must be 40 days old - minimum before maturation
02681     // date must be after 29/3
02682     if (m_Age < 41) return 0; // don't mature
02683     if (m_OurLandscape->SupplyDayInYear()<
02684               m_OurPopulation->SupplyGrowthStartDate()) return 0;// don't mature
02685     else return 1; // matures
02686 }
```

|  |  |  |  |  |  |
| --- | --- | --- | --- | --- | --- |
| void Vole\_Male::Step | ( | void |  | ) | `[virtual]` |

Male vole Step.

The Step is one of the three timestep divisions. This is called repeatedly after BeginStep and before EndStep, until all voles report that they are done with Step.   
  
Most of the behaviours are controlled by moving voles between behavioural states in Step (for other models this is also done in BeginStep and EndStep).   
When a vole is done for the day it will signal this by setting StepDone==true. NB that a call to one behaviour may trigger a call to another behaviour on the next call to step inside the same timestep. In this way a daily cycle of activity can be undertaken.

Reimplemented from Vole\_Base.

References TALMaSSObject::CurrentStateNo, Vole\_Base::CurrentVState, FreeLocation(), Vole\_Base::m\_Mature, TAnimal::m\_OurLandscape, st\_Eval\_n\_Explore(), st\_Infanticide(), st\_JuvenileExplore(), st\_Maturation(), TALMaSSObject::StepDone, tovs\_Infanticide, tovs\_InitialState, tovs\_JuvenileExploration, tovs\_MDying, tovs\_MEvaluateExplore, and tovs\_MMaturation.

```
02539 {
02540   if (StepDone || CurrentStateNo == -1) return;
02541   switch (CurrentVState)
02542   {
02543    case tovs_InitialState:  //initial state
02544      CurrentVState=tovs_JuvenileExploration;
02545      break;
02546    case tovs_JuvenileExploration: // Juvenile Exploration
02547     st_JuvenileExplore();
02548     CurrentVState=tovs_MEvaluateExplore;
02549     StepDone=true;
02550     break;
02551    case tovs_MMaturation: // Maturation
02552     if (st_Maturation()) m_Mature=true;
02553     CurrentVState=tovs_MEvaluateExplore; // Eval and Explore
02554     StepDone=true;
02555     break;
02556    case tovs_MEvaluateExplore: // Eval&Explore
02557     switch(st_Eval_n_Explore())
02558     {
02559       case 0:  // No change
02560         break;
02561       case 1:  // Died of starvation
02562         CurrentVState=tovs_MDying;
02563         StepDone=true;
02564         break;
02565       case 2: // New territory but not far from old
02566         break;
02567       case 3: // New territory long way from old
02568         CurrentVState=tovs_Infanticide; // Infanticide
02569         break;
02570     }
02571     if (m_Mature)
02572     {
02573       StepDone=true;
02574     }
02575     else if (!StepDone) CurrentVState=tovs_MMaturation;
02576     break;
02577    case tovs_Infanticide: // Infanticide
02578     st_Infanticide();
02579     CurrentVState=tovs_MEvaluateExplore;
02580     break;
02581    case tovs_MDying:
02582     FreeLocation();
02583     StepDone=true;
02584     break;
02585    default:
02586      m_OurLandscape->Warn("Vole_Male::Step - unknown return error",NULL);
02587      exit(1);
02588 
02589   }
02590 }
```

---

## Member Data Documentation

|  |
| --- |
| int Vole\_Male::m\_fertile `[protected]` |

Flag indicating the fertility state (0=fertile).

Referenced by GetFertile(), and SetFertile().

---

The documentation for this class was generated from the following files:

- vole\_all.h- Vole\_all.cpp

---

Generated on Thu Jan 22 14:13:48 2009 for ALMaSS ODDox by 
 1.5.6 
