# Supplementary material for: Predation and fragmentation portrayed in the statistical structure of prey time series
Source: BMC Ecol. 2009 May 6;9:10. doi: 10.1186/1472-6785-9-10 (PMC2689204; doi:10.1186/1472-6785-9-10)
Supplement: Additional file 2 — Voles and related classes ODDox Documentation. ODDox documentation of the agent-based model (ALMaSS) applied by Hendrichsen et al. The documentation is started by activating main.html. [file 1472-6785-9-10-S2.zip › Vole_ODDox/class_vole___population___manager-members.html]

ALMaSS ODDox: Member List

- Main Page
- Related Pages
- Classes
- Files

- Alphabetical List
- Class List
- Class Hierarchy
- Class Members

# Vole\_Population\_Manager Member List

This is the complete list of members for Vole\_Population\_Manager, including all inherited members.

|  |  |  |
| --- | --- | --- |
| AddToGeneticImpacted() | Vole\_Population\_Manager | `[inline]` |
| AddToImpacted() | Vole\_Population\_Manager | `[inline]` |
| AddToJuvs(int juvs) | Vole\_Population\_Manager | `[inline]` |
| AddToNotImpacted() | Vole\_Population\_Manager | `[inline]` |
| AddToYoung(int young) | Vole\_Population\_Manager | `[inline]` |
| AlFreq | Vole\_Population\_Manager |  |
| BeforeStepActions | Population\_Manager | `[protected]` |
| BeginningOfMonth() | Population\_Manager |  |
| BreedingPairsOutput(int, int, int) | Population\_Manager | `[inline, virtual]` |
| BreedingSuccessProbeOutput(double, int, int, int, int, int, int, int) | Population\_Manager | `[inline, virtual]` |
| Catastrophe() | Vole\_Population\_Manager | `[protected, virtual]` |
| Population\_Manager::Catastrophe(int) | Population\_Manager | `[inline, virtual]` |
| CIPEGridOutputPrb | Population\_Manager | `[protected]` |
| CIPEGridOutputPrbB | Population\_Manager | `[protected]` |
| CloseTheCIPEGridOutputProbe() | Population\_Manager |  |
| CloseTheMonthlyRipleysOutputProbe() | Population\_Manager |  |
| CloseTheReallyBigOutputProbe() | Population\_Manager |  |
| CloseTheRipleysOutputProbe() | Population\_Manager |  |
| Counts | Population\_Manager |  |
| CreateObjects(int ob\_type, TAnimal \*pvo, struct\_Vole\_Adult \*data, int number) | Vole\_Population\_Manager |  |
| CreateObjects\_Init(int ob\_type, TAnimal \*pvo, struct\_Vole\_Adult \*data, int number) | Vole\_Population\_Manager |  |
| DisplayLocations() | Population\_Manager | `[virtual]` |
| DoAfter() | Population\_Manager | `[protected, virtual]` |
| DoAlmostLast() | Population\_Manager | `[protected, virtual]` |
| DoBefore() | Population\_Manager | `[protected, virtual]` |
| DoFirst() | Vole\_Population\_Manager | `[protected, virtual]` |
| DoLast() | Population\_Manager | `[protected, virtual]` |
| FindClosest(int x, int y, unsigned Type) | Population\_Manager |  |
| FindClosestFemale(int p\_x, int p\_y, int p\_steps) | Vole\_Population\_Manager |  |
| FindClosestMale(int p\_x, int p\_y, int p\_steps) | Vole\_Population\_Manager |  |
| FindRandomMale() | Vole\_Population\_Manager |  |
| FledgelingProbeOutput(int, int) | Population\_Manager | `[inline, virtual]` |
| FList | Vole\_Population\_Manager |  |
| GeneticsResultsOutput(FILE \*ofile, unsigned listindex) | Vole\_Population\_Manager | `[virtual]` |
| gridcount | Population\_Manager | `[protected]` |
| IDNumber | Vole\_Population\_Manager |  |
| ImpactedProbe() | Vole\_Population\_Manager | `[virtual]` |
| ImpactProbeReport(int a\_Time) | Population\_Manager |  |
| IndexArrayX | Population\_Manager |  |
| Init(void) | Vole\_Population\_Manager | `[virtual]` |
| InSquare(int p\_x, int p\_y, int p\_sqx, int p\_sqy, int p\_range) | Vole\_Population\_Manager |  |
| IsLast(unsigned listindex) | Population\_Manager | `[inline]` |
| JuvsProducedToday | Vole\_Population\_Manager |  |
| LamdaBirth(int x, int y) | Population\_Manager | `[inline]` |
| LamdaBirth(int x, int y, int z) | Population\_Manager | `[inline]` |
| LamdaClear() | Population\_Manager | `[inline]` |
| LamdaDeath(int x, int y) | Population\_Manager | `[inline]` |
| LamdaDumpOutput() | Population\_Manager |  |
| lamdagrid | Population\_Manager | `[protected]` |
| ListClosestFemales(int p\_x, int p\_y, int p\_steps) | Vole\_Population\_Manager |  |
| ListClosestMales(int p\_x, int p\_y, int p\_steps) | Vole\_Population\_Manager |  |
| ListNameLength | Population\_Manager | `[protected]` |
| ListNames | Population\_Manager | `[protected]` |
| LOG(const char \*fname) | Population\_Manager |  |
| m\_AlleleFreqsFile | Population\_Manager | `[protected]` |
| m\_catastrophestartyear | Population\_Manager | `[protected]` |
| m\_EasyPopRes | Population\_Manager | `[protected]` |
| m\_f1sterilitychance | Vole\_Population\_Manager | `[protected]` |
| m\_geneticimpacted | Vole\_Population\_Manager | `[protected]` |
| m\_geneticproductfertilityeffect | Vole\_Population\_Manager |  |
| m\_GeneticsFile | Population\_Manager | `[protected]` |
| m\_geneticsterilitychance | Vole\_Population\_Manager | `[protected]` |
| m\_GrowthStartDate | Vole\_Population\_Manager | `[protected]` |
| m\_impacted | Vole\_Population\_Manager | `[protected]` |
| m\_MainForm | Population\_Manager |  |
| m\_NoProbes | Population\_Manager | `[protected]` |
| m\_notimpacted | Vole\_Population\_Manager | `[protected]` |
| m\_SimulationName | Population\_Manager |  |
| m\_StepSize | Population\_Manager | `[protected]` |
| m\_TheLandscape | Population\_Manager |  |
| MList | Vole\_Population\_Manager |  |
| OpenTheBreedingPairsProbe() | Population\_Manager | `[inline, virtual]` |
| OpenTheBreedingSuccessProbe() | Population\_Manager | `[inline, virtual]` |
| OpenTheCIPEGridOutputProbe() | Population\_Manager |  |
| OpenTheFledgelingProbe() | Population\_Manager | `[inline, virtual]` |
| OpenTheMonthlyRipleysOutputProbe() | Population\_Manager |  |
| OpenTheReallyBigProbe() | Population\_Manager |  |
| OpenTheRipleysOutputProbe() | Population\_Manager |  |
| Population\_Manager(Landscape \*L) | Population\_Manager |  |
| Probe(int ListIndex, probe\_data \*p\_TheProbe) | Population\_Manager | `[virtual]` |
| ProbeFileInput(char \*p\_Filename, int p\_ProbeNo) | Population\_Manager |  |
| ProbeReport(int a\_time) | Population\_Manager |  |
| ProbesSet | Population\_Manager |  |
| ReallyBigOutputPrb | Population\_Manager | `[protected]` |
| ReproductionProbe() | Vole\_Population\_Manager |  |
| ReproTable | Vole\_Population\_Manager |  |
| RipleysOutputPrb | Population\_Manager | `[protected]` |
| RipleysOutputPrb1 | Population\_Manager | `[protected]` |
| RipleysOutputPrb10 | Population\_Manager | `[protected]` |
| RipleysOutputPrb11 | Population\_Manager | `[protected]` |
| RipleysOutputPrb12 | Population\_Manager | `[protected]` |
| RipleysOutputPrb2 | Population\_Manager | `[protected]` |
| RipleysOutputPrb3 | Population\_Manager | `[protected]` |
| RipleysOutputPrb4 | Population\_Manager | `[protected]` |
| RipleysOutputPrb5 | Population\_Manager | `[protected]` |
| RipleysOutputPrb6 | Population\_Manager | `[protected]` |
| RipleysOutputPrb7 | Population\_Manager | `[protected]` |
| RipleysOutputPrb8 | Population\_Manager | `[protected]` |
| RipleysOutputPrb9 | Population\_Manager | `[protected]` |
| Run(int NoTSteps) | Population\_Manager | `[virtual]` |
| SendMessage(TTypeOfVoleMessage p\_message, unsigned p\_x, unsigned p\_y, unsigned p\_range, unsigned p\_age, bool p\_sex) | Vole\_Population\_Manager |  |
| SetNoProbes(int a\_pn) | Population\_Manager | `[inline]` |
| Shuffle(unsigned Type) | Population\_Manager | `[protected]` |
| Shuffle\_or\_Sort(unsigned Type) | Population\_Manager | `[protected]` |
| SimH | Population\_Manager |  |
| SimW | Population\_Manager |  |
| SortState(unsigned Type) | Population\_Manager | `[protected]` |
| SortX(unsigned Type) | Population\_Manager | `[protected]` |
| SortXIndex(unsigned Type) | Population\_Manager | `[protected]` |
| SortY(unsigned Type) | Population\_Manager | `[protected]` |
| SpeciesSpecificReporting(int a\_species, int a\_time) | Population\_Manager |  |
| StateList | Population\_Manager | `[protected]` |
| StateNames | Population\_Manager | `[protected]` |
| StateNamesLength | Population\_Manager | `[protected]` |
| StepFinished() | Population\_Manager | `[protected, virtual]` |
| SupplyCovPosx(int) | Population\_Manager | `[inline, virtual]` |
| SupplyCovPosy(int) | Population\_Manager | `[inline, virtual]` |
| SupplyGrowthStartDate() | Vole\_Population\_Manager | `[inline]` |
| SupplyHowManyVoles(unsigned p\_x, unsigned p\_y, unsigned p\_size) | Vole\_Population\_Manager |  |
| SupplyInOlderTerr(unsigned p\_x, unsigned p\_y, unsigned p\_Age, unsigned p\_Range) | Vole\_Population\_Manager |  |
| SupplyListIndexSize() | Population\_Manager | `[inline]` |
| SupplyListName(int i) | Population\_Manager | `[inline]` |
| SupplyListNameLength() | Population\_Manager | `[inline]` |
| SupplyListSize(unsigned listindex) | Population\_Manager | `[inline]` |
| SupplyLocXY(unsigned listindex, unsigned j, int &x, int &y) | Population\_Manager | `[inline, virtual]` |
| SupplyOlderFemales(unsigned p\_x, unsigned p\_y, unsigned p\_Age, unsigned p\_range) | Vole\_Population\_Manager |  |
| SupplyPegPosx(int) | Population\_Manager | `[inline, virtual]` |
| SupplyPegPosy(int) | Population\_Manager | `[inline, virtual]` |
| SupplySimH() | Population\_Manager | `[inline]` |
| SupplySimW() | Population\_Manager | `[inline]` |
| SupplyState(unsigned listindex, unsigned j) | Population\_Manager | `[inline]` |
| SupplyStateList() | Population\_Manager | `[inline]` |
| SupplyStateNames(int i) | Population\_Manager | `[inline]` |
| SupplyStateNamesLength() | Population\_Manager | `[inline]` |
| SupplyStepSize() | Population\_Manager | `[inline]` |
| SupplyVoleList(unsigned x, unsigned y, unsigned range) | Vole\_Population\_Manager |  |
| TestFile | Vole\_Population\_Manager |  |
| TestFile2 | Vole\_Population\_Manager |  |
| TheArray | Population\_Manager | `[protected]` |
| TheBreedingFemalesProbe(int) | Population\_Manager | `[inline, virtual]` |
| TheBreedingSuccessProbe(int &, int &, int &, int &, int &, int &) | Population\_Manager | `[inline, virtual]` |
| TheCIPEGridOutputProbe() | Vole\_Population\_Manager | `[virtual]` |
| TheCIPEGridOutputProbeB() | Population\_Manager | `[inline, virtual]` |
| TheFledgelingProbe() | Population\_Manager | `[inline, virtual]` |
| TheGeneticProbe(unsigned, int, unsigned &) | Population\_Manager | `[inline, virtual]` |
| TheProbe | Population\_Manager |  |
| TheReallyBigOutputProbe() | Vole\_Population\_Manager | `[virtual]` |
| TheRipleysOutputProbe(FILE \*a\_prb) | Vole\_Population\_Manager | `[virtual]` |
| thisYear | Vole\_Population\_Manager | `[protected]` |
| Vole\_Population\_Manager(Landscape \*p\_L) | Vole\_Population\_Manager |  |
| VoleMap | Vole\_Population\_Manager |  |
| WriteToTest2File(int n, int n2) | Population\_Manager | `[inline]` |
| WriteToTest2File(char \*n, int n2) | Population\_Manager | `[inline]` |
| WriteToTestFile(int n, int n2) | Population\_Manager | `[inline]` |
| WriteToTestFile(char \*n, int n2) | Population\_Manager | `[inline]` |
| YearsTotal | Vole\_Population\_Manager | `[protected]` |
| YoungProducedToday | Vole\_Population\_Manager |  |
| YoungsFile | Vole\_Population\_Manager |  |
| ~Population\_Manager(void) | Population\_Manager | `[virtual]` |
| ~Vole\_Population\_Manager() | Vole\_Population\_Manager |  |

---

Generated on Thu Jan 22 14:13:48 2009 for ALMaSS ODDox by 
 1.5.6 
