# Supplementary material for: Predation and fragmentation portrayed in the statistical structure of prey time series
Source: BMC Ecol. 2009 May 6;9:10. doi: 10.1186/1472-6785-9-10 (PMC2689204; doi:10.1186/1472-6785-9-10)
Supplement: Additional file 2 — Voles and related classes ODDox Documentation. ODDox documentation of the agent-based model (ALMaSS) applied by Hendrichsen et al. The documentation is started by activating main.html. [file 1472-6785-9-10-S2.zip › Vole_ODDox/class_vole___population___manager.html]

ALMaSS ODDox: Vole\_Population\_Manager Class Reference

- Main Page
- Related Pages
- Classes
- Files

- Alphabetical List
- Class List
- Class Hierarchy
- Class Members

# Vole\_Population\_Manager Class Reference

`#include <VolePopulationManager.H>`

Inheritance diagram for Vole\_Population\_Manager:

List of all members.

---

## Detailed Description

The class to handle all vole population related matters.

|  |
| --- |
|  |
| Public Member Functions | |
| void | AddToGeneticImpacted () |
| void | AddToImpacted () |
| void | AddToJuvs (int juvs) |
| void | AddToNotImpacted () |
| void | AddToYoung (int young) |
| void | CreateObjects (int ob\_type, TAnimal \*pvo, struct\_Vole\_Adult \*data, int number) |
| void | CreateObjects\_Init (int ob\_type, TAnimal \*pvo, struct\_Vole\_Adult \*data, int number) |
| Vole\_Female \* | FindClosestFemale (int p\_x, int p\_y, int p\_steps) |
| Vole\_Male \* | FindClosestMale (int p\_x, int p\_y, int p\_steps) |
| Vole\_Male \* | FindRandomMale () |
| virtual void | GeneticsResultsOutput (FILE \*ofile, unsigned listindex) |
| virtual void | ImpactedProbe () |
| virtual void | Init (void) |
| bool | InSquare (int p\_x, int p\_y, int p\_sqx, int p\_sqy, int p\_range) |
| int | ListClosestFemales (int p\_x, int p\_y, int p\_steps) |
| int | ListClosestMales (int p\_x, int p\_y, int p\_steps) |
| void | ReproductionProbe () |
| void | SendMessage (TTypeOfVoleMessage p\_message, unsigned p\_x, unsigned p\_y, unsigned p\_range, unsigned p\_age, bool p\_sex) |
| int | SupplyGrowthStartDate () |
| int | SupplyHowManyVoles (unsigned p\_x, unsigned p\_y, unsigned p\_size) |
| int | SupplyInOlderTerr (unsigned p\_x, unsigned p\_y, unsigned p\_Age, unsigned p\_Range) |
| bool | SupplyOlderFemales (unsigned p\_x, unsigned p\_y, unsigned p\_Age, unsigned p\_range) |
| vector< Vole\_Base \* > \* | SupplyVoleList (unsigned x, unsigned y, unsigned range) |
| virtual void | TheCIPEGridOutputProbe () |
| virtual void | TheReallyBigOutputProbe () |
| virtual void | TheRipleysOutputProbe (FILE \*a\_prb) |
|  | Vole\_Population\_Manager (Landscape \*p\_L) |
|  | ~Vole\_Population\_Manager () |
| Public Attributes | |
| AlleleFreq \* | AlFreq |
| Vole\_FemaleList | FList |
| unsigned | IDNumber |
| int | JuvsProducedToday |
| int | m\_geneticproductfertilityeffect |
| Vole\_MaleList | MList |
| int | ReproTable [4][12] |
| FILE \* | TestFile |
| FILE \* | TestFile2 |
| IDMap \* | VoleMap |
| int | YoungProducedToday |
| FILE \* | YoungsFile |
| Protected Member Functions | |
| virtual void | Catastrophe () |
| void | DoFirst () |
| Protected Attributes | |
| int | m\_f1sterilitychance |
| int | m\_geneticimpacted |
| int | m\_geneticsterilitychance |
| int | m\_GrowthStartDate |
| int | m\_impacted |
| int | m\_notimpacted |
| int | thisYear |
| int | YearsTotal |

---

## Constructor & Destructor Documentation

|  |  |  |  |  |  |
| --- | --- | --- | --- | --- | --- |
| Vole\_Population\_Manager::Vole\_Population\_Manager | ( | Landscape \* | *p\_L* | ) |  |

Class for managing lists of all voles in the simulation and responsible for generating output. Main functionality is the the base class Population\_Manager

References AlFreq, IDNumber, Init(), m\_GrowthStartDate, Population\_Manager::TheArray, thisYear, VoleMap, and YearsTotal.

```
00095    : Population_Manager(p_L)
00096 {
00097   // two lists are needed so need to remove 8 of the ten default arrays
00098   // Males,Females
00099   for (int i=0; i<8; i++)
00100   {
00101     TheArray.pop_back();
00102   }
00103   IDNumber=0;
00104   thisYear=0;
00105   YearsTotal=0;
00106   VoleMap = new IDMap(p_L);
00107   AlFreq = new AlleleFreq();
00108   m_GrowthStartDate=9000; // delays this until we know the real one
00109   Init();
00110 }
```

|  |  |  |  |  |
| --- | --- | --- | --- | --- |
| Vole\_Population\_Manager::~Vole\_Population\_Manager | ( |  | ) |  |

Vole\_Population\_Manager destructor   
Only needed to close any potentially open output files

References AlFreq, Population\_Manager::m\_AlleleFreqsFile, Population\_Manager::m\_EasyPopRes, Population\_Manager::m\_GeneticsFile, TestFile, TestFile2, and VoleMap.

```
00118 {
00119   // Save the impacted file
00120   //errno_t errNum;
00121         // Output files for the vole
00122         fclose( m_GeneticsFile );
00123         fclose( m_AlleleFreqsFile );
00124         fclose( m_EasyPopRes );
00125   // Uncomment below if test files are needed
00126   fclose( TestFile );
00127   fclose( TestFile2 );
00128   delete VoleMap;
00129   delete AlFreq;
00130 }
```

---

## Member Function Documentation

|  |  |  |  |  |
| --- | --- | --- | --- | --- |
| void Vole\_Population\_Manager::AddToGeneticImpacted | ( |  | ) | `[inline]` |

References m\_geneticimpacted.

Referenced by Vole\_Female::st\_Lactating().

```
00073 {m_geneticimpacted++;}
```

|  |  |  |  |  |
| --- | --- | --- | --- | --- |
| void Vole\_Population\_Manager::AddToImpacted | ( |  | ) | `[inline]` |

References m\_impacted.

Referenced by Vole\_Female::st\_Lactating().

```
00071 {m_impacted++;}
```

|  |  |  |  |  |  |
| --- | --- | --- | --- | --- | --- |
| void Vole\_Population\_Manager::AddToJuvs | ( | int | *juvs* | ) | `[inline]` |

References JuvsProducedToday.

Referenced by Vole\_Female::st\_Lactating().

```
00106 {JuvsProducedToday+=juvs;}
```

|  |  |  |  |  |
| --- | --- | --- | --- | --- |
| void Vole\_Population\_Manager::AddToNotImpacted | ( |  | ) | `[inline]` |

References m\_notimpacted.

Referenced by Vole\_Female::st\_Lactating().

```
00072 {m_notimpacted++;}
```

|  |  |  |  |  |  |
| --- | --- | --- | --- | --- | --- |
| void Vole\_Population\_Manager::AddToYoung | ( | int | *young* | ) | `[inline]` |

References YoungProducedToday.

Referenced by Vole\_Female::st\_GiveBirth().

```
00103                               {
00104            YoungProducedToday+=young;
00105    }
```

|  |  |  |  |  |  |
| --- | --- | --- | --- | --- | --- |
| void Vole\_Population\_Manager::Catastrophe | ( | void |  | ) | `[protected, virtual]` |

This version simply alters populations after 1st January - it is very dangerous to add individuals in many of the models so beware!!!!

Reimplemented from Population\_Manager.

References cfg\_pm\_eventfrequency, cfg\_pm\_eventsize, Vole\_Base::CopyMyself(), Vole\_Base::CurrentVState, Population\_Manager::m\_catastrophestartyear, Population\_Manager::m\_TheLandscape, Population\_Manager::TheArray, tovs\_FDying, and tovs\_MDying.

```
01915                                                 {
01919         // First do we have the right day?
01920         int today = m_TheLandscape->SupplyDayInYear();
01921         if (today!=1) return;
01922         // First do we have the right year?
01923         int year = m_TheLandscape->SupplyYearNumber()-m_catastrophestartyear;
01924         if (year%cfg_pm_eventfrequency.value()!=0) return;
01925 
01926         Vole_Male* MV = NULL; // assignment to get rid of warning
01927         Vole_Female* FV = NULL;
01928         Vole_Base* VB = NULL;
01929         // Now if the % decrease is higher or lower than 100 we need to do different things
01930         int esize=cfg_pm_eventsize.value();
01931         if (esize<100) {
01932                 unsigned size2 = (unsigned) TheArray[ 0 ].size();
01933                         for ( unsigned j = 0; j < size2; j++ ) {
01934                                 if (random(100) > esize) {
01935                         MV = dynamic_cast < Vole_Male * > ( TheArray[ 0 ] [ j ] );
01936                                             MV->CurrentVState=tovs_MDying; // Kill it
01937                                 }
01938                                 }
01939                         size2 = (unsigned) TheArray[ 1 ].size();
01940                         for ( unsigned j = 0; j < size2; j++ ) {
01941                                 if (random(100) > esize) {
01942                         FV = dynamic_cast < Vole_Female * > ( TheArray[ 1 ] [ j ] );
01943                                             FV->CurrentVState=tovs_FDying;; // Kill it 
01944                                 }
01945                                 }
01946                 }
01947         else if (esize>100) {
01948                 // This is a tricky thing to do because we need to duplicate voles, but dare not mess 
01949                 // mate pointers etc up.
01950                 // This also requires a copy method in the target vole
01951                 // esize also needs translating  120 = 20%, 200 = 100% 
01952                 if (esize<200) {
01953                         esize-=100;
01954                         for (unsigned i=0; i<1; i++) {
01955                         unsigned size2;
01956                         size2 = (unsigned) TheArray[ i ].size();
01957                         for ( unsigned j = 0; j < size2; j++ ) {
01958                                 if (random(100) < esize) {
01959                         VB = dynamic_cast < Vole_Base * > ( TheArray[ i ] [ j ] );
01960                                             VB->CopyMyself(i); // Duplicate it
01961                                         }
01962                                 }
01963                                 }
01964                 } else {
01965                         esize-=100;
01966                         esize/=100; // this will throw away fractional parts so will get 1, 2, 3  from 200, 350 400
01967                         for (unsigned i=0; i<2; i++) {
01968                                 unsigned size2;
01969                                 size2 = (unsigned) TheArray[ i ].size();
01970                                 for ( unsigned j = 0; j < size2; j++ ) {
01971                                         for ( int e=0; e<esize; e++) {
01972                         VB = dynamic_cast < Vole_Base * > ( TheArray[ i ] [ j ] );
01973                                             VB->CopyMyself(i); // Duplicate it
01974                                                 }
01975                                 }
01976                         }
01977                 }
01978                 }
01979         else return; // No change so do nothing
01980 }
```

|  |  |  |  |
| --- | --- | --- | --- |
| void Vole\_Population\_Manager::CreateObjects | ( | int | *ob\_type*, |
|  |  | TAnimal \* | *pvo*, |
|  |  | struct\_Vole\_Adult \* | *as*, |
|  |  | int | *number* |  |
|  | ) |  |  |  |

Creates 'number' of vole objects of the type ob\_type using 'as' for the base data

References struct\_Vole\_Adult::Genes, GeneticMaterial::GetAllele(), struct\_Vole\_Adult::L, struct\_Vole\_Adult::m\_dflag, m\_f1sterilitychance, m\_geneticsterilitychance, struct\_Vole\_Adult::m\_gflag, Vole\_Base::m\_pesticideInfluenced2, Vole\_Base::SetDirectFlag(), Vole\_Male::SetFertile(), Vole\_Base::SetGeneticFlag(), Population\_Manager::TheArray, Vole\_Base::UnsetDirectFlag(), Vole\_Base::UnsetGeneticFlag(), VoleMap, struct\_Vole\_Adult::VPM, struct\_Vole\_Adult::x, and struct\_Vole\_Adult::y.

Referenced by Vole\_Base::CopyMyself(), and Vole\_Female::st\_Lactating().

```
01822 {
01823    Vole_Male*  new_Male;
01824    Vole_Female*  new_Female;
01825 
01826 
01827    for (int i=0; i<number; i++)
01828    {
01829     if (ob_type == 0)
01830     {
01831        #ifdef __MORT1__
01832               if (as->Genes.GetAllele(0,0) == 1) return;  // Kill the male rather than make him infertile
01833        #endif
01834        new_Male = new Vole_Male(as->x, as->y, as->L,as->Genes, as->VPM);
01835 #ifdef __SpecificPesticideEffectsVinclozolinLike__
01836            if (as->m_dflag) {  // Chromo 1. direct effect
01837                    new_Male->SetDirectFlag();                      
01838                    new_Male->SetGeneticFlag();
01839                    if (random(100)>=m_f1sterilitychance) new_Male->SetFertile(0); 
01840                    else new_Male->SetFertile(-1); //
01841                    new_Male->m_pesticideInfluenced2=true;
01842            }
01843            else {
01844                    if (as->m_gflag) {  // Chromo 0, genetic effect
01845                            new_Male->UnsetDirectFlag();
01846                            new_Male->SetGeneticFlag();
01847                            if (random(100) >=m_geneticsterilitychance) new_Male->SetFertile(0); //100 means all are sterile, 0 means none are
01848                            else new_Male->SetFertile(-1); 
01849                            new_Male->m_pesticideInfluenced2=true;
01850                    }
01851                    else {
01852                                 new_Male->UnsetGeneticFlag();
01853                                 new_Male->UnsetDirectFlag();
01854                                 new_Male->SetFertile(1);
01855                    }
01856 // DEBUG
01857 //                 if (new_Male->GetGeneticFlag()) {
01858 //                         int rubbish=0;
01859 //                 }
01860            }
01861 #else
01862            new_Male->SetFertile(1);
01863 #endif
01864            TheArray[ob_type].push_back(new_Male);
01865        VoleMap->SetMapValue(as->x,as->y,(TAnimal*)new_Male);
01866     }
01867     if (ob_type == 1)
01868     {
01869        new_Female = new Vole_Female(as->x, as->y, as->L,as->Genes, as->VPM);
01870        TheArray[ob_type].push_back(new_Female);
01871        VoleMap->SetMapValue(as->x,as->y,(TAnimal*)new_Female);
01872     }
01873    }
01874 }
```

|  |  |  |  |
| --- | --- | --- | --- |
| void Vole\_Population\_Manager::CreateObjects\_Init | ( | int | *ob\_type*, |
|  |  | TAnimal \* | *pvo*, |
|  |  | struct\_Vole\_Adult \* | *data*, |
|  |  | int | *number* |  |
|  | ) |  |  |  |

Creates 'number' of vole objects of the type ob\_type using 'as' for the base data for use at the beginning of a simulation.   
A number of the attributes are set at defaults or randomised

References struct\_Vole\_Adult::Genes, struct\_Vole\_Adult::L, Vole\_Base::Set\_Age(), Vole\_Male::SetFertile(), Vole\_Base::Setm\_Mature(), Vole\_Base::SetWeight(), Population\_Manager::TheArray, Vole\_Base::UnsetDirectFlag(), Vole\_Base::UnsetGeneticFlag(), struct\_Vole\_Adult::VPM, struct\_Vole\_Adult::x, and struct\_Vole\_Adult::y.

Referenced by Init().

```
01883 {
01884    struct_Vole_Adult *as=NULL;
01885 
01886    for (int i=0; i<number; i++)
01887    {
01888     if (ob_type == 0)
01889     {
01890        Vole_Male*  new_Male;
01891        as=dynamic_cast<struct_Vole_Adult *>(data);
01892        new_Male = new Vole_Male(as->x, as->y, as->L,as->Genes, as->VPM);
01893        TheArray[ob_type].push_back(new_Male);
01894        new_Male->SetWeight(40.0);
01895        new_Male->Setm_Mature();
01896        new_Male->Set_Age(random(500));
01897         new_Male->UnsetGeneticFlag();
01898                 new_Male->UnsetDirectFlag();
01899                 new_Male->SetFertile(1);
01900     }
01901     if (ob_type == 1)
01902     {
01903        Vole_Female*  new_Female;
01904        as=dynamic_cast<struct_Vole_Adult *>(data);
01905        new_Female = new Vole_Female(as->x, as->y, as->L,as->Genes, as->VPM);
01906        TheArray[ob_type].push_back(new_Female);
01907        new_Female->SetWeight(40.0);
01908        new_Female->Setm_Mature();
01909        new_Female->Set_Age(random(500));
01910     }
01911    }
01912 }
```

|  |  |  |  |  |
| --- | --- | --- | --- | --- |
| void Vole\_Population\_Manager::DoFirst | ( |  | ) | `[protected, virtual]` |

This method is called before the BeginStep of the main program loop by the base class.   
It controls when the grass starts to grow and therefore triggers start of reproduction but the primary role is as a safe location where outputs can be generated. At the time this is called there no chance that the voles are engaged in any kind of activity.

Reimplemented from Population\_Manager.

References cfg\_GrassStartGrowth(), JuvsProducedToday, Population\_Manager::LamdaClear(), Population\_Manager::LamdaDumpOutput(), m\_geneticimpacted, m\_GrowthStartDate, m\_impacted, m\_notimpacted, Population\_Manager::m\_TheLandscape, AnimalPosition::m\_x, AnimalPosition::m\_y, Vole\_Female::OnKilled(), Vole\_Male::OnKilled(), TAnimal::SupplyPosition(), Population\_Manager::TheArray, thisYear, YearsTotal, and YoungProducedToday.

```
00308 {
00309   int today=m_TheLandscape->SupplyDayInYear();
00310   if (today==0) m_GrowthStartDate=9000;
00311   if ((today>59)&& (m_GrowthStartDate==9000))
00312   {
00313     if (m_TheLandscape->SupplyMeanTemp(m_TheLandscape->SupplyGlobalDate()-7,7)
00314                                                  > cfg_GrassStartGrowth.value())
00315     {
00316       m_GrowthStartDate=-today;
00317       printf("The start day is: %d\n",today);
00318     }
00319   }
00320 
00321   YoungProducedToday=0; // Zero the number of young
00322   JuvsProducedToday=0; // Zero the number of juveniles
00323   if (today==0) {
00324           // Save the impacted file
00325           FILE* impf = fopen("Impacted.res","a");
00326           if (!impf) {
00327                   m_TheLandscape->Warn("Vole_Population_Manager Destructor","Could Not Open Impacted.Res File");
00328                   exit(0);
00329           }
00330           fprintf(impf,"%d\t%d\t%d\n",m_impacted, m_notimpacted, m_geneticimpacted);
00331           fclose(impf);
00332           m_impacted=0;
00333           m_notimpacted=0;
00334           m_geneticimpacted=0;
00335   }
00336 
00337 
00338 #ifdef __VOLE_SETAC_PESTICIDE_ON
00339   int now=TheLandscape->SupplyDayInYear();
00340 /*
00341   if ((now>0)&&(now<365))//((now==165) ||(now==180)||(now==195))
00342   {
00343     // Kill all voles in field boundaries
00344     // Uses SubSize to return the population size for each call
00345     unsigned size=TheArray[0].size();
00346     AnimalPosition AP;
00347     if (size>0)
00348     {
00349       Vole_Male* MV;
00350       for (unsigned j=0; j<size; j++)
00351       {
00352         MV=dynamic_cast<Vole_Male*>(TheArray[0][j]);
00353         AP=MV->SupplyPosition();
00354         TTypesOfLandscapeElement tole =TheLandscape->SupplyElementType(AP.m_x,AP.m_y);
00355         if ((tole!=tole_NaturalGrass)) MV->OnKilled();
00356       }
00357     }
00358     size=TheArray[1].size();
00359     if (size>0)
00360     {
00361       Vole_Female* FV;
00362       for (unsigned j=0; j<size; j++)
00363       {
00364         FV=dynamic_cast<Vole_Female*>(TheArray[1][j]);
00365         AP=FV->SupplyPosition();
00366         TTypesOfLandscapeElement tole =TheLandscape->SupplyElementType(AP.m_x,AP.m_y);
00367         if ((tole!=tole_NaturalGrass)) FV->OnKilled();
00368       }
00369     }
00370   }
00371 */
00372   if (now==150)
00373   {
00374    if (YearsTotal++>500)
00375   if (thisYear++==5)
00376   {
00377 
00378     thisYear=0;
00379     // Kill all voles in field boundaries
00380     // Uses SubSize to return the population size for each call
00381     unsigned size=TheArray[0].size();
00382     AnimalPosition AP;
00383     const int xpos[2][4] = {{0,1000,2000,0},{1001,2001,3001,10000}};
00384     int index1=random(3);
00385     index1=3;
00386     if (size>0)
00387     {
00388       Vole_Male* MV;
00389       for (unsigned j=0; j<size; j++)
00390       {
00391         MV=dynamic_cast<Vole_Male*>(TheArray[0][j]);
00392         AP=MV->SupplyPosition();
00393         TTypesOfLandscapeElement tole =TheLandscape->SupplyElementType(AP.m_x,AP.m_y);
00394         if ((AP.m_x>xpos[0][index1]) &&(AP.m_x<xpos[1][index1]))
00395 //        if ((tole==tole_NaturalGrass)&& (random(100)>-1)) MV->OnKilled();
00396         if ((random(100)>4)) MV->OnKilled();
00397       }
00398     }
00399     size=TheArray[1].size();
00400     if (size>0)
00401     {
00402       Vole_Female* FV;
00403       for (unsigned j=0; j<size; j++)
00404       {
00405         FV=dynamic_cast<Vole_Female*>(TheArray[1][j]);
00406         AP=FV->SupplyPosition();
00407         TTypesOfLandscapeElement tole =TheLandscape->SupplyElementType(AP.m_x,AP.m_y);
00408         if ((AP.m_x>xpos[0][index1]) &&(AP.m_x<xpos[1][index1]))
00409         //if ((tole==tole_NaturalGrass)&& (random(100)>-1)) FV->OnKilled();
00410         if ((random(100)>4)) FV->OnKilled();
00411       }
00412     }
00413   }
00414   }
00415 #endif
00416 #ifdef __LAMBDA_RECORD
00417   if (today==March) {
00418           LamdaDumpOutput();
00419           LamdaClear();
00420   }
00421 #endif
00422 
00423 }
```

|  |  |  |  |
| --- | --- | --- | --- |
| Vole\_Female \* Vole\_Population\_Manager::FindClosestFemale | ( | int | *p\_x*, |
|  |  | int | *p\_y*, |
|  |  | int | *p\_steps* |  |
|  | ) |  |  |  |

looks for the closest female within p\_Steps of p\_x,p\_y

References Population\_Manager::SimH, Population\_Manager::SimW, Vole\_Base::SupplySex(), Vole\_Base::SupplyTerritorial(), and VoleMap.

```
01045 {
01047   // as usual there are 4 possibilities of overlap
01048   // First convert centre co-rdinates to square co-ordinates
01049 
01050   int x=p_x - p_steps;
01051   if (x<0) x+=SimW;  // ensure we start in the simulation area!
01052   int y=p_y - p_steps;
01053   if (y<0) y+=SimH;
01054   int range_x=p_steps*2;
01055   int range_y=p_steps*2;
01056 
01057   // create the extent variables
01058   int xextent0 = x+range_x;
01059   int yextent0 = y+range_y;
01060   int xextent1 = (x+range_x)-SimW;
01061   int yextent1 = (y+range_y)-SimH;
01062   // Create the looping variables needed
01063   // Create the looping variables needed
01064   int Dfinx;
01065   int Dfiny;
01066   int Afinx=0;  //unless the finx values for A are changed this stop
01067   int Afiny=0;  //the loop from executing
01068   int Asty=0;
01069   // int Astx,Dstx,Dtsy are always default so variables not used from them
01070   // NB Astx, Asty and Dstx are always 0, 0 & x respectively
01071   // Dsty is always y, Afiny is always yextent1 if it is used
01072   // Now create the loop values;
01073   if (xextent0<=SimW)   // No overlap with the eastern side
01074   {
01075     // Dstx, Dsty, Asty set by defaults
01076     //Astx & Afinx are not needed
01077     Dfinx=xextent0;
01078     // do we overlap the bottom?
01079     // Type B & D (overlap bottom, no overlap)
01080     if (yextent0>SimH)
01081     {
01082       // Type B (overlap bottom only)
01083       Dfiny=SimH;  // stop at the end
01084       Afiny=yextent1; // the overlap with the top
01085     }
01086     else Dfiny=yextent0;
01087   }
01088   else
01089   {
01090     // Type A & C overlap bottom & eastern edgdes
01091     if (yextent0>SimH)
01092     {
01093       // relies on the default start for Asty, Astx, Dstx, Dsty
01094       Afinx=xextent1;
01095       Afiny=yextent1;
01096       Dfinx=SimW;  // Stop at the end
01097       Dfiny=SimH;
01098     }
01099     else
01100     {
01101       // Type C overlap left edge only
01102       // Astx & Afiny are not needed here
01103       //Astx, Dstx, Dsty set by default
01104       Afinx=xextent1;
01105       Dfinx=SimW;  // Stop at the end
01106       Dfiny=yextent0;
01107     }
01108   }
01109   Vole_Female* AFemale;
01110   Vole_Female* Found=NULL;
01111   int dist, disty;
01112   int FoundDist=SimW; // too big
01113   Vole_Base* Ap;
01114 
01115     // A Loop
01116     for (int i=0; i<Afinx; i++)
01117     {
01118       for (int j=Asty; j<Afiny; j++)
01119       {
01120         Ap=(Vole_Base*) (VoleMap->GetMapValue(i,j)) ;
01121         if (Ap)
01122         {
01123           if (!Ap->SupplySex()) // female
01124           {
01125             AFemale=(Vole_Female*) (Ap) ;
01126             if (AFemale->SupplyTerritorial())
01127             {
01128               dist=abs(p_x-i); // remove the signed bit
01129               disty=abs(p_y-j);
01130               dist+=disty;
01131               if (dist<FoundDist)
01132               {
01133                 Found=AFemale;
01134                 FoundDist=dist;
01135               }
01136             }
01137           }
01138         }
01139       }
01140       // C Loop
01141       for (int j=y; j<Dfiny; j++)
01142       {
01143         Ap=(Vole_Base*) (VoleMap->GetMapValue(i,j)) ;
01144         if (Ap)
01145         {
01146           if (!Ap->SupplySex()) // female
01147           {
01148             AFemale=(Vole_Female*) (Ap) ;
01149             if (AFemale->SupplyTerritorial())
01150             {
01151               dist=abs(p_x-i); // remove the signed bit
01152               disty=abs(p_y-j);
01153               dist+=disty;
01154               if (dist<FoundDist)
01155               {
01156                 Found=AFemale;
01157                 FoundDist=dist;
01158               }
01159             }
01160           }
01161         }
01162       }
01163     }
01164     // D Loop
01165     for (int i=x; i<Dfinx; i++)
01166     {
01167       for (int j=y; j<Dfiny; j++)
01168       {
01169         Ap=(Vole_Base*) (VoleMap->GetMapValue(i,j)) ;
01170         if (Ap)
01171         {
01172           if (!Ap->SupplySex()) // female
01173           {
01174             AFemale=(Vole_Female*) (Ap) ;
01175             if (AFemale->SupplyTerritorial())
01176             {
01177               dist=abs(p_x-i); // remove the signed bit
01178               disty=abs(p_y-j);
01179               dist+=disty;
01180               if (dist<FoundDist)
01181               {
01182                 Found=AFemale;
01183                 FoundDist=dist;
01184               }
01185             }
01186           }
01187         }
01188       }
01189       // B Loop
01190       for (int j=0; j<Afiny; j++)
01191       {
01192         Ap=(Vole_Base*) (VoleMap->GetMapValue(i,j)) ;
01193         if (Ap)
01194         {
01195           if (!Ap->SupplySex()) // female
01196           {
01197             AFemale=(Vole_Female*) (Ap) ;
01198             if (AFemale->SupplyTerritorial())
01199             {
01200               dist=abs(p_x-i); // remove the signed bit
01201               disty=abs(p_y-j);
01202               dist+=disty;
01203               if (dist<FoundDist)
01204               {
01205                 Found=AFemale;
01206                 FoundDist=dist;
01207               }
01208             }
01209           }
01210         }
01211       }
01212     }
01213   return Found;
01214 }
```

|  |  |  |  |
| --- | --- | --- | --- |
| Vole\_Male \* Vole\_Population\_Manager::FindClosestMale | ( | int | *p\_x*, |
|  |  | int | *p\_y*, |
|  |  | int | *p\_steps* |  |
|  | ) |  |  |  |

looks for the closest male within p\_Steps of p\_x,p\_y

References Population\_Manager::SimH, Population\_Manager::SimW, Vole\_Base::SupplySex(), Vole\_Base::SupplyTerritorial(), and VoleMap.

```
01218 {
01220   // as usual there are 4 possibilities of overlap
01221   // First convert centre co-rdinates to square co-ordinates
01222 
01223   int x=p_x - p_steps;
01224   if (x<0) x+=SimW;  // ensure we start in the simulation area!
01225   int y=p_y - p_steps;
01226   if (y<0) y+=SimH;
01227   int range_x=p_steps*2;
01228   int range_y=p_steps*2;
01229 
01230   // create the extent variables
01231   int xextent0 = x+range_x;
01232   int yextent0 = y+range_y;
01233   int xextent1 = (x+range_x)-SimW;
01234   int yextent1 = (y+range_y)-SimH;
01235   // Create the looping variables needed
01236   // Create the looping variables needed
01237   int Dfinx;
01238   int Dfiny;
01239   int Afinx=0;  //unless the finx values for A are changed this stop
01240   int Afiny=0;  //the loop from executing
01241   int Asty=0;
01242   // int Astx,Dstx,Dtsy are always default so variables not used from them
01243   // NB Astx, Asty and Dstx are always 0, 0 & x respectively
01244   // Dsty is always y, Afiny is always yextent1 if it is used
01245   // Now create the loop values;
01246   if (xextent0<=SimW)   // No overlap with the eastern side
01247   {
01248     // Dstx, Dsty, Asty set by defaults
01249     //Astx & Afinx are not needed
01250     Dfinx=xextent0;
01251     // do we overlap the bottom?
01252     // Type B & D (overlap bottom, no overlap)
01253     if (yextent0>SimH)
01254     {
01255       // Type B (overlap bottom only)
01256       Dfiny=SimH;  // stop at the end
01257       Afiny=yextent1; // the overlap with the top
01258     }
01259     else Dfiny=yextent0;
01260   }
01261   else
01262   {
01263     // Type A & C overlap bottom & eastern edgdes
01264     if (yextent0>SimH)
01265     {
01266       // relies on the default start for Asty, Astx, Dstx, Dsty
01267       Afinx=xextent1;
01268       Afiny=yextent1;
01269       Dfinx=SimW;  // Stop at the end
01270       Dfiny=SimH;
01271     }
01272     else
01273     {
01274       // Type C overlap left edge only
01275       // Astx & Afiny are not needed here
01276       //Astx, Dstx, Dsty set by default
01277       Afinx=xextent1;
01278       Dfinx=SimW;  // Stop at the end
01279       Dfiny=yextent0;
01280     }
01281   }
01282   Vole_Male* AMale;
01283   Vole_Male* Found=NULL;
01284   int dist, disty;
01285   int FoundDist=SimW; // too big
01286   Vole_Base* Ap;
01287 
01288     // A Loop
01289     for (int i=0; i<Afinx; i++)
01290     {
01291       for (int j=Asty; j<Afiny; j++)
01292       {
01293         Ap=(Vole_Base*) (VoleMap->GetMapValue(i,j)) ;
01294         if (Ap)
01295         {
01296           if (Ap->SupplySex()) // male
01297           {
01298             AMale=(Vole_Male*) (Ap) ;
01299             if (AMale->SupplyTerritorial())
01300             {
01301               dist=abs(p_x-i); // remove the signed bit
01302               disty=abs(p_y-j);
01303               dist+=disty;
01304               if (dist<FoundDist)
01305               {
01306                 Found=AMale;
01307                 FoundDist=dist;
01308               }
01309             }
01310           }
01311         }
01312       }
01313       // C Loop
01314       for (int j=y; j<Dfiny; j++)
01315       {
01316         Ap=(Vole_Base*) (VoleMap->GetMapValue(i,j)) ;
01317         if (Ap)
01318         {
01319           if (Ap->SupplySex()) // male
01320           {
01321             AMale=(Vole_Male*) (Ap) ;
01322             if (AMale->SupplyTerritorial())
01323             {
01324               dist=abs(p_x-i); // remove the signed bit
01325               disty=abs(p_y-j);
01326               dist+=disty;
01327               if (dist<FoundDist)
01328               {
01329                 Found=AMale;
01330                 FoundDist=dist;
01331               }
01332             }
01333           }
01334         }
01335       }
01336     }
01337     // D Loop
01338     for (int i=x; i<Dfinx; i++)
01339     {
01340       for (int j=y; j<Dfiny; j++)
01341       {
01342         Ap=(Vole_Base*) (VoleMap->GetMapValue(i,j)) ;
01343         if (Ap)
01344         {
01345           if (Ap->SupplySex()) // male
01346           {
01347             AMale=(Vole_Male*) (Ap) ;
01348             if (AMale->SupplyTerritorial())
01349             {
01350               dist=abs(p_x-i); // remove the signed bit
01351               disty=abs(p_y-j);
01352               dist+=disty;
01353               if (dist<FoundDist)
01354               {
01355                 Found=AMale;
01356                 FoundDist=dist;
01357               }
01358             }
01359           }
01360         }
01361       }
01362       // B Loop
01363       for (int j=0; j<Afiny; j++)
01364       {
01365         Ap=(Vole_Base*) (VoleMap->GetMapValue(i,j)) ;
01366         if (Ap)
01367         {
01368           if (Ap->SupplySex()) // male
01369           {
01370             AMale=(Vole_Male*) (Ap) ;
01371             if (AMale->SupplyTerritorial())
01372             {
01373               dist=abs(p_x-i); // remove the signed bit
01374               disty=abs(p_y-j);
01375               dist+=disty;
01376               if (dist<FoundDist)
01377               {
01378                 Found=AMale;
01379                 FoundDist=dist;
01380               }
01381             }
01382           }
01383         }
01384       }
01385     }
01386   return Found;
01387 }
```

|  |  |  |  |  |
| --- | --- | --- | --- | --- |
| Vole\_Male \* Vole\_Population\_Manager::FindRandomMale | ( |  | ) |  |

Returns a pointer to a random male - he does not even have to have a territory

References TALMaSSObject::CurrentStateNo, and Population\_Manager::TheArray.

Referenced by Vole\_Female::st\_Mating().

```
01391 {
01394   Vole_Male* Found=NULL;
01395   TAnimal* Ap;
01396   int size=(int) TheArray[0].size();
01397   do
01398   {
01399     int test =random(size);
01400     Ap=TheArray[0][test];
01401     Found=(Vole_Male*)(Ap);
01402     if (Found->CurrentStateNo==-1) Found=NULL;
01403   } while ((Found==NULL) && (size>0));
01404   return Found;
01405 }
```

|  |  |  |  |
| --- | --- | --- | --- |
| void Vole\_Population\_Manager::GeneticsResultsOutput | ( | FILE \* | *ofile*, |
|  |  | unsigned | *listindex* |  |
|  | ) |  |  | `[virtual]` |

Output rectording the genetic information for each individual in the population of listindex type

Reimplemented from Population\_Manager.

References Population\_Manager::m\_TheLandscape, TAnimal::Supply\_m\_Location\_x(), Vole\_Base::SupplyAllele(), and Population\_Manager::TheArray.

```
00676 {
00677   char month [13] = {'0','J','F','M','A','m','j','u','a','S','O','N','D'};
00678   int ID=0;
00679   int Y=m_TheLandscape->SupplyYear();
00680   int M=m_TheLandscape->SupplyMonth();
00681   unsigned size= (unsigned) TheArray[listindex].size();
00682   if (size>0)
00683   {
00684    if (listindex==0)
00685    {
00686     Vole_Male* MV;
00687     for (unsigned j=0; j<size; j++)
00688     {
00689       MV=dynamic_cast<Vole_Male*>(TheArray[0][j]);
00690         int x=MV->Supply_m_Location_x();
00691         int y=MV->Supply_m_Location_x();
00692           // Outputs genetic results
00693           // Year \t Month \t sex \t individual code \t XX\tXX\tXX\tXX\tXX\tXX\tXX\tXXn .... 32 loci x 2 alleles
00694           fprintf(ofile,"%d\t",ID);
00695           fprintf(ofile,"M");
00696           fprintf(ofile,"%c",month[M]);
00697           fprintf(ofile,"%d\t",Y);
00698           fprintf(ofile,"%d\t",x);
00699           fprintf(ofile,"%d\t",y);
00700           for (int g=0; g<32; g++)
00701           {
00702              int allele=1+MV->SupplyAllele(g,0);
00703              fprintf(ofile,"%d\t",allele);
00704              allele=1+MV->SupplyAllele(g,1);
00705              fprintf(ofile,"%d\t",allele);
00706           }
00707           fprintf(ofile,"\n");
00708           ID++;
00709         }
00710   }
00711   else
00712   {
00713     Vole_Female* FV;
00714     for (unsigned j=0; j<size; j++)
00715     {
00716       FV=dynamic_cast<Vole_Female*>(TheArray[1][j]);
00717         int x=FV->Supply_m_Location_x();
00718         int y=FV->Supply_m_Location_x();
00719           // Outputs genetic results
00720           // Year \t Month \t sex \t individual code \t XX\tXX\tXX\tXX\tXX\tXX\tXX\tXXn .... 32 loci x 2 alleles
00721           fprintf(ofile,"%d\t",ID);
00722           fprintf(ofile,"F");
00723           fprintf(ofile,"%c",month[M]);
00724           fprintf(ofile,"%d\t",Y);
00725           fprintf(ofile,"%d\t",x);
00726           fprintf(ofile,"%d\t",y);
00727           for (int g=0; g<32; g++)
00728           {
00729              int allele=1+FV->SupplyAllele(g,0);
00730              fprintf(ofile,"%d\t",allele);
00731              allele=1+FV->SupplyAllele(g,1);
00732              fprintf(ofile,"%d\t",allele);
00733           }
00734           fprintf(ofile,"\n");
00735           ID++;
00736         }
00737       }
00738   }
00739 }
```

|  |  |  |  |  |
| --- | --- | --- | --- | --- |
| void Vole\_Population\_Manager::ImpactedProbe | ( |  | ) | `[virtual]` |

use for pesticide simulations only to determine the number of individual males currently impacted by pesticide Added April 2006

Reimplemented from Population\_Manager.

References Vole\_Base::GetDirectFlag(), Vole\_Base::GetGeneticFlag(), and Population\_Manager::TheArray.

```
00279                                              {
00280         FILE* MyFile;
00281         MyFile=fopen("VoleImpactedProbe.txt","a");
00282         // This just needs to trawl through the males and to count how many are affected by 
00283         // a pesticide.  This function needs to be redefined for every new pesticide case
00284         // Current implementation is for Trine Dalkvists 'vinclozolin' work
00285     int dno=0;
00286     int gno=0;
00287         Vole_Male* vm;
00288     unsigned size=(unsigned) TheArray[0].size();
00289         for (unsigned j=0; j<size; j++) {
00290                 vm=dynamic_cast<Vole_Male*>(TheArray[0][j]);
00291                 if (vm->GetGeneticFlag()!=0) gno++;
00292                 if (vm->GetDirectFlag()!=0) dno++;
00293         }
00294         int tno=size;
00295         fprintf(MyFile,"%d\t%d\t%d\n",tno,dno,gno);
00296         fclose(MyFile);
00297 
00298 }
```

|  |  |  |  |  |  |
| --- | --- | --- | --- | --- | --- |
| void Vole\_Population\_Manager::Init | ( | void |  | ) | `[virtual]` |

autom. called by constructor.   
Sets up output files and creates intial population

Used in pesticide simulations

Used in pesticide simulations

Used in pesticide simulations

use for pesticide simulations only

use for pesticide simulations only

use for pesticide simulations only

References AlFreq, Population\_Manager::BeforeStepActions, cfg\_CIPEGridOutput\_used, cfg\_extradispmort, cfg\_f1sterilitychance(), cfg\_geneticproductfertilityeffect(), cfg\_geneticsterilitychance(), cfg\_MoveToLessFavourable(), cfg\_ReallyBigOutput\_used, cfg\_RipleysOutput\_used, cfg\_vole\_starting\_number(), CreateObjects\_Init(), g\_extradispmort, struct\_Vole\_Adult::Genes, GeneticMaterial::Initiation(), struct\_Vole\_Adult::L, Population\_Manager::ListNameLength, Population\_Manager::ListNames, Population\_Manager::m\_AlleleFreqsFile, Population\_Manager::m\_EasyPopRes, m\_f1sterilitychance, m\_geneticimpacted, m\_geneticproductfertilityeffect, Population\_Manager::m\_GeneticsFile, m\_geneticsterilitychance, m\_impacted, m\_notimpacted, Population\_Manager::m\_SimulationName, Population\_Manager::m\_StepSize, Population\_Manager::m\_TheLandscape, MoveToLessFavourable, Population\_Manager::OpenTheCIPEGridOutputProbe(), Population\_Manager::OpenTheReallyBigProbe(), Population\_Manager::OpenTheRipleysOutputProbe(), Population\_Manager::ReallyBigOutputPrb, ReproTable, ReproTable\_base, GeneticMaterial::SetAllele(), Population\_Manager::SimH, Population\_Manager::SimW, Population\_Manager::StateNames, TestFile, TestFile2, tovs\_FDying, tovs\_FEvaluateExplore, tovs\_FMaturation, tovs\_GiveBirth, tovs\_Infanticide, tovs\_InitialState, tovs\_JuvenileExploration, tovs\_Lactating, tovs\_Mating, tovs\_MDying, tovs\_MEvaluateExplore, tovs\_MMaturation, tovs\_ReproBehaviour, tovs\_SpecialExplore, tovs\_UpdateGestation, struct\_Vole\_Adult::VPM, struct\_Vole\_Adult::x, and struct\_Vole\_Adult::y.

Referenced by Vole\_Population\_Manager().

```
00134 {
00139   if ( cfg_RipleysOutput_used.value() ) {
00140     OpenTheRipleysOutputProbe();
00141   }
00142   if ( cfg_ReallyBigOutput_used.value() ) {
00143     OpenTheReallyBigProbe();
00144   } else ReallyBigOutputPrb=0;
00145   if ( cfg_CIPEGridOutput_used.value() ) {
00146         OpenTheCIPEGridOutputProbe();
00147   }
00148   g_extradispmort=cfg_extradispmort.value();
00149 
00151   m_impacted=0;
00153   m_notimpacted=0;
00155   m_geneticimpacted=0;
00156   // Save the impacted file
00157   FILE* impf;
00158   impf= fopen("Impacted.res","w");
00159           if (!impf) {
00160                   m_TheLandscape->Warn("Vole_Population_Manager Destructor","Could Not Open Impacted.Res File");
00161                   exit(0);
00162           }
00163   fclose(impf);
00164   impf = fopen("VoleImpactedProbe.txt","w");
00165           if (!impf) {
00166                   m_TheLandscape->Warn("Vole_Population_Manager Destructor","Could Not Open VoleImpactProbe.txt File");
00167                   exit(0);
00168           }
00169   fclose(impf);
00170 
00171   /*
00172    Create probabilities for ReproTable
00173    
00174    ReproTable is two years each with 12 numbers representing the minimum litter size in the 0,1 position and in 2,3 positions then it is the number out of 100 that have one more than the first number\n
00175    Currently this is set to no variation and litter sizes follow Andrea (1981).
00176   */
00177   for (int i=0; i<12; i++)
00178   {
00179      ReproTable[0][i]=(int)floor(ReproTable_base[0][i]);
00180      ReproTable[2][i]=(int)floor(0.5+(ReproTable_base[0][i]*100)-(ReproTable[0][i]*100));
00181      ReproTable[1][i]=(int)floor(ReproTable_base[1][i]);
00182      ReproTable[3][i]=(int)floor(0.5+(ReproTable_base[1][i]*100)-(ReproTable[1][i]*100));
00183   }
00184   strcpy(m_SimulationName,"Field Vole");
00185   // Create males and females
00186   struct_Vole_Adult* av;
00187   av = new struct_Vole_Adult;
00188   av->VPM = this;
00189   av->L = m_TheLandscape;
00190   GeneticMaterial AGene;
00191   for (int i=0; i<cfg_vole_starting_number.value(); i++)  {
00192       AGene.Initiation(AlFreq);
00193 #ifdef __SpecificPesticideEffectsVinclozolinLike__
00194           AGene.SetAllele(0,0,0);
00195           AGene.SetAllele(0,0,1);
00196 #endif
00197       av->Genes=AGene;
00198       av->x = random(SimW);
00199       av->y = random(SimH);
00200       CreateObjects_Init(0,NULL,av,1);
00201     }
00202     for (int i=0; i<cfg_vole_starting_number.value(); i++)
00203     {
00204       AGene.Initiation(AlFreq);
00205 #ifdef __SpecificPesticideEffects__
00206           AGene.SetAllele(0,0,0);
00207           AGene.SetAllele(0,0,1);
00208 #endif
00209       av->Genes=AGene;
00210       av->x = random(SimW);
00211       av->y = random(SimH);
00212       CreateObjects_Init(1,NULL,av,1);
00213   }
00214   delete av;
00215  // DEBUG
00216  //LOG("FirstLog.txt");
00217   // Load List of Animal Classes
00218   ListNames[0]="Male";
00219   ListNames[1]="Female";
00220   ListNameLength = 2;
00221 
00222   // Set up before step action sorts
00223   // sort w.r.t. x
00224   BeforeStepActions[0]=0; // SortXIndex
00225   BeforeStepActions[1]=0; // SortXIndex
00226 
00227   // Load State Names
00228   StateNames[tovs_InitialState] = "Initial State";
00229   //Males
00230   StateNames[tovs_JuvenileExploration] = "Juvenile Exploration";
00231   StateNames[tovs_MMaturation] = "Maturation";
00232   StateNames[tovs_MEvaluateExplore] = "Evaluate & Explore";
00233   StateNames[tovs_Infanticide] = "Infanticide";
00234   StateNames[tovs_MDying] = "Dying";
00235   //Females
00236   StateNames[tovs_FEvaluateExplore] = "Evaluate & Explore";
00237   StateNames[tovs_ReproBehaviour] = "Repro. Behaviour";
00238   StateNames[tovs_Lactating] = "Lactating";
00239   StateNames[tovs_GiveBirth] = "Give Birth";
00240   StateNames[tovs_FMaturation] = "Maturation";
00241   StateNames[tovs_Mating] = "Mating";
00242   StateNames[tovs_UpdateGestation] = "Update Gestation";
00243   StateNames[tovs_SpecialExplore] = "Special Explore";
00244   StateNames[tovs_FDying] = "Dying";
00245 
00246   MoveToLessFavourable=cfg_MoveToLessFavourable.value();
00247   // OPEN THE TEST FILES _ THIS CODE CAN BE COMMENTED OUT IF THEY ARE NOT NEEDED - TODO TIDY THIS UP
00248   TestFile = fopen("TestFile.Txt", "w" );
00249   if (!TestFile) {
00250           m_TheLandscape->Warn("Population_Manager::Population_Manager -", "Could Not Open TestFile.txt");
00251           exit(0);
00252   }
00253   TestFile2 = fopen("TestFile2.Txt", "w" );
00254   m_StepSize = 1440; // Default - it is up to the individual animal models to
00255   if (!TestFile2) {
00256           m_TheLandscape->Warn("Population_Manager::Population_Manager -", "Could Not Open TestFile2.txt");
00257           exit(0);
00258   }
00260   m_geneticproductfertilityeffect = cfg_geneticproductfertilityeffect.value();
00262   m_geneticsterilitychance = cfg_geneticsterilitychance.value();
00264   m_f1sterilitychance = cfg_f1sterilitychance.value();
00265 // Other output files
00266     char Nme[ 511 ];
00267     strcpy( Nme, "GeneticsOutput.txt" );
00268     m_GeneticsFile = fopen( Nme, "w" );
00269     strcpy( Nme, "AlleleFreqs.txt" );
00270     m_AlleleFreqsFile = fopen( Nme, "w" );
00271     strcpy( Nme, "forEASYPOP.txt" );
00272     m_EasyPopRes = fopen( Nme, "w" );
00273 
00274 }
```

|  |  |  |  |
| --- | --- | --- | --- |
| bool Vole\_Population\_Manager::InSquare | ( | int | *p\_x*, |
|  |  | int | *p\_y*, |
|  |  | int | *p\_sqx*, |
|  |  | int | *p\_sqy*, |
|  |  | int | *p\_range* |  |
|  | ) |  |  |  |

Determines if p\_x,p\_y is the in the square denoted by p\_sqx,p\_sqy + p\_range

References Population\_Manager::SimH, and Population\_Manager::SimW.

Referenced by SendMessage().

```
01605 {
01608     int x_extent = p_sqx+p_range;
01609     int y_extent = p_sqy+p_range;
01610     if (x_extent >= SimW)
01611     {
01612       if (y_extent >= SimH)  // overlaps TR corner of sim area
01613                              // Must test four rectangles
01614       /*
01615         1) p_sqx to <SimW, p_sq_y to SimW (TR)
01616         2) 0 to x_extent-SimW p_sq_y to SimH (TL)
01617         3) 0 to x_extent-SimW, 0 to y_extent-SimH (BL)
01618         4) p_sq_x to <SimW, 0 to y_extent-SimH (BR)
01619       */
01620       {
01621         // 1 Top right square (limited by SimAreaHeight & SimAreaWidth
01622         if ((p_x >=p_sqx) && (p_y >=p_sqy)) return true;
01623         // 2 Top Left Square (limited by 0,SimAreaHeight)
01624         if ((p_x <x_extent-SimW)&& (p_y>p_sqy)) return true;
01625         // 3 Bottom Left square (limited by 0,0)
01626         if ((p_x <x_extent-SimW)&&(p_y<y_extent-SimH)) return true;
01627         // Bottom Right square (limited by SimAreaWidth,0)
01628         if ((p_x >=p_sqx)&& (p_y<y_extent-SimH)) return true;
01629 
01630       }
01631       else // Overlaps the west edge of the sim area
01632       {
01633         if ((p_y >=p_sqy) && (p_y<y_extent))
01634         {  // y is in square
01635           if (p_x >=p_sqx) return true;
01636           else if (p_x <x_extent-SimW) return true;
01637         }
01638       }
01639     }
01640     else
01641     {
01642       if (y_extent >= SimH) // overlaps top of simulation area
01643       {
01644         if ((p_x >=p_sqx) && (p_x<x_extent))
01645         {
01646           // x is OK
01647           if (p_y >=p_sqy) return true;
01648           else if (p_y<y_extent-SimH) return true;
01649         }
01650       }
01651       else // square does not overlap end of simulation area
01652       {
01653         if ((p_x >=p_sqx) && (p_x<x_extent) &&
01654                          (p_y >=p_sqy) && (p_y<y_extent)) return true;
01655       }
01656     }
01657     return false; // not in square
01658 }
```

|  |  |  |  |
| --- | --- | --- | --- |
| int Vole\_Population\_Manager::ListClosestFemales | ( | int | *p\_x*, |
|  |  | int | *p\_y*, |
|  |  | int | *p\_steps* |  |
|  | ) |  |  |  |

Lists all females within p\_steps of p\_x & p\_y and returns the number in the list

References FList, Population\_Manager::SimH, Population\_Manager::SimW, Vole\_Base::SupplySex(), Vole\_Base::SupplyTerritorial(), and VoleMap.

```
00896 {
00897   // First clear the FemaleVoleList
00898   FList.clear();
00899   // looks for a female within p_Steps of p_x,p_y
00900   // as usual there are 4 possibilities of overlap
00901   // First convert centre co-ordinates to square co-ordinates
00902 
00903   int x=p_x - p_steps;
00904   if (x<0) x+=SimW;  // ensure we start in the simulation area!
00905   int y=p_y - p_steps;
00906   if (y<0) y+=SimH;
00907   int range_x=p_steps*2;
00908   int range_y=p_steps*2;
00909 
00910   // create the extent variables
00911   int xextent0 = x+range_x;
00912   int yextent0 = y+range_y;
00913   int xextent1 = (x+range_x)-SimW;
00914   int yextent1 = (y+range_y)-SimH;
00915   // Create the looping variables needed
00916   // Create the looping variables needed
00917   int Dfinx;
00918   int Dfiny;
00919   int Afinx=0;  //unless the finx values for A are changed this stop
00920   int Afiny=0;  //the loop from executing
00921   int Asty=0;
00922   // int Astx,Dstx,Dtsy are always default so variables not used from them
00923   // NB Astx, Asty and Dstx are always 0, 0 & x respectively
00924   // Dsty is always y, Afiny is always yextent1 if it is used
00925   // Now create the loop values;
00926   if (xextent0<=SimW)   // No overlap with the eastern side
00927   {
00928     // Dstx, Dsty, Asty set by defaults
00929     //Astx & Afinx are not needed
00930     Dfinx=xextent0;
00931     // do we overlap the bottom?
00932     // Type B & D (overlap bottom, no overlap)
00933     if (yextent0>SimH)
00934     {
00935       // Type B (overlap bottom only)
00936       Dfiny=SimH;  // stop at the end
00937       Afiny=yextent1; // the overlap with the top
00938     }
00939     else Dfiny=yextent0;
00940   }
00941   else
00942   {
00943     // Type A & C overlap bottom & eastern edgdes
00944     if (yextent0>SimH)
00945     {
00946       // relies on the default start for Asty, Astx, Dstx, Dsty
00947       Afinx=xextent1;
00948       Afiny=yextent1;
00949       Dfinx=SimW;  // Stop at the end
00950       Dfiny=SimH;
00951     }
00952     else
00953     {
00954       // Type C overlap left edge only
00955       // Astx & Afiny are not needed here
00956       //Astx, Dstx, Dsty set by default
00957       Afinx=xextent1;
00958       Dfinx=SimW;  // Stop at the end
00959       Dfiny=yextent0;
00960     }
00961   }
00962   Vole_Female* AFemale;
00963   Vole_Base* Ap;
00964   int NoFound=0;
00965 
00966     // A Loop
00967     for (int i=0; i<Afinx; i++)
00968     {
00969       for (int j=Asty; j<Afiny; j++)
00970       {
00971         Ap=(Vole_Base*) (VoleMap->GetMapValue(i,j)) ;
00972         if (Ap)
00973         {
00974           if (!Ap->SupplySex()) // female
00975           {
00976             AFemale=(Vole_Female*) (Ap) ;
00977             if (AFemale->SupplyTerritorial())
00978             {
00979               FList.push_back(AFemale);
00980               NoFound++;
00981             }
00982           }
00983         }
00984       }
00985       // C Loop
00986       for (int j=y; j<Dfiny; j++)
00987       {
00988         Ap=(Vole_Base*) (VoleMap->GetMapValue(i,j)) ;
00989         if (Ap)
00990         {
00991           if (!Ap->SupplySex()) // female
00992           {
00993             AFemale=(Vole_Female*) (Ap) ;
00994             if (AFemale->SupplyTerritorial())
00995             {
00996               FList.push_back(AFemale);
00997               NoFound++;
00998             }
00999           }
01000         }
01001       }
01002     }
01003     // D Loop
01004     for (int i=x; i<Dfinx; i++)
01005     {
01006       for (int j=y; j<Dfiny; j++)
01007       {
01008         Ap=(Vole_Base*) (VoleMap->GetMapValue(i,j)) ;
01009         if (Ap)
01010         {
01011           if (!Ap->SupplySex()) // female
01012           {
01013             AFemale=(Vole_Female*) (Ap) ;
01014             if (AFemale->SupplyTerritorial())
01015             {
01016               FList.push_back(AFemale);
01017               NoFound++;
01018             }
01019           }
01020         }
01021       }
01022       // B Loop
01023       for (int j=0; j<Afiny; j++)
01024       {
01025         Ap=(Vole_Base*) (VoleMap->GetMapValue(i,j)) ;
01026         if (Ap)
01027         {
01028           if (!Ap->SupplySex()) // female
01029           {
01030             AFemale=(Vole_Female*) (Ap) ;
01031             if (AFemale->SupplyTerritorial())
01032             {
01033               FList.push_back(AFemale);
01034               NoFound++;
01035             }
01036           }
01037         }
01038       }
01039     }
01040   return NoFound;
01041 }
```

|  |  |  |  |
| --- | --- | --- | --- |
| int Vole\_Population\_Manager::ListClosestMales | ( | int | *p\_x*, |
|  |  | int | *p\_y*, |
|  |  | int | *p\_steps* |  |
|  | ) |  |  |  |

Looks for males within p\_Steps of p\_x,p\_y and returns the number of them. Pointers to these are saved in MList

References MList, Population\_Manager::SimH, Population\_Manager::SimW, Vole\_Base::SupplySex(), Vole\_Base::SupplyTerritorial(), and VoleMap.

```
00746 {
00747   // First clear the MaleVoleList
00748   MList.clear();
00749 
00750   // as usual there are 4 possibilities of overlap
00751   // First convert centre co-rdinates to square co-ordinates
00752 
00753   int x=p_x - p_steps;
00754   if (x<0) x+=SimW;  // ensure we start in the simulation area!
00755   int y=p_y - p_steps;
00756   if (y<0) y+=SimH;
00757   int range_x=p_steps*2;
00758   int range_y=p_steps*2;
00759 
00760   // create the extent variables
00761   int xextent0 = x+range_x;
00762   int yextent0 = y+range_y;
00763   int xextent1 = (x+range_x)-SimW;
00764   int yextent1 = (y+range_y)-SimH;
00765   // Create the looping variables needed
00766   // Create the looping variables needed
00767   int Dfinx;
00768   int Dfiny;
00769   int Afinx=0;  //unless the finx values for A are changed this stop
00770   int Afiny=0;  //the loop from executing
00771   int Asty=0;
00772   // int Astx,Dstx,Dtsy are always default so variables not used from them
00773   // NB Astx, Asty and Dstx are always 0, 0 & x respectively
00774   // Dsty is always y, Afiny is always yextent1 if it is used
00775   // Now create the loop values;
00776   if (xextent0<=SimW)   // No overlap with the eastern side
00777   {
00778     // Dstx, Dsty, Asty set by defaults
00779     //Astx & Afinx are not needed
00780     Dfinx=xextent0;
00781     // do we overlap the bottom?
00782     // Type B & D (overlap bottom, no overlap)
00783     if (yextent0>SimH)
00784     {
00785       // Type B (overlap bottom only)
00786       Dfiny=SimH;  // stop at the end
00787       Afiny=yextent1; // the overlap with the top
00788     }
00789     else Dfiny=yextent0;
00790   }
00791   else
00792   {
00793     // Type A & C overlap bottom & eastern edgdes
00794     if (yextent0>SimH)
00795     {
00796       // relies on the default start for Asty, Astx, Dstx, Dsty
00797       Afinx=xextent1;
00798       Afiny=yextent1;
00799       Dfinx=SimW;  // Stop at the end
00800       Dfiny=SimH;
00801     }
00802     else
00803     {
00804       // Type C overlap left edge only
00805       // Astx & Afiny are not needed here
00806       //Astx, Dstx, Dsty set by default
00807       Afinx=xextent1;
00808       Dfinx=SimW;  // Stop at the end
00809       Dfiny=yextent0;
00810     }
00811   }
00812   Vole_Base* Ap;
00813   Vole_Male* AMale;
00814   int NoFound=0;
00815 
00816     // A Loop
00817     for (int i=0; i<Afinx; i++)
00818     {
00819       for (int j=Asty; j<Afiny; j++)
00820       {
00821         Ap=(Vole_Base*) (VoleMap->GetMapValue(i,j)) ;
00822         if (Ap)
00823         {
00824           if (Ap->SupplySex()) // male
00825           {
00826             AMale=(Vole_Male*) Ap;
00827             if (AMale->SupplyTerritorial())
00828             {
00829               MList.push_back(AMale);
00830               NoFound++;
00831             }
00832           }
00833         }
00834       }
00835       // C Loop
00836       for (int j=y; j<Dfiny; j++)
00837       {
00838         Ap=(Vole_Base*) (VoleMap->GetMapValue(i,j)) ;
00839         if (Ap)
00840         {
00841           if (Ap->SupplySex()) // male
00842           {
00843             AMale=(Vole_Male*) (Ap) ;
00844             if (AMale->SupplyTerritorial())
00845             {
00846               MList.push_back(AMale);
00847               NoFound++;
00848             }
00849           }
00850         }
00851       }
00852     }
00853     // D Loop
00854     for (int i=x; i<Dfinx; i++)
00855     {
00856       for (int j=y; j<Dfiny; j++)
00857       {
00858         Ap=(Vole_Base*) (VoleMap->GetMapValue(i,j)) ;
00859         if (Ap)
00860         {
00861           if (Ap->SupplySex()) // male
00862           {
00863             AMale=(Vole_Male*) (Ap) ;
00864             if (AMale->SupplyTerritorial())
00865             {
00866               MList.push_back(AMale);
00867               NoFound++;
00868             }
00869           }
00870         }
00871       }
00872       // B Loop
00873       for (int j=0; j<Afiny; j++)
00874       {
00875         Ap=(Vole_Base*) (VoleMap->GetMapValue(i,j)) ;
00876         if (Ap)
00877         {
00878           if (Ap->SupplySex()) // male
00879           {
00880             AMale=(Vole_Male*) (Ap) ;
00881             if (AMale->SupplyTerritorial())
00882             {
00883               MList.push_back(AMale);
00884               NoFound++;
00885             }
00886           }
00887         }
00888       }
00889     }
00890   return NoFound;
00891 }
```

|  |  |  |  |  |
| --- | --- | --- | --- | --- |
| void Vole\_Population\_Manager::ReproductionProbe | ( |  | ) |  |

References JuvsProducedToday, YoungProducedToday, and YoungsFile.

```
00667 {
00668    fprintf(YoungsFile,"%i   %i\n",YoungProducedToday,JuvsProducedToday);
00669 }
```

|  |  |  |  |
| --- | --- | --- | --- |
| void Vole\_Population\_Manager::SendMessage | ( | TTypeOfVoleMessage | *p\_message*, |
|  |  | unsigned | *p\_x*, |
|  |  | unsigned | *p\_y*, |
|  |  | unsigned | *p\_range*, |
|  |  | unsigned | *p\_age*, |
|  |  | bool | *p\_sex* |  |
|  | ) |  |  |  |

Passes a message to recipients. In this case the only one we use is infanticide sent to all females within an area

References InSquare(), Population\_Manager::m\_TheLandscape, Vole\_Female::OnInfanticideAttempt(), Vole\_Base::SupplyAge(), Vole\_Base::SupplyX(), Vole\_Base::SupplyY(), Population\_Manager::TheArray, and tovm\_Infanticide.

Referenced by Vole\_Male::st\_Infanticide().

```
01779 {
01781   if (p_message_type==tovm_Infanticide)
01782   {
01783       if (p_sex==false) // female
01784       {
01785         for (unsigned i=0; i<TheArray[1].size(); i++)
01786         {
01787           Vole_Female *AFemale;
01788           AFemale=(Vole_Female *)TheArray[1][i];
01789           if (AFemale->SupplyAge() > p_age)
01790           {
01791              // is it in the square defined by p_x,p_y & p_range
01792              unsigned x=AFemale->SupplyX();
01793              unsigned y=AFemale->SupplyY();
01794              if (InSquare(x,y,p_x,p_y,p_range))
01795               {
01796                  if (AFemale->SupplyAge()>p_age)
01797                  {
01798                    AFemale->OnInfanticideAttempt();
01799                  }
01800               }
01801           }
01802         }
01803       }
01804       else
01805       {
01806         m_TheLandscape->Warn("Vole_Population_Manager::SendMessage Error","Wrong sex specified for infanticide");
01807       }
01808   }
01809   else
01810   {
01811     m_TheLandscape->Warn("Vole_Population_Manager::SendMessage Error","Unknown message");
01812   }
01813 
01814 }
```

|  |  |  |  |  |
| --- | --- | --- | --- | --- |
| int Vole\_Population\_Manager::SupplyGrowthStartDate | ( |  | ) | `[inline]` |

References m\_GrowthStartDate.

Referenced by Vole\_Male::Dispersal(), Vole\_Male::EndStep(), Vole\_Female::EndStep(), Vole\_Female::st\_BecomeReproductive(), Vole\_Male::st\_Eval\_n\_Explore(), Vole\_Female::st\_Evaluate\_n\_Explore(), Vole\_Female::st\_Mating(), and Vole\_Male::st\_Maturation().

```
00091 {return m_GrowthStartDate;}
```

|  |  |  |  |
| --- | --- | --- | --- |
| int Vole\_Population\_Manager::SupplyHowManyVoles | ( | unsigned | *p\_x*, |
|  |  | unsigned | *p\_y*, |
|  |  | unsigned | *p\_range* |  |
|  | ) |  |  |  |

Counts vole postions on the map from p\_x-p\_range,p-y-p\_range to p\_x+p\_size, p\_y+p\_range

References Population\_Manager::SimH, Population\_Manager::SimW, and VoleMap.

Referenced by Vole\_Male::st\_Eval\_n\_Explore(), and Vole\_Female::st\_Evaluate\_n\_Explore().

```
00575 {
00576   int x=p_x - p_range;
00577   if (x<0) x+=SimW;  // ensure we start in the simulation area!
00578   int y=p_y - p_range;
00579   if (y<0) y+=SimH;
00580   int range_x=p_range*2;
00581   int range_y=p_range*2;
00582   int Voles=0;
00583   // create the extent variables
00584   int xextent0 = x+range_x;
00585   int yextent0 = y+range_y;
00586   int xextent1 = (x+range_x)-SimW;
00587   int yextent1 = (y+range_y)-SimH;
00588   // Create the looping variables needed
00589   // Create the looping variables needed
00590   int Dfinx;
00591   int Dfiny;
00592   int Afinx=0;  //unless the finx values for A are changed this stop
00593   int Afiny=0;  //the loop from executing
00594   int Asty=0;
00595   // int Astx,Dstx,Dtsy are always default so variables not used from them
00596   // NB Astx, Asty and Dstx are always 0, 0 & x respectively
00597   // Dsty is always y, Afiny is always yextent1 if it is used
00598   // Now create the loop values;
00599   if (xextent0<=SimW)   // No overlap with the eastern side
00600   {
00601     // Dstx, Dsty, Asty set by defaults
00602     //Astx & Afinx are not needed
00603     Dfinx=xextent0;
00604     // do we overlap the bottom?
00605     // Type B & D (overlap bottom, no overlap)
00606     if (yextent0>SimH)
00607     {
00608       // Type B (overlap bottom only)
00609       Dfiny=SimH;  // stop at the end
00610       Afiny=yextent1; // the overlap with the top
00611     }
00612     else Dfiny=yextent0;
00613   }
00614   else
00615   {
00616     // Type A & C overlap bottom & eastern edgdes
00617     if (yextent0>SimH)
00618     {
00619       // relies on the default start for Asty, Astx, Dstx, Dsty
00620       Afinx=xextent1;
00621       Afiny=yextent1;
00622       Dfinx=SimW;  // Stop at the end
00623       Dfiny=SimH;
00624     }
00625     else
00626     {
00627       // Type C overlap left edge only
00628       // Astx & Afiny are not needed here
00629       //Astx, Dstx, Dsty set by default
00630       Afinx=xextent1;
00631       Dfinx=SimW;  // Stop at the end
00632       Dfiny=yextent0;
00633     }
00634   }
00635   // A Loop
00636   for (int i=0; i<Afinx; i++)
00637   {
00638     for (int j=Asty; j<Afiny; j++)
00639     {
00640       if(VoleMap->GetMapValue(i,j)) Voles++;
00641     }
00642     // C Loop
00643     for (int j=y; j<Dfiny; j++)
00644     {
00645       if(VoleMap->GetMapValue(i,j)) Voles++;
00646     }
00647   }
00648   // D Loop
00649   for (int i=x; i<Dfinx; i++)
00650   {
00651     for (int j=y; j<Dfiny; j++)
00652     {
00653       if(VoleMap->GetMapValue(i,j)) Voles++;
00654     }
00655     // B Loop
00656     for (int j=0; j<Afiny; j++)
00657     {
00658       if(VoleMap->GetMapValue(i,j)) Voles++;
00659     }
00660   }
00661   // End of search algorithm
00662   return Voles;
00663 }
```

|  |  |  |  |
| --- | --- | --- | --- |
| int Vole\_Population\_Manager::SupplyInOlderTerr | ( | unsigned | *p\_x*, |
|  |  | unsigned | *p\_y*, |
|  |  | unsigned | *p\_Age*, |
|  |  | unsigned | *p\_Range* |  |
|  | ) |  |  |  |

returns -1 if a male has p\_x,p\_y in his territory and is older than p\_Age else returns the number of females present

References Population\_Manager::SimH, Population\_Manager::SimW, Vole\_Base::SupplyAge(), Vole\_Base::SupplySex(), Vole\_Base::SupplyTerritorial(), and VoleMap.

```
01411 {
01415   // Before checking the map remove ourselves so we don't count
01416   PointerInt c=VoleMap->GetMapValue(p_x,p_y);
01417   TAnimal* caller=(TAnimal*) c;
01418   VoleMap->ClearMapValue(p_x,p_y);
01419   int x=p_x - p_Range;
01420   if (x<0) x+=SimW;  // ensure we start in the simulation area!
01421   int y=p_y - p_Range;
01422   if (y<0) y+=SimH;
01423   int range_x=p_Range*2;
01424   int range_y=p_Range*2;
01425   int Females=0;
01426 
01427   // create the extent variables
01428   int xextent0 = x+range_x;
01429   int yextent0 = y+range_y;
01430   int xextent1 = (x+range_x)-SimW;
01431   int yextent1 = (y+range_y)-SimH;
01432   // Create the looping variables needed
01433   // Create the looping variables needed
01434   int Dfinx;
01435   int Dfiny;
01436   int Afinx=0;  //unless the finx values for A are changed this stop
01437   int Afiny=0;  //the loop from executing
01438   int Asty=0;
01439   // int Astx,Dstx,Dtsy are always default so variables not used from them
01440   // NB Astx, Asty and Dstx are always 0, 0 & x respectively
01441   // Dsty is always y, Afiny is always yextent1 if it is used
01442   // Now create the loop values;
01443   if (xextent0<=SimW)   // No overlap with the eastern side
01444   {
01445     // Dstx, Dsty, Asty set by defaults
01446     //Astx & Afinx are not needed
01447     Dfinx=xextent0;
01448     // do we overlap the bottom?
01449     // Type B & D (overlap bottom, no overlap)
01450     if (yextent0>SimH)
01451     {
01452       // Type B (overlap bottom only)
01453       Dfiny=SimH;  // stop at the end
01454       Afiny=yextent1; // the overlap with the top
01455     }
01456     else Dfiny=yextent0;
01457   }
01458   else
01459   {
01460     // Type A & C overlap bottom & eastern edgdes
01461     if (yextent0>SimH)
01462     {
01463       // relies on the default start for Asty, Astx, Dstx, Dsty
01464       Afinx=xextent1;
01465       Afiny=yextent1;
01466       Dfinx=SimW;  // Stop at the end
01467       Dfiny=SimH;
01468     }
01469     else
01470     {
01471       // Type C overlap left edge only
01472       // Astx & Afiny are not needed here
01473       //Astx, Dstx, Dsty set by default
01474       Afinx=xextent1;
01475       Dfinx=SimW;  // Stop at the end
01476       Dfiny=yextent0;
01477     }
01478   }
01479   Vole_Male* AMale;
01480   Vole_Base* Ap;
01481 
01482     // A Loop
01483     for (int i=0; i<Afinx; i++)
01484     {
01485       for (int j=Asty; j<Afiny; j++)
01486       {
01487         Ap=(Vole_Base*) (VoleMap->GetMapValue(i,j)) ;
01488         if (Ap)
01489         {
01490           if (Ap->SupplySex()) // male
01491           {
01492             AMale=(Vole_Male*) (Ap) ;
01493             if (AMale->SupplyTerritorial())
01494             {
01495               if (AMale->SupplyAge()>=p_Age)
01496               {
01497                  VoleMap->SetMapValue(p_x,p_y,caller);
01498                  return -1;   // No Good
01499               }
01500             }
01501           }
01502           else  // is a female
01503           {
01504             if (Ap->SupplyTerritorial())
01505             {
01506               Females++;
01507             }
01508           }
01509         }
01510       }
01511       // C Loop
01512       for (int j=y; j<Dfiny; j++)
01513       {
01514         Ap=(Vole_Base*) (VoleMap->GetMapValue(i,j)) ;
01515         if (Ap)
01516         {
01517           if (Ap->SupplySex()) // male
01518           {
01519             AMale=(Vole_Male*) (Ap) ;
01520             if (AMale->SupplyTerritorial())
01521             {
01522               if (AMale->SupplyAge()>=p_Age)
01523               {
01524                  VoleMap->SetMapValue(p_x,p_y,caller);
01525                  return -1;   // No Good
01526               }
01527             }
01528           }
01529           else  // is a female
01530           {
01531             if (Ap->SupplyTerritorial())
01532             {
01533               Females++;
01534             }
01535           }
01536         }
01537       }
01538     }
01539     // D Loop
01540     for (int i=x; i<Dfinx; i++)
01541     {
01542       for (int j=y; j<Dfiny; j++)
01543       {
01544         Ap=(Vole_Base*) (VoleMap->GetMapValue(i,j)) ;
01545         if (Ap)
01546         {
01547           if (Ap->SupplySex()) // male
01548           {
01549             AMale=(Vole_Male*) (Ap) ;
01550             if (AMale->SupplyTerritorial())
01551             {
01552               if (AMale->SupplyAge()>=p_Age)
01553               {
01554                  VoleMap->SetMapValue(p_x,p_y,caller);
01555                  return -1;   // No Good
01556               }
01557             }
01558           }
01559           else  // is a female
01560           {
01561             if (Ap->SupplyTerritorial())
01562             {
01563               Females++;
01564             }
01565           }
01566         }
01567       }
01568       // B Loop
01569       for (int j=0; j<Afiny; j++)
01570       {
01571         Ap=(Vole_Base*) (VoleMap->GetMapValue(i,j)) ;
01572         if (Ap)
01573         {
01574           if (Ap->SupplySex()) // male
01575           {
01576             AMale=(Vole_Male*) (Ap) ;
01577             if (AMale->SupplyTerritorial())
01578             {
01579               if (AMale->SupplyAge()>=p_Age)
01580               {
01581                  VoleMap->SetMapValue(p_x,p_y,caller);
01582                  return -1;   // No Good
01583               }
01584             }
01585           }
01586           else  // is a female
01587           {
01588             if (Ap->SupplyTerritorial())
01589             {
01590               Females++;
01591             }
01592           }
01593         }
01594       }
01595     }
01596    // End of search algorithm
01597    VoleMap->SetMapValue(p_x,p_y,caller);
01598   return Females; // No Males so return the number of adult females
01599 }
```

|  |  |  |  |
| --- | --- | --- | --- |
| bool Vole\_Population\_Manager::SupplyOlderFemales | ( | unsigned | *p\_x*, |
|  |  | unsigned | *p\_y*, |
|  |  | unsigned | *p\_Age*, |
|  |  | unsigned | *p\_range* |  |
|  | ) |  |  |  |

Returns false if there is an older female within the area p\_x,p\_y +/- range

References Population\_Manager::SimH, Population\_Manager::SimW, Vole\_Base::SupplyAge(), Vole\_Base::SupplySex(), and VoleMap.

Referenced by Vole\_Female::st\_Special\_Explore().

```
00428 {
00431   // Before checking the map remove ourselves so we don't count
00432   TAnimal* caller=(TAnimal*)VoleMap->GetMapValue(p_x,p_y);
00433   VoleMap->ClearMapValue(p_x,p_y);
00434   // This is reset when the result is known
00435   int x=p_x - p_range;
00436   if (x<0) x+=SimW;  // ensure we start in the simulation area!
00437   int y=p_y - p_range;
00438   if (y<0) y+=SimH;
00439   int range_x=p_range*2;
00440   int range_y=p_range*2;
00441   // create the extent variables
00442   int xextent0 = x+range_x;
00443   int yextent0 = y+range_y;
00444   int xextent1 = (x+range_x)-SimW;
00445   int yextent1 = (y+range_y)-SimH;
00446   // Create the looping variables needed
00447   // Create the looping variables needed
00448   int Dfinx;
00449   int Dfiny;
00450   int Afinx=0;  //unless the finx values for A are changed this stop
00451   int Afiny=0;  //the loop from executing
00452   int Asty=0;
00453   // int Astx,Dstx,Dtsy are always default so variables not used from them
00454   // NB Astx, Asty and Dstx are always 0, 0 & x respectively
00455   // Dsty is always y, Afiny is always yextent1 if it is used
00456   // Now create the loop values;
00457   if (xextent0<=SimW)   // No overlap with the eastern side
00458   {
00459     // Dstx, Dsty, Asty set by defaults
00460     //Astx & Afinx are not needed
00461     Dfinx=xextent0;
00462     // do we overlap the bottom?
00463     // Type B & D (overlap bottom, no overlap)
00464     if (yextent0>SimH)
00465     {
00466       // Type B (overlap bottom only)
00467       Dfiny=SimH;  // stop at the end
00468       Afiny=yextent1; // the overlap with the top
00469     }
00470     else Dfiny=yextent0;
00471   }
00472   else
00473   {
00474     // Type A & C overlap bottom & eastern edgdes
00475     if (yextent0>SimH)
00476     {
00477       // relies on the default start for Asty, Astx, Dstx, Dsty
00478       Afinx=xextent1;
00479       Afiny=yextent1;
00480       Dfinx=SimW;  // Stop at the end
00481       Dfiny=SimH;
00482     }
00483     else
00484     {
00485       // Type C overlap left edge only
00486       // Astx & Afiny are not needed here
00487       //Astx, Dstx, Dsty set by default
00488       Afinx=xextent1;
00489       Dfinx=SimW;  // Stop at the end
00490       Dfiny=yextent0;
00491     }
00492   }
00493   Vole_Base* Ap;
00494   // A Loop
00495   for (int i=0; i<Afinx; i++)
00496   {
00497     for (int j=Asty; j<Afiny; j++)
00498     {
00499       Ap=(Vole_Base*) (VoleMap->GetMapValue(i,j)) ;
00500       if (Ap)
00501       {
00502         if (Ap->SupplySex()) // female
00503         {
00504           if (Ap->SupplyAge()>=p_Age)
00505           {
00506             VoleMap->SetMapValue(p_x,p_y,caller);
00507             return false;
00508           }
00509         }
00510       }
00511     }
00512     // C Loop
00513     for (int j=y; j<Dfiny; j++)
00514     {
00515       Ap=(Vole_Base*) (VoleMap->GetMapValue(i,j)) ;
00516       if (Ap)
00517       {
00518         if (Ap->SupplySex()) // female
00519         {
00520           if (Ap->SupplyAge()>=p_Age)
00521           {
00522             VoleMap->SetMapValue(p_x,p_y,caller);
00523             return false;
00524           }
00525         }
00526       }
00527     }
00528   }
00529   // D Loop
00530   for (int i=x; i<Dfinx; i++)
00531   {
00532     for (int j=y; j<Dfiny; j++)
00533     {
00534       Ap=(Vole_Base*) (VoleMap->GetMapValue(i,j)) ;
00535       if (Ap)
00536       {
00537         if (Ap->SupplySex()) // female
00538         {
00539           if (Ap->SupplyAge()>=p_Age)
00540           {
00541             VoleMap->SetMapValue(p_x,p_y,caller);
00542             return false;
00543           }
00544         }
00545       }
00546     }
00547     // B Loop
00548     for (int j=0; j<Afiny; j++)
00549     {
00550       Ap=(Vole_Base*) (VoleMap->GetMapValue(i,j)) ;
00551       if (Ap)
00552       {
00553         if (Ap->SupplySex()) // female
00554         {
00555           if (Ap->SupplyAge()>=p_Age)
00556           {
00557             VoleMap->SetMapValue(p_x,p_y,caller);
00558             return false;
00559           }
00560         }
00561       }
00562     }
00563   }
00564   // End of search algorithm
00565    VoleMap->SetMapValue(p_x,p_y,caller);
00566   return true;
00567 }
```

|  |  |  |  |
| --- | --- | --- | --- |
| vector< Vole\_Base \* > \* Vole\_Population\_Manager::SupplyVoleList | ( | unsigned | *x*, |
|  |  | unsigned | *y*, |
|  |  | unsigned | *range* |  |
|  | ) |  |  |  |

returns a list of all voles in p\_x,p\_y, p\_range square

References Population\_Manager::SimH, Population\_Manager::SimW, and VoleMap.

Referenced by TPredator::st\_Hunting().

```
01664 {
01667   vector<Vole_Base*> *vbl;
01668   vbl=new vector<Vole_Base*>;
01669   // This is reset when the result is known
01670   int x=p_x;
01671   if (x<0) x+=SimW;  // ensure we start in the simulation area!
01672   int y=p_y;
01673   if (y<0) y+=SimH;
01674   int range_x=p_Range;
01675   int range_y=p_Range;
01676   // create the extent variables
01677   int xextent0 = x+range_x;
01678   int yextent0 = y+range_y;
01679   int xextent1 = (x+range_x)-SimW;
01680   int yextent1 = (y+range_y)-SimH;
01681   // Create the looping variables needed
01682   // Create the looping variables needed
01683   int Dfinx;
01684   int Dfiny;
01685   int Afinx=0;  //unless the finx values for A are changed this stop
01686   int Afiny=0;  //the loop from executing
01687   int Asty=0;
01688   // int Astx,Dstx,Dtsy are always default so variables not used from them
01689   // NB Astx, Asty and Dstx are always 0, 0 & x respectively
01690   // Dsty is always y, Afiny is always yextent1 if it is used
01691   // Now create the loop values;
01692   if (xextent0<=SimW)   // No overlap with the eastern side
01693   {
01694     // Dstx, Dsty, Asty set by defaults
01695     //Astx & Afinx are not needed
01696     Dfinx=xextent0;
01697     // do we overlap the bottom?
01698     // Type B & D (overlap bottom, no overlap)
01699     if (yextent0>SimH)
01700     {
01701       // Type B (overlap bottom only)
01702       Dfiny=SimH;  // stop at the end
01703       Afiny=yextent1; // the overlap with the top
01704     }
01705     else Dfiny=yextent0;
01706   }
01707   else
01708   {
01709     // Type A & C overlap bottom & eastern edgdes
01710     if (yextent0>SimH)
01711     {
01712       // relies on the default start for Asty, Astx, Dstx, Dsty
01713       Afinx=xextent1;
01714       Afiny=yextent1;
01715       Dfinx=SimW;  // Stop at the end
01716       Dfiny=SimH;
01717     }
01718     else
01719     {
01720       // Type C overlap left edge only
01721       // Astx & Afiny are not needed here
01722       //Astx, Dstx, Dsty set by default
01723       Afinx=xextent1;
01724       Dfinx=SimW;  // Stop at the end
01725       Dfiny=yextent0;
01726     }
01727   }
01728   Vole_Base* Ap;
01729 
01730     // A Loop
01731     for (int i=0; i<Afinx; i++)
01732     {
01733       for (int j=Asty; j<Afiny; j++)
01734       {
01735         Ap=(Vole_Base*) (VoleMap->GetMapValue(i,j)) ;
01736         if (Ap)
01737         {
01738           vbl->push_back(Ap);
01739         }
01740       }
01741       // C Loop
01742       for (int j=y; j<Dfiny; j++)
01743       {
01744         Ap=(Vole_Base*) (VoleMap->GetMapValue(i,j)) ;
01745         if (Ap)
01746         {
01747           vbl->push_back(Ap);
01748         }
01749       }
01750     }
01751     // D Loop
01752     for (int i=x; i<Dfinx; i++)
01753     {
01754       for (int j=y; j<Dfiny; j++)
01755       {
01756         Ap=(Vole_Base*) (VoleMap->GetMapValue(i,j)) ;
01757         if (Ap)
01758         {
01759           vbl->push_back(Ap);
01760         }
01761       }
01762       // B Loop
01763       for (int j=0; j<Afiny; j++)
01764       {
01765         Ap=(Vole_Base*) (VoleMap->GetMapValue(i,j)) ;
01766         if (Ap)
01767         {
01768           vbl->push_back(Ap);
01769         }
01770       }
01771     }
01772   return vbl;
01773 }
```

|  |  |  |  |  |
| --- | --- | --- | --- | --- |
| void Vole\_Population\_Manager::TheCIPEGridOutputProbe | ( |  | ) | `[virtual]` |

This method MUST be overridden in descendent classes, if you want it to work

Reimplemented from Population\_Manager.

References \_\_CIPEGRIDSIZE, Population\_Manager::CIPEGridOutputPrb, Population\_Manager::gridcount, TAnimal::Supply\_m\_Location\_x(), TAnimal::Supply\_m\_Location\_y(), Vole\_Base::SupplyTerritorial(), and Population\_Manager::TheArray.

```
01983                                                        {
01985   Vole_Female* FS; 
01986   unsigned int totalF= (unsigned int) TheArray[1].size();
01987   int x,y;
01988   //int mx=m_TheLandscape->SupplySimAreaWidth();
01989   //int my=m_TheLandscape->SupplySimAreaHeight();
01990   //int gridcount[25][25]; // NB This is dangerous, do not make bigger maps than can fit in here!
01991   for (int i=0; i<25; i++) for (int j=0; j<25; j++) gridcount[i][j]=0;
01992   for (unsigned j=0; j<totalF; j++)      //adult females 
01993   { 
01994           FS=dynamic_cast<Vole_Female*>(TheArray[1][j]);
01995           if (FS->SupplyTerritorial()) {
01996                   x=FS->Supply_m_Location_x(); 
01997                   y=FS->Supply_m_Location_y(); 
01998                   int gx=x/__CIPEGRIDSIZE; // 500m grids is default
01999                   int gy=y/__CIPEGRIDSIZE;
02000                   gridcount[gx][gy]++;
02001           }
02002   }
02003   for (int i=0; i<20; i++) for (int j=0; j<20; j++) {
02004           float res=float(gridcount[i][j]);
02005           fprintf(CIPEGridOutputPrb,"%f\t",res);
02006   }
02007   fprintf(CIPEGridOutputPrb,"\n");
02008   fflush(CIPEGridOutputPrb);
02009 }
```

|  |  |  |  |  |
| --- | --- | --- | --- | --- |
| void Vole\_Population\_Manager::TheReallyBigOutputProbe | ( |  | ) | `[virtual]` |

This method must be overridden in descendent classes

Reimplemented from Population\_Manager.

References Vole\_Base::GetDirectFlag(), Vole\_Base::GetGeneticFlag(), Population\_Manager::m\_TheLandscape, Population\_Manager::ReallyBigOutputPrb, TAnimal::Supply\_m\_Location\_x(), TAnimal::Supply\_m\_Location\_y(), Vole\_Base::SupplyAge(), Vole\_Base::SupplyTerritorial(), and Population\_Manager::TheArray.

```
02030                                                       {
02032   Vole_Female* FV; 
02033   Vole_Male* MV; 
02034   unsigned int totalM= (unsigned int) TheArray[0].size();
02035   int x,y;
02036   int w = m_TheLandscape->SupplySimAreaWidth(); 
02037   int h = m_TheLandscape->SupplySimAreaWidth();
02038   fprintf(ReallyBigOutputPrb,"%d %d %d %d %d\n", 0,w ,0, h, totalM+TheArray[1].size());
02039   for (unsigned j=0; j<totalM; j++)      //adult females 
02040   { 
02041           MV=dynamic_cast<Vole_Male*>(TheArray[0][j]);
02042           x=MV->Supply_m_Location_x(); 
02043           y=MV->Supply_m_Location_y(); 
02044           int poly=m_TheLandscape->SupplyPolyRef(x,y);
02045           int ele=m_TheLandscape->SupplyElementType(poly);
02046           int vegt=m_TheLandscape->SupplyVegType(poly);
02047           int GA=MV->GetGeneticFlag();
02048           int DA=MV->GetDirectFlag();
02049           int Age=MV->SupplyAge();
02050           int Ter=(int)MV->SupplyTerritorial();
02051           fprintf(ReallyBigOutputPrb,"%d\t%d\t%d\t%d\t%d\t%d\t%d\t%d\t%d\t%d\n", x,y,0,poly,ele,vegt,GA,DA,Age,Ter); 
02052   }
02053   // Do the same for females
02054   unsigned int totalF= (unsigned int) TheArray[1].size();
02055   w = m_TheLandscape->SupplySimAreaWidth(); 
02056   h = m_TheLandscape->SupplySimAreaWidth();
02057   for (unsigned j=0; j<totalF; j++)      //adult females 
02058   { 
02059           FV=dynamic_cast<Vole_Female*>(TheArray[1][j]);
02060           x=FV->Supply_m_Location_x(); 
02061           y=FV->Supply_m_Location_y(); 
02062           int poly=m_TheLandscape->SupplyPolyRef(x,y);
02063           int ele=m_TheLandscape->SupplyElementType(poly);
02064           int vegt=m_TheLandscape->SupplyVegType(poly);
02065           int GA=FV->GetGeneticFlag();
02066           int DA=FV->GetDirectFlag();
02067           int Age=FV->SupplyAge();
02068           int Ter=(int)FV->SupplyTerritorial();
02069           fprintf(ReallyBigOutputPrb,"%d\t%d\t%d\t%d\t%d\t%d\t%d\t%d\t%d\t%d\n", x,y,1,poly,ele,vegt,GA,DA,Age,Ter); 
02070   }
02071   fflush(ReallyBigOutputPrb);
02072 }
```

|  |  |  |  |  |  |
| --- | --- | --- | --- | --- | --- |
| void Vole\_Population\_Manager::TheRipleysOutputProbe | ( | FILE \* | *a\_prb* | ) | `[virtual]` |

This method must be overridden in descendent classes

Reimplemented from Population\_Manager.

References Population\_Manager::m\_TheLandscape, TAnimal::Supply\_m\_Location\_x(), TAnimal::Supply\_m\_Location\_y(), and Population\_Manager::TheArray.

```
02012                                                                  {
02013   Vole_Female* FS; 
02014   unsigned int totalF= (unsigned int) TheArray[1].size();
02015   int x,y;
02016   int w = m_TheLandscape->SupplySimAreaWidth(); 
02017   int h = m_TheLandscape->SupplySimAreaWidth();
02018   fprintf(a_prb,"%d %d %d %d %d\n", 0,w ,0, h, totalF);
02019   for (unsigned j=0; j<totalF; j++)      //adult females 
02020   { 
02021           FS=dynamic_cast<Vole_Female*>(TheArray[1][j]);
02022           x=FS->Supply_m_Location_x(); 
02023           y=FS->Supply_m_Location_y(); 
02024           fprintf(a_prb,"%d\t%d\n", x,y); 
02025   }
02026   fflush(a_prb);
02027 }
```

---

## Member Data Documentation

|  |
| --- |
| AlleleFreq\* Vole\_Population\_Manager::AlFreq |

Reimplemented from Population\_Manager.

Referenced by Init(), Vole\_Population\_Manager(), and ~Vole\_Population\_Manager().

|  |
| --- |
| Vole\_FemaleList Vole\_Population\_Manager::FList |

Used to store lists of females for further use

Referenced by ListClosestFemales().

|  |
| --- |
| unsigned Vole\_Population\_Manager::IDNumber |

Referenced by Vole\_Base::Vole\_Base(), and Vole\_Population\_Manager().

|  |
| --- |
| int Vole\_Population\_Manager::JuvsProducedToday |

Referenced by AddToJuvs(), DoFirst(), and ReproductionProbe().

|  |
| --- |
| int Vole\_Population\_Manager::m\_f1sterilitychance `[protected]` |

Referenced by CreateObjects(), and Init().

|  |
| --- |
| int Vole\_Population\_Manager::m\_geneticimpacted `[protected]` |

Referenced by AddToGeneticImpacted(), DoFirst(), and Init().

|  |
| --- |
| int Vole\_Population\_Manager::m\_geneticproductfertilityeffect |

Referenced by Init(), and Vole\_Female::st\_Mating().

|  |
| --- |
| int Vole\_Population\_Manager::m\_geneticsterilitychance `[protected]` |

Referenced by CreateObjects(), and Init().

|  |
| --- |
| int Vole\_Population\_Manager::m\_GrowthStartDate `[protected]` |

Referenced by DoFirst(), SupplyGrowthStartDate(), and Vole\_Population\_Manager().

|  |
| --- |
| int Vole\_Population\_Manager::m\_impacted `[protected]` |

Referenced by AddToImpacted(), DoFirst(), and Init().

|  |
| --- |
| int Vole\_Population\_Manager::m\_notimpacted `[protected]` |

Referenced by AddToNotImpacted(), DoFirst(), and Init().

|  |
| --- |
| Vole\_MaleList Vole\_Population\_Manager::MList |

Used to store lists of males for further use

Referenced by ListClosestMales().

|  |
| --- |
| int Vole\_Population\_Manager::ReproTable[4][12] |

Referenced by Init(), Vole\_Female::st\_GiveBirth(), and Vole\_Base::Vole\_Base().

|  |
| --- |
| FILE\* Vole\_Population\_Manager::TestFile |

Reimplemented from Population\_Manager.

Referenced by Init(), and ~Vole\_Population\_Manager().

|  |
| --- |
| FILE\* Vole\_Population\_Manager::TestFile2 |

Reimplemented from Population\_Manager.

Referenced by Init(), and ~Vole\_Population\_Manager().

|  |
| --- |
| int Vole\_Population\_Manager::thisYear `[protected]` |

Referenced by DoFirst(), and Vole\_Population\_Manager().

|  |
| --- |
| IDMap\* Vole\_Population\_Manager::VoleMap |

Referenced by CreateObjects(), FindClosestFemale(), FindClosestMale(), Vole\_Male::FreeLocation(), Vole\_Female::FreeLocation(), Vole\_Male::GetLocation(), Vole\_Female::GetLocation(), ListClosestFemales(), ListClosestMales(), Vole\_Base::MoveQuality(), Vole\_Male::SetLocation(), Vole\_Female::SetLocation(), SupplyHowManyVoles(), SupplyInOlderTerr(), SupplyOlderFemales(), SupplyVoleList(), Vole\_Population\_Manager(), and ~Vole\_Population\_Manager().

|  |
| --- |
| int Vole\_Population\_Manager::YearsTotal `[protected]` |

Referenced by DoFirst(), and Vole\_Population\_Manager().

|  |
| --- |
| int Vole\_Population\_Manager::YoungProducedToday |

Referenced by AddToYoung(), DoFirst(), and ReproductionProbe().

|  |
| --- |
| FILE\* Vole\_Population\_Manager::YoungsFile |

Referenced by ReproductionProbe().

---

The documentation for this class was generated from the following files:

- VolePopulationManager.H- VolePopulationManager.cpp

---

Generated on Thu Jan 22 14:13:48 2009 for ALMaSS ODDox by 
 1.5.6 
