# Supplementary material for: Predation and fragmentation portrayed in the statistical structure of prey time series
Source: BMC Ecol. 2009 May 6;9:10. doi: 10.1186/1472-6785-9-10 (PMC2689204; doi:10.1186/1472-6785-9-10)
Supplement: Additional file 2 — Voles and related classes ODDox Documentation. ODDox documentation of the agent-based model (ALMaSS) applied by Hendrichsen et al. The documentation is started by activating main.html. [file 1472-6785-9-10-S2.zip › Vole_ODDox/class_weasel.html]

ALMaSS ODDox: Weasel Class Reference

- Main Page
- Related Pages
- Classes
- Files

- Alphabetical List
- Class List
- Class Hierarchy
- Class Members

# Weasel Class Reference

`#include <Predators.H>`

Inheritance diagram for Weasel:

List of all members.

---

## Detailed Description

The Weasel class is one of two current implementations of TPredator.

It is configurable via config parameters and in other than name and default configuration it is identical to the Owl class

|  |
| --- |
|  |
| Public Member Functions | |
| virtual void | BeginStep (void) |
| virtual void | EndStep (void) |
| virtual void | Step (void) |
|  | Weasel (Vole\_Population\_Manager \*ThePrey, int p\_x, int p\_y, Landscape \*p\_L, TPredator\_Population\_Manager \*p\_PPM) |
|  | ~Weasel () |

---

## Constructor & Destructor Documentation

|  |  |  |  |
| --- | --- | --- | --- |
| Weasel::Weasel | ( | Vole\_Population\_Manager \* | *ThePrey*, |
|  |  | int | *p\_x*, |
|  |  | int | *p\_y*, |
|  |  | Landscape \* | *p\_L*, |
|  |  | TPredator\_Population\_Manager \* | *p\_PPM* |  |
|  | ) |  |  |  |

References cfg\_weasel\_DispersalMax(), cfg\_weasel\_home\_range(), cfg\_weasel\_kill\_efficiency(), cfg\_weasel\_NoFailuresBeforeDispersal(), cfg\_weasel\_search\_area(), TPredator::m\_DispersalMax, TPredator::m\_HomeRange, TPredator::m\_KillEfficiency, TPredator::m\_NoFailuresBeforeDispersal, TPredator::m\_SearchArea, TPredator::PreyResponse1, TPredator::PreyResponse2, and TPredator::SpeciesID.

```
00348                                           : TPredator(ThePrey,p_x,p_y,p_L,p_PPM)
00349 {
00350     SpeciesID=0;
00351     m_KillEfficiency=cfg_weasel_kill_efficiency.value();
00352     m_HomeRange=cfg_weasel_home_range.value();
00353     m_SearchArea=cfg_weasel_search_area.value();
00354     m_DispersalMax=cfg_weasel_DispersalMax.value();
00355     m_NoFailuresBeforeDispersal=cfg_weasel_NoFailuresBeforeDispersal.value();
00356     PreyResponse1=1;
00357     PreyResponse2=1;
00358 }
```

|  |  |  |  |  |
| --- | --- | --- | --- | --- |
| Weasel::~Weasel | ( |  | ) |  |

```
00363 {
00364     //Nothing to do
00365 }
```

---

## Member Function Documentation

|  |  |  |  |  |  |
| --- | --- | --- | --- | --- | --- |
| void Weasel::BeginStep | ( | void |  | ) | `[virtual]` |

Reimplemented from TPredator.

References TPredator\_Population\_Manager::CreateObjects(), TPredator\_Population\_Manager::dec\_inds(), TAnimal::KillThis(), struct\_Predator::L, TPredator::m\_kills\_this\_season, TAnimal::m\_Location\_x, TAnimal::m\_Location\_y, TAnimal::m\_OurLandscape, TPredator::m\_OurPopulationManager, struct\_Predator::PM, TPredator\_Population\_Manager::supply\_no\_inds(), WEASEL, weasel\_breed\_day, weasel\_breed\_threshold, weasel\_death\_threshold, struct\_Predator::x, and struct\_Predator::y.

```
00369 {
00370   int day= m_OurLandscape->SupplyDayInYear();
00371   if (day==weasel_breed_day)
00372   {
00373     int noToMake=m_kills_this_season/weasel_breed_threshold;
00374     for (int k=0; k<noToMake; k++)
00375     {
00376       // make a new weasel
00377       struct_Predator* sp;
00378       sp = new struct_Predator;
00379       sp->PM = m_OurPopulationManager;
00380       sp->L = m_OurLandscape;
00381       sp->x = m_Location_x;
00382       sp->y = m_Location_y;
00383       m_OurPopulationManager->CreateObjects(0,NULL,sp,1); // 0 = weasel
00384       delete sp;
00385     }
00386   }
00387   else if (day==364)
00388   {
00389     if (m_kills_this_season<weasel_death_threshold)
00390     {
00391       if (m_OurPopulationManager->supply_no_inds(WEASEL)>1)
00392       {
00393         m_OurPopulationManager->dec_inds(WEASEL);
00394         KillThis();
00395       }
00396     }
00397     m_kills_this_season=0; // reset the count
00398   }
00399 }
```

|  |  |  |  |  |  |
| --- | --- | --- | --- | --- | --- |
| virtual void Weasel::EndStep | ( | void |  | ) | `[inline, virtual]` |

Reimplemented from TPredator.

```
00171 {}
```

|  |  |  |  |  |  |
| --- | --- | --- | --- | --- | --- |
| void Weasel::Step | ( | void |  | ) | `[virtual]` |

Reimplemented from TPredator.

References TPredator::CurrentPState, TALMaSSObject::CurrentStateNo, TPredator::m\_FailureCount, TPredator::m\_HaveTerritory, TPredator::m\_NoFailuresBeforeDispersal, TPredator::PreyResponse1, TPredator::PreyResponse2, TPredator::st\_Dispersal(), TPredator::st\_Hunting(), TPredator::st\_Movement(), TALMaSSObject::StepDone, tops\_Dispersal, tops\_Hunting, tops\_InitialState, and tops\_Movement.

```
00403 {
00404   if (StepDone || CurrentStateNo == -1) return;
00405   switch (CurrentPState)
00406   {
00407    case tops_InitialState: // Initial state
00408     CurrentPState=tops_Dispersal;
00409     m_HaveTerritory=false;
00410     break;
00411    case tops_Hunting:
00412     if (st_Hunting()<PreyResponse1) CurrentPState=tops_Movement;
00413     StepDone=true;
00414     break;
00415    case tops_Dispersal:
00416     st_Dispersal();
00417     if (m_HaveTerritory) CurrentPState=tops_Hunting;
00418     StepDone=true;
00419     break;
00420    case tops_Movement:
00421     st_Movement();
00422     if (st_Hunting()<PreyResponse2)   // alter this figure to increase functional response
00423      m_FailureCount++;
00424     else m_FailureCount=0;
00425     if (m_FailureCount>m_NoFailuresBeforeDispersal)
00426     {
00427      m_HaveTerritory=false;
00428      CurrentPState=tops_Dispersal;
00429     }
00430     else CurrentPState=tops_Hunting;
00431     StepDone=true;
00432     break;
00433    default:
00434     exit(1);
00435    }
00436 }
```

---

The documentation for this class was generated from the following files:

- Predators.H- Predators.cpp

---

Generated on Thu Jan 22 14:13:48 2009 for ALMaSS ODDox by 
 1.5.6 
