# Supplementary material for: Predation and fragmentation portrayed in the statistical structure of prey time series
Source: BMC Ecol. 2009 May 6;9:10. doi: 10.1186/1472-6785-9-10 (PMC2689204; doi:10.1186/1472-6785-9-10)
Supplement: Additional file 2 — Voles and related classes ODDox Documentation. ODDox documentation of the agent-based model (ALMaSS) applied by Hendrichsen et al. The documentation is started by activating main.html. [file 1472-6785-9-10-S2.zip › Vole_ODDox/class_winter_wheat-members.html]

ALMaSS ODDox: Member List

- Main Page
- Related Pages
- Classes
- Files

- Alphabetical List
- Class List
- Class Hierarchy
- Class Members

# WinterWheat Member List

This is the complete list of members for WinterWheat, including all inherited members.

|  |  |  |
| --- | --- | --- |
| Do(Farm \*a\_farm, LE \*a\_field, FarmEvent \*a\_ev) | WinterWheat | `[virtual]` |
| GetFirstDate(void) | Crop | `[inline]` |
| m\_count | Crop | `[protected]` |
| m\_ev | Crop | `[protected]` |
| m\_farm | Crop | `[protected]` |
| m\_field | Crop | `[protected]` |
| m\_first\_date | Crop | `[protected]` |
| m\_last\_date | Crop | `[protected]` |
| SimpleEvent(long a\_date, int a\_todo, bool a\_lock) | Crop | `[protected]` |
| WinterWheat() | WinterWheat | `[inline]` |
| ~Crop() | Crop | `[inline, virtual]` |

---

Generated on Thu Jan 22 14:13:48 2009 for ALMaSS ODDox by 
 1.5.6 
