# Supplementary material for: Predation and fragmentation portrayed in the statistical structure of prey time series
Source: BMC Ecol. 2009 May 6;9:10. doi: 10.1186/1472-6785-9-10 (PMC2689204; doi:10.1186/1472-6785-9-10)
Supplement: Additional file 2 — Voles and related classes ODDox Documentation. ODDox documentation of the agent-based model (ALMaSS) applied by Hendrichsen et al. The documentation is started by activating main.html. [file 1472-6785-9-10-S2.zip › Vole_ODDox/class_winter_wheat.html]

ALMaSS ODDox: WinterWheat Class Reference

- Main Page
- Related Pages
- Classes
- Files

- Alphabetical List
- Class List
- Class Hierarchy
- Class Members

# WinterWheat Class Reference

`#include <WinterWheat.H>`

Inheritance diagram for WinterWheat:

List of all members.

---

## Detailed Description

WinterWheat class   
.

See WinterWheat.h::WinterWheatToDo for a complete list of all possible events triggered codes by the winter wheat management plan. When triggered these events are handled by Farm and are available as information for other objects such as animal and bird models.

|  |
| --- |
|  |
| Public Member Functions | |
| virtual bool | Do (Farm \*a\_farm, LE \*a\_field, FarmEvent \*a\_ev) |
|  | The one and only method for a crop management plan. All farm actions go through here. |
|  | WinterWheat () |

---

## Constructor & Destructor Documentation

|  |  |  |  |  |
| --- | --- | --- | --- | --- |
| WinterWheat::WinterWheat | ( |  | ) | `[inline]` |

References Crop::m\_first\_date.

```
00118    {
00119                 // When we start it off, the first possible date for a farm operation is 1st October
00120                 // This information is used by other crops when they decide how much post processing of 
00121                 // the management is allowed after harvest before the next crop starts.
00122                 m_first_date=g_date->DayInYear( 1,10 );
00123    }
```

---

## Member Function Documentation

|  |  |  |  |
| --- | --- | --- | --- |
| bool WinterWheat::Do | ( | Farm \* | *a\_farm*, |
|  |  | LE \* | *a\_field*, |
|  |  | FarmEvent \* | *a\_ev* |  |
|  | ) |  |  | `[virtual]` |

The one and only method for a crop management plan. All farm actions go through here.

Called every time something is done to the crop by the farmer in the first instance it is always called with m\_ev->todo set to start, but susequently will be called whenever the farmer wants to carry out a new operation.   
This method details all the management and relationships between operations necessary to grow and ALMaSS crop - in this case conventional winter wheat.

Reimplemented from Crop.

References Farm::AutumnHarrow(), Farm::AutumnPlough(), Farm::AutumnRoll(), Farm::AutumnSow(), cfg\_fungi\_app\_prop, cfg\_greg\_app\_prop, cfg\_herbi\_app\_prop, cfg\_ins\_app\_prop, Farm::DeepPlough(), Farm::DoIt(), Farm::FA\_Manure(), Farm::FA\_NPK(), Farm::FA\_Slurry(), Farm::FP\_ManganeseSulphate(), Farm::FP\_NPK(), Farm::FP\_Slurry(), Farm::FungicideTreat(), Farm::GrowthRegulator(), Farm::Harvest(), Farm::HayBailing(), Farm::HayTurning(), Farm::HerbicideTreat(), Farm::InsecticideTreat(), Farm::IsStockFarmer(), Crop::m\_ev, Crop::m\_farm, Crop::m\_field, Crop::m\_first\_date, FarmEvent::m\_first\_year, FarmEvent::m\_lock, FarmEvent::m\_startday, FarmEvent::m\_todo, Crop::SimpleEvent(), Farm::SpringRoll(), Farm::StrawChopping(), Farm::Strigling(), Farm::StubbleHarrowing(), Farm::Water(), ww\_autumn\_harrow, ww\_autumn\_plough, WW\_AUTUMN\_PLOUGH, ww\_autumn\_roll, ww\_autumn\_sow, ww\_ferti\_p1, ww\_ferti\_p2, ww\_ferti\_p3, ww\_ferti\_p4, ww\_ferti\_p5, ww\_ferti\_s1, ww\_ferti\_s2, ww\_ferti\_s3, ww\_ferti\_s4, ww\_ferti\_s5, ww\_fungicide, ww\_fungicide2, ww\_GR, ww\_grubning, ww\_harvest, ww\_hay\_baling, ww\_hay\_turning, ww\_herbicide1, ww\_herbicide2, ww\_insecticide1, ww\_insecticide2, ww\_insecticide3, ww\_spring\_roll, ww\_start, ww\_straw\_chopping, ww\_strigling1, ww\_strigling2, ww\_stubble\_harrow1, ww\_stubble\_harrow2, ww\_water1, and ww\_water2.

Referenced by Farm::LeSwitch().

```
00070 {
00071   m_farm  = a_farm;
00072   m_field = a_field;
00073   m_ev    = a_ev;
00074   // The boolean value done indicates when we are totally finished with this plan
00075   bool done = false;
00076   // Depending what event has occured jump to the correct bit of code e.g. for ww_start jump to line 67 below
00077   switch ( m_ev->m_todo )
00078   {
00079     case ww_start:
00080                 { // This is just to hold a local variable in scope and prevent compiler errors
00081                         // ww_start just sets up all the starting conditions and reference dates that are needed to start a ww 
00082                         // crop off
00083                         WW_AUTUMN_PLOUGH         = false;
00084                         // Set up the date management stuff
00085                         // The next bit of code just allows for altering dates after harvest if it is necessary
00086                         // to allow for a crop which starts its management early.
00087 
00088                         // 5 start and stop dates for all events after harvest for this crop
00089                         int noDates=5;
00090                         m_field->SetMDates(0,0,g_date->DayInYear(20,8));
00091                         // Determined by harvest date - used to see if at all possible
00092                         m_field->SetMDates(1,0,g_date->DayInYear(20,8));
00093                         m_field->SetMDates(0,1,0); // Subbleharrow start
00094                         m_field->SetMDates(1,1,g_date->DayInYear(20,8));
00095                         m_field->SetMDates(0,2,g_date->DayInYear(5,8));
00096                         m_field->SetMDates(1,2,g_date->DayInYear(25,8));
00097                         m_field->SetMDates(0,3,g_date->DayInYear(10,8));
00098                         m_field->SetMDates(1,3,g_date->DayInYear(15,9));
00099                         m_field->SetMDates(0,4,g_date->DayInYear(15,8));
00100                         m_field->SetMDates(1,4,g_date->DayInYear(15,10));
00101                         // Can be up to 10 of these. If the shortening code is triggered
00102                         // then these will be reduced in value to 0
00103                         m_field->SetMConstants(0,1);
00104                         // Check the next crop for early start, unless it is a spring crop
00105                         // in which case we ASSUME that no checking is necessary!!!!
00106                         // So DO NOT implement a crop that runs over the year boundary (i.e. from spring to spring!), at least not without fixing this.
00107                         if (m_ev->m_startday>g_date->DayInYear(1,7)) {
00108                                 if (m_field->GetMDates(0,0) >=m_ev->m_startday)
00109                                 {
00110                                   g_msg->Warn( WARN_BUG, "WinterWheat::Do(): ","Harvest too late for the next crop to start!!!" );
00111                                   exit( 1 );
00112                                 }
00113                                 // Now fix any late finishing problems
00114                                 bool toggle=false;
00115                                 for (int i=0; i<noDates; i++) {
00116                                   if  (m_field->GetMDates(0,i)>=m_ev->m_startday) {
00117                                         toggle=true;
00118                                         m_field->SetMDates(0,i,m_ev->m_startday-1);
00119                                   }
00120                                   if  (m_field->GetMDates(1,i)>=m_ev->m_startday){
00121                                         toggle=true;
00122                                         m_field->SetMDates(1,i,m_ev->m_startday-1);
00123                                   }
00124                                 }
00125                                 if (toggle) for (int i=0; i<10; i++) m_field->SetMConstants(i,0);
00126                         }
00127                         // Now no operations can be timed after the start of the next crop.
00128                         int d1;
00129                         if ( ! m_ev->m_first_year ) {
00130                                 // Are we before July 1st?
00131                                 d1 = g_date->OldDays() + g_date->DayInYear( 1,7 );
00132                                 if (g_date->Date() < d1 ) {
00133                                 // Yes, too early. We assumme this is because the last crop was late
00134                                 printf ("Poly: %d\n", m_field->GetPoly());
00135                                   g_msg->Warn( WARN_BUG, "WinterWheat::Do(): ","Crop start attempt between 1st Jan & 1st July" );
00136                                   exit( 1 );
00137                                 }
00138                                 else {
00139                                          d1 = g_date->OldDays() + m_first_date; // Add 365 for spring crop
00140                                          if (g_date->Date() > d1) {
00141                                          // Yes too late - should not happen - raise an error
00142                                          g_msg->Warn( WARN_BUG, "WinterWheat::Do(): ", "Crop start attempt after last possible start date" );
00143                                          exit( 1 );
00144                                    }
00145                                 }
00146                         }
00147                         else {
00148                                 // Is the first year
00149                                 // Some special code to cope with that first start-up year in ALMaSS - ignore for all practical purposes
00150                                 SimpleEvent( g_date->OldDays() + g_date->DayInYear( 1,4 ), ww_spring_roll, false );
00151                                 break;
00152                         }
00153                         // End single block date checking code. Please see next line comment as well.
00154                         // Reinit d1 to first possible starting date.
00155                         d1 = g_date->OldDays() + g_date->DayInYear( 21,8 );
00156                         // OK, let's go.
00157                         // Here we queue up the first event - this differs depending on whether we have a
00158                         // stock or arable farmer
00159                         if (m_farm->IsStockFarmer()) { // StockFarmer
00160                                 SimpleEvent( d1, ww_ferti_s1, false );
00161                         }
00162                         else SimpleEvent( d1, ww_ferti_p1, false ); 
00163                 }
00164                 break;
00165         // This is the first real farm operation
00166     case ww_ferti_p1:
00167                 // Unless we are forced to do this (probably because we tried once and failed due to bad weather)
00168                 // then 10% of all threads that reach here will attempt to do ww_feri_p1
00169                 if ( m_ev->m_lock || m_farm->DoIt( 10 ))
00170                 {
00171                         // Calls the FP_Slurry application with the number of days after 1,10 as a parameter
00172                         // the FP_Slurry routine will attempt to apply slurry on a probabilistic basis up to 1,10 when
00173                         // the operation will be forced
00174                         // Many other constraints can be added to the FP_Slurry, e.g. temperature might have to be >0 degrees C
00175                         // Other operations need low wind speed (e.g. spraying insecticide). These differ for each operation
00176                         if (!m_farm->FP_Slurry( m_field, 0.0, g_date->DayInYear( 1,10 ) - g_date->DayInYear())) {
00177                                 // If we don't suceed on the first try, then try and try again (until 1/10 when we will suceed)
00178                                 SimpleEvent( g_date->Date() + 1, ww_ferti_p1, true );
00179                                 break;
00180                         }
00181                 }
00182                 // Queue up the next event -in this case autumn ploughing
00183                 SimpleEvent( g_date->Date(),ww_autumn_plough, false );
00184                 break;
00185         case ww_ferti_s1:
00186             if (!m_farm->FA_Slurry( m_field, 0.0, g_date->DayInYear( 1,10 ) - g_date->DayInYear())) {
00187                   SimpleEvent( g_date->Date() + 1, ww_ferti_s1, true );
00188                   break;
00189                 }
00190                 // This may cause two applications of fertilizer in one day...which is OK because one is Slurry and one Manure
00191                 SimpleEvent( g_date->Date(),ww_ferti_s2, false );
00192                 break;
00193         case ww_ferti_s2:
00194                 if ( m_ev->m_lock || m_farm->DoIt( 10 ))
00195                 {
00196                   if (!m_farm->FA_Manure( m_field, 0.0,
00197                            g_date->DayInYear( 1,10 ) - g_date->DayInYear())) {
00198                         SimpleEvent( g_date->Date() + 1, ww_ferti_s2, true );
00199                         break;
00200                   }
00201                 }
00202                 SimpleEvent( g_date->Date(),ww_autumn_plough, false );
00203                 break;
00204         case ww_autumn_plough:
00205                 // Almost all will autumn plough, but a few will get away with non-inversion
00206                 if ( m_ev->m_lock || m_farm->DoIt( 95 ))
00207                 {
00208                   if (!m_farm->AutumnPlough( m_field, 0.0,
00209                            g_date->DayInYear( 1,10 ) - g_date->DayInYear())) {
00210                         SimpleEvent( g_date->Date() + 1, ww_autumn_plough, true );
00211                         break;
00212                   }
00213                   else
00214                   {
00215                           // 95% of farmers will do this, but the other 5% won't so we need to remember whether 
00216                           // we are in one or the other group
00217                         WW_AUTUMN_PLOUGH=true;
00218                         // Queue up the next event
00219                         SimpleEvent( g_date->Date()+1,ww_autumn_harrow, false );
00220                         break;
00221                   }
00222                 }
00223                 SimpleEvent( g_date->Date()+1,ww_stubble_harrow1, false );
00224                 break;
00225         case ww_autumn_harrow:
00226                 if (!m_farm->AutumnHarrow( m_field, 0.0,
00227                            g_date->DayInYear( 10,10 ) - g_date->DayInYear())) {
00228                   SimpleEvent( g_date->Date() + 1, ww_autumn_harrow, true );
00229                   break;
00230                 }
00231                 SimpleEvent( g_date->OldDays() + g_date->DayInYear( 10,9 ),
00232                                    ww_autumn_sow, false );
00233                 break;
00234         case ww_stubble_harrow1:
00235                 if (!m_farm->StubbleHarrowing( m_field, 0.0,
00236                            g_date->DayInYear( 10,10 ) - g_date->DayInYear())) {
00237                   SimpleEvent( g_date->Date() + 1, ww_stubble_harrow1, true );
00238                   break;
00239                 }
00240                 SimpleEvent( g_date->OldDays() + g_date->DayInYear( 10,9 ),
00241                                    ww_autumn_sow, false );
00242                 break;
00243         case ww_autumn_sow:
00244                 if (!m_farm->AutumnSow( m_field, 0.0,
00245                            g_date->DayInYear( 20,10 ) - g_date->DayInYear())) {
00246                   SimpleEvent( g_date->Date() + 1, ww_autumn_sow, true );
00247                   break;
00248                 }
00249                 SimpleEvent( g_date->Date() + 1, ww_autumn_roll, false );
00250                 break;
00251         case ww_autumn_roll:
00252                 // If they did plough, then 5% will also do an autumn rolling
00253                 if (( m_ev->m_lock || m_farm->DoIt( 5 ))&& (WW_AUTUMN_PLOUGH))
00254                 {
00255                   if (!m_farm->AutumnRoll( m_field, 0.0,
00256                            g_date->DayInYear( 27,10 ) - g_date->DayInYear())) {
00257                         SimpleEvent( g_date->Date() + 1, ww_autumn_roll, true );
00258                         break;
00259                   }
00260                 }
00261                 SimpleEvent( g_date->OldDays() + g_date->DayInYear( 15,9 ),
00262                                                   ww_ferti_p2, false );
00263                 break;
00264                 // Plant farmers start here.
00265         case ww_ferti_p2:
00266                 if (( m_ev->m_lock || m_farm->DoIt( 20 )) && (!m_farm->IsStockFarmer()))
00267                 {
00268                   if ( m_field->GetVegBiomass()>0)
00269                   //only apply this when there has been a bit of growth
00270                   {
00271                         if (!m_farm->FP_ManganeseSulphate( m_field, 0.0,
00272                            g_date->DayInYear( 30,10 ) - g_date->DayInYear()))
00273                         {
00274                           SimpleEvent( g_date->Date() + 1, ww_ferti_p2, true );
00275                           break;
00276                         }
00277                   }
00278                 }
00279                 SimpleEvent( g_date->OldDays() + g_date->DayInYear( 20,9 ),
00280                                                   ww_herbicide1, false );
00281                 break;
00282         case ww_herbicide1: // The first of the pesticide managements. NB these are externally scalable
00283                 if ( m_ev->m_lock || m_farm->DoIt( (int) ( 100*cfg_herbi_app_prop.value() )))
00284                 {
00285                   if (!m_farm->HerbicideTreat( m_field, 0.0,
00286                                  g_date->DayInYear( 5,10 ) - g_date->DayInYear())) {
00287                         SimpleEvent( g_date->Date() + 1, ww_herbicide1, true );
00288                         break;
00289                   }
00290                 }
00291                 SimpleEvent( g_date->OldDays() + g_date->DayInYear( 1,4 )+365,
00292                                  ww_spring_roll, false );
00293                 break;
00294         case ww_spring_roll:
00295                 if ( m_ev->m_lock || m_farm->DoIt( 5 ))
00296                 {
00297                   if (!m_farm->SpringRoll( m_field, 0.0,
00298                            g_date->DayInYear( 30,4 ) - g_date->DayInYear())) {
00299                         SimpleEvent( g_date->Date() + 1, ww_spring_roll, true );
00300                         break;
00301                   }
00302                 }
00303                 // Here we split the main thread in to depending on what fertilizer the farmer will use
00304                 // these rejoin other later on.
00305                 if (m_farm->IsStockFarmer()) // StockFarmer
00306                 {
00307                   SimpleEvent( g_date->Date() + 1, ww_ferti_s3, false );
00308                   SimpleEvent( g_date->OldDays() + g_date->DayInYear( 10,4 ), ww_ferti_s4, false );
00309                 }
00310                 else SimpleEvent( g_date->OldDays() + g_date->DayInYear( 25,3 ),ww_ferti_p3, false );
00311                 // Here we see an example of setting up parallel threads
00312                 // None of these are the main thread which will lead to the termination of the plan - they 
00313                 // just run along until they peter out e.g. ww_herbicide2
00314                 // All paths through the plan need the next threads
00315                 SimpleEvent( g_date->OldDays() + g_date->DayInYear( 15,4 ),
00316                                  ww_herbicide2, false );
00317                 SimpleEvent( g_date->OldDays() + g_date->DayInYear( 25,4 ),
00318                                  ww_GR, false );
00319                 SimpleEvent( g_date->OldDays() + g_date->DayInYear( 20,4 ),
00320                                  ww_fungicide, false );
00321                 SimpleEvent( g_date->OldDays() + g_date->DayInYear( 1,5 ),
00322                                  ww_insecticide1, false );
00323                 SimpleEvent( g_date->OldDays() + g_date->DayInYear( 10,4 ),
00324                                  ww_strigling1, false );
00325                 SimpleEvent( g_date->OldDays() + g_date->DayInYear( 1,5 ),
00326                                  ww_water1, false );
00327                 break;
00328         case ww_herbicide2:
00329                 if ( m_ev->m_lock || m_farm->DoIt( (int) ( (int) ( 100*cfg_herbi_app_prop.value() )))) // was 40
00330                 {
00331                   if (!m_farm->HerbicideTreat( m_field, 0.0,
00332                                  g_date->DayInYear( 30,4 ) - g_date->DayInYear())) {
00333                         SimpleEvent( g_date->Date() + 1, ww_herbicide2, true );
00334                         break;
00335                   }
00336                 }
00337                 // End of thread
00338                 break;
00339         case ww_GR:
00340                 if ( m_ev->m_lock || m_farm->DoIt( (int) ( 15*cfg_greg_app_prop.value() )))
00341                 {
00342                   if (!m_farm->GrowthRegulator( m_field, 0.0,
00343                                  g_date->DayInYear( 10,5 ) - g_date->DayInYear())) {
00344                         SimpleEvent( g_date->Date() + 1, ww_GR, true );
00345                         break;
00346                   }
00347                 }
00348                 // End of thread
00349                 break;
00350         case ww_fungicide:
00351                 if ( m_ev->m_lock || m_farm->DoIt( (int) ( 70*cfg_fungi_app_prop.value()) ))
00352                 {
00353                   if (!m_farm->FungicideTreat( m_field, 0.0,
00354                                  g_date->DayInYear( 10,5 ) - g_date->DayInYear())) {
00355                         SimpleEvent( g_date->Date() + 1, ww_fungicide, true );
00356                         break;
00357                   }
00358                 }
00359                 SimpleEvent( g_date->OldDays() + g_date->DayInYear( 20,5 ),ww_fungicide2, false );
00360                 break;
00361 
00362         case ww_fungicide2:
00363                 if ( m_ev->m_lock || m_farm->DoIt( (int) ( 50*cfg_fungi_app_prop.value()) ))
00364                 {
00365                   if (!m_farm->FungicideTreat( m_field, 0.0,
00366                                  g_date->DayInYear( 15,5 ) - g_date->DayInYear())) {
00367                         SimpleEvent( g_date->Date() + 1, ww_fungicide2, true );
00368                         break;
00369                   }
00370                 }
00371                 // End of thread
00372                 break;
00373                 // Note that for special pesticide cases this code would be de-defined to call another specific spraying event instead of InsecticideTreat() which is a generic function.
00374         case ww_insecticide1:
00375                 if ( m_ev->m_lock || m_farm->DoIt( (int) ( 16*cfg_ins_app_prop.value() )))
00376                 {
00377                   if (!m_farm->InsecticideTreat( m_field, 0.0,g_date->DayInYear( 15,5 ) - g_date->DayInYear())) {
00378                         SimpleEvent( g_date->Date() + 1, ww_insecticide1, true );
00379                         break;
00380                   }
00381                   else {
00382                           SimpleEvent( g_date->OldDays() + g_date->DayInYear( 1,6 ),ww_insecticide2, false );
00383                           break;
00384                         }
00385                 }
00386                 break;
00387         case ww_insecticide2:
00388                 if ( m_ev->m_lock || m_farm->DoIt( (int) ( 33*cfg_ins_app_prop.value() )))
00389                 {
00390                   if (!m_farm->InsecticideTreat( m_field, 0.0, g_date->DayInYear( 10,6 ) - g_date->DayInYear())) {
00391                         SimpleEvent( g_date->Date() + 1, ww_insecticide2, true );
00392                         break;
00393                   }
00394                   else {
00395                         if ((g_date->Date()+7)<( g_date->OldDays() + g_date->DayInYear( 15,6 )))
00396                           SimpleEvent( g_date->OldDays() + g_date->DayInYear( 15,6 ), ww_insecticide3, false );
00397                         else SimpleEvent( g_date->Date()+7, ww_insecticide3, false );
00398                         break;
00399                   }
00400                 }
00401         break;
00402         case ww_insecticide3:
00403                 if ( m_ev->m_lock || m_farm->DoIt( (int) ( 67*cfg_ins_app_prop.value() )))
00404                 {
00405                   if (!m_farm->InsecticideTreat( m_field, 0.0,g_date->DayInYear( 30,6 ) - g_date->DayInYear())) {
00406                         SimpleEvent( g_date->Date() + 1, ww_insecticide3, true );
00407                         break;
00408                   }
00409                 }
00410                 // End of thread
00411                 break;
00412         case ww_strigling1:
00413                 if ( m_ev->m_lock || m_farm->DoIt( 5 ))
00414                 {
00415                   if (!m_farm->Strigling( m_field, 0.0,
00416                                  g_date->DayInYear( 25,4 ) - g_date->DayInYear())) {
00417                         SimpleEvent( g_date->Date() + 1, ww_strigling1, true );
00418                         break;
00419                   }
00420                   else {
00421                         if ((g_date->Date()+7)<( g_date->OldDays() + g_date->DayInYear( 15,6 )))
00422                                   SimpleEvent( g_date->OldDays() + g_date->DayInYear( 25,4),
00423                                                    ww_strigling2, false );
00424                         else SimpleEvent( g_date->Date()+7,ww_strigling2, false );
00425                   }
00426                 }
00427                 break;
00428         case ww_strigling2:
00429           if (!m_farm->Strigling( m_field, 0.0,g_date->DayInYear( 5,5 ) - g_date->DayInYear())) {
00430                 SimpleEvent( g_date->Date() + 1, ww_strigling2, true );
00431                 break;
00432           }
00433           // End of thread
00434           break;
00435         case ww_water1:
00436                 if ( m_ev->m_lock || m_farm->DoIt( 10 )) // **CJT** Soil type 1-4 only
00437                 {
00438                   if (!m_farm->Water( m_field, 0.0,g_date->DayInYear( 15,5 ) - g_date->DayInYear())) {
00439                         SimpleEvent( g_date->Date() + 1, ww_water1, true );
00440                         break;
00441                   }
00442                   else
00443                         if ((g_date->Date()+5)<( g_date->OldDays() + g_date->DayInYear( 2,5 )))
00444                                 SimpleEvent( g_date->OldDays() + g_date->DayInYear( 2,5 ),ww_water2, false );
00445                         else SimpleEvent( g_date->Date()+5, ww_water2, false );
00446                 }
00447                 break;
00448         case ww_water2:
00449                 if (!m_farm->Water( m_field, 0.0, g_date->DayInYear( 1,6 ) - g_date->DayInYear())) {
00450                   SimpleEvent( g_date->Date() + 1, ww_water2, true );
00451                   break;
00452                 }
00453                 // End of thread
00454                 break;
00455         case ww_ferti_p3:
00456                 if (!m_farm->FP_NPK( m_field, 0.0,
00457                          g_date->DayInYear( 15,4 ) - g_date->DayInYear())) {
00458                   SimpleEvent( g_date->Date() + 1, ww_ferti_p3, true );
00459                   break;
00460                 }
00461 
00462                 SimpleEvent( g_date->OldDays() + g_date->DayInYear( 25,4 ), ww_ferti_p4, false );
00463                 SimpleEvent( g_date->OldDays() + g_date->DayInYear(  1,4 ), ww_ferti_p5, false );
00464                 break;
00465         case ww_ferti_p4:
00466                 if ( m_ev->m_lock || m_farm->DoIt( 50 ))
00467                 {
00468                   if (!m_farm->FP_NPK( m_field, 0.0, g_date->DayInYear( 15,5 ) - g_date->DayInYear())) {
00469                         SimpleEvent( g_date->Date() + 1, ww_ferti_p4, true );
00470                         break;
00471                   }
00472                 }
00473                 // The Main thread
00474                 // The Main thread - this will leads to harvest, whilst all the others stop before this point
00475                 // This thread is only for arable farmers whereas you'll see below the other forks for stock farmers
00476                 SimpleEvent( g_date->OldDays() + g_date->DayInYear( 5,8 ), ww_harvest, false );
00477                 break;
00478         case ww_ferti_p5:
00479                 if ( m_ev->m_lock || m_farm->DoIt( 20 ))
00480                 {
00481                   if (!m_farm->FP_ManganeseSulphate( m_field, 0.0, g_date->DayInYear( 5,5 ) - g_date->DayInYear())) {
00482                         SimpleEvent( g_date->Date() + 1, ww_ferti_p5, true );
00483                         break;
00484                   }
00485                 }
00486                 break;
00487         case ww_ferti_s3:
00488                 if (!m_farm->FA_Slurry(m_field, 0.0, g_date->DayInYear( 30,4 ) - g_date->DayInYear())) {
00489                   SimpleEvent( g_date->Date() + 1, ww_ferti_s3, true );
00490                   break;
00491                 }
00492                 // The Main thread - this will leads to harvest, whilst all the others stop before this point
00493                 SimpleEvent( g_date->OldDays() + g_date->DayInYear( 5,8 ), ww_harvest, false );
00494                 break;
00495         case ww_ferti_s4:
00496                 if ( m_ev->m_lock || m_farm->DoIt( 75 ))
00497                 {
00498                   if (!m_farm->FA_NPK( m_field, 0.0, g_date->DayInYear( 20,4 ) - g_date->DayInYear())) {
00499                         SimpleEvent( g_date->Date() + 1, ww_ferti_s4, true );
00500                         break;
00501                   }
00502                   SimpleEvent( g_date->OldDays() + g_date->DayInYear( 21,4 ), ww_ferti_s5, false );
00503                 }
00504                 break;
00505         case ww_ferti_s5:
00506                 if ( m_ev->m_lock || m_farm->DoIt( 40 ))
00507                 {
00508                   if (!m_farm->FA_NPK( m_field, 0.0, g_date->DayInYear( 1,5 ) - g_date->DayInYear())) {
00509                         SimpleEvent( g_date->Date() + 1, ww_ferti_s5, true );
00510                         break;
00511                   }
00512                 }
00513                 break;
00514         case ww_harvest:
00515                 if (!m_farm->Harvest( m_field, 0.0, g_date->DayInYear( 20,8 ) - g_date->DayInYear()))
00516                 {
00517                         SimpleEvent( g_date->Date() + 1, ww_harvest, true );
00518                         break;
00519                 }
00520                 SimpleEvent( g_date->Date(), ww_straw_chopping, false );
00521                 break;
00522         case ww_straw_chopping:
00523                 if ( m_ev->m_lock || m_farm->DoIt( 75 ))
00524                 {
00525                   if (!m_farm->StrawChopping( m_field, 0.0, m_field->GetMDates(1,0) - g_date->DayInYear())) {
00526                         SimpleEvent( g_date->Date() + 1, ww_straw_chopping, true );
00527                         break;
00528                   }
00529                   else {
00530                           SimpleEvent( g_date->Date()+m_field->GetMConstants(0), ww_stubble_harrow2, false );
00531                   }
00532                 }
00533                 else {
00534                   SimpleEvent( g_date->Date()+m_field->GetMConstants(0), ww_hay_turning, false );
00535                 }
00536                 break;
00537         case ww_hay_turning:
00538                 if ( m_ev->m_lock || m_farm->DoIt( 5 ))
00539                 {
00540                   if (!m_farm->HayTurning( m_field, 0.0, m_field->GetMDates(1,1) - g_date->DayInYear())) {
00541                         SimpleEvent( g_date->Date() + 1, ww_hay_turning, true );
00542                         break;
00543                   }
00544                 }
00545                 SimpleEvent( g_date->OldDays() + m_field->GetMDates(0,2), ww_hay_baling, false );
00546                 break;
00547         case ww_hay_baling:
00548                 if (!m_farm->HayBailing( m_field, 0.0, m_field->GetMDates(1,2) - g_date->DayInYear())) {
00549                   SimpleEvent( g_date->Date() + 1, ww_hay_baling, true );
00550                   break;
00551                 }
00552                 SimpleEvent( g_date->OldDays() + m_field->GetMDates(0,3), ww_stubble_harrow2, false );
00553                 break;
00554         case ww_stubble_harrow2:
00555                 if ( m_ev->m_lock || m_farm->DoIt( 65 ))
00556                 {
00557                   if (!m_farm->StubbleHarrowing( m_field, 0.0, m_field->GetMDates(1,3) - g_date->DayInYear())) {
00558                         SimpleEvent( g_date->Date() + 1, ww_stubble_harrow2, true );
00559                         break;
00560                   }
00561                 }
00562                 SimpleEvent( g_date->OldDays() + m_field->GetMDates(0,4), ww_grubning, false );
00563                 break;
00564         case ww_grubning:
00565                 if ( m_ev->m_lock || m_farm->DoIt( 10 )) {
00566                   if (!m_farm->DeepPlough( m_field, 0.0, m_field->GetMDates(1,4) - g_date->DayInYear())) {
00567                         SimpleEvent( g_date->Date() + 1, ww_grubning, true );
00568                         break;
00569                   }
00570                 }
00571                 done=true;
00572                 // So we are done, and somewhere else the farmer will queue up the start event of the next crop
00573                 // END OF MAIN THREAD
00574                 break;
00575         default:
00576                 g_msg->Warn( WARN_BUG, "WinterWheat::Do(): "
00577                          "Unknown event type! ", "" );
00578                 exit( 1 );
00579         }
00580     return done;
00581 }
```

---

The documentation for this class was generated from the following files:

- WinterWheat.H- WinterWheat.cpp

---

Generated on Thu Jan 22 14:13:48 2009 for ALMaSS ODDox by 
 1.5.6 
