# Supplementary material for: Predation and fragmentation portrayed in the statistical structure of prey time series
Source: BMC Ecol. 2009 May 6;9:10. doi: 10.1186/1472-6785-9-10 (PMC2689204; doi:10.1186/1472-6785-9-10)
Supplement: Additional file 2 — Voles and related classes ODDox Documentation. ODDox documentation of the agent-based model (ALMaSS) applied by Hendrichsen et al. The documentation is started by activating main.html. [file 1472-6785-9-10-S2.zip › Vole_ODDox/croprotation_8cpp.html]

ALMaSS ODDox: croprotation.cpp File Reference

- Main Page
- Related Pages
- Classes
- Files

# croprotation.cpp File Reference

`#include <cstdio>`  
`#include <math.h>`  
`#include <string>`  
`#include "configurator.h"`  
`#include "maperrormsg.h"`  
`#include "plants.h"`  
`#include "croprotation.h"`  

|  |
| --- |
|  |
| Defines | |
| #define | \_CRT\_SECURE\_NO\_DEPRECATE |
| Functions | |
| CfgStr | l\_map\_rotation\_files\_prefix ("MAP\_ROTATION\_FILES\_PREFIX", CFG\_CUSTOM,"") |
| Variables | |
| class CropRotation \* | g\_rotation |

---

## Define Documentation

|  |
| --- |
| #define \_CRT\_SECURE\_NO\_DEPRECATE |

---

## Function Documentation

|  |  |  |  |
| --- | --- | --- | --- |
| CfgStr l\_map\_rotation\_files\_prefix | ( | "MAP\_ROTATION\_FILES\_PREFIX" | , |
|  |  | CFG\_CUSTOM | , |
|  |  | "" |  |  |
|  | ) |  |  |  |

Referenced by CropRotation::CropRotation().

---

## Variable Documentation

|  |
| --- |
| class CropRotation\* g\_rotation |

---

Generated on Thu Jan 22 14:13:45 2009 for ALMaSS ODDox by 
 1.5.6 
