# Supplementary material for: Predation and fragmentation portrayed in the statistical structure of prey time series
Source: BMC Ecol. 2009 May 6;9:10. doi: 10.1186/1472-6785-9-10 (PMC2689204; doi:10.1186/1472-6785-9-10)
Supplement: Additional file 2 — Voles and related classes ODDox Documentation. ODDox documentation of the agent-based model (ALMaSS) applied by Hendrichsen et al. The documentation is started by activating main.html. [file 1472-6785-9-10-S2.zip › Vole_ODDox/croprotation_8h.html]

ALMaSS ODDox: croprotation.h File Reference

- Main Page
- Related Pages
- Classes
- Files

# croprotation.h File Reference

`#include <vector>`  
`#include "tov_declaration.h"`  

|  |
| --- |
|  |
| Classes | |
| class | CropRotation |
| struct | Rotation |
| struct | Starter |
| Variables | |
| class CropRotation \* | g\_rotation |
| const int | NoFarmTypes = 2 |

---

## Variable Documentation

|  |
| --- |
| class CropRotation\* g\_rotation |

|  |
| --- |
| const int NoFarmTypes = 2 |

Referenced by CropRotation::CropRotation().

---

Generated on Thu Jan 22 14:13:45 2009 for ALMaSS ODDox by 
 1.5.6 
