# Supplementary material for: Predation and fragmentation portrayed in the statistical structure of prey time series
Source: BMC Ecol. 2009 May 6;9:10. doi: 10.1186/1472-6785-9-10 (PMC2689204; doi:10.1186/1472-6785-9-10)
Supplement: Additional file 2 — Voles and related classes ODDox Documentation. ODDox documentation of the agent-based model (ALMaSS) applied by Hendrichsen et al. The documentation is started by activating main.html. [file 1472-6785-9-10-S2.zip › Vole_ODDox/farmfuncs_8cpp.html]

ALMaSS ODDox: farmfuncs.cpp File Reference

- Main Page
- Related Pages
- Classes
- Files

# farmfuncs.cpp File Reference

---

## Detailed Description

**Farmfuncs.cpp This file contains the source for implementing the farm events**   

by Frank Nikolaisen & Chris J. Topping   
Initial version of June 2003, but under continual change.   
All rights reserved.   
  
Doxygen formatted comments in July 2008   

`#include "ls.h"`  

|  |
| --- |
|  |
| Defines | |
| #define | DO\_IT\_PROB   (l\_farm\_event\_do\_it\_prob.value()) |
| #define | UNREFERENCED\_PARAMETER(P)   (P) |
| Functions | |
| static CfgFloat | l\_farm\_cattle\_veg\_reduce ("FARM\_CATTLE\_VEG\_REDUCE", CFG\_PRIVATE, 0.10) |
| static CfgFloat | l\_farm\_cattle\_veg\_reduce2 ("FARM\_CATTLE\_VEG\_REDUCE\_LOW", CFG\_PRIVATE, 0.025) |
| static CfgInt | l\_farm\_event\_do\_it\_prob ("FARM\_EVENT\_DO\_IT\_PROB", CFG\_PRIVATE, 50) |
| static CfgBool | l\_farm\_herbicide\_kills ("FARM\_PESTICIDE\_KILLS", CFG\_CUSTOM, true) |
| static CfgBool | l\_farm\_insecticide\_kills ("FARM\_INSECTICIDE\_KILLS", CFG\_CUSTOM, true) |
| static CfgFloat | l\_farm\_pig\_veg\_reduce ("FARM\_PIG\_VEG\_REDUCE", CFG\_PRIVATE, 0.98) |
| Variables | |
| Landscape \* | g\_landscape\_p |
| CfgFloat | l\_pest\_insecticide\_amount |
| CfgFloat | l\_pest\_product\_0\_amount |
| CfgFloat | l\_pest\_product\_1\_amount |
| CfgInt | l\_pest\_productapplic\_period |
| CfgInt | l\_pest\_productapplic\_startdate |

---

## Define Documentation

|  |
| --- |
| #define DO\_IT\_PROB   (l\_farm\_event\_do\_it\_prob.value()) |

Referenced by Farm::AutumnHarrow(), Farm::AutumnPlough(), Farm::AutumnRoll(), Farm::AutumnSow(), Farm::BurnStrawStubble(), Farm::CattleOut(), Farm::CattleOutLowGrazing(), Farm::CutToHay(), Farm::CutToSilage(), Farm::CutWeeds(), Farm::DeepPlough(), Farm::FA\_AmmoniumSulphate(), Farm::FA\_GreenManure(), Farm::FA\_Manure(), Farm::FA\_NPK(), Farm::FA\_PK(), Farm::FA\_Sludge(), Farm::FA\_Slurry(), Farm::FP\_GreenManure(), Farm::FP\_LiquidNH3(), Farm::FP\_ManganeseSulphate(), Farm::FP\_Manure(), Farm::FP\_NPK(), Farm::FP\_NPKS(), Farm::FP\_PK(), Farm::FP\_Sludge(), Farm::FP\_Slurry(), Farm::FungicideTreat(), Farm::GrowthRegulator(), Farm::Harvest(), Farm::HayBailing(), Farm::HayTurning(), Farm::HerbicideTreat(), Farm::HillingUp(), Farm::InsecticideTreat(), Farm::Molluscicide(), Farm::PigsOut(), Farm::RowCultivation(), Farm::SpringHarrow(), Farm::SpringPlough(), Farm::SpringRoll(), Farm::SpringSow(), Farm::StrawChopping(), Farm::Strigling(), Farm::StriglingSow(), Farm::StubbleHarrowing(), Farm::Swathing(), Farm::Water(), and Farm::WinterPlough().

|  |  |  |  |  |  |
| --- | --- | --- | --- | --- | --- |
| #define UNREFERENCED\_PARAMETER | ( | P |  | ) | (P) |

Referenced by Farm::AutumnHarrow(), Farm::AutumnPlough(), Farm::AutumnRoll(), Farm::AutumnSow(), Farm::BurnStrawStubble(), Farm::CattleIsOut(), Farm::CattleIsOutLow(), Farm::CattleOut(), Farm::CattleOutLowGrazing(), Farm::CutToHay(), Farm::CutToSilage(), Farm::CutWeeds(), Farm::DeepPlough(), Farm::FA\_AmmoniumSulphate(), Farm::FA\_GreenManure(), Farm::FA\_Manure(), Farm::FA\_NPK(), Farm::FA\_PK(), Farm::FA\_Sludge(), Farm::FA\_Slurry(), Farm::FP\_GreenManure(), Farm::FP\_LiquidNH3(), Farm::FP\_ManganeseSulphate(), Farm::FP\_Manure(), Farm::FP\_NPK(), Farm::FP\_NPKS(), Farm::FP\_PK(), Farm::FP\_Sludge(), Farm::FP\_Slurry(), Farm::FungicideTreat(), Farm::Glyphosate(), Farm::GrowthRegulator(), Farm::Harvest(), Farm::HayBailing(), Farm::HayTurning(), Farm::HerbicideTreat(), Farm::HillingUp(), Farm::InsecticideTreat(), Farm::Molluscicide(), Farm::PigsAreOut(), Farm::PigsAreOutForced(), Farm::PigsOut(), Farm::ProductApplication0(), Farm::ProductApplication1(), Farm::RowCultivation(), Farm::SleepAllDay(), Farm::SpringHarrow(), Farm::SpringPlough(), Farm::SpringRoll(), Farm::SpringSow(), Farm::StrawChopping(), Farm::Strigling(), Farm::StriglingSow(), Farm::StubbleHarrowing(), Farm::Swathing(), Farm::SynInsecticideTreat(), Farm::Trial\_Control(), Farm::Trial\_InsecticideTreat(), Farm::Trial\_ToxicControl(), Farm::Water(), and Farm::WinterPlough().

---

## Function Documentation

|  |  |  |  |
| --- | --- | --- | --- |
| static CfgFloat l\_farm\_cattle\_veg\_reduce | ( | "FARM\_CATTLE\_VEG\_REDUCE" | , |
|  |  | CFG\_PRIVATE | , |
|  |  | 0. | *10* |  |
|  | ) |  |  | `[static]` |

Referenced by Farm::CattleIsOut(), and Farm::CattleOut().

|  |  |  |  |
| --- | --- | --- | --- |
| static CfgFloat l\_farm\_cattle\_veg\_reduce2 | ( | "FARM\_CATTLE\_VEG\_REDUCE\_LOW" | , |
|  |  | CFG\_PRIVATE | , |
|  |  | 0. | *025* |  |
|  | ) |  |  | `[static]` |

Referenced by Farm::CattleIsOutLow(), and Farm::CattleOutLowGrazing().

|  |  |  |  |
| --- | --- | --- | --- |
| static CfgInt l\_farm\_event\_do\_it\_prob | ( | "FARM\_EVENT\_DO\_IT\_PROB" | , |
|  |  | CFG\_PRIVATE | , |
|  |  | 50 |  |  |
|  | ) |  |  | `[static]` |

|  |  |  |  |
| --- | --- | --- | --- |
| static CfgBool l\_farm\_herbicide\_kills | ( | "FARM\_PESTICIDE\_KILLS" | , |
|  |  | CFG\_CUSTOM | , |
|  |  | true |  |  |
|  | ) |  |  | `[static]` |

Referenced by Farm::HerbicideTreat().

|  |  |  |  |
| --- | --- | --- | --- |
| static CfgBool l\_farm\_insecticide\_kills | ( | "FARM\_INSECTICIDE\_KILLS" | , |
|  |  | CFG\_CUSTOM | , |
|  |  | true |  |  |
|  | ) |  |  | `[static]` |

Referenced by Farm::InsecticideTreat(), Farm::ProductApplication0(), Farm::ProductApplication1(), and Farm::SynInsecticideTreat().

|  |  |  |  |
| --- | --- | --- | --- |
| static CfgFloat l\_farm\_pig\_veg\_reduce | ( | "FARM\_PIG\_VEG\_REDUCE" | , |
|  |  | CFG\_PRIVATE | , |
|  |  | 0. | *98* |  |
|  | ) |  |  | `[static]` |

Referenced by Farm::PigsAreOutForced(), and Farm::PigsOut().

---

## Variable Documentation

|  |
| --- |
| Landscape\* g\_landscape\_p |

|  |
| --- |
| CfgFloat l\_pest\_insecticide\_amount |

|  |
| --- |
| CfgFloat l\_pest\_product\_0\_amount |

Referenced by Farm::ProductApplication0().

|  |
| --- |
| CfgFloat l\_pest\_product\_1\_amount |

Referenced by Farm::ProductApplication1().

|  |
| --- |
| CfgInt l\_pest\_productapplic\_period |

|  |
| --- |
| CfgInt l\_pest\_productapplic\_startdate |

---

Generated on Thu Jan 22 14:13:45 2009 for ALMaSS ODDox by 
 1.5.6 
