# Supplementary material for: Predation and fragmentation portrayed in the statistical structure of prey time series
Source: BMC Ecol. 2009 May 6;9:10. doi: 10.1186/1472-6785-9-10 (PMC2689204; doi:10.1186/1472-6785-9-10)
Supplement: Additional file 2 — Voles and related classes ODDox Documentation. ODDox documentation of the agent-based model (ALMaSS) applied by Hendrichsen et al. The documentation is started by activating main.html. [file 1472-6785-9-10-S2.zip › Vole_ODDox/farm_8cpp.html]

ALMaSS ODDox: farm.cpp File Reference

- Main Page
- Related Pages
- Classes
- Files

# farm.cpp File Reference

---

## Detailed Description

**Farm.cpp This file contains the source for the Farm class**   

by Frank Nikolaisen & Chris J. Topping   
Version of June 2003   
All rights reserved.   
  
Doxygen formatted comments in July 2008   

`#include <vector>`  
`#include "ALMaSS_Setup.h"`  
`#include "ls.h"`  
`#include "cropprogs/Carrots.h"`  
`#include "cropprogs/CloverGrassGrazed1.h"`  
`#include "cropprogs/CloverGrassGrazed2.h"`  
`#include "cropprogs/FieldPeas.h"`  
`#include "cropprogs/Fodderbeet.h"`  
`#include "cropprogs/Maize.h"`  
`#include "cropprogs/OBarleyPeaCloverGrass.h"`  
`#include "cropprogs/OSBarleySilage.h"`  
`#include "cropprogs/OCarrots.h"`  
`#include "cropprogs/OCloverGrassGrazed1.h"`  
`#include "cropprogs/OCloverGrassGrazed2.h"`  
`#include "cropprogs/OCloverGrassSilage1.h"`  
`#include "cropprogs/OFirstYearDanger.h"`  
`#include "cropprogs/OGrazingPigs.h"`  
`#include "cropprogs/OPermanentGrassGrazed.h"`  
`#include "cropprogs/OSpringBarleyPigs.h"`  
`#include "cropprogs/OFieldPeas.h"`  
`#include "cropprogs/OFieldPeasSilage.h"`  
`#include "cropprogs/OOats.h"`  
`#include "cropprogs/Oats.h"`  
`#include "cropprogs/OPotatoes.h"`  
`#include "cropprogs/OSpringBarley.h"`  
`#include "cropprogs/OWinterBarley.h"`  
`#include "cropprogs/OWinterRape.h"`  
`#include "cropprogs/OWinterRye.h"`  
`#include "cropprogs/OWinterWheatUndersown.h"`  
`#include "cropprogs/PermanentGrassGrazed.h"`  
`#include "cropprogs/PermanentGrassLowGrazed.h"`  
`#include "cropprogs/PermanentSetAside.h"`  
`#include "cropprogs/Potatoes.h"`  
`#include "cropprogs/seedgrass1.h"`  
`#include "cropprogs/seedgrass2.h"`  
`#include "cropprogs/setaside.h"`  
`#include "cropprogs/SpringBarley.h"`  
`#include "cropprogs/SpringBarleyCloverGrass.h"`  
`#include "cropprogs/SpringBarleySeed.h"`  
`#include "cropprogs/SpringBarleySilage.h"`  
`#include "cropprogs/Triticale.h"`  
`#include "cropprogs/WinterBarley.h"`  
`#include "cropprogs/winterrape.h"`  
`#include "cropprogs/WinterRye.h"`  
`#include "cropprogs/WinterWheat.h"`  
`#include "cropprogs/WWheatPControl.h"`  
`#include "cropprogs/WWheatPTreatment.h"`  
`#include "cropprogs/WWheatPToxicControl.h"`  
`#include "cropprogs/AgroChemIndustryCereal.h"`  
`#include "cropprogs/WinterWheatStrigling.h"`  
`#include "cropprogs/WinterWheatStriglingCulm.h"`  
`#include "cropprogs/WinterWheatStriglingSingle.h"`  
`#include "cropprogs/SpringBarleyCloverGrassStrigling.h"`  
`#include "cropprogs/SpringBarleyStrigling.h"`  
`#include "cropprogs/SpringBarleyStriglingCulm.h"`  
`#include "cropprogs/SpringBarleyStriglingSingle.h"`  
`#include "cropprogs/MaizeStrigling.h"`  
`#include "cropprogs/WinterRapeStrigling.h"`  
`#include "cropprogs/WinterRyeStrigling.h"`  
`#include "cropprogs/WinterBarleyStrigling.h"`  
`#include "cropprogs/FieldPeasStrigling.h"`  
`#include "cropprogs/SpringBarleyPeaCloverGrassStrigling.h"`  
`#include "cropprogs/YoungForest.h"`  
`#include "map_cfg.h"`  

|  |
| --- |
|  |
| Defines | |
| #define | \_CRT\_SECURE\_NO\_DEPRECATE |
| #define | \_CRT\_SECURE\_NO\_DEPRECATE |
| #define | \_CRTDBG\_MAP\_ALLOC |
| Functions | |
| CfgBool | cfg\_organic\_extensive ("FARM\_ORGANIC\_EXTENSIVE", CFG\_CUSTOM, false) |
| Variables | |
| Landscape \* | g\_landscape\_p |

---

## Define Documentation

|  |
| --- |
| #define \_CRT\_SECURE\_NO\_DEPRECATE |

|  |
| --- |
| #define \_CRT\_SECURE\_NO\_DEPRECATE |

|  |
| --- |
| #define \_CRTDBG\_MAP\_ALLOC |

---

## Function Documentation

|  |  |  |  |
| --- | --- | --- | --- |
| CfgBool cfg\_organic\_extensive | ( | "FARM\_ORGANIC\_EXTENSIVE" | , |
|  |  | CFG\_CUSTOM | , |
|  |  | false |  |  |
|  | ) |  |  |  |

---

## Variable Documentation

|  |
| --- |
| Landscape\* g\_landscape\_p |

Referenced by Farm::AutumnHarrow(), Farm::AutumnPlough(), Farm::AutumnRoll(), Farm::AutumnSow(), Farm::BurnStrawStubble(), Farm::CattleIsOut(), Farm::CattleIsOutLow(), Farm::CattleOut(), Farm::CattleOutLowGrazing(), Farm::CutToHay(), Farm::CutToSilage(), Farm::CutWeeds(), Farm::DeepPlough(), Farm::FA\_AmmoniumSulphate(), Farm::FA\_GreenManure(), Farm::FA\_Manure(), Farm::FA\_NPK(), Farm::FA\_PK(), Farm::FA\_Sludge(), Farm::FA\_Slurry(), Farm::FP\_GreenManure(), Farm::FP\_LiquidNH3(), Farm::FP\_ManganeseSulphate(), Farm::FP\_Manure(), Farm::FP\_NPK(), Farm::FP\_NPKS(), Farm::FP\_PK(), Farm::FP\_Sludge(), Farm::FP\_Slurry(), Farm::Harvest(), Farm::HayBailing(), Farm::HayTurning(), Farm::HillingUp(), Farm::PigsAreOut(), Farm::PigsAreOutForced(), Farm::PigsOut(), Farm::RowCultivation(), Farm::SleepAllDay(), Farm::SpringHarrow(), Farm::SpringPlough(), Farm::SpringRoll(), Farm::SpringSow(), Farm::StrawChopping(), Farm::Strigling(), Farm::StriglingSow(), Farm::StubbleHarrowing(), Farm::Swathing(), Farm::Water(), and Farm::WinterPlough().

---

Generated on Thu Jan 22 14:13:45 2009 for ALMaSS ODDox by 
 1.5.6 
