# Supplementary material for: Predation and fragmentation portrayed in the statistical structure of prey time series
Source: BMC Ecol. 2009 May 6;9:10. doi: 10.1186/1472-6785-9-10 (PMC2689204; doi:10.1186/1472-6785-9-10)
Supplement: Additional file 2 — Voles and related classes ODDox Documentation. ODDox documentation of the agent-based model (ALMaSS) applied by Hendrichsen et al. The documentation is started by activating main.html. [file 1472-6785-9-10-S2.zip › Vole_ODDox/farm_8h.html]

ALMaSS ODDox: farm.h File Reference

- Main Page
- Related Pages
- Classes
- Files

# farm.h File Reference

---

## Detailed Description

**Farm.h This file contains the headers for the Farm class**   

by Frank Nikolaisen & Chris J. Topping   
Version of June 2003   
All rights reserved.   
  
Doxygen formatted comments in July 2008   

`#include <vector>`  
`#include "lowqueue.h"`  
`#include "tov_declaration.h"`  

|  |
| --- |
|  |
| Classes | |
| class | AgroChemIndustryCerealFarm1 |
|  | Inbuilt special purpose farm type. More... |
| class | AgroChemIndustryCerealFarm2 |
|  | Inbuilt special purpose farm type. More... |
| class | AgroChemIndustryCerealFarm3 |
|  | Inbuilt special purpose farm type. More... |
| class | ConventionalCattle |
|  | Inbuilt farm type. More... |
| class | ConventionalPig |
|  | Inbuilt farm type. More... |
| class | ConventionalPlant |
|  | Inbuilt farm type. More... |
| class | ConvMarginalJord |
|  | Inbuilt special purpose farm type. More... |
| class | Crop |
|  | The base class for all crops. More... |
| class | Farm |
| struct | FarmEvent |
|  | A struct to hold the information required to trigger a farm event. More... |
| class | NoPesticideBaseFarm |
|  | Inbuilt special purpose farm type. More... |
| class | NoPesticideNoPFarm |
|  | Inbuilt special purpose farm type. More... |
| class | OrganicCattle |
|  | A farm that can have its rotation defined by the user at runtime. More... |
| class | OrganicPig |
|  | A farm that can have its rotation defined by the user at runtime. More... |
| class | OrganicPlant |
|  | A farm that can have its rotation defined by the user at runtime. More... |
| class | PesticideTrialControl |
|  | Inbuilt special purpose farm type. More... |
| class | PesticideTrialToxicControl |
|  | Inbuilt special purpose farm type. More... |
| class | PesticideTrialTreatment |
|  | Inbuilt special purpose farm type. More... |
| class | UserDefinedFarm1 |
|  | A farm that can have its rotation defined by the user at runtime. More... |
| class | UserDefinedFarm10 |
|  | A farm that can have its rotation defined by the user at runtime. More... |
| class | UserDefinedFarm11 |
|  | A farm that can have its rotation defined by the user at runtime. More... |
| class | UserDefinedFarm12 |
|  | A farm that can have its rotation defined by the user at runtime. More... |
| class | UserDefinedFarm13 |
|  | A farm that can have its rotation defined by the user at runtime. More... |
| class | UserDefinedFarm14 |
|  | A farm that can have its rotation defined by the user at runtime. More... |
| class | UserDefinedFarm15 |
|  | A farm that can have its rotation defined by the user at runtime. More... |
| class | UserDefinedFarm16 |
|  | A farm that can have its rotation defined by the user at runtime. More... |
| class | UserDefinedFarm2 |
|  | A farm that can have its rotation defined by the user at runtime. More... |
| class | UserDefinedFarm3 |
|  | A farm that can have its rotation defined by the user at runtime. More... |
| class | UserDefinedFarm4 |
|  | A farm that can have its rotation defined by the user at runtime. More... |
| class | UserDefinedFarm5 |
|  | A farm that can have its rotation defined by the user at runtime. More... |
| class | UserDefinedFarm6 |
|  | A farm that can have its rotation defined by the user at runtime. More... |
| class | UserDefinedFarm7 |
|  | A farm that can have its rotation defined by the user at runtime. More... |
| class | UserDefinedFarm8 |
|  | A farm that can have its rotation defined by the user at runtime. More... |
| class | UserDefinedFarm9 |
|  | A farm that can have its rotation defined by the user at runtime. More... |
| Defines | |
| #define | PROG\_START   1 |
| Enumerations | |
| enum | TTypesOfFarm {     tof\_ConventionalCattle = 0, tof\_ConventionalPig, tof\_ConventionalPlant, tof\_OrganicCattle,     tof\_OrganicPig, tof\_OrganicPlant, tof\_PTrialControl, tof\_PTrialTreatment,     tof\_PTrialToxicControl, tof\_ConvMarginalJord, tof\_AgroChemIndustryCerealFarm1, tof\_AgroChemIndustryCerealFarm2,     tof\_AgroChemIndustryCerealFarm3, tof\_NoPesticideBase, tof\_NoPesticideNoP, tof\_UserDefinedFarm1,     tof\_UserDefinedFarm2, tof\_UserDefinedFarm3, tof\_UserDefinedFarm4, tof\_UserDefinedFarm5,     tof\_UserDefinedFarm6, tof\_UserDefinedFarm7, tof\_UserDefinedFarm8, tof\_UserDefinedFarm9,     tof\_UserDefinedFarm10, tof\_UserDefinedFarm11, tof\_UserDefinedFarm12, tof\_UserDefinedFarm13,     tof\_UserDefinedFarm14, tof\_UserDefinedFarm15, tof\_UserDefinedFarm16   } |

---

## Define Documentation

|  |
| --- |
| #define PROG\_START   1 |

Referenced by Farm::HandleEvents(), and Farm::InitiateManagement().

---

## Enumeration Type Documentation

|  |
| --- |
| enum TTypesOfFarm |

**Enumerator:**
:   |  |  |
    | --- | --- |
    | *tof\_ConventionalCattle* |  |
    | *tof\_ConventionalPig* |  |
    | *tof\_ConventionalPlant* |  |
    | *tof\_OrganicCattle* |  |
    | *tof\_OrganicPig* |  |
    | *tof\_OrganicPlant* |  |
    | *tof\_PTrialControl* |  |
    | *tof\_PTrialTreatment* |  |
    | *tof\_PTrialToxicControl* |  |
    | *tof\_ConvMarginalJord* |  |
    | *tof\_AgroChemIndustryCerealFarm1* |  |
    | *tof\_AgroChemIndustryCerealFarm2* |  |
    | *tof\_AgroChemIndustryCerealFarm3* |  |
    | *tof\_NoPesticideBase* |  |
    | *tof\_NoPesticideNoP* |  |
    | *tof\_UserDefinedFarm1* |  |
    | *tof\_UserDefinedFarm2* |  |
    | *tof\_UserDefinedFarm3* |  |
    | *tof\_UserDefinedFarm4* |  |
    | *tof\_UserDefinedFarm5* |  |
    | *tof\_UserDefinedFarm6* |  |
    | *tof\_UserDefinedFarm7* |  |
    | *tof\_UserDefinedFarm8* |  |
    | *tof\_UserDefinedFarm9* |  |
    | *tof\_UserDefinedFarm10* |  |
    | *tof\_UserDefinedFarm11* |  |
    | *tof\_UserDefinedFarm12* |  |
    | *tof\_UserDefinedFarm13* |  |
    | *tof\_UserDefinedFarm14* |  |
    | *tof\_UserDefinedFarm15* |  |
    | *tof\_UserDefinedFarm16* |  |

```
00123 {
00124   tof_ConventionalCattle=0,
00125   tof_ConventionalPig,
00126   tof_ConventionalPlant,
00127   tof_OrganicCattle,
00128   tof_OrganicPig,
00129   tof_OrganicPlant,
00130   tof_PTrialControl,
00131   tof_PTrialTreatment,
00132   tof_PTrialToxicControl,
00133   tof_ConvMarginalJord,
00134   tof_AgroChemIndustryCerealFarm1, //10
00135   tof_AgroChemIndustryCerealFarm2,
00136   tof_AgroChemIndustryCerealFarm3,
00137   tof_NoPesticideBase,
00138   tof_NoPesticideNoP,
00139   tof_UserDefinedFarm1, //15
00140   tof_UserDefinedFarm2,
00141   tof_UserDefinedFarm3,
00142   tof_UserDefinedFarm4,
00143   tof_UserDefinedFarm5,
00144   tof_UserDefinedFarm6, //20
00145   tof_UserDefinedFarm7,
00146   tof_UserDefinedFarm8,
00147   tof_UserDefinedFarm9,
00148   tof_UserDefinedFarm10,
00149   tof_UserDefinedFarm11,
00150   tof_UserDefinedFarm12,
00151   tof_UserDefinedFarm13,
00152   tof_UserDefinedFarm14,
00153   tof_UserDefinedFarm15,
00154   tof_UserDefinedFarm16
00155 }TTypesOfFarm;
```

---

Generated on Thu Jan 22 14:13:45 2009 for ALMaSS ODDox by 
 1.5.6 
