# Supplementary material for: Predation and fragmentation portrayed in the statistical structure of prey time series
Source: BMC Ecol. 2009 May 6;9:10. doi: 10.1186/1472-6785-9-10 (PMC2689204; doi:10.1186/1472-6785-9-10)
Supplement: Additional file 2 — Voles and related classes ODDox Documentation. ODDox documentation of the agent-based model (ALMaSS) applied by Hendrichsen et al. The documentation is started by activating main.html. [file 1472-6785-9-10-S2.zip › Vole_ODDox/files.html]

ALMaSS ODDox: File Index

- Main Page
- Related Pages
- Classes
- Files

- File List
- File Members

# File List

Here is a list of all files with brief descriptions:

|  |  |
| --- | --- |
| BoostRandomGenerators.h | **BoostRandomGenerators.h Boost headers for using the boost random number generation utilities** |
| croprotation.cpp |  |
| croprotation.h |  |
| farm.cpp | **Farm.cpp This file contains the source for the Farm class** |
| farm.h | **Farm.h This file contains the headers for the Farm class** |
| farmfuncs.cpp | **Farmfuncs.cpp This file contains the source for implementing the farm events** |
| GeneticMaterial.cpp | **GeneticMaterial.cpp This file contains the source for the genetic material classes** |
| GeneticMaterial.H | **GeneticMaterial.h This file contains the headers for the genetic material classes** |
| PopulationManager.cpp | **PopulationManager.cpp This is the code file for the population manager and associated classes** |
| PopulationManager.h | **PopulationManager.h This is the header file for the population manager and associated classes** |
| Predators.cpp | **The main source code for all predator lifestage and population manager classes** |
| Predators.H | **The header file for all predator lifestages and population manager classes** |
| References\_ODDox.h |  |
| setaside.cpp | **Setaside.cpp This file contains the source for the setaside class** |
| setaside.h | **Setaside.h This file contains the headers for the setaside class** |
| tole\_declaration.h |  |
| tov\_declaration.h |  |
| treatment.h |  |
| Vole\_all.cpp | **Vole\_all.cpp This file contains the code for all vole lifestage classes** |
| vole\_all.h | **Vole\_all.h This header file contains the code for all vole lifestage classes** |
| vole\_ODDox.h |  |
| VolePopulationManager.cpp | **VolePopulationManager.cpp This file contains the source for the vole population manager class** |
| VolePopulationManager.H | **VolePopulationManager.h This is the header file for the vole population manager class** |
| WinterWheat.cpp | **WinterWheat.cpp This file contains the source for the WinterWheat class** |
| WinterWheat.H | **WinterWheat.h This file contains the headers for the WinterWheat class** |

---

Generated on Thu Jan 22 14:13:48 2009 for ALMaSS ODDox by 
 1.5.6 
