# Supplementary material for: Predation and fragmentation portrayed in the statistical structure of prey time series
Source: BMC Ecol. 2009 May 6;9:10. doi: 10.1186/1472-6785-9-10 (PMC2689204; doi:10.1186/1472-6785-9-10)
Supplement: Additional file 2 — Voles and related classes ODDox Documentation. ODDox documentation of the agent-based model (ALMaSS) applied by Hendrichsen et al. The documentation is started by activating main.html. [file 1472-6785-9-10-S2.zip › Vole_ODDox/functions_0x64.html]

ALMaSS ODDox: Class Members

- Main Page
- Related Pages
- Classes
- Files

- Alphabetical List
- Class List
- Class Hierarchy
- Class Members

- All
- Functions
- Variables

- a
- b
- c
- d
- e
- f
- g
- h
- i
- j
- k
- l
- m
- n
- o
- p
- r
- s
- t
- u
- v
- w
- x
- y
- ~

Here is a list of all class members with links to the classes they belong to:

### - d -

- dec\_inds()
  : TPredator\_Population\_Manager- DecideQualityAction()
    : Vole\_Male- DeepPlough()
      : Farm- DetermineTerritorySize()
        : Vole\_Male- Dispersal()
          : Vole\_Male
          , Vole\_Female- DisplayLocations()
            : Population\_Manager- Do()
              : Crop
              , WinterWheat
              , SetAside- DoAfter()
                : Population\_Manager
                , TPredator\_Population\_Manager- DoAlmostLast()
                  : Population\_Manager- DoBefore()
                    : TPredator\_Population\_Manager
                    , Population\_Manager- DoFirst()
                      : Population\_Manager
                      , TPredator\_Population\_Manager
                      , Vole\_Population\_Manager- DoIt()
                        : Farm- DoLast()
                          : Population\_Manager
                          , TPredator\_Population\_Manager- DoWalking()
                            : Vole\_Base- DoWalkingCorrect()
                              : Vole\_Base- Dying()
                                : TAnimal

---

Generated on Thu Jan 22 14:13:45 2009 for ALMaSS ODDox by 
 1.5.6 
