# Supplementary material for: Predation and fragmentation portrayed in the statistical structure of prey time series
Source: BMC Ecol. 2009 May 6;9:10. doi: 10.1186/1472-6785-9-10 (PMC2689204; doi:10.1186/1472-6785-9-10)
Supplement: Additional file 2 — Voles and related classes ODDox Documentation. ODDox documentation of the agent-based model (ALMaSS) applied by Hendrichsen et al. The documentation is started by activating main.html. [file 1472-6785-9-10-S2.zip › Vole_ODDox/functions_0x67.html]

ALMaSS ODDox: Class Members

- Main Page
- Related Pages
- Classes
- Files

- Alphabetical List
- Class List
- Class Hierarchy
- Class Members

- All
- Functions
- Variables

- a
- b
- c
- d
- e
- f
- g
- h
- i
- j
- k
- l
- m
- n
- o
- p
- r
- s
- t
- u
- v
- w
- x
- y
- ~

Here is a list of all class members with links to the classes they belong to:

### - g -

- Genes
  : struct\_Vole\_Adult- GeneticMaterial()
    : GeneticMaterial- GeneticMaterial1616()
      : GeneticMaterial1616- GeneticsResultsOutput()
        : Population\_Manager
        , Vole\_Population\_Manager- GetAllele()
          : GeneticMaterial
          , GeneticMaterial1616- GetDirectFlag()
            : Vole\_Base
            , GeneticMaterial
            , GeneticMaterial1616- GetFarmNumber()
              : Farm- GetFertile()
                : Vole\_Male- GetFirstCrop()
                  : CropRotation- GetFirstCropIndex()
                    : Farm- GetFirstDate()
                      : Farm
                      , Crop- GetGeneticFlag()
                        : Vole\_Base
                        , GeneticMaterial
                        , GeneticMaterial1616- GetIntensity()
                          : Farm- GetLocation()
                            : Vole\_Female
                            , Vole\_Base
                            , Vole\_Male- GetNextCrop()
                              : CropRotation- GetNextCropIndex()
                                : Farm- GetNextCropStartDate()
                                  : Farm- GetType()
                                    : Farm- Glyphosate()
                                      : Farm- gridcount
                                        : Population\_Manager- GrowthRegulator()
                                          : Farm

---

Generated on Thu Jan 22 14:13:45 2009 for ALMaSS ODDox by 
 1.5.6 
