# Supplementary material for: Predation and fragmentation portrayed in the statistical structure of prey time series
Source: BMC Ecol. 2009 May 6;9:10. doi: 10.1186/1472-6785-9-10 (PMC2689204; doi:10.1186/1472-6785-9-10)
Supplement: Additional file 2 — Voles and related classes ODDox Documentation. ODDox documentation of the agent-based model (ALMaSS) applied by Hendrichsen et al. The documentation is started by activating main.html. [file 1472-6785-9-10-S2.zip › Vole_ODDox/functions_0x6d.html]

ALMaSS ODDox: Class Members

- Main Page
- Related Pages
- Classes
- Files

- Alphabetical List
- Class List
- Class Hierarchy
- Class Members

- All
- Functions
- Variables

- a
- b
- c
- d
- e
- f
- g
- h
- i
- j
- k
- l
- m
- n
- o
- p
- r
- s
- t
- u
- v
- w
- x
- y
- ~

Here is a list of all class members with links to the classes they belong to:

### - m -

- m\_Age
  : RoeDeerInfo
  , Vole\_Base- m\_agrochemindustrycereal
    : Farm- m\_AlleleFreqsFile
      : Population\_Manager- m\_BornLastYear
        : Vole\_Female- m\_carrots
          : Farm- m\_catastrophestartyear
            : Population\_Manager- m\_CGG1
              : Farm- m\_CGG2
                : Farm- m\_count
                  : Crop- m\_DaysUntilBirth
                    : Vole\_Female- m\_dflag
                      : struct\_Vole\_Adult- m\_DispersalMax
                        : TPredator- m\_DispVector
                          : Vole\_Base- m\_EasyPopRes
                            : Population\_Manager- m\_EleType
                              : AnimalPosition- m\_ev
                                : Crop- m\_event
                                  : FarmEvent- m\_f1sterilitychance
                                    : Vole\_Population\_Manager- m\_FailureCount
                                      : TPredator- m\_farm
                                        : Crop- m\_farm\_num
                                          : Farm- m\_farmtype
                                            : Farm- m\_fertile
                                              : Vole\_Male- m\_field
                                                : FarmEvent
                                                , Crop- m\_fieldpeas
                                                  : Farm- m\_fieldpeasstrigling
                                                    : Farm- m\_fields
                                                      : Farm- m\_first\_date
                                                        : Crop- m\_first\_year
                                                          : FarmEvent- m\_flag
                                                            : struct\_Vole\_Adult- m\_fodderbeet
                                                              : Farm- m\_geneticimpacted
                                                                : Vole\_Population\_Manager- m\_geneticproductfertilityeffect
                                                                  : Vole\_Population\_Manager- m\_GeneticsFile
                                                                    : Population\_Manager- m\_geneticsterilitychance
                                                                      : Vole\_Population\_Manager- m\_gflag
                                                                        : struct\_Vole\_Adult- m\_GrowthStartDate
                                                                          : Vole\_Population\_Manager- m\_Have\_Territory
                                                                            : Vole\_Base- m\_HaveTerritory
                                                                              : TPredator- m\_HomeRange
                                                                                : TPredator- m\_impacted
                                                                                  : Vole\_Population\_Manager- m\_intensity
                                                                                    : Farm- m\_KillEfficiency
                                                                                      : TPredator- m\_kills\_this\_season
                                                                                        : TPredator- m\_last\_date
                                                                                          : Crop- m\_LifeSpan
                                                                                            : Vole\_Base- m\_Location\_x
                                                                                              : TAnimal- m\_Location\_y
                                                                                                : TAnimal- m\_lock
                                                                                                  : FarmEvent- m\_MainForm
                                                                                                    : Population\_Manager- m\_maize
                                                                                                      : Farm- m\_maizestrigling
                                                                                                        : Farm- m\_Mature
                                                                                                          : Vole\_Base- m\_next\_tov
                                                                                                            : FarmEvent- m\_no\_individuals
                                                                                                              : TPredator\_Population\_Manager- m\_NoAreas
                                                                                                                : probe\_data- m\_NoEleTypes
                                                                                                                  : probe\_data- m\_NoFailuresBeforeDispersal
                                                                                                                    : TPredator- m\_NoFarms
                                                                                                                      : probe\_data- m\_NoOfYoung
                                                                                                                        : Vole\_Female- m\_NoProbes
                                                                                                                          : Population\_Manager- m\_notimpacted
                                                                                                                            : Vole\_Population\_Manager- m\_NoVegTypes
                                                                                                                              : probe\_data- m\_oats
                                                                                                                                : Farm- m\_OBarleyPCG
                                                                                                                                  : Farm- m\_ocarrots
                                                                                                                                    : Farm- m\_OCGG1
                                                                                                                                      : Farm- m\_OCGG2
                                                                                                                                        : Farm- m\_OCGS1
                                                                                                                                          : Farm- m\_ofieldpeas
                                                                                                                                            : Farm- m\_ofieldpeassilage
                                                                                                                                              : Farm- m\_ofirstyeardanger
                                                                                                                                                : Farm- m\_ograzingpigs
                                                                                                                                                  : Farm- m\_OldRange\_x
                                                                                                                                                    : RoeDeerInfo- m\_OldRange\_y
                                                                                                                                                      : RoeDeerInfo- m\_ooats
                                                                                                                                                        : Farm- m\_opermgrassgrazed
                                                                                                                                                          : Farm- m\_opotatoes
                                                                                                                                                            : Farm- m\_OSBarleysilage
                                                                                                                                                              : Farm- m\_ospringbarley
                                                                                                                                                                : Farm- m\_ospringbarleypigs
                                                                                                                                                                  : Farm- m\_OurLandscape
                                                                                                                                                                    : TAnimal- m\_OurPopulation
                                                                                                                                                                      : Vole\_Base- m\_OurPopulationManager
                                                                                                                                                                        : TPredator- m\_owinterbarley
                                                                                                                                                                          : Farm- m\_owinterrape
                                                                                                                                                                            : Farm- m\_owinterrye
                                                                                                                                                                              : Farm- m\_owinterwheatundersown
                                                                                                                                                                                : Farm- m\_permanentsetaside
                                                                                                                                                                                  : Farm- m\_permgrassgrazed
                                                                                                                                                                                    : Farm- m\_permgrasslowgrazed
                                                                                                                                                                                      : Farm- m\_pesticide\_accumulation
                                                                                                                                                                                        : Vole\_Female- m\_pesticideInfluenced
                                                                                                                                                                                          : Vole\_Base- m\_pesticideInfluenced2
                                                                                                                                                                                            : Vole\_Base- m\_potatoes
                                                                                                                                                                                              : Farm- m\_Pregnant
                                                                                                                                                                                                : Vole\_Female- m\_Prey
                                                                                                                                                                                                  : TPredator
                                                                                                                                                                                                  , TPredator\_Population\_Manager- m\_queue
                                                                                                                                                                                                    : Farm- m\_Range\_x
                                                                                                                                                                                                      : RoeDeerInfo- m\_Range\_y
                                                                                                                                                                                                        : RoeDeerInfo- m\_Rect
                                                                                                                                                                                                          : probe\_data- m\_RefEle
                                                                                                                                                                                                            : probe\_data- m\_RefFarms
                                                                                                                                                                                                              : probe\_data- m\_RefVeg
                                                                                                                                                                                                                : probe\_data- m\_ReportInterval
                                                                                                                                                                                                                  : probe\_data- m\_Reserves
                                                                                                                                                                                                                    : Vole\_Base- m\_rotation
                                                                                                                                                                                                                      : Farm- m\_rotation\_sync\_index
                                                                                                                                                                                                                        : Farm- m\_rots
                                                                                                                                                                                                                          : CropRotation- m\_run
                                                                                                                                                                                                                            : FarmEvent- m\_sbarleyclovergrass
                                                                                                                                                                                                                              : Farm- m\_Search\_x
                                                                                                                                                                                                                                : TPredator- m\_Search\_y
                                                                                                                                                                                                                                  : TPredator- m\_SearchArea
                                                                                                                                                                                                                                    : TPredator- m\_seedgrass1
                                                                                                                                                                                                                                      : Farm- m\_seedgrass2
                                                                                                                                                                                                                                        : Farm- m\_setaside
                                                                                                                                                                                                                                          : Farm- m\_Sex
                                                                                                                                                                                                                                            : Vole\_Base- m\_SimulationName
                                                                                                                                                                                                                                              : Population\_Manager- m\_Size
                                                                                                                                                                                                                                                : RoeDeerInfo- m\_springbarley
                                                                                                                                                                                                                                                  : Farm- m\_springbarleyclovergrassstrigling
                                                                                                                                                                                                                                                    : Farm- m\_springbarleypeaclovergrassstrigling
                                                                                                                                                                                                                                                      : Farm- m\_springbarleyseed
                                                                                                                                                                                                                                                        : Farm- m\_springbarleysilage
                                                                                                                                                                                                                                                          : Farm- m\_springbarleystrigling
                                                                                                                                                                                                                                                            : Farm- m\_springbarleystriglingculm
                                                                                                                                                                                                                                                              : Farm- m\_springbarleystriglingsingle
                                                                                                                                                                                                                                                                : Farm- m\_start
                                                                                                                                                                                                                                                                  : CropRotation- m\_startday
                                                                                                                                                                                                                                                                    : FarmEvent- m\_StarvationDays
                                                                                                                                                                                                                                                                      : Vole\_Base- m\_StepSize
                                                                                                                                                                                                                                                                        : Population\_Manager- m\_stockfarmer
                                                                                                                                                                                                                                                                          : Farm- m\_TargetTypes
                                                                                                                                                                                                                                                                            : probe\_data- m\_TerrRange
                                                                                                                                                                                                                                                                              : Vole\_Base- m\_TheLandscape
                                                                                                                                                                                                                                                                                : Population\_Manager- m\_todo
                                                                                                                                                                                                                                                                                  : FarmEvent- m\_triticale
                                                                                                                                                                                                                                                                                    : Farm- m\_VegType
                                                                                                                                                                                                                                                                                      : AnimalPosition- m\_Weight
                                                                                                                                                                                                                                                                                        : Vole\_Base- m\_winterbarley
                                                                                                                                                                                                                                                                                          : Farm- m\_winterbarleystrigling
                                                                                                                                                                                                                                                                                            : Farm- m\_winterrape
                                                                                                                                                                                                                                                                                              : Farm- m\_winterrapestrigling
                                                                                                                                                                                                                                                                                                : Farm- m\_winterrye
                                                                                                                                                                                                                                                                                                  : Farm- m\_winterryestrigling
                                                                                                                                                                                                                                                                                                    : Farm- m\_winterwheat
                                                                                                                                                                                                                                                                                                      : Farm- m\_winterwheatstrigling
                                                                                                                                                                                                                                                                                                        : Farm- m\_winterwheatstriglingculm
                                                                                                                                                                                                                                                                                                          : Farm- m\_winterwheatstriglingsingle
                                                                                                                                                                                                                                                                                                            : Farm- m\_wwheatpcontrol
                                                                                                                                                                                                                                                                                                              : Farm- m\_wwheatptoxiccontrol
                                                                                                                                                                                                                                                                                                                : Farm- m\_wwheatptreatment
                                                                                                                                                                                                                                                                                                                  : Farm- m\_x
                                                                                                                                                                                                                                                                                                                    : AnimalPosition- m\_x1
                                                                                                                                                                                                                                                                                                                      : rectangle- m\_x2
                                                                                                                                                                                                                                                                                                                        : rectangle- m\_y
                                                                                                                                                                                                                                                                                                                          : AnimalPosition- m\_y1
                                                                                                                                                                                                                                                                                                                            : rectangle- m\_y2
                                                                                                                                                                                                                                                                                                                              : rectangle- m\_YoungAge
                                                                                                                                                                                                                                                                                                                                : Vole\_Female- m\_youngforest
                                                                                                                                                                                                                                                                                                                                  : Farm- MakeStockFarmer()
                                                                                                                                                                                                                                                                                                                                    : PesticideTrialTreatment
                                                                                                                                                                                                                                                                                                                                    , PesticideTrialControl
                                                                                                                                                                                                                                                                                                                                    , Farm
                                                                                                                                                                                                                                                                                                                                    , ConventionalPlant
                                                                                                                                                                                                                                                                                                                                    , OrganicPlant
                                                                                                                                                                                                                                                                                                                                    , PesticideTrialToxicControl- Management()
                                                                                                                                                                                                                                                                                                                                      : Farm- MatesGenes
                                                                                                                                                                                                                                                                                                                                        : Vole\_Female- MList
                                                                                                                                                                                                                                                                                                                                          : Vole\_Population\_Manager- Molluscicide()
                                                                                                                                                                                                                                                                                                                                            : Farm- MortalityTest()
                                                                                                                                                                                                                                                                                                                                              : Vole\_Base- MoveQuality()
                                                                                                                                                                                                                                                                                                                                                : Vole\_Base- MoveTo()
                                                                                                                                                                                                                                                                                                                                                  : Vole\_Base- Mutation\_1()
                                                                                                                                                                                                                                                                                                                                                    : GeneticMaterial- Mutation\_1ab()
                                                                                                                                                                                                                                                                                                                                                      : GeneticMaterial- Mutation\_2()
                                                                                                                                                                                                                                                                                                                                                        : GeneticMaterial- Mutation\_3()
                                                                                                                                                                                                                                                                                                                                                          : GeneticMaterial- MyFile
                                                                                                                                                                                                                                                                                                                                                            : probe\_data- MyFileName
                                                                                                                                                                                                                                                                                                                                                              : probe\_data- MyGenes
                                                                                                                                                                                                                                                                                                                                                                : Vole\_Base

---

Generated on Thu Jan 22 14:13:45 2009 for ALMaSS ODDox by 
 1.5.6 
