# Supplementary material for: Predation and fragmentation portrayed in the statistical structure of prey time series
Source: BMC Ecol. 2009 May 6;9:10. doi: 10.1186/1472-6785-9-10 (PMC2689204; doi:10.1186/1472-6785-9-10)
Supplement: Additional file 2 — Voles and related classes ODDox Documentation. ODDox documentation of the agent-based model (ALMaSS) applied by Hendrichsen et al. The documentation is started by activating main.html. [file 1472-6785-9-10-S2.zip › Vole_ODDox/functions_0x6e.html]

ALMaSS ODDox: Class Members

- Main Page
- Related Pages
- Classes
- Files

- Alphabetical List
- Class List
- Class Hierarchy
- Class Members

- All
- Functions
- Variables

- a
- b
- c
- d
- e
- f
- g
- h
- i
- j
- k
- l
- m
- n
- o
- p
- r
- s
- t
- u
- v
- w
- x
- y
- ~

Here is a list of all class members with links to the classes they belong to:

### - n -

- n
  : IntArray100- NewCrop
    : Rotation- NoAlleles
      : AlleleFreq- NoPesticideBaseFarm()
        : NoPesticideBaseFarm- NoPesticideNoPFarm()
          : NoPesticideNoPFarm- NoPredatorTypes
            : TPredator\_Population\_Manager

---

Generated on Thu Jan 22 14:13:45 2009 for ALMaSS ODDox by 
 1.5.6 
